# Identity of zinc finger nucleases with specificity to herpes simplex virus type II genomic DNA: novel HSV-2 vaccine/therapy precursors

**DOI:** 10.1186/1742-4682-8-23

**Published:** 2011-06-24

**Authors:** Misaki Wayengera

**Affiliations:** 1 Unit of Genetics, Genomics & Theoretical Biology, Dept of Pathology, School of Biomedical Science, College of Health Sciences, Makerere University. P o Box 7072 Kampala, Uganda

## Abstract

**Background:**

Herpes simplex type II (HSV-2) is a member of the family *herpesviridae*. Human infection with this double stranded linear DNA virus causes genital ulcerative disease and existing treatment options only serve to resolve the symptomatology (ulcers) associated with active HSV-2 infection but do not eliminate latent virus. As a result, infection with HSV-2 follows a life-long relapsing (active *versus *latent) course. On the basis of a primitive bacterium anti-phage DNA defense, the restriction modification (R-M) system, we previously identified the *Escherichia coli *restriction enzyme (REase) EcoRII as a novel peptide to excise or irreversibly disrupt latent HSV-2 DNA from infected cells. However, sequences of the site specificity palindrome of EcoRII 5'-CCWGG-3' (W = A or T) are equally present within the human genome and are a potential source of host-genome toxicity. This feature has limited previous HSV-2 EcoRII based therapeutic models to microbicides only, and highlights the need to engineer artificial REases (zinc finger nucleases-ZFNs) with specificity to HSV-2 genomic-DNA only. Herein, the therapeutic-potential of zinc finger arrays (ZFAs) and ZFNs is identified and modeled, with unique specificity to the HSV-2 genome.

**Methods and results:**

Using the whole genome of HSV-2 strain HG52 (Dolan A et *al.*,), and with the ZFN-consortium's CoDA-ZiFiT software pre-set at default, more than 28,000 ZFAs with specificity to HSV-2 DNA were identified. Using computational assembly (through *in-silico *linkage to the Flavobacterium *okeanokoites *endonuclease Fok I of the type IIS class), 684 ZFNs with specificity to the HSV-2 genome, were constructed. Graphic-analysis of the HSV-2 genome-cleavage pattern using the afore-identified ZFNs revealed that the highest cleavage-incidence occurred within the 30,950 base-pairs (~between the genomic context coordinates 0.80 and 1.00) at the 3' end of the HSV-2 genome. At approximately 3,095 bp before and after the 5' and 3' ends of the HSV-2 genome (genomic context coordinates 0.02 and 0.98, respectively) were specificity sites of ZFNs suited for the complete excision of over 60% of HSV-2 genomic material from within infected human cells, through the process of non-homologous end joining (NHEJ). Furthermore, a model concerning a recombinant (ICP10-PK mutant) replication competent HSV-2 viral vector for delivering and transducing a diploid copy (or pair) of the HSV-2-genome-specific ZFN genotype within neuronal tissue, is presented.

**Conclusion:**

ZFNs with specificity to HSV-2 genomic DNA that are precursors of novel host-genome expressed HSV-2 gene-therapeutics or vaccines were identified.

## Background

### Herpes simplex virus type II as a cause of human genital ulcerative disease

Herpes simplex virus types I and II (HSV-1 and 2, respectively), together with the varicella-zoster (chicken-pox) virus, are members of the *herpesviridae *taxonomic family of viruses [1]. Human infection with these largely neuro-tropic viruses can be active or latent [1,2]. Active infection with HSV-1 or HSV-2 leads to ulcerative lesions of the oral or genital mucosa, respectively [3,4]; latent infection with these viral species is largely asymptomatic. Latent HSV-2 infection occurs primarily in neurons of the sacral root ganglia. Therefore, the clinical spectrum of HSV-2 can be said to comprise primary-active infection, followed by resolution and establishment of a lifelong phase of latency [4,5]. Primary HSV-2 infection is characterized by the appearance of blisters or vesicles on the vulva or penis that break to leave shallow, painful ulcerating lesions [6,7]. These ulcers spontaneously heal within 2-3 weeks, although healing can be very slow in immunocompromised patients [7]. Latent-HSV-2 is characterized by recurrent episodes of clinical disease (4-5 per year); the subclinical status intermittent between reactivation episodes can be associated with infectious viral shedding and transmission of HSV-2 in genital secretions. The incidence of symptomatic HSV-2 infections varies geographically but is higher in HIV positive individuals [8-10]. The genital lesions associated with active- HSV-2 infection have become a particular public health concern, as there is evidence that links them to an increased risk of sexually-acquiring or transmitting human immunodeficiency virus type I (HIV-1) [5-8]. Therefore, treatment for HSV-2 and other genital ulcerative diseases [9] is an acceptable measure when considering reducing the risk of HIV-1 sexual transmission and acquisition [10-12].

### Challenges in existing options for the treatment or prevention of HSV-2 acquisition

Existing biomedical interventions for HSV-2 treatment are only applicable to actively replicating HSV-2 particles [13]. This limits their clinical relevance to only treating ulcerative lesions of HSV-2 [14,15], rather than clearing the infection. Specifically, existing chemotherapy options for treating HSV-2 only inhibit actively replicating virus, while latent virus is unaffected. However, the latent HSV-2 is the source of future episodes of genital ulcerations following reactivation during or following a bout of immune suppression caused by a common cold, after infection with HIV or chemotherapy treatment, among others [16,17]. This picture of a lifelong latent infection that can be reactivated presents unique challenges for the biomedical management of HSV-2 infections [18]. There have been substantive efforts to develop an efficacious HSV-2 vaccine based on existing biomedical evidence, and these are on-going [19,20]. Specifically, randomized clinical trials of prior HSV-2 vaccine candidates, comprising single or double-component (gB2 and gD2) recombinant glycoproteins formulated in adjuvants or expressed within live-attenuated replication-incompetent (disable-infectious single cycle-DISC, ICP8 gene mutation) or replication-competent (ICP10 gene mutation) HSV-2 derived viral vectors, have only demonstrated partial efficacy towards the end goal of protecting against the sexual transmission or acquisition of HSV-2 [21-25].

### -Virology of HSV-2 and the concept of pre-integration viral-genome slicing (PRINT-vGSX)

Like the other generic members of the *alphaherpesviridae *subfamily, HSV-2 is a large, enveloped virus with an outer lipid envelope studded with at least 10 viral glycoproteins, an intermediate tegument layer comprising at least 15 viral proteins, and an icosahedral nucleocapsid containing the double-stranded DNA genome [26]. The complete sequenced genome of a strain of HSV-2, HG52 (Dolan *et al. *[27]), reveals that HSV-2 genomic DNA is organized into two unique regions of double-stranded DNA (long 126-kb and short 26-kb short) denoted as U_L _and U_S_. These are bracketed by inverted repeat sequences (TRL-IRL and IRS-TRS) that readily allow isomerization and recombination of the two regions [27,28]. The entire genome has a G+C content of 70.4%, and comprises 84 open reading frames coding approximately 74 proteins that can be grouped into three categories: (i) *immediate-early genes *(whose transcription depends on a virally-encoded activating protein, VP16, and which encode the viral α proteins); (ii) *early genes *(which are turned on by the α proteins and whose products (β proteins) are involved in DNA replication); and (iii) *late genes*, the products of which (γ proteins) are virion structural proteins and proteins required for virus particle assembly and egress. The majority of the viral envelope glycoproteins (gD) are antigenically related to those of HSV-1, whereas gG1 and gG2 are type-specific [27,29]. This array of numerous gene-products, many of which are indispensable for virus growth *in vitro*, underlies the efficacious virulence evolved by HSV-2 towards the evasion of host defenses including preventing apoptosis in the infected host cell, blockade of pathways for interferon-induction and production, and down-regulation of HSV-2 antigen presentation under the context of type I Major-histocompatibility complex (MHC) [30].

On basis of the linear double stranded (*ds*) DNA genomic-structure of HSV-2 [27] and the functioning of the primitive anti-phage defense inherent within bacteria, the restriction modification (R-M) system, the possibility of directly attacking and disrupting or excising HSV-2 genomic DNA has been proposed as an alternative, novel biomedical intervention against HSV-2 [31]. Specifically, we demonstrated that the *Escherichia coli *restriction enzyme (REase)-EcoRII, which cleaves the HSV-2 genome at more than 800, is an ideal precursor for excising sections of genomes of infectious (actively replicating) HSV-2 particles or irreversibly disrupting latent-HSV-2 genomic DNA from within infected cells. Sequences of the site specificity palindrome of EcoRII 5'-CCWGG-3' (W = A or T) were noted to be prevalent within the human-host genome and therefore a source of host-genome toxicity. This finding limited the HSV-2/EcoRII based therapeutic models to microbicides only [32,33], and highlighted the requirement to engineer artificial REases (or zinc finger nucleases - ZFNs) [34-36] with unique specificity to genome sequences of HSV-2 as safe precursors for therapeutic exploration *in-vivo*. Grosse *et al. *[37], recently identified such HSV-1 specific homing (mega-) endonucleases, demonstrating that their expression in African green monkey kidney fibroblast (COS-7) and BSR cells inhibits infection by HSV-1 at low and moderate multiplicities of infection (MOIs), inducing a significant reduction in the viral load. Furthermore, the remaining viral genomes display a high rate of mutation (up to 16%) at the meganuclease cleavage site, consistent with a mechanism of action based on the cleavage of the viral genome. This work highlighted mega (and zinc finger) nucleases as an alternative class of antiviral agent with the potential to address replicative and latent DNA viral forms [37].

In this paper, the identification, assembly and modeling of the *in-vivo *therapeutic potential of zinc finger arrays (ZFAs), ZFNs and viral vectors transducing the ZFN-genotype, with unique specificity to sequences of the HSV-2 genome, are presented for the first time.

## Results

### Identification of HSV-2 genome specific zinc finger array (ZFAs) and zinc finger nucleases (ZFN)

**Figure 1:**
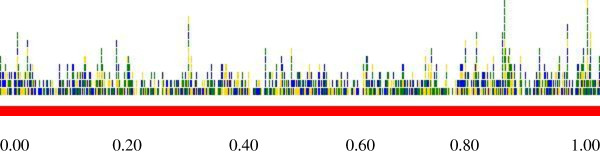
**Schematics of HSV-II genome- cleavage by ZFN**. This figure graphically illustrates the sites and frequency of ZFN cleavage of the HSV-2 genome by the 684 ZFNs identified. Cleavage was present at almost all positions within the HSV-2 genome. Note, however, that the highest incidence of HSV-2 genomic cleavage is situated within the last 30,950 base-pairs (154,784/5: ~between the genomic context coordinates 0.80 and 1.00) at the 3' end of the HSV-2 genome. That said, at approximately 3,094.9 bp (30,950/10) before and after the 5' and 3' ends of the HSV-2 genome (genomic context coordinates 0.02 and 0.98, respectively), there is another array of specificity sites for ZFNs that are potentially best suited for the complete excision of over 60% of HSV-2 genomic material from within infected human cells.

**U**sing the FASTA format of the nucleotide sequences of the whole genome of HSV-2 strain HG52 (Dolan *et al. *[27]) (provided in additional file 1) and CoDA-ZiFiT [34-36], a proprietary computational software of the ZFN-consortium that was pre-set at its default-user options, identification of over 28,000 ZFAs with specificity to HSV-2 genomic DNA (**see **additional file 2) was achieved. Using computational assembly (based on the *in-silico *linkage of the Zif arrays (ZFAs) to the Flavobacterium *okeanokoites *the type II class endonuclease, F_N _(Fok I) as previously achieved *in-vivo *by Kim *et al. *[38]), constructs of 684 ZFNs (hybrid, chimeric Zif-F_N_) with specificity to the HSV-2 genome (see additional file 3) was attained. Throughout the latter experiments, the description of the spacer regions was maintained at 5-7 base-pairs. These ZFNs can bind as dimers to their target HSV-2 DNA sites, with each monomer using its zinc finger domain to recognize a 'half-site' of the targeted DNA sequence. *In-vivo*, dimerization of ZFNs is mediated by the FokI cleavage domain through cleavage of a five or six base pair 'spacer' sequence that separates the two inverted target 'half sites' [34-36]. Importantly, since the DNA-binding specificities of zinc finger domains can be re-engineered using various methods, customized ZFNs can, in principle, be constructed to specifically target almost any gene sequence [34]. Three methods are currently available for engineering zinc finger domains: Context-dependent Assembly (CoDA), Oligomerized Pool Engineering (OPEN), and Modular Assembly [34-36]. Herein, the CoDA approach, using the ZiFiT software based on work by researchers from the Barbas lab [35], was employed for assembly of ZFAs and ZFNs. A list of four ZFNs, inclusive of their -1 to 6 alpha-helical nucleotide binding and recognition domains (F1, F2/F3, F2, F1) alongside the respective site specific sequence within the HSV-2 genome, are presented in table 1. Residues -1 to 6 (numbered relative to the start of the helix) of the alpha-helix of the ZFAs are responsible for the specific recognition of triplets of DNA sequences through the formation of base-specific contacts in the major groove of the double-stranded target DNA [34-36]. Therefore, residues -1 to 6 within the ZFs' alpha helixes are denoted as 'recognition' residues and are listed in N- to C-terminal direction; all other residues in the ZF are called the 'backbone' [34-36]. As a consequence, the recognition sequences of the ZFNs bind target DNA sites through amino acids -1 to 6 of the 'recognition' alpha helix in the *3' *to *5' *direction, a reverse-pattern that can be confusing as the DNA target site is always referred to in the *5' *to *3' *direction, whereas amino acid sequences are referred to from the *N *to *C *terminus. In this study, there were ZFAs cleaving at every 5-9 bp within the linear-context of the HSV-2 genome. Graphic-analysis (Figure 1) of the HSV-2 genome cleavage-pattern by the identified 684 ZFNs revealed that the highest incidence of HSV-2 genomic cleavage was situated within the last 30,950 base-pairs (154,784/5: ~between the genomic context coordinates 0.80 and 1.00) at the 3' end of the HSV-2 genome. That said, at approximately 3094.9 bp (30,950/10) before and after the 5' and 3' ends of the HSV-2 genome (genomic context coordinates 0.02 and 0.98, respectively) another array of specificity sites for ZFNs were identified that are potentially best-suited for the complete excision of over 60% of HSV-2 genomic material from within infected latently human cells (see additional files 4 and 5**, **respectively, and Table 1) through the process of non-homologous end joining (NHEJ), following the introduction of a double-strand break (DSB)[39]. According to Dolan *et al. *[27], this targeted region comprises over 58 genes on the U_L _region of the HSV-2 genome, inclusive of the (a) *native-structural ones*: a virion glycoprotein gene (UL1), capsid protein genes (UL18, 35), tegument protein-genes (UL46-49); and (b) *others expressed-functionally*: DNA polymerase genes (UL30, 42), a DNA helicase-primase gene (UL52) and other indispensable genes.

**Table 1:** List of ZFNs cleaving within 3,094 bp located 5' and 3' of the HSV-2 genome context

Zinc Finger Nuclease (ZFN)	Left Fn; triplet- α-Helix	Right Fn; triplet- α-Helix
-**target HSV-2-DNA site 5'**		
**ZFN-unknown-SP-7-1**		
1 aGTCCCCGTCCTGCCGC**GCGGGGGCG**g 27	F1; EEANLRR; (**GAC**)	F1; KRHTLTR;(**GCG**)
1 t**CAGGGGCAG**GACGGCGCGCCCCCGCc 27	F2; RREHLVR; (**GGG**)	F2; RREHLVR;(**GGG**)
	F3; DPSNLQR; (**GAC**)	F3; RTDSLPR; (**GCG**)
**ZFN-unknown-SP-5-1**		
69 gCCCCGCGGCGCGCG**GGGGGAGGG**g 93	F1; RGNHLRR; (**GGG**)	F1; KKDHLHR;(**GGG**)
69 c**GGGGCGCCG**CGCGCCCCCCTCCCc 93	F2; RTDTLAR; (**GCG**)	F2; QSAHLKR; (**GGA**)
	F3; ERRGLAR; (**GCC)**	F3; RTEHLAR; (**GGG**)
**-target HSV-2-DNA site 3'**		
**ZFN-unknown-SP-6-685**		
154647 gCCCTGCCGCCCGCCC**GCCGCCGCC**g154672	F1; RRAHLQN; (**GGG**)	F1; DGSTLRR;(**GCC**)
154647 c**GGGACGGCG**GGCGGGCGGCGGCGGc154672	F2; QSTTLKR; (**GCA**)	F2; DSSVLRR;(**GCC**)
	F3; RLDMLAR; (**GCG**)	F3; ERRGLAR;(**GCC**)

**ZFN-unknown-SP-6-682**		
154561 gCCCCGCGGCGCGCGG**GGGGAGGGG**c154586	F1; RGNHLRR; (**GGG**)	F1; RNTHLAR;(**GGG**)
154561 c**GGGGCGCCG**CGCGCCCCCCTCCCCg154586	F2; RTDTLAR; (**GCG**)	F2; RQDNLG; (**GAG**)
	F3; IRHHLKR; (**GGT**)	F3;QQGNLQL; (**TAA**)

### Modeling of a recombinant HSV-2 viral vector for delivering and transducing the HSV-2 genome specific ZFN, genotype

Recombinant, conditionally-replicating Herpes simplex virus Type 1 (HSV-1) and II (HSV-2) vectors for purposes of malignant glioma treatment [40] and HSV-2 vaccination [41-46], respectively, have been described previously. The majority of recombinant-HSV-2-based vectors have yielded encouraging results in Phase I and Phase II clinical trials [21-24,47,48]; while genetically modified-HSV-I-based vectors raise safety-concerns around their potential to induce apoptosis in CNS neurons, causing severe and often fatal encephalitis or epileptiform seizures in immune competent humans. HSV-2 viral vectors are thought to be safe at this time.

Here, this modeling will focus on recombinant, yet replication competent, HSV-2 viral vectors for delivering and transducing a diploid copy (or pair) of the HSV-2 genome- specific- ZFN-genotype within neuronal tissue, to be used as a novel HSV-2 genomic-vaccine/therapy.

• Firstly, it was observed in accordance with Dudek and Knipe [42], that it is possible to use two types of replication-impaired HSV mutant viral strains as vectors for HSV vaccines, these being (a) replication-defective mutant strains and (b) single-cycle mutant strains. Replication-defective mutants can infect cells and express immediate-early and early viral gene products and several late gene products but contain defects in viral DNA replication, so that their replication cycle is irreversibly blocked.

• Secondly, it was noted that two sub-types of HSV-2 replication-defective vectors are equally possible. On one hand, Dudek and Knipe [42] have described HSV-2 mutant vectors defective for ICP8, demonstrating that these vectors express viral late genes in absence of viral DNA replication, probably as ICP8 and/or a complex of viral DNA replication proteins exerts an inhibitory effect on viral late gene expression in the absence of viral DNA synthesis. On the other hand, Laing *et al. *[25] have presented two variants of HSV-2 vectors with deletions in the ICP10 genes. Among the latter group of HSV-2 mutant vectors, the HSV-2 mutant ΔRR is deleted in the ICP10 RR domain for the enzyme ribonucleotide reductase, which is required for virus replication in neurons; the HSV-2 mutant ΔPK is deleted in only the protein kinase (PK) domain of the large subunit of ribonucleotide reductase (R1, also known as ICP10)[25,49-51].

To construct the proposed model-recombinant HSV-2 viral vector for delivering and transducing the diploid copy of the HSV-2 genome specific ZFN-genotype it is suggested that the *Stu*I/*Bgl*II fragment encompassing the ICP10PK domain within the HSV-2 mutant ΔRR previously described by Laing *et al. *[25], should be replaced by an alternate pair of restriction enzymes' franked fragments (specifically, the ZFN-consortium's preference of XbaI/NotI [34-36]) that encompass or accommodate a diploid copy (or pair) of the HSV-2-specific-ZFN-genotype of interest (denoted as 2**x**Zif_HSV-2_-FN). The final construct, denoted as HSV-2ΔRR [ICP10PK- 2**x**Zif_HSV-2_-FN] would contain ICP10PK- 2**x**Zif_HSV-2_-FN driven by the authentic ICP10 promoter, which is regulated by IE kinetics (independent of virus replication) and responds to AP-1 [25,52,53]. The diploid copy (or pair) of ZFN is necessary, as ZFN operates as a dimer. *In-vivo*, this dimerization is mediated by the FokI cleavage domain through cleavage of a five or six base pair 'spacer' sequence that separates the two inverted target 'half sites' [34-36]. Pre-clinical testing for the efficacy and safety of this model vector is proposed within Vero cells following transfection with 1 μg of infectious HSV-2 DNA at 20-fold molar excess using Lipofectamine (Invitrogen), with (test sample) or without (control) prior transduction using our model-recombinant vector (HSV-2ΔRR[PK- 2**x**Zif_HSV-2_-FN]). Efficacy for Zif_HSV-2_-FN expression should be monitored using an Zif_HSV-2_-FN specific ELISA, while the extent of infectious HSV-2 DNA abrogation can be measured using RR-assays that will not detect viral vectors which are deleted for the RR-gene. Safety can be monitored by measuring the extent of Vero-cell death using staining with ethidium homodimer (Molecular Probes, Eugene, OR, USA), a fluorescent nuclear stain in the red spectrum that penetrates dead cells and increases intensity after binding to DNA, as described by Laing *et al. *[25]. Stained cells are counted in five randomly selected microscopic fields (at least 250 cells) and the percentage of apoptotic cells is calculated relative to the total number of cells visualized by permeating the cultures with 5% Triton X-100 for 30 s followed by incubation with the fluorescent nuclear stain, DAPI (Sigma). Data are expressed as the mean percentage positive cells ± SEM.

## Discussion

This paper reports the first identification of ZFNs with specificity to HSV-2 genomic DNA that are potential precursors for novel host-genome expressed HSV-2 gene-therapeutics or vaccines. Based on the fact that infection with HSV-2 is associated with an increased risk of HIV-1 acquisition and transmission [9,10], that existing treatment options for HSV-2 only serve to resolve the symptomatology (ulcers) associated with active-HSV-2 infection and do not eliminate latent-virus [13-15], and that there are no clinically approved vaccines with substantiated efficacy for preventing HSV-2 infection [19-25], myself and various colleagues [31], using a primitive bacteria anti-phage DNA defense, the restriction modification (R-M) system, previously identified the *Escherichia coli *restriction enzyme (REase)-EcoRII as a novel peptide to excise or irreversibly inactivate latent HSV-2 DNA from within infected cells. The high prevalence of sequences of the site specificity palindrome of EcoRII 5'-CCWGG-3' (W = A or T) within the human genome was noted to be a potential source of host-genome toxicity. As a consequence, all previously modeled EcoRII-based HSV-2 therapeutics were allocated for *ex-vivo *expression as topical applications or microbicides [32,33]. The consensus was that HSV-2 specific nucleases were required for *in-vivo *use, as these would not cause potential human-genome toxicity. Grosse *et al. *[37] previously identified homing (mega-) endonucleases specific to HSV-1 and demonstrated their ability to inhibit HSV-1 infection in cultured cells. The hybrid ZFAs (additional file 2) and ZFNs (additional file 3) identified here have unique specificity for HSV-2 genomic sequences (additional file 1and Figure 1). Therefore, they too may offer a safer alternative to EcoRII, particularly as they can be expressed *in-vivo *with no or minimal risk of toxicity to the host-genome.

Zinc fingers (Zif or ZF), like those specific to the nucleotide sequences of the complete HSV-2 genome (provided **in **additional file 1) presented **in **additional file 2, are protein motifs capable of targeted DNA-binding [34-36]. Each individual zinc finger usually recognizes three nucleotide bases, but many zinc fingers can be combined to generate an array capable, in the case of our listed ZFAs of three fingers, of recognizing nine nucleotides [54-57]. Owing to this unique ability to target and bind to a specified nucleotide sequence, zinc fingers have previously been used to direct small molecules into unique sequences within the human genome including genes of integrated viruses [58,59]. For example, inhibition of HIV replication *in-vivo *using small artificial molecules modified to harness target DNA, a binding mechanism inherent in zinc finger (ZF) domains as a strategy to repress HIV transcription, has previously been reported by Segal *et al. *[58] and Eberhardy *et al. *[59]. Despite these reports, and that by Grosse *et al. *[37] concerning homing (mega-) endonucleases specific to HSV-1, ZF-nuclease-based disruption or abolition of HSV-2 genomes has yet to be reported. The latter ZFNs, like the 645 HSV-2 genome specific ones presented in Table 1 and additional files 2, 4 and 5, are artificial hybrid (chimera) restriction endonucleases constructed by covalently linking the DNA-binding domains of an array of 3-6 zinc fingers on to the non-specific DNA cleavage domain (or simply F_N_) of the Flavobacterium *okeanokoites *bacteria restriction endonuclease, FokI [34-36]. *In-vivo*, as noted above, the zinc finger nucleases function as dimers (or pairs) [54-57]. Therefore, considering our three ZF array based ZFNs, eighteen (9 × 2) or more (plus the 5-7 spacer- sequence) nucleotide base pairs will be recognized and cleaved. As a result, unlike the five base-pair specificity palindrome of EcoRII {5'-CCWGG-3' (W = A or T)} [31], ZFNs possess longer (> 18 bp) sequence recognition abilities, a unique feature that endows ZFNs with more target-specificity to HSV-2 genomic DNA, without risking off-target damage to host genomic DNA. Although the ZFNs identified in Table 1 are specifically proposed for excision of over 60% of the HSV-2 genomic DNA from infected host cells through NHEJ, it is possible to alternatively target and disrupt HSV-2 virulence genes using those ZFNs that cleave several times within the contextual position corresponding to the target-gene of interest (see additional file 3 for all ZFNs cleaving within the HSV-2 genome), although there are questions surrounding these propositions.

One may argue that excision of 60% or more of HSV-2 DNA in the host genome can not structurally abolish latency (RL, US and RS regions remain largely unaffected), despite functionally disabling the virus due to deletion of several vital structural and functional genes located within the deleted target UL-region. As a consequence, the ultimate fate of the residual 40% HSV-2 DNA (RL, US and RS regions) warrants explanation. For instance, since the HSV-2 genome content occasionally exists in doublex [27], it can be argued that the residual 40% HSV-2 DNA, alone or in association with DSB by-products [34-38,53-57], presents a potential source of homologous recombination (HR) and/or repair of the HSV-2 genome-cleavage products. This underlines the requirement for *in-vivo *data to evaluate the long-term effects on the host and the HSV-2 genome, and of using ZFNs as *in-vivo *therapeutics using methods similar to those proposed above. The coverage and efficiency of gene delivery and transduction by the advanced model-*delta-RR *vectors remains questionable and requires focused experimental evaluation. Specifically, it remains unclear which cells (latent genome is retained in a fraction of the sensory neurons) will be therapeutically targeted and modified, and to what extent. For example, if sensory neuronal cells are heterogeneously targeted and not homogenously transduced to the necessary-efficacious extents, one may argue that the less-effected neuronal cells could be a source of new virus that could result in recombination and re-established latency. The exact modus of clinical administration and use of the proposed model-*delta*-RR vectors to deliver the diploid copy (or pair) of the HSV-2 specific ZFN genotype remains unclear, although sub-dermal injection may be possible. This carries the potential risk of off-target gene-delivery, to epithelial cells for example, and further emphasizes the need for preclinical studies and trails. It may be argued that resistance to ZFNs could arise through mutations. However, such mutations can not simply be single-point mutations, as is the case with those that cause resistance to REase activity. As early as 1984, Brown *et al. *[60] demonstrated that several HSV-2 mutants with cumulative restriction site deletions in one of the repeats (TRL or IRL) have a measurable growth disadvantage relative to wild-type virus in tissue culture. Therefore, it is can be argued that any attempt to accumulate mutations in the over 23 bp (18 palindrome bp, 9 for each ZFN dimer; plus a 5 bp-spacer) sequence targeted by the ZFNs could exert deleterious effects on the survival and replication of the mutant isolate of the HSV-2. Moreover, existing evidence from Grosse *et al. *[37] and Cradick *et al. *[61], who have previously identified HSV-1 specific homing (mega-) endonucleases and demonstrated their inhibition of HSV-1 infection in cultured cells, shows that artificial mega-nucleases (zinc finger nucleases) mediated mutagenesis offers a novel therapeutic strategy for targeting HSV-1 and episomal Hepatitis B Virus DNAs, respectively, and clearly supports these propositions. The design of specific ZFNs for *in-vivo *use may require further improvement, say-with more than the 3 zinc finger arrays (4-6, to be exact) [54]. In addition, the efficiency of ZFN cleavage could be improved by further optimization [54] and modifications to the cleavage domain, as Doyon *et al. *[62] previously demonstrated in order to generate a hybrid capable of functionally interrogating the ZFN dimer interface to prevent homodimerization, while enhancing the efficiency of cleavage. A number of other concerns may be cited concerning the use of the proposed vectors (the advanced model **o**f recombinant HSV-2 vectors carrying and delivering the desirable therapeutic diploid copy (pair) of the genotype of HSV-2-genome-specific-ZFNs (HSV-2ΔRR [PK- 2**x**Zif_HSV-2_-FN])) in a clinical setting. For example, it can be argued that using vectors constructed from a basic-core of HSV-2 genes may render the genomes of these viral vectors susceptible to cleavage by the HSV-2-genome specific-ZFNs they carry, if the vectors are replication competent. Here, we suggest that use of viral vector constructs that are defective of HSV-2 genomic DNA (amplicons) could replace the HSV-2 core-gene vectors, and eliminate this possibility. Alternatively, although several clinical trials concerning HSV based viral vectors have been conducted and demonstrated their safety [40-46], no clinical trial-data exists concerning the safety of our proposed and modeled HSV-2 viral vectors that deliver and transduce the diploid copy or pair of the HSV-2 genome-specific ZFN genotype. Therefore, data concerning safety and efficacy of the advanced model-vectors is required before this model is clinically used in humans.

In conclusion, this research has identified ZFNs with specificity to HSV-2 genomic DNA. These HSV-2 genome-specific-ZFNs present ideal precursors for use as novel host-genome expressed HSV-2 gene-therapeutics or vaccines. Specifically, using the advanced model of recombinant HSV-2 vectors carrying and delivering a diploid copy (or pair) of the desirable therapeutic genotype of the HSV-2-genome- specific-ZFNs (HSV-2ΔRR [PK- 2**x**Zif_HSV-2_-FN]), it may be possible to irreversibly inactivate infectious HSV-2, through excision of over 60% of its genomic DNA or targeted disruption of specific virulence genes.

## Methods

### Identification of HSV-2 genome-specific ZFAs and ZFNs

#### Design

*In-silico *informatics

#### Materials and software

FASTA format of the nucleotide sequences of the whole genome of HSV-2 strain HG52 (Dolan *et al. *[27]) (provided in additional file 1**; **the NCBI accession number provided at end) and the Zinc-Finger Nuclease-Consortium's software CoDA-ZiFiT [34-36] (see software and availability section for URL link).

#### Interventions

The FASTA format of the nucleotide sequences of the HSV-2 genome were fed into the user interfaces of CoDA-ZiFiT-ZFA and the CoDA-ZiFiT-ZFN, both of which were pre-set at default, with a spacer-option of 5-9 bp selected for the latter.

#### Measured variables

Lists of ZFAs and ZFNs, inclusive of graphic maps of their action in the genomic context of HSV-2, were generated as per the user protocol [34-36].

### Modeling the prototype recombinant HSV-2 viral vector delivering and transducing HSV-2 genome-specific-ZFN genotype

A review of available relevant literature [25,41-50] concerning HSV-2 viral vectors was carried out, as is sequentially described in the respective results' sub-section above.

### Software and database availability

- The ZFN consortium CoDA-ZiFiT-ZFA/ZFN software and algorithms used are available at the following url: http://www.zincfingers.org/scientific-background.htm

- The NCBI viral genome database hosting the complete HSV-2 genome used, is available at the following url: http://www.ncbi.nlm.nih.gov/genomes/GenomesHome.cgi?taxid=10239

### Accession numbers

The NCBI gene identity of the complete HSV-2 strain HG52 used in this study is: |NC_001798|, and gene-bank identity (gi) is |6572414|emb|Z86099.2|. Sequencing Center: A. Dolan, MRC Virology Unit, Church Street,, Glasgow,, G11 5JR, UK

## Competing interests

WM is Chief scientific officer at Restrizymes Biotherapeutics (U) Ltd, and a member of the steering committee of the Young and Early Careers' Investigators (YECI) of the Global HIV Vaccine Enterprise.

## Authors' contributions

WM concieved the idea for this article, designed and undertook the experiments, and wrote the MS.

## Supplementary Material

Additional file 1 **A detailed list of the 154,746 nucleotide bases within the HSV-2 genome used**. This file offers FASTA-format listing of the 154,746 nucleotide bases within the HSV-2 strain HG52 genome used, as deposited in the NCBI viral genome database by Dolan *et al. *[27]. Click here for file

Additional file 2 **A detailed list of some of the over 28,000 Multi-Zif assembly as specific to the HSV-2 genome**. This file lists the Multi-Zif assembly specific to the first 1, 478 nucleotide sequences within the genomic context of HSV-2 from bp 1 to 8,174. Click here for file

Additional file 3 **A list of the 684 ZFN that are specific to the HSV-2 genome**. This file details the entire list of the 684 ZFN that are specific to the HSV-2 genome along with their specificity positions or sites of clevage. Click here for file

Additional file 4 **A list of the -1 to 6 recognition domians of the alpha-helix for some of the ZFN cleaving within the 3,094.9 bp at the 5' end of the HSV-2 genome**. This file lists details of the -1 to 6 recognition domians (denoted F1, F2, F3/F3, F2, F1) of the alpha-helix for some of the ZFN cleaving within the 3,094.9 bp at the 5' end of the HSV-2 genome. Click here for file

Additional file 5 **A list of the -1 to 6 recognition domians of the alpha-helix for some of the ZFN cleaving within the 3,094.9 bp at the 3' end of the HSV-2 genome**. This file lists details of the -1 to 6 recognition domians (denoted F1, F2, F3/F3, F2, F1) of the alpha-helix for some of the ZFN cleaving within the 3,094.9 bp at the 3' end of the HSV-2 genome. Click here for file
