# Supplementary material for: Identity of zinc finger nucleases with specificity to herpes simplex virus type II genomic DNA: novel HSV-2 vaccine/therapy precursors
Source: Theor Biol Med Model. 2011 Jun 24;8:23. doi: 10.1186/1742-4682-8-23 (PMC3138452; doi:10.1186/1742-4682-8-23)
Supplement: Additional file 1 — A detailed list of the 154,746 nucleotide bases within the HSV-2 genome used. This file offers FASTA-format listing of the 154,746 nucleotide bases within the HSV-2 strain HG52 genome used, as deposited in the NCBI viral genome database by Dolan et al. [27]. [file 1742-4682-8-23-S1.DOC]

>gi|6572414|emb|Z86099.2| Herpes simplex virus type 2 (strain HG52), complete genome (~154,748 base-pairs)

AGTCCCCGTCCTGCCGCGCGGGGGCGGGCGCGGGAAAAAAGCCGCGCGGGGGCGCCCGCGGGAAGGCAGC

CCCGCGGCGCGCGGGGGGAGGGGCGGCGCCCGCGGGGGAGCGGCCGGCTCCGGGGGAGGGACGGGGAAGG

GGGCGCGCGGGGCTGCCCTGCCGCCCGCCCGCCGCCGCCGCCCGCCTTCGCGCCCCCCCCCAAAAAACAC

CCCCCCCGGGGGTTGACTCCCCGGGGGAAAAGAGGCGGGGCGGGAGTCCCCGTCCTGCCGCCGCCCCTTA

AGAGGGCCCGCAACACGGCCCGGGCTGCGCACGCCAGCCGGGACGGGTGAGTTCGCTAGGCAAGCACGGA

CTGGCGGTTACACGTGCATGCGTGCCGAGTGAACTCTCCCGCCCCGACGCGCTCCGGCTCCGGGCCTACG

CCGAGCCCAGCCGCCCGCCATGTCCCGCCGCCGGGGTCCCCGCCGCCGGGGTCCCCGGCGCCGGCCGCGC

CCCGGCGCTCCAGCCGTGCCGCGCCCCGGCGCTCCAGCCGTGCCGCGCCCCGGCGCGCTCCCAACCGCAG

ACTCCCAAATGGTCCCTGCGTACGACTCGGGAACCGCGGTCGAGAGCGCGCCGGCCGCGTCCTCGCTCCT

GCGGCGCTGGCTGCTGGTGCCCCAGGCGGACGACAGCGACGACGCGGACTACGCCGGCAACGACGACGCA

GAGTGGGCGAACAGCCCCCCGAGCGAGGGCGGGGGGAAGGCGCCGGAGGCCCCGCACGCCGCGCCTGCCG

CCGCCTGCCCCCCGCCGCCGCCGCGCAAGGAGCGCGGGCCGCAGCGCCCCCTTCCGCCCCACCTGGCGCT

ACGGCTGCGCACCACGACGGAGTACCTGGCGCGCCTGAGCCTGCGCCGGCGGCGGCCCCCCGCGTCCCCG

CCCGCGGACGCGCCGCGCGGGAAGGTACGCCTCCCCTCCGACCCCCTGACGCCCCTCCGACCCCCTGACG

CCCCTCCGACCCCCTGACGCCCCTCCGACCCCCTGACGCCCCTCCGACCCCCTGACGCCCCTCCGACCCC

CTGACGCCCCTCCGACCCCCGTGTCTCCCCGCCCGCAGGTGTGCTTCTCGCCGCGCGTGCAGGTGCGCCA

TCTGGTGGCCTGGGAGACGGCCGCGCGCCTGGCCCGACGGGGGTCCTGGGCGCGCGAGCGGGCCGACCGC

GACCGGTTCCGGCGCCGCGTGGCGGCGGCCGAGGCGGTCATCGGACCGTGCCTGGAGCCCGAGGCCCGAG

CTCGGGCCCGAGCCCGAGCCCGGGCCCACGAAGACGGCGGACCCGCGGAGGAGGAGGAGGCGGCGGCGGC

GGCGCGCGGGTCCTCCGCCGCCGCGGGCCCGGGCCGTCGGGCGGTCTAGGGTTGAACCGGCGAGGGCGGC

CTCGGCCGGCGGAGCCCCGGAGCTCCGAAGGTCTGCGCGAGGCCGCTCTCCGAAGAGACGATGGGAGCCC

CGCGTATATATCCGCGAGGGCCCGGCGCCGCCCCGCCGCTCCGCCCGCCCCAGGGGGCGGCGCCGGCCAA

CCGCGCGCCGCCGCGCGGGCCCGGACTCCGCCCCGGCGACCGCCCCGCGCCGGCTTCCCGGTATGGTAAT

TAGAAACTTTTAATAGGCGGTCCCGGCCGCCATCCCCGCGCATGGTAATTAGCAACTTTTAATGGGCCGG

CGTTCCCGCTCGCGGTAATTAGCAGCTTTTAACGGGCCGCCATTCCCGCTTATGGTAATTAAAAACGTTC

GGACGGCCCCTCGCTCCCCGCGTAATTACTCCCTCGGGGTTCCGGGTTATGCTGATTACTTTCTTGGCAG

AACACGCAGAGCCTCGCGCGCCGCCGGGTGGGTGGGCTGATCGGCCCCTATTGGTCCCCTGGGCTTCCTA

GTATGCTAATGAATTTTTCCCCGGGGGCGGGCACCACTCAGGGCCGCGCCGGCGGGGCGCCGGGGGGACT

CCCATCTGCGTCGGCGGGGGGCGGCGCATGCTAATGGGGTTCTTGGAGTACACCCGGTTGGTCCCCGGGG

ACGGGGCCGCCCCGAGAGGGGGGGATTCCCTCCCTCCGCCCCCGCCGGGGCGCGCGGCTATTGGGGGAAT

CGTAAATGCCGCCCCTTTGGGGGAGTGGATAGGCGCCGGGTATAAGGCAGCCCCGTGTGACGGTCGGGCC

GCATTCGCACCCCGGCACTGCGAGCGACGGAGCGGCGGCCCGGCGGGAGGAGGAGACCCGGAGAGACAGA

GACTAAAACCCGGCAAGAGAGAGACCGCGGGCCGCCGTCTCGAGTCTACCCTACCCCGGCTCATGGAACC

CCGGCCCGGCACGAGCTCCCGGGCGGACCCCGGCCCCGAGCGGCCGCCGCGGCAGACCCCCGGCACGGTG

AGAGGGCGACCCCCGGGTCTCAGGCCCCCCCTTTTCCCCGGACCACCCGGCTGCGGGTTGGGGGTGGTCG

CGGGCGGTGGGCTCGGGGGCGGGGACGCTTGACGGGGCCGACCCCCGGCCCGCTTAAGCGGTCGGGGGAC

CCCCGTGGGCCGTGCGCCGCCCCCCGACCCTCTGGGGGGGCGAGGGAGGCAGGGAGGAGCCCGAGAGCGG

GGGACAGGGGGGGAGACGAGGGGTCGGAATCCAAAGGACGCAGACCACCTTTGGTTACGGACCCCTTTCT

CCCCCCCTTCCGAACAAAAAGCAGCGGGCGGGGGGCCGGGGTGAGGGAGGGACACGGGGGACACGGCGCG

GGGGTCCCGCCTCACGCCCCGCGCCCTCTAAATCCCCCCCGTTGCTTTGTCAAGCAGCCCGCCGCCCCGC

ACGCCTGGGGGATGCTCAACGACATGCAGTGGCTCGCCAGCAGCGACTCGGAGGAGGAGACCGAGGTGGG

AATCTCTGACGACGACCTTCACCGCGACTCCACCTCCGAGGCGGGCAGCACGGACACGGAGATGTTCGAG

GCGGGCCTGATGGACGCGGCCACGCCCCCGGCCCGGCCCCCGGCCGAGCGCCAGGGCAGCCCCACGCCCG

CCGACGCGCAGGGATCCTGTGGGGGTGGGCCCGTGGGTGAGGAGGAAGCGGAAGCGGGAGGGGGGGGCGA

CGTGTGTGCCGTGTGCACGGACGAGATCGCCCCGCCCCTGCGCTGCCAGAGTTTTCCCTGCCTGCACCCC

TTCTGCATCCCGTGCATGAAGACCTGGATTCCGTTGCGCAACACGTGTCCCCTGTGCAACACCCCGGTGG

CGTACCTGATAGTGGGCGTGACCGCCAGCGGGTCGTTCAGCACCATCCCGATAGTGAACGACCCCCGGAC

CCGCGTGGAGGCCGAGGCGGCCGTGCGGGCCGGCACGGCCGTGGACTTTATCTGGACGGGCAACCCGCGG

ACGGCCCCGCGCTCCCTGTCGCTGGGGGGACACACGGTCCGCGCCCTGTCGCCCACCCCCCCGTGGCCCG

GCACGGACGACGAGGACGATGACCTGGCCGACGGTGAGGGCGGGCGGGGGTCGGGCGGGGGGCGGGCGGG

GGTCGGGCGGGGGTCGGGCGGGGGTCGGGCGGGGGTCGGGCGGGGGTCGGGCGGGGGTCGGGCGGGGGTC

GGGCGGGGGTCGGGCGGGGGTCGGGCGGGGGTCGGGCACTAACCGGGGGCTCCCGTCTCTGTCTCCCTCT

GCAGTGGACTACGTCCCGCCCGCCCCCCGAAGAGCGCCCCGGCGCGGGGGCGGCGGTGCGGGGGCGACCC

GCGGAACCTCCCAGCCCGCCGCGACCCGACCGGCGCCCCCTGGCGCCCCGCGGAGCAGCAGCAGCGGCGG

CGCCCCGTTGCGGGCGGGGGTGGGATCTGGGTCTGGGGGCGGCCCTGCCGTCGCGGCCGTCGTGCCGAGA

GTGGCCTCTCTTCCCCCTGCGGCCGGCGGGGGGCGCGCGCAGGCGCGGCGGGTGGGCGAAGACGCCGCGG

CGGCGGAGGGCAGGACGCCCCCCGCGAGACAGCCCCGCGCGGCCCAGGAGCCCCCCATAGTCATCAGCGA

CTCTCCCCCGCCGTCTCCGCGCCGCCCCGCGGGCCCCGGGCCGCTCTCCTTTGTCTCCTCCTCCTCCGCA

CAGGTGTCCTCGGGCCCCGGGGGGGGAGGTCTGCCACAGTCGTCGGGGCGCGCCGCGCGCCCCCGCGCGG

CCGTCGCCCCGCGCGTCCGGAGTCCGCCCCGCGCCGCCGCCGCCCCCGTGGTGTCTGCGAGCGCGGACGC

GGCCGGGCCCGCGCCGCCCGCCGTGCCGGTGGACGCGCACCGCGCGCCCCGGTCGCGCATGACCCAGGCT

CAGACCGACACCCAAGCACAGAGTCTGGGCCGGGCAGGCGCGACCGACGCGCGCGGGTCGGGAGGGCCGG

GCGCGGAGGGAGGACCCGGGGTCCCCCGCGGCACCAACACCCCCGGTGCCGCCCCCCACGCCGCGGAGGG

GGCGGCGGCCCGCCCCCGGAAGAGGCGCGGGTCGGACTCGGGCCCCGCGGCCTCGTCCTCCGCCTCTTCC

TCCGCCGCCCCGCGCTCGCCCCTCGCCCCCCAGGGGGTGGGGGCCAAGAGGGCGGCGCCGCGCCGGGCCC

CGGACTCGGACTCGGGCGACCGCGGCCACGGGCCGCTCGCCCCGGCGTCCGCGGGCGCCGCGCCCCCGTC

GGCGTCTCCGTCGTCCCAGGCCGCGGTCGCCGCCGCCTCCTCCTCCTCCGCCTCCTCCTCCTCCGCCTCC

TCCTCCTCCGCCTCCTCCTCCTCCGCCTCCTCCTCCTCCGCCTCCTCCTCCTCCGCCTCCTCCTCCTCCG

CCTCTTCCTCTGCGGGCGGGGCTGGTGGGAGCGTCGCGTCCGCGTCCGGCGCTGGGGAGAGACGAGAAAC

CTCCCTCGGCCCCCGCGCTGCTGCGCCGCGGGGGCCGAGGAAGTGTGCCAGGAAGACGCGCCACGCGGAG

GGCGGCCCCGAGCCCGGGGCCCGCGACCCGGCGCCCGGCCTCACGCGCTACCTGCCCATCGCGGGGGTCT

CGAGCGTCGTGGCCCTGGCGCCTTACGTGAACAAGACGGTCACGGGGGACTGCCTGCCCGTCCTGGACAT

GGAGACGGGCCACATAGGGGCCTACGTGGTCCTCGTGGACCAGACGGGGAACGTGGCGGACCTGCTGCGG

GCCGCGGCCCCCGCGTGGAGCCGCCGCACCCTGCTCCCCGAGCACGCGCGCAACTGCGTGAGGCCCCCCG

ACTACCCGACGCCCCCCGCGTCGGAGTGGAACAGCCTCTGGATGACCCCGGTGGGCAACATGCTCTTTGA

CCAGGGCACCCTGGTGGGCGCGCTGGACTTCCACGGCCTCCGGTCGCGCCACCCGTGGTCTCGGGAGCAG

GGCGCGCCCGCGCCGGCCGGCGACGCCCCCGCGGGCCACGGGGAGTAGGGGGAGCTAACACTCGGCTTGC

TGCCCGAAGGAAGCCGCCCCCCACCGGACCACCGGCCGAGGCGCCTCGGGGGCAGGGGGAGGTGGGGGGG

GGGAAAGACGGGGAGGAGACAGGAAGTGGGGGTGGGAGTGGGGGGGGGGGACGGACACGGCCCCGAACAG

CAACACACACCAGCATTTTGTTATGGACTTTCTGGCCTTGTTGAAAACTTGAGGAAAAAAAAAACTTTAT

ATTTATAAAAATTTTACAATAAAGTTTTGTGATGCTTTTGACACACTTTGTTGTTGGCCTTTGATGCAGC

TCCCCCGCGCAGGGGGGCCGGGGATGGGGGGGAAGGGAGGAGGAGGAGGGGGGGCGGGCACGAGAAGCCG

CCCCCACCCCCGAGGCCTGTTGGTCTTTATCATAGAACAGAGCCGGGGCCCGGCCTCGTTCTGGCTCCCT

GTCTTGGTGGGTGGGCGGGCTGGCTGGCGGGTAAAAAAAGAGTGTGTCCGTGTTGACAGGGAGGGGGGCC

CGATCGTGCAGAGCACGCACGTCTGGCCGGCCAGACCCTGGGGGTGGTGGGCAGGAGTGGGAGGGCGCCT

GGCTCGGGGAGGGAGGAGGGGGGGGGTCAGCCGCACCACCGGCGCGAAGCCAGGGGCCAGGGAACTTTGA

TAGAGAGGGGGGAAAGTGGGGCGGGGGCGAGGGCGGTTGAATCACAACGCATGCACGCCCTCTGCCCCCG

GGGACGGGTGGGAGGAAGGAGGAGGGAGAAGAGAAGACCCGAGGCATGCACCCGCACTTACGCCCGTGCC

CACCCCCGCCCCGGCGCCCACCCCGCCCGCACACCTGCCCGCCACGCCCGCCCCTCCTCACCCTGGCTGG

GAGAAAGGAGGAGGAGCAGGAAGAGGAGACCCGAGGCATGCAACCGCACTCACCCCACCCCGCCCGCACA

CCTGCCCGCCACGCCCGCCCCTCCTTACCCTGGCTGCGGGGAGACTCCCATCGGGGCGAGGGGGCTCGCG

CGTTCGCAACACCACACCACACCACACGGCCCACCACAACACGGCCCACCACGACACAACACGACACGAC

GCGTTTTGCGGGGCATGCAAGTCGACACACCGCGCGCGTGCCTACCTTTCCCTAGCGGCCCCGGCCCCCG

GCCCGTTTCCTTCCGCCACCACTACCACCACCCCCCCGCCCGCGCCCACGCGGTAGAGGAAGGGGACGGG

CGCCACACCCACGGCTGTGGCCGGGCACGCGCCTTTGGGGTTGTTGGGGGGGGGTGACCGGCGCGTGGGG

GCGGTGGGCGTACGGGCCCGACCCGCGCCTGCCCCCCCGGGAACGACGACGGGGGGGGGGGAAACGGGGG

TGGGTGGAAGGGAAGAGGAAGGAGAAAGGGGGGGTGGATCCGAACACGCCGGATCCGCGAAAATAATAAC

AAAACAAACAAAAACAGAAACAAAAACAAAAACACCTAGAAAAAAAGGATACGGGTTGGCTCGCGGGCGG

TGCGGCTGACCTGCCTGCCCTTTCTGGGACCCCCGCCTCGTGTTTCTTGAAAGGGGGAGGAAGAACAGTT

CTCCCCCAACCCCTGCTCTCTTCTCTCTTCCGCCCGCCCCCCCCCCCTCTCCCCGCCGCCTCAGCAGAAG

CTCACCTGTACGACCCTAAACCTACCTGCGAGAACGCGCGGCGTTCGAGGGGCGCGCTCTCTCACACGAG

ACACACGCAGGCGCCCCCCCCCCCCGGAGCCTGGGTCCCCCGGCGGACGGCTCACGCGGCGCGGCGTCTC

GGTGGGACGCGGGCAAAGGGCGGCGGCGGCGGGGGGGGGGGGGGGAAATGTGAGGAGAGCGAGACAGAGA

GAGAGAAGGAAGAGGGAAGGGGCGCGGCGGGACGGGGGAAGACGAGGAGAAGGGAAGGGGCGAGGGTCGG

GCCCGGGAGCGGGGCGGCCCGGGAGGGAGAAGAAACGGAACGCGGAAACGCCGCCGGCGCGGCCCGGGGC

CCCGGGGCCCCCGCGCTCCGCCGGGGGCCCGGGCCGGACCGCCGGGCGGGGGACGCCTTCCGCCCGGCGC

CGGGCGGCTACCCGGGACCCCCGGCCGGGAATCGAAAAAAGCCTCCGGGGGCCCCTTTCGCGCCTTTCGC

GAACGCGCGGCGCCGGAGGGGGCGGCCGCCGAGGTGCGGGGGCCCCTCCGGCCGGGGCGCACCTCGGCGG

CCAAGCCCCGGCCCGCCCGGGGGTCCCCGAGGCAAGAGGCGGACCCTCGGAGGCGCGGAAGAAGACGGGA

GGCGGGGGAAAAAAGGGGGAAGAGAGGGGGAGGTAGGGAGGGGAGAGGAGAAGGGCGCGCCGGTGCGCGG

AGCAGCCTTCCTTCTCCGGAGTCCCTCTCGATCGGCGGCGGGCCCCTGCGTTCGTTGCTGCCGCGCCCCC

GGTTTTATAAAGACAGGGATGACGCAGCAGAAATGCCCACAGCAACACGCGGGCGGGGCTCGGGCTCTCC

GGCGGCTTAATGGATCTCCGGGCACGGCGCCCGCAACCGCAGAGCACTCAGCTGGCGCGCCCCCCCCCAA

CGTGGGAGTGTTTAATGGAAGGGCGTGGGGCCGGCCGCCGGATGCCCGCGGGGGCCTAATGCGGCGGGAG

GCGTGGGCCGCTGGCGCCGCGGCCCGTCTGCTGGCCCGCGGCCCGTCTGCTGGCCCGCGGCCACGTAAAC

AATGACACAGGGGTTCTCTCCGCCGCGGCCGGCGCGGGGCGTTGCCGGCCCGGCCCGGCCCCGGAGCCCG

CGGCGCTGCTCGGCTGCGGCCGCGGGCTCCGGGGGCTCCGCACTCTGCCCGGCTCGCCCCGTCCCCCCTC

TTGCTGCTTTTCCGCGCGCCTCTCTTTCCCGTTGCTTTCCCTCTCCCCCCCCCCCCCTCTCTCTCTCTCT

CTCTCTCTCTCCGCCATCCTCCCGCCCGGCCGCCCACTCCCCGCTCGGCCTCTCCGGCTGCGGTGCTTGG

GTCTCCTTCGTCGGGCGGCGGGGGGGGGGCGTCGGGACTCGCGGAGGGCCGGAGAATGGAAGGCGAGGGG

ATGCAGGAGGAGGATCGGGACTCCCCATCTTCTGCCCTTCCATCCTCCGTTTTTCCGCTTTCCACCGCCG

CCGCCACCACCCCCCCTTCCTTCGCCCGCCCGCCTCGCCCCGGACCCCTCCCCCCCGTGTTCCCCCCATC

GTTCACCACCACGCCCCCCACCGCGCCTTGGCTGTTTGGGGGGTGGCGGCGGTGGTCGGCGTGCTGCCGG

AGGCTGCGGGCGCGGGGTAGGTGGGTGGGCGGGTGGTGGGGGGGGGCCCGGCTGCGTCTCGCCGCGATCC

CGCCGGTGGGGCGCGGCGGCGGTCGGGGTGGGGGGAGAGTGTCGTGGGTGTGTTTTCGTGTCCCCCACCA

CCACTCCCACCCCGACCGCCGCCGCGCCCGCGTTTCTGCCGCCCGCGCGCTCCTGTGTGGACCCCGGGGT

GGGCGGCGGGGGGGGGTGCCGTGGGTGTGGCGGCGGGGCGCGGGCCGGGGCCGGGGCTCGCTGGTCCGCC

GAAGTAAAGAAAAGATCGCCACCGTGTGTTCGTCTGTGTGTTCTGCGCGGCGCCGGGGCCCCCCTGCCGG

GCGGGGCGGTGGGGCGGGGTCGGGGTCGCGGCGGGGAAGGAAGGAAAGACCCCGGAAGCGCCGGGAGGGG

GCGCCGGCGCGACGCGGGCGGCCGGGCGGGGGCGCGCGGCGGCCGGGCGGGGGCGCGCGGCGGCCGGGCG

GGGGCGCGCGGCGGCCGGGCGGGGGCGCGCGGCGGCCGGGCGGGGGCGCGCGGCGGCCGGGCGGGGGCGC

GCGGCGGCCGGGCGGGGGCGCGCTTTCCCCGCGTCGCCCCTCGGGTTCCCAAGACCTATCACGTGTGCGC

AGGGGAGGGGAGGACGCGGGGGAGGGGAGGACGCGGGGGAGGGGAGGACGCGGGGGATATATAAAGCGGT

AGAAAGCGCGGGAATGGGCATATTGGACCCGCGTGATTCGGTTGCTCGCGGTTGTCTTGTTTGGACGTTT

TTTATGCGGGAACAAGGGGGCTTACCGGTTACACTGTCCGCTCGCTATGGGGTTCGTCTGTCTGTTTGGG

CTTGTCGTTATGGGAGCCTGGGGGGCGTGGGGTGGGTCACAGGCAACCGAATATGTTCTTCGTAGTGTTA

TTGCCAAAGAGGTGGGGGACATACTAAGAGTGCCTTGCATGCGGACCCCCGCGGACGATGTTTCTTGGCG

CTACGAGGCCCCGTCCGTTATTGACTATGCCCGCATAGACGGAATATTTCTTCGCTATCACTGCCCGGGG

TTGGACACGTTTTTGTGGGATAGGCACGCCCAGAGGGCGTATCTGGTTAACCCCTTTCTCTTTGCGGCGG

GATTTTTGGAGGACTTGAGTCACTCTGTGTTTCCGGCCGACACCCAGGAAACAACGACGCGCCGGGCCCT

TTATAAAGAGATACGCGATGCGTTGGGCAGTCGAAAACAGGCCGTCAGCCACGCACCCGTCAGGGCCGGG

TGTGTAAACTTTGACTACTCACGCACTCGCCGCTGCGTCGGGCGACGCGATTTACGGCCTGCCAACACCA

CGTCAACGTGGGAACCGCCTGTGTCGTCGGACGATGAAGCGAGCTCGCAGTCGAAGCCCCTCGCCACCCA

GCCGCCCGTCCTCGCCCTTTCGAACGCCCCCCCACGGCGGGTCTCCCCGACGCGAGGTCGGCGCCGGCAT

ACTCGCCTCCGACGCAACTAGCCACGTCTGCATCGCAAGCCACCCTGGGTCGGGAGCAGGACAGCCGACC

CGTCTAGCGGCCGGGTCGGCTGTCCAGCGTCGTCGCCCTAGAGGCTGTCCGCCGGGCGTGATGTTTTCCG

CATCTACGACCCCCGAACAGCCCCTGGGGCTGTCGGGCGATGCGACGCCGCCCCTGCCGACTTCCGTGCC

CCTGGACTGGGCCGCGTTTCGGCGCGCGTTTCTGATCGACGACGCCTGGCGGCCCCTGTTGGAGCCGGAG

CTCGCGAACCCCCTAACCGCGCGCCTCCTCGCGGAGTATGACCGTCGGTGCCAGACCGAAGAGGTGCTGC

CGCCGCGGGAGGATGTGTTCTCCTGGACGCGGTATTGTACCCCCGACGACGTGCGCGTGGTTATCATCGG

GCAGGACCCGTACCACCATCCCGGCCAGGCGCACGGCCTGGCGTTTAGCGTGCGTGCGGATGTGCCGGTG

CCTCCGAGTCTACGGAACGTGCTGGCGGCGGTTAAAAATTGTTACCCCGACGCGCGCATGAGCGGCCGCG

GCTGCCTGGAAAAGTGGGCTCGCGACGGCGTGCTGTTGTTGAACACGACCCTGACCGTCAAGCGCGGGGC

GGCGGCGTCCCACTCCAAGCTTGGATGGGACCGTTTTGTGGGCGGGGTGGTCCAACGGCTGGCCGCGCGC

CGCCCGGGCCTGGTCTTTATGCTCTGGGGCGCCCATGCCCAGAACGCGATCAGGCCCGACCCTCGCCAAC

ACTACGTCCTCAAGTTTTCTCACCCGTCGCCCCTCTCCAAGGTCCCGTTTGGGACGTGCCAGCATTTCCT

CGCCGCGAATCGCTACCTCGAAACCCGGGACATTATGCCGATCGACTGGTCGGTATAAGATGCCGACATC

CGGGGTCTTGATTTACGAGGGGGCAATTAATAAAGACTGTTGATGGTTAAATCTCGGGTCTCATACCGGT

CCGTGATGTCGGGCGTGGGGGAAGAGAGGGTCCCCTCTGCGTTTACTATCCTTGCCTCGTGGGGCTGGAC

GTTTGCACCCCAGAACCATGATCCTGGCGCGTCGCCGAATACGACGCCCATAGAGTCGATTGCGGGGACC

GCACCGGACGCGCACGTGGGGCCTCTCGACGGAGAGCCGGACCGGGATGCGATCTCCCCGCTTACGTCGA

GCGTGGCCGGCGACCCGCCGGGGGCGGACGGCCCCTACGTCACCTTTGATACTCTGTTTATGGTATCTTC

GATCGACGAACTGGGGCGCCGCCAGCTCACGGATACGATCCGTAAGGACCTGCGGCTGTCGCTGGCCAAG

TTCAGCATCGCGTGTACCAAGACCTCGTCGTTTTCGGGGACGGCCGCGCGCCAGCGCAAGCGCGGAGCAC

CGCCGCAACGCACATGCGTACCACGCAGCAACAAGAGCCTCCAGATGTTCGTTTTGTGCAAGCGCGCCAA

CGCCGCGCAGGTGCGCGAGCAGCTGCGGGCGGTTATTCGGTCGCGCAAGCCGCGCAAGTATTACACGCGG

TCCTCGGATGGGCGGCTCTGCCCGGCCGTCCCCGTGTTTGTACACGAGTTTGTTTCGTCCGAACCCATGC

GCCTCCATCGAGATAACGTCATGCTGTCTACGGAACCAGACTAAGCACCCCCGCCGTCCCCTTTCTTTTC

CCCCTACCCTTCCCCCGTTACTGATGTGTTGTACGTTTCAATAAATAACACGTAGCTTATTTTGTTGGAT

GATGGATTGATTGATTTTATTGACCGTTCGTTCGCCCGGCGGTGCCGTCGCCGCGCGCAGAGGGAATATG

CAAGCGGGCGGGGTGGGGAGGAAAGAAGGTTTCAGGTTCCGGGGGTTGGGTCTGCGTCGTCCAGGGTGGG

GCTGATCTGAATTTCCCGCAGAACCTCGACCAGTAGGTCTGTTGTGTTTGCTGGGAACTCGCCCGCCGTT

GGGGATACGGGGGCGGGGGGTGTGGTCGGGCGGACGTCCAGGGGTGCGTTATCGCACCCCCGCGCCGCCT

CGGGGGCCGTCCCGTAGATCGTTGCGGTGATGTAGATGGTGTCCGGGGTCCACACCACCGTCAGGATGCC

GGCCGTCGCACTCCGGACGCTTTCGCCGTGCGATGAGCTGACCCAGGAGTCAAAGGGGTACGCGTACATA

TGGGCGTCCCACCAGCGCTCCAGCCTCTGGGTACTAGCGCGTCCTATAAAGCGGTATGCGCAAAATTCGG

CACGACAGTCGATAATCACCAGCAGCCCGATGGGGGTGTGTTGTATCACCACGCCTCCGCGGGGCAGGCG

GTCCTGGCGCGCTCGACCCCGCGTCAGAACCGCGCGCGTCCCTGACTCAAACACGTGCACCACCTGTGCC

GCGTCCGGCAGCGCGCTCGTTAGCGACGCCCTGGGGTGATGTAGGCTGTACGCGATGGTCGTCTGGGGGT

TCCCCATGTCTCGGGGGGGTGGGGGTGAATGTCACCCGGCCCGGGTGCGGTGGGAACGCGAGGGAATGGA

GGGTTAATAGACAATGACCACATTCGGATCGCGTAGAGCAGATAGTATGTGCTCGCTAATGACGTCATCG

CGTTCGTGGCGCTCCCGGAGCGGGTTTAGATTCATGTGCAGGAACTCGGATGAGGTGGTGCGGGACATGG

CTACGTACGCGCTGTTTAGGCGCAGGTTTCCGGGCGTGAAGCATATGGCGACCTTGTCCAGACTGAGCCC

CTGGGAGCGCGTGATGGTCATCGCGAGTTTGGAGCTGATGCCGTAGTCGGCGTTGATGGCCATGGCCAGC

TCCGTGGAGTCGATCGACTCGACAAACTCACTGATGTTGGTATTGACGACAGACATGAAGCCGTGCTGGT

CCCGCAGGACGATGTAGGGCAGGGGGGACTCCTCCAAGAACTCGGCCACGCCGGCCGTCGCGTGCCGCCG

CCGCAGCTCCTCCGCGAACGCGAACACCCGGGTGTACGTGTACCCCATCAGCGTGTAGTTGTCCGTCTGC

AGGGCCACGGACATCAGCCCCCCGCGCGGCGAGCCGGTCAGCAGCTCGCAGCCCCGGAAAATGACATTGT

CCACGTAGGTGCTGAAGGGGGCGCTCTCAAACACCTCCCCGAAGAGCTCCCGTAGGATAAGGTATCGCCC

CAGAAAGGCCCTCTTCAGGAGCCCAAACTGGGCGTGGACGGCCGCGGTGGTCTCAGGCTCTTCGAGGGCG

TAGTGGCAGTAGAACACGTCCAGCTGCTGTTCGTCCAGCCCGGCGAAGATAACGTCAAGGTCGTCGTCGG

GGAAGTCGTCCGGGCCCCCGTCCCGCGGGCCCAGGTGCTTAAAATTGAACGCACGCTCCCCCGGAGAGCG

GTCGCTGGTGTCGGCGGCCCTGGTTGCCGATGCGCCGGCGGCGTCCCGGCGTAGCGACAGGAGTTCTGCC

GTCAGCTCCCCTAGGCGGCCGTAGGCCAGGGTCCTCTGGGTCGCGTCCAGGCCGGGGCGCTGGAGAAAGT

TGTAAAAGTGAATCAGCCCGCCGAACATGAGCCGCGACAGGAACCGGTAGGCGAACTCCACCGAGGTCTC

CCCCTGGGTCTTCACGAAGCTGTCGTCGCGCAGCACAGCCTCGAAGGTCCGAAACGTCCCGTCGAACCCA

AACACCATCTTTCGGAGGCGCGCGGTCACCGCGACCTGGCTGTTGAGGACGTACGTGATGTCGTTCCGGG

CCACGACTAGCTGTTGCTTGCTGTGCACCTCACAGCGCACGTGCCCCGCGTCCTGGTCCTGACTCTGGGA

GTAGTTGGTGATGCGACTGGCGTTGGCCGTGATCCACTTTTCCATGGTCAGCGTGGGTTGCTGCGTGAGC

CGTCGATACTCGTCAAACTCTTTGACCGACACAAACGTGAGCACGGGGAGGGTAAACACAACAAACTCCC

CCTCGCGAGTCACCTTTAGGTAGGCGTGGAGCTTGGCCATGTACGCGCTGACCTCCTTGTGGGACGAGAA

CAGCCGCGTCCACCCCGGAAGGTTGGCCGGGTTGGTGATGTAACTTTCCGGGACGACAAAGCGGTCCACA

AACTGCATGTGCTCCTCGGTGATGGGAAGGCCGTACTCCAGCACCTTCATGAGGTTCCCGAACTCGTGCT

CCACACATCGCTTGTTGTTAATGAAAATGGCCCAGCTGTGCGAGAGGCGCGTGTACTCGCGTAGGGTGCG

GTTGCAGATGAGGTACGTGAGCACGTTTTCGCTCTGCCGGACGGAGCATCGCAGTTTTTGGTGTTCGAAG

GTGGACTCCAGCGAGGCCGTCTGGGTCGGCGACCCCACGCACACCAGCACCGGCCGCAGGCGGCCCGCGT

ACTGGGGGGTGTGGTACAGGGCGTTAATCATCCACCAGCAATACACCACGGTCGTGAGTAGGTGCCGCCC

CAGGAGCCCGGCCTCGTCGATGACGATAATGTTGCTGCGGGTGAAAGCCGGCAGCGCCCCGTGTGTGACC

GAGGCCAGGCGCGTGAGGGCACCCTGGCCCAGCCCCAAAGTCTGCTCTAGGGCGGTGAGGGCGTGGAACT

CGTTTCGCGCGTCTTCGCCCCCGTGCGCCGCCAGGGCCCGCTTGGTGATGTCGAGGATCACCTCCCAGTA

GTACGTCAGGTCTCGCCGCTGCAGGTCTTCCAGCGAGGCGGGGCTGCTGGCCAGGGTGTACGGGTGCTGC

CCCAGCTGGGCCTGGACGTGATTCCCGCGAAACCCGAACTCGTGAAAGATGGTGTTGATGGGTCGACTCA

GAAACGCCCCCGAGAGCTTAACGTACATGTTCTGCGCCGCGATTCGCGTGGCGCCCGTGACCACGCAGTC

CAGGACCTCGTTGAGGGTCTGCACGCACGTACTCTTTCCGGATCCGGCGTTGCCGGTGATGAGATACGCC

GCGAACGGAAACTCCCGGAGCGGCAGGCCGGTCGGGACCTCCAAGGCCGCCACGTCCCGGAACCACTGCA

GGCGCGGCACCTGCGTGACGTCGAGCTGCTGCTGCGAGAGCTCTCGGATGCGTGCGATGATTGGTTGGAC

CCCGTGCATGGACGTAAAATTTAAAAACGCCTCGTCCCTGAACCGCACGGCGGGTCTGGCCCCGGGCTGC

TGTGGGGGCGGACCTGGTGCCCGGACGTCCCGCGAGCCCTCCCCGCCGGACGCCGCCATGGCCGCACAGC

GCGCGCGGGCGCCGGCGATGCGGACGCGGGGCGGCGACGCGGCGCTATGCGCCCCCGAGGACGGCTGGGT

GAAGGTTCACCCCACCCCCGGGACGATGTTGTTCCGCGAGATTCTCCTCGGGCAGATGGGGTACACCGAG

GGTCAGGGGGTGTACAACGTCGTCCGGTCCAGCGAGGCCGCCACCCGACAGCTGCAGGCGGCGATCTTCC

ACGCGCTCCTCAACGCCACGACGTACCGGGACCTGGAGGAGGACTGGCGCCGCCACGTGGTGGCCCGCGG

CCTCCAGCCGCAGCGGCTGGTTCGCAGGTACCGGAACGCCCGGGAGGGCGATATCGCCGGGGTGGCCGAG

CGGGTGTTCGACACGTGGCGATGCACGCTCAGGACGACGCTGCTGGACTTTGCCCACGGGGTGGTAGACT

GCTTTGCGCCGGGCGGCCCAAGCGGACCGACCAGCTTCCCCAAATATATCGACTGGCTGACGTGTCTGGG

GCTGGTTCCCATATTGCGCAAGACGCGCGAGGGGGAGGCGACGCAGCGCCTGGGGGCGTTTCTCAGGCAG

CACACGCTGCCCCGGCAGCTGGCCACGGTCGCCGGGGCCGCGGAGCGCGCCGGCCCGGGGCTTCTGGATC

TGGCCGTCGCGTTCGACTCCACGCGCATGGCGGAATACGACCGCGTGCACATCTACTACAACCATCGCCG

GGGGGAGTGGCTGGTGCGCGACCCGGTCAGCGGGCAGCGCGGCGAGTGCCTGGTGCTGTGCCCCCCCCTG

TGGACCGGCGACCGCCTGGTCTTCGATTCGCCCGTTCAGCGGCTGTGCCCCGAGATCGTCGCGTGCCACG

CCCTCCGGGAACACGCGCACATCTGCCGTCTGCGCAACACCGCGTCCGTCAAGGTGCTGTTGGGGCGCAA

GAGCGACAGCGAGCGCGGGGTGGCTGGCGCCGCGCGGGTCGTCAATAAGGCGCTGGGGGAGGATGACGAG

ACGAAGGCCGGCTCGGCCGCCTCGCGTCTCGTGCGGCTCATCATCAACATGAAGGGCATGCGCCACGTGG

GCGACATCAACGACACGGTACGCGCCTACTTGGACGAGGCGGGGGGGCACCTGATCGACACCCCCGCCGT

CGACCACACCCTCCCTGGGTTCGGCAAGGGCGGCACCGGCCGCGGGTCGCGCCCCCAGGACCCGGGGGCG

CGACCGCAGCAGCTTCGCCAGGCGTTTCAGACGGCCGTGGTCAACAACATCAACGGCATGCTGGAGGGCT

ATATCAATAATCTCTTTGGAACCATAGAACGCCTGCGAGAGACGAACGCGGGTCTGGCGACCCAGCTGCA

GGCGCGCGACCGCGAGCTGCGGCGCGCCCAGGCGGGGGCGCTGGAGCGGGAGCAGCGCGCGGCGGACCGG

GCGGCCGGGGGAGGCGCGGGCCGCCCGGCGGAGGCGGATCTTCTCCGGGCCGACTACGACATTATCGACG

TCAGCAAGTCCATGGACGACGACACGTACGTGGCCAACAGTTTCCAGCACCAGTACATCCCCGCGTACGG

CCAGGACCTCGAGCGCCTGTCGCGCCTCTGGGAGCACGAGCTGGTGCGCTGCTTCAAGATTCTGCGCCAC

CGCAACAAGCAGGGCCAGGAAACGTCGATCTCGTACTCTAGCGGGGCGATCGCCTCCTTCGTGGCCCCGT

ATTTCGAGTACGTGCTTCGCGCCCCCCGAGCGGGCGCGCTCATCACCGGCTCCGATGTCATCCTAGGGGA

GGAGGAGTTATGGGAGGCGGTCTTTAAGAAAACCCGCCTGCAGACGTACCTGACAGACGTCGCGGCCCTG

TTCGTGGCGGACGTACAGCACGCGGCTCTGCCCCGGCCCCCCTCCCCAACCCCCGCCGATTTCCGGGCGA

GCGCGTCCCCGCGGGGCGGGTCCCGGTCCCGGACCCGGACCCGATCCCGGTCGCCCGGGAGAACGCCGAG

GGGTGCGCCGGACCAGGGCTGGGGCGTCGAACGCAGGGATGGCCGACCCCACGCCCGCCGATGAGGGAAC

GGCCGCCGCCATCCTCAAACAGGCCATCGCCGGGGACCGCAGTCTGGTCGAGGTGGCGGAGGGGATCAGC

AACCAGGCGCTGCTGCGCATGGCCTGCGAGGTGCGCCAGGTCAGCGATCGCCAGCCGCGGTTTACCGCGA

CCAGCGTCCTGCGCGTTGACGTCACCCCCAGGGGGCGGTTGCGGTTCGTTCTGGACGGGAGTTCCGACGA

CGCGTACGTGGCGTCGGAGGATTACTTTAAGCGCTGCGGGGACCAGCCGACGTATCGCGGTTTTGCGGTC

GTCGTCCTCACGGCCAACGAGGACCACGTGCACAGCCTGGCCGTGCCCCCCCTCGTTCTGCTGCACCGGC

TCTCCTTGTTTCGCCCCACGGACCTCCGGGACTTCGAGCTCGTCTGCCTGCTGATGTACCTGGAGAACTG

TCCCCGGAGCCACGCCACGCCCTCGCTGTTCGTCAAGGTGTCGGCGTGGTTGGGGGTCGTGGCCCGCCAC

GCGTCTCCCTTCGAGCGCGTCCGCTGCCTTCTCCTCCGCAGCTGCCACTGGATCCTGAACACGCTAATGT

GCATGGCGGGCGTGAAGCCCTTCGACGACGAGCTAGTCCTGCCCCACTGGTACATGGCCCACTACCTGCT

GGCCAACAATCCGCCCCCCGTCCTCTCGGCCCTGTTTTGCGCCACCCCGCAGAGCTCTGCGTTGCAGTTG

CCCGGGCCCGTCCCCCGCACGGACTGTGTGGCCTATAACCCGGCCGGCGTCATGGGAAGCTGCTGGAATT

CCAAGGACCTGCGTTCGGCTCTGGTGTATTGGTGGCTTTCGGGGAGCCCCAAACGACGGACCTCGTCGCT

TTTCTATCGGTTTTGCTAACTCCGGAAAATAAACGTGTTTTTTATGGAACGTTCCCCACCTGTCGTGTCA

TCTCTCGGGGGATGGTGGTGGGCCTGTGTGTGTGTCTTGTGCACCGAAGGAGGAAAGTGGGGGGGTGGTG

GTGCTGGTGGTGGAAAGACATGATAGAGGGAACAAAGAAATAGAAGAAAACCACAACCGGCGCGTGCCAG

TAAATACGGACGCGCGCACACGCGGGGGGTAAGTTGGAGCACGGGGCCCCGGTTTATTGACCAAATTCAG

GGAAACAGAAACCGAATCTTTTCATCGAAAGGGTACACAAAGCTCCCGCCCTCGCCCCACACGCCTTCCA

GAACCCCCGTAAACACCAGTTGAATCTCGCGCAGGATCTCGCGCAGGTGATGGGCGCAGTCCACGGGGGG

GAGCACCAAGGGCCGCGGGTACAGATCCACGGGGACGCCGACCGACTCCCCGCCCCCGGGACATACGCGC

ACGACGCGTCTCCAGTATTGCTCCGCGTCCAGCAGGGCGCCTCCGCGGAAGGCCGTTTGGGGCAGGGGGT

CGTCGGCCTCGCCTGGGGGGGTCAGAACGCTCCAGTACTCCGCGTCCAGACGCCTCCCGAAGGCATCCAG

GACAAAGCGGTCACAGGCGTCCTCCATGACGCCCCGGGCCGCGCACACGGCCTCCTCCGGCGGGCCGGCG

GCCGGCCGCCGGAGGATTCGTCTCAGCGCGTCGCGCATAACCTCGGCCGCCGCGGCGTACGCGGCCCCGC

GGAGAGGAAATCCCTGCAGGAAGTCGGTGTCATCGCGGGAGTTCCAGAACCACGCCCCGGTCTGGCTCCA

GGTGACGACGTGGGTGTAGACGCCCTCTGGCGCCAGGGAGGGGGCGAGGCGCGGGCGTATGCCGTTGGCC

GAAAGTACGGCGCGCACGGACGCCTCGAGGGCCCGGCGGGCGTCCTGGATCGCGCCGTGCGCGGCGTCCG

CGTCCCCGGGGTCCACGTTGAACAGCCCCCAGAACGCAGCCCCGGTGCCGCCGCAGACCGCAAACTTCAC

CGAGCTGGCCGTCTGCTCGATCTGCAGGCAGACGGCGGCCATGACCCCGCCGAGCAGCTGCCGGAGCGCG

GGGCAGGCGTCGCACGCGTCCGGCACCAGGCGCTCCAGCACGGCCCGGGCCCAGGGCTCCGAGGGGGCGG

CCGCCACCAGCGCGTCCAGCCTTTCCAGGCCCGCCCGCCCCCGGGCTTCCGGCAGCCCGGCCTCCCCGAG

GCCCGCGAGGGCGGCCAGGAGCTGGGCCTGGAGCCCGGAGAAACAAAACCGCGCCGTCCAGACCGGCCCG

ACGGCCGCCGGGGGGTCGAGTAGTTGGATGGTGGTGGCCGTGGGGTGCCACCGCGCGACCGCTTCCCGAA

AGGCGGGCAGGAGGCGGCCGGCCGCCTCCGAGGCCACGGCCGGCCATGCCCGCGGGGGCAGGACGACCCT

GGCGCCCACCGCGGGCCAGGCCCCCAGGCACGCGGCATGGGTGGCCGCGGCGCCCCGCACCAGGTCACGC

GCCGACTCGGCGGCGGCGGCGGCCGGCACGGTAAACGTGGGCCAGCCCGGAAATCCCAGCACGGCAAAGT

ATTGGACGGGCCCTCCCCGGACCTCAAACCCGGGCCCCAGAAAAGCGAAGACGGGGGCCAGGGCTCCGGG

GGCGGCGTGGACCGTGGTATGCCACTGCCGGAAGAGGGCGACCAGCGCCGGGGCGGAGAACCCGTCGCCG

GCGCTCACGAAGTAGTCGTAGCCGCGCGGCAGCAGCACCCGCGCCGTGACCCGCTGCGGGTGTCCGCGGG

GCCGCAGGCCGACCTCGCACACCTCGACCAGGTCCGCGAAGGCGCCCTCCTTCCTGGTCGGCGGAAACGC

CAGGGTGGTGTATTCGCGCGCAAAACGCGCGGTCCTCGTCGTGATGGTGACGGCGAGCGAGGCGGAGGAC

GCGCACTGGGGGCTGTCGCGAATGGCGGCCAGGCGCGCCCACGCCAACCGCGCGCCGGGGTGCTCGGCGA

CGCGCGCGGACAGGGCCAGCGGGTCGACGTCGACCTTGGCCTCCACGTCCAGGAGGGCGGCGCGAGGAGC

GGCCGGCGGGCCCCACGACGCCCTTTCGACCCTCACGACCAGACCCGTCTGCGGGTCCCAGCCCAGGCGC

AGCGGGACGAAGAGGGCCCACCGGCCCGTCTGGCGCTCCAGGGCCGCCAGAACGCACGCATACAGCGCCC

GCCACAGGGTCGGGTCCCCCAGGGGCTCCAGCGGGGAGGCGGCCGGGGCCGTCGCGGCGCGGGCGGCCGC

GACGGCCCCGGGGGCCGAGACGTCGGGGGAGCCGTAGAAGTCCTGCAGGTCGGACGAACCAACGGACACC

TCCGCGAAGCGCGCGCGCGCCTCCCCCGCGGCGTCGCGACAGACCAGATACAGCAGGGCGTGGAGGCAGT

CGCGCGTGCGCGGGGGCAGCCATACCGCGTATAGGGTAATGGCGCTGACGCTCTCCTCCACCCAAACGAT

GCCGGGGGCTTCCATGCCACGACGCCCGGGGGTTGCCGTGTATCGAACGAGCGCGGCCCCAGACTTATAG

GGTGCTAAAGTTCACCGCCCCCTGCATCATGGGCCAGGCCTCGGTGGGAAGCTCCGACAGAGCCGCCTCG

AGAATGATGTCAGTGTTGGGCTGGGCGCCGGAGGCGTGCGTGCGCAAGCAGCGCCCCCACGCGGGCGCGC

GCAGCTTGAAGCGCGCGCCCGCAAACTCCCGCTTATGGGCCATCAGCAGCGCGTACAGCTGTCTGTGCGT

CCGGCAGGCGCTGTGGTCGATGCGGTGGGCGTCCAGCAGCTCCACGATGGCTCGCTTGGTGAGGTTTTTA

ACGCGCCCCGCCCCGGGAAACGTCTGCGTGCTCTTGGCCAGCTGCACCCCGAACAGTTCGCCCCAGATGA

TCTTGAACAGCGACAGCGCGTGCTCCGTCTCGCTCACGGACCCGCGCGGGGGGCAGCCGCTCAGGGCGTC

GGCCACGCGCTTAACCGCGTCCTCCGACAGCAAGGGGCCGTCGGTCACGTTACAGTGGCCCAGTTCGAAC

ACCAGCTGCATGTAGCGGTCGTAGTGGGGGTTCAGCAGCTCCAGCACGTCCTCGGGGCTAAAGGTTCGCC

CCGACCCCCCGGCCATCGAGTCCCACTGCAGGCACGCGGCCATGGTGCTGCACAGACGGAACAGCTCCCA

GACGGGGGCGACGTTTAGGGTGGGGTGTAGGGCCACAAGCTCCAGCTCTCCGGCGGCGTTGATCGTGGGG

ATGACGCCCGTGGCGTAGTGGTCGTAAAGCCGCCGGAAGATGGCGCTGCTATGGGCGGCCATGGGGACGC

GAAGACAGGCCTCCAGCAGCACCAGGTAGATGAACCGCGTGCGGCCGACCAGGCTGTTGAGGCCGCGCAT

GAGCGCGACCACCTCGGCCGGCGCGACGTCCGGCCGGAGGTACTTTTCGACGAAAAGGCCCACCTCCTCC

GTCTCGGCGGCCTGGGCCGACAGGGACGTGTCGGGGTCCTGGCAGCGCAGCTCCCGCAGATCCCGCTGGG

CCCTCAGGGCATCAAAATGTATCCCCCGCAAAAACAGACAAAAGTTCCTCGGGGTCAGCGCGGCGTCGTG

GCCCCAGAACCGCACGTGCATGCAGTTGAGGGTCAGAAGCATGTGGAGGATGTTAAGACTGTCCGCGAGG

CACGCCAGCGTGCACCTCTCGAAGTAGTGCTTGTACCGGAATTTGCTGTAGATGCGCGACCCCCGCGCCT

GCGCCGCGTCGGCGTGCGACGCGTCGCAGCGCCCTTTGAACCGGCGGCACAACAGGTTCGTCACCTGGGA

AAACTGTGCCGGCCACTGCCCGCTGGCGCTCACCACGTGGTTGAGCAGCATGGGCGTAAAGACGGGCTCC

GAGCGCGCCCCGGACCCGTCCATGTAGATCAGCAGCTCCCCCTTGCGGAGAGTCCGTACCCGCCCCAGCG

ACTGGTACACGGACACCATGTCCGGCCCGTAGTTCATGGGTTTCACGTAGGCGAACATGCTGTCAAAGTG

CGGCGGATCGAAGCTAAGGCCCACCGTCACGACCGTTGTGTAGATGACCACCCGGTACCGGCCCCATGTG

GTCACGTCGCCGGGCGGGGTGAGCGAGTGGAGCAGCAGCACGCGGTCCGTAAACTGCCGGCAGAACCTGG

CAACGACCTCCGCGAAGGAGACCGTCGACGAGAAGATGCAGACGTTATCTCCGCCGGCCAGGCGCGCCTC

CAGCTCCCCGAAGAAGGTGGCGTCCGGGGGGGCGTCCGGGGGGGGCGCCCCGCCCGCCGGCCCCCGGCGG

CGCAGGGCCGCCTGCAGGACCTCGGGCCCCAGGCGCGGGAGAAACAGACAACGGCGCGCCGAAAATCCGG

GCATGGCGTACTCCCCGATGACCACGTGAACGTTCTTTTCGCCCCGGAGGCTGCACAGAAAGTCCACCAG

CTGCGCGTTGGCGGTGGCGTCCATGGCGATGATCCGCGGGCACGTGCGCAGCAGGCGCAGCATCAACGCG

TCGACGCGGCCCAGCTGCTGCATCGTCGGCGAGTACAGTTGGCCCAACGTCGACATGACTTCGTCCAGGA

CGAGCACGTCGTAGTTGTTCAACAGGTTCGGGCCCACGCGATGAAGACTTTCCACCTGCACGATGAGACG

GTGGAAGGGGCGGTCGTTCATGATGTAATTGGTGGATGAGAAGTAGGTGACGAAGTCGGGCAACCCTGAC

TCAGCGAACCGCGTCGCCAGGGTCTGAGTAAAACTCCGACGACAGGAGACGACCAGCACACTCGTGTCCG

GAGAGTGGATCGCTTCCCCCAACCAGCGGATCAGCGCGGTAGTTTTTCCCGAGCCCATTGGCGCGCGGAC

CACAGTTACGCACCGGGCCGTCGGGGCGCTCGCGTCCGGGAAGGTGACGGGTCCGTGTTGCTGCCGCTCG

ATCGTTGTTTTCGGGTGGACCCGGGGAACCCACTCGGCCAAATCCCCCCCGTAAAGCATCCGCGCCAGCG

ATACACTCGACGTGTACTGCTCGCACTCGTCATCCCCGATGGGACGCCGGGCCCCCAGGGGATCCCCCGA

GGCCGCGCCGGGCGCCGACGTCGCGCCCGGGGCGCGGGCGGCGTGGTGGGTCTGGTGTGTGCAGGTGGCG

ACGTTCATCGTCTCGGCCATCTGCGTCGTGGGGCTCCTGGTGCTGGCCTCTGTGTTCCGGGACAGGTTTC

CCTGCCTTTACGCCCCCGCGACCTCTTATGCGAAGGCGAACGCCACGGTCGAGGTGCGCGGGGGTGTAGC

CGTCCCCCTCCGGTTGGACACGCAGAGCCTGCTGGCCACGTACGCAATTACGTCTACGCTGTTGCTGGCG

GCGGCCGTGTACGCCGCGGTGGGCGCGGTGACCTCGCGCTACGAGCGCGCGCTGGATGCGGCCCGTCGCC

TGGCGGCGGCCCGTATGGCGATGCCACACGCCACGCTAATCGCCGGAAACGTCTGCGCGTGGCTGTTGCA

GATCACAGTCCTGCTGCTGGCCCACCGCATCAGCCAGCTGGCCCACCTTATCTACGTCCTGCACTTTGCG

TGCCTCGTGTATCTCGCGGCCCATTTTTGCACCAGGGGGGTCCTGAGCGGGACGTACCTGCGTCAGGTTC

ACGGCCTGATTGACCCGGCGCCGACGCACCATCGTATCGTCGGTCCGGTGCGGGCAGTAATGACAAACGC

CTTATTACTGGGCACCCTCCTGTGCACGGCCGCCGCCGCGGTCTCGTTGAACACGATCGCCGCCCTGAAC

TTCAACTTTTCCGCCCCGAGCATGCTCATCTGCCTGACGACGCTGTTCGCCCTGCTTGTCGTGTCGCTGT

TGTTGGTGGTCGAGGGGGTGCTGTGTCACTACGTGCGCGTGTTGGTGGGCCCCCACCTCGGGGCCATCGC

CGCCACCGGCATCGTCGGCCTGGCCTGCGAGCACTACCACACCGGTGGTTACTACGTGGTGGAGCAGCAG

TGGCCGGGGGCCCAGACGGGAGTCCGCGTCGCCCTGGCGCTCGTCGCCGCCTTTGCCCTCGCCATGGCCG

TGCTTCGGTGCACGCGCGCCTACCTGTATCACCGGCGACACCACACTAAATTTTTCGTGCGCATGCGCGA

CACCCGGCACCGCGCCCATTCGGCGCTTCGACGCGTACGCAGCTCCATGCGCGGTTCTAGGCGTGGCGGG

CCGCCCGGAGACCCGGGCTACGCGGAAACCCCCTACGCGAGCGTGTCCCACCACGCCGAGATCGACCGGT

ATGGGGATTCCGACGGGGACCCGATCTACGACGAAGTGGCCCCCGACCACGAGGCCGAGCTCTACGCCCG

AGTGCAACGCCCCGGGCCTGTGCCCGACGCCGAGCCCATTTACGACACCGTGGAGGGGTATGCGCCAAGG

TCCGCGGGGGAGCCGGTGTACAGCACCGTTCGGCGATGGTAGCCGTTTCGTTCGTTTTAATAAACCGACG

TTGTGCGTTTCACCATACTTCGGCGCGCGTGTGTGTGTGTTTTTTTTTTTGTGGTGTTTATTTTCCCCCC

ACCCCTTCCTTTTCTTTCGGCCACCACCCCCCTCCTCCCCCGTACTATACAACAAAAAATACCACACATA

CGACCAAATACGGACAATCATTTCTGTCTTTATTCGCTATCAGAGAGTGGGGGCGTGAGCGTGGCAGGAG

GGCGGGCCACGTCGGGGTCCCGCCGTCTGGTGTGACGCGATGGGGGGTCCGATGCGCGCCGGTACTGGGG

CCCCGGCGCCCGGGTGACCACGCGCACGTCGGGGGGCACGTAGAAGTTACCCTCTTCTTCGGACTCGATG

TCCACGACGTCAAATTCGTGGGCGGTCAGCGAGACGACCTCCCCGCCGTCGGTGGTGATGACGTTGTGTC

GGCAGCAGCAGGGCCGCGCCCCGGAGAACGCGAGGCCCATAACTTGGCGAGCGTATCGTCGAAGGCCAGG

CGGCTGTTTCGCCGGATGTCCCGGTAGATCCCCGGCTCGACGCGGACGGGGGTGATGATCAGGGCGATCG

GAACGGCCTGGTCCGGGAGGATCGATGCCTTGGCGGGTCCGGGGGCCCCGCCAGGCCCGGCGGGCGCTCC

GCGGCCGTCCTCCAGGCGGAACGTCACGCCCTCCTCCGCGCCCGCGCGGTGCCTGCCGAGGAACGTCACC

AGGTGCGGTTGCAGGGGGCAGTCGGGAAAGTGGCTGTCGAGGACGTATCCCTGCACCAAGATCTGTTTGA

AGTTCGGGTGGCGGGGGTTGGCGAAGATGGGCTCGCGGCGAACCAGCTCCCCGGAGCTCCAGGCCACGGG

AGAGATGGTGCGACGCTCAAGGTCGGGGACGCCAAACAGAAGCACCTCCGAGACAACGCCGCTATTTAAC

TCCACCAGCGCCCGATCCGGGGCGGAGCATCGCCTTTTTTCGCCGGCGGCGCGGGAATCGAGCCAGTCCC

GGTCTTGGGTGACGAGCGCCTCCTCCGGGCCCGGAACGCGCCCGGGCGCGAAGTAGCGCACGCCGGGGTT

GGGGATGGACCGGATGAACGCCCGGAACGCCTCCGGCGATCGCCGCGCCATCAGGTCCTCGTACGCGGAG

GCCGCGGGGGCGCCGGGGTCCGCGGGGTCGAACGCGTACTTGGCTCGGCACTTAACCTCGTAGAAGGCCA

GGGGGGTCTGGGGGGCGGGGGCCAGGTAGCCGTGAGGGTCCCTGGGGCACACGAGGATGTCCAGGGACGC

CCCCACCATGCCCGTGTGGCCGTCCATGAGGACCCCGCACGCGTGCACGTTCTCCTCGGCGAGGTCCCCG

GGTTGGTGAAAGACGAAGCGCCCGGCGTCGGCGTCGTCGTTGACGCCCGCGTCCGCGCGGCCCACGCAGT

AGCGAAACAGCAGGTTTCGGGCCGTCGGCTCGTTCACCCGCCCGAACATCACCGCCGACGACTGGGCGTC

CAGCCGCAGGCTGGCGTTGTGGGTGAGCCACTGGGACGAGAAGCACGGACCCTGCGCGCCCCACCGCAGC

GTGGAGGCGGTCGTCAGGCCCCGCCGAAGCAGGGCCCAGAGCTGGCAGTCGGCCTGGTTTTGCGTCGCCG

CCTCGTAAAATCCCATAAGCGGGCGGGGGGCGACGGCTTCGGCGGCGGACGGGGGGGCGCGGCGCGTCAG

GCGCCAGAGGTGCCGGCCGAGCCCGCGGTCCACCATGCCGGCCGCCTCCAGCGACACGACGAGGGAGCAC

AGATAGTCCAGGCGAGCCCACAGGGGCCCGATGGCCAGAGGGGAGCGGACGCCGCGCAGCAGGCCGCGCA

GGTGGCGCTCGAACGTTTCCGCCAAGATATGGGGGGGCAGTGCGTTGGGGATCGCCGACGCCGACCACAT

CGGGTCGGGGTCCGGGGGACCGGGGCTGCAGTCCGGGTCGATGGCGTGTGCGCCCCCCGGCGAGAGGGGA

ATGTCGGGGGTTGGCGGGCCGGATGAGGCCTCAGAGAGGGCCGGGGACGCGGGCCGGGCCTTTTCGCCCG

GGGCCCCGCCGTCGGGTTGCCCACGTGGGGGGCTCTGGGGCCAATGGGAACCCGGGGCCCCCGGTGACGT

GGGGCGGGGTGGGGCGGGGCGGGGCCCAAAGACGGTCGCCAGATCTAGGCTGTTGGGTCGGGGCCGCTTC

GGGGGACTATCGGGGTCGCGGGCGGGGTCCGCGGGGCGCTTGGCGCCGGGTGTTGCGGCGGCCGCCATTT

TTACGAGCAGCCGAAGAGCTCGAGGGCGGAAGGGATCCTCACGACAGAGAGTGGCGCGCGGCCGGGTTGG

CGTGACAGAGGCGGGAGACCAGCACCAGCAGCGGCCTCAGCTCGGGCGGCAGCGACACCGACGACAGGAC

GGCCTTGTGCGTGCGCTGGTAATTTATACACTGCTCCGTGAACGCGCGCCGAATCTTGGGATTGCGAAGG

TGGCGCCGGATGCCCTCCGGCACGTCATACGCCAGGCCGTGGGTGTTGGTCTCGGCCGAGTTGACAAAGA

GGGCGGGGTGCAGAACGCAGCGATAGGCGAGGAGGGCCACGGCAAAGTCCGGCGAGAGCTGGTTGTTAAA

GTACTGGTAGCCCGGGACGCGGGTCACGGGGACGCCCAGGCTCGGGGCCACGTACACGCTAACCAGCAGC

TCCAGCAGCGTCTGCCCCAGGGCGTAGAGATCGACCGCCAGCCCGACGTCGTGCTTCAGGGGGCGGTTGT

TAAACTCGGCCCGCTCGTTGTTGAGGTACTTTACCGAGAGCTCCGGTGGCTGGTTGTACCCGTGCCCCAC

CAGAGTGTGAAAGTTGGCCGTGGTCAGGGCGGCGGGCATCCCAAACCCCCGGGGGGACTCGAGGTCCGGC

TCCTGGAGGCAAAACTGGCCCCGGGATATCGTGGAGTTGGAGTTCAGGGTCACCAGGCTAAAGTCGGCCA

GGACGGCCGGCCGGAGCGACACCGCGTCCGATCGCAGCATCACGAGGACGTTGGCGCACTTGATGTCCAG

GTGGCTGATCCCGCACCTGGTGTTCAGGAACACCACGGCGCGCGCCAGGTCTGTGAAGCAGTGGTGGAGG

GCCGTCGCGACGGAGGGGGTGGTCGCGCGCAGGGACGCCAGCTGGCCGATGTACTTGCCGAGGTCCATGT

CGTACGCGGGGAACACGATCTGGCGCTGCTGCAGCGAGAACCCGAGCGGGGTGATAAAGCCGCGGATGTC

GTGGGTGCGGCCGCCGCGAAGAGCGCACTCCCCCACGAGCAGGGTCGCGACGAGCTCCACGGCAAACCAC

TCTTTTTCCCGGATGGTCTTCACGGCGAGCTTGTGTTCGCGAATCAACTGCACCTCGCCGTACCCCCCCG

AGCCCCCGAAGCTGCGGGCCCCGGGGATCTCCAGGGTCGTGTAGCGGAGGGCGGGGTTGACGGCGAATAC

GGGGATGCATAGCTTGTGGATGCGCGCGAGGGACAGGATGTGCGAGGGGGGCGACGGGGGCGAGGTCATG

GCCGTCTCGGACCTGCGCAGGGGCGGGCGCCTTAGCTTGGCCGCAGGGCCGGGGGCCTCGGGGGACGAGC

GGCGACGAGACGAGCGGCTCACTCGCCATCGGGACAGTCCCGCGCGAAGCCGCTCCCGGAAGCTGGATCG

GCGGCGGGACCCGGGGCGGGCTCCGGAGACGGCGCCGTCTCGGGGGGAGGGGCCGCTTGGGCGTCCGGAC

GCCCGGCGGCTGAGGGAGTGTATGTAGGACGCGAGCCAGGCCTTGAAGGAGCGTCGGTGTGCACCTTGGG

GGCTGATGTCAGCTGCCACATGACTAGCAGGTCGCTGTCGCCCGGACTCATCCATCCGTCCGCCAGGTCG

CCGTCCCCCCACAGAGACGCGTTCGCCGCGGCCTCTTCGAGCTGCTCCTCCTGGTCCGCAAGACGATCGT

CCGCCGCGTCCAGGCGCTCGCTAAGCGCGGGATCGAGGTACCGTCGGTGTGCGGTTAGAAAATCACGTCG

CGCCGCTTGCTCTTCCACGCGAATTTTAACACAGGTCGCTCGCTGTCGCATCATCTCTAAGCGCGCGCGG

GACTTTAGCCGCGCCTCCAATTCCAAGTGGGCCGCCTTGGCGGCCATAAAGGCGCCAACAAACCTAGGAT

CTTGTGTACTCACGCCCTCCCGGTGTAGCTGCAGGGTCTGGTCCCTGTACACCTCGGCCCGGAGGTGCGT

CTCGGCCAAACGTCGGCGCAGGGCCGCGTGGCTGGCGTCTCGGCTCATCTCGCCGCCCCCGCGCGCGCCC

GACGTCGGACTCCTTCGCCCCGACCCCCCTGACCTCAGCCGCCCCCGCCTCGCCCGCGATGTTTGGCCAG

CAGCTGGCGTCCGACGTGCAGCAGTACCTGGAGCGCCTGGAGAAACAGAGGCAACAGAAGGTGGGCGTCG

ACGAGGCGTCGGCGGGCCTGACGCTCGGCGGCGATGCGCTGCGCGTCCCTTTTTTGGATTTTGCCACCGC

GACGCCCAAGCGCCACCAGACCGTGGTCCCGGGCGTCGGGACGCTCCACGACTGCTGCGAGCACTCGCCG

CTCTTCTCGGCCGTCGCGCGGCGGTTGCTGTTTAATAGCCTGGTGCCGGCGCAACTCAGGGGGCGTGACT

TTGGGGGCGACCACACGGCCAAGCTGGAGTTCCTGGCCCCCGAGCTGGTGCGGGCGGTGGCGCGCCTGCG

GTTTCGGGAGTGCGCGCCGGAGGACGCCGTGCCCCAACGCAACGCCTACTACAGCGTCCTGAACACGTTT

CAGGCCCTGCACCGCTCCGAAGCCTTTCGGCAGTTGGTTCACTTCGTGCGGGACTTCGCCCAGTTGTTGA

AAACCTCGTTCCGGGCCTCTAGTCTCGCGGAGACTACGGGCCCCCCGAAGAAACGGGCCAAGGTGGACGT

GGCCACCCACGGGCAGACGTACGGCACCTTGGAGCTCTTCCAGAAAATGATACTAATGCACGCGACCTAC

TTTCTGGCCGCCGTGCTGCTCGGGGACCACGCGGAGCAGGTCAACACGTTCCTGCGGCTCGTGTTCGAGA

TCCCCCTGTTTAGCGACACGGCCGTGCGGCACTTCCGCCAGCGCGCCACCGTGTTTCTAGTCCCCAGGCG

CCACGGAAAGACCTGGTTTTTGGTGCCCCTCATCGCGCTGTCGCTCGCGTCCTTCCGGGGGATCAAGATA

GGCTACACGGCCCACATCCGCAAGGCGACCGAGCCCGTGTTTGATGAGATCGACGCCTGCCTGCGGGGCT

GGTTTGGCTCGTCCCGGGTGGACCACGTCAAGGGGGAAACCATCTCGTTCTCGTTCCCGGACGGCTCGCG

CAGCACGATCGTGTTTGCCTCCAGCCACAACACGAACGTAAGTACGCCTTCCTCCCGCGGTGCCTGTTTC

CCCGGTGCCGCCCTCCCCGAGATCGACCGACAGACAAACACAGCCAGACGCGAGTGTGGGACGACACGCC

CGCAGCCCCCCCCCCGCCATGGCGGGGGGGAAGCCTTACTGTTTATTTGTAATCGGACGATGAGGCTCTG

GCCACGGCCCGCGCGACCGCGGGGCAGCTCGTTGCAAACAGGCGGCTGGTATACGATGACAGAACGCAGA

GGCGCCACCCGGCGCTGGTCGGGCGGATGACGCTTTCCGCGCCGTCCCGGCCCACGACGACCTCGTGCAG

GTGGGCCGTGATGCGCGGGCGGCGGGTCGCCTGCCGCAGGATAACCGCGTCCACGGGGTGCCCGAAGAGG

AGCTGACACAGGCTCGCGTCCCCCCGGACGGCCAGGGTGCGCTGGGCCATATTGGACCACATGCACGGGG

CGACGCAGGGACAGGCCTCCGCCACGGCGGGGGCGCGCCACAGCGCGTTGGCGGAATCGATGTGGGCCGT

CGGGGCGCAGGCGCCGCCTCCTCCCGGGGGGTCGGTAATCCTGGATAGCAGCCATCCTAAATGGCGGGCC

CGGCTGCCCGGGGGACAGAGCGACCCCAGGTCATCATCCATGGCCCAGCAGTATATGCGGCCGCCGGGGA

GGTGCCACCAGGCCCCCGGACCCAGGGCACAGCACGCCCCGGATTCGGGGGCCGTGTCCGTGGGTACCAG

GTAGGCGCCGTCGAGCTCGTGGGCCACGGGCTCGTCCGCGAGCTGTTCGGCGGCGGGGTCGGGGGTTTCC

TCCGGGGGGGAGGCAGCTTCCAGGTGGCCGAAGGCTAGGGTGCACAGCAGCGGGGTCCGGGGGTGCGTTA

CGCTGCGGAGGTGGACGGTGGCGCAGTAGCGGCGCTCGCGGTTAAAGAAGAAAATGGCAAAGAACGTGTT

CGAAGGCAGGCGCAGCGCCTTGGGCCGCGTCAGGTACAGGAAGATCTCGCAGAAAAGGGCACGCTCGGGG

TCGGGGTCCGGAAGGGCCACCTGGCACAGCGGCTCGGTGAGGACCGTGAGGCACCGAAAAATCTTAAGCC

GCTCGTCCCCCCGAACGACGCGCCACACGAAGACAGAGTTGGCGATGCGCGCGACGAGGTCGGCTTCGGG

CCCCGGGTCGGGGGCGCGCGCGTCGGGGGGGGCGCCCCGGTGACCCGGCGGGGCCGCGGCTCCCGGGGGG

CCTGGCGTCGCCTGGGGACGCCAGAGTGCCCGCTGTGCCAGGTTGGTGGTGGGGAAGGGACCGGAGACGC

ACCAAAAGCAGAGGGGCCAGCGCGTGTATGAGTTGGGGGGGGGGTGGGTGAGCGGTGGAACAAAAGCACG

CGTCAGCGGACAAGGCCGGGTCCCGTAGCCGCCCCGCGACAGAACCGGAGTCCGACGGCACGCGCGACGG

GGTCTGCGAGGCTGAGGTACGCCGCGGTGTTAATGGTAAACGCAAAGCCTCCCGGAAAGACCACTAGCCC

GCAGAGGCGGCGATTGAACCCAAGGCAGAGGTACGCGTAGCTCTCTCCCGGAAGGTATTGCTCGCAGACC

CTGTGTGGGGCAGTGGAGGGGCTGCCCTCCATGAAGCGACATTTACTCTGCTCGCGTCCATTGACGTCAC

CGTCAATCACCACTGCGATTGGACGGTTGGTGAGGCGCAGCGTGTCTCCGCTGGTGCTGTAGTAGTCAAA

CGCGTAGTGGGCGTCGGAGTCGGCGAAGCGGGCGGGGATGTCGTCGCTGAGAGGGACGAGCCGCCGCCGC

CGCCCCCGACCGCCCTGGCCGCCCAGATGCGCCAGCACGGCCAGGGCGTACGCGGTGTGAAAGAACGCGT

CGGGGGCGGTCCCCTCGAGGGCGCGCATCAGGTTCTCCAGGAGCACGGGGAAGCGCCGCGTCACCTCCCC

TAGCCACTCGCTCTGGTGGGGGCCAAAGTCGTAGCGCAGGCGCTGGAAGATGCGCGGGCCGCCTTGGAGC

GCGGCCCGGATAGAGTGGCCCAGGGCCCGCAGACACGCGATCTGGATGCGCGCGACGAAGGCCACCTCGG

CCGCGATGTCAAAGGGCTGCAGCACGGGGCGCGGGTGGCGCAGGGGTCCCTCGAGCGCGGGAAAGCGACG

CAGCAGCGCCGTCTGGGCCGCGGGGGACAGCTGGTGGGGGCGCACGACGCGCTCGGCGGCACAGGCCTCC

GTCAGGGCCGTGGCCAGCTCGGAGGACAGCCGCGGGGGGCGGGCGCGTCGCCCGCCCCACGCCACCGAAT

TCTCGTAGGAGACGACGACGAAGCGCTGCTTGGTCCCGTAGTGATGGCGCAGGACCACGGAGATGGAGCG

ACGGCTCCACAGCCAGTCGGGCCGGTCGCCGCCGGCCAGAGCTTCCCACCCGCGGTCCAGCCACTCGACC

AGCGATCGCGGCTTGGCGGTCCCCGGCACGAGGGTGAGCACGTCGTTGAGGACGTCCTCGCCCGCGGCCC

GGGGGCCCCCCCGGCTGGCAAAGCGCCCCCCGCCGGGCGGCTCCAGGCCCGCCAGCACCGCCTCCGCGTC

CGACGCGCCCAGGGCTCCCCCGCTGACGGCCTGGTGGACCAGGGCGCCCTGGCGGAGCCCCGAGGCGACG

CCGGAGGCCGCGTGCTTGGGGCGCGCGCGGACCGGGTGGCGGCGGGTGACGTCCTGCACGGCCCGCTGGA

CCAGCGCGAGGATCTCCTCGTTCTCTTGCGTGATGGACACGTCCTCCGCGGTGGCCGTGTCGCCTCCCGG

GGCCGTGAGCTGCTCCTCCGGGGAGATGGGGGGGTCTGGGGTGCCGACAACGGCCGGCCCGGCCCCGCCC

GAGACCGAGGACGCCTGGGGAGTGGGGGTGCCGCTTTCCCCCATCCCCAGGGACAGGTGGGCCGCCGCCT

CCGTCGCGGCGGCGGGAGCCGCGGCCCCCAGCCGCGCGACGTAGCGACAAAAGTGGCGACAGAGGCGCAT

GAGGCGCGCGCCGTCGGCCGCGTATCGCGTGTTTGGCGGGACGAGCTCGTCGTAACTGAACAGGAGCACG

CGGGCACAGGTCGCCCACGGGCCCCACGCCAGGCGCAGCGCCGCGACCGTGTACGGGTCGTACACGCCTT

GGGCGTCGCACGCGACCGGCAGGGAGACGAACAGCCCGCCCGCGCTGGGGACGCGCGGCAGGAGGTCCGG

GTGCGCCGGGATGACGGGGGCTAGGATCGCCCCCACCGCATCCGCCGGCACGTAGGCGGCAAACGCCGAA

CGCCACGGGGTGCAGTCGCCGGTCGCGTGGGCCCGGGTCTGGGTTTCGACCCGGAAGTTCGCGGCCGCCC

CACCGTCGGGGCGGCCGCGCACGAGGGCGGACAGCGGGACCCCCGCCGCCGCCAGGCACTCGCTGGAGAT

GATGACGTGAATCAGCGAGGCGGGGCTGCTCGGGTCCCGGGTGAGATCGTATTGGACCTCGTTGGCAAAG

TGCGCGTTCATGGCCCGGCCGGCGGTGCGAGCCCTTCCCGGTGCCGGAAGGGGCGTGGGTGGGGGGTGCG

TGTGCGCGTCCTCGGGGCCCGCGGGCGCACGTGCGCTTATACGCTGTGTGTTTCGTCTGTCCCCAGGGAA

TCCGGGGCCAGGACTTTAACCTGCTTTTCGTCGACGAGGCCAACTTTATTCGCCCGGATGCGGTCCAGAC

GATTATGGGCTTTCTCAATCAGGCCAACTGCAAGATCATCTTCGTCTCGTCGACCAACACCGGGAAGGCC

AGCACGAGCTTTTTGTACAACCTCCGCGGGGCCGCCGACGAGCTGCTCAACGTGGTCACCTATATATGCG

ACGACCACATGCCGCGGGTGGTGACGCACACCAACGCCACGGCCTGTTCCTGCTATATCCTGAACAAACC

CGTGTTTATCACGATGGACGGCGCCGTTCGCCGGACGGCCGATCTGTTTCTGCCCGACTCCTTCATGCAG

GAGATCATCGGGGGGCAGGCCCGCGAGACCGGCGACGACCGGCCCGTCCTAACAAAGTCGGCGGGGGAGC

GGTTTCTGCTGTACCGCCCCTCCACCACCACCAACAGCGGCCTGATGGCCCCCGAGCTGTACGTGTACGT

GGACCCGGCGTTCACGGCCAACACGCGCGCCTCCGGCACCGGCATCGCGGTCGTCGGGAGGTACCGCGAC

GATTTCATTATCTTCGCCCTGGAGCACTTTTTCCTCCGCGCGCTCACGGGATCGGCCCCCGCGGACATCG

CCCGCTGCGTCGTGCACAGCCTCGCCCAGGTGCTGGCGCTGCACCCCGGGGCGTTTCGCAGCGTTCGCGT

GGCGGTCGAGGGCAACAGCAGCCAGGACTCGGCCGTGGCCATCGCCACACACGTGCATACCGAGATGCAC

CGCATCCTGGCCTCGGCGGGGGCCAACGGCCCGGGGCCCGAGCTCCTCTTCTATCACTGCGAGCCGCCCG

GCGGCGCGGTATTGTACCCCTTCTTTCTGCTCAACAAACAGAAGACGCCCGCCTTCGAATACTTTATCAA

AAAGTTCAACTCCGGGGGCGTCATGGCGTCCCAGGAGCTCGTCTCCGTGACGGTGCGCCTGCAGACCGAC

CCGGTCGAGTATCTGTCCGAGCAGCTCAACAACCTCATCGAAACCGTCTCTCCCAACACCGACGTCCGCA

TGTACTCCGGAAAACGCAACGGTGCCGCGGACGACCTCATGGTCGCGGTCATCATGGCCATTTACCTGGC

GGCCCCGACCGGGATCCCCCCGGCCTTTTTTCCGATCACGCGCACGTCTTGAGTCTTTCTTGCCGTTTCT

TTTGTTTCTCTTTCTTTCCCCCCCTCTCTCCGCAATAAACGCCTTCCCGGAACTGTGTTTCCCCCCCTAC

AACAGTGTTGTCCGTTGGTTGGGTGGTTGGGGTGCGGGGGTGGGCGGGGGAAGCAAGAAAACGGTCGGCG

AACACAACATCGGGAAAACGGATTCCCGCACGTGCGTCTTCCCAGATTCGACACACACACCCCCCTTCTC

CTTAAATAAACACAAACCACACGCTCGTTGGTTGGTTAATGCCAGCGCTTTATTTACGTCTTGTTTTTTT

TGCGTTTCCTCCGCGGGTCCCTTCCCAACACGCCTGCCCCCGCCTCAGGGGTAGCGGATAACCGGGGCCA

TGTCGCCGGATTGCACAACGGCGGCGCCGTCGAACGTACACACCCGAACCGCCGGGGCCAGGGCCAGGAT

GTCCCCGAGTTGGCCCGCGTGCGCCAGCCAGGCGACCAGCGCCTCGTAAAGCGGCAGCCTGCGTTCGCCG

TCCTGCATCAGCATGGGGGCTTCGGGGTGGATGAGCTGGGCGGCTTCTCGCGTGACGCTCTGCATCTGCA

GGAGCGCGTTCACGTATCCGTCCTGGGCGCTCAGCGCGAGCAGCCGGGGGATGAGCGTGAGGATGAGGGT

GGTTCCTTCGGTTATGGAGTAGACCATGTTGAGGACGAGCGACCGCAGCTCGGTGTTTACGGAGGCGAGT

TGCTGGACGTCGGCCACGAGCGAGAGACGGGCCCCGTTGTAATACAGCACGTTGAGGTCGGGGAGCTCCC

CGGGCGTCCGGGGGTCGGGGTTGAGGTCCCGGATGCCCCGGGCGACCAGCCGCGCGACTATCTCGCGGGC

CAGGGGCGTTGGGAGCGGGACCGGAAACCGCAGCGTGAGGTCCAGCGACTCCAGGCGCACGTCCGTCGCC

TGGCCCTCGAAGACGGGCGGGACGAGGCTGACGGGATCCCCGTTGCAGAGGTCGACGGGGGAGGTGTTGC

GGAGATTGACGGTGCCGGCGTGCGTGAGCCCCAGGTCCACGGGGCAGGCGACGATTCGCGTGGGCAGCAC

CCGCGTGATTACCGCGGGGAAGCGCCTGCGGTACGCCAGCAACAACCCCAACGTGTCGGGACTAACTCCT

CCGGAGACGAACGATTCGTGCGCCACGTCCGCGAGCGCCAGCTGGCGGCGGATGGTCGGCAGAAAGACCA

CTCGACCCTCGCACCGCTGCAGCGCCGCGGCATCGGGGCGCGAGATACCCGAGGGGATCGCGATGTCTGC

TTCGAAACAATCCGTGATCATGGCGCCGGGCCGCGAGACACCGGAACGCGGGGGTGCGGGAGGGCCGGAA

AGCGCAACGCAACCGGGACGATGATGAAACAGAGATGGGGGGCACCGACCGTGTGGGAGAGGGGGCGGGG

CAGGGCTCAGCAGCACGCACGGGGAGGTCTGTCGTGCGCAGGAGCCCCAGGTGAGAATCAGTCCCCCGGA

GCTCGGGTCTGGGTTTTATTGGGACCTGCCCTCGGAATCGCGGCTCCCAGTCCAAGCCCCCCTGGGGGGG

GCGGGGACAGGGGGTGTGTGTGGGTAAAAGCAACGTCGGAAAATCAAACCCAATGCCCCAAACAGGAAAA

AAAAAGACGGGCGGGTGGAGGGAAAGCTGGGGAAGAAGAAGCCAATTTTACAGAGACAGGCCCTTTAGCG

GGGAGGCGTCGTAGATGAGATACTGCGTAAAGTGGGTCTCTCGCGCGTGGGCCTCCCCATCGCGGGCGCT

GCGTAGCAGGGCGGGGTCGCTGGCGCAGGTGATCGGGTAGGCTTCCTGAAACAGGCCGCACGGGTCTTCC

ACGAGCTCGCGGCACCCCGGCGGGCGCTTAAACTGCACGTCGCTGGCAGCGGTGGCCGTGGATACCGCCG

ATCCCGTTTCCACGATGAGACGCTCCAGGCAGCGATGTTTGGCCGTGATGTCGGCCGCGGTGAAGAACTT

GAAGCAGGGGCTGAGGACGGGCGAGGCCCCGTTGAGGTGATAGGCCCCGTTGTACAGCAGGTCCCCGTAC

GAGAACCGCTGCGACGCCCACGGGTTGGCCGTGGCCGCGAAGGGCCGCGCCGGGTCGCTCTGGCCGTGGT

CGTACATGAGGGCTATGACGTCCCCCTCCTTGTCCCCCGCGTACACGCCGCCGGCCGCGCGTCCCCGCGG

GTTGCAGGGCCGGCGAAAGTAGTTGATGTCCGTGGCCACGGGGGTGGCGATGAACTCACACACGGCATCC

TGCCCGTGGTCCATGCCGGCGCGCCGCGGCACCTGGGCGCAGCCAAAGACCGGGAGGGGCTGGGCCGGCC

CCAGCCGGTTTCCCGCCACGACCGCGTTGCGCAGGTACACGGCGGCCGCGTTGTCTAGCAGCGGGGGGGC

CCCGCGGCCGAGGTAAAAGTTTTGGGGGAGGTTGCCCATGTCCGTAACGGGGTTGCGGACGGTGCCCGTG

GCCGCGACGGCGGTGTAGCCCACACCCAGGTCCACGTTTCCGCGCGGCTGGGTGAGCGTGAAGTTGACCC

CCCCGCCCGTTTCGTGGCGGGCCACCTGGAGCTGGCCCAGAAAGTACGCCTCCGACGCGCGCTCGGAAAA

CAGCACGTTCTCGGTCACGAAGCGGTCCTGCCGCACGACGGTGAACCCGAACCCGGGGTGGAGGCCCGTC

TTGAGCTGGTGATACAGGGCCACGGGGCTCATCTTGAAGTACCCCGCCATGAGCGCGTAGGTCAGCGCGT

TCTCCCCCGCCGCGCTCTCGCGGGCGTGCTGCACCACGGGCTGGCGGATGGAGGAGAAGTAGTTGGCCCC

CAGGGCCGGGGGGACCAGGGGGACGTCGCGCGCCAGGTCGCGCAGGGCCGGGGGGAAGTTGGGCGCGTTG

GCCACGTGGTCGGCGCCCGCAAACAGCGCGTGGACGGGCAGGACGTAGAAGTATTCGCCATTTTGGATGG

TGTGGTCCAGGTGCTGGGGGGCCATGAGCAGCACGCCGGCGTGCAGCGCCCCGTCGAAGATGCGCATGTT

GGCCGTCGACGCGGTGTTGGCGCCCGCGTCGGGCGCCGCGGAGCACAGCAGCGCCGTCGTGCGCTCGGCC

ATGTTGTGCGCCAGCACCTGCAGCGTGAGCATGGCGGGCCCGTCGACGACGACGCGCCCGTTGTGGAACA

TGCGCTTGACCGTGTTGGCCACCAGATTGGCGGGATGCAGCGGGTGGGCGGGGTCGGTCACGGGATCGCT

CGGGCACTCCTCACCGGGGGCGATCTCCGGGACCACCATGTTCTGCAGCGTGGCGTACACGCGGTCGAAG

CGGACCCCCGCGGTGCAGCAGCGCCCCCGCGAGAAGGCCGGCACCAGCACGTAATAGTAGATTTTGTGGT

GGACGGTCCAGTCGGCCGGCCGGTGCGGCCGGTCGTCGGCGGCGTCGGCCGCGCGGGCCTGGGTGTTGTG

CAGCAGCCGGCCGTCGTTGCGGTTAAAGTCGGCCGTCGCCACGTTGCACGCCGCCGCGTAGACGGGCTCG

TGCCCCCCCGCGTCAATCCGGCAGTCTCGGTGGCGGTCCAGGGCCGCGTGTCGCATAAGGCCGTCGCAGT

CCCACACGAGGGGCGGCAGCAGCGCCGGGTCGCGCATCAGGTGATTCAGCTCGGCCTGAGCCTGCCCGCC

CAGCTCCGGGCCCGGCAGGGTAAAGTCGTCCACCAGCTGGGCCAGGGCCTCGACGTGGGCCACCAGGTCC

CGATACACGGCCATGCACTCCTCGGGGAGGTCGCCCCCGAGGTAGGTCACGATGTACGAGACCAGCGAGT

AGTCGTTCACGAACGCCGCGCATCGCGTGTTGTTCCAGTAGCTGGTGATGCACTGAGTCACGAGCCGCGC

CAGGGCGCAGAACACGTGCTCGTTGCCGTGAATCGCGGCTTGCAGCAGGTAAAACACCGCCGGGTAGCTG

CGGTCCTCGAACGCCCCGCGGACGGCGGCTATGGTAGCCGGCGCCATGGCGTGGCGGCCAACGCCGAGCT

CCAGGCCCCGGGCGTCACGAAACGCCACCGGACACAGCGCCAGGGGCAGGTTGCCGTTGACCACGCGCCA

GGTGGCCTGGATCGCCCCCGGACCGGCCGGGGGGACTTCGCCGCCGGGAAGCTCGACGTCGGCCACGCCC

GCGAAGAAGTCGAACGCGGGGTGCAGCTCCAGAGCCAGGTTGGCGTTGTCGGGCTGCATGAACTGCTCCG

CGGTCATCTGGCACTCGGCGACCCACCGGACCCGGCCGTGGGCGAGGCGCTGCCGCCAGGCGTTCAGAAA

ACGCTGCTGCATGTCCGCGCCGGGGCCGGCCGGGGCCGCGACGTACGCCCCGTACGGATTCGCGGCCTCG

ACGGGGTCGTGGTTCACGCCCCCGACGGCCGCGTCGATGTTCATGAGCGAAGGATGACACACGGTCCCGA

CCGCGTTCTCCATGGACAGCCGCAGAACCTGGTGGTCCTTTCCCCAAAAAAACAGCTGCCGGGGAGGGAA

CGCGCGGGGCTCCGGGTGGCCGGGGGCGGGCACCAGGTCCCCGGCGTGCGCGGCGAAGCGCTCCATGGCC

GGGTTGAACAGCCCCAGGGGCAGGACGAACGTCAGGTCCATGGCGCCCACCAGGGGGTAGGGCACGTTGG

TGGCGGCGTAGATGCGTCTCTCCAGGGCCTCCAGGAAGACCAGCCTGTCGCCTATGGCCACCAGATCCGC

GCGCACGCGCGTTGTCTGGGGGGCGCTTTCGAGTTCATCCAGCGTCTCCCGGTTCGCCTCGAGTTGCTCC

TCCTGCATATCCAGCAGGTGGCGGCCCACGTCGTCCAGGCTCCGCACGGCCTTGCCCATCACCAGCGCCG

TGACGAGGTTGGCCCCGTTCAAGACCATCTCGCCGTAGGTCACCGGCACGTCGGCCTCGGTGTCCTCCAC

CTTCAGGAAGGACTGCAGGAGGCGCTGTTTGATGGCGGCGGTGGTGACCAGCACCCCGTCGACCGGCCGC

CCGCGCGTGTCGGCGTGCGTCAGGCGGGGCACGGCCACGGACGGCTGCGTCGCCGTGGTCAGGTCCACGA

GCCAGGCCTCGATGGCCTCGCGGCGATGGCCCGCCTTGCCCAGGAAGAAGCTCGTGTCGCAAAAGCTCCG

CTTCAGCTCGGCGACCAGGGTCGCCCGGGCAACCCTGGTCGCCAGGCGCCCGTTGTCGAGATATCGTTGC

ATGGGCAACAGCAGGGCCAGGGGAGGCGCCTTCTCCAACAGCACGTGCAGCATCTGGTCGGCCGTGCCGC

GCTCAAACGCCCCCAGGACGGCCTGGACGTTGCGCGCGAGCTGCTGGATGGCGCGCAGCTGGCGATGCAG

GCTAATGCCCGTCCCGTCCAGGGCCTCCCCCGTGAGCAGGGCAATGGCCTCGGTGGCCAGGCTGAAGGCG

GCGTTCAGGGCCCGGCGGTCGATGACCTTCGTCATGTAATTATGCACGGGCTGCTCGACGGGGTGCGGGC

CGTCGCGGGCGATGAGGGGCTGGTGGACCTCGAACTGCACACGCCCTTCGTTCATGTAAGCCAGCTCCGG

GAACTTGGTGCACACGCACGCCACGGACAGGCCGAGCTCCAGAAAGCGCACGAGCGACAGGGTGTTGCAG

TAGGACCCCAGCAGGGCGTCAAACTCTACGTCATACAGGCTGTTTTCGTCGGAGCGCACGGCGGCGAAAA

AATCAAAGAGTCTGCGGTGGGACGCCACCTCGATCGTACTCAGGATGGAGCCGGTGGGCAGGATGGCCGC

GGCGTACCGGTAACCCGGGGGGTCGCGGGCAGGAGCGGCCATTGGGTTCCTTGGGGGATTCGCAGGCTCC

ATCAAGCCGAGCTCGGGAAGGCCAAGCCCCTCCCGCACAACGCCTCACCGCCGGCGGACGCGACTAACAA

CCCACGGGCCGCCAAAACCCCAAGGGGCAACCCGACCAACAACAGGCGAGGGGAGGAAAGGCGTAAAGGG

GGCGTTGGGAGGCAAAAAGAAAGAAAACACCCAGACGTAGGCCCGAGGACCGGCCGGCGTCCTCTGTCCC

CGAGCACCCACTGTGCCCAACAGGCACGGGGGCGAGCTGCCCCTGCCTTATATACCCCCCCGCCACACCC

CCGTTAGAACGCGACGGGTGCCTTCAAGATGGCCCTGGTCCAAAAGCGTGCTAGAAAAAAGTTGGTAAAG

GCGGCAAAGCAGTCCGCCGCCGCCACCCACATGGCGGCGCCGGCCGCGCAGGCGATTCCCAGAGAACGGG

CGCGGAGGGGATCCGTGCGGGGCAGCAGCTGGCTGGCGGTGATCCAATGGAAAAGCCCGTCGGGACTGAA

CGTCTCATGGGCGGCCGCCACCAGGGCGCACAGGGCCGCGCCGCCCATGATCACGCACAACCCCCAAAAC

ACGGGTGGCGACAACGGCAGGCGATCCCGTTTGATGTTCACGTACAGGAGGAGCGCCCGTGCCAGCCACG

TGACATAGTAGGCGAGGACGGCGGCTATAATACATGCCGGCGCCACCGCCCGTCCGGTCCACCCGTAATA

CATGCCCGCGGCCACCAGCTCCAGCGGCTTGAGGACCAGGAACGACCAAGCAAACATCACCACCCGCTTG

GAAAAGACCGGCTGGGTGTGGGGCGGAAGACGCGAGTAGGCCGAACTGACAAAAAAATCAGACGTGCCGT

ACGAGGACAGCGAAAACTGTTCATCGAGCGGCAGTTCTCCGTCCTCCCCGCCACACGCGGCCTCGTCTAC

CAGCTCGCGATCCAACAAAGGAACATCATCCCGCATTGTCATGGTCGGTGCGGGGAGCCGGCGAGGCAGC

AAAACCGAAAGTAGTGCTGGCGGCGCGGGCCCGGGTCCGGACCCAAGCTTCAGGGATGGGGGGCGGAGGC

CAAAATCAAACAAGCACCGCGCGGGTTCTACACACAACCCCCACCCGGGTAGTATCCGCGGATGCGAGTG

CCTGGCGAAGTCACGTCCCAGCAGGATATAAACCTCGGCCGTTGGGCCCGGAACCCCCGAAATTCACACC

CACGCCCTGACGCCCAAATCATGGGTGGATGTGGTTCGCGAGCCGCACATCCGTGCGTCCGCCCTCCCCC

GCGGGCTGATGACGTGGCGGTTAGTCAGTGGGAAGGCAGGGGGAAAGATGGGTTGGGGGAGGAAACGAAG

AAAACACCCAGAGGGCCACGTCGGGAATGCGCCCGGAGTTGTCCTTAAAAGGCCGGCCGTGCGTGACGGA

AGCCGTCGTTTGCCCAAGCACCGACGCCGCGATCCACAGTGGGGGGAGTTCCTCCGTCCGGCCACAACCC

TACGCGCGGGCGGCACGCGCGAGAGCAACCCACGGGTCCCGTTCGCGCCACCGCCAGCCCTTGCTCCCAC

CACCCTCCTCCCACCACCCCACTATTCCCCCCCCCAAGTCCGCCCCGTGGCTCGCCGGCCATGGAGCTCA

GCTATGCCACCACCCTGCACCACCGGGACGTTGTGTTTTACGTCACGGCAGACAGAAACCGCGCCTACTT

TGTGTGCGGGGGGTCCGTTTATTCCGTAGGGCGGCCTCGGGATTCTCAGCCGGGGGAAATTGCCAAGTTT

GGCCTGGTGGTCCGGGGGACAGGCCCCAAAGACCGCATGGTCGCCAACTACGTACGAAGCGAGCTCCGCC

AGCGCGGCCTGCGGGACGTGCGGCCCGTGGGGGAGGACGAGGTGTTCCTGGACAGCGTGTGTCTGCTAAA

CCCGAACGTGAGCTCCGAGCGAGACGTGATTAATACCAACGACGTTGAAGTGCTGGACGAATGCCTGGCC

GAATACTGCACCTCGCTGCGAACCAGCCCGGGGGTGCTGGTGACCGGGGTGCGCGTGCGCGCGCGAGACA

GGGTCATCGAGCTATTTGAGCACCCGGCGATCGTCAACATTTCCTCGCGCTTCGCGTACACCCCCTCCCC

CTACGTATTCGCCCTGGCCCAGGCGCACCTCCCCCGGCTCCCGAGCTCGCTGGAGCCCCTGGTGAGCGGC

CTGTTTGACGGCATTCCCGCCCCGCGCCAGCCCCTGGACGCCCGCGACCGGCGCACGGATGTCGTGATCA

CGGGCACCCGCGCCCCCAGACCGATGGCCGGGACCGGGGCCGGGGGCGCGGGGGCCAAGCGGGCCACCGT

CAGCGAGTTCGTGCAAGTGAAGCACATCGACCGTGTTGTGTCCCCGAGCGTCTCTTCCGCCCCCCCGCCG

AGCGCCCCCGACGCGAGTCTGCCGCCCCCGGGGCTCCAGGAGGCCGCCCCGCCGGGCCCCCCGCTCAGGG

AGCTGTGGTGGGTGTTCTACGCCGGCGACCGGGCGCTGGAGGAGCCCCACGCCGAGTCGGGATTGACGCG

CGAGGAGGTCCGCGCCGTGCATGGGTTCCGGGAGCAGGCGTGGAAGCTGTTTGGGTCGGTGGGGGCTCCG

CGGGCGTTTCTCGGGGCCGCGCTGGCCCTGAGCCCGACCCAAAAGCTCGCCGTCTACTACTATCTCATCC

ACCGGGAGCGGCGCATGTCCCCCTTCCCCGCGCTCGTGCGGCTCGTCGGTCGGTACATCCAGCGCCACGG

CCTGTACGTTCCCGCGCCCGACGAACCGACGTTGGCCGATGCCATGAACGGGCTGTTCCGCGACGCGCTG

GCGGCCGGGACCGTGGCCGAGCAGCTCCTCATGTTCGACCTCCTCCCGCCCAAGGACGTGCCGGTGGGGA

GCGACGCGCGGGCCGACAGCGCCGCCCTGCTGCGCTTTGTGGACTCGCAACGCCTGACCCCGGGGGGGTC

CGTCTCGCCCGAGCACGTCATGTACCTCGGCGCGTTCCTGGGCGTGTTGTACGCCGGCCACGGACGCCTG

GCCGCGGCCACGCATACCGCGCGCCTGACGGGCGTGACGTCCCTGGTCCTGACCGTGGGGGACGTCGACC

GGATGTCCGCGTTTGACCGCGGGCCGGCGGGGGCGGCTGGCCGCACGCGAACCGCCGGGTACCTGGACGC

GCTGCTTACCGTTTGCCTGGCTCGCGCCCAGCACGGCCAGTCTGTGTGAGATATCCCAATAAAGTGCAGT

CGTTTTCTAACCCACGGATGCCGTTGTATGCCTATACGGGGGACTATGGGGGGGGAAAGGAAAGGAAACA

GGAATGGAGAAGGGAAAGGAACAGAGGCGGTAGCGGACGCACGGCGGACACAATAACAAACAGACCGCGG

ACACGGAGGGAGTCGGTTGGGTTGGGCGTGGACGCCGCTGCGTCCACACACCCGTTTATTCGCGTCTCCA

CAAAAATGGGACGCACGTTCGGACCACCCTAAGGATGCCCGCCAGGGCCGCGGTAATCATAACGACCCCC

AGCGCGGACGCGGCCAGAAACCCGGGGGCGATGGTGGCGATGGGCAGCGTGTCAAAGGCCAGCAGATGAA

TCACAGTTCCGTTGGGGAACAACAACAGGGCCACGGACGGCACGTCGCTGGAAAACACGTTCGGGGTGCC

CGCCACCGGCCCCTGGGCCAGCTGCTGTTGGGTGGCATCCGTGTCCACCAGCAGCACCGACATGACCTCC

CCGGCCGGGGTGTAGCGCAGAAACACGGCCCCCACGAGGCCGAGGTCGCGCCGGTTTTCGGTGCGCACCA

GCCGCTTCGGCTCAATCTCCCGCGCGTGCCCTTCGCAGGTGGCGGTGAGATAGGTGATAAACAGCGGGCG

GCGGACGTCAACGCCCGTAAGCTTGTATCCGATCCCGCGGGGCAAGGGGGTGTGGGTGACGACGTAGCTG

GCGTTGTGGGTGATGGGCACGAGGATCCGGGGCTCCGCGTTGTGCGACGGGCCGCTACACTGGTGGGTGG

CCTCCGGGACGAAGGCGCGGATCAGGGCGTTGTAGTGCGCCCAGCGCGTGAGAACGGAGGCCACGCCGCG

GGTCTGTTGTGCCATGACGTCCGCCGGGATGTCGGATCGGGTGGCCATGGCCAGCGCGTCCAGGATGAAC

CCGCCCTCGGCGAGATCGAAGCGCAGGGAAGCTGCGCATGGGGAAAAGTGGTCCGGGAGCCAGAAGAGGT

TTTTCTGGTGGTCGGTCCTGGCTAGCGCGGCCCGGAGATCGGCGTGGGTCGCCGCGGCGACGTCGGACGT

ACACAGGGCCGTGGTTATGAGGAGGCCCCGGCGGGCGCGTTCCCGCTGCTCGGCCGAGGGCGCGCCCGCC

AGGAACGGCGCCCGGAGGACGGCCGTGGCGTAAAACAGCGCTCGGCGGACCATCGGGGCGGTTAGCGCGC

GGCCGCCGAGAAACTCGGCGTACAGGGCGTCGATCAGGCGGGCCGCGCTCGGGGCCACCGCGCCATAGGC

CGCGGGGCTGTCCAACACGAACGCCAGCTGATAGCCCAGCGCGTGCGCCACCAGGCTCTGCTCTCGCTCG

AGGATCGCGGCCACCAGATGCCCGAGGCGCGCCTCCAGCCGCAGGCGGGCCGCCGGGTCCAACACGGACA

CGTTCAGGAACACCGAGTCGGCCGCGCAGCCCGCTGCTCCCCGGGCGGCCAGGCCGGCCAGCACGCGCGA

GTGGGCCAAAAAGCCCAGCAGGTCGGAGAGGCGAATCGCGTCGTGGGCGTGGGCCGCGTTGACGAACGCA

AACCCCGACGAGGCGAGCAGCCCCGCGAGGCGCCAGAACAGGGACGGACGCGCGTCCGTGCCGGAGCCCG

GGTCCTCCCCCAAAAACTCCGCATAGGCCCGCGACATATACTGGGCGTAGTTCGTGCTCTCCTCGGGGTA

GCCGGCCACCCGCCGGAGGGCGTCCAGCGCCGAGCCGTTGTCGGCGGGCGTCGGGGCCCCCAGGACAAAG

ACGCGATACCTGGGGCCGGCCGGAGGCCCGGGGAGCACCGCGGGGGCGTTTTCGTCGGTCGGATTTCCGA

CCCGAGCGAGGGTCTTGTCCGCAGGCACCACTATGATCTCGGCCGGAGGGCTGTCCCGCATCGATATCAC

GAGCCCCATGAAGCCCTTCCCGTATCGCGCGCGCACGAGCGCGGCGTCGCACCCGAACGCCAGCCCGCCC

GTCGTCCAGACGCCCACGGGCCACGTCGAGGCCGACGGGGAGAGGTACACGTACCGACCCGGAGTCCGTA

GCAGGCCCCTGGCGGCCAGCCAGGTCACGGATGCGTTGTGCAGATGCGCGATGCTCAGGTTCGTCGTCGG

ATGCCTCGGTGTCCCCGCGGGCGGCCCCGGGGGCGGCGCGTTGCGTCGGCCGTCCGGGTGCCTCTCGGTC

GCCCCGTCGTCTCCCCGCGGGAACGTAAGCCCCTCGCGGTCCGGCGCGGCCGCGAATGTTACCCAGGCCC

GGGACCGCAACAGCGCGGAGGCGCCGGGGTTGTGCGACAGTCCCTTGAGCTGGGTCACCTCGGCGGGGGG

ACGGGACGTGGGCCCCGCCTCGGGGAGCTCGGGCAGGCTCGCGTTCCGAGGCCGGCCGAGCAGATAGGTC

TTTGGGATGTAAAGCAGCTGCCCGGGGTCCCGAGGAAACTCGGCCGTGGTGACCAACACGAAACAAAAGC

GCTCGGCGTACCACCGAAGCATGGGCACGGATGCCGTAGTCAGGTTGAGTTCGCCCGGGGGCGCCAAGCG

TCCGCGCTGGGGGTCGCTGGCGTCGGGGGTGTTGGGCAACCACAGACGCCCGGTGTTTGTGTCGCGCCAG

TACGTGCGGGCCAACCCCAGACCGTGCAAAAACCACGGGTCGATTTGCTCCGTCCAGTACGTGTCATGGC

CCCCGGCAACGCCCACCAGGACCCCCATCACCACCCACAGACCGGGGCCCATGGTCGTCGTCCCGGCTGC

CAGTCCGCAGATGGGGGGGGGTGTCCGTACCCACGGCCCAAAGAGGCTCCGCACCTCGGAGGCTATCGGA

GGCCCTTTGTTGCCGTAAGCGCGGGCCAAAGGATGGGGTGGGGTGAGGGTAAAAGCACAAAGGGAGTACC

AGACCGAAAACAAGGACGGATCGGCCCGCTCCGTTTTTCGGTGGGGTGCTGATACGGTGCCAGCCCTGGC

CCCGAACCCCCGCGCTTATGGACACACCACACGACAACAATGCCTTTTATTCTGTTCTTTTATTGCCGTC

ATCGCCGGGAGGCCTTCCGTTCGGGCTTCCGTGTTTGAACTAAACTCCCCCCACCTCGCGGGCAAACGTG

CGCGCCAGGTCGCGTATCTCGGCGATGGACCCGGCGGTTGTGACGCGGGTTGGGATCATCCCGGCGGTGA

GGCGCAACAGGGCGTCTCGACACCCGACGGGCGACTGATCGTAATCCAGGACAAATAGATGCATCGGAAG

GAGGCGGTCGGCCAAGACGTCCAAGACCCAGGCAAAAATGTGGTACAAGTCCCCGTTGGGGGCCAGCAGC

TCGGGAACGCGGAACAGGGCAAACAGCGTGTCCTCGATGCGGGGCAGAGACCCCGCGCCGTCCTCGGGGT

CGGGGCGCGGGGTCGCCGCGGCGACCCCCGTCAGCCGGCCCCAGTCCTCCCGCCACCTCCCGCCGCGCTG

CAGGTACCGCACCGTGTTGGCGAGTAGATCGTAGACACGGCGAATGGCGGACAGCATGGCCAGGTCAAGC

CGCTCGCCCGGGCGTTGGCGTCTGGCCAGGCGGTCGGCGTGTTCGGCCTCCGGAAGGACACCCAGGACCA

GGTTCGTGCCGGGCGCGGTCGGGGGCATGAGGGCCACGAACGCCAACACGGCCTGGGGGGTCATGCTTCC

CATGAGGTACCGCGCGGCCGGGTAGCACAGCAGGGAGGCGATAGGGTGCCGGTCGAAAACAAGGGTGAGG

GCCGGGGGCGGGGCTTGCGGGCCCACAGCCTCCCCCCCGATATGAGGAGCCAAAACGGCGTCCGTCGCCG

CATAAGGCGTGCTCATTGTTATCTGGGCGCTGGTCATTACCACCGCCGCCTCCCCGGCCGATATCTCGCC

GCGGTCCAGACGGTGCTGCGTGTTGTAGATGTTCGTCAGGGTCTCGGAGGCCCCCAGCACCTGCCAGTAA

GTCATCGGCTCGGGGACGTAGACGATATTGTCGCGCGGCCCCAGGGCCTCCATCAGCTGCGCGGAGGTGG

TGGTCTTCCCCACCCCGTGGGGTCCGTCTATATAAACCCGCAGCAGCGTGGGCAGCTCCGGATCCCCGCG

GGCTCCGGAGGCCCCCTGGCGATGGCTAGGACGGGACGCCGCGCGGCCGTCGGTAGGCCCGCTCGCACGA

GCAGCCTGACCGAACGCAGGCGCGTGCTGTTGGCCGGCGTGAGAAGCCATACCCGCTTCTACAAGGCGTT

CGCCCGAGAGGTGCGGGAGTTCAACGCCACCAGGATTTGTGGAACGCTGCTGACGCTGATGAGCGGGTCG

CTGCAGGGTCGCTCGCTGTTCGAGGCCACGCGCGTCACCTTAATATGCGAAGTGGACCTCGGGCCGCGCC

GCCCAGACTGCATCTGCGTGTTCGAATTCGCCAATGACAAAACGTTGGGAGGTGTGTGCGTCATCCTGGA

GCTAAAGACATGCAAATCGATTTCTTCCGGGGACACGGCCAGCAAACGCGAACAGCGGACCACGGGCATG

AAGCAGCTGCGCCACTCCCTGAAGCTGCTGCAGTCGCTCGCGCCTCCGGGGGACAAGGTCGTCTACCTGT

GTCCTATTTTGGTGTTTGTCGCGCAGCGTACGCTGCGCGTCAGCCGCGTGACCCGGCTCGTCCCGCAAAA

GATCTCCGGCAACATCACCGCGGCCGTGCGGATGCTCCAAAGCCTGTCCACGTATGCCGTGCCGCCGGAA

CCGCAGACCCGGCGGTCGCGGCGCCGGGTCGCCGCGACCGCCAGACCGCAAAGGCCCCCCTCCCCGACAC

GTGACCCGGAAGGCACGGCGGGTCATCCGGCCCCACCAGAGAGCGACCCCCCCTCCCCAGGGGTCGTAGG

CGTCGCTGCGGAGGGTGGGGGTGTGCTTCAGAAAATCGCGGCGCTTTTTTGCGTGCCGGTGGCCGCCAAG

AGCAGACCCCGGACCAAAACCGAGTGAGGTTCTGTGTGTTGTTTTTTTTTTTTTTTTTCCTCGTTTTGTT

TTCTCTTCTTTCCCCCCCCCCTCCCCCGCTTCTGGCCAAGCATCCTCACCTGCTTAAGCGGAACCCGCGG

GCGCGCGGGGACTCATTTGTCGCCGGCGACACCCACCCGACAACAGCCCCTGGGTGTCGACCGCTGTCGC

CCCCGTCTGTCGCCTCTCCCTTTTTTCCCCCCCTCAAAGAACGTGGTGTTGGGCGCCGGCCAATTCTTCC

CGGAGCGCCGTCGTCGCCCGCCCGCCGCCCTCGAACATGGACCCGTACTACCCTTTCGACGCGCTGGACG

TTTGGGAACACAGGCGCTTCATCGTCGCCGACTCCAGGAGCTTCATCACCCCCGAGTTCCCCCGGGACTT

CTGGATGTTGCCCGTGTTCAACATCCCCCGGGAGACGGCGGCGGAGCGGGCGGCAGTGCTGCAGGCCCAG

CGCACCGCGGCCGCGGCGGCCCTGGAGAACGCCGCCCTCCAGGCCGCCGAGCTGCCCGTCGACATCGAGC

GCCGGATACGCCCGATCGAGCAGCAGGTGCATCACATCGCCGACGCCCTGGAGGCGCTGGAGACCGCGGC

GGCCGCGGCCGAAGAGGCGGATGCCGCGCGGGACGCCGAGGCGAGGGGGGAGGGCGCTGCGGACGGGGCA

GCGCCGTCGCCCACCGCGGGCCCCGCCGCCGCGGAGATGGAGGTTCAGATCGTACGCAACGACCCGCCGC

TACGATACGATACCAACCTCCCCGTGGATCTGCTACACATGGTGTACGCGGGCCGCGGGGCCGCGGGTTC

GTCGGGAGTCGTCTTTGGTACCTGGTACCGCACGATCCAGGAACGCACCATCGCGGACTTCCCCCTGACC

ACCCGCAGCGCCGACTTTCGAGACGGGCGCATGTCCAAGACCTTCATGACCGCGCTGGTCCTGTCTCTGC

AGTCGTGCGGCCGGCTGTACGTGGGCCAGCGCCACTATTCCGCCTTCGAGTGCGCCGTGCTGTGTCTGTA

TCTGCTGTACCGAACCACCCACGAGTCCTCCCCCGATCGCGATCGCGCTCCCGTTGCGTTCGGGGACCTG

CTGGCCCGCCTGCCGCGCTACCTGGCGCGTCTGGCCGCGGTAATCGGCGACGAGAGCGGACGCCCGCAGT

ACCGCTACCGCGACGACAAGCTGCCCAAAGCGCAGTTCGCGGCGGCCGGCGGCCGCTACGAGCACGGGGC

CCTGGCCACCCACGTCGTGATCGCCACGTTGGTGCGCCACGGGGTGCTACCGGCGGCCCCGGGCGACGTT

CCCCGAGACACCAGCACCCGCGTGAACCCCGACGACGTGGCCCACCGCGACGACGTCAACCGCGCCGCCG

CCGCGTTTTTGGCACGCGGCCACAACCTCTTCCTGTGGGAGGACCAGACGCTGCTGCGGGCGACCGCCAA

CACCATTACGGCCCTGGCCGTGCTTCGGCGGCTCCTCGCGAACGGCAACGTGTACGCGGACCGCCTCGAC

AACCGCCTGCAGCTGGGCATGCTGATCCCGGGAGCCGTCCCGGCGGAGGCCATCGCTCGGGGGGCGTCCG

GATTGGACTCGGGCGCCATAAAAAGCGGCGACAACAACCTGGAGGCGCTGTGCGTTAACTATGTACTTCC

GCTGTATCAGGCAGACCCCACGGTCGAGCTGACCCAGTTGTTTCCGGGGCTGGCCGCCCTGTGCCTGGAC

GCCCAGGCGGGGCGGCCACTGGCGTCGACGAGGCGCGTGGTGGATATGTCGTCGGGCGCCCGCCAGGCGG

CGCTCGTGCGCCTCACCGCGCTGGAGCTCATCAACCGCACCCGCACAAACACCACCCCTGTGGGGGAGAT

TATTAACGCCCACGATGCCTTGGGGATACAATACGAACAGGGGCCTGGGCTGCTCGCCCAGCAGGCACGC

ATCGGCTTGGCGTCAAACACCAAGCGATTCGCCACGTTCAACGTGGGCAGCGACTACGACCTGTTGTACT

TTTTGTGTCTCGGGTTCATTCCCCAGTACCTGTCCGTGGCCTAGGGAAGGGTGGGGGTGGTGGTGGTGGG

GTGTTTTTCTGTTGTTGTTTCTGGTCCGCCTGGTCACAAAAGGCACGGCGCCCCGAAACGCGGGCTTTAG

TCCCGGCCCGGACGTCGGCGGACACGCAACAACGGCGGGCCCCGTGGGTGGGTAAGTTGGTTCGGGGGCA

TCGCTGTATTCCCTTGCCCGCTTCCACCCCCCCCCCCCTTCCCGTTTTGTTTGTTTGTGCGGGTGCCCAT

GGCGTCGGCGGAAATGCGCGAGCGGTTGGAGGCGCCTCTGCCCGACCGGGCGGTGCCCATCTACGTGGCC

GGGTTTTTGGCCCTGTACGACAGCGGGGACCCGGGCGAGCTGGCCCTGGACCCAGACACGGTGCGTGCGG

CCCTGCCTCCGGAGAACCCCCTGCCGATCAACGTAGACCACCGCGCTCGGTGCGAGGTGGGCCGGGTGCT

CGCCGTGGTCAACGACCCTCGGGGGCCGTTTTTTGTGGGGCTGATCGCGTGCGTGCAGCTGGAGCGCGTC

CTCGAGACGGCCGCCAGCGCCGCTATTTTTGAGCGCCGCGGACCCGCGCTCTCCCGGGAGGAGCGTCTGC

TGTACCTGATCACCAACTACCTGCCATCGGTCTCGCTGTCCACAAAACGCCGGGGGGACGAGGTTCCGCC

CGACCGCACCCTGTTTGCGCACGTGGCCCTGTGCGCCATCGGGCGGCGCCTTGGAACCATCGTCACCTAC

GACACCAGCCTAGACGCGGCCATCGCTCCGTTTCGCCACCTGGACCCGGCGACGCGCGAGGGGGTGCGAC

GCGAGGCCGCCGAGGCCGAGCTCGCGCTGGCCGGGCGCACCTGGGCCCCCGGCGTGGAGGCGCTCACACA

CACGCTGCTCTCCACCGCCGTCAACAACATGATGCTGCGTGACCGCTGGAGCCTTGTGGCCGAGCGGCGG

CGGCAGGCCGGGATCGCCGGACACACGTACCTTCAGGCGAGCGAAAAATTTAAAATATGGGGGGCGGAGT

CTGCCCCTGCGCCGGAGCGCGGGTATAAAACCGGCGCCCCGGGTGCCATGGACACATCCCCCGCCGCGAG

CGTTCCCGCGCCGCAGGTCGCCGTCCGTGCGCGTCAAGTCGCGTCGTCGTCTTCTTCTTCTTCTTTTCCG

GCACCGGCCGATATGAACCCCGTTTCGGCATCGGGCGCCCCGGCCCCTCCGCCGCCCGGCGACGGGAGTT

ATTTGTGGATCCCCGCCTCTCATTACAATCAGCTCGTCACCGGGCAATCCGCGCCCCGCCACCCGCCGCT

GACCGCGTGCGGCCTGCCGGCCGCGGGGACGGTGGCCTACGGACACCCCGGCGCCGGCCCGTCCCCGCAC

TACCCGCCTCCTCCCGCCCACCCGTACCCGGGTATGCTGTTCGCGGGCCCCAGTCCCCTGGAGGCCCAGA

TCGCCGCGCTGGTGGGGGCCATCGCCGCCGACCGCCAGGCGGGTGGGCTTCCGGCGGCCGCCGGAGACCA

CGGGATCCGGGGGTCGGCGAAGCGCCGCCGACACGAGGTGGAGCAGCCGGAGTACGACTGCGGCCGTGAC

GAGCCGGACCGGGACTTCCCGTATTACCCGGGCGAGGCCCGCCCCGAGCCGCGCCCGGTCGACTCCCGGC

GCGCCGCGCGCCAGGCTTCCGGGCCCCACGAAACCATCACGGCGCTGGTGGGGGCGGTGACGTCCCTGCA

GCAGGAACTGGCGCACATGCGCGCGCGTACCCACGCCCCCTACGGGCCGTATCCGCCGGTGGGGCCCTAC

CACCACCCCCACGCAGACACGGAGACCCCCGCCCAACCACCCCGCTACCCCGCCAAGGCCGTCTATCTGC

CGCCGCCGCACATCGCCCCCCCGGGGCCTCCTCTATCCGGGGCGGTCCCCCCACCCTCGTATCCCCCAGT

TGCGGTTACCCCCGGTCCCGCTCCCCCGCTACATCAGCCCTCCCCCGCACACGCCCACCCCCCTCCGCCG

CCGCCGGGACCCACGCCTCCCCCCGCCGCGAGCTTACCCCAACCCGAGGCGCCCGGCGCGGAGGCCGGCG

CCTTAGTTAACGCCAGCAGCGCGGCCCACGTGAACGTGGACACGGCCCGGGCCGCCGATCTGTTTGTGTC

ACAGATGATGGGGTCCCGCTAACTCGCCTCCAGGATCCGGACTTGGGGGGGGTGTGTGTTTTCATATATT

TTAAATAAACAAACAACCGGACAAAAGTATACCCACTTCGTGTGCTTGTGTTTTTGTTTGAGAGGGGGGG

GTGGAGTGGGGGGGAAAGTGGGCCGAATGACACAAAAATTAGGTCGGAGGGGTGAGGGGGGGGGGCTAGG

AGCCGAACCGATGGCCCCCACACGCGACGGAAGGCCCGGAAGACTACCACGGGGAGGGGGTGTGGAAAGC

GACCGGTCGCAGGGAGACGGGGTTGGTTTGGGGTTGGTTTGGGGTTGGTTTTCCCGTTAGCACATGTCTG

CATTTGTTTTTCTAGTCACACGCCCCCCCCCCCCCAAATAAAAACCAAGGCAAAACAATACCAGAAGTCA

TGTGTATTTTTGAACATCGGTGTCTTTTTATTTATACACAAGCCCAGCTCCCCTCCCCTCCCTTAGAGCT

CGTCTTCGTCTCCGGCCTCGTCCTCGTTGTGGAGCGGAGAGTACCTGGCTTTGTTGCGCTTGCGCAGAAC

CATGTTGGTGACCTTGGAGCTGAGCAGGGCGCTCGTGCCCTTCTTTCTGGCCTTGTGTTCCGTGCGCTCC

ATGGCCGACACCAAAGCCATATATCGGATCATTTCTCGGGCCTCGGCCAACTTGGCCTCGTCAAACCCGC

CCCCCTCCGCGCCTTCCTCCCCCTCCCCGCCCACGCCCCCGGGGTCGGAAGTCTTGAGTTCCTTGGTGGT

GAGCGGATACAGGGCCTTCATGGGATTGCGTTGCAGTTGCAGGACGTAGCGGAAGGCGAAGAAGGCCGCG

ACCAGGCCGGCCAGGACCAGCAGCCCCACGGCAAGCGCCCCGAAGGGGTTGGACATAAAGGAGGACACGC

CCGAGACGGCCGACACCACGCCCCCCACTACTCCCATGACTACCTTGCCGACCGCGCGCCCCAAGTCCCC

CATCCCCTCGAAGAACGCGCACAGCCCCGCGAACATGGCGGCGTTGGCGTCGGCGCGGATGACCGTGTCG

ATGTCGGCAAAGCGCAGGTCGTGCAGCTGGTTGCGGCGCTGGACCTCCGTGTAGTCCAGCAGGCCGCTGT

CCTTGATCTCGTGGCGCGTGTAGACCTCCAGGGGCACAAACTCGTGGTCCTCCAGCATGGTGATGTTCAG

GTCGATGAAGGTGCTGACGGTGGTGACGTCGGCGCGACTCAGCTGGTGAGAGTACGCGTACTCCTCGAAG

TACACGTAGCCCCCGCCGAAGATGAAGTAGCGCCGGTGGCCCACGGTGCACGGCTCGAGCGCGTCGCGGG

TGAGGCGCAGCTCGTTGTTCTCGCCCAGCTGCCCCTCGATCAGCGGGCCCTGGTCTTCGTACCGAAAGCT

GACCAGGGGGCGGCTGTAGCACGTCCCCGGCCGCGAGCTGACGCGCATCGAGTTCTGCACGATCACGTTG

TCCGGGGCGACGGGCACGCACGTGGAGACGGCCATGACGTCTCCGAGCATGCGCGCGCTCACCCGCCGGC

CGACGGTGGCGGAGGCGATGGCGTTGGGGTTGAGCTTGCGGGCCTCGTTCCAGAGAGTCAGCTCGTGGTT

CTGCAGCTCGCACCACGCGACGGCGATGCGCCCCAGCATGTCATTCACGTGGCGCTGTATGTGGTTATAC

GTAAACTGCAGCCGGGCGAACTCGATCGAGGAGGTGGTCTTGATGCGCTCCACGGACGCGTTGGCGCTGG

GCGCCTCCCGCAGTGGCGCGGGCGTGGCATTCCGGGGCTTGCGGTCCTGCTCCCGCATGTACTCCCGCAC

GTACAGCTCGGCGAGCGTGTTGCTGAGGAGGGGCTGGTACGCGATGAGGAAGCCCCCCGTGGCCAGGTAG

TACTGCGGCTGGCCCACCTTGATGTGCGTGGCGTTGTACTTGCGCGCAAACATGCGGTCGATGGCCTCGC

GGGCATCCCGGCCGATGCAGTCGCCCAGGTCGACGCGCGAGAGCGAGTACTCGGTCAGGTTGGTGGTGAA

GGTGGTCGAGATGGCGTCGGAGGAGAAGCGGAAGGAGCCGCCGTACTCGGCGCGGAGCATCTCGTCCACC

TCCTGCCACTTGGTCATGGTGCAGACCGCCGGTCGCTTCGGCACCCAGTCCCAGGCCACGGTAAACTTGG

GGGTCGTCAGCAAGTTGCGGGTCGTCGGCGACGTGGCCCGGGCCTTCGTGGTGAGGTCGCGCGCGTAGAA

GCCGTCGACCTGCTTGAAGCGGTCGGCGGCGTAGCTGGTGTGCTCGGTGTGCGACCCCTCCCGGTAGCCG

TAAAACGGGGACATGTACACAAAGTCGCCCGTCGCCAGCACAAACTCATCGTACGGGTACACCGACCGCG

CGTCCACCTCCTCGACGATGCAGTTGACCGTCGTGCCGTACCGATGGAACGCCTCCACCCGCGAGGGGTT

GTACTTGAGGTCGGTGGTGTGCCACCCCCGGCTCGTGCGCGTGGCGACCTTCGCCGGCTTGAGCTCCATG

TCGGTCTCGTGGTCGTCCCGGTGAAACGCGGTGGTCTCCATGTTGTTCCGCACGTACTTGGCCGTGGAGC

GGCAGACCCCCTTGGTGTTAATCTTGTCGATCACCTCCTCGAAGGGAACGGGGGCGCGGTCCTCGAATAT

CCCCATAAACTGGGAGTAGCGGTGGCCGAACCACACCTGCGACACGGTCACGTCTTTGTAGTACATGGTG

GCCTTGAATTTGTACGGGGCGATGTTCTCCTTGAAGACCACCGCGATGCCCTCCGTGTAGTTCTGCCCCT

CCGGGCGCGTCGGGCAGCGGCGCGGCTGCTCAAACTGCACCACCGTGGCGCCCGTCGGGGGCGGGCACAC

GTAAAACTGGGCATCGGCGTTCTCGACCTTGATTTCCCGCAGGTGCGCGCGCAGCGTGGCGTGGCCGGCG

GCGACGGTCGCGTTGGCGTCGGGGGGCGGGGTCGCCTCGGGCCGCTTGGGCGGCTTTTTGGTTTTCCGCT

TCCGGGCCTTGGTGGTCGCGGGGCTCGGGACGGGGGGCGGCCGGGAGGCGGGACCCCCGTTCGCCGCGAC

GGTCGCGGCCACGCCGCCCGAGGCGCGGGGGGCCGCCGGGGCCGCCGGGGCCGCCGACGCCACCGCGGCC

ACCAGCGCCCCCACGACCAGCGCGCAAATCAAGCCCCCCCCGCGCATGGCGGGCCTACGGGGGCGCGTCG

CTCCCGCCGCCCGCTAGTCTGGGGGCGAGGTGCTGCAGGACCGAGTAGAGGATGGAAAAAACGTCTCGGT

CGTAAACCACGACCGAGCGGGGTCCGATGCAGCCGTCGGGGCCGCTCTCGACGATGGCCACCAGCGGACA

GTCGGAGTTGTACGTGAGGTACACGCCCGGCGGGTAGCGGTACAGACCTTCGGAGGTCGGGCGGCTGCAG

TCGGGGCGGCGCAACTCAAGCTCCCCGCACCGGTAGACCGACGCAAAGAGTGTGGTGGCGATAATGAGCT

CGCGAATATATCGCCAGGCGGCGCGCTGGGTGGGCGTGATTCCGGAAACACCGTCAAAACAGTAGAACTT

TTGAAACTCGCTGACGGCCCAATCAGCGCCCGAACCCCCCGCGCCCATGATGAAGCGGGCGAGTTCCTCC

TTGAGGTGCGGCAGGAGCCCCACGTTCTCGACGCTGTAGTACAGCGCGGTGTTGGGGGGCTGGGCGAAGC

TGTGGGTGGAGTGGTCGAACAGGGGCCCGTTGACGAGCTCGAAGAAGCGATGGGTGATGCTGGGGAGCAG

GGCCGGGTCCACCTGGTGGCGCAGCAGCGACGCTCGCATGAACCGGTGCGCGTCAAACACGCCCGGGGCG

GCGCGGTTGTCGATGACCGTGCCCGCGCCCGCCGTCAGGGCGCAGAAGCGCGCGCGCGCCGCGAAGCCGT

TGGCGACCGCGGCGAAGGTCGCGGGCAGCACCTCGCCGTGGACGCTGACCCGCAGCATCTTCTCGAGCTC

CCCGCGCTGCTCGCGCACGCAGCGCCCGAGGCTGGCCAGCGACCGCTTGGTCAGGCGGTCCGCGTACAGC

CGCCGGCGCTCCCGCACGTCCGCGGCGGCCCGCGTCGCGATGTCGCCCCAGCTCTCCGGCCCCTGCGCCC

CTGGCTCGGGGCCGCGCTCCCCGTCCTCGCTCGCGGGCGTCCCCGCGCCACGCCTCCGCCCCCCCTCCTC

CGCGGCGGCCCGGGGCTCTTCCTCCTCGGCCCCCCCGGTCGCGCCGCCGGCCCCCAGCCGCGCCAGCACG

CGGCGCAGCGCCTCCTCGTCGCACTGCTCGGGGCTGACGAGCCGCCGCAGCAGCGGCGTCGTCAGGTGGT

GGTCGTAGCACGCGCGTATCAGCGCCTCGATCTGATCGTCGGGCGACGTCGCCTGGCCGCCGATGATCAG

GGCGTCCACCATGTCCAGCGCCGCCAGGTGGCCCCCGAACGCGCGATCGAAGTGCTCCGCCCGCCGCCCG

AACAGCGCCAGCTCCACGGCCACCGCGGCGGTCTCCTGCTGCAGCTCGCGCTGCGCCAGCGCGTTCAGGT

TGTCGGCGAAGGCGTCCATGGTGGAGTGGCGGGCGCGATCGCCGGACGCCAGCCAGAAGCGCAGCTCGCT

GATGGCGTACAGGCCGGGCGTAGTGGCCTGAAACACGTCATGCGCCTCCAGCAGGGCGTCGGCCTCCTCG

CGGACAGAAGAGCTATCGGCGGGCGGCGGGCCGGCCCTGGCCCCGCCGCCCGCCGCGGTCCGCGCCAGCG

CCTGGTCCAGCACACAGAGCGCTCGCGCGCGGGCGGCGTCCGACAGCCCGGCGGCGTGGGGCAGGTACCG

TCGCAGCTCGTTGGCGTCCAGCCGCACCTGGGCCTGTTGGGTGACGTGGTTACAGATGCGGTCCGCCAGG

CGGCGGGCGATGGTCGCCCCTTGGTTCGCGGTGACGCACAGCTCCTCGAAACAGACCGCGCACGGGTGGG

ACGGGTCGCTCAGCTCCGGGGGCACGATGAGGCCCGACCCCACCGCCGCCACCATAAACTCCCGGACGCG

CTCCAGCGCGGCCGTGGCGCCGCTCGGGGGGGTGATGAGGTGGCAGTAGTTCAGCTGCTTGAGAAAATTC

TCGACATCATGCAGGAAGCACAGCTCCATGCGGACGTCCCCGCCGTACGTCTGCAGCCGGATCTGCTGGT

GGTACGGACAGGGTCGGGCCAGACCCATGGTCTCGGTGAAAAAGGCAGAGACGTCACCCGTGGTCGCGAA

CGTTTCCAGGTGGCCCAGGAGCCGCTCCCCCTCGCGCCACGCGTACTCCAGGAGCAACTCCAGGGTGACC

GACAGCGGGGTGAGAAAGGCGGCGGCCTGAGCCTCCAGCCCCGGCCGCAGGTGCCGCCGCAGCACGCGCA

CCTGGAGCGCGTTGAGTTTTAGCTGGGCGAGCTTCCCCAGGCCGATCTGGGGGTCGCATCGTCGAAGCAG

CTCTAGCTGAAAAACGTACGTCTGTACCTGCCCGAGCAGGGCCAACAGTTTCTGTCGGGCCGCAGTGGGC

TCGGAAACCGCGGCCGGGGGCGCGGCCGCCATGGCGAGTCACCCGGCCGTGCTGTGGTTTAGTTAAGGTT

TGGGGGGGGGTGGGTCAGAGGCGCGCCCCGCGCGGACTGATGCGGCGGCGGGCCCCTGACATCCCCTCTT

TATGCCCGTCGCCCGCCCGCCCGCCCCGCCGGTGTGCCGTGATTCGCGGAGTCGGGGCCTTGTGTTTCTT

TCTTTCCCCCCCGAATCCGTTCTTTCTTCCTCACCCCCCCCTCCCCACACACCCACCCAGGACTCGCCAC

CACAAGGAGGCGAGAGCCCGTCGCTAACCCAAAGACACAGTCACGAGACACGATATCGACTGTAGTTGCG

ATCGTTTATTTTATACACAACACCAACCTTTCCTTCGACCCCCCCCACCCCCGCCCCTAGAGCATATCCA

ACGTCAGGTCCTTTTTCTCCGGTGGTCCCTCCCCAAACGGATCGTCGCCGTGAAACGCCCGCTTTCGGGC

GACGCCGGCCGCCCCCGCCGCCGCCGCCAAACCGCCGAACGACGCCGCGTGGTCATCCTCGTCGCCGAAA

TCCCCAAAGTTAAACACCTCCCCGGCGGCGCCGAGCTGGCTGACCAGGGCCTCCGCCTCGTGGGCCACCT

CCAGGGCCGCGTCGGTCGACCACTCGCCATGCCCGCGCTCCAGGGCGCGGGTGGTAAACTCCATCATTTC

CTCGCTCAGGTACTCGTCCTCCAGCAGCGCCAGCCAGTCCTCGATCTGCAGCTGCTGGGTGCGGGGGCCC

AGGCTCTTGACGGTCGCCACAAACACGCTGCTGGCGACCGCCGCCCCGCCCTCCGCAATGATGCCCCGGA

GCTGCTCGCACAGCGAATGCTCGTGGGCCCCGCCCCCGAGACTCGACGCCGCGCACACAAACCCGGCCCT

GGGGCAGGCCAGGACAAACTTGCGGGTGCGGTCAAAGATCAGCAGCGGGCACGCGTTTTTGCCGCCCAGC

AGGCTGGCCCAGTTCCCGGCCTGAAACACGCGGTCGTTGCCGGCCATGCCGTAGTATTTGCTGATGCTGA

GGCCCAGCACGACCATCGGGCGCGCGGCCATCACGGGCCGCAGCAGGTTGCAGCTCGCGAACATGGACGT

CCAGGCGCCGGGGTGCGCGTCGAGGGAGTCCATCAGCGCGCGGGCCCCGGCCTCCAGGCCCGCGCCGCCC

TGCGGGGCCCAGGCGGCCGCCGCCTGCACGCTGGGGGGACGGCGGGACCCGGCGATGACGGCCGTGAGGG

TGTTTATGAAGTACGTCGAGTGGTCGCAGTACCTCAAGATCTGGTTGGCCATGTAGTACATGGCCAGTTC

GCTCACGTTATTGGGGGCCAGGTTGATAAAGTTAATCGCGCCGTAGTCCAGGGAGAACCTCTTAATGAAC

GCGATGGTCTCTATGTCCTCGCGCGACAAGAGCCGGGCGGGGAGCTGGTTGCGCTGGAGGGCGGTCCAGA

ACCACTGCGGGTTCGGCTGGTTCGACCCCGGGGGCTTGCCGTTGGGAAAGATGACCGCGTGGAACTGCTT

CAGCAGGAAGCCCAGCGGTCCGAGGAGGATGTCCACGCGCTTGTCGGGCTTCTGGTAGGCGCTCTGGAGG

CTGGCGACCCGCGCCTTGGCGGCCTCGGACGCGTTGGCGCTCGCGCCCGCGAACAACACGCGGCTCTTGA

CGCGCAGTTCCTTGGGAAACCCAAGGGTCACGCGGGCAACGTCGCCCTCGAAGCTGCTCTCGGCGGGGGC

CGTCTGGCCGGCCGTTAGGCTGGGGGCGCAGATAGCCGCCCCCTCCGAGAGCGCGACCGTCAGCGTCTTC

GCCGACAGGAACCCGTTGTTGAACAGGTCCATGACGCGCCGCCGCAGCACCGGTTGGAATTGATTGCGAA

AGTTGCGCCCCTCGACCGACTGCCCGGCGAACACCCCGTGGCACTGGCTCAGGGCCAGGTCCTGGTACAC

GGCGAGGTTGGACCGCCGCGCGAGGAGCTGCAGCAGGGGGCACGGCCCGCAGGTGTACGGGTCCAGCGAC

AGCGACATGGCGTGGTTGGCCTCGGCCAGACCGTCGCGGAACTTAAAGTTGCGCCCCTCGATCAGGTTGC

GCATCAGCTGTTCCACCTCGCGATCCACCAGCTGCTTGATGTTGTTCACCACCGTGTGCAGGGCCTCGCG

GTTGCCGATAATCGTCTCCAGCCTCCCCAGGGCCGTGGGCACCGCCTGGTCCACGTACTGCAGGGCCTCG

AGCTCGGCCATGACGCGCTCGGTGGCCGCGCGGTACGTCTCCTGCATGATGGTCCGGGTGTTCTCGGACC

CGTCCGCGCGCTTCAGGGCCGAGAAGGCGGCGTAGTTCCCCAGCACGTCGCAGTCGCTGTACGCGCTGTT

CATCGTTCCGAAGACCCCAATGGCCCCCCGGGCGGCGCTCGCGAACTTGGGGTGGCGGGCCCGCAGCCGC

ATCAGCGTCGTGTGCGCGCAGGCGTGGCGGGTCTCGAAGGTACACAGGTTGCAGGGCACGTCGGTCTGGC

CCGAGTCCGCGACGTAGCGAAACACGTCCATCTCCTGGCGCCCGACGATGACTCCGCCGTCGCAGCGCTC

CAGGTAAAACAGCATCTTGGCCAGCAGGGCCGGAGAGAACCCGCACAGCATGGCCAGGTGCTCGCCGGCG

AACTCCTGGGTTCCGCCGACGAGGGGCGCCGTGGGGCGCCCCTCGTACCCGGGCACCACGTGGCCCTCGC

GGTCCAGCTGCGGGTTGGCCGCCACGTGCGTGCCGGGCACGAGAAAGAAGCGGTAAAAGGAGGGCTTGCT

GTGGTCCTTGGGGTCCGCCGGCCCGGCGTCGTCCACCTCGGTCAGGTGGAGGGCCGAATTGGTGCTGAAC

ACCATGGCGCCCACGAGGCCCGCGGCGCGCGCCAGGTACGCCCCGACGGCGCCGGCGCGGGCCGCGGGCG

TTTCCTGGCCCTCAAGCAGGGGCCACGTGGTGATGTCGGGGGGCGGCTCGTCAAAGACCGCCATCGACAC

GATGGACTCCAGGGCCAGGGCGGCGTCGCCCGCCATCACCGAGGCCAGGCGCTGCTCAAACCCGCCCGCC

GGGCCCTTGTTCCCGGCGTCGCGCGCGCCCCGCTGGGGCTTACCCTGGCTGGCCTCGAAGGCCGTGAACG

TAATGTCGGCGGGGAGGGCCGCGCCCTCGTGGTTTTCGTCGAACGCCAGGTGGGCGGCCGCGCGGGCCAC

GGCGTCCACGTTCCGGGCACGCAGGGCCACGGCGGCGGGCCCGACGACCGCCTCGAACAGCAGGCGGGCG

AGGGGGCGGTTGAAAAACGGAAGGGGGTAGTTGAAATTCTCCCCGATCGATCGGTGGTTGCAGTTAAACG

GATCGGCGATGACCCGGCTAAAATCCGGCATAAACATCTGCAGCGGATACACGGGGATGCGGTGAACCTC

CGCGTCCCCGATGGTTACCTTGTCCATCCCGCCCAGATGCAGGAAGGTGTTGCTGATGCACACGGCCTCC

CGGAAGCCCTCCGTGATCACCAGATACAGCAAGGCCCGGTCCGGGTCCAGTCCGAGCCGCTCGCACAGCG

CGTCCCCCGTCGTCTCGTGCTTTAGGTCGCAGGGCCGGGGCGCGTAGTCCGCGAAGCCAAAATGCGGGCG

CGCCCGCTCGCAGAGCCGCGTCAGGTTGGGGGCCTGGGTGCTGGGGGCCAGGTGGCGGCCGCCGTGAAAG

ACGTAAACGGACGGGCTGTAGTGCGAGGGCATAAGCTTGAGGGACACCGCGGTCCCCCCAAGGCCCGTCG

TGCGGGACCCGACGACCGCGGCCACGTTGGCCTCAAACCCGCTCTCCACGGTCAGGCCGACGATGAGGGG

CGCGACGGCGACGTCCGCGTCGCCGCTGCGCGCCGACAGTAGCGACAGCAGCTCCAGGCCTTCGGCCGGA

CAGGCGCGGCCATACACGTACCCCATCGGCCCCGGAGGAACCTTGACGGTGGTCGTCGTTTTGGGCTTGG

TGTCCATGGCTTTCGGGAGATCGGCGACCGGCAGGAACGGGGGCCCGGCAAGACGACCGGGGGCAGACGG

GGGAGGCCGCGCGTGGTCGACGGCTGCTGCCCGCCGTCGTCTCTCCGATGGGGTCGAATGCCGGCGCTGG

GGGTGGGGTCTACACCCGCCCGTTCGCCGAGCGGCCCCTGGTGGGGGTGGGATGGGTGGGATGGGGTGGG

CGAGAATGGCCCGCCACCGGATCGCGCCGGACGGGGGGGCCCGGGGTTGGGCAAGGTTTGGGCGCAAGGC

TCCAGCGGCGATTCGAGAGGCCTGCGGATGGCGGCCCAGAGCTGGGTATGCTCGGCCGGGGCGGCCGGTA

TATGTACGGCGTGCTGGGAGGGGCGGCGTCGGGCCCCGCCCACGGTCCGCCACGCCCCGCGCGTCATCGG

CAGGGGGCGTGGCCGCCCTTCTAAAAAAAGTGAGAACGCGAAGCGTTCGCACTTTGTCCTAATAATATAT

ATACTATTAGGACAAAGTGCGAACGCTTCGCGTTCTCACTTTTTTTAGAAGGGCGGCCACGCCCCCTTTG

ACGTCACGCTCACCCGGGCGGCCGGCCGCCCATAAGCGCGGCCTGCCGGGCCGATAAAAAGAAACCGCGG

CGCCCCCGCGGACACCACACACTGGCTCTCGAACCCCGGACGCGCAGAAGGGACCCGGGCGCGGGTCCGC

CGGTAAGAGCCGGGGGGAACATCGGCACCGCCATCCCACCCCGAGCTGTTGGGTGGGCGGGTGGGGGGGC

TGGTGAGGCGGTGGTGGGAGGGGGCGGCGTATAGCAGGACAACGACCGGCGGCGATGTTTTGTGCCGCGG

GCGGCCCGGCTTCCCCCGGGGGGAAGCCGGCGGCTCGGGCGGCGTCTGGGTTTTTTGCCCCCCACAACCC

CCGGGGAGCCACCCAGACGGCACCGCCGCCTTGCCGCCGGCAGAACTTCTACAACCCCCACCTCGCTCAG

ACCGGAACGCAGCCAAAGGCCCTCGGGCCGGCTCAGCGCCATACGTACTACAGCGAGTGCGACGAATTTC

GATTTATCGCCCCGCGTTCGCTGGACGAGGACGCCCCCGCGGAGCAGCGCACCGGGGTCCACGACGGCCG

CCTCCGGCGCGCCCCTAAGGTGTACTGCGGGGGGGACGAGCGCGACGTCCTCCGCGTGGGCCCGGAGGGC

TTCTGGCCGCGTCGCTTGCGCCTGTGGGGCGGTGCGGACCATGCCCCCGAGGGGTTCGACCCCACCGTCA

CCGTCTTCCACGTGTACGACATCCTGGAGCACGTGGAACACGCGTACAGCATGCGCGCCGCCCAGCTCCA

CGAGCGATTTATGGACGCCATCACGCCCGCCGGGACCGTCATCACGCTTCTGGGTCTGACCCCCGAAGGC

CATCGCGTCGCCGTTCACGTCTACGGCACGCGGCAGTACTTTTACATGAACAAGGCGGAGGTGGATCGGC

ACCTGCAGTGCCGTGCCCCGCGCGATCTCTGCGAGCGCCTGGCGGCGGCCCTGCGCGAGTCGCCGGGGGC

GTCGTTCCGCGGCATCTCCGCGGACCACTTCGAGGCGGAGGTGGTGGAGCGCGCCGACGTGTACTATTAC

GAAACGCGCCCGACCCTGTACTACCGCGTCTTCGTGCGAAGCGGGCGCGCGCTGGCCTACCTGTGCGACA

ACTTTTGCCCCGCGATCAGGAAGTACGAGGGGGGCGTCGACGCCACCACCCGGTTTATCCTGGACAACCC

GGGGTTTGTCACCTTCGGCTGGTACCGCCTCAAGCCCGGCCGCGGGAACGCGCCGGCCCAACCGCGCCCC

CCGACGGCGTTCGGAACCTCGAGCGACGTCGAGTTTAACTGCACGGCGGACAACCTGGCCGTCGAGGGGG

CCATGTGTGACCTGCCGGCCTACAAGCTCATGTGCTTCGATATCGAATGCAAGGCCGGGGGGGAGGACGA

GCTGGCCTTTCCGGTCGCGGAACGCCCGGAAGACCTCGTCATCCAGATCTCCTGTCTGCTCTACGACCTG

TCCACCACCGCCCTCGAGCACATCCTCCTGTTTTCGCTCGGATCCTGCGACCTCCCCGAGTCCCACCTCA

GCGATCTCGCCTCCAGGGGCCTGCCGGCCCCCGTCGTCCTGGAGTTTGACAGCGAATTCGAGATGCTGCT

GGCCTTCATGACCTTCGTCAAGCAGTACGGCCCCGAGTTCGTGACCGGGTACAACATCATCAACTTCGAC

TGGCCCTTCGTCCTGACCAAGCTGACGGAGATCTACAAGGTCCCGCTCGACGGGTACGGGCGCATGAACG

GCCGGGGTGTGTTCCGCGTGTGGGACATCGGCCAGAGCCACTTTCAGAAGCGCAGCAAGATCAAGGTGAA

CGGGATGGTGAACATCGACATGTACGGCATCATCACCGACAAGGTCAAACTCTCCAGCTACAAGCTGAAC

GCCGTCGCCGAGGCCGTCTTGAAGGACAAGAAGAAGGATCTGAGCTACCGCGACATCCCCGCCTACTACG

CCTCCGGGCCCGCGCAGCGCGGGGTGATCGGCGAGTATTGTGTGCAGGACTCGCTGCTGGTCGGGCAGCT

GTTCTTCAAGTTTCTGCCGCACCTGGAGCTTTCCGCCGTCGCGCGCCTGGCGGGCATCAACATCACCCGC

ACCATCTACGACGGCCAGCAGATCCGCGTCTTCACGTGCCTCCTGCGCCTTGCGGGCCAGAAGGGCTTCA

TCCTGCCGGACACCCAGGGGCGGTTTCGGGGCCTCGACAAGGAGGCGCCCAAGCGCCCGGCCGTGCCTCG

GGGGGAAGGGGAGCGGCCGGGGGACGGGAACGGGGACGAGGATAAGGACGACGACGAGGACGGGGACGAG

GACGGGGACGAGCGCGAGGAGGTCGCGCGCGAGACCGGGGGCCGGCACGTTGGGTACCAGGGGGCCCGGG

TCCTCGACCCCACCTCCGGGTTTCACGTCGACCCCGTGGTGGTGTTTGACTTTGCCAGCCTGTACCCCAG

CATCATCCAGGCCCACAACCTGTGCTTCAGTACGCTCTCCCTGCGGCCCGAGGCCGTCGCGCACCTGGAG

GCGGACCGGGACTACCTGGAGATCGAGGTGGGGGGCCGACGGCTGTTCTTCGTGAAGGCCCACGTACGCG

AGAGCCTGCTGAGCATCCTGCTGCGCGACTGGCTGGCCATGCGAAAGCAGATCCGCTCGCGGATCCCCCA

GAGCACCCCCGAGGAGGCCGTCCTCCTCGACAAGCAACAGGCCGCCATCAAGGTGGTGTGCAACTCGGTG

TACGGGTTCACCGGGGTGCAGCACGGTCTTCTGCCCTGCCTGCACGTGGCCGCCACCGTGACGACCATCG

GCCGCGAGATGCTCCTCGCGACGCGCGCGTACGTGCACGCGCGCTGGGCGGAGTTCGATCAGCTGCTGGC

CGACTTTCCGGAGGCGGCCGGCATGCGCGCCCCCGGTCCGTACTCCATGCGCATCATCTACGGGGACACG

GACTCCATTTTCGTTTTGTGCCGCGGCCTCACGGCCGCGGGCCTGGTGGCCATGGGCGACAAGATGGCGA

GCCACATCTCGCGCGCGCTGTTCCTCCCCCCGATCAAGCTCGAGTGCGAAAAAACGTTCACCAAGCTGCT

GCTCATCGCCAAGAAAAAGTACATCGGCGTCATCTGCGGGGGCAAGATGCTCATCAAGGGCGTGGATCTG

GTGCGCAAAAACAACTGCGCGTTTATCAACCGCACCTCCAGGGCCCTGGTCGACCTGCTGTTTTACGACG

ATACCGTATCCGGAGCGGCCGCCGCGTTAGCCGAGCGCCCCGCAGAGGAGTGGCTGGCGCGACCCCTGCC

CGAGGGACTGCAGGCGTTCGGGGCCGTCCTCGTAGACGCCCATCGGCGCATCACCGACCCGGAGAGGGAC

ATCCAGGACTTTGTCCTCACCGCCGAACTGAGCAGACACCCGCGCGCGTACACCAACAAGCGCCTGGCCC

ACCTGACGGTGTATTACAAGCTCATGGCCCGCCGCGCGCAGGTCCCGTCCATCAAGGACCGGATCCCGTA

CGTGATCGTGGCCCAGACCCGCGAGGTAGAGGAGACGGTCGCGCGGCTGGCCGCCCTCCGCGAGCTAGAC

GCCGCCGCCCCAGGGGACGAGCCCGCCCCCCCAGCGGCCCTGCCCTCCCCGGCCAAGCGCCCCCGGGAGA

CGCCGTCGCATGCCGACCCCCCGGGAGGCGCGTCCAAGCCCCGCAAGCTGCTGGTGTCCGAGCTGGCGGA

GGATCCCGGGTACGCCATCGCCCGGGGCGTTCCGCTCAACACGGACTATTACTTCTCGCACCTGCTGGGG

GCGGCCTGCGTGACGTTCAAGGCCCTGTTTGGAAATAACGCCAAGATCACCGAGAGTCTGTTAAAGAGGT

TTATTCCCGAGACGTGGCACCCCCCGGACGACGTGGCCGCGCGGCTCAGGGCCGCGGGGTTCGGGCCGGC

GGGGGCCGGCGCTACGGCGGAGGAAACTCGTCGAATGTTGCATAGAGCCTTTGATACTCTAGCATGAGCC

CCCCGTCGAAGCTGATGTCCCGCATCTTGCAATAAATGTCTGCGGCCGACACGGTCGGAATTTCCGCGTC

CGCTGGTTTCTCTGCGTTGCGTCTGACCACGAGCACAAACGTGCTCTGCCACACGTGGGCGGCGAACCGG

TAGCCGGGGCACGCGGTCAGCATCCGATCGATGAGCCGGTAGTGCAGGTGGGCCGACGTGCCGGGGAAGA

TGACGTACAGCATGTGGCCCCCGTACGTGGGGTCCGGGTAAAAAAGAAACCGGGGGTCGCACGCCCCCCC

TCCGCGCAGGATCGTGTGCACGAAAAAGAGCTCGGGCTGGCCGAGCGTATCGGCCAGGAGGTCCTGGAGG

GGGGTGCTGTGGCGGTCGGCCAGCACGACCAGGGAGGCCAGAAAGGTGCGGTGCTCAAAGATCGTATTGA

TCTGCTGCACGAAGGCCAGGATGAGGGCCTCGCGGCTGACGGTGGCCAGCCGCCCGTCGCCCGCGCTGCA

CGCGGGGCAGCAGCCCCCGATCCCCAGGTAGTAGCCCATGCCCGAGAGGGTCAGGCAGTTGTCGGCCACG

GTCTGGTCCAGGCTGAAGGGGAGCGACACGGGGGTCGTCTTCACCAGGGGCACGGAGAGCGAGCGCACGA

TGGCGATCTCCTCGGAGGGCGTCTGGGCGAGGGCGGCGAAGAAGCCGCGGTAGCGACGGCGCTCGTGCAG

GCAGAGCTCCAGCCTGCGCGCGTGCGACGGCAGGCTCTTGCGGGAGGCCCGGCGCTCCACGCCGGGGTTC

CCGGCGGCGGAAAAGCGCGACCGCCGCCGGGTCTTGTCGCGGCCGGGCCCGGGCCGGGAGCCGGAGCGAC

GGGGGGCGATGTCATACATAGGTACAGAGGGTGTGCTCCAGGGACAGGAGAGAGATCGAGTGTCGTCTGA

GCAGCGCGCCGGCCTCGCGGACAAATGTGGCCAGCGCGGTGGGCTTCGGCACAAATACCTGGTACGTCTT

GAAGGTGTAGATGAGGGCCCGCAGGGCTATACAGACCCGCCCCTCGAACTCGTTGCCGCAGGCCAACTTG

GCCTTGTGAAGCTGCAGCTCGTCGCGATGGTCGGCGCGGGGGTGGCCAAACAGGACCCAGGGGTCGACTT

CCATCTCCGTGATGGCGCACATCGGATCGCAGAACATGTGCTTGAAGATGGCCTCGGGGCCCGCGGCCCG

AAGCAGGCTCACGAACCGGCCCCCGTCCCCGGGCTGCGCCTCGGGGTCCGCCTCGAGCTGGTCCACGACC

GGCACTATGCAGTCGAAGAGGCTGGTGTTGTTCTCCGAGTAGCGGACGACGGACGCCCTCAGGCGTCGCA

TGGCCAGCCAGTAGGCCCGCACCAGCAACAGATTGCACAGCAGGCATTCCCCGCCGGTGCGCCCGCGCCC

CCGGCCGTGCTTCAGCACGGTGGCCATCAGCGGGCCCAGGTCCAGGTCGGGCTGGGGCTGGGGCTCGGCG

AACTGCGCAAAACGCGGGGCCGCGTCGCGCATGCGCGCCCCGCGGTGCGCTTCCCAGGACTCGCTGACCG

CGGCGCGGCGGGCGTCCGCGGCGGCGCGCAGCCGGGGCCCCGACTCCCAGACGGCGGGGGTGCCGGCGAG

CAGCAGCAGGATCAGGTCGGCGTACGCCCACGTCTCCGGCTCACCCCCCTGCGCCAGCGCCCCGGCGGCG

GCCTCGAACTCCCCGTTGCGGGCGGCGGCGCGCGTGCAGCAGCTGTCTCCGCCCCCGCGCTTGCCCTCGG

TGCAGTCGAGCAGGCGGGCGCAGTCCTTCCAGTTCATCAGGGCGGTGGTGAGGGAGGGTTGCGTTCCCGA

GCCCCCGCCCGCCCCCGCCCCCGCCCCGTCATCGCCCCCGGAGGCCAGGGTCCCGATGAGGGCCCGGGTT

GCGGACTGCGCGAGGAAGGAATAGTTGGAGTACTGCACCTTGGCGGCGCCCGGGGAGGGCGTCGGCCTGG

GTTGCTTCTGGGCGTGGCGCCCGGGCACCCCGCCGTCGGTCCGGAAGCAGCAGTGGAGAAAGAAATGCCG

GTGGATGTCGTTGATGGTCAGGGCGAAGCGCGCGAAGGAGCCGACAAGGGTCGCCTTCTTGGTGCGCAGG

AAGTGGTGGTCCATGACGTAGACGAACTCGAAGGCGGCCACGAAGATGCTCGCGGCGCAGTGGGGCGCGC

CCAGGCACTTGGCGCAGAGGAACGCGTAATCGGCCACCCACTGGGGCGAGAGGCGGTAGGCCTGCTTGTA

CAGCTCGATGGTGCGGCAGACCAGACAGGGGCGGTCCAGCGCGAAGGTGTCGACGGACGCCGCGGCGAAG

GGCCCCGTGTCCAAGAGTCCCTCTGCCGTGGGGTCTGCGGGCGGGCCGCGGGCGGACCCCGGCCCCCGCC

CCCCCGAAGCCTCGCGCGCGGCCCCGCGCGGCCGCGGGGGGGCGGGCGCGACGTCGCTCTCCACGTCCTC

GTCGAGCGCGCTCGCGGGCGGCACGCCTACCACGTGACAGGCCGCCAGGAGCTCGGCGCACAGGGCCTCG

TTAAGAGCCAGAAGGTCGGGATCGAAGGCCACATACGGACGCTCGAACGCGCCCTCCTTCCAGCTGCTGC

CCGGCGACTCTTCGCGCACGGCGGCGCTCGACGGCACCCCCGGGGCGGACGTCGCCATGGCCGGTCGAGC

GGGGCGCACGCGTCCGCGAACGTTACGGGACGCGATCCCCGACTGCGCGCTGCGGTCCCAGACCCTGGAA

AGTCTAGACGCGCGCTACGTCTCGCGAGACGGCGCGGGGGACGCGGCCGTCTGGTTCGAGGACATGACCC

CCGCCGAACTAGAGGTTATATTCCCGACCACGGACGCCAAGCTGAACTACCTCTCGCGGACGCAGCGGCT

GGCCTCCCTCCTGACGTACGCCGGGCCTATAAAAGCGCCCGACGGCCCCGCCGCCCCACATACGCAGGAC

ACCGCGTGCGTGCACGGCGAGCTGCTCGCCCGAAAGCGCGAACGGTTCGCGGCGGTCATTAACCGGTTCC

TGGACCTGCACCAGATCCTGCGGGGCTGACGCGCGCTTCGGCGGGGCACCGGCACCGGGACCGACTTGTT

TTACATAACAGTAGGGGGTGGGGGAACGCGCACCCTTGCCCGGTCGCGATGGCGGGGATGGGGAAGCCCT

ACGGCGGCCGCCCGGGGGACGCGTTCGAGGGTCTCGTTCAGCGCATCAGGCTCATTGTTCCCGCCACGCT

GCGCGGCGGGGGTGGGGAGTCGGGCCCCTACTCGCCATCCAACCCGCCCTCGAGATGTGCCTTCCAGTTC

CACGGCCAGGATGGGTCCGACGAGGCCTTCCCGATCGAGTACGTCCTGCGGCTCATGAACGACTGGGCCG

ATGTGCCCTGCAACCCCTACCTGCGCGTGCAGAACACCGGCGTTTCGGTGCTGTTTCAGGGGTTTTTTAA

CCGGCCCCACGGCGCCCCGGGGGGCGCGATCACGGCGGAGCAGACCAACGTGATTCTGCACTCCACCGAG

ACGACGGGACTGTCCCTCGGAGACCTGGACGACGTCAAGGGGCGCCTCGGCCTGGACGCCCGGCCGATGA

TGGCCAGCATGTGGATCAGCTGCTTTGTGCGCATGCCCCGGGTGCAGCTCGCGTTTCGGTTCATGGGCCC

CGAGGACGCCGTTCGCACGCGGCGGATCCTGTGTCGCGCCGCCGAGCAGGCCCTCGCCCGTCGCCGCCGG

TCCAGGCGGTCCCAGGATGACTACGGGGCGGTGGTGGTGGCGGCGGCGCACCACTCTTCCGGAGCGCCCG

GGCCGGGGGTCGCCGCCTCGGGCCCGCCAGCGCCGCCCGGACGGGGACCGGCCCGTCCGTGGCATCAGGC

CGTGCAGTTGTTCCGGGCCCCGCGTCCGGGCCCCCCGGCGCTTCTGTTGCTGGCGGCGGGGCTGTTTCTG

GGGGCCGCTATCTGGTGGGCGGTTGGCGCGCGCCTATGAAAGGGGGCGAGCCACCGTCCCGCCCGCCAGT

GCATCCCAGACGCCCGCGAGCCGCACATCCCCTCCGCTCCCGCCTCCGGCCCGATTCTTACGGCGCGACC

CAAGGTCCCGATGGCCGCCCCGCAGTTTCACCGCCCCAGCACCATTACCGCCGACAACGTCCGGGCGCTC

GGCATGCGCGGGCTCGTGTTGGCCACCAACAACGCTCAGTTCATCATGGATAACAGCTACCCGCATCCGC

ACGGAACGCAGGGTGCGGTGCGAGAGTTTCTTCGCGGGCAGGCCGCGGCGCTGACGGACCTCGGGGTGAC

CCACGCCAACAACACGTTCGCCCCGCAGCCTATGTTCGCGGGCGACGCCGCGGCCGAATGGCTGCGGCCC

TCGTTCGGTCTTAAGCGCACGTATTCCCCCTTTGTCGTTCGCGACCCCAAGACCCCCAGCACCCCGTGAG

TCCTCGGCGGGTCCCTCCGCGGCCGTCTCTCGTTGCCCCCCTTTCCCCCTTCCCGGGTGGTTCAATAAAA

AACACCAACATACGATATTCGCGTTTGATACGTTTATTGGGGGGGGTGTAGGGCCCAACGATCGGCGATT

AACAACACCAAACAATCGAGCGCGTCTAACCCAGTAACATGCGCACGTGATGTAGGCTGGTCAGCACGGC

GTTGCTGCGCTGAAACAGCGCCCTGCGGGTCCGCTGCAGCTGTTGTTGTATGCGGCGGCATGCGCGGATC

AAAACCGCCAGGGCGCTACGACCGGTGCTTCGTACGTAGCGTCGCGACAAGACGGCATTTGCCTGTACGG

GCAAGGGGCCAAATTGCGAGTGTGGTGACTGGAGGTGGTCGGCGGCCAATGGGCCGGGTGGTTCGTCGGC

GGGGGGCAAGTGCGGTTCCGGTGGGAGGGGGTCGAGCGCCTCGGTATCATCCGAGTCCGAGAAACGCAGG

GAGTCTGCGTCGGAGTGTTCATCATCGGAGGAGATGTGCAGCGTCTGAAGCAGCGATGCGGGTGGGGGCG

CGGAGTCGACGTGAAGCGCGAGAGAGGAAGCCCACGAAGTCACAGCGGACACTGGGAGGTGGGTGTTTGT

ATGTGTGGGAGACTCGGGCGTCGGGACCGAGTCTCGGCTCTGGGGTGTAAGCGTCCGAGTTACGGGCGGC

AGGGGCGGCTGGGGCAGGGGCGGCTGGGGCAGGGGCGGCTGGGGCAGGGGCGGCTGGGGCAGGGGCGGCT

GGGGCAGGGGCGGCTGGGGCAGGGGCGGCTGGGGCAGGGGCGGCTGGGGCAGGGGCGGCTGGGGCAGGGG

CGGCTGGGGCAGGGGCGGCTGGGGCACCGAGCGCGCGCGGATGCGCGTCCGCGCGGCGGGTTTGGTCGCG

GGTGACTGGGGTGGGGGGCGGCGGGCAACCGGGCCTCCGGGCACGACCCAACCGCACAAAGGCTCGCTCG

GGGCAACCGGGCCTGGGGCCAAAGGCGGGGGGCTGGTCTGGACGGCGGAGGTCGGGGGGGCAAGGCCCGG

AGAAGGCGGCACTGCCGCCGCTGCGGCGGAAACCGCGGCCGCGTGGTCGGCTGGGTCCCGGGGAGAGGGG

AGGGAGTTCAACGAGGCCGAGAGCGAGGCGACCGCGGGGCGCGTGAGGCGCCGGGGTGGGCCGGCCGCGG

GGCCCCGGGGGGGTGTCGGCGAGGGACCCGCTGTTGTCTGGCGGCGGCCGCGGCGGCGGTCGCCCCCGGG

GACGACCGCTCCTTCGGCGGGCGGAGGCGGGATGGGCGCGAGCGTGGGGGCGGGAAAGGCCCCGCGAGCC

GAGGCGGGGCCGGGCGGAAGGGGCAAAGCAGAAACCCAAGCCGGGGGCGCGGACTCCGGGGTGGGCGGCT

GGTCGGGAGGACGCGCGGAAGCGGCGACCGGGGCGACCGGGGCGGGGAGTGCCGGCGGACGCCACCCCTC

GGGGGGGGCGGAGGCCCGGGGCGCGCGCGATTTGGCACGCGTCCGGCGGGACCTGCGCACGCGCGGCACG

GCGGCGGAGAAAGCGGCGGCAGAGCCGGAAAAGGCCGGGGGAGGAAGCGCGGCATCCGCGGGGGGACTCG

GTGTGGGTGGCGAGGGCCGTGGGTCGTCGCGAGGGGCCACGGGCACGCGCCCCGTGTTTTGTTGAGGCGG

GACACTCGGTCGTGTTTCGCGAGCCGTAGCTGCCGGCCCGATGGGCCGCGGTGCGTACTGGGACGTGGGG

ACGGACTGATCGGTGGCGGGGGGGGGAAGAAGGGCCGGGGCCGGATTGGGCGTGGGGCCGCCGGCGTCGT

CGGACGCCAGCTCCTCCAGGCCGTGGATCCAGGCCCACATGCGAGGGGGGACGGGCTCGCCGGTGGTGGC

GTCGGTGAGGAGAGTGGGGGCGAGGACCCCCGGGTCCGCCTGCCGTGCGGGGGGGGCAGCGGGGTCCTCG

GGACCCGATCCGCCATCCCCCCCCGCAAGGTCCCGCGGGTCGCGGGCGGCGGTCGGGGCAGAGGGACCTG

CCTCGTCGGCGAGGGGGCGCTGGTAAACCGGGTGTCCCGGGAACAGCTCCCCCGTCAGGAGGGAGGCGTC

GAAGGGCCGCCCGAGGATGGCCCGCGCGAAGAAGGGGTCCGCGTCGGCGGCGCTCGCCGCGAGAACGTCC

CCCGCGGTAGCCACAAACGGAAGCTCCTCGGTGGCCTCGCTGCCCACAAACCGCACGTCAGGGGGGCCGG

GGGGCTCCGGGGCTTCCCACAAGACCGCGACCGGGGTCATGGAGATGTCCACGAGGACCAGGCACGGGGG

CCCGTCGGCGAGAGGGCGCTCGGCGATGAGCGCCGACAGGCGCGGGAGCTGCGCCGCCAGACACGCGTTT

TCGATCGGGTTGAGATCGGTGTGGAGGAGGCCGACGGCCCACGTCTCGATGTCGGACGACACGACGTCGC

GCAGGGCGGCGTCCGGCCCGCCGGGGCGCGAGTCGAAGAGCGTCAGGCACAGTTCCAGTTCCGACTCGCG

GGAGAAGGCCGTGGTGTTGCGGAGCGCCACCACGACGGGCGCGCCGAGGAGCACCGCGGCCAGAACCAGG

TCCATGGCCGTAACGCGCGCGGCGGGGGTGCGGTGGGTCGCGGCGGCCAGCACGGCCACGTGCTGGCCCG

TGGGTCGGTAGAGGGCGTGGGGGGCCTCGGGGAGGGACGCCTCGCGCCCCCCCGCCGGGCCGAGCGTCTG

GCCAGACTCCAGGCGTGCGGCCAGGAGGGCGTCGAAGCTGTCGTACTCGGTGTAGTCGTCGGGAAACATG

CAGGTCCACAGCGCGGCCAAAGCGGCGCTCGGCAGACACATGCGCCCGAGGACGCTCACCGCCGCCAGGG

CCTGGGCCGGACTGAGCTTCCCGAGCGCCGGGACGTCCCGGCGCTGGGTCCCGAGCTCCAAGGCCGAGCG

CCAGGGCGCCAGCGGGTCGGTTTCGGACAGCTTGCCCCGGCGCCAGTCGGCCAGCCGCGTGCCGAACAGG

AGGCCCCGGGTCGGGGGGCCTCCGTCCAAAAACGTCGGCAACACGCGGATGCGGGCGTCGGGATGCGGGG

TCAGGCGCTGGACGAACAGCATGGACTCCGCTGCGTCCTCGAACGCGCGTTCGAGGGTGAGGTGCATGTA

CTCGTGCTGGCGAACGAGGTCCAGGCGCCAGAAGTTGTAGATGTGTTCCGGAACGCCGGCCACCAGCGCG

ACCAGCACGTCGTTCTCGTTGAAGGCGACGCAGTGGCGCTGGGACCCCCGGGGGCCCGGCGGCGGACGCG

GCGCCGCCGCTCCGGACGCCCAGCCCAGCTGGGCCCAGCGACACCCAAACTCGCGCGTGAGGGTGGTGGC

GACGAGGGCGACGTACAGCTCGGCCGCCGCGTCCATCGAGGCGCCCCACGTCGCCTGGCGATGGCGCACG

AAGCGACCGAACAGCTGAAAGTTGGCGGCCTGGGCGTCGCTGAGGGCCAGCTGGAGCCGGTTCACGACGG

TCAGCACGTACATGGCCGTGACCGTCGGGGCCGATTCGAGGACGTCCGTCGGAAGCGGGGGCCGCACGCA

GGCCGCCTCGGGACGCATCAGCAGCGCGCCGAGTTTGTCGGTGACGGCCGGGAAGCATAGCGCGTACTGC

AGCGGCGTTCCGTCCGGGGCCAAAAAGCTGGTGGCGAACGGCAGATCCAGAGCGCTGACGGCCTCACGCA

GCACCAGGGGCCCCGGGTCTCCGCCGGCGCGCAGATACGCCTCGCCCCGGCGGCGCAGCAGCTGCGGGTC

GACCTCGTGGCCCTCGGGGGAAGAAGAGGCCCGGGCGCGGGCGTCGAGGGCGCGAAGATCAACGAGCAGG

GGCGCGGGCGCGGACTCCGCGCCCGCGCCCGTCTGGCCGCCGGCCCTGGCGTACGCGCTATATAAGCCCA

TGCGGTATTGGATGAGTTCCCGCGCGCCCCGGAACTCCTCCACCGCCCACGGGGCCAGGTCCGCGGCCGC

CGCGTCGAACTCCGCCAGCAGGCCCCCCAGGGCGTCAAAGTTCATCTCCCAGGGCACCCTGCGCACCACC

TCATCCCGCAGCCGGGCGCACAGGGCGGTGTGCTTGGTGACGCGCGCGCCCAGCTCCTCCACGGCCTCCG

CGCGCTCGGCGCCCTTGGCGCCCAGGACGCCCTGGTACCTGGCGGAAAGGCGCTCGTAGGCCGGCTGGGC

CCGCAGCCCCGACACCGTGTTGGTGGTGTCCTGCAGGGCGCGCAGCTGCTCGTGCATGGCGCGGAACCCC

TCGGGGGACTTCCAGGCGCCCCCCCGGACGCGGCCAAAGCGACCCCAGACCTCGTCCCACTCCGCCTCGG

CCTCCTCCAGGGACCTCCGCAGGGCGTCGACGCGGCGCCGAGTATCAAAGAGCGCCCCCAGGCGGCCGGC

GTGCCGCGCCAGGGGGCCGGGGCCGTCGCCGCGGGCGGCGCTTAGCGGGTGCGTCTCGAAGGTGCGCTGG

GCGTGCTCTAGCCAGATAACCGCGGGCACGTCGAGCTCGCGCGTTTTCTCGGTCTGATCCAACAGAACCT

CGACCTGGTCGGCGATCTCCGCCACCGAGCGCGCCTGGTCGAGCGTCTTGGCCACGGTCGCCGGGACGGC

GACCACCTTCAGCATGGTCTTGAGGTTGGCCAGGCCCTCGGCCTCGATCTGGGCCCGGCGCTCGCGCGCG

GCCAGCGCCTCCCGCAGGCCCGCCATGACCCGCTCGGTGGCCTCCGCGCGCTGCTGTTTGGCGCGCACCA

CTGCGTCCTTGGTCTCGGCCGTGTCCTGCCGGGTCACGAAGGCGACATACTCGGCGTACGCCGTGTTCTT

CACGGGGCTCTGGTCCACGCGCTCCAACGCCGCCGCGCACGCGACCAGCGCGTCCTCGCTGGGACACGGC

AGGGTGACCCCGGTCCGGACCAGCTCCGCGGTGGCCTCCGGGTCATTCCGGGCCGCGGATATCTGCTCCG

CGGCGGCCGCCAGGTCCAGGGGCACGCCGCCGAGCGCCCGGTGCACGTCGGCCCGGATGGCGTCCAGGCG

ATCGCGGAGCTCCACGTAGTCGGCGTAGCCATGTTGGAAGAACGGCACGTACCGGCGCAGGCCGGGCACG

CTCGTCATGTCGTCCGCCAGGCGCCCCACGGCCTCGTGGTAGTCGATAAACCCGTCGCCCGCCTGGGCCA

TTTCCAGGAGCCCCTCCGCGATGCGCAGCAGCCGCGCCAGGGGCTCGGCGTCGACCCGAAACATGTCGGC

GTAGGTTTCGGCGGCGGCGTGGAACGCCGCGCTCCAGCCGAGGCGGTGGATGGCGGCGAGCGGGGGGAGC

ATGGGGTGGCGCTGGTTCTCGGGGGTGTAGGGGTTAAACGCGAAGGCCGTATCCAGGGCGAGGGTGACCG

CCTCGGCGTTGGCCGCGAGCGCCTGCTCGGCGCGCTTGCGGAAGTCCCGGGGGTTGTAGCCGTGCGTGCC

CGCCAGCGCCTGCAGGCGGCGCAGCTCGACCACGTCGAACTCGGCGCGGTTCTCGACGCGGTCCAGCGCC

GCCTCGACGCCGGCGGCCCAGCGCTCGCTGCTGCCCCGGGCGCGCTGGGCCGCCATCTTCGCCGTCAGGT

CGGCGACGGCGGCCTCAAGTTCGTCGGCGCGGCGTCGCGTGGCGCCGATGACCTTGCCCAGCTCCTGCAG

GGCGCGCCCGCTGGGGGAATGGTCCCCGGCCGTCCCTTCGGCGTGCAGCAGGCCCCCGAACCCAGCCTCG

TGCCCCGCGAGGCTTTCCCGAGCAGCGGTCGTCGCGCGGGCCGCGGCATCGATGAGGGCGGCATGGTCCC

CCTCCGGCTGGGCGCAGGCCCGGCGCGCCTGGACTACCAGGTCGGCGGCCGCCGACCCCAGGGTCGTGAG

CTCGTCGATGGCCCCCCGCGCCTCCAGGGCCAGCCGAGTCGCCTTTACATACCCCGCGGCGCTATCGGCC

AGCACCGCGAGGAAGGACAGGGGCGAGGCCGGGTCGCGGGCGGCCGCGCCCAGGGCCGACACCGCGTCCG

CCAGGGCGCCATGCGCCCGCACGGCCGCGTCCACCGTCGCCGCGGGACTTGCCGTCGCGACGGCGGCGCT

CCCGGCGTTGATGGCGTTTGACACGGCTTTGGCGATTGTGGGGGCGTGATCGGAAAAGAACTGCACGAGG

ACCGGCGTCTCGGGGGCGTCGGCGAACAGGGTCTTCAGCACCACCACGAAGGCGGGATGCAGGCCGGCCA

GAGCCGTCGCGGTATCCGGGGTCGGGTGTTCCAGGGCCTCCCGGTACTGCCCCAGCAGCCCCCACAGGTC

CGCCCGCAGCGCCGCCGTGACTTCCGGGGGGGGGCCCCGGACGGCATCGGCCAGGTCGGTCCACCCCGCG

GGCAGGGAGGCCCGCAGGGTCGCCAGCACGGCCGGACACGCCTTTAGCCCCACAAAGTCCGGGAGGGGCC

GCAGGACCCCTTGGAGTTTGTGCAGGAACTTCTCCCGGGCGTCGTGGGCCACCTTGGCGCGCTCCCGCGC

GTCGTTGAGCATCGCCTCCAGGGCGTGGGCGCGCTCCCGAAGCCGGGAGCGCGCCTCCGGAGCGAGCTCC

GCCGTCATCTTGGCCGCCTCCATGGCCCTCGCCTGCCGCAGCGCGTCTTCGGCCATGCGCGTGGCCTCGG

GGGACAGCCCGCCCCCGTCGACGTACGGCGCGGGGCCGGTCGCCGGGACGAAGGCCGCGTCGCTGTCCAG

CTGCTGCGCGAGCGCCGCGTCGAGGGCGTCGAAGCGCTGCAGTTCGGCCAGCCCCGAGCTGCGCCGCGCC

TGCTGGTCGTTGATGCCGTGGATGCTGCGCGCCAGCTCTTCCAGGGGCTTGCGTTCGATGAGCCCCTGGG

TCGCGGCGTCGGTCAGGACCGAGAGCCAGGCCGCCAGGTCCTCGGGGGCATCTAGGGGCTGGCCCCGCTG

GAGCAGGTCCCGCAGCAGGATGGCCTGGGGGCTGGTGGCGAGGGGGGGCGGGGGGGGGAGCGCGGCGCGC

TGAGCGACGTCCCGCGTGTGTTGGTCAAAGGCCGGTAGCGATTCCAGCAACTGGACCATGGGCACGACCG

CGGCCGAGGCCACGTGAAACCGACAGTCGTGGCTGTCGCTGGCCTGCAGGGCCTTCGCGCTGTATACGGC

TCCCCGGTGGAAGTACTCCTTGACCGCGCTCTCGATCGCCCGGCGGGCCTGGATCCGCACGTCCTCCAGC

CGCGCCTGGATGGCCTCGGGGCCCAGGGCGGGCGGGCACGGGGCCCTGCCGCCGGCGCCCGGGGCGGCGG

GCACGGGCATCACGGTCAGGGGCCCGGCGCGCTGCGAGACCGAGTCGACCCCGCGGGCGAGGGCGTCTAA

GGCCTCGCGCATCTCGCGGGCCTCCGCCTCGACCCGCATCTCTTCGCCCCGGGCAAACTGGGCCAGCGCC

TGGATCCGATGGAGAAGCGGCTCCGGGTGCGTCGGGGTGGCGGGGGCGAACAGGGTGTTCGGGTGGGCGC

GCGAGCGCTCCAGGAGCCACTCTCCGAGGCGTGCGTACAGATTGGCCGGCGGGGCGGCGCGCAGCTGCAG

ATCCAGGTCCGCGAGGTCCCCGTAAAAGGCGTCCGTCTCCCGAATAACGTCCCTGGCGACCAGGACCAGC

TTAGCGAGGGCCAGGCGCCCGATCTGCGAATTTTCGTCCAGCACGTGCTGGATGAGGGGCCGGTGGGCGG

CCACGTCCGCCAGGCTCATGCGCGTGGACGCCAGGAAGTCCCCGACGGCCGTTTTGCGGGGCAGCATGCG

CAGGGTGAAGTCCAGCAGGGCCGCGGCCGGGCCGGCCACCCCGGCCTGCGTATGCGTGCGGGCCCCGTTC

TCGATCAAAAAGGCGAGGACGCGCTCAAAGAAGAAGATGACGCAGAGCTCCAACAGCCCCGGGTGCGCCG

GGTACGGCGACCGCAGGGCGTTGATGGTGAGCTGCGAACACGCGGCCACCTCGCGGGCCAGGGCGGCATC

GCGCGCCGCGAGCCGGACCGCCGTGGCGGCCACATTGGGGTGGACCTCGAACAGCTGCGCCAGGTCGGCG

CCGGGGGGCTCCGGGGGGCGGCGGGCCCCCAGCGTCTCGAGCACGGACGGCGACGACGGGCTCGCGGGCC

CGTCGTCGCCGCCGCCCTGCCCGGACTGCGGGGGGGTATCCGGTGCGGGAGGGACCGTGGCGGCTATGGG

CGTCGGGGAGGAGGCGGGGACCTCGGCGGCGACGGGGGCCTTCTTCTTGGGCGCGGACTTCTTCTTGGCC

TTGGCGGGCGGGGCCTTGGGGGCGGGCCTCTCGCCCGAGGTCAGATCCTCCACGCTGGACGGTGGGGTCC

AGGTGGGCCGGCGGCGCTTGGGCAAGCCGGTAGAATAGCGCGCCCGGTGGCGACCCACCGGCACTGCCCC

CACCTCCAGGACCCGCAGGTCCTCGGCTTCTTCGGCCGCGTCCCCGGCGGGTGTCTGCGGGGGCGGGGCG

GCGTGCGGTGGACCCGAGGCCGCGGCGTCCGGGGCCGAGGGCTTCGCGGGCGGGGTCCCCTCCAGGGCTG

CTGCCCACACATCATCGGGGGGGCGGTTTGGGTGCCCCGCCTGCGGTGTGTCGGGTGGGCCCGAGGCCCC

CCGGGGGGCCTCGGGGGGCCGGTCGGCCCCAGGGGTCTGGACGTGGGTGGGCGCGGGGAGCGCGGGGACG

ACCGGGCCCGAGCCTTCTCCGTCCCCCCTGGGGACCACACCGACAAAGAGCGCCCCGAGCCCCCCGATCT

CGCCCCGCAGGGGGTGGGTGATGGCCACGCGCCGCTCGACGAACGGTTCGTCCTGCAGGTAAGTCTCGCT

GGCCCCGTAGAGGTGCAGGGCCGCGGCGGTCAGGTCCGCCGGCGCCACGGCCCCCGGGCCGGAGGGCACA

AAAAACACCATGGCGCCCGCCCACCGCACCTTGGGGCGGTCGTGGGCGTAATACGTCAGGTACGGGTACA

CGTCGCCCGCCCGCACCTTGGCGATAAACGCGGGCGTTCCCGCGGGCAGGCCGTGCGGGTCAAACAGATA

GGCCGTGTCGCCGTCCCGGTAGAGCCCCATGCCCAGGGGGCCGATGGTCAGGAGCGTGTAGGACAGCGGC

CGCATGGCCCAGGGGCCGGCGAAGAACGTGTGCGCGGGGCATTGCGTCTCCAGCAGCCCCGCCGTGGGCT

CCCCGAAGAAGCCCACCTCGCCGTACACCCGCGAAAACACGCAACGCAGGCCGCCGCGCGCCGCCGGGTA

CTCCAGGAAGTTGGGGAGCTCGATAATGGAACACATGCGCGGCGGCCCGGAGCCCGCGGCCGCGCGCGTC

CACTCGCCCCCCTCCACCAGACATCCCTCGATGGCCTCCGCGGACAGCACGTCGCGGGGCCCCACGTCGA

AAAGAAGACTGAGAAACGACAGGGACGAGCGCATGCACGATACCGACCCCCCCGGCTCCAGATCGGTCGC

GAACTGGTTCCGAACACCGGTGACCACGATATCGCGATCCCCCTGGCGCTTCATCGTGGGGTGAGGTAGC

GCGGCCGGAATCATGTGTGCCGCGCCCGCCACGAGCGGGGCCTGTTTATGGGCCGGGCGTCCCGATGAGT

ACTGTTGTTTCCGCCGCCCGAACCCCCCCGCCCATCAACCGCCTGTTCGTCCCCCTAACCACACACCCGG

TATCGCGTGTGTGTGGTTTCCCGGGAAGACACATCCCACCCCATGAAGTTTTGCCCTTTTTTTCCGTCCC

GCACTACGCCACCTTTCCACCCCCCCCCAAAAAAACAACAACCAACTCCCAGATGGATGGGTGCGATAAT

AAAGCTTTATTATTGTTTAACCAAAGGCGAGTCCTACGGGTGTACCGGTGGTGTCTCCTGCGGCGTCATC

TCGTCGTCCTCCACGGGGGTGTTGGGCCAAGGGACCGTCTCGCGGCCCGCCGGGCGCGTCGACGGCGCGC

GGGCCTGCGTGTCCTGTGGGCCGGGTGTCGTGGGTTCGGGGGTGCTACCGCCGGCATCTTGGGCCTCCAG

GTCCCCGGGGGCCCCCGGGCCGGCGGAAGGCCGAAACGCCGAGGCGCGAAACACGCCGTCGGTGACCTGC

AGGAGCTCGTTTATTAATAGCCAGTCCATGCTCAGCGTAGCGGCCAGCCCCTGGGGAGACAGGTCCACGG

AGTCCGGAACCACCGTCGGCTGACCCAGGGGCCCCAGGCTGTAGTCCCCCCAGGCCCCCAGGTCATGACG

GTTCGTGAGCACGACGAGGTCTGCGGCCGGGCTGGGGGGCGCGTCCTCGGTCGCGTGGGCCATCACCTCC

TGAATGGCTGCGGTGCGCTGATCGGCCGAGCTGGCGAAGCGCGCCACGACCAGCGCGCGCTCCGTCTGCA

GGCCCTTCCACGTGTCGTGGAGTTCCTGAACGAACTCGGCCACCCGCTCGGGGCCCGTGGCCGCGCGTGC

GGCCTGATAGCCGGCCGAGAGGCGCCGCCAGCGCGCCAGGAACTGACTCATGTAACAGAACCCGGGGACC

TGGTCCCCCGACATCAACTTTGACGCCCTGGCGTGGATGCCCGACACGATGGCCAGGAACCCGTGGATTT

CCCGCCGCACGACGGCCAGCACGTTACCCTCGTGCGAGACCTGGGCCGCCAGCTCGTCGCATACCCCGAG

GTGCGCCGTCGTCTCGGTGACGACGGACCGCAGCCCCGCGAGGGACGCGACCAGCGCGCGCTTGGCGTCG

TGATACATGCCGCAGTACTGGCTCACCGCGTCGCCCATGGCCTCGGGGCGCCAGGGCCCCAGGCGCTCGT

GGGCGTCTGCGACCACGGCGTACAGGCGGTGCCCGTCGCTCTCGAACCGGCACTCAAAGAAGGCGGCGAG

CGTGCGCATGTGCAGCCGCAGCAGCACGATCGCGTCCTCCAGCTGGCGGACCAGGGGGTCGGCGCGCTCG

GCGAGCTCCTGCAGCACCCCCCGGGCCGCCAGGGCGTACATGCTGATCAGCAGCAGGCTGCTGCCCACCT

CGGGAGGCTGGGGGGGAGGCAGCTGGACCGCGGGCCGCAGCTGCTCGACGGCCCCCCTGGCGATCACGTA

CAGCTCGCGCAGCAGCTGCTCGATGTTGTCGGCCATCTGCATCGTGGGCCCGACGCCGGCCCGGGTGGCC

GGTTCGAGGAGGGTGATCAGCGCGCCCAATTTTGTGCGGTGCCCCTCGACGGTGGGGAGATAGCCCAGGC

CGAAGTCGCGCGCCCAGGCCAGCACCCGCAGGGCAAACTCGATGGGGCGGGGCAGGTAGGCAGCGTTGCA

CGTGGCCCTCAGCGCGTCCCCGACCACCAGGGCCAGCACGTAAGGGACGAACCCCGGGTCGGCGAGGACG

TTGGGGTGGATGCCCTCCAGGGCCGGGAAGCGGATCTTGGTGGCCGCGGCCAGGTGAACCGAGGGGGCGT

GGCTAGGCGGCCCGACGGGGAGCAGCGCGGACAGCGGCGTGGCCGGGGTGGTGGGGGTCAGGTCCCAGTG

GGTCTGGCCGTACACGTCGAGCCAGATGAGCGCCGTCTCGCGCAGGAGGCTGGGCTGGCCGGCGCTGAAG

CGGCGCTCGGCCGTCTCAAACTCCCCCACGAGCGTGCGCCGCAGGCTCGCCAGGTGTTCCGTCGGCACGG

CCGGGCCCATGATGCGCGCCAGCGTCTGGCTGAGGACGCCGCCCGACAGGCCGACCGCCTCACAGAGCCG

CCCGTGCGTGTGCTCGCTGGCGCCCTGGATCCGCCGGAACGTTTTCACGTAGCCGGCGTAGTGCCCGTAC

TCCCGCGCGAGCCCGAACACGTTCGCCCCCGCAAGGGCAATGCACCCAAAGAGCTGCTGGATCTCGCTGA

GCCCGTGGCCGGGGGGCGTCCGCGCGGGCACCCCCGCCACCAAAAACCCCTCCAGGGCCGATATGTACTG

GGTGCAGTGCGCGGGCGTGAACCCCGCGTCGGTAAGCGTGTTGATCACCACGGAGGGCGAGTTGCTGTTC

TGGACCAAAGCCCACGTCTGCTGCAGCAGCGCGAGGAGCCGTTGCTGGGCCCCGGCGGAGGGCGGCTCCC

CTAGCTGCAGCAGGCCGGTGACGGCCGGACGGAAGATGGCCAGCGCCGACGCACTCAGAAACGGCACGTC

GGGGTCGAAGACGGCCGCGTCCGTCCGCACGCGCGCCATCAGCGTCCCCGGGGGCGCGCACGCCGACCGC

GGGCTGACGCGGCTTAGGGCGGTCGACACGCGCACCTCCTCGCGACTGCGAACCATTTTGGTGGCCTCGA

GGGGCGGGATCATGATAGCCGGGTCGATCTCCCGCACCGTGTGCTGAAACTGGGCCAGCAGCGGCGGCGG

GACCACCGCGCCCCGATCGGGGGTCGTCAGGTAGTCGTCCACCAGCGCCAGCGTAAACAGGGCCCGCGTG

AGGGGGGTCAGGGCGGCGTCGTCGATGCGCTGTAGGTGCGCCGAGAACAGCGTCACCCAATTGCTGACCA

GGGCCAAGAACCGGAGACCCTCTTGCACGATCGGGGACGGGAAGAGCAGGCTGTACGCCGGGGTGGTCAG

GTTGGCGCCGGGTTGCCCCAGGGGAACCGGGGACATCTTAAGCGACATCTCCCCGAGGGCCTCCAGGGAG

GTCCGCGGGTTCATGGCCAGGCAGCTCTGGGTGACGGTCCGCCAGCGGTCGATCCACTCCACGGCACACT

GGCGGACGCGCACCGGCCCCAGGGCCGCCGTGGTGCGCAGCCCGGCGGCCTCCAGCGCGTGGGTCGTGTC

GGAGCCGGTGATCGCCAGGACCGTGTCCTTGATGACGTCCATCTCCCGGAAGGCCGCCTCGGGGGTCTCG

GGGAGCGCCACCGCCATGCGGTGCACCAGCAGCCCGGGGAGGTTCTCGGCCAAGAGCGCCGTCTCCGGAA

GCCCGTGGGCCCGGTGCAAGGCGCACAGTTGCTCCAGGAGCGGGTGCCAGCACGCCCGCGCCTCCGCCGG

GCCGACCGCCGCGCCCGACAACAGAAACGCCGCCGTGGCGGCGTGCAGTTTGGCCGCGGACAGAAACGCC

GGCTCGTCCGCGCTGCCCGCCGGCTCGCTCGAGGGGGAGGGCGGCCGGCGGAGGTTGGTCAGGCTCCCCA

ACAGGACCTGCAACGGTCCGTTTGGGGGTGGAGCGGACGGGGGGGTCATGCCGGCGGGCGCCGGGACCTG

GAGCGCGCTGTCCGACATGGCGACCGGCGTGCGCGCTCGGCGACGCGGCGCGGAGACCGCGGGCCCAAAC

GGGAATGACTGCCGCCGCCCTATACGGAGGGGCTAAGTATCGCCCGGGGACCCTTCGAAACCCCGGGCGT

GTCGCAAGTACGCCGCGAAGGCGCGGCGTGTTATACGGCGCGTTATGTCCCGGCATTCCGTTCGTGGGTT

CGGGCCCGGGTGCTGTCGGGTGGGAGTGTGTGTGGGGGGGGGCGGCGCGACGGCGGCCCGGACCAAGTGT

ATCGCGGCCGTTCCGTGGGGCGGCCCAACAGGCCCTTTAAACATTTGCGTATGCACCGGCCCAGCCAGTC

GGACACCGGAACCCACCAGAGGCGGAAGCCGCCTTCGCCCGTGAGGGTGCGTGTGTTTTCTGGTGGCGTG

TTTTTCCTTTCCGCCCTCCTCCCTCCCCACCTCCACCACCCCCCCCCACAACTCGCCCGTTGGCGATCGG

CGGGAAAACCATGAAAACCAAGCCACTCCCGACAGCCCCGATGGCGTGGGCCGAGAGTGCCGTGGAAACC

ACCACCAGCCCGCGCGAGCTCGCGGGCCACGCCCCGCTCCGGCGCGTCCTGCGCCCGCCCATCGCTCGCC

GCGACGGCCCGGTGCTTTTGGGGGACAGGGCCCCCAGGAGGACGGCCAGTACGATGTGGCTGCTGGGGAT

CGACCCCGCGGAGTCGTCTCCGGGAACGCGCGCTACCCGAGACGATACCGAGCAGGCCGTGGACAAGATC

CTCAGGGGAGCCCGGCGCGCGGGAGGGCTGACCGTCCCCGGCGCCCCCCGCTATCACCTGACCCGCCAGG

TAACCCTGACGGATCTCTGCCAACCAAACGCGGAGCCGGCCGGGGCGCTCCTTTTGGCCCTGCGGCACCC

CACCGACCTCCCCCACCTGGCCCGCCATCGGGCTCCGCCCGGCCGGCAGACCGAGCGACTGGCCGAGGCC

TGGGGCCAGCTCCTGGAGGCCTCCGCCCTGGGGTCCGGGCGGGCCGAGAGCGGCTGCGCGCGCGCGGGCC

TTGTGTCGTTTAACTTTCTGGTGGCCGCGTGCGCCGCCGCCTACGATGCGCGCGACGCCGCCGAGGCGGT

CCGGGCCCACATCACGACCAACTACGGCGGGACGCGGGCCGGGGCGCGGCTGGACCGGTTTTCCGAATGC

CTGCGCGCCATGGTCCACACGCACGTGTTTCCCCACGAGGTCATGCGGTTTTTCGGGGGGCTAGTGTCGT

GGGTCACACAGGACGAGCTGGCTAGCGTCACCGCCGTCTGCAGCGGACCCCAGGAGGCCACACACACCGG

CCACCCGGGCAGGCCCCGTTCGGCCGTTACCATCCCGGCCTGCGCCTTCGTGGACCTGGACGCCGAGCTG

TGCCTGGGGGGCCCCTGGGGGGCGTTCCTGTACTTGGTCTTCACCTACCGACAGTGCCGGGACCAAGAGC

TCTGTTGCGTGTACGTGGTCAAGAGCCAGCTCCCCCCGCGCGGACTGGAGGCGGCCCTCGAGCGGCTGTT

CGGGCGCCTCCGGATAACCAACACGATTCACGGGGCCGAGGACATGACGCCCCCTCCCCCGAACCGAAAC

GTTGACTTTCCGCTCGCCGTCCCGGCCGCGAGCTCGCAATCCCCGCGGTGCTCGGCGAGCCAAGTCACGA

ACCCCCAGTTTGTCGACAGGCTGTACCGCTGGCAGCCGGATCTGCGGGGGCGCCCTACCGCACGCACCTG

CACATACGCCGCCTTCGCAGAGCTGGGTGTCATGCCAGACGACAGCCCCCGCTGTCTGCACCGCACCGAG

CGGTTTGGGGCGGTCGGCGTTCCGGTTGTCATCCTGGAGGGCGTGGTGTGGCGCGCGGCGGGGTGGCGGG

CCTGCGCGTGATCGTCTATTGACGACGGCCGCCCAACCCGAGCGACCTTCCCCTCCCACTTCCCCCCCCC

TACACACCAACTCCGCCCTCGCCGTCTTGGCCGTGCGCGGCCCCGTGCGTCCGTCTCAATAAAGCCAGGT

TAAATCCGTGACGTGGTGTGTTTGGCGTGTGTCTCTGAAATGGCGGAAACCGACATGCAAATGGGATTCA

TGGACATGTTACACCCCCCTGACTCAGGAGATAGGCATATCCTCCTTAGATTGACTCAGCACACGATCGC

ACCCCACCCCTGTGTGCCGGGGATAAAAGCCAACGCGGGCGGTCTGGGTTACCACAACAGGTGGGTGCTT

CGGGGACTTGACGGTCGCCACTCTCCTGCGAGCCCTCACGTCTTCGCCCACCGATTCCTGTTGCGTTCCT

GTCGGCCGGTGCTGTCCTGTCGACAGATTGTTGGCGACTGCCCGGGTGATTCGTCGGCCGGTGCGTCCTT

TCGGTCGTACCGCCCACCCCGCCTCCCACGGGCCCGCCGCTGTTTCCGTTCATCGCGTCCGAGCCACCGT

CACCTTGGTTCCAATGGCCAACCGCCCTGCCGCATCCGCCCTCGCCGGAGCGCGGTCTCCGTCCGAACGA

CAGGAACCCCGGGAGCCCGAGGTCGCCCCCCCTGGCGGCGACCACGTGTTTTGCAGGAAAGTCAGCGGCG

TGATGGTGCTTTCCAGCGATCCCCCCGGCCCCGCGGCCTACCGCATTAGCGACAGCAGCTTTGTTCAATG

CGGCTCCAACTGCAGTATGATAATCGACGGAGACGTGGCGCGCGGTCATTTGCGTGACCTCGAGGGCGCT

ACGTCCACCGGCGCCTTCGTCGCGATCTCAAACGTCGCAGCCGGCGGGGATGGCCGAACCGCCGTCGTGG

CGCTCGGCGGAACCTCGGGCCCGTCCGCGACTACATCCGTGGGGACCCAGACGTCCGGGGAGTTCCTCCA

CGGGAACCCAAGGACCCCCGAACCCCAAGGACCCCAGGCTGTCCCCCCGCCCCCTCCTCCCCCCTTTCCA

TGGGGCCACGAGTGCTGCGCCCGTCGCGATGCCAGGGGCGGCGCCGAGAAGGACGTCGGGGCCGCGGAGT

CATGGTCAGACGGCCCGTCGTCCGACTCCGAAACGGAGGACTCGGACTCCTCGGACGAGGATACGGGTTC

GGAGACGCTGTCTCGATCCTCTTCGATCTGGGCCGCAGGGGCGACTGACGACGATGACAGCGACTCCGAC

TCGCGGTCGGACGACTCCGTGCAGCCCGACGTTGTCGTTCGTCGCAGATGGAGCGACGGCCCCGCCCCCG

TGGCCTTTCCCAAGCCCCGGCGCCCCGGCGACTCCCCCGGAAACCCCGGCCTGGGCGCCGGCACCGGGCC

GGGCTCCGCGACGGACCCGCGCGCGTCGGCCGACTCCGATTCCGCGGCCCACGCCGCCGCACCCCAGGCG

GACGTGGCGCCGGTTCTGGACAGCCAGCCCACTGTGGGAACGGACCCCGGCTACCCAGTCCCCCTAGAAC

TCACGCCCGAGAACGCGGAGGCGGTGGCGCGGTTTCTGGGGGACGCCGTCGACCGCGAGCCCGCGCTCAT

GCTGGAGTACTTCTGTCGGTGCGCCCGCGAGGAGAGCAAGCGCGTGCCCCCACGAACCTTCGGCAGCGCC

CCCCGCCTCACGGAGGACGACTTTGGGCTCCTGAACTACGCGCTCGCTGAGATGCGACGCCTGTGCCTGG

ACCTTCCCCCGGTCCCCCCCAACGCATACACGCCCTATCATCTGAGGGAGTATGCGACGCGGCTGGTTAA

CGGGTTCAAACCCCTGGTGCGGCGGTCCGCCCGCCTGTATCGCATCCTGGGGGTTCTGGTCCACCTGCGC

ATCCGTACCCGGGAGGCCTCCTTTGAGGAATGGATGCGCTCCAAGGAGGTGGACCTGGACTTCGGGCTGA

CGGAAAGGCTTCGCGAACACGAGGCCCAGCTAATGATCCTGGCCCAGGCCCTGAACCCCTACGACTGTCT

GATCCACAGCACCCCGAACACGCTCGTCGAGCGGGGGCTGCAGTCGGCGCTGAAGTACGAAGAGTTTTAC

CTCAAGCGCTTCGGCGGGCACTACATGGAGTCCGTCTTCCAGATGTACACCCGCATCGCCGGGTTTCTGG

CGTGCCGGGCGACCCGCGGCATGCGCCACATCGCCCTGGGGCGACAGGGGTCGTGGTGGGAAATGTTCAA

GTTCTTTTTCCACCGCCTCTACGACCACCAGATCGTGCCGTCCACCCCCGCCATGCTGAACCTCGGAACC

CGCAACTACTACACGTCCAGCTGCTACCTGGTAAACCCCCAGGCCACCACTAACCAGGCCACCCTCCGGG

CCATCACCGGCAACGTGAGCGCCATCCTCGCCCGCAACGGGGGCATCGGGCTGTGCATGCAGGCGTTCAA

CGACGCCAGCCCCGGCACCGCCAGCATCATGCCGGCCCTGAAGGTCCTCGACTCCCTGGTGGCGGCGCAC

AACAAACAGAGCACGCGCCCCACCGGGGCGTGCGTGTACCTGGAACCCTGGCACAGCGACGTTCGGGCCG

TGCTCAGAATGAAGGGCGTCCTCGCCGGCGAGGAGGCCCAGCGCTGCGACAACATCTTCAGCGCCCTCTG

GATGCCGGACCTGTTCTTCAAGCGCCTGATCCGCCACCTCGACGGCGAGAAAAACGTCACCTGGTCCCTG

TTCGACCGGGACACCAGCATGTCGCTCGCCGACTTTCACGGCGAGGAGTTCGAGAAGCTGTACGAGCACC

TCGAGGCCATGGGGTTCGGCGAAACGATCCCCATCCAGGACCTGGCGTACGCCATCGTGCGCAGCGCGGC

CACCACCGGAAGCCCCTTCATCATGTTTAAGGACGCGGTAAACCGCCACTACATCTACGACACGCAAGGG

GCGGCCATCGCCGGCTCCAACCTCTGCACCGAGATCGTCCACCCGGCCTCCAAGCGATCCAGTGGGGTCT

GCAACCTGGGAAGCGTGAATCTGGCCCGATGCGTCTCCAGGCAGACGTTTGACTTTGGGCGGCTCCGCGA

CGCCGTGCAGGCGTGCGTGCTGATGGTGAACATCATGATCGACAGCACGCTACAACCCACGCCCCAGTGC

ACCCGCGGCAACGACAACCTGCGGTCCATGGGCATTGGCATGCAGGGCCTGCACACGGCGTGCCTCAAGA

TGGGCCTGGATCTGGAGTCGGCCGAGTTCCGGGACCTGAACACACACATCGCCGAGGTGATGCTGCTCGC

GGCCATGAAGACCAGTAACGCGCTGTGCGTTCGCGGGGCGCGTCCCTTCAGCCACTTTAAGCGCAGCATG

TACCGGGCCGGCCGCTTTCACTGGGAGCGCTTTTCGAACGCCAGCCCGCGGTACGAGGGCGAGTGGGAGA

TGCTACGCCAGAGCATGATGAAACACGGCCTGCGCAACAGCCAGTTCATCGCGCTCATGCCCACCGCCGC

CTCGGCCCAGATCTCGGACGTCAGCGAGGGCTTTGCCCCCCTGTTCACCAACCTGTTCAGCAAGGTGACC

AGGGACGGCGAGACGCTGCGCCCCAACACGCTCTTGCTGAAGGAACTCGAGCGCACGTTCGGCGGGAAGC

GGCTCCTGGACGCGATGGACGGGCTCGAGGCCAAGCAGTGGTCTGTGGCCCAGGCCCTGCCTTGCCTGGA

CCCCGCCCACCCCCTCCGGCGGTTCAAGACGGCCTTCGACTACGACCAGGAACTGCTGATCGACCTGTGT

GCAGACCGCGCCCCCTATGTTGATCACAGCCAATCCATGACTCTGTATGTCACAGAGAAGGCGGACGGGA

CGCTCCCCGCCTCCACCCTGGTCCGCCTTCTCGTCCACGCATATAAGCGCGGCCTGAAGACGGGGATGTA

CTACTGCAAGGTTCGCAAGGCGACCAACAGCGGGGTGTTCGCCGGCGACGACAACATCGTCTGCACAAGC

TGCGCGCTGTAAGCAACAGCGCTCCGATCGGGGTCAGGCGTCGCTCTCGGTCCCGCATATCGCCATGGAT

CCCGCCGTCTCCCCCGCGAGCACCGACCCCCTAGATACCCACGCGTCGGGGGCCGGGGCGGCCCCGATTC

CGGTGTGCCCCACCCCCGAGCGGTACTTCTACACCTCCCAGTGCCCCGACATCAACCACCTTCGCTCCCT

CAGCATCCTGAACCGCTGGCTGGAGACCGAGCTCGTGTTCGTGGGGGACGAGGAGGACGTCTCCAAGCTC

TCCGAGGGCGAGCTCGGCTTCTACCGCTTTCTGTTTGCCTTCCTGTCGGCCGCGGACGACCTGGTGACGG

AAAACCTGGGCGGCCTCTCCGGCCTCTTCGAACAGAAGGACATTCTTCACTACTACGTGGAGCAGGAATG

CATCGAGGTCGTCCACTCGCGCGTCTACAACATCATCCAGCTGGTGCTCTTTCACAACAACGACCAGGCG

CGCCGCGCCTATGTGGCCCGCACCATCAACCACCCGGCCATTCGCGTCAAGGTGGACTGGCTGGAGGCGC

GGGTGCGGGAATGCGACTCGATCCCGGAGAAGTTCATCCTCATGATCCTCATCGAGGGCGTCTTTTTTGC

CGCCTCGTTCGCCGCCATCGCGTACCTGCGCACCAACAACCTCCTGCGGGTCACCTGCCAGTCGAACGAC

CTCATCAGCCGCGACGAGGCCGTGCATACGACAGCCTCGTGCTACATCTACAACAACTACCTCGGGGGCC

ACGCCAAGCCCGAGGCGGCGCGCGTGTACCGGCTGTTTCGGGAGGCGGTGGATATCGAGATCGGGTTCAT

CCGATCCCAGGCCCCGACGGACAGCTCTATCCTGAGTCCGGGGGCCCTGGCGGCCATCGAGAACTACGTG

CGATTCAGCGCGGATCGCCTGCTGGGCCTGATCCATATGCAGCCCCTGTATTCCGCCCCCGCCCCCGACG

CCAGCTTTCCCCTCAGCCTCATGTCCACCGACAAACACACCAACTTCTTCGAGTGCCGCAGCACCTCGTA

CGCCGGGGCCGTCGTCAACGATCTGTGAGGGTCTGGGCGCCCTTGTAGCGATGTCTAACCGAAATAAAGG

GGTCGAAACGGACTGTTGGGTCTCCGGTGTGATTATTACGCAGGGGAGGGGGGTGGCGGCTGGGGAAAGG

GAAGGAACGCCCGAAACCAGAGAAAAGGACCAAAAGGGAAACGCGTCCAACCGATAAATCAAGCGCCGAC

CAGAACCCCGAGATGCATAATAACGATTTTATTACTCTTATTATTAACAGGTCGGGCATCGGGAGGGGAT

GGGGGCGCGCGTTTCCTCCGTTCCGGCTACTCGTCCCAGAATTTAGCCAGGACGTCCTTGTAAAACGCGG

GCGGGGGCGCGTGGGCCCACAGCTGCGCCAGAAACCGGTCGGCGATGTCCGGGGCGGTGATATGCCGAGT

CACGATGGAGCGCGCTAAATCTTCGTCGCGGAGGTCCTGATAGATGGGCAGTCTTTTTAGAAGAGTCCAG

GGTCCCCGCTCCTTGGGGCTGATAAGCGATATGACGTACTTGACGTATCTGTGCTCCACCAGCTCGGCGA

TGGTCATCGGATCGGGCAGCCAGTCCAGGGCCTCCGGGGCGTCGTGGATGACGTGGCGGCGACGTCCGGC

GACATAGCCGCGGTGTTCCGCGACCCGCTGCGCGTTGGGGACCTGCACGAGCTCGGGCGGGGTGAGTATC

TCCGAGGAGGACGACCGGGCGCCGTCGCGCGGCCCACCGGCGACGTCCGGGGGCTGGAGGGGGGGGTCTT

CTTCGTAGTCGTCCTCGCCCGCGATCTGTTGGGCCAGAATTTCGGTCCACGAGATGCGCGTCTCGAGGCC

GACCGGGGCCGCGGTCAGCGTAGGCATGCTCTCCAGGGAGCGCGAGTTGGCGCGCTCCCGCCGGGCCGCC

CGGCGGGCCTGGGATCGGCTCGGGGCGGTCCAGTGACACTCGCGCAGCACGTCCTCGACGGACGCGTAGG

TGTTATTGGGGTGCAGGTCTGTGTGGCAGCGGACGAACAGCGCCAGGAACTGCGGGTAACTCATCTTGAA

GTACTGCAGCAGGTCGCGGCAGTGAATCGTCGGAATGTAGCCGGTGCTGATGTCCAACACGATATCGCAG

CCCATCAGCAGGAGATCGGTATCCGTGGTATGCACGTACGCGACCGTGTTGGTATGATAGAGGTTCGCGC

AGGCGTCGTCGGCCTCCAGCTGACCCGAGTTGATGTAGGCGTACCCCAGCGCCCGCAGAACGCGGATACA

GAACAGGTGAGCCAGGCGCAGGGCCGGCTTCGAGGGCGCGCCCGAGGGGGCCGCCGGGCCTGGGCCGGCG

GCCCGCGTTCCCCGGTCCCCCGGGGCGAAGGCGTGCCCGCGGCGGCGCATGTTGGAAAAGGCGAAACTGG

GCCTGGAGTCGGTGATGGGGGAAGGCGGCGGCGAGGCGTCTACGTCACTGGCCTCCTCGTCCGTGCGGCA

CTGGGCCGTCGTGCGGGCCAGGATCGCCTTGGCCCCGAACACAACCGGCTCGGTACACTCGACCCCGCGA

TCGGTCACGAAGATGGGGAACAGGGACTTTTGGGTAAACACCCGTAACATACTACAGAGACAGTGTAGCG

TGATTGCCTCGCGGTCGTAACTTGGGTAGCGGCGCTGATATTTAACCACCAGGGTATACATGACATTCCA

CAGGTCCACGGCGATGGGGGTAAAGTAGCCCTCCGGGGCCCGGAGGCCCCGGCGCTTCACCAGATGGTGA

GTCTGGGCAAACTTCATCATGCCAAACAGACCCATTCCGGCACGATTGTAGGTGCGGATAGGTCTCTCTA

CAGAGCTGTATAGGTGTGACGGTCCGGGACACCCAAGCCCGCCGCCCCTGTGTACAGTGGCTGCGGCGAC

GACCCCGCTCCAACAAGACGCTATCCCGGGAAAGGCACGCTCTTTATAATTCTTTTTTATTTCCCATCTA

CGTGCGGATTGGTGCAACCGCCGGCGCGCGCCGGTGCAGGCCGACCATCTCTCTCTTCCCCCCCTCCCCC

TCCCCCGAGCCCTCAAAGAGGGTGTGGCCTAACTAGCGGAAGGCGTATTTAACCAGACTAGGGCGGCGGG

TCCGCCGTAGTCCTTGGCTCGGGTAGCCACTGCTCTGTGGCTCGGGTCCCCCGGCCCCCCTAACCCCCAT

CCGGTCCGCGTCATCCGCCCCCTCCGCCTGCGACACAAACGGCCGCGCCTCCGGGCCCGGTGACACGACG

CGCCTCGTCTCTGCGGATTGTCCCGGGAGCGTCGCGGCATGGCTCATCTTCCCGGCGGTGCGGCCGCCGC

CCCCCTTTCGGAGGACGCGATCCCGTCGCCGCGCGAGCGGACGGAAGACTGGCCGCCCTGCCAGATAGTG

CTGCAGGGCGCCGAGCTGAACGGGATCCTGCAGGCCTTTGCGCCGCTTCGCACGAGCCTTTTGGACTCGC

TCCTGGTCGTGGGCGACCGAGGCATCCTTGTACATAACGCGATTTTCGGCGAGCAGGTGTTTCTGCCCCT

CGACCATTCGCAGTTCAGTCGCTATCGATGGGGCGGACCCACCGCGGCGTTCCTGTCTCTCGTGGACCAG

AAGCGATCCCTGCTGAGCGTGTTTCGCGCCAACCAGTACCCTGACCTGCGGCGGGTGGAGCTGACGGTCA

CGGGCCAGGCCCCGTTTCGCACGCTGGTGCAGCGCATATGGACGACCGCGTCCGACGGAGAGGCCGTGGA

GCTTGCCAGCGAGACGCTCATGAAACGCGAGTTGACGAGCTTCGCGGTACTACTCCCCCAGGGCGACCCC

GACGTCCAGCTGCGCCTCACGAAGCCCCAGCTCACGAAGGTGGTGAACGCCGTCGGGGACGAGACCGCCA

AACCCACCACGTTCGAGCTCGGCCCCAACGGCAAGTTTTCCGTGTTTAACGCGCGCACCTGCGTCACCTT

TGCCGCCCGCGAGGAGGGCGCGTCGTCCAGCACCAGCGCCCAGGTCCAGATTCTGACCAGCGCGCTGAAG

AAGGCGGGCCAAGCGGCCGCCAACGCCAAGACGGTCTACGGGGAAAACACACACCGCACATTCTCGGTGG

TCGTCGACGACTGCAGCATGCGGGCGGTCCTCCGGCGGCTCCAGGTCGGCGGGGGGACCCTCAAGTTCTT

CCTCACGGCCGACGTCCCCAGCGTGTGTGTCACCGCCACCGGCCCCAACGCGGTGTCGGCGGTGTTTCTT

TTAAAACCCCAGCGGGTCTGCCTGAACTGGCTCGGCCGGAGCCCGGGTTCCTCGACCGGGAGCTTGGCGT

CCCAGGACTCTCGGGCCGGCCCGACCGACAGCCAGGACTCCTCCTCCGAGCCGGACGCGGGCGACCGCGG

CGCCCCAGAAGAAGAAGGCCTCGAGGGCCAGGCCCGGGTACCGCCCGCGTTCCCGGAACCGCCGGGAACC

AAGCGGAGGCACCCCGGGGCCGAAGTTGTCCCCGCGGACGACGCCACCAAGCGCCCGAAGACGGGCGTGC

CCGCCGCCCCCACGCGAGCCGAGTCGCCCCCCCTCTCCGCGAGATACGGACCCGAGGCGGCGGAGGGTGG

TGGGGACGGCGGCCGCTACGCGTGCTACTTTCGCGACCTCCAGACCGGCGACGCGAGCCCCAGCCCCCTC

TCCGCCTTCCGGGGTCCCCAAAGACCCCCATACGGCTTTGGGTTGCCCTGACGGCAACGGGTGGTGGCCG

AACGCCTCACCGCGCCCGGGCACGCGGGGTGCGTTGTGTTAAAAAAATAAATAAATGGGGTAGTGTGTCC

CCCCCCCCTCCAACCAATATGGCTGTCGTGTGTGGTTCCGGGTTGCGCCTCCGTCCTTTCCACCCCCCTT

CCCCCTCCTTTTTTGTTTTGCGTGCGCTTATAAGAGCGGGCCCGGGGCCCTTCGCAGCTTCACCGAGAGC

GCCGTCGGGCCCCGGGTGCGGGATGTGTCGCGGGGACAGCCCCGGGGTCGCGGGCGGGAGCGGCGAACAC

TGCCTCGGAGGGGATGATGGGGACGACGGGCGCCCCCGCCTCGCCTGCGTGGGTGCCATCGCTCGGGGGT

TCGCGCATCTCTGGCTCCAGGCCGCCACGCTGGGCTTCGTGGGGTCTGTCGTTCTGTCGCGCGGCCCGTA

TGCGGACGCCATGTCGGGGGCGTTCGTGATCGGGAGCACCGGCCTGGGGTTCCTCCGCGCCCCCCCCGCG

TTCGCCCGGCCGCCGACGCGTGTGTGCGCGTGGCTGAGGCTGGTCGGCGGGGGAGCGGCCGTGGCCCTGT

GGAGCCTCGGGGAGGCCGGCGCGCCTCCGGGGGTTCCGGGCCCGGCGACCCAGTGCCTGGCGCTCGGGGC

CGCCTACGCGGCGCTGCTGGTGCTGGCCGACGACGTCCATCCCCTTTTCCTCCTCGCCCCGCGGCCCCTG

TTTGTCGGCACCCTGGGGGTTGTCGTCGGCGGGCTGACGATAGGCGGCAGTGCGCGCTACTGGTGGATCG

ACCCCCGCGCCGCCGCGGCCCTGACGGCGGCGGTGGTGGCGGGCCTCGGGACAACCGCCGCCGGGGACAG

CTTTTCCAAGGCCTGTCCCCGCCACCGCCGCTTTTGCGTCGTCTCCGCGGTCGAGTCTCCCCCGCCCCGA

TACGCCCCGGAGGACGCCGAGCGGCCAACAGACCACGGACCCCTGTTACCGTCGACGCACCACCAGCGAT

CTCCGCGGGTCTGCGGCGACGGGGCCGCACGGCCCGAAAACATCTGGGTTCCCGTGGTGACCTTTGCGGG

CGCGCTCGCGCTGGCCGCCTGCGCCGCGCGAGGGTCTGACGCGGCTCCGTCAGGCCCGGTCCTGCCGCTG

TGGCCCCAGGTGTTTGTCGGGGGCCACGCGGCGGCGGGCCTGACGGAGCTGTGTCAGACCCTCGCGCCCC

GGGACCTCACGGACCCGCTGCTGTTTGCGTACGTCGGATTCCAGGTCGTGAACCACGGGCTGATGTTTGT

GGTCCCCGACATCGCCGTATACGCGATGCTGGGGGGCGCCGTGTGGATCTCGCTGACGCAGGTGCTTGGG

CTCCGGCGCCGCCTTCACAAGGACCCAGACGCCGGGCCCTGGGCGGCCGCGACCCTGCGGGGCCTCTTTT

TCTCCGTCTACGCATTGGGGTTTGCGGCGGGGGTGCTGGTGCGGCCGCGGATGGCGGCGAGCCGGCGGTC

GGGGTGATCGCCATTTCAAATAAAAGGCACGAGTTCCCCGAATACCACCGGCGTGTGATGATTTCGCCCT

ACCGCTCCGATCCCCGGGGGGAGGGGGGAAGGAAATGGGGGCGGGGGTGCCGTGGACGGGTATAAAGGCC

AGGGGGGCAGGCGGGCCCATCACTGTTAGGGTGTTAGGTTGGGAGGTGGCACAAAAAGCGACACTCCCGT

GTTGTAGTTGTCCGCGGGAGGCGGTGGTTTCCGGCAACCCTCCTCGCTGCGCCGGGCGCGCCCACCGGTC

CTTCGCGGGGGCCGGGGCTCTTCTGGTCATGGCCCTTGGACGGGTGGGCCTAGCCGTGGGCCTGTGGGGC

CTGCTGTGGGTGGGTGTGGTCGTGGTGCTGGCCAATGCCTCCCCCGGACGCACGATAACGGTGGGCCCGC

GGGGGAACGCGAGCAATGCCGCCCCCTCCGCGTCCCCGCGGAACGCATCCGCCCCCCGAACCACACCCAC

GCCCCCCCAACCCCGCAAGGCGACGAAAAGTAAGGCCTCCACCGCCAAACCGGCCCCGCCCCCCAAGACC

GGGCCCCCGAAGACATCCTCGGAGCCCGTGCGATGCAACCGCCACGACCCGCTGGCCCGGTACGGCTCGC

GGGTGCAAATCCGATGCCGGTTTCCCAACTCCACCCGCACGGAGTTCCGCCTCCAGATCTGGCGTTATGC

CACGGCGACGGACGCCGAGATCGGAACGGCGCCTAGCTTAGAGGAGGTGATGGTAAACGTGTCGGCCCCG

CCCGGGGGCCAACTGGTGTATGACAGCGCCCCCAACCGAACGGACCCGCACGTGATCTGGGCGGAGGGCG

CCGGCCCGGGCGCCAGCCCGCGGCTGTACTCGGTCGTCGGGCCGCTGGGTCGGCAGCGGCTCATCATCGA

AGAGCTGACCCTGGAGACCCAGGGCATGTACTACTGGGTGTGGGGCCGGACGGACCGCCCGTCCGCGTAC

GGGACCTGGGTGCGCGTTCGCGTGTTCCGCCCTCCGTCGCTGACCATCCACCCCCACGCGGTGCTGGAGG

GCCAGCCGTTTAAGGCGACGTGCACGGCCGCCACCTACTACCCGGGCAACCGCGCGGAGTTCGTCTGGTT

CGAGGACGGTCGCCGGGTGTTCGATCCGGCCCAGATACACACGCAGACGCAGGAGAACCCCGACGGCTTT

TCCACCGTCTCCACCGTGACCTCCGCGGCCGTCGGCGGCCAGGGCCCCCCGCGCACCTTCACCTGCCAGC

TGACGTGGCACCGCGACTCCGTGTCGTTCTCTCGGCGCAACGCCAGCGGCACGGCATCGGTGCTGCCGCG

GCCAACCATCACCATGGAGTTTACGGGCGACCATGCGGTCTGCACGGCCGGCTGTGTGCCCGAGGGGGTG

ACGTTTGCCTGGTTCCTGGGGGACGACTCCTCGCCGGCGGAGAAGGTGGCCGTCGCGTCCCAGACATCGT

GCGGGCGCCCCGGCACCGCCACGATCCGCTCCACCCTGCCGGTCTCGTACGAGCAGACCGAGTACATCTG

CCGGCTGGCGGGATACCCGGACGGAATTCCGGTCCTAGAGCACCACGGCAGCCACCAGCCCCCGCCGCGG

GACCCCACCGAGCGGCAGGTGATCCGGGCGGTGGAGGGGGCGGGGATCGGAGTGGCTGTCCTTGTCGCGG

TGGTTCTGGCCGGGACCGCGGTAGTGTACCTCACCCACGCCTCCTCGGTGCGCTATCGTCGGCTGCGGTA

ACTCCGGGGCCGGGCCCGGCCGCCGGTTGTCTTCTTTTCCACCCCTTCCGTCCCCCGTACCCACCACACC

CCACCCCACCCCCCCGCCGTCCCCCGGGCGTTATAAGCCGCCGCACTCGCTTTTCCCACCGGAAAATCCT

CGGCCCGATCCGAACGGCGCACGCCGCGTGGGCTCCAAACGCCTCCGGAAGAGAGCGCCCCGCCCCGATA

TTCAAGCCCGCGGTGGTGCTATGGCTTTCCGTGCTTCGGGACCCGCCTACCAGCCCCTCGCCCCCGCGGC

CTCCCCGGCGCGGGCTCGTGTTCCGGCCGTGGCCTGGATCGGCGTCGGAGCGATCGTCGGGGCCTTTGCG

CTCGTCGCCGCGTTGGTTCTCGTACCCCCTCGGTCCTCGTGGGGACTCTCGCCGTGCGACAGCGGCTGGC

AGGAATTCAACGCGGGATGCGTCGCGTGGGACCCCACCCCCGTCGAGCACGAGCAGGCGGTCGGCGGCTG

CAGCGCGCCGGCCACCCTTATCCCCCGTGCGGCCGCCAAGCACCTGGCCGCTCTGACACGCGTCCAGGCG

GAGAGATCGTCGGGTTACTGGTGGGTGAACGGAGACGGCATCCGGACCTGTCTGAGACTCGTCGACAGCG

TCAGTGGCATCGACGAGTTTTTCGAGGAGCTCGCGATCCGCATATGCTACTACCCACGAAGCCCCGGCGG

GTTTGTCCGCTTCGTAACTTCGATACGTAACGCCCTGGGGTTGCCGTGAGGCGCGCGTCCGACGGTCCCG

CTTCTCGCCTCTCTTCTTCCCCCACCCCACCCACCGACCAACGACGGCGTTTGGCCAATACCCTCCTTTT

TTCTTTTTCTCTTCCCCCCCCCCCAAAAAAAACAATAAACAGCTAATTGCGTACGACAAACCATGCGGAA

CTCGCTGTTTTTTTTCTCTGTTTGTTACTTTTTATTGAAACAGACATACGGGGAAAGGGGCCGGAAACCG

AGACGGTGGGGCCGGCGGTCGCATTTTTTTAATGGCTCTGGTGTCGGCCGCGTTTGAGCTTCGTCAACAG

GGCGCTGAGGGCGGCGACGTTCGTCGGGCCGTCGTTGGCCAGCGCGTTGGTCCGGGGGCGGGCGGGCATG

GGCGACAGGCTTAGTCCCGGGTCCGGGGCGCGTGTGGCCCGCCGAGGGGAGAAGAGGGCAGACCCGCCCC

AGTCGTACAGGGGATTTTCCGCCTCGATGTACGGGGAGTCCGGGGCGTCTCCCGGCAGGGCGGCCCCGCC

GGCAAGACGCCGGCGAGGGCAGATGTTTTCGTATACCCGAACCCAGGGGATCTCCTCGTAGACGCGCCCC

CCATCCTCGCCCACCGACTCGTAAATGGAATCTGCGTCCTCGGAGGGGGCGCGGGGGGCGTGGCTTTCGG

CCGGCCAGGCGGCGGCGGCGGTGGTGTCGGCGGCGGGGGTGGCGCCAAGCCCGACGCCCGCGGGCATGGC

GGCGTCATCGTCGGGCAGCAGATACGTGTTTTCCATCTGGTCCGGTTCGGCCTCCGCGTCCGGCCCCCAG

GTCCGCACCGCGTCGTAGACCCCGGCGGCCTCGCGCTGAGCCGCGAGCGGGCGCGCCGCGGCTGCCGGCC

GCTGCTCGGGGGGCGCGGGGTTGCGGGGCGGGAGGCGCGGGGGCGCCCCGGCCATATGCGTGTAATACGT

GGCCGGCCGGCCGGCGCAGGGCTCGGGACCCCGGTCGGCCGCGTCGACGTGCGGGGGCTCGGGGAGGTCC

TCGCGGTGGCGCCTGCACCTCCGAGGGGCCGCGGGGGTCGAGTGGGGGCGAGCCCGGGGGAGCGGCGGGG

GTGCGTTGTCGCGCCGGGTCCGTTGTATCTTGTCCCGGCAGCTCCCGCCGACCGCGCCGCGGCCCCCCGG

TGGGCCGGACGCCGCGAGGCGCAGGATGGACTCGTAGTGGGGCGACGGGGTTCCGCTCCGAAGCAGGTCC

GGGGCCAGGGCGGCCCCGAACCAGGACTTGATGCTGAGTTCCATCCGGGCCCAGCTCGGGGCGGTCATCG

TGGGGAACAGGGGGGCGGCGGTCCTGCAGAAGCGCTCCTGGCTGTCCACCGCCGCCGTAAGGTACTCGTT

GTTCAGGCTGTCGGAGGCCCAGACGACATACCCGGTAAGCGTCGCGTTAATTATATACTGGGCGTGGTGG

TGGACTATGGATAGAACCTCGACGGTCGAGACGATGGCGTCCACGATCCCGTACGTGCCGCCGCTGCGCT

TGCCGGTCTCCCACAGGTGGGCCAGGCGCGTCAGGTGGCCCAGGACGTCGCTGACCGCCGCCCGCAGGGC

CATGCACTGCATCGAGCCCGTGGTGCCGCTGGGCCCGCGGTCCAGGTGGCGCGCAAACGTCTCCGCGGGC

GCCTCCAGACTCCCGCTGAGCGCCACGAACCGGCGATCGGCGGGGCCCAGGCGGCGACACACGTACTTGT

CCGCCGTCCACAGCATCCACGAGGCCCAATGGTACAACACGGAGACGTAGGCCAGGAGCTCGCTCAGCCG

CAGTGCGGTGTCCGTGCTCGGCCGGCTCGGGTCTGCGGGGCGCATAAAGAACATGTACTGCTGGAGCCTG

TGGGCCGCGTCGCGCAACCCCGCCACCGCGGCGGCGTACTTGGCCGCGGCGGCCCCGCTCTTGAACGGGG

CGCGCACCAGCAGCTTCGGGAGCAGGGTGGGCCGCAGCAGCACGTGCAGGCTGGGGTCGCAGTCGCCCGC

CGGGTCGTCGGGGATGTCCAGGCCGCTGGGCACGACCGTCTGGAGGTACTTCCAGTACTGCGCTAGGATG

GCGCGGCTCAGCTGGCCGCCCGACAGCTCCACCTCGCCGAGCGCCTGCTTGGCGGCCGACGCGTAGTGCC

GGATGTAGTCGTAGTGCGGGTCGCTGGCGAGCCCGTCTACGATCAGGCTCTCGGGGACGGTGTTATGGTG

CCGCGCCGCCAGCCGGACGCTGCGATCGGCGCCGGTCAGAAACGCCGGCTGCAGGTCGTCGGCGCGCTGC

CGCAGGACGCCCACGGCCGCGCTGAGGAGCCCCTCCGGGGTGGGGAGCAGACACCCGGCGAAGATGCGCC

GCTCGGGGACGCCCGCGTTGGCGCCGCGGATGAGGTTGGCCGGCGTCAGGCACCGCGCCAGCCGCAGGGA

GCTCGCGCCGCGCGCCCGGCGTTGCATGGCGGAGACCGTTCGGTCGGGGGCCCCGCCGGTCGGAGGTATG

CCGCGTCCCGGGATATAGGGTTGCTTTTTATGGGGAGGCGCCTATGGGCGTGGCGGGCCGCCCAGCCCGG

TCGCGCGCCTCCCGGACACGTGCGCCCGGAGGGCGGCGGTCTCCTCGTCGCCCATGAGCAGTTTCCGAAA

CTGCGCCATGATGTCCACGACGCGGACCCGCGGCCCCAGCACGGACTCGCTATTCAGGGGGGCGGGGGGG

AAGGCCGCCAGGTCTTCGAGCAGGAAGGCGGGGTCTGCCGTCCCGCTCACGGGCGCCCGGGGCGCCGAGG

ACGCGGGGCGAAGGTCCACGTGTTCCGCGGCGGCGCGCACGTCCGCCCAAAATTTGGCGGGGGTGGTCCG

CGCGTACAGGGGCTGGGTCGCGCGGAGGACGCACGCGTAGCGCAGGGGGGTGTACGTGCCCACCTCGGGG

GCCGTCGACCCGCCGTCAAACGCGGCCAGGGCCACGCACGCGACCACCGTGTCGGCCAGGCCCAGCAGCC

GCTGCAGGATGAGCCCCGTCGCCAGCACGGCGCGCGCGGCCGCCGCGTCGTCCCTGCGCCGGCGCGCGTC

CCCGCAGGCCAGGGCGTATTTCAGGGTAACGGTCGCCAGGGCCGTGTGCAGCGCGTACACGGCCGCGCCC

AGCACGGCGTTCAGCCCGCTGGTGGCGAGCAGGCGGCGCGCCGCGGTGTCGCCCAGCGCCTCGTGCTCGG

CCGCCACGACCCCGGGGCTGCCCAGGGGCAGGGCGCGAAACAGCGCCTCCTGCTCCACGTCCGCAAACGC

GGGGTGGGCGGAGTGCGGGTGCAGGCGCGCCCCCACGACCACCGAGAGCCACTGGACCGTCTGCTCCGCC

AGGACCGCCAGCACGTCCAGGACGCGCCCCGCAAACGCGGCCTCCCGCGGGAGCACGCATTTGACGGCGC

CGGGGTTGAAGCGGGCGAGCAGAGCCCCGGTGGCGATGTACGTCATGCGCCCCGCGTAGCGGGCGGCCAC

GCGACAGTCGCGCCCCAGGAGCGCGCGCACCCCGGGCCAGTACAGCAGGGACCCCAGCGAACTGCGAAAG

ACCGCGGCGTCGGGGCCGGGGTGGGGGGGCGCGGCCCCTCCCGCGCTGAGCAGCGGCACGGCGGCGGCCC

CCACGGGCCGCAACGCCGTGAGGCTCGCGAACTGCCGTCGGAGCTCGGCCGCCCTGTCGTCGAGCTCCGA

GCCGCGCCCCTCCGTGTGCAGGCGCGTCCCGCAGACCCACCCGTTGATCGCCACCCGCACGATGGCGTCC

ACCAGAAAACCCATCGCGCGGGAGGGGCTGGTTTTTGCCCGCCGATCCGTCAGGTCGAGGATCGCGTCGC

CCGTGACGTACCAGGCCAGCGCCTCGCCCTGCTGCAGCGTCTGGCGGAAAAACACCTTTGGGTCGGCCGG

GGAGGCAAAGTGCATGACCCCCACGCGCGACAGCCCGAACGCGCTATCCGGACACGGGTAGAACCCGGCC

GGATGTCCCAGGGCCAGGGCCGAGCGCACGGACTCGTCCCACGCGGCGACTCGGGGGGTCAGGCGGTCCA

GGGGGAATGCCGCCTGCAGCTCCGGGCCCGACACGCGGCCCGCGAGAATCTCGACCGTCGCGGAAGGCCG

CGCCCCGGGGCCGTCATCGTGCGCGACGGCGGCGGGGTAGTCGTCCTCCTCGTAGTTGAGCTCGTCCAGG

AACAGCGGCGAGGGCACCACCCGCGAACCGCCCACCCGCCCCAAAACGTCGCGTGGGTCCATCGGGCCCA

GGTAGCCTCCCCGCGGGGCCCGCGTGATGGCGCTGTCCCGGCGTCCGCGAACGGACTGGCTCCTGGCCGT

AACGGACCTGGGGCGCGGAAAGGACGCCCGGCGGGGGGGCGCCGCCGCCCGGGCCTCGGACGCGCGTCGG

GACCCGGGGTGACCGCGGGCCTCCCGGCGACGGCGCGGGGGCGGCTCTTCGCTCGCCATCTCCCCCGCGG

CCTCGACCTCGCTGTCGTCGTCCACGTTAAACACCGCCCGCAGGTACCCCATTAACCCGACTCCACCGCC

CTCGGGCTCGTCCTCCACGGGCGAGTCGGCGCGATGCGCGGACGGGGCATGGGACCGGGTGGAGGCGCGC

CTCCGGCGTACGGCATGCCCGCGCACGGACATGGTGGCCGGAGGCCCGATTTTTTACACACGCCCTCCCC

GCAGACGGACGAGGAAAGGGGTGGTGCGAGGGGGGAGGCCCAAACGGGGAGGTGGGGGGTAGGGGGCGGT

CCCAGGGAGCGGGGGGTAGGAACCGGCACGACGGGAACAGAGAAAACGCGACCGCTCCAACAAGGGTGGG

GGGGTGGGCCTCGTCCCCACGCAGACCCGCGGGCAAATGCGAGAACGGGACCCGCGCGCCTGCCTTTATA

CGCGGACCCCAGCACCACGAGCCGTTCTGTGACGCGAATCTACACGACCGCGGGCTCGTAGGCGCGACTA

ACGCCCAACCCAACGGCACACACCCCCCACCCCGCGCGTAACCCCATTTCTTTCATGGTCCCGTAATAAA

CAGCCAACGCACGCCGCGTATGATGAGTTGCTTGCCAATGTTTATTGCTGTGGTTGCGAACCCTCTATCG

CGATACAGACGGAGGTGAGGCGGGGCGGTGGTGGGGGGGGGGCGCGCCGCCCGGTCGCACATCCTACCCC

CCAAAGTCGTCAATGCCCATGGCATCGGTAAACATCTGTTCAAACTCAAAATCGTCCACGTCCAAAGCCC

CATACGAGACGGGGTCGTGGGTCATTCCCGGGGAGGGGGACTCCACGTCCCCCAGCATCTCCAAGTCGAA

GTCGTCCAGGGCGTCGGCGGGCGTCATATCCACCTCCTCGCCGTCCAGGCGGAGTTCGTCTCCCAGGCTG

ACGTCGGTAATGGGGGCGGTGGTGGACAGTCTGCGGGGGCGTTGTCCCGCGGAGAGAAACGACATGCGCG

GCGCCACCAGCCCGGCCTCCGCAGGAGCGTCATCGTCGTCCGGGAGGTCGAGCAGGCCCTCGATTGTCGA

TCCGTAATTGTTTCTGGTCCGCCCGCGGCTATACGCGTGCTCCCGCATGACGGACTCGCCCTCCGAGGTC

GCGACGCTGGAGTACGAGTCCAACTTGGCCCGGATCAGCAGCATAAAGTACCCAGAGGAGCGGGCCTGGT

TGCCCTGCAGGACGGGCGGGGTCGTGAGGGGCGCCCCGGGTTCCTCCGCCGCCGCACTTCGCACCAGCGG

GAGGTTCAGGTGCTCGCGAATGTGGTTTAGCTCCCGCAGTCGCCGGGCCTCCACGGGAACTCCCCGCACG

GTGAGCGATCCGTTGATAAACATCAGGGGCTGAAACAGACACGCCAACTGGCGCCAGCTCTCCAGGTCGC

AGCAGAGGCCGTCGAACAGATCGGGCCGCATCATCTGCTCGGCGTACGCGGCCCATAGGATCTCGCGGCT

CAGAAAGAGGTATAGATGCAGAAACAGGACGCGCGCCAGGCGCGCGGTCTCGCGGTAGTACCTGTCCGCG

ATCGTGGTGCGCAGCATCTCCCGCAGGTCGCGGTTGCGGCCCCGCATGTGTGCCTGGCGGTGTAGCTGCC

GAACGCTGGCGCGCAGGTACCGGTACAGGGCCGAGCAAAAATTTGCCAACACGGTCCGGTAGCTCTCCTC

CCGCGCCCGCAGCTCACCGCGGAAAAACTGCGCCATGGCCTCGTAGTACGAAGGCAGCTCGTCGCGGGTG

GCGGGCAGGGTGGGGAACGCCACGTCGCCGTGGGCGCGAATGTCGATCGGGGAGCGCTCGGGGACGTGCG

CATCCCCCCAGTCGATCACGTCGCTGGGCAGCGTCGACAGAAACTTGCACTCCCGGTACATGTCGGCGTT

GGTCGGGAACCCAGAGAACAGGTCCTCGTTCCAGGTATCTAGCATGGTACACAGCGCGGGACCCGCGCTG

AAGCCCAGATCGTCGAGGAGACGGTTAAACAGGGCCGCGGGGGGGACGGGCATGGGCGGCGAGGGCATCA

GCTGGGCCTGACTCAGCCGACCGGTGGCGTACAGCGGAGGGGCGGCTGGGGTGTTCTTGGGACCCCCGGC

TGGCCTGGGGGGCGGTGGCGAAACCCCGTCCGCGTCCGCAAACAGATCGTCGACCAACAGGTCCATGGGG

GCGGTTGGGTCCGGGAATAACGATCTCGAGAGGCGAATGAGACGTGCCCGAGCGCCCGGCGGCGGAGAGG

GGGGGAGGGATCCGGGACCCGCGACAGAAAAAGGCCGGGGCCCTCGCGAAGGGAATCGCCGGGGGTGCCG

TGCGTCCCCGAGGACTGACATCTCGCGTCCACCACCCCGCATTTAAGTATCACCCCAGTGCCGCCCCAAA

CCTCGTGACTTCCCCACCGCTCCGGGCGGCCCGTCCCCCGCGCTCGGAAGGGAGGCGTGTCCTTCCTCCC

GCCCCTCCCGCCCCTCCCGCCCCTCCCGCCCCTCCCGCCCCTCCCGCCCCTCCCGCCCCTCCCGCCCCTC

CCGCCCCTCCCGCCCCTCCCGCCCCTCCCGCCCCTCCCGCCCCTCGCCACAAACGCGTGCTGACAGCGAA

GTGGTTAAATCGACCGTGATGCTTTATTGTCTGTCGTCTGAACGCGGTCGGGGTCGCTACTCGAGGGGGC

GGCGGGGACGGGAAGCCGAGCGGGCGGGGGCCCGTGCGGTCGCGGCGGCACGCCCCGCGGGGCGGCCCCG

GGCGGCCGCGGTCGCGTCGACGTCCTGCGCCGCGTCGGGATTCACCAACTCGTTCGCGCGCTGCAGGAGG

TTCTTGCCCTCGCAGACCGTCACGCGAATGGTGGTGAGGTCGAGGAGCTCGTTGAGGTCTTCGTCGGTGT

GCGGCCGCGACATGTCCCACAGCTGTACCGCCGCCAGCCGGGCGTGCGTGGCCGCCAGGCGCCCGACCGC

GGCGCAGAAGACGCGCTTGTTGAACCCGGCCACCCGGGGGGTCCACGGCGCCGTGGGGCTCGGTGGGGCG

GTGCTGAAGTGCAGCTTCTTGGCCAGTCCCTGGGCGGGTGTCTTGGTTCTTCCCGAGGCCGTGGGAGCGG

GGGCGTCTAGGAGCACGGCGGAGTCGGCCTGGGCGGGTCGCCTGCCGCGGGCGGGGTCGGTCGCCGGGGT

CGCGGAGGCCTTAGGCGCCCCGCGCGTCATTTTGGGGGTCCGCGCGGGAGGGGCGTGCGAGCGCCCGCCG

GCGCCCACGGGGCCCCCGGGGGGTGGAGGAGCGCGCGCGGGGCCGGGGCCGTGAGAGCCCGCGACGGACG

CCGAACGACGCGGTCGCGCGGTATCCCGGGACTCGTCGTCGTCCGAAGACGAGTCCCGGTAGAGGGCATA

CCCAGCCTCGTCATAATGGAGAAAGCGAACCTCGCCCCTCGGGCGCGCGCGCATCGGGCCAGCGCCGCGG

CGGAAGTCGTCGCGCGGACTCTCTGGGTCCGCCGGGGAGACCGGGCCATAGTACAGCTCCTCGTGGGTCC

CGCGCGGCGCTTCCCGCGGACACGACTTGACGGAGCGGCGAGAGGTCATGGTCTATCGGAGACACCGGGG

ACGCCCGTGCGGATCACAGGGAAGGCGTCGGCGAAGGAGGCAGAGAGCGTCGGAAGGCGGCGAGGGAGGG

AAAGAGGGAGACCGGCGGGGTACGGGAGAGCAGCGAGGGCCTGCGTAACCCACGGGGGCCGCGGGAGTGG

CTCCCTGCGGGTTGCGGGGGAGAGTTTATAGGAAGTGGATATAACCGCAGGCGACGGGACTAACCAATCC

CCGGGGGGGCAACGGACAGACACGCCCCGAACAGGCCCGACTTCCGCGAGGAAGCAAAGGCCGGGGGCCG

CCCAACGACACGCCCACCCCTTCCCAACAGGGCGGGCTCAGGCTGACCCGGCGGCCAGTGCCCGCTGACA

TATCTGATACACGTGCGCGATCATACATACGCCCATCGAGGTCATGCCTAGATAAAAGGGCACCAGGACC

CCCGGGACGGACACCACACCGGCGCTGTCGCCCCGGCATTGCGCGTCCCCGATAACGCCGCGTGCGCCTG

CCGCGTTCGGCGGCTCCCCGGGCACGCCCGCGACGAGCGCGACGAACAACAGCACCACCCAGCGGCCCAG

TCTTGCGGGTTTCCCCGTCATCGCGGCGATGAGTCAGTGGGGGCCCAGGGCGATCCTTGTCCAGACGGAC

AGCACCAACCGGAATGCCGATGGGGACTGGCAAGCGGCCGTAGCTATTCGCGGGGGCGGAGTCGTTCAAC

TGAACATGGTCAACAAACGCGCCGTGGATTTTACCCCGGCAGAATGCGGGGACTCCGAATGGGCCGTGGG

CCGCGTCTCTCTGGGCCTGCGAATGGCAATGCCGCGGGACTTCTGCGCGATTATTCACGCCCCCGCGGTA

TCCGGCCCCGGGCCCCACGTGATGCTCGGTCTCGTCGACTCGGGCTACCGCGGAACCGTCCTGGCCGTGG

TCGTAGCCCCGAACGGGACGCGCGGGTTTGCCCCCGGGGCCCTCCGGGTCGACGTGACGTTTCTGGACAT

CCGGGCCACCCCCCCGACCCTCACCGAGCCGAGCTCCCTGCACCGGTTTCCGCAGTTGGCGCCGTCCCCG

CTGGCAGGGTTACGAGAAGATCCTTGGTTGGACGGGGCGCTCGCGACCGCCGGGGGGGCGGTGGCCCTGC

CGGCCAGACGGCGCGGGGGATCGCTGGTCTACGCGGGCGAGCTAACGCAGGTGACCACCGAGCACGGCGA

CTGCGTGCACGAGGCGCCCGCCTTTCTGCCAAAGCGCGAGGAGGACGCAGGCTTTGACATTCTCATCCAC

CGAGCCGTGACCGTCCCGGCCAACGGCGCCACGGTCATACAGCCGTCCCTCCGCGTATTGCGCGCGGCCG

ACGGACCAGAGGCCTGCTATGTGCTGGGGCGGTCGTCGCTCAATGCCAGGGGCCTCCTGGTCATGCCTAC

GCGCTGGCCCTCCGGGCACGCCTGTGCGTTTGTTGTATGTAACCTGACCGGAGTCCCGGTGACCCTACAA

GCCGGGTCCAAGGTCGCCCAGCTGCTCGTCGCGGGGACCCACGCCCTCCCCTGGATCCCCCCCGACAACA

TCCACGAGGACGGCGCATTCCGGGCCTACCCCAGAGGGGTTCCGGACGCGACCGCCACCCCCCGAGACCC

GCCGATTTTGGTGTTTACGAACGAGTTTGACGCGGACGCCCCCCCAAGCAAGCGGGGGGCCGGGGGGTTT

GGCTCCACTGGCATCTAGACCGCGCCTCGCGTCGGGCCAGATGGGGCCCCGGTCAATAAAGAGCTCTGTT

TCGCATATGCCCTGGTGTTGGCGGTTTTTTTTTGTTGTCTGTCTGCCCGGCGCTCGGTTGTCCGTTCTGT

CGTCGCTATCACATACGCACAAACACACGGGTAGAGTGGAACCGAAACCGGTCGACGTTTATTCACCACA

CAGAAACACAAGCTAAGCGAGAAGGAGGGGGGCCTCGGTCGACGAGGCCTGGCGTTTGGGGGCGGACGTG

CGATGACGTGGGTCCGGTGTAGGGTCCGCGGGGGGCACGGGCCCGGGGCGAACGGGGGATCTGTCGCCGG

CGTGGGTGACTGGGACCGACGCAACCTCCGGGGCTTGTGCCCTCGTAGGCCCGGGGGGGGCCTCGGTCGC

TCCAAGCCCCGCGGTGCGGGTCCCTCCGGCCAGAGCCGAGGTGGAGAGACCAAGGGCCCGCTCCGCGATC

GCCACGTCCTCCATGACCACGTCGCTCTCGGCCATGCTCCGAATGGCCTGGGAGACGAGCACGTCCGCCG

ACTTGTCCGCGGCCCCCACCGACATGTACATCTGCAGGATGGTGGCCATGCACGTGTCCGCCAGGCGGCG

CATCTTGTCCCGATGCGCCGCAACGGCCCCGTCGATGGTGGAGCCCTCGAGTCCCGGGTGGTGGCGCGCC

AGCCTCTCGAGGTTGACCATGCAGGCGTGGTATGTGCGGGCCAGGGCGCGCGCCTTCACGAGGCGCCGGG

TGTCGTCCAGCGACTCTAGGGCGTCGTCGAGCGTGATGGGGGCGGGCAAAAGCGCATTGACCACCGCCAG

GGCCTCCTGCAGCCGCGGCTCCGCCTCCGAGGGCGGAGCCGCGGCCCGAATCATCTCATATTGTTGTTCC

TCGGGGCGCGTTCCCCAACCGCACAGCACCCCGAGCAGGGACGCCATCCCGGAACACGCGCGCGGCTCTG

CGCCGGCTTTCCCCCACCCCACCCCCTCCGGGTTCGCAGGGGCGATGGGGACGGAAGACTGCGATCACGA

AGGGCGGTCGGTTGCGGCTCCCGTGGAGGTTACGGCGCTGTATGCGACCGACGGGTGCGTTATCACCTCC

TCGCTCGCCCTCCTCACAAACTGCCTGCTGGGGGCCGAGCCGTTGTATATATTCAGCTACGACGCGTACC

GGTCCGATGCGCCCAATGGCCCCACGGGCGCGCCCACCGAACAGGAGAGGTTCGAGGGGAGCCGGGCGCT

CTACCGGGATGCGGGGGGGCTAAATGGCGATTCATTTCGGGTGACCTTTTGTTTATTGGGGACGGAAGTG

GGCGTGACCCACCACCCGAAAGGGCGCACCCGGCCCATGTTTGTGTGCCGCTTCGAGCGAGCGGACGACG

TCGCCGTGCTCCAAGACGCCCTGGGCCGCGGGACCCCATTGCTCCCGGCCCACGTCACAGCAACTCTGGA

CTTGGAGGCGACGTTTGCGCTCCACGCTAACATCATCATGGCTCTCACCGTGGCCATCGTCCACAACGCC

CCCGCCCGCATCGGCAGCGGCAGCACCGCCCCCCTGTATGAGCCCGGCGAATCGATGCGCTCGGTCGTCG

GGCGCATGTCCCTGGGGCAGCGCGGCCTCACCACGCTGTTCGTGCACCACGAGGCGCGCGTGCTGGGGGC

GTACCGCCGGGCGTATTATGGGAGCGCCCAAAGCCCCTTTTGGTTTCTGAGCAAATTCGGCCCGGACGAA

AAGAGCCTGGTGCTGGCCGCTAGGTACTACCTACTCCAGGCTCCGCGCTTGGGGGGCGCCGGAGCCACGT

ACGATCTGCAGGCCGTGAAAGACATCTGCGCGACCTACGCAATCCCCCACGACCCACGCCCCGACACCCT

CAGTGCCGCGTCCTTGACCTCGTTCGCCGCCATCACTCGGTTCTGTTGCACGAGCCAGTACTCCCGCGGG

GCCGCGGCCGCTGGGTTTCCGCTGTATGTGGAGCGCCGCATCGCCGCCGACGTACGCGAGACCGGCGCGC

TGGAGAAGTTCATCGCCCACGATCGCAGCTGCCTGCGCGTGTCCGACCGGGAATTCATTACGTACATCTA

CCTGGCCCACTTTGAGTGCTTCAGCCCCCCGCGCCTGGCCACGCATCTCCGGGCCGTGACCACCCACGAC

CCCAGCCCCGCGGCCAGCACGGAGCAGCCCTCGCCCCTGGGTCGGGAGGCGGTGGAACAGTTCTTCCGGC

ACGTGCGCGCCCAGCTGAACATCCGCGAGTACGTAAAGCAAAACGTCACCCCCAGGGAAACCGCCCTGGC

GGGAGACGCGGCCGCCGCCTACCTGCGCGCGCGCACGTATGCCCCGGCGGCCCTCACGCCCGCCCCCGCG

TACTGCGGGGTCGCAGACTCGTCCACCAAAATGATGGGACGTCTGGCGGAAGCAGAAAGGCTCCTAGTCC

CCCACGGCTGGCCCGCGTTCGCACCAACAACCCCCGGGGACGACGCGGGGGGCGGCACTGCCGCCCCCCA

GACCTGCGGAATCGTCAAGCGCCTCCTCAAGCTGGCCGCCACGGAGCAGCAGGGCACGACGCCCCCGGCG

ATCGCGGCTCTCATGCAGGACGCGTCGGTCCAAACCCCCCTGCCCGTGTACAGGATTACCATGTCCCCGA

CCGGCCAGGCGTTTGCCGCGGCGGCGCGGGACGACTGGGCCCGCGTGACGCGGGACGCGCGCCCGCCGGA

AGCGACCGTGGTCGCGGACGCGGCGGCGGCGCCCGAGCCCGGCGCGCTCGGCCGGCGGCTCACGCGCCGC

ATTTGCGCCCGGGGCCCCGCGCTCCCCCCGGGCGGCCTGGCCGTCGGGGGCCAGATGTACGTGAACCGCA

ACGAGATCTTCAACGCCGCGCTGGCCGTTACGAACATCATCCTGGATCTGGACATCGCCCTGAAGGAGCC

CGTCCCCTTTCCCCGGCTCCACGAGGCCCTGGGTCACTTTAGGCGCGGGGCGCTGGCGGCGGTTCAGCTG

TTGTTTCCCGCGGCCCGCGTAGACCCCGACGCCTATCCCTGTTATTTTTTCAAAAGCGCCTGTCGGCCCC

GCGCGCCGCCCGTCTGTGCGGGCGACGGGCCCTCGGCCGGTGGCGACGACGGCGACGGGGACTGGTTCCC

CGACGCCGGTGGTCCCGGCGACGAGGAGTGGGAGGAGGACACGGACCCCATGGACACGACCCACGGCCCC

CTCCCGGACGACGAGGCCGCGTACCTCGACCTGCTACACGAACAGATACCAGCGGCGACGCCCAGCGAAC

CGGACTCCGTCGTGTGTTCCTGCGCCGACAAGATCGGGCTGCGCGTGTGCCTACCGGTCCCCGCCCCGTA

CGTTGTGCACGGCTCCCTGACGATGCGTGGGGTGGCGAGGGTGATCCAGCAGGCGGTGCTGTTGGACCGC

GACTTCGTGGAGGCCGTAGGGAGCCACGTAAAGAACTTTTTGCTGATCGATACGGGCGTGTACGCCCACG

GCCACAGCCTGCGCTTGCCGTATTTCGCCAAGATCGGCCCCGACGGCTCCGCGTGCGGCCGGTTATTGCC

CGTCTTCGTGATCCCCCCCGCGTGCGAGGACGTTCCGGCGTTCGTCGCCGCGCACGCCGACCCGCGGCGC

TTCCACTTTCACGCCCCGCCCATGTTTTCCGCGGCCCCGCGGGAGATCCGCGTCCTCCACAGCCTGGGCG

GGGACTATGTCAGCTTTTTCGAGAAGAAGGCGTCGCGCAACGCCCTGGAGCACTTTGGGCGACGCGAGAC

CCTGACGGAGGTTCTGGGCCGCTACGATGTGCGGCCCGACGCCGGGGAGACCGTGGAGGGGTTCGCGTCA

GAACTGCTGGGGCGAATAGTCGCGTGCATCGAGGCTCACTTTCCCGAGCACGCGCGGGAATATCAGGCCG

TGTCCGTTCGCCGGGCCGTCATTAAGGACGACTGGGTCCTGCTGCAGCTGATCCCCGGCCGCGGCGCCCT

GAACCAAAGCCTCTCGTGTCTGCGCTTCAAGCACGGCAGGGCAAGTCGCGCGACGGCCCGGACCTTTCTC

GCGCTGAGCGTCGGGACCAACAACCGCCTATGCGCGTCCCTGTGTCAGCAGTGCTTTGCCACTAAATGCG

ATAACAACCGCCTGCACACGCTGTTTACCGTCGATGCGGGCACGCCATGCTCGCGGTCCGCTCCCTCCAG

CACCTCACGACCGTCATCTTCATAACGGCCTACGGCCTCGTGCTCGCGTGGTACATCGTCTTTGGTGCCA

GTCCGCTCCACCGATGTATTTACGCGGTGCGCCCCGCCGGGGCGCACAACGATACCGCCCTCGTGTGGAT

GAAGATAAACCAGACGCTGTTGTTTCTGGGCCCGCCGACCGCCCCCCCCGGCGGGGCATGGACCCCCCAC

GCCCGCGTCTGCTACGCCAATATCATCGAAGGTCGGGCCGTGTCCCTCCCGGCCATCCCCGGCGCCATGA

GCCGCCGGGTCATGAACGTGCACGAGGCCGTAAACTGCTTGGAGGCCCTCTGGGACACCCAGATGCGCCT

GGTGGTCGTCGGTTGGTTTCTGTATCTAGCGTTCGTCGCCCTTCACCAACGACGATGCATGTTCGGCGTC

GTGAGTCCCGCGCACAGCATGGTGGCCCCGGCGACCTATCTTTTGAACTACGCCGGCCGCATAGTGTCGA

GCGTGTTCTTGCAATACCCCTACACGAAAATCACCCGCCTCCTCTGCGAGCTATCCGTTCAACGCCAGAC

CCTGGTGCAGCTGTTCGAGGCGGATCCGGTCACCTTCTTGTACCACCGCCCGGCCATTGGCGTCATCGTG

GGCTGCGAGCTGCTGCTCCGCTTCGTGGCCCTCGGTCTCATCGTCGGCACCGCTCTCATCTCCCGGGGCG

CCTGCGCGATCACACACCCCCTGTTTCTAACAATCACCACCTGGTGTTTCGTGTCCATCATCGCCCTGAC

GGAGCTGTATTTCATCCTGCGGCGGGGCTCGGCCCCCAAAAACGCGGAACCAGCGGCCCCCAGGGGGCGC

TCCAAAGGGTGGTCGGGCGTCTGCGGGCGCTGCTGTTCCATCATCCTCTCCGGTATCGCCGTGCGCCTGT

GCTATATCGCCGTCGTGGCCGGGGTGGTGCTCGTGGCGCTTCGCTACGAACAGGAGATTCAGCGGCGCCT

GTTTGATCTGTGACGTAACGCCTCTTCCGTTGGAAGAGGCGGACCCAGTCGCCCATACAAATTAAATACA

CGACCCGCCTCGGGCCTACGCACCCTCGCACGTCGCATGCAAATTAAAATCGTGCACAGAGCCGATCCGG

CCTCGGGTCTGCTTGCCCCTCCCCCGGCCCAGCACAGGCAGGCTCGTCCGACTTCCGCATACACCCCACC

CTACCGCGTGCTTCCGCACCCCCGCCTACGCGTGTACGCGAAGGCGGACCCAGACCTGCCGTATGCTAAT

TAAATACATAAAACCCACCCTCGGTGTCCGATTGGTTTCTGGGGACGGCGGGGGCGGGGGCGGTGACGCC

CGACGGGGAGGGACAAGGAGGAGTTTCGGAAAGCCGGCCCCGGTCGTGCGGGTATAAGGGCAGCCACCGG

CCCACTGGGCGCTGTGTGCTGCCGTGTGCCGACCCCGGTTGCGCGTCGGTGCCGCTCCTCGATTCGGACC

CGGCCACTCTCTTCCGACACGCGCCCCCTCGGAGGACACCCGCCATCCCAGCCCCGGCGACCTACAACAT

GGCTACCGACATTGATATGCTAATCGACCTAGGATTGGACCTGTCCGACAGCGAGCTCGAGGAGGACGCT

CTGGAGCGGGACGAGGAGGGCCGCCGCGACGACCCCGAGTCCGACAGCAGCGGGGAGTGTTCCTCGTCGG

ACGAGGACATGGAAGACCCCTGCGGAGACGGAGGGGCGGAGGCCATCGACGCGGCGATTCCCAAAGGTCC

CCCGGCCCGCCCCGAGGACGCCGGCACCCCCGAAGCCTCGACGCCTCGCCCGGCAGCGCGGCGGGGAGCC

GACGATCCGCCACCCGCGACCACCGGCGTGTGGTCGCGCCTCGGGACCAGGCGGTCGGCTTCCCCCCGGG

AACCGCACGGGGGGAAGGTGGCCCGCATCCAACCCCCGTCGACCAAGGCACCGCATCCCCGAGGCGGGCG

GCGAGGTCGCCGCCGGGGCCGGGGTCGATACGGCCCCGGCGGCGCCGACTCCACACCAAAACCCCGCCGG

CGCGTCTCCAGAAACGCCCACAACCAAGGGGGTCGCCACCCCGCGTCGGCGCGGACGGACGGCCCCGGCG

CCACCCACGGCGAGGCGCGGCGCGGAGGGGAGCAGCTCGACGTCTCCGGGGGCCCGCGGCCACGAGGCAC

GCGCCAGGCCCCCCCTCCGCTGATGGCGCTGTCCCTGACCCCCCCGCACGCGGACGGCCGCGCCCCGGTC

CCGGAGCGAAAGGCGCCCTCTGCCGACACCATCGACCCCGCCGTTCGGGCGGTTCTGCGATCCATATCCG

AGCGCGCGGCGGTCGAGCGCATCAGCGAAAGCTTTGGACGCAGTGCCCTGGTCATGCAAGACCCCTTTGG

CGGGATGCCGTTTCCCGCCGCGAACAGCCCCTGGGCTCCCGTGCTGGCCACCCAAGCGGGGGGGTTTGAC

GCCGAGACCCGTCGGGTTTCCTGGGAAACCCTGGTCGCTCACGGCCCGAGCCTCTACCGCACATTCGCAG

CCAACCCGCGGGCCGCGTCGACAGCCAAGGCCATGCGCGACTGCGTGCTGCGCCAGGAAAATCTCATCGA

GGCCCTGGCGTCCGCGGATGAGACGCTGGCGTGGTGCAAGATGTGCATTCACCACAATCTGCCGCTCCGC

CCCCAGGACCCTATCATCGGAACGGCGGCCGCCGTGCTGGAAAACCTCGCCACGCGCCTGCGCCCCTTTC

TGCAGTGCTACCTGAAGGCCCGAGGCCTGTGCGGGCTGGACGACCTGTGCTCGCGGCGACGCCTGTCGGA

CATTAAGGATATTGCCTCCTTTGTGTTGGTCATCCTGGCCCGCCTCGCCAACCGCGTCGAGCGCGGCGTG

TCGGAGATCGACTACACGACCGTGGGGGTTGGGGCCGGCGAGACGATGCACTTTTACATCCCGGGGGCCT

GCATGGCGGGTCTCATTGAAATACTGGACACGCACCGCCAGGAGTGTTCCAGTCGCGTGTGCGAGCTGAC

GGCCAGTCACACTATCGCCCCCTTATATGTGCACGGCAAATACTTCTACTGCAACTCCCTATTTTAGGCA

AGAATAAACATATTGACGTCAACCCAAGTGGTTCCGTGTGATGTTCTTGGCGCGCGCGGCGGGTGGGGCG

GAGACTCCGGGGCGATGCCGGCGTGCGCGTGGGAGGAGGGCGATGACCCACCGGATAAATGTGGGGCCCC

GGCCCGGCCCGCTTCATAGCGCGTCCAGGAACTCACGGCAGACGCGTATTCACCGACCCCCCCCTCGCAA

CATGACAACGACGCCCCTCTCGAACCTGTTTTTACGGGCCCCGGACATCACCCACGTCGCCCCCCCGTAC

TGTCTGAATGCCACGTGGCAGGCCGAAAACGCCCTGCACACGACCAAAACGGACCCCGCGTGCCTGGCCG

CGCGGAGTTATTTAGTCCGCGCCTCCTGCTCGACCAGCGGCCCCATCCACTGTTTTTTCTTTGCGGTGTA

CAAGGACTCGCAGCACTCCCTTCCGCTGGTTACCGAGCTCCGCAACTTCGCGGACCTGGTCAACCACCCG

CCCGTCTTGCGCGAACTAGAGGATAAGCGTGGGGGGCGGCTGCGGTGCACGGGCCCATTCAGCTGCGGAA

CCATCAAGGACGTCTCCGGTGCATCCCCCGCGGGGGAATACACGATAAACGGTATCGTGTACCACTGTCA

CTGTCGGTATCCGTTCTCCAAAACCTGCTGGCTCGGGGCATCCGCGGCCCTACAACACCTTCGCTCTATA

AGCTCAAGCGGCACGGCCGCTCGCGCGGCAGAACAGCGACGCCACAAAATCAAAATCAAAATCAAGGTAT

AACCCACCCCCTTCCCTCCGAGTCCGTATGCAACCTCATTAATAAAGAGTGAGAACCAACCAAAACAGAC

GCGGTGTGAGTTTGTGGGTTATAGGAACCCGGTAAATACCACGCGACGAACCAGCATGTGTGTTAACGCA

ACTTTTATTCGTTGTATCGCGGGAGGGGGGAAGCTTACCGCCAAAGGAAGGCCAAGATGATAACGACGAC

CACCGCGACCACCCCAAAAACCGCATGACGACACGTCCCGCCACACCACCCTGGGGCTTGGGGCGTGTCG

GAGCTCGACGCACAGCGGGCCGCGCGTTGGGCCCGGTACAGCTCTCGCGAATTGACAAGCGGGGGTCGCC

ACGTGCGCGAGCTTTGCACGCGGGGTTGGTCGGCCGGCCCCACGGACCCGCCCGGTGGCTCGGTCGGACA

TGCGGCCATGACCATGGCGTAGGTGGGGGGGCGATCCGAGGTCGCCTCTGCGTAAGTAGGGAGGCCCGAC

GGGAGGTCGCCTCCCACGCCAGGGTGGGCCCCAATCATAGTTTCCGGTAGAAACAGGGGGGTCTCCACAA

ACAACCCCCCTGGGCCAAAGCTCCGGCGCCGCGCCCGTCGTTCGGCGCGGCGCCTGGCGCGCCGAGCGGC

CCGCCAGGCGGCGCGGCGCGAGCGGCCACGCTCACACACCTCGCCGTCACCGGAAGAAGCCGGTGAAACA

AGCCCAACCGGCGACGTCCCTGCAGAGTACGGTGGAGGCGAGTCCGTGGGGGTGTCGATATCAATAACGA

CAAACTGGCCCGCGCTCGCGCCGGCCACACTCTCGTATGGGGGCGGGGCGTCAATCACGCTATCATCTCC

GTCATCCCTGCATGCGTGGGCATGCCCAGCCCCCAACGCCATGGTGGGGATTCGCGGCTCAGAAGCCTGC

ATGTCGTGTGGTCGGTCGTAGTCCAACGTGCCTCCCCCACCCACCACACAGCCGGTCCCCACGCCGACCA

CTAGACCGCAGACGTCGCCCAACCGAGGTCCCCGTGCACAGACCGCGCCTTTTATAGCCCCAGGGGTTGC

TAATTAACGCACGCATGCAGACGCAATTTATTTTGCTCCCCCGCGTCCTCCCCTCCCCTGCGCACACGTG

ATAGGTCTTGGGAACCCGAGGGGCGACGCGGGGAAAGCGCGCCCCCGCCCGGCCGCCGCGCGCCCCCGCC

CGGCCGCCGCGCGCCCCCGCCCGGCCGCCGCGCGCCCCCGCCCGGCCGCCGCGCGCCCCCGCCCGGCCGC

CGCGCGCCCCCGCCCGGCCGCCGCGCGCCCCCGCCCGGCCGCCCGCGTCGCGCCGGCGCCCCCTCCCGGC

GCTTCCGGGGTCTTTCCTTCCTTCCCCGCCGCGACCCCGACCCCGCCCCACCGCCCCGCCCGGCAGGGGG

GCCCCGGCGCCGCGCAGAACACACAGACGAACACACGGTGGCGATCTTTTCTTTACTTCGGCGGACCAGC

GAGCCCCGGCCCCGGCCCGCGCCCCGCCGCCACACCCACGGCACCCCCCCCCGCCGCCCACCCCGGGGTC

CACACAGGAGCGCGCGGGCGGCAGAAACGCGGGCGCGGCGGCGGTCGGGGTGGGAGTGGTGGTGGGGGAC

ACGAAAACACACCCACGACACTCTCCCCCCACCCCGACCGCCGCCGCGCCCCACCGGCGGGATCGCGGCG

AGACGCAGCCGGGCCCCCCCCCACCACCCGCCCACCCACCTACCCCGCGCCCGCAGCCTCCGGCAGCACG

CCGACCACCGCCGCCACCCCCCAAACAGCCAAGGCGCGGTGGGGGGCGTGGTGGTGAACGATGGGGGGAA

CACGGGGGGGAGGGGTCCGGGGCGAGGCGGGCGGGCGAAGGAAGGGGGGGTGGTGGCGGCGGCGGTGGAA

AGCGGAAAAACGGAGGATGGAAGGGCAGAAGATGGGGAGTCCCGATCCTCCTCCTGCATCCCCTCGCCTT

CCATTCTCCGGCCCTCCGCGAGTCCCGACGCCCCCCCCCCGCCGCCCGACGAAGGAGACCCAAGCACCGC

AGCCGGAGAGGCCGAGCGGGGAGTGGGCGGCCGGGCGGGAGGATGGCGGAGAGAGAGAGAGAGAGAGAGA

GAGGGGGGGGGGGGGAGAGGGAAAGCAACGGGAAAGAGAGGCGCGCGGAAAAGCAGCAAGAGGGGGGACG

GGGCGAGCCGGGCAGAGTGCGGAGCCCCCGGAGCCCGCGGCCGCAGCCGAGCAGCGCCGCGGGCTCCGGG

GCCGGGCCGGGCCGGCAACGCCCCGCGCCGGCCGCGGCGGAGAGAACCCCTGTGTCATTGTTTACGTGGC

CGCGGGCCAGCAGACGGGCCGCGGGCCAGCAGACGGGCCGCGGCGCCAGCGGCCCACGCCTCCCGCCGCA

TTAGGCCCCCGCGGGCATCCGGCGGCCGGCCCCACGCCCTTCCATTAAACACTCCCACGTTGGGGGGGGG

CGCGCCAGCTGAGTGCTCTGCGGTTGCGGGCGCCGTGCCCGGAGATCCATTAAGCCGCCGGAGAGCCCGA

GCCCCGCCCGCGTGTTGCTGTGGGCATTTCTGCTGCGTCATCCCTGTCTTTATAAAACCGGGGGCGCGGC

AGCAACGAACGCAGGGGCCCGCCGCCGATCGAGAGGGACTCCGGAGAAGGAAGGCTGCTCCGCGCACCGG

CGCGCCCTTCTCCTCTCCCCTCCCTACCTCCCCCTCTCTTCCCCCTTTTTTCCCCCGCCTCCCGTCTTCT

TCCGCGCCTCCGAGGGTCCGCCTCTTGCCTCGGGGACCCCCGGGCGGGCCGGGGCTTGGCCGCCGAGGTG

CGCCCCGGCCGGAGGGGCCCCCGCACCTCGGCGGCCGCCCCCTCCGGCGCCGCGCGTTCGCGAAAGGCGC

GAAAGGGGCCCCCGGAGGCTTTTTTCGATTCCCGGCCGGGGGTCCCGGGTAGCCGCCCGGCGCCGGGCGG

AAGGCGTCCCCCGCCCGGCGGTCCGGCCCGGGCCCCCGGCGGAGCGCGGGGGCCCCGGGGCCCCGGGCCG

CGCCGGCGGCGTTTCCGCGTTCCGTTTCTTCTCCCTCCCGGGCCGCCCCGCTCCCGGGCCCGACCCTCGC

CCCTTCCCTTCTCCTCGTCTTCCCCCGTCCCGCCGCGCCCCTTCCCTCTTCCTTCTCTCTCTCTGTCTCG

CTCTCCTCACATTTCCCCCCCCCCCCCCCGCCGCCGCCGCCCTTTGCCCGCGTCCCACCGAGACGCCGCG

CCGCGTGAGCCGTCCGCCGGGGGACCCAGGCTCCGGGGGGGGGGGGCGCCTGCGTGTGTCTCGTGTGAGA

GAGCGCGCCCCTCGAACGCCGCGCGTTCTCGCAGGTAGGTTTAGGGTCGTACAGGTGAGCTTCTGCTGAG

GCGGCGGGGAGAGGGGGGGGGGGCGGGCGGAAGAGAGAAGAGAGCAGGGGTTGGGGGAGAACTGTTCTTC

CTCCCCCTTTCAAGAAACACGAGGCGGGGGTCCCAGAAAGGGCAGGCAGGTCAGCCGCACCGCCCGCGAG

CCAACCCGTATCCTTTTTTTCTAGGTGTTTTTGTTTTTGTTTCTGTTTTTGTTTGTTTTGTTATTATTTT

CGCGGATCCGGCGTGTTCGGATCCACCCCCCCTTTCTCCTTCCTCTTCCCTTCCACCCACCCCCGTTTCC

CCCCCCCCCGTCGTCGTTCCCGGGGGGGCAGGCGCGGGTCGGGCCCGTACGCCCACCGCCCCCACGCGCC

GGTCACCCCCCCCCAACAACCCCAAAGGCGCGTGCCCGGCCACAGCCGTGGGTGTGGCGCCCGTCCCCTT

CCTCTACCGCGTGGGCGCGGGCGGGGGGGTGGTGGTAGTGGTGGCGGAAGGAAACGGGCCGGGGGCCGGG

GCCGCTAGGGAAAGGTAGGCACGCGCGCGGTGTGTCGACTTGCATGCCCCGCAAAACGCGTCGTGTCGTG

TTGTGTCGTGGTGGGCCGTGTTGTGGTGGGCCGTGTGGTGTGGTGTGGTGTTGCGAACGCGCGAGCCCCC

TCGCCCCGATGGGAGTCTCCCCGCAGCCAGGGTAAGGAGGGGCGGGCGTGGCGGGCAGGTGTGCGGGCGG

GGTGGGGTGAGTGCGGTTGCATGCCTCGGGTCTCCTCTTCCTGCTCCTCCTCCTTTCTCCCAGCCAGGGT

GAGGAGGGGCGGGCGTGGCGGGCAGGTGTGCGGGCGGGGTGGGCGCCGGGGCGGGGGTGGGCACGGGCGT

AAGTGCGGGTGCATGCCTCGGGTCTTCTCTTCTCCCTCCTCCTTCCTCCCACCCGTCCCCGGGGGCAGAG

GGCGTGCATGCGTTGTGATTCAACCGCCCTCGCCCCCGCCCCACTTTCCCCCCTCTCTATCAAAGTTCCC

TGGCCCCTGGCTTCGCGCCGGTGGTGCGGCTGACCCCCCCCCTCCTCCCTCCCCGAGCCAGGCGCCCTCC

CACTCCTGCCCACCACCCCCAGGGTCTGGCCGGCCAGACGTGCGTGCTCTGCACGATCGGGCCCCCCTCC

CTGTCAACACGGACACACTCTTTTTTTACCCGCCAGCCAGCCCGCCCACCCACCAAGACAGGGAGCCAGA

ACGAGGCCGGGCCCCGGCTCTGTTCTATGATAAAGACCAACAGGCCTCGGGGGTGGGGGCGGCTTCTCGT

GCCCGCCCCCCCTCCTCCTCCTCCCTTCCCCCCCATCCCCGGCCCCCCTGCGCGGGGGAGCTGCATCAAA

GGCCAACAACAAAGTGTGTCAAAAGCATCACAAAACTTTATTGTAAAATTTTTATAAATATAAAGTTTTT

TTTTTCCTCAAGTTTTCAACAAGGCCAGAAAGTCCATAACAAAATGCTGGTGTGTGTTGCTGTTCGGGGC

CGTGTCCGTCCCCCCCCCCCACTCCCACCCCCACTTCCTGTCTCCTCCCCGTCTTTCCCCCCCCCCACCT

CCCCCTGCCCCCGAGGCGCCTCGGCCGGTGGTCCGGTGGGGGGCGGCTTCCTTCGGGCAGCAAGCCGAGT

GTTAGCTCCCCCTACTCCCCGTGGCCCGCGGGGGCGTCGCCGGCCGGCGCGGGCGCGCCCTGCTCCCGAG

ACCACGGGTGGCGCGACCGGAGGCCGTGGAAGTCCAGCGCGCCCACCAGGGTGCCCTGGTCAAAGAGCAT

GTTGCCCACCGGGGTCATCCAGAGGCTGTTCCACTCCGACGCGGGGGGCGTCGGGTAGTCGGGGGGCCTC

ACGCAGTTGCGCGCGTGCTCGGGGAGCAGGGTGCGGCGGCTCCACGCGGGGGCCGCGGCCCGCAGCAGGT

CCGCCACGTTCCCCGTCTGGTCCACGAGGACCACGTAGGCCCCTATGTGGCCCGTCTCCATGTCCAGGAC

GGGCAGGCAGTCCCCCGTGACCGTCTTGTTCACGTAAGGCGCCAGGGCCACGACGCTCGAGACCCCCGCG

ATGGGCAGGTAGCGCGTGAGGCCGGGCGCCGGGTCGCGGGCCCCGGGCTCGGGGCCGCCCTCCGCGTGGC

GCGTCTTCCTGGCACACTTCCTCGGCCCCCGCGGCGCAGCAGCGCGGGGGCCGAGGGAGGTTTCTCGTCT

CTCCCCAGCGCCGGACGCGGACGCGACGCTCCCACCAGCCCCGCCCGCAGAGGAAGAGGCGGAGGAGGAG

GAGGCGGAGGAGGAGGAGGCGGAGGAGGAGGAGGCGGAGGAGGAGGAGGCGGAGGAGGAGGAGGCGGAGG

AGGAGGAGGCGGAGGAGGAGGAGGCGGCGGCGACCGCGGCCTGGGACGACGGAGACGCCGACGGGGGCGC

GGCGCCCGCGGACGCCGGGGCGAGCGGCCCGTGGCCGCGGTCGCCCGAGTCCGAGTCCGGGGCCCGGCGC

GGCGCCGCCCTCTTGGCCCCCACCCCCTGGGGGGCGAGGGGCGAGCGCGGGGCGGCGGAGGAAGAGGCGG

AGGACGAGGCCGCGGGGCCCGAGTCCGACCCGCGCCTCTTCCGGGGGCGGGCCGCCGCCCCCTCCGCGGC

GTGGGGGGCGGCACCGGGGGTGTTGGTGCCGCGGGGGACCCCGGGTCCTCCCTCCGCGCCCGGCCCTCCC

GACCCGCGCGCGTCGGTCGCGCCTGCCCGGCCCAGACTCTGTGCTTGGGTGTCGGTCTGAGCCTGGGTCA

TGCGCGACCGGGGCGCGCGGTGCGCGTCCACCGGCACGGCGGGCGGCGCGGGCCCGGCCGCGTCCGCGCT

CGCAGACACCACGGGGGCGGCGGCGGCGCGGGGCGGACTCCGGACGCGCGGGGCGACGGCCGCGCGGGGG

CGCGCGGCGCGCCCCGACGACTGTGGCAGACCTCCCCCCCCGGGGCCCGAGGACACCTGTGCGGAGGAGG

AGGAGACAAAGGAGAGCGGCCCGGGGCCCGCGGGGCGGCGCGGAGACGGCGGGGGAGAGTCGCTGATGAC

TATGGGGGGCTCCTGGGCCGCGCGGGGCTGTCTCGCGGGGGGCGTCCTGCCCTCCGCCGCCGCGGCGTCT

TCGCCCACCCGCCGCGCCTGCGCGCGCCCCCCGCCGGCCGCAGGGGGAAGAGAGGCCACTCTCGGCACGA

CGGCCGCGACGGCAGGGCCGCCCCCAGACCCAGATCCCACCCCCGCCCGCAACGGGGCGCCGCCGCTGCT

GCTGCTCCGCGGGGCGCCAGGGGGCGCCGGTCGGGTCGCGGCGGGCTGGGAGGTTCCGCGGGTCGCCCCC

GCACCGCCGCCCCCGCGCCGGGGCGCTCTTCGGGGGGCGGGCGGGACGTAGTCCACTGCAGAGGGAGACA

GAGACGGGAGCCCCCGGTTAGTGCCCGACCCCCGCCCGACCCCCGCCCGACCCCCGCCCGACCCCCGCCC

GACCCCCGCCCGACCCCCGCCCGACCCCCGCCCGACCCCCGCCCGACCCCCGCCCGACCCCCGCCCGCCC

CCCGCCCGACCCCCGCCCGCCCTCACCGTCGGCCAGGTCATCGTCCTCGTCGTCCGTGCCGGGCCACGGG

GGGGTGGGCGACAGGGCGCGGACCGTGTGTCCCCCCAGCGACAGGGAGCGCGGGGCCGTCCGCGGGTTGC

CCGTCCAGATAAAGTCCACGGCCGTGCCGGCCCGCACGGCCGCCTCGGCCTCCACGCGGGTCCGGGGGTC

GTTCACTATCGGGATGGTGCTGAACGACCCGCTGGCGGTCACGCCCACTATCAGGTACGCCACCGGGGTG

TTGCACAGGGGACACGTGTTGCGCAACGGAATCCAGGTCTTCATGCACGGGATGCAGAAGGGGTGCAGGC

AGGGAAAACTCTGGCAGCGCAGGGGCGGGGCGATCTCGTCCGTGCACACGGCACACACGTCGCCCCCCCC

TCCCGCTTCCGCTTCCTCCTCACCCACGGGCCCACCCCCACAGGATCCCTGCGCGTCGGCGGGCGTGGGG

CTGCCCTGGCGCTCGGCCGGGGGCCGGGCCGGGGGCGTGGCCGCGTCCATCAGGCCCGCCTCGAACATCT

CCGTGTCCGTGCTGCCCGCCTCGGAGGTGGAGTCGCGGTGAAGGTCGTCGTCAGAGATTCCCACCTCGGT

CTCCTCCTCCGAGTCGCTGCTGGCGAGCCACTGCATGTCGTTGAGCATCCCCCAGGCGTGCGGGGCGGCG

GGCTGCTTGACAAAGCAACGGGGGGGATTTAGAGGGCGCGGGGCGTGAGGCGGGACCCCCGCGCCGTGTC

CCCCGTGTCCCTCCCTCACCCCGGCCCCCCGCCCGCTGCTTTTTGTTCGGAAGGGGGGGAGAAAGGGGTC

CGTAACCAAAGGTGGTCTGCGTCCTTTGGATTCCGACCCCTCGTCTCCCCCCCTGTCCCCCGCTCTCGGG

CTCCTCCCTGCCTCCCTCGCCCCCCCAGAGGGTCGGGGGGCGGCGCACGGCCCACGGGGGTCCCCCGACC

GCTTAAGCGGGCCGGGGGTCGGCCCCGTCAAGCGTCCCCGCCCCCGAGCCCACCGCCCGCGACCACCCCC

AACCCGCAGCCGGGTGGTCCGGGGAAAAGGGGGGGCCTGAGACCCGGGGGTCGCCCTCTCACCGTGCCGG

GGGTCTGCCGCGGCGGCCGCTCGGGGCCGGGGTCCGCCCGGGAGCTCGTGCCGGGCCGGGGTTCCATGAG

CCGGGGTAGGGTAGACTCGAGACGGCGGCCCGCGGTCTCTCTCTTGCCGGGTTTTAGTCTCTGTCTCTCC

GGGTCTCCTCCTCCCGCCGGGCCGCCGCTCCGTCGCTCGCAGTGCCGGGGTGCGAATGCGGCCCGACCGT

CACACGGGGCTGCCTTATACCCGGCGCCTATCCACTCCCCCAAAGGGGCGGCATTTACGATTCCCCCAAT

AGCCGCGCGCCCCGGCGGGGGCGGAGGGAGGGAATCCCCCCCTCTCGGGGCGGCCCCGTCCCCGGGGACC

AACCGGGTGTACTCCAAGAACCCCATTAGCATGCGCCGCCCCCCGCCGACGCAGATGGGAGTCCCCCCGG

CGCCCCGCCGGCGCGGCCCTGAGTGGTGCCCGCCCCCGGGGAAAAATTCATTAGCATACTAGGAAGCCCA

GGGGACCAATAGGGGCCGATCAGCCCACCCACCCGGCGGCGCGCGAGGCTCTGCGTGTTCTGCCAAGAAA

GTAATCAGCATAACCCGGAACCCCGAGGGAGTAATTACGCGGGGAGCGAGGGGCCGTCCGAACGTTTTTA

ATTACCATAAGCGGGAATGGCGGCCCGTTAAAAGCTGCTAATTACCGCGAGCGGGAACGCCGGCCCATTA

AAAGTTGCTAATTACCATGCGCGGGGATGGCGGCCGGGACCGCCTATTAAAAGTTTCTAATTACCATACC

GGGAAGCCGGCGCGGGGCGGTCGCCGGGGCGGAGTCCGGGCCCGCGCGGCGGCGCGCGGTTGGCCGGCGC

CGCCCCCTGGGGCGGGCGGAGCGGCGGGGCGGCGCCGGGCCCTCGCGGATATATACGCGGGGCTCCCATC

GTCTCTTCGGAGAGCGGCCTCGCGCAGACCTTCGGAGCTCCGGGGCTCCGCCGGCCGAGGCCGCCCTCGC

CGGTTCAACCCTAGACCGCCCGACGGCCCGGGCCCGCGGCGGCGGAGGACCCGCGCGCCGCCGCCGCCGC

CTCCTCCTCCTCCGCGGGTCCGCCGTCTTCGTGGGCCCGGGCTCGGGCTCGGGCCCGAGCTCGGGCCTCG

GGCTCCAGGCACGGTCCGATGACCGCCTCGGCCGCCGCCACGCGGCGCCGGAACCGGTCGCGGTCGGCCC

GCTCGCGCGCCCAGGACCCCCGTCGGGCCAGGCGCGCGGCCGTCTCCCAGGCCACCAGATGGCGCACCTG

CACGCGCGGCGAGAAGCACACCTGCGGGCGGGGAGACACGGGGGTCGGAGGGGCGTCAGGGGGTCGGAGG

GGCGTCAGGGGGTCGGAGGGGCGTCAGGGGGTCGGAGGGGCGTCAGGGGGTCGGAGGGGCGTCAGGGGGT

CGGAGGGGCGTCAGGGGGTCGGAGGGGAGGCGTACCTTCCCGCGCGGCGCGTCCGCGGGCGGGGACGCGG

GGGGCCGCCGCCGGCGCAGGCTCAGGCGCGCCAGGTACTCCGTCGTGGTGCGCAGCCGTAGCGCCAGGTG

GGGCGGAAGGGGGCGCTGCGGCCCGCGCTCCTTGCGCGGCGGCGGCGGGGGGCAGGCGGCGGCAGGCGCG

GCGTGCGGGGCCTCCGGCGCCTTCCCCCCGCCCTCGCTCGGGGGGCTGTTCGCCCACTCTGCGTCGTCGT

TGCCGGCGTAGTCCGCGTCGTCGCTGTCGTCCGCCTGGGGCACCAGCAGCCAGCGCCGCAGGAGCGAGGA

CGCGGCCGGCGCGCTCTCGACCGCGGTTCCCGAGTCGTACGCAGGGACCATTTGGGAGTCTGCGGTTGGG

AGCGCGCCGGGGCGCGGCACGGCTGGAGCGCCGGGGCGCGGCACGGCTGGAGCGCCGGGGCGCGGCCGGC

GCCGGGGACCCCGGCGGCGGGGACCCCGGCGGCGGGACATGGCGGGCGGCTGGGCTCGGCGTAGGCCCGG

AGCCGGAGCGCGTCGGGGCGGGAGAGTTCACTCGGCACGCATGCACGTGTAACCGCCAGTCCGTGCTTGC

CTAGCGAACTCACCCGTCCCGGCTGGCGTGCGCAGCCCGGGCCGTGTTGCGGGCCCTCTTAAGGGGCGGC

GGCAGGACGGGGACTCCCGCCCCGCCTCTTTTCCCCCGGGGAGTCAACCCCCGGGGGGGGTGTTTTTTGG

GGGGGGGCGCGAAGGCGGGCGGCGGCGGCGGGCGGGCGGCAGGGCAGCCCCGCGCGCCCCCTTCCCCGTC

CCTCCCCCGGAGCCGGCCGCTCCCCCGCGGGCGCCGCCCCTCCCCCCGCGCGCCGCGGGGCTGCCTTCCC

GCGGGCGCCCCCGCGCGGCTTTTTTCCCGCGCCCGCCCCCGCGCGGCAGGACGGGGACTAGCAGGCTGTG

CCGCAGACCACCACACACTCCCAAGCTCCCCGCCCCCCCGAAGACGCCAGTCGCACCACCGCTCGCCCTC

GCAGACCAGACAGTTGCACCAAGCACCCGCCCGCCCGCACACGGTTCCCCGCCACCCCCTCCCTCCCCTC

CATCCCGCCGAGCTCGCGGCAGCCCCTCCCCCCCGCGCGCCACGGGGCTGCGGTCCCGCGGCCGCCTCCC

CCGCGGCCGCCTCCCCCGCGCCCCGCCCCGGGGGCTTCCCCCGCCCCTCCCCCCGCGCCCGCGGCCCCGA

GCTCGCAGCAGCCCCTCCCTCCCGCGCCCCGTGCCTTCCCTCCCGCTCCTGCGGGGGGGCTCGGGCCACC

TGACCTTCGTAACCTGCACTCAGGTCAGAGCCCCAGACCCCCCGCGGGCGCGGGAGACGTGCCGCCCGCC

CGACCCCCGCCCGCCCGACCCCCGCCCGCCCGACCCCCGCCCGCCCGACCCCCGCCCGCCCGACCCCCGC

CCGCCCGACCCCCGCCCGCCCGACCCCCGCCCGCCCGACCCCCGCCCGCCCGACCCCCGCCCGCCCGACC

CCCGCCCGCCCGACCCCCGCCCGCCCGACCCCCGCCCGCCCGACCCCCGCCCGCCCGACCCCCGCCCGCC

CGACCCCCGCCCGCCCGACCCCCGAATAAACCACACAAGGCGGTACGTTTTCGTCTGTCTCGTTCTTTAT

TTCTCACACACGCGCGCGGCCATCGCCGCGTCTGTCTTAAAGGCGCACAGACGCCCGATTCCTTCCCCCT

CTCCCCATCTCCCCCCTCCCCCGCTCCCGGAAGTTTCCCCCCCCGTCACTCCCCAAACAGTCCGTCGTCG

TCGTCCTCCAGCTCCGCGTCCATGTCCACGGGCTCGCGCCTCGGCGGCGTGGCCAGCCCCGCGGCGGTCC

CCACCACCTCCACGCCGCCGCCCGCCGCGGCCAGCACCGTCCCCGCGCGGCCCGCGGCCGACGCCCAGCG

TATCTGCGGGGGCGGGCCCGCGTCCGCGTCGTCGCGCAGCACCAGCGGGGGCGCGTCGCCGTCGGGCTCG

AGCAGCGCCCGCGCGCAGAACTCCCGCCGCGGCCCGCGCAGCTCCGCCGGGCCGCCGCGCACGGCGTCGC

GCCCCAGCGCCACGTAGACGGGCCGCAGCGGCGCGCCCAGGCCCCAGCGCGCGCAGGCGCGGTGCGAGTG

CGCCTCGTCCTCGCAGAAGTCCGGCGCGCCGGGCGCCATGGCGTCGCCCGCGCCCGAGGCGGCGGCCCGG

CCGTCCAGCGCCGGGAGCACGGCGCGGCGGTACTCGCGCGGGGACATGGGCACCAGCGTGTCGGGGCCGA

AGCGCGTGCGCACGCGGTACCGCACGTTGGCCCCGCGGCAGAGGCGCAGCGGCGGCGCGTCGGGGTACAG

GCGCGCGTGCGCGGCCTCCACGCGCGCGAAGACCCCCGGCCCGAACACGCGGCCGGAGGCCAGCACGGTG

CGGCGCAGGTCCCGCGCCGCCGGCCAGCGCACGGCGCACTGCACGGCGGGCAGCACCTCGCAGGCCAGGT

AGGCGTGCTGCCGCGAGACCACGGGCCCGTCGGCGGGCCAGTCCGCGGCGCGCACGGCGTTGACGACGAT

GAGGCGGCGGTCGCAGGCGCCGGCCAGCAGCCCCAGGAACTCCACGGCGCCGGCGAAGGCCAGGTCCCGC

GTGGACAGCAGCAGCACGCCCTGCGCGCCCAGCGCCGAGACGTCGGGGGCGCCGGTCCAGTTGCCCGCCC

AGGCGGCCGTGGCGGGCCCGCAGAGCCGGTTGCCCAGGGCCGCCAGCAGGCAGGACAGCCCGCCGCGCTC

GGCGGACCACTCCGGGGGGGGCCCGCCCCCGGCGCGGCCCGCGGCCAGGTCCTCGCCCGGCAGCGGCGAG

TAGAGGATCACCACGCGCACGTCCTCCGGGTCGGGCACCTGGCGCATCCAGGCCGCCGCGCGGCGCAGCG

GGCCCGAGGCGCGCAGCGGGCCGAAGGCGGCGGGCGCGCCGCCGGGGGGCGGGGCGGCGCAGCGCGCGGC

CAGCGAGGCCAGCGCGCGCGGGTCGAACATGAGGGCCGGGCGCCACGGCGCGGGGAAGAGCGGGTGGTCC

GTGAGCTCGGCCACGGCCCGCGGGGCGCAGTAGGCCTCCAGGGCGGCGGCCGAGGGCGCCGGCGTGTGGC

TGGGCCCCGGCGGCTGGCGGCGCCAGCCGCCCTGCGGGTCGGGGCCCTCGGCGGGCCGGCGGGTCAGCGC

CGCGGGGCGCGGCGGCCGCGGCGGCGGCGTCGGCGGGGCGGGGGGCGCGGCCCCCGCGGGAGGGGCGGCC

GCGGGGCGGGGGGCGTCCGCGCGGCTCTTCTTCGGGGGGCGCGGGGCGCCGCCCGGCGGCGCCCTGGCCG

GGGCGGGGCTCTTGCGCTTGCGCGCCTCCCGCGGCGCGGAGGCGGGCGCGGCGAGCGAGTCGGCCGCGGC

GACGGTGTCGGCCAGCAGGGGGCGCAGGCTCTGGTTCTGGAAGAGCAGGTCCGCGGCGGCGGCGGCGGCG

GAGCTCAGCAGGCGCGGGCTCCGCGGCAGCGCCGGGCCCAGGGCCCCGGCGACCAGGCTCACGGCGCGCA

CGGCGGCCACGGCGGCCTCGCTGCCGCCGGCCACGCGCAGGTCCCCGCGCAGGCGCATCAGCACCAGCGC

GTCGCGCACGAACCGCAGCTCGCGCAGCCAGGCGCGCAGGCGGGGCGCGTCGGCGTGCGGCGGGGCGGCC

GCGCCCGCGGGCCCCGGGCGCGGGGGCGCGGCGGGCCGGGCTCCGGCCAGCCCCGGCACGGCCGCCAGGT

CGCCGTCGAAGCCCTCCGCCAGCGCCTCCAGGATCCCGCGGCAGGCGGCCAGGCACTCCACGGCCACGCG

GCCCGCCTCCGCGCGCCGGCCGCCGCCACCACCGCCGGCGCCGTCGTCGTCGTCGTCGTCGTCGGCCCCG

GCCGGCGCGGAGGCGGGCGCGGCGCTCAGGCGCCCCAGGGCGGCGAGCACCCCCGCGGCGCCGTAGCCGG

CGGGCACCGCGCGCTCGTCGGCCGGCGACGCCGCCGCCGACGGCAACGGGGCGGCGGCGGCGGCGGGCTT

CCCGCGGGCGTCGTCGCCGTCGTGGCGGTTGGCGTCGCCGCCGTCGTCGGGGGTTCGCGCCCCGGTCAGC

GCCGCGTTCTCGCGCGCCAGCAGGGGCGCGTAGGCGCGGCGCAGGCTGGTCAGCAGGAAGCCCTTCTGCG

CGCGGTCGTAGCGGCGGCTCATGGCCACGGCGGCCGCCACGTGCGCCAGGCCCCAGCCGAAGCGGCCCGC

CGCCATGGCGTACCCCAGGTGGGGCACGGCCCGCGCCACGCTGCCGGAGATGAAGGAGCTGCTGTTGCGC

GCCGCGCCCGAGATCCGGAAGCAGGCCTGGTCCAGCGCCACGTCCCCGGGCGCCACGCGCGGGTTCTGGA

GCCACCCCATCGCCTCCGCGTCCGGCGTGTACAGCAGCCGCGTGATCAGGGCGTACTGCTGCGCCGCGTC

GCCCAGCTCGGGCGCCCACACGGGCGCCGGGGCGCCCGAGGCCTCGAACCGGGCCCGCGCCTCCTCCGCC

TCGGGCGCCCCCCAGAGGCCGGGGCGGCTGTCGCCCAGCCCGCCGTACAGCACGCGCCCCGGGGGCGGGG

GGCCGGCCCCGGGCCACGGCTCCCCGCTGACGTACCCGTCGCGGTAGCGCGCGTAGAAGGCGCCGGAGGC

CGCGTCGGCGTCCAGCTCGACCCGCCGGGGCCGCCCGGCCGTGAAGCGGCCCGTGGCGTCGCGGCCGGCC

ACCGCCGCGCGGGCCCGGCGGCGCTCCAGGCGGCCCGCGGTCGCCGCGGGGGTCCGGGCCGGGGCGGGCT

CGGCCCTGGGCGGGCTCGGCCGGGGCGCCGCCCCCGGGGCCCTCGCGGGCACCCCCGCCTCCTCGTCGTC

CGCGCCGAGGGTCCCGCCCGCGGCGTGGTCTGCGGCGCTGGCGGGGGCGCGGGCGGCGTCGTCGTCGTCG

TCGTCGTCAGACGAGGAGGCGGATGCAGACGAGGAGGAGGAGGCGGAGGAGGAGGCGGAGGACGCCGACG

ACGAGGATCCGGATTTTGATGAGTCAGAGGCGGCCGAGCGCCGGCGGGGGGCGCGCCGGCGGCGGTGGTG

GTGGTGGTGGTGGTGTCGGCGGGGCGCCGGGGGTCGCGGCGACAGGCTGGCCATGGGGTCCGGGTACGCC

CCGCGGACCGCGGACGTCGTCTCCGGTCCGCGGACCCAGCGGCCCGCGTCGCGGTCGTCGTCATCGTCGT

CGTCGTCGTCGTCGTCGTTCTCCTCGCCATAATCGGCGCGCATGGAGGGGGTCCGCGGCGGAGAAGGCGA

GCGGGCCGCTTCTTCTTGCGCGCCGTCGCGCTCCGGGGGGGGCGACGGGATCGTGCGAACGGCCTCGTCC

ACCATCGAGGCCAGCAGGGCCAGCTGCCGCGGCGAGACGACGCCGTCCGCGGCAGGCTCGTCGACGGCCT

CCCCGGACGCCGGGGCCGCCTCGTCGGCATCGGCATCGGCGGCGGCGTCGTCGGCCTCGTCTTCGTTCTC

CTCCGGCCCACCGTGCCACCCGAACCCGGGCCGCGCGGCGGGGCGACGGTCCGGGTTCGGGGTGGGCGGC

GGTCCGTCGGCTGGATCCGGAGATCCGGGGCCGCCGGTCGTCTCCGCCGCGGCCCGGAGACGTCCCCCGT

CCTCGTCCGCCATCGCGACCTCGGCCCCGCGGCCCTGCGTCGTCGTCGTCGTCTTCTTCTTCTTCCGCTG

CTCCGCCGACATCGCCTCCGACCGGGGTGTGCGGGGGGGGGGTCTTCTTCTTCTTCTTCAGGGGCGGCAG

TGGGGGGGGGTGGTTGGCAGTCTCTCTCCCCCCCGTGCGGTGCGTGCGTGTGCCTGTGTCTTTTCGCCTC

TCCGCGCCGATCGGGTAGATCCTGGCGGCCGCGTCGGTAGCCGCGCTCCGTGTGGACGATCGCCCCGTCG

CCTGGCTGATATAGTCCTCGGGGCGCGCGGGGCGGGGGGAAAGGAGGAGGACGCGGAGGAGGAGCGATCG

ACGCCGCCGCGCCCCGGCTCGCCGGGGTTCCGCCCCCAGGTGGAACCGCATTATGCGCGGCCCCGCCCCG

ACGCCCGCGCGTCCGCGTCCGTGGCGGCGGCCCGTTGGTCGCGCCGCCGCCGCTCCGCCCGCGCGGCATC

TCATTAGCGCCCGGCGCGGGCGGCTTCCGCTTCCGCCCGCGATGCTAATGAGACCCTCGTCGCGGGCGGG

CTCGCTCCCCTGCCCTTCCGGGTTCGTGGTAATGAGATGCCGGCCCCGCGCTCCCGTTGGCCCCCGCCGG

CCCCAAAGGGGCCGGCGAGGTCGCCCCGTTGGTCCGCGGGCGGCTCCGCCCCAAAGGGGGCGGGGCCGCA

GGGTAAAAGAAGTGAGAACGCGAAGCGTTCGCACTTCGTCCTAATAGTATATATATTATTAGGGCAAAGT

GCGAGCGCTGGCGCCCTGCCCGGGGCCCGCGTCATCCCGCGCTCCGCCCCAAAGGGGGCGGGGCCGCAGG

GTAAAAGAAGTGAGAACGCGAAGCGTTCGCACTTCGTCCTAATAGTATATATATTATTAGGGCAAAGTGC

GAGCACTGGCGCCCTGCCCGGGGCCCGCGTCATCCCGCGGGCTCCGCCCCGAGGCGGGCCCGGACGGGGG

GCGGGCCGTTCCTCGCGCACATAAAGGGCCGGCGTCCCGGTCGCCGCCGCACCAGGGGCACACCGGCTGC

GCGGCGGAGACCGGGACGGCAGCGGCGGCATCGCGAAGGGGGCCACAGCGAGACAGAGACGCCGGCGGCG

AGCGGGGCACCGACGCACCCGGATCGGATCGGATACAGAGACGCGGGCGCATCGGTTCCTTTTCGTTCTG

CCTTTCCCTCCCCCCCCCCCCCCCCCACCCTGTACGTACCGCGAGGACCCATCCACCCACTGCAGCCTTA

TCGCAGGTACGGTGACCCGGGGGGCCGGCCGGGGGGACGGGCGGGGGACGGGGGGACGGGCCGGGGGGAC

GGGCCGGGGGGACGGGCCGGGGGGACGGGCCGGGGGGACGGGCCGGGGGGACGGGCCGGGGGGACGGGCC

GGGGGGACGGGCCGGGGGGACGGGCCGGGGGGCCGGGGGGCCGGGGGGCCGGGGGGCCGGGGGGACGGGG

GGACGGGGGGACGGGGGGACGGGGGGACGGGGGGACGGGGGGACGGGGGGACGGGGGGACGGGGGGACGG

GGGGACGGGGGGACGGGGGGACGGGGGGACGGGGGGACGGGGGGACGGGGGGACGGGCCGGGGGGACGGG

GGGACGGGCCGGGGGGACGGGGGGACGGGCCGGGGGGACGGGGGGACGGGCCGGGGGGACGGGGGGACGG

GCCGGGGGGACGGGGCCCCGATCCCAACATCCGCGCTTTCTCGCAGGCCGGGCGCCGCCTTCGTGGACGG

GACACCGGTGTGGTAACTGGCGACAAGGCGTTGCCACTATGGCAGACATCCCCCCGGACCCGCCCGCGCT

CAACACGACGCCTGCGAATCATGCTCCCCCATCCCCACCCCCGGGTTCACGGAAGCGCAGACGCCCCGTC

CTCCCCAGCTCGTCGGAATCTGAGGGTAAGCCCGACACAGAATCGGAATCCTCCTCGACCGAGTCGTCCG

AGGATGAGGCGGGAGACCTACGCGGCGGGCGCCGTCGCTCCCCGCGGGAGCTCGGGGGGAGGTATTTTTT

GGATCTGTCGGCAGAATCGACCACGGGGACGGAATCGGAGGGAACGGGGCCGTCGGACGACGATGATGAT

GATGCGTCAGACGGCTGGTTGGTTGACACACCCCCCCGCAAATCCAAGCGACCCCGAATCAACCTGCGAT

TAACGAGCTCCCCCGACCGGCGTGCGGGTGTGGTTTTCCCCGAGGTGTGGAGAAGCGACAGACCTATCCG

CGCGGCGCAACCCCAGGCCCCGGCCAGTCTTCCGGGGATCGCGCACGCGCACCGGCGCTCTGCTCGCCAG

GCCCAGATGCGGAGCGGAGCCGCCTGGACGCTTGATCTGCATTACATACGCCAGTGCGTCAACCAGCTCT

TTCGGATCCTGCGTGCCGCCCCGAACCCGCCCGGCAGCGCCAACCGCCTGCGCCACCTGGTGCGAGACTG

CTACCTCATGGGCTACTGCCGGACCCGCCTGGGGCCGCGCACGTGGGGCCGCCTGCTGCAGATCTCGGGC

GGAACCTGGGACGTGCGCCTGCGAAACGCAATCCGGGAGGTCGAGGCGCATTTTGAACCCGCCGCCGAGC

CCGTGTGCGAGCTGCCCTGTCTGAACGCCAGGCGTTACGGCCCCGAGTGTGATGTTGGCAATCTCGAGAC

CAACGGCGGCTCGACGAGCGATGATGAGATATCGGATGCGACGGACTCGGACGATACCCTCGCGTCCCAT

TCCGACACGGAGGGGGGGCCCTCCCCGGCCGGCCGGGAGAACCCGGAATCCGCGTCCGGCGGGGCTATCG

CGGCTCGGCTGGAGTGTGAGTTTGGGACGTTTGACTGGACGTCCGAGGAGGGCTCCCAGCCCTGGCTGTC

CGCGGTGGTCGCCGATACCAGCTCCGCCGAACGCTCTGGCCTACCCGCCCCGGGCGCGTGTCGCGCAACG

GAAGCCCCAGAACGCGAGGACGGGTGCCGAAAAATGCGCTTCCCCGCCGCCTGCCCCTATCCCTGCGGCC

ACACATTTCTCCGGCCATGAGCGCGGGACCCCCAGCCCGGTGTGTTTGCCAAACGAAAAATAAACGCCCT

ACAAGAAAGCTTTTGTGTCTGAGTGTCTGGTTTTTCTGGGGGTGGAGGAAGGAACGACAAAAAAAAGAAA

CAAACGCGACACCGCTCGTACGTGTAATGGGGCCGCAGTGTTTTTTATTAGCATCGGGGGGGGTTAGAGG

TTGGTGATTGGATAGCAAACGTGGGATGACGGAGGCCACTCGTCGCCAACGGCCAGCGGGGGCCCGGGGT

TCTGGGGGTCATCGTCCCCCGTCTGCCAGGAGGGCTCATCGGGAATCTCGGGTCGCCCCATGCACGTAAA

ACACGGGCGCTGCGTGGGGTGGGTCGCCGGATGCGGGCGGGATGATGCGGGGCGGGGTTTGTTGTGAGGA

GCCACGAGGGACCGTAGCCAGCGAAGACAGCTGCGTTCCCGGTCGCCGGGCACCACCACGCCGTATTGGT

ATTCGTATCGGCTAAGGAGATTTTCCAGGGGGTGATTAGGCGCTGCGGGGAACGGGGTCCACGACACGGT

CCGCTCGGGCAAAAACCGATCGGGCAGGGGCCACGGTTCCCCCACCCACGCGTCGTTGGTCTTCGTGGCG

ATGAAGCGAAACCCCAGCCGGGTTTTTTGTGCGTACTCGAAAAACGGCACACACAGGTCCGCCGCCCCGA

CCACCCACAGGTGGTATAGCCGGTGGGGGCCGGGGCGCTCTTGATGCAGGAGCCGAAAACACGCAGGGGC

ATCCAGAATCTCGATGCTTTCCAGGGGGTCGTCCTCCGCAAACAGGCCCGTCGTGGTGTTTGGGGGACAG

CGACAGGAGCGGGTTCGCACGATCGGTCGGGTGAATTTGGGCAAGTCCATCAGAGGCTCGGCCAGCCTGC

GAAGGTTCGCCGGGCGAACCACCACCGGGGTTCCCAGAGGCTCGGAGGCCAGGATCCGGCATTGCCGAAG

CAGAAAACTCCACAGAGCCGGGCTTGCGTCAGCGGAAGTCCGCGGCAGGGCGTTTCGTTGGTCTAGGAGG

GTAACCACACTTACAACAACAACGCCCATGTCGGTATATTAGGCCCGTGGTCCGATCTTCACTCACTCGC

CTGTCTGCGGACCTATGCACGGCGGGACGGCGCGCGGACCCGGGGGGGCTGCTTGCTATCACACGGCCCG

TTCGCACGTTCGATTTTTTCAGCCTTGTTTGGTTGGCTAGGTATCCCGGATAATCTGACGTTCCGGATAT

AGGGGGCGGGGGGTAGTGGGGGGTGTGTCGACAAACTGCCGCTTCTTAAAACACCGGGGCCCGTCGCTCG

GGGTGCTCGTTGGTTGGCACGCGCGACGCGGCAAATGGCCTGTCGTAAGTTCTGTGGGGTCTACCGTAGA

CCCGACAAGAGACAGGAGGCGTCCGTCCCGCCGGAGACAAACACGGCCCCGGCCTTCCCGGCGAGCACCT

TTTATACCCCCGCGGAGGATGCGTACCTGGCCCCCGGGCCCCCGGAAACCATCCACCCTTCCCGCCCACC

GTCCCCCGGCGAGGCTGCGCGCCTGTGTCAGCTGCAGGAGATCTTGGCCCAGATGCACAGCGACGAGGAC

TACCCCATCGTGGACGCCGCGGGTGCGGAGGAGGAAGACGAGGCCGACGATGACGCCCCGGATGACGTGG

CCTACCCGGAGGACTACGCGGAGGGGCGTTTTCTGTCCATGGTTTCGGCCGCCCCCCTGCCCGGAGCCAG

CGGCCATCCTCCTGTTCCGGGCCGCGCAGCCCCCCCCGACGTCCGGACCTGCGACACGGGTAAGGTGGGG

GCCACGGGGTTCACCCCGGAAGAGCTCGACACCATGGACCGGGAGGCACTTCGGGCCATCAGCCGCGGGT

GCAAGCCCCCTTCGACCCTGGCAAAACTGGTGACCGGGCTGGGATTCGCGATCCACGGAGCGCTCATCCC

GGGGTCGGAGGGGTGTGTCTTTGATAGCAGCCACCCGAACTACCCTCATCGGGTAATCGTCAAGGCGGGG

TGGTACGCCAGCACGAGCCACGAGGCGCGGCTGCTGAGACGCCTGAACCACCCCGCGATCCTACCCCTCC

TGGACCTGCACGTCGTTTCTGGGGTCACGTGTCTGGTCCTCCCCAAGTATCACTGCGACCTGTATACCTA

TCTGAGCAAGCGCCCGTCTCCGTTGGGCCACCTACAGATAACCGCGGTCTCCCGGCAGCTCTTGAGCGCC

ATCGACTACGTCCACTGCAAAGGCATCATCCACCGCGATATTAAGACCGAGAACATCTTCATCAACACCC

CCGAGAACATCTGTCTGGGGGACTTTGGGGCGGCGTGCTTTGTGCGCGGGTGTCGATCGAGCCCCTTCCA

TTACGGGATCGCAGGCACCATCGATACAAACGCCCCCGAGGTCCTGGCCGGGGATCCGTACACCCAGGTA

ATCGACATCTGGAGCGCCGGCCTGGTGATCTTTGAGACCGCCGTCCACACCGCGTCCTTGTTCTCGGCCC

CGCGCGACCCCGAAAGGCGGCCGTGCGACAACCAGATCGCGCGCATCATCCGACAGGCCCAGGTACACGT

CGACGAGTTTCCGACGCACGCGGAATCGCGCCTCACCGCGCACTACCGCTCGCGGGCGGCCGGGAACAAT

CGTCCGGCGTGGACCCGACCGGCGTGGACCCGCTACTACAAGATCCACACAGACGTCGAATATCTCATAT

GCAAAGCCCTTACCTTTGACGCGGCGCTCCGCCCAAGCGCCGCGGAGTTGCTGCGCCTGCCGCTATTTCA

CCCTAAGTGACCCCGCTCCCCCCGGGGGGCGTGGAGGGGGGGGCTGGTTGGATGTTTTTGCACAAAAAGA

CGCGGCCCTCGGGCTTTGGTGTTTTTGGCACCTTGCCGCCCGGCGTCATGCACGCCATCGCTCCCAGGTT

GCTTCTTCTTTTTGTTCTTTCTGGTCTTCCGGGGACACGCGGCGGGTCGGGTGTCCCCGGACCAATTAAT

CCCCCCAACAGCGATGTTGTTTTCCCGGGAGGTTCCCCCGTGGCTCAATATTGTTATGCCTATCCCCGGT

TGGACGATCCCGGGCCCTTGGGTTCCGCGGACGCCGGGCGGCAAGACCTGCCCCGGCGCGTCGTCCGTCA

CGAGCCCCTGGGCCGCTCGTTCCTCACGGGGGGGCTGGTTTTGCTGGCGCCGCCGGTACGCGGATTTGGC

GCACCCAACGCAACGTATGCGGCCCGTGTGACGTACTACCGGCTCACCCGCGCCTGCCGTCAGCCCATCC

TCCTTCGGCAGTATGGAGGGTGTCGCGGCGGCGAGCCGCCGTCCCCAAAGACGTGCGGGTCGTACACGTA

CACGTACCAGGGCGGCGGGCCTCCGACCCGGTACGCTCTCGTAAATGCTTCCCTGCTGGTGCCGATCTGG

GACCGCGCCGCGGAGACATTCGAGTACCAGATCGAACTCGGCGGCGAGCTGCACGTGGGTCTGTTGTGGG

TAGAGGTGGGCGGGGAGGGCCCCGGCCCCACCGCCCCCCCACAGGCGGCGCGTGCGGAGGGCGGCCCGTG

CGTCCCCCCGGTCCCCGCGGGCCGCCCGTGGCGCTCGGTGCCCCCGGTATGGTATTCCGCCCCCAACCCC

GGGTTTCGTGGCCTGCGTTTCCGGGAGCGCTGTCTGCCCCCACAGACGCCCGCCGCCCCCAGCGACCTAC

CACGCGTCGCTTTTGCTCCCCAGAGCCTGCTGGTGGGGATTACGGGCCGCACGTTTATTCGGATGGCACG

ACCCACGGAAGACGTCGGGGTCCTGCCGCCCCATTGGGCCCCCGGGGCCCTAGATGACGGTCCGTACGCC

CCCTTCCCACCCCGCCCGCGGTTTCGACGCGCCCTGCGGACAGACCCCGAGGGGGTCGACCCCGACGTTC

GGGCCCCCCGAACCGGGCGGCGCCTCATGGCCTTGACCGAGGACACGTCCTCCGATTCGCCTACGTCCGC

TCCGGAGAAGACGCCCCTCCCTGTGTCGGCCACCGCCATGGCACCCTCAGTCGACCCAAGCGCGGAACCG

ACCGCCCCCGCAACCACTACTCCCCCCGACGAGATGGCCACACAAGCCGCAACGGTCGCCGTTACGCCGG

AGGAAACGGCAGTCGCCTCCCCGCCCGCGACTGCATCCGTGGAGTCGTCGCCACTCCCCGCCGCGGCGGC

GGCAACGCCCGGGGCCGGGCACACGAACACCAGCAGCGCCTCCGCAGCGAAAACGCCCCCCACCACACCA

GCCCCCACGACCCCCCCGCCCACGTCTACCCACGCGACCCCCCGCCCCACGACTCCGGGGCCCCAAACAA

CCCCTCCCGGACCCGCAACCCCGGGTCCGGTGGGCGCCTCCGCCGCGCCCACGGCCGATTCCCCCCTCAC

CGCCTCGCCCCCCGCTACCGCGCCGGGGCCCTCGGCCGCCAACGTTTCGGTCGCCGCGACCACCGCCACG

CCCGGAACCCGGGGCACCGCCCGTACCCCCCCAACGGACCCAAAGACGCACCCACACGGACCCGCGGACG

CTCCCCCCGGCTCGCCAGCCCCCCCACCCCCCGAACATCGCGGCGGACCCGAGGAGTTTGAGGGCGCCGG

GGACGGCGAACCCCCCGAGGACGACGACAGCGCCACCGGCCTCGCCTTCCGAACTCCGAACCCCAACAAA

CCACCCCCCGCGCGCCCCGGGCCCATCCGCCCCACGCTCCCGCCAGGAATTCTTGGGCCGCTCGCCCCCA

ACACGCCTCGCCCCCCCGCCCAAGCTCCCGCTAAGGACATGCCCTCGGGCCCCACACCCCAACACATCCC

CCTGTTCTGGTTCCTAACGGCCTCCCCTGCTCTAGATATCCTCTTTATCATCAGCACCACCATCCACACG

GCGGCGTTCGTTTGTCTGGTCGCCTTGGCAGCACAACTTTGGCGCGGCCGGGCGGGGCGCAGGCGATACG

CGCACCCGAGCGTGCGTTACGTATGTCTGCCACCCGAGCGGGATTAGGGGGTGGGGGTGGGGGGCGAGAA

ACGATGAAGGACGGGAAAGGGAACAGCGACCAAATGTCACGATAAGAACAATAAACCTGTGACGTCAATC

AGATATGTGAGTTTGGTTGTGTTTTGTGGGACTGGGGGCGGGGGGTGGGAGGTATCAGTGGGTGACAGAG

TCTTTTAAAAGACGTGTCCCGGGGCCCTCGAGATGCGCAACTTTTGGCCACACAGAGAAAGGCCCCCAGA

CGAAGTCACCCGGGTCCCCGAACAAAAACAAAAACCTTGACCGCCGCCGGGGGGCGTGCCTGTTGTTTTG

GTCTCAATGGATCGGTATGCCGTTCGGACCTGGGGGATTGTGGGAATCCTCGGGTGTGCTGCTGTTGGGG

CCGCACCCACCGGCCCCGCGTCCGATACAACAAACGCGACCGCACGCCTCCCCACGCACCCCCCACTCAT

CCGTTCCGGGGGCTTTGCCGTCCCCCTCATCGTGGGGGGGCTGTGTCTCATGATTCTGGGGATGGCGTGT

CTACTCGAGGTCCTGCGTCGCCTGGGTCGCGAGTTGGCGAGGTGCTGCCCCCACGCGGGCCAATTTGCCC

CATGATTTTTCGCCTTTCTGGCCTTGCCCCCACCCCATCGCCCCGATTGTGTGTCGGGTGCCCGGGGTAC

AGCAGCTATGGAGCGGTCGGTAATATAACTTTGGTTGTCGCCACACGCCCCGTGCCGGGCATGGGTTGTG

CGGGAAGGACGAAATAATCCGGCGATCCCCAAGCGTACCAACTGGGGGGGGGGGGGGGGGGGAAAAGAAA

CTAAAAACACATCAAGCCCACAACCCATCCCACAATGGGGGTTATGGCGGACCCACCGCACCACCATACT

CCGATTCGACCACATATGCAACCAAATCACCCCCAGAGGGGAGGTTCCATTTTTACGAGGAGGAGGAGTA

TAATAGAGTCTTTGTGTTTAAAACCCGGGGTCGGTGTGGTGTTCGGTCATAAGCTGCATTGCGAACGACT

AGTCGCCGTTTTTCGTGTGCATCGCGTATCACGGCATGGGGCGTTTGACCTCCGGCGTCGGGACGGCGGC

CCTGCTAGTTGTCGCGGTGGGACTCCGCGTCGTCTGCGCCAAATACGCCTTAGCAGACCCCTCGCTTAAG

ATGGCCGATCCCAATCGATTTCGCGGGAAGAACCTTCCGGTTTTGGACCAGCTGACCGACCCCCCCGGGG

TGAAGCGTGTTTACCACATTCAGCCGAGCCTGGAGGACCCGTTCCAGCCCCCCAGCATCCCGATCACTGT

GTACTACGCAGTGCTGGAACGTGCCTGCCGCAGCGTGCTCCTACATGCCCCATCGGAGGCCCCCCAGATC

GTGCGCGGGGCTTCGGACGAGGCCCGAAAGCACACGTACAACCTGACCATCGCCTGGTATCGCATGGGAG

ACAATTGCGCTATCCCCATCACGGTTATGGAATACACCGAGTGCCCCTACAACAAGTCGTTGGGGGTCTG

CCCCATCCGAACGCAGCCCCGCTGGAGCTACTATGACAGCTTTAGCGCCGTCAGCGAGGATAACCTGGGA

TTCCTGATGCACGCCCCCGCCTTCGAGACCGCGGGTACGTACCTGCGGCTAGTGAAGATAAACGACTGGA

CGGAGATCACACAATTTATCCTGGAGCACCGGGCCCGCGCCTCCTGCAAGTACGCTCTCCCCCTGCGCAT

CCCCCCGGCAGCGTGCCTCACCTCGAAGGCCTACCAACAGGGCGTGACGGTCGACAGCATCGGGATGCTA

CCCCGCTTTATCCCCGAAAACCAGCGCACCGTCGCCCTATACAGCTTAAAAATCGCCGGGTGGCACGGCC

CCAAGCCCCCGTACACCAGCACCCTGCTGCCGCCGGAGCTGTCCGACACCACCAACGCCACGCAACCCGA

ACTCGTTCCGGAAGACCCCGAGGACTCGGCCCTCTTAGAGGATCCCGCCGGGACGGTGTCTTCGCAGATC

CCCCCAAACTGGCACATCCCGTCGATCCAGGACGTCGCGCCGCACCACGCCCCCGCCGCCCCCAGCAACC

CGGGCCTGATCATCGGCGCGCTGGCCGGCAGTACCCTGGCGGTGCTGGTCATCGGCGGTATTGCGTTTTG

GGTACGCCGCCGCGCTCAGATGGCCCCCAAGCGCCTACGTCTCCCCCACATCCGGGATGACGACGCGCCC

CCCTCGCACCAGCCATTGTTTTACTAGAGGAGTTTCCCCGCTCCCGTGTACCTCTGGGCCCGTGTGGGAG

GGTGGCTGGGGTATTTGGGTGGGACTTGGACTCCGCATAAAGGGAGTCTCGAAGGAGGGAAACTAGGACA

GTTCATAGGCCGGGAGCGTGGGGCGCGCACCGCTGTCCCGACGATTAGCCACCGCGCCCACAGCCACCTC

GACCCGTCCGATCCCGGTATGCCCGGCCGCTCGCTGCAGGGCCTGGCGATCCTGGGCCTGTGGGTCTGCG

CCACCGGCCTGGTCGTCCGCGGCCCCACGGTCAGTCTGGTCTCAGACTCACTCGTGGATGCCGGGGCCGT

GGGGCCCCAGGGCTTCGTGGAAGAGGACCTGCGTGTTTTCGGGGAGCTTCATTTTGTGGGGGCCCAGGTC

CCCCATACAAACTACTACGACGGCATCATCGAGCTGTTTCACTACCCCCTGGGGAACCACTGCCCCCGCG

TTGTACACGTGGTCACACTGACCGCATGCCCCCGCCGCCCCGCCGTGGCGTTCACCTTGTGTCGCTCGAC

GCACCACGCCCACAGCCCCGCCTATCCGACCCTGGAGCTGGGTCTGGCGCGGCAGCCGCTTCTGCGGGTT

CGAACGGCAACGCGCGACTATGCCGGTCTGTATGTCCTGCGCGTATGGGTCGGCAGCGCGACGAACGCCA

GCCGGTTTGTTTTGGGGGTGGCGCTCTCTGCCAACGGGACGTTTGTGTATAACGGCTCGGACTACGGCTC

CTGCGATCCGGCGCAGCTTCCCTTTTCGGCCCCGCGCCTGGGACCCTCGAGCGTATACACCCCCGGAGCC

TCCCGACCCACCCCTCCACGGACAACGACACCCCCGTCCTCCCCCCGAGACCCGACCCCCGCCCCCGGGG

ACACAGGGACGCCCGCGCCCGCGAGCGGCGAGATAGCCCCGCCCAATTCCACGCGATCGGCCAGCGAATC

GAGACACAGGCTAACCGTAGCCCAGGTAATCCAGATCGCCATACCGGCGTCCATCATCGCCTTTGTGTTT

CTGGGCAGCTGTATCTGCTTCATCCATAGATGCCAGCGCCGATACAGGCGCCCCCGCGGCCAGATTTACA

ACCCCGGGGGCGTTTCCTGCGCGGTCAACGAGGCGGCCATGGCCCGCCTCGGAGCCGAGCTGCGATCCCA

CCCAAACACCCCCCCCAAACCCCGACGCCGTTCGTCGTCGTCCACGACCATGCCTTCCCTAACGTCGATA

GCTGAGGAATCGGAGCCAGGTCCAGTCGTGCTGCTGTCCGTCAGTCCTCGGCCCCGCAGTGGCCCGACGG

CCCCCCAAGAGGTCTAGGTCCAAGCGGGCCGTTCGGCAGGCCCGCCCCACCGCCCCCATCGTGGTTATTT

CCCCCCCCCCCCCCCCAATAAACCGATGTTATTTGCCTATATGCGTGTGTTGGATCCCTTTGTGATCGTT

CGTCATTCCCCGGATGGCATGGGAGGCGGGTAATGGATGGGCGGGGCCCGGGGGGAGGAAAAAGAATAAA

GGGGGTAGTGTCGGAGAGGCCCGCCGCGCATTTAAGGAGTCGCCGCCCCGACTCTGTGTCTTCGGGTGAC

TTGGTGCGCCGCCGTCAGCTAGTCTCCGATCTGCCCCGACCGACGGCTCCTGCCACCCGAACATGGCTCG

CGGGGCCGGGTTGGTGTTTTTTGTTGGAGTTTGGGTCGTATCGTGCCTGGCGGCAGCACCCAGAACGTCC

TGGAAACGGGTAACCTCGGGCGAGGACGTGGTGTTGCTTCCGGCGCCCGCGGAACGCACCCGGGCCCACA

AACTACTGTGGGCCGCGGAACCCCTGGATGCCTGCGGTCCCCTGCGCCCGTCGTGGGTGGCGCTGTGGCC

CCCCCGACGGGTGCTCGAGACGGTCGTGGATGCGGCGTGCATGCGCGCCCCGGAACCGCTCGCCATAGCA

TACAGTCCCCCGTTCCCCGCGGGCGACGAGGGACTGTATTCGGAGTTGGCGTGGCGCGATCGCGTAGCCG

TGGTCAACGAGAGTCTGGTCATCTACGGGGCCCTGGAGACGGACAGCGGTCTGTACACCCTGTCCGTGGT

CGGCCTAAGCGACGAGGCGCGCCAAGTGGCGTCGGTGGTTCTGGTCGTGGAGCCCGCCCCTGTGCCGACC

CCGACCCCCGACGACTACGACGAAGAAGACGACGCGGGCGTGACGAACGCACGCCGGTCAGCGTTCCCCC

CCCAACCCCCCCCCCGTCGTCCCCCCGTCGCCCCCCCGACGCACCCTCGTGTTATCCCCGAGGTGTCCCA

CGTGCGCGGGGTAACGGTCCATATGGAGACCCTGGAGGCCATTCTGTTTGCCCCCGGGGAGACGTTTGGG

ACGAACGTCTCCATCCACGCCATTGCCCACGACGACGGTCCGTACGCCATGGACGTCGTCTGGATGCGGT

TTGACGTGCCGTCCTCGTGCGCCGATATGCGGATCTACGAAGCTTGTCTGTATCACCCGCAGCTTCCAGA

GTGTCTATCTCCGGCCGACGCGCCGTGCGCCGTAAGTTCCTGGGCGTACCGCCTGGCGGTCCGCAGCTAC

GCCGGCTGTTCCAGGACTACGCCCCCGCCGCGATGTTTTGCCGAGGCTCGCATGGAACCGGTCCCGGGGT

TGGCGTGGCTGGCCTCCACCGTCAATCTGGAATTCCAGCACGCCTCCCCCCAGCACGCCGGCCTCTACCT

GTGCGTGGTGTACGTGGACGATCATATCCACGCCTGGGGCCACATGACCATCAGCACCGCGGCGCAGTAC

CGGAACGCGGTGGTGGAACAGCACCTCCCCCAGCGCCAGCCCGAGCCCGTCGAGCCCACCCGCCCGCACG

TGAGAGCCCCCCATCCCGCGCCCTCCGCGCGCGGCCCGCTGCGCCTCGGGGCGGTGCTGGGGGCGGCCCT

GTTGCTGGCCGCCCTCGGGCTGTCCGCGTGGGCGTGCATGACCTGCTGGCGCAGGCGCTCCTGGCGGGCG

GTTAAAAGCCGGGCCTCGGCGACGGGCCCCACTTACATTCGCGTGGCGGACAGCGAGCTGTACGCGGACT

GGAGTTCGGACAGCGAGGGGGAGCGCGACGGGTCCCTGTGGCAGGACCCTCCGGAGAGACCCGACTCTCC

CTCCACAAATGGATCCGGCTTTGAGATCTTATCACCAACGGCTCCGTCTGTATACCCCCATAGCGAGGGG

CGTAAATCTCGCCGCCCGCTCACCACCTTTGGTTCGGGAAGCCCGGGCCGTCGTCACTCCCAGGCCTCCT

ATCCGTCCGTCCTCTGGTAAGGCGTCTTCCGACGACGCGGACGTCGGCGATGAACTGATTGCCATCGCGG

ACGCACGCGGGGACCCGCCAGAGACCCTGCCCCCCGGCGCGGGCGGCGCCGCGCCCGCGTGCCGCAGACC

ACCTCGCGGCGGCTCCCCCGCGGCCTTTCCCGTGGCCCTCCACGCCGTGGACGCCCCCTCCCAATTCGTC

ACCTGGCTCGCCGTGCGCTGGCTGCGGGGGGCGGTGGGTCTCGGGGCCGTCCTGTGCGGGATTGCGTTTT

ACGTGACGTCAATCGCCCGAGGCGCATAAAGGTCCGGCGGCCACCCCGCCGCAGCTCATAAAAATCGTGA

GTCACGGCAACCCCACCTTCGCCTCCGCCCTCCGCCAGCGCCCTTCCGCGTCCGCGATGACCTCCCGGCC

CGCCGACCAAGACTCGGTGCGTTCCAGCGCGTCGGTGCCGCTTTACCCCGCGGCCTCGCCCGTCCCGGCA

GAAGCCTACTACTCGGAAAGCGAAGACGAGGCCGCCAACGACTTCCTCGTGCGCATGGGCCGCCAGCAGT

CGGTCCTAAGGCGCCGACGGCGGCGCACGCGGTGCGTCGGGCTGGTTATCGCCTGTCTCGTCGTGGCCCT

CCTATCTGGAGGGTTCGGGGCACTTTTGGTGTGGCTGCTCCGCTAAATGACGCCTCGATGTATGGCGCCT

TCTTCGCCCCCACCCCTCGCCGCGACCCACGTCCGTATGTTAATTGCAATAAAGTGGTTGATTGTCATTA

CGGTCTACTAGGTTGTCTTTTTTTTTTGGGGGGGGGGGAGGAAATGCAGAAAAGGGTAAGAAATTCTCGG

AATTTCACCCCCGGGGGGGGGCAAGTGCAGTAACCCAGTTCCTCAGTGTTTGGGAAATCTATTGAACTCT

CCCGGCTCCTCCGTGTTAGGGAAGTCTCTTGGGGAAATCTATTGACCTCTCGCCCCCCCCCCCCCAGGAG

GGGGGCAGTGCAGTACCCCAGTTCCTCCGTGCTGGGGAAATCTCTCTGCCGGGTACGGGCTCCAGACGAA

GGACCCATACATTTCCCCATCCGCACCCCACATCTGGCGTTCTAGAGTCACGACGCATTTGCCCCCGTCC

CCGCAGCAACACACAAAGCGATTTCAATTTTCACGATTTTATTATTAATTACACCAACCACCCTGTCCCC

GGGACGTGGTCAGGACCGGGGGTCCGCACCCAAACGCACGAAACAAATGCTGGCAGTGTGCCGAATATAA

CCCCGCGTAGGAACACGTCGACGCGTGCGCCAAACAGCACCAGAAGGCGCATGCCATCAGCAGGTCGTGC

ATATGGCGATGTGTTTGGACGCAGGGCGCAGCCGCGGCGATAAAATTCATGGCGGCCGTCCGCCAGGGCC

ACAGCGGCGAGGACTCCCTGTTGGCCCGAAGCCATTGGGTATGAACCAGCTGCGCCTCCTGTCCGACCCT

GGCTCCCGCCAGCGGGGGCGGTGGGTCGTGGGTGTTGAGAGCACACAGGCGGGACACCTCGATCACCGTC

CGAAAAAAGGCCCGGTGGTCCGCGGGCAGCATCTGCAGGTGCGCCAGGGCCTGGGCGTTGAGAGGGTACA

ACTCGGAGCCGGGGGACTCCGGGGGCCGGTCCGCGCGGTGCCGCGAGTTGGCACGCTTTGGGGCCCGGGT

GTCGGACGCGGGCGCGTTATGGATCCCGACGCGGGGCAGAACGTACGTGCGTTGGCGCGGCGATGAGGGG

TCCGGGCTGCCGAGGGGGGCGTAGGGGACCGGGCTAGGCAAGCCCGCGGGTTGCGCGGGGTTCCCGTGGG

GGTCTAGGCTCCCTGGGCACCCGTGGGGGTCGTGGGGGTCGCGGGTCCCTGGGTATGCGCGGGACCCTGG

GTTCTCTGGGAGATCGTGGAACTCGCGGTTCCCTGGGCTCTCGGGGAACCCGGGGCTCCCTGGGGACACG

TGGTGCCCTGGGAATTCTTGATGGTCGGACGGCTTCAGATGGCTTCGGGATCGAGAGGGCCGCACAGACT

CGTAGTAGACCCGAATCTCCACGTTTCCCCGCCGCCGGATCATGGTCGCCGCCCCGGTGCGGGGGCCCGT

CGGTCGGAAGCGAGTGCCCTTCAAGCGTGTCCGCTCCTCTGGGCTGCATGCCGTCGGATGGGGTGCCTTT

TAAGGAAAGGTCTCGGCTGCCCGCCCCAACCGGGGTTTGGGGGTGGGCCGGGGAAACCCCGGATGCCATG

GGGGGGTCACACCCTAAGCGCCGGCGCGCTGGTTGGGTGGGGGTAGAGGGGAGTCCCCGGTCGACGAGAT

CGTATCAAGGGGCCAGCACGCGATCCTGCCGCTCGTTCGATCTAGCACACCCACGGGTCTGCTGTGTGGG

ATTTCGACTCGCGGGATCCGATCGCACGTCCGGAGGACACAGCAGCGGGAGCTCCGGGTCGGTCACCGCA

GTTCTGGCCGCCTCTCGGTCCTCCCGTTCCCTTTTATGGATCTCCGCGCAGACATCGCCATACGTCCGGT

GTGTGCACCGCGAAGAATCCAGAAACATGTCCGTCGTTTTCAGGGCCCAAGACATGGTGTCCCGTCCACG

AAGGCGGCGCCCGGCCTGCGAGAAAGCGCGGATGTTGGGATCGGGGCCCCGTCCCCCCGGCCCGTCCCCC

CGTCCCCCCGGCCCGTCCCCCCGTCCCCCCGGCCCGTCCCCCCGTCCCCCCGGCCCGTCCCCCCGTCCCC

CCGGCCCGTCCCCCCGTCCCCCCGTCCCCCCGTCCCCCCGTCCCCCCGTCCCCCCGTCCCCCCGTCCCCC

CGTCCCCCCGTCCCCCCGTCCCCCCGTCCCCCCGTCCCCCCGTCCCCCCGTCCCCCCGTCCCCCCGTCCC

CCCGGCCCCCCGGCCCCCCGGCCCCCCGGCCCCCCGGCCCGTCCCCCCGGCCCGTCCCCCCGGCCCGTCC

CCCCGGCCCGTCCCCCCGGCCCGTCCCCCCGGCCCGTCCCCCCGGCCCGTCCCCCCGGCCCGTCCCCCCG

GCCCGTCCCCCCGTCCCCCGCCCGTCCCCCCGGCCGGCCCCCCGGGTCACCGTACCTGCGATAAGGCTGC

AGTGGGTGGATGGGTCCTCGCGGTACGTACAGGGTGGGGGGGGGGGGGGGGGAGGGAAAGGCAGAACGAA

AAGGAACCGATGCGCCCGCGTCTCTGTATCCGATCCGATCCGGGTGCGTCGGTGCCCCGCTCGCCGCCGG

CGTCTCTGTCTCGCTGTGGCCCCCTTCGCGATGCCGCCGCTGCCGTCCCGGTCTCCGCCGCGCAGCCGGT

GTGCCCCTGGTGCGGCGGCGACCGGGACGCCGGCCCTTTATGTGCGCGAGGAACGGCCCGCCCCCCGTCC

GGGCCCGCCTCGGGGCGGAGCCCGCGGGATGACGCGGGCCCCGGGCAGGGCGCCAGTGCTCGCACTTTGC

CCTAATAATATATATACTATTAGGACGAAGTGCGAACGCTTCGCGTTCTCACTTCTTTTACCCTGCGGCC

CCGCCCCCTTTGGGGCGGAGCGCGGGATGACGCGGGCCCCGGGCAGGGCGCCAGCGCTCGCACTTTGCCC

TAATAATATATATACTATTAGGACGAAGTGCGAACGCTTCGCGTTCTCACTTCTTTTACCCTGCGGCCCC

GCCCCCTTTGGGGCGGAGCCGCCCGCGGACCAACGGGGCGACCTCGCCGGCCCCTTTGGGGCCGGCGGGG

GCCAACGGGAGCGCGGGGCCGGCATCTCATTACCACGAACCCGGAAGGGCAGGGGAGCGAGCCCGCCCGC

GACGAGGGTCTCATTAGCATCGCGGGCGGAAGCGGAAGCCGCCCGCGCCGGGCGCTAATGAGATGCCGCG

CGGGCGGAGCGGCGGCGGCGCGACCAACGGGCCGCCGCCACGGACGCGGACGCGCGGGCGTCGGGGCGGG

GCCGCGCATAATGCGGTTCCACCTGGGGGCGGAACCCCGGCGAGCCGGGGCGCGGCGGCGTCGATCGCTC

CTCCTCCGCGTCCTCCTCCTTTCCCCCCGCCCCGCGCGCCCCGAGGACTATATCAGCCAGGCGACGGGGC

GATCGTCCACACGGAGCGCGGCTACCGACGCGGCCGCCAGGATCTACCCGATCGGCGCGGAGAGGCGAAA

AGACACAGGCACACGCACGCACCGCACGGGGGGGAGAGAGACTGCCAACCACCCCCCCCCACTGCCGCCC

CTGAAGAAGAAGAAGAAGACCCCCCCCCCGCACACCCCGGTCGGAGGCGATGTCGGCGGAGCAGCGGAAG

AAGAAGAAGACGACGACGACGACGCAGGGCCGCGGGGCCGAGGTCGCGATGGCGGACGAGGACGGGGGAC

GTCTCCGGGCCGCGGCGGAGACGACCGGCGGCCCCGGATCTCCGGATCCAGCCGACGGACCGCCGCCCAC

CCCGAACCCGGACCGTCGCCCCGCCGCGCGGCCCGGGTTCGGGTGGCACGGTGGGCCGGAGGAGAACGAA

GACGAGGCCGACGACGCCGCCGCCGATGCCGATGCCGACGAGGCGGCCCCGGCGTCCGGGGAGGCCGTCG

ACGAGCCTGCCGCGGACGGCGTCGTCTCGCCGCGGCAGCTGGCCCTGCTGGCCTCGATGGTGGACGAGGC

CGTTCGCACGATCCCGTCGCCCCCCCCGGAGCGCGACGGCGCGCAAGAAGAAGCGGCCCGCTCGCCTTCT

CCGCCGCGGACCCCCTCCATGCGCGCCGATTATGGCGAGGAGAACGACGACGACGACGACGACGACGATG

ACGACGACCGCGACGCGGGCCGCTGGGTCCGCGGACCGGAGACGACGTCCGCGGTCCGCGGGGCGTACCC

GGACCCCATGGCCAGCCTGTCGCCGCGACCCCCGGCGCCCCGCCGACACCACCACCACCACCACCACCGC

CGCCGGCGCGCCCCCCGCCGGCGCTCGGCCGCCTCTGACTCATCAAAATCCGGATCCTCGTCGTCGGCGT

CCTCCGCCTCCTCCTCCGCCTCCTCCTCCTCGTCTGCATCCGCCTCCTCGTCTGACGACGACGACGACGA

CGACGCCGCCCGCGCCCCCGCCAGCGCCGCAGACCACGCCGCGGGCGGGACCCTCGGCGCGGACGACGAG

GAGGCGGGGGTGCCCGCGAGGGCCCCGGGGGCGGCGCCCCGGCCGAGCCCGCCCAGGGCCGAGCCCGCCC

CGGCCCGGACCCCCGCGGCGACCGCGGGCCGCCTGGAGCGCCGCCGGGCCCGCGCGGCGGTGGCCGGCCG

CGACGCCACGGGCCGCTTCACGGCCGGGCGGCCCCGGCGGGTCGAGCTGGACGCCGACGCGGCCTCCGGC

GCCTTCTACGCGCGCTACCGCGACGGGTACGTCAGCGGGGAGCCGTGGCCCGGGGCCGGCCCCCCGCCCC

CGGGGCGCGTGCTGTACGGCGGGCTGGGCGACAGCCGCCCCGGCCTCTGGGGGGCGCCCGAGGCGGAGGA

GGCGCGGGCCCGGTTCGAGGCCTCGGGCGCCCCGGCGCCCGTGTGGGCGCCCGAGCTGGGCGACGCGGCG

CAGCAGTACGCCCTGATCACGCGGCTGCTGTACACGCCGGACGCGGAGGCGATGGGGTGGCTCCAGAACC

CGCGCGTGGCGCCCGGGGACGTGGCGCTGGACCAGGCCTGCTTCCGGATCTCGGGCGCGGCGCGCAACAG

CAGCTCCTTCATCTCCGGCAGCGTGGCGCGGGCCGTGCCCCACCTGGGGTACGCCATGGCGGCGGGCCGC

TTCGGCTGGGGCCTGGCGCACGTGGCGGCCGCCGTGGCCATGAGCCGCCGCTACGACCGCGCGCAGAAGG

GCTTCCTGCTGACCAGCCTGCGCCGCGCCTACGCGCCCCTGCTGGCGCGCGAGAACGCGGCGCTGACCGG

GGCGCGAACCCCCGACGACGGCGGCGACGCCAACCGCCACGACGGCGACGACGCCCGCGGGAAGCCCGCC

GCCGCCGCCGCCCCGTTGCCGTCGGCGGCGGCGTCGCCGGCCGACGAGCGCGCGGTGCCCGCCGGCTACG

GCGCCGCGGGGGTGCTCGCCGCCCTGGGGCGCCTGAGCGCCGCGCCCGCCTCCGCGCCGGCCGGGGCCGA

CGACGACGACGACGACGACGGCGCCGGCGGTGGTGGCGGCGGCCGGCGCGCGGAGGCGGGCCGCGTGGCC

GTGGAGTGCCTGGCCGCCTGCCGCGGGATCCTGGAGGCGCTGGCGGAGGGCTTCGACGGCGACCTGGCGG

CCGTGCCGGGGCTGGCCGGAGCCCGGCCCGCCGCGCCCCCGCGCCCGGGGCCCGCGGGCGCGGCCGCCCC

GCCGCACGCCGACGCGCCCCGCCTGCGCGCCTGGCTGCGCGAGCTGCGGTTCGTGCGCGACGCGCTGGTG

CTGATGCGCCTGCGCGGGGACCTGCGCGTGGCCGGCGGCAGCGAGGCCGCCGTGGCCGCCGTGCGCGCCG

TGAGCCTGGTCGCCGGGGCCCTGGGCCCGGCGCTGCCGCGGAGCCCGCGCCTGCTGAGCTCCGCCGCCGC

CGCCGCCGCGGACCTGCTCTTCCAGAACCAGAGCCTGCGCCCCCTGCTGGCCGACACCGTCGCCGCGGCC

GACTCGCTCGCCGCGCCCGCCTCCGCGCCGCGGGAGGCGCGCAAGCGCAAGAGCCCCGCCCCGGCCAGGG

CGCCGCCGGGCGGCGCCCCGCGCCCCCCGAAGAAGAGCCGCGCGGACGCCCCCCGCCCCGCGGCCGCCCC

TCCCGCGGGGGCCGCGCCCCCCGCCCCGCCGACGCCGCCGCCGCGGCCGCCGCGCCCCGCGGCGCTGACC

CGCCGGCCCGCCGAGGGCCCCGACCCGCAGGGCGGCTGGCGCCGCCAGCCGCCGGGGCCCAGCCACACGC

CGGCGCCCTCGGCCGCCGCCCTGGAGGCCTACTGCGCCCCGCGGGCCGTGGCCGAGCTCACGGACCACCC

GCTCTTCCCCGCGCCGTGGCGCCCGGCCCTCATGTTCGACCCGCGCGCGCTGGCCTCGCTGGCCGCGCGC

TGCGCCGCCCCGCCCCCCGGCGGCGCGCCCGCCGCCTTCGGCCCGCTGCGCGCCTCGGGCCCGCTGCGCC

GCGCGGCGGCCTGGATGCGCCAGGTGCCCGACCCGGAGGACGTGCGCGTGGTGATCCTCTACTCGCCGCT

GCCGGGCGAGGACCTGGCCGCGGGCCGCGCCGGGGGCGGGCCCCCCCCGGAGTGGTCCGCCGAGCGCGGC

GGGCTGTCCTGCCTGCTGGCGGCCCTGGGCAACCGGCTCTGCGGGCCCGCCACGGCCGCCTGGGCGGGCA

ACTGGACCGGCGCCCCCGACGTCTCGGCGCTGGGCGCGCAGGGCGTGCTGCTGCTGTCCACGCGGGACCT

GGCCTTCGCCGGCGCCGTGGAGTTCCTGGGGCTGCTGGCCGGCGCCTGCGACCGCCGCCTCATCGTCGTC

AACGCCGTGCGCGCCGCGGACTGGCCCGCCGACGGGCCCGTGGTCTCGCGGCAGCACGCCTACCTGGCCT

GCGAGGTGCTGCCCGCCGTGCAGTGCGCCGTGCGCTGGCCGGCGGCGCGGGACCTGCGCCGCACCGTGCT

GGCCTCCGGCCGCGTGTTCGGGCCGGGGGTCTTCGCGCGCGTGGAGGCCGCGCACGCGCGCCTGTACCCC

GACGCGCCGCCGCTGCGCCTCTGCCGCGGGGCCAACGTGCGGTACCGCGTGCGCACGCGCTTCGGCCCCG

ACACGCTGGTGCCCATGTCCCCGCGCGAGTACCGCCGCGCCGTGCTCCCGGCGCTGGACGGCCGGGCCGC

CGCCTCGGGCGCGGGCGACGCCATGGCGCCCGGCGCGCCGGACTTCTGCGAGGACGAGGCGCACTCGCAC

CGCGCCTGCGCGCGCTGGGGCCTGGGCGCGCCGCTGCGGCCCGTCTACGTGGCGCTGGGGCGCGACGCCG

TGCGCGGCGGCCCGGCGGAGCTGCGCGGGCCGCGGCGGGAGTTCTGCGCGCGGGCGCTGCTCGAGCCCGA

CGGCGACGCGCCCCCGCTGGTGCTGCGCGACGACGCGGACGCGGGCCCGCCCCCGCAGATACGCTGGGCG

TCGGCCGCGGGCCGCGCGGGGACGGTGCTGGCCGCGGCGGGCGGCGGCGTGGAGGTGGTGGGGACCGCCG

CGGGGCTGGCCACGCCGCCGAGGCGCGAGCCCGTGGACATGGACGCGGAGCTGGAGGACGACGACGACGG

ACTGTTTGGGGAGTGACGGGGGGGGAAACTTCCGGGAGCGGGGGAGGGGGGAGATGGGGAGAGGGGGAAG

GAATCGGGCGTCTGTGCGCCTTTAAGACAGACGCGGCGATGGCCGCGCGCGTGTGTGAGAAATAAAGAAC

GAGACAGACGAAAACGTACCGCCTTGTGTGGTTTATTCGGGGGTCGGGCGGGCGGGGGTCGGGCGGGCGG

GGGTCGGGCGGGCGGGGGTCGGGCGGGCGGGGGTCGGGCGGGCGGGGGTCGGGCGGGCGGGGGTCGGGCG

GGCGGGGGTCGGGCGGGCGGGGGTCGGGCGGGCGGGGGTCGGGCGGGCGGGGGTCGGGCGGGCGGGGGTC

GGGCGGGCGGGGGTCGGGCGGGCGGGGGTCGGGCGGGCGGGGGTCGGGCGGGCGGGGGTCGGGCGGGCGG

CACGTCTCCCGCGCCCGCGGGGGGTCTGGGGCTCTGACCTGAGTGCAGGTTACGAAGGTCAGGTGGCCCG

AGCCCCCCCGCAGGAGCGGGAGGGAAGGCACGGGGCGCGGGAGGGAGGGGCTGCTGCGAGCTCGGGGCCG

CGGGCGCGGGGGGAGGGGCGGGGGAAGCCCCCGGGGCGGGGCGCGGGGGAGGCGGCCGCGGGGGAGGCGG

CCGCGGGACCGCAGCCCCGTGGCGCGCGGGGGGGAGGGGCTGCCGCGAGCTCGGCGGGATGGAGGGGAGG

GAGGGGGTGGCGGGGAACCGTGTGCGGGCGGGCGGGTGCTTGGTGCAACTGTCTGGTCTGCGAGGGCGAG

CGGTGGTGCGACTGGCGTCTTCGGGGGGGCGGGGAGCTTGGGAGTGTGTGGTGGTCTGCGGCACAGCCTG

CTAGTCCCCGTCCTGCCGCGCGGGGGCGGGCGCGGGAAAAAAGCCGCGCGGGGGCGCCCGCGGGAAGGCA

GCCCCGCGGCGCGCGGGGGGAGGGGCGGCGCCCGCGGGGGAGCGGCCGGCTCCGGGGGAGGGACGGGGAA

GGGGGCGCGCGGGGCTGCCCTGCCGCCCGCCCGCCGCCGCCGCCCGCCTTCGCGCCCCCCCCCAAAAAAC

ACCCCCCCCGGGGGTTGACTCCCCGGGGGAAAAGAGGCGGGGCGGG
