# Supplementary material for: Identity of zinc finger nucleases with specificity to herpes simplex virus type II genomic DNA: novel HSV-2 vaccine/therapy precursors
Source: Theor Biol Med Model. 2011 Jun 24;8:23. doi: 10.1186/1742-4682-8-23 (PMC3138452; doi:10.1186/1742-4682-8-23)
Supplement: Additional file 2 — A detailed list of some of the over 28,000 Multi-Zif assembly as specific to the HSV-2 genome. This file lists the Multi-Zif assembly specific to the first 1, 478 nucleotide sequences within the genomic context of HSV-2 from bp 1 to 8,174. [file 1742-4682-8-23-S2.PDF]

Print

Zinc Finger Site Type: Array
Zinc Finger Engineering Method: CoDA
Sequence Name :
Sequence Length:154748
Nucleotide Sequence
:nAGTCCCCGTCCTGCCGCGCGGGGGCGGGCGCGGGAAAAAAGCCGCGCGGGGGCGCCCGCGGGAAGGCAGCCCCGCGGCGCGCGGGGGGAGGGGCGGCGCCCGCGGGGGAGCGGCCGGCTCCGGGGGAGGGACGG[illegible]
Selected Module Sets:
Selected Module Count: 3
Ignore Asp Overlap: True

The results below are zinc finger arrays that can be constructed using CoDA. Note that other methods (including modular assembly and OPEN) can also potentially be used to target the input sequence of interest.”

Sort By: Position

```
ZFA-unknown-1
11 gGACGGGGACt 1
11 cCTGCCCCTGa 1

ZFA-unknown-2
18 cGCGGCAGGAc 8
18 gCGCCGTCCTg 8

ZFA-unknown-3
17 cGCGGGGGCGg 27
17 gCGCCCCCGCc 27

ZFA-unknown-4
19 cGGGGGCGGGc 29
19 gCCCCCGCCCg 29

ZFA-unknown-5
20 gGGGGCGGGCg 30
20 cCCCCGCCCGc 30

ZFA-unknown-6
23 gGCGGGCGCGg 33
23 cCGCCCGCGCc 33

ZFA-unknown-7
26 gGGCGCGGGAa 36
26 cCCGCGCCCTt 36

ZFA-unknown-8
45 cGCGGGGGCGc 55
45 gCGCCCCCGCg 55

ZFA-unknown-9
47 cGGGGGCGCCc 57
47 gCCCCCGCGGg 57

ZFA-unknown-10
60 cGCGGGCGCCc 50
60 gCGCCCGCGGg 50

ZFA-unknown-11
57 cGCGGGAAGGc 67
57 gCGCCCTTCCg 67

ZFA-unknown-12
74 cGGGGCTGCCt 64
74 gCCCCGACGGa 64
```

ZFA-unknown-13
79 cGCCGCGGGGc 69
79 gCGGCGCCCCg 69

ZFA-unknown-14
80 cGCGGGGGGAg 90
80 gCGCCCCCCTc 90

ZFA-unknown-15
83 gGGGGGAGGGg 93
83 cCCCCCTCCCc 93

ZFA-unknown-16
84 gGGGGAGGGGc 94
84 cCCCCTCCCCg 94

ZFA-unknown-17
86 gGGAGGGGCGg 96
86 cCCTCCCCGCc 96

ZFA-unknown-18
89 aGGGGCGGCGc 99
89 tCCCCGCCGCg 99

ZFA-unknown-19
101 gGGCGCCGCCc 91
101 cCCGCGGCGGg 91

ZFA-unknown-20
104 cGCGGGCGCCg 94
104 gCGCCCGCGGc 94

ZFA-unknown-21
101 cGCGGGGGAGc 111
101 gCGCCCCCTCg 111

ZFA-unknown-22
103 cGGGGGAGCGg 113
103 gCCCCCTCGCc 113

ZFA-unknown-23
106 gGGAGCGGCCg 116
106 cCCTCGCCGGc 116

ZFA-unknown-24
109 aGCGGCCGGCt 119
109 tCGCCGGCCGa 119

ZFA-unknown-25
122 cGGAGCCGGCc 112
122 gCCTCGGCCGg 112

ZFA-unknown-26
121 cGGGGGAGGGa 131
121 gCCCCCTCCCt 131

ZFA-unknown-27
122 gGGGGAGGGAc 132
122 cCCCCTCCCTg 132

ZFA-unknown-28
124 gGGAGGGACGg 134
124 cCCTCCCTGCc 134

ZFA-unknown-29
129 gGACGGGGAAg 139

```
129 cCTGCCCCTTc 139

ZFA-unknown-30
132 cGGGGAAGGGg 142
132 gCCCCTTCCCc 142

ZFA-unknown-31
135 gGAAGGGGGCg 145
135 cCTTCCCCCGc 145

ZFA-unknown-32
138 aGGGGGCGCGc 148
138 tCCCCCGCGCg 148

ZFA-unknown-33
148 cGGGGCTGCCc 158
148 gCCCCGACGGg 158

ZFA-unknown-34
165 gGCGGCAGGGc 155
165 cCGCCGTCCCg 155

ZFA-unknown-35
169 gGCGGGCGGCa 159
169 cCGCCCGCCGt 159

ZFA-unknown-36
173 gGCGGGCGGGc 163
173 cCGCCCGCCCg 163

ZFA-unknown-37
176 gGCGGCGGGCg 166
176 cCGCCGCCCGc 166

ZFA-unknown-38
179 gGCGGCGGCGg 169
179 cCGCCGCCGCc 169

ZFA-unknown-39
170 cGCCGCCGCCg 180
170 gCGGCGGCGGc 180

ZFA-unknown-40
182 gGCGGCGGCGg 172
182 cCGCCGCCGCc 172

ZFA-unknown-41
173 cGCCGCCGCCc 183
173 gCGGCGGCGGg 183

ZFA-unknown-42
186 gGCGGGCGGCg 176
186 cCGCCCGCCGc 176

ZFA-unknown-43
190 cGAAGGCGGGc 180
190 gCTTCCGCCCg 180

ZFA-unknown-44
198 gGGGGGCGCGa 188
198 cCCCCCGCGCt 188

ZFA-unknown-45
200 gGGGGGGGCGc 190
200 cCCCCCCCGCg 190
```

ZFA-unknown-46
201 gGGGGGGGGCg 191
201 cCCCCCCCCGc 191

ZFA-unknown-47
202 tGGGGGGGGGc 192
202 aCCCCCCCCCg 192

ZFA-unknown-48
203 tTGGGGGGGGg 193
203 aACCCCCCCCc 193

ZFA-unknown-49
215 gGGGGGTGTTt 205
215 cCCCCCACAAa 205

ZFA-unknown-50
216 gGGGGGGTGTt 206
216 cCCCCCCACAa 206

ZFA-unknown-51
217 gGGGGGGGTGt 207
217 cCCCCCCCACa 207

ZFA-unknown-52
218 cGGGGGGGGTg 208
218 gCCCCCCCCAc 208

ZFA-unknown-53
218 gGGGGTTGACt 228
218 cCCCCAACTGa 228

ZFA-unknown-54
241 aGAGGCGGGGc 251
241 tCTCCGCCCCg 251

ZFA-unknown-55
243 aGGCGGGGCGg 253
243 tCCGCCCCGCc 253

ZFA-unknown-56
246 cGGGGCGGGAg 256
246 gCCCCGCCCTc 256

ZFA-unknown-57
249 gGCGGGAGTCc 259
249 cCGCCCTCAGg 259

ZFA-unknown-58
265 gGACGGGGACt 255
265 cCTGCCCCTGa 255

ZFA-unknown-59
272 gGCGGCAGGAc 262
272 cCGCCGTCCTg 262

ZFA-unknown-60
275 gGCGGCGGCAg 265
275 cCGCCGCCGTc 265

ZFA-unknown-61
266 tGCCGCCGCCc 276
266 aCGGCGGCGGg 276

ZFA-unknown-62

278 aGGGGCGGCGg 268
278 tCCCCGCCGCc 268

ZFA-unknown-63
281 tTAAGGGGCGg 271
281 aATTCCCCGCc 271

ZFA-unknown-64
278 tTAAGAGGGCc 288
278 aATTCTCCCGg 288

ZFA-unknown-65
295 tGTTGCGGGCc 285
295 aCAACGCCCGg 285

ZFA-unknown-66
316 aGCCGGGACGg 326
316 tCGGCCCTGCc 326

ZFA-unknown-67
393 gGCGGGAGAGt 383
393 cCGCCCTCTCa 383

ZFA-unknown-68
396 cGGGGCGGGAg 386
396 gCCCCGCCCTc 386

ZFA-unknown-69
399 cGTCGGGGCGg 389
399 gCAGCCCCGCc 389

ZFA-unknown-70
409 aGCCGGAGCGc 399
409 tCGGCCTCGCg 399

ZFA-unknown-71
412 cGGAGCCGGAg 402
412 gCCTCGGCCTc 402

ZFA-unknown-72
423 cGGCGTAGGCc 413
423 gCCGCATCCGg 413

ZFA-unknown-73
433 cGGCTGGGCTc 423
433 gCCGACCCGAg 423

ZFA-unknown-74
435 gGCGGCTGGGc 425
435 cCGCCGACCCg 425

ZFA-unknown-75
439 gGCGGGCGGCt 429
439 cCGCCCGCCGa 429

ZFA-unknown-76
452 gGCGGCGGGAc 442
452 cCGCCGCCCTg 442

ZFA-unknown-77
446 cGCCGCCGGGg 456
446 gCGGCGGCCCc 456

ZFA-unknown-78
449 cGCCGGGGTCc 459
449 gCGGCCCCAGg 459

ZFA-unknown-79
465 cGGCGGGGACc 455
465 gCCGCCCCTGg 455

ZFA-unknown-80
467 gGCGGCGGGGa 457
467 cCGCCGCCCCt 457

ZFA-unknown-81
461 cGCCGCCGGGg 471
461 gCGGCGGCCCc 471

ZFA-unknown-82
464 cGCCGGGGTCc 474
464 gCGGCCCCAGg 474

ZFA-unknown-83
480 cGCCGGGGACc 470
480 gCGGCCCCTGg 470

ZFA-unknown-84
483 cGGCGCCGGGg 473
483 gCCGCGGCCCc 473

ZFA-unknown-86
486 gGCCGGCGCCg 476
486 cCGGCCGCGGc 476

ZFA-unknown-85
476 cGGCGCCGGCc 486
476 gCCGCGGCCGg 486

ZFA-unknown-87
489 cGCGGCCGGCg 479
489 gCGCCGGCCGc 479

ZFA-unknown-88
492 gGGCGCGGCCg 482
492 cCCGCGCCGGc 482

ZFA-unknown-89
497 cGCCGGGGCGc 487
497 gCGGCCCCGCg 487

ZFA-unknown-90
505 gGCTGGAGCGc 495
505 cCGACCTCGCg 495

ZFA-unknown-91
516 gGGCGCGGCAc 506
516 cCCGCGCCGTg 506

ZFA-unknown-92
521 cGCCGGGGCGc 511
521 gCGGCCCCGCg 511

ZFA-unknown-93
529 gGCTGGAGCGc 519
529 cCGACCTCGCg 519

ZFA-unknown-94
540 gGGCGCGGCAc 530
540 cCCGCGCCGTg 530

ZFA-unknown-95
545 cGCCGGGGCGc 535
545 gCGGCCCCGCg 535

ZFA-unknown-96
559 tGCGGTTGGGa 549
559 aCGCCAACCCt 549

ZFA-unknown-97
566 gGGAGTCTGCg 556
566 cCCTCAGACGc 556

ZFA-unknown-98
568 tTGGGAGTCTg 558
568 aACCCTCAGAc 558

ZFA-unknown-99
595 cGCGGTCGAGa 605
595 gCGCCAGCTCt 605

ZFA-unknown-100
616 gGCCGGCGCGc 606
616 cCGGCCGCGCg 606

ZFA-unknown-101
619 cGCGGCCGGCg 609
619 gCGCCGGCCGc 609

ZFA-unknown-102
622 gGACGCGGCCg 612
622 cCTGCGCCGGc 612

ZFA-unknown-103
625 cGAGGACGCGg 615
625 gCTCCTGCGCc 615

ZFA-unknown-104
630 aGGAGCGAGGa 620
630 tCCTCGCTCCt 620

ZFA-unknown-105
633 cGCAGGAGCGa 623
633 gCGTCCTCGCt 623

ZFA-unknown-106
636 cGCCGCAGGAg 626
636 gCGGCGTCCTc 626

ZFA-unknown-107
632 cGGCGCTGGCt 642
632 gCCGCGACCGa 642

ZFA-unknown-108
639 gGCTGCTGGTg 649
639 cCGACGACCAc 649

ZFA-unknown-109
642 tGCTGGTGCCc 652
642 aCGACCACGGg 652

ZFA-unknown-110
655 gGCGGACGACa 665
655 cCGCCTGCTGt 665

ZFA-unknown-111
668 cGCTGTCGTCc 658

```
668 gCGACAGCAGg 658

ZFA-unknown-112
671 cGTCGCTGTCg 661
671 gCAGCGACAGc 661

ZFA-unknown-113
674 cGTCGTCGCTg 664
674 gCAGCAGCGAc 664

ZFA-unknown-114
667 cGACGACGCGg 677
667 gCTGCTGCGCc 677

ZFA-unknown-115
679 gTCCGCGTCGt 669
679 cAGGCGCAGCa 669

ZFA-unknown-116
670 cGACGCGGACt 680
670 gCTGCGCCTGa 680

ZFA-unknown-117
686 cGGCGTAGTCc 676
686 gCCGCATCAGg 676

ZFA-unknown-118
689 tGCCGGCGTAg 679
689 aCGGCCGCATc 679

ZFA-unknown-119
692 cGTTGCCGGCg 682
692 gCAACGGCCGc 682

ZFA-unknown-120
695 cGTCGTTGCCg 685
695 gCAGCAACGGc 685

ZFA-unknown-122
701 cTGCGTCGTCg 691
701 gACGCAGCAGc 691

ZFA-unknown-121
691 cGACGACGCAg 701
691 gCTGCTGCGTc 701

ZFA-unknown-123
694 cGACGCAGAGt 704
694 gCTGCGTCTCa 704

ZFA-unknown-124
702 aGTGGGCGAAc 712
702 tCACCCGCTTg 712

ZFA-unknown-125
703 gTGGGCGAACa 713
703 cACCCGCTTGt 713

ZFA-unknown-126
719 gGGGGCTGTTc 709
719 cCCCCGACAAg 709

ZFA-unknown-127
723 cTCGGGGGGCt 713
723 gAGCCCCCCGa 713
```

ZFA-unknown-128
724 cGAGGGCGGGg 734
724 gCTCCCGCCCc 734

ZFA-unknown-129
727 gGGCGGGGGGa 737
727 cCCGCCCCCCt 737

ZFA-unknown-130
728 gGCGGGGGGAa 738
728 cCGCCCCCCTt 738

ZFA-unknown-131
731 gGGGGGAAGGc 741
731 cCCCCCTTCCg 741

ZFA-unknown-132
732 gGGGGAAGGCg 742
732 cCCCCTTCCGc 742

ZFA-unknown-133
735 gGAAGGCGCCg 745
735 cCTTCCGCGGc 745

ZFA-unknown-134
738 aGGCGCCGGAg 748
738 tCCGCGGCCTc 748

ZFA-unknown-135
741 cGCCGGAGGCc 751
741 gCGGCCTCCGg 751

ZFA-unknown-136
757 gTGCGGGGCCt 747
757 cACGCCCCGGa 747

ZFA-unknown-137
763 cGCGGCGTGCg 753
763 gCGCCGCACGc 753

ZFA-unknown-138
766 aGGCGCGGCGt 756
766 tCCGCGCCGCa 756

ZFA-unknown-139
769 gGCAGGCGCGg 759
769 cCGTCCGCGCc 759

ZFA-unknown-140
772 gGCGGCAGGCg 762
772 cCGCCGTCCGc 762

ZFA-unknown-141
775 gGCGGCGGCAg 765
775 cCGCCGCCGTc 765

ZFA-unknown-142
766 tGCCGCCGCCt 776
766 aCGGCGGCGGa 776

ZFA-unknown-144
779 gGCAGGCGGCg 769
779 cCGTCCGCCGc 769

ZFA-unknown-143

769 cGCCGCCTGCc 779
769 gCGGCGGACGg 779

ZFA-unknown-145
782 gGGGGCAGGCg 772
782 cCCCCGTCCGc 772

ZFA-unknown-146
786 gGCGGGGGGCa 776
786 cCGCCCCCCGt 776

ZFA-unknown-147
787 cGGCGGGGGGc 777
787 gCCGCCCCCCg 777

ZFA-unknown-148
789 gGCGGCGGGGg 779
789 cCGCCGCCCCc 779

ZFA-unknown-149
792 gGCGGCGGCGg 782
792 cCGCCGCCGCc 782

ZFA-unknown-150
783 cGCCGCCGCCg 793
783 gCGGCGGCGGc 793

ZFA-unknown-151
795 cGCGGCGGCGg 785
795 gCGCCGCCGCc 785

ZFA-unknown-152
786 cGCCGCCGCGc 796
786 gCGGCGGCGCg 796

ZFA-unknown-153
798 tTGCGCGGCGg 788
798 aACGCGCCGCc 788

ZFA-unknown-154
816 cGCTGCGGCCc 806
816 gCGACGCCGGg 806

ZFA-unknown-155
807 gGCCGCAGCGc 817
807 cCGGCGTCGCg 817

ZFA-unknown-156
819 gGGCGCTGCGg 809
819 cCCGCGACGCc 809

ZFA-unknown-157
822 aGGGGGCGCTg 812
822 tCCCCCGCGAc 812

ZFA-unknown-158
825 gGAAGGGGGCg 815
825 cCTTCCCCCGc 815

ZFA-unknown-159
828 gGCGGAAGGGg 818
828 cCGCCTTCCCc 818

ZFA-unknown-160
829 gGGCGGAAGGg 819
829 cCCGCCTTCCc 819

ZFA-unknown-161
831 tGGGGCGGAAg 821
831 aCCCCGCCTTc 821

ZFA-unknown-162
832 gTGGGGCGGAa 822
832 cACCCCGCCTt 822

ZFA-unknown-163
834 aGGTGGGGCGg 824
834 tCCACCCCGCc 824

ZFA-unknown-164
846 aGCCGTAGCGc 836
846 tCGGCATCGCg 836

ZFA-unknown-165
849 cGCAGCCGTAg 839
849 gCGTCGGCATc 839

ZFA-unknown-166
859 cGTCGTGGTGc 849
859 gCAGCACCACg 849

ZFA-unknown-167
855 cGACGGAGTAc 865
855 gCTGCCTCATg 865

ZFA-unknown-168
888 cGGCGCAGGCt 878
888 gCCGCGTCCGa 878

ZFA-unknown-170
891 cGCCGGCGCAg 881
891 gCGGCCGCGTc 881

ZFA-unknown-169
881 cTGCGCCGGCg 891
881 gACGCGGCCGc 891

ZFA-unknown-172
894 cGCCGCCGGCg 884
894 gCGGCGGCCGc 884

ZFA-unknown-171
884 cGCCGGCGGCg 894
884 gCGGCCGCCGc 894

ZFA-unknown-173
897 gGCCGCCGCCg 887
897 cCGGCGGCGGc 887

ZFA-unknown-174
888 gGCGGCGGCCc 898
888 cCGCCGCCGGg 898

ZFA-unknown-175
900 gGGGGCCGCCg 890
900 cCCCCGGCGGc 890

ZFA-unknown-176
904 cGCGGGGGGCc 894
904 gCGCCCCCCGg 894

ZFA-unknown-177
907 gGACGCGGGGg 897
907 cCTGCGCCCCc 897

ZFA-unknown-178
910 cGGGGACGCGg 900
910 gCCCCTGCGCc 900

ZFA-unknown-179
912 gGCGGGGACGc 902
912 cCGCCCCTGCg 902

ZFA-unknown-180
913 gGGCGGGGACg 903
913 cCCGCCCCTGc 903

ZFA-unknown-181
916 cGCGGGCGGGg 906
916 gCGCCCGCCCc 906

ZFA-unknown-182
919 gTCCGCGGGCg 909
919 cAGGCGCCCGc 909

ZFA-unknown-183
913 cGCGGACGCGc 923
913 gCGCCTGCGCg 923

ZFA-unknown-184
926 cGCGGGAAGGt 936
926 gCGCCCTTCCa 936

ZFA-unknown-185
930 gGAAGGTACGc 940
930 cCTTCCATGCg 940

ZFA-unknown-186
945 gGGAGGCGTAc 935
945 cCCTCCGCATg 935

ZFA-unknown-187
947 aGGGGAGGCGt 937
947 tCCCCTCCGCa 937

ZFA-unknown-188
950 cGGAGGGGAGg 940
950 gCCTCCCCTCc 940

ZFA-unknown-189
952 gTCGGAGGGGa 942
952 cAGCCTCCCCt 942

ZFA-unknown-190
953 gGTCGGAGGGg 943
953 cCAGCCTCCCc 943

ZFA-unknown-191
956 gGGGGTCGGAg 946
956 cCCCCAGCCTc 946

ZFA-unknown-192
969 cGGAGGGGCGt 959
969 gCCTCCCCGCa 959

ZFA-unknown-193
971 gTCGGAGGGGc 961

971 cAGCCTCCCCg 961

ZFA-unknown-194
972 gGTCGGAGGGg 962
972 cCAGCCTCCCc 962

ZFA-unknown-195
975 gGGGGTCGGAg 965
975 cCCCCAGCCTc 965

ZFA-unknown-196
988 cGGAGGGGCGt 978
988 gCCTCCCCGCa 978

ZFA-unknown-197
990 gTCGGAGGGGc 980
990 cAGCCTCCCCg 980

ZFA-unknown-198
991 gGTCGGAGGGg 981
991 cCAGCCTCCCc 981

ZFA-unknown-199
994 gGGGGTCGGAg 984
994 cCCCCAGCCTc 984

ZFA-unknown-200
1007 cGGAGGGGCGt 997
1007 gCCTCCCCGCa 997

ZFA-unknown-201
1009 gTCGGAGGGGc 999
1009 cAGCCTCCCCg 999

ZFA-unknown-202
1010 gGTCGGAGGGg 1000
1010 cCAGCCTCCCc 1000

ZFA-unknown-203
1013 gGGGGTCGGAg 1003
1013 cCCCCAGCCTc 1003

ZFA-unknown-204
1026 cGGAGGGGCGt 1016
1026 gCCTCCCCGCa 1016

ZFA-unknown-205
1028 gTCGGAGGGGc 1018
1028 cAGCCTCCCCg 1018

ZFA-unknown-206
1029 gGTCGGAGGGg 1019
1029 cCAGCCTCCCc 1019

ZFA-unknown-207
1032 gGGGGTCGGAg 1022
1032 cCCCCAGCCTc 1022

ZFA-unknown-208
1045 cGGAGGGGCGt 1035
1045 gCCTCCCCGCa 1035

ZFA-unknown-209
1047 gTCGGAGGGGc 1037
1047 cAGCCTCCCCg 1037

ZFA-unknown-210
1048 gGTCGGAGGGg 1038
1048 cCAGCCTCCCc 1038

ZFA-unknown-211
1051 gGGGGTCGGAg 1041
1051 cCCCCAGCCTc 1041

ZFA-unknown-212
1064 cGGAGGGGCGt 1054
1064 gCCTCCCCGCa 1054

ZFA-unknown-213
1066 gTCGGAGGGGc 1056
1066 cAGCCTCCCCg 1056

ZFA-unknown-214
1067 gGTCGGAGGGg 1057
1067 cCAGCCTCCCc 1057

ZFA-unknown-215
1070 gGGGGTCGGAg 1060
1070 cCCCCAGCCTc 1060

ZFA-unknown-216
1079 gGGAGACACGg 1069
1079 cCCTCTGTGCc 1069

ZFA-unknown-217
1084 gGGCGGGGAGa 1074
1084 cCCGCCCCTCt 1074

ZFA-unknown-218
1087 tGCGGGCGGGg 1077
1087 aCGCCCGCCCc 1077

ZFA-unknown-219
1084 cGCAGGTGTGc 1094
1084 gCGTCCACACg 1094

ZFA-unknown-220
1108 tGCAGGTGCGc 1118
1108 aCGTCCACGCg 1118

ZFA-unknown-221
1123 aGATGGCGCAc 1113
1123 tCTACCGCGTg 1113

ZFA-unknown-222
1130 cTGGGAGACGg 1140
1130 gACCCTCTGCc 1140

ZFA-unknown-223
1132 gGGAGACGGCc 1142
1132 cCCTCTGCCGg 1142

ZFA-unknown-224
1145 cGCGGCCGTCt 1135
1145 gCGCCGGCAGa 1135

ZFA-unknown-225
1155 cGACGGGGGTc 1165
1155 gCTGCCCCCAg 1165

ZFA-unknown-226

1191 cGCGGTCGGCc 1181
1191 gCGCCAGCCGg 1181

ZFA-unknown-227
1194 gGTCGCGGTCg 1184
1194 cCAGCGCCAGc 1184

ZFA-unknown-228
1207 cGGCGCCGGAa 1197
1207 gCCGCGGCCTt 1197

ZFA-unknown-229
1200 cGGCGCCGCGt 1210
1200 gCCGCGGCGCa 1210

ZFA-unknown-230
1208 cGTGGCGGCGg 1218
1208 gCACCGCCGCc 1218

ZFA-unknown-231
1220 gGCCGCCGCCa 1210
1220 cCGGCGGCGGt 1210

ZFA-unknown-232
1211 gGCGGCGGCCg 1221
1211 cCGCCGCCGGc 1221

ZFA-unknown-233
1223 cTCGGCCGCCg 1213
1223 gAGCCGGCGGc 1213

ZFA-unknown-234
1214 gGCGGCCGAGg 1224
1214 cCGCCGGCTCc 1224

ZFA-unknown-235
1217 gGCCGAGGCGg 1227
1217 cCGGCTCCGCc 1227

ZFA-unknown-236
1220 cGAGGCGGTCa 1230
1220 gCTCCGCCAGt 1230

ZFA-unknown-237
1289 cGAAGACGGCg 1299
1289 gCTTCTGCCGc 1299

ZFA-unknown-238
1292 aGACGGCGGAc 1302
1292 tCTGCCGCCTg 1302

ZFA-unknown-239
1310 cTCCGCGGGTc 1300
1310 gAGGCGCCCAg 1300

ZFA-unknown-240
1304 cGCGGAGGAGg 1314
1304 gCGCCTCCTCc 1314

ZFA-unknown-241
1306 cGGAGGAGGAg 1316
1306 gCCTCCTCCTc 1316

ZFA-unknown-242
1307 gGAGGAGGAGg 1317
1307 cCTCCTCCTCc 1317

ZFA-unknown-243
1309 aGGAGGAGGAg 1319
1309 tCCTCCTCCTc 1319

ZFA-unknown-244
1310 gGAGGAGGAGg 1320
1310 cCTCCTCCTCc 1320

ZFA-unknown-245
1312 aGGAGGAGGCg 1322
1312 tCCTCCTCCGc 1322

ZFA-unknown-246
1313 gGAGGAGGCGg 1323
1313 cCTCCTCCGCc 1323

ZFA-unknown-247
1315 aGGAGGCGGCg 1325
1315 tCCTCCGCCGc 1325

ZFA-unknown-248
1316 gGAGGCGGCGg 1326
1316 cCTCCGCCGCc 1326

ZFA-unknown-249
1328 cGCCGCCGCCt 1318
1328 gCGGCGGCGGa 1318

ZFA-unknown-250
1319 gGCGGCGGCGg 1329
1319 cCGCCGCCGCc 1329

ZFA-unknown-251
1331 cGCCGCCGCCg 1321
1331 gCGGCGGCGGc 1321

ZFA-unknown-252
1322 gGCGGCGGCGg 1332
1322 cCGCCGCCGCc 1332

ZFA-unknown-253
1334 cGCCGCCGCCg 1324
1334 gCGGCGGCGGc 1324

ZFA-unknown-254
1325 gGCGGCGGCGc 1335
1325 cCGCCGCCGCg 1335

ZFA-unknown-255
1349 gGCGGAGGACc 1339
1349 cCGCCTCCTGg 1339

ZFA-unknown-256
1350 cGGCGGAGGAc 1340
1350 gCCGCCTCCTg 1340

ZFA-unknown-257
1352 gGCGGCGGAGg 1342
1352 cCGCCGCCTCc 1342

ZFA-unknown-258
1355 cGCGGCGGCGg 1345
1355 gCGCCGCCGCc 1345

ZFA-unknown-259
1346 cGCCGCCGCGg 1356
1346 gCGGCGGCGCc 1356

ZFA-unknown-260
1349 cGCCGCGGGCc 1359
1349 gCGGCGCCCGg 1359

ZFA-unknown-261
1362 gGCCGTCGGGc 1372
1362 cCGGCAGCCCg 1372

ZFA-unknown-262
1366 gTCGGGCGGTc 1376
1366 cAGCCCGCCAg 1376

ZFA-unknown-263
1370 gGCGGTCTAGg 1380
1370 cCGCCAGATCc 1380

ZFA-unknown-264
1388 cGGCGAGGGCg 1398
1388 gCCGCTCCCGc 1398

ZFA-unknown-265
1391 cGAGGGCGGCc 1401
1391 gCTCCCGCCGg 1401

ZFA-unknown-266
1404 cGAGGCCGCCc 1394
1404 gCTCCGGCGGg 1394

ZFA-unknown-267
1407 gGCCGAGGCCg 1397
1407 cCGGCTCCGGc 1397

ZFA-unknown-268
1401 cTCGGCCGGCg 1411
1401 gAGCCGGCCGc 1411

ZFA-unknown-269
1404 gGCCGGCGGAg 1414
1404 cCGGCCGCCTc 1414

ZFA-unknown-270
1407 cGGCGGAGCCc 1417
1407 gCCGCCTCGGg 1417

ZFA-unknown-271
1433 cTGCGCGAGGc 1443
1433 gACGCGCTCCg 1443

ZFA-unknown-272
1438 cGAGGCCGCTc 1448
1438 gCTCCGGCGAg 1448

ZFA-unknown-273
1451 cGAAGAGACGa 1461
1451 gCTTCTCTGCt 1461

ZFA-unknown-274
1456 aGACGATGGGa 1466
1456 tCTGCTACCCt 1466

ZFA-unknown-275
1480 aTCCGCGAGGg 1490

1480 tAGGCGCTCCc 1490

ZFA-unknown-276
1500 cGGCGCCGGGc 1490
1500 gCCGCGGCCCg 1490

ZFA-unknown-277
1493 cGGCGCCGCCc 1503
1493 gCCGCGGCGGg 1503

ZFA-unknown-278
1505 cGGGGCGGCGc 1495
1505 gCCCCGCCGCg 1495

ZFA-unknown-279
1508 cGGCGGGGCGg 1498
1508 gCCGCCCCGCc 1498

ZFA-unknown-280
1510 aGCGGCGGGGc 1500
1510 tCGCCGCCCCg 1500

ZFA-unknown-281
1513 cGGAGCGGCGg 1503
1513 gCCTCGCCGCc 1503

ZFA-unknown-282
1516 gGGCGGAGCGg 1506
1516 cCCGCCTCGCc 1506

ZFA-unknown-283
1519 gGCGGGCGGAg 1509
1519 cCGCCCGCCTc 1509

ZFA-unknown-284
1522 tGGGGCGGGCg 1512
1522 aCCCCGCCCGc 1512

ZFA-unknown-285
1523 cTGGGGCGGGc 1513
1523 gACCCCGCCCg 1513

ZFA-unknown-286
1522 aGGGGGCGGCg 1532
1522 tCCCCCGCCGc 1532

ZFA-unknown-287
1523 gGGGGCGGCGc 1533
1523 cCCCCGCCGCg 1533

ZFA-unknown-288
1535 cGGCGCCGCCc 1525
1535 gCCGCGGCGGg 1525

ZFA-unknown-290
1538 gGCCGGCGCCg 1528
1538 cCGGCCGCGGc 1528

ZFA-unknown-289
1528 cGGCGCCGGCc 1538
1528 gCCGCGGCCGg 1538

ZFA-unknown-291
1545 cGCGGTTGGCc 1535
1545 gCGCCAACCGg 1535

ZFA-unknown-292
1555 cGCGGCGGCGc 1545
1555 gCGCCGCCGCg 1545

ZFA-unknown-293
1546 cGCCGCCGCGc 1556
1546 gCGGCGGCGCg 1556

ZFA-unknown-294
1573 gGGCGGAGTCc 1563
1573 cCCGCCTCAGg 1563

ZFA-unknown-295
1575 cGGGGCGGAGt 1565
1575 gCCCCGCCTCa 1565

ZFA-unknown-296
1578 cGCCGGGGCGg 1568
1578 gCGGCCCCGCc 1568

ZFA-unknown-297
1581 gGTCGCCGGGg 1571
1581 cCAGCGGCCCc 1571

ZFA-unknown-298
1584 gGCGGTCGCCg 1574
1584 cCGCCAGCGGc 1574

ZFA-unknown-299
1587 cGGGGCGGTCg 1577
1587 gCCCCGCCAGc 1577

ZFA-unknown-300
1592 cGGCGCGGGGc 1582
1592 gCCGCGCCCCg 1582

ZFA-unknown-301
1595 aGCCGGCGCGg 1585
1595 tCGGCCGCGCc 1585

ZFA-unknown-302
1598 gGAAGCCGGCg 1588
1598 cCTTCGGCCGc 1588

ZFA-unknown-303
1623 aTAGGCGGTCc 1633
1623 tATCCGCCAGg 1633

ZFA-unknown-304
1641 gGCGGCCGGGa 1631
1641 cCGCCGGCCCt 1631

ZFA-unknown-305
1645 gGATGGCGGCc 1635
1645 cCTACCGCCGg 1635

ZFA-unknown-306
1648 cGGGGATGGCg 1638
1648 gCCCCTACCGc 1638

ZFA-unknown-307
1653 aTGCGCGGGGa 1643
1653 tACGCGCCCCt 1643

ZFA-unknown-308

1672 aTGGGCCGGCg 1682
1672 tACCCGGCCGc 1682

ZFA-unknown-309
1675 gGCCGGCGTTc 1685
1675 cCGGCCGCAAg 1685

ZFA-unknown-310
1690 aGCGGGAACGc 1680
1690 tCGCCCTTGCg 1680

ZFA-unknown-311
1748 tTCGGACGGCc 1758
1748 aAGCCTGCCGg 1758

ZFA-unknown-312
1761 aGGGGCCGTCc 1751
1761 tCCCCGGCAGg 1751

ZFA-unknown-313
1768 gGGAGCGAGGg 1758
1768 cCCTCGCTCCc 1758

ZFA-unknown-314
1786 cGAGGGAGTAa 1776
1786 gCTCCCTCATt 1776

ZFA-unknown-315
1839 cGCCGCCGGGt 1849
1839 gCGGCGGCCCa 1849

ZFA-unknown-316
1847 gGTGGGTGGGc 1857
1847 cCACCCACCCg 1857

ZFA-unknown-317
1848 gTGGGTGGGCt 1858
1848 cACCCACCCGa 1858

ZFA-unknown-318
1891 cTAGGAAGCCc 1881
1891 gATCCTTCGGg 1881

ZFA-unknown-319
1912 cGGGGGCGGGc 1922
1912 gCCCCCGCCCg 1922

ZFA-unknown-320
1913 gGGGGCGGGCa 1923
1913 cCCCCGCCCGt 1923

ZFA-unknown-321
1941 cGGCGCGGCCc 1931
1941 gCCGCGCCGGg 1931

ZFA-unknown-322
1944 cGCCGGCGCGg 1934
1944 gCGGCCGCGCc 1934

ZFA-unknown-323
1937 cGCCGGCGGGg 1947
1937 gCGGCCGCCCc 1947

ZFA-unknown-324
1940 cGGCGGGGCGc 1950
1940 gCCGCCCCGCg 1950

ZFA-unknown-325
1945 gGGCGCCGGGg 1955
1945 cCCGCGGCCCc 1955

ZFA-unknown-326
1948 cGCCGGGGGGa 1958
1948 gCGGCCCCCCt 1958

ZFA-unknown-327
1970 cGCAGATGGGa 1960
1970 gCGTCTACCCt 1960

ZFA-unknown-328
1973 cGACGCAGATg 1963
1973 gCTGCGTCTAc 1963

ZFA-unknown-330
1976 cGCCGACGCAg 1966
1976 gCGGCTGCGTc 1966

ZFA-unknown-329
1966 cTGCGTCGGCg 1976
1966 gACGCAGCCGc 1976

ZFA-unknown-331
1969 cGTCGGCGGGg 1979
1969 gCAGCCGCCCc 1979

ZFA-unknown-332
1970 gTCGGCGGGGg 1980
1970 cAGCCGCCCCc 1980

ZFA-unknown-333
1972 cGGCGGGGGGc 1982
1972 gCCGCCCCCCg 1982

ZFA-unknown-334
1973 gGCGGGGGGCg 1983
1973 cCGCCCCCCGc 1983

ZFA-unknown-335
1976 gGGGGGCGGCg 1986
1976 cCCCCCGCCGc 1986

ZFA-unknown-336
1977 gGGGGCGGCGc 1987
1977 cCCCCGCCGCg 1987

ZFA-unknown-337
1989 aTGCGCCGCCc 1979
1989 tACGCGGCGGg 1979

ZFA-unknown-338
1982 cGGCGCATGCt 1992
1982 gCCGCGTACGa 1992

ZFA-unknown-339
2026 cGGGGACGGGg 2036
2026 gCCCCTGCCCc 2036

ZFA-unknown-340
2029 gGACGGGGCCg 2039
2029 cCTGCCCCGGc 2039

ZFA-unknown-341
2032 cGGGGCCGCCc 2042
2032 gCCCCGGCGGg 2042

ZFA-unknown-342
2044 cGGGGCGGCCc 2034
2044 gCCCCGCCGGg 2034

ZFA-unknown-343
2045 aGAGGGGGGGa 2055
2045 tCTCCCCCCCt 2055

ZFA-unknown-344
2049 gGGGGGATTCc 2059
2049 cCCCCCTAAGg 2059

ZFA-unknown-345
2067 gGAGGGAGGGa 2057
2067 cCTCCCTCCCt 2057

ZFA-unknown-346
2070 gGCGGAGGGAg 2060
2070 cCGCCTCCCTc 2060

ZFA-unknown-347
2071 gGGCGGAGGGa 2061
2071 cCCGCCTCCCt 2061

ZFA-unknown-348
2073 gGGGGCGGAGg 2063
2073 cCCCCGCCTCc 2063

ZFA-unknown-349
2074 cGGGGGCGGAg 2064
2074 gCCCCCGCCTc 2064

ZFA-unknown-350
2076 gGCGGGGGCGg 2066
2076 cCGCCCCCGCc 2066

ZFA-unknown-351
2077 cGGCGGGGGCg 2067
2077 gCCGCCCCCGc 2067

ZFA-unknown-352
2073 cGCCGGGGCGc 2083
2073 gCGGCCCCGCg 2083

ZFA-unknown-353
2116 aGGGGCGGCAt 2106
2116 tCCCCGCCGTa 2106

ZFA-unknown-354
2118 tGGGGGAGTGg 2128
2118 aCCCCCTCACc 2128

ZFA-unknown-355
2124 aGTGGATAGGc 2134
2124 tCACCTATCCg 2134

ZFA-unknown-356
2131 aGGCGCCGGGt 2141
2131 tCCGCGGCCCa 2141

ZFA-unknown-357
2155 cGGGGCTGCCt 2145

2155 gCCCCGACGGa 2145

ZFA-unknown-358
2180 gTGCGAATGCg 2170
2180 cACGCTTACGc 2170

ZFA-unknown-359
2187 tGCCGGGGTGc 2177
2187 aCGGCCCCACg 2177

ZFA-unknown-360
2195 cGACGGAGCGg 2205
2195 gCTGCCTCGCc 2205

ZFA-unknown-361
2198 cGGAGCGGCGg 2208
2198 gCCTCGCCGCc 2208

ZFA-unknown-362
2210 gGCCGCCGCTc 2200
2210 cCGGCGGCGAg 2200

ZFA-unknown-363
2201 aGCGGCGGCCc 2211
2201 tCGCCGCCGGg 2211

ZFA-unknown-364
2212 gGCGGGAGGAg 2222
2212 cCGCCCTCCTc 2222

ZFA-unknown-365
2215 gGGAGGAGGAg 2225
2215 cCCTCCTCCTc 2225

ZFA-unknown-366
2216 gGAGGAGGAGa 2226
2216 cCTCCTCCTCt 2226

ZFA-unknown-367
2218 aGGAGGAGACc 2228
2218 tCCTCCTCTGg 2228

ZFA-unknown-369
2280 aGACGGCGGCc 2270
2280 tCTGCCGCCGg 2270

ZFA-unknown-368
2270 gGCCGCCGTCt 2280
2270 cCGGCGGCAGa 2280

ZFA-unknown-370
2298 cGGGGTAGGGt 2288
2298 gCCCCATCCCa 2288

ZFA-unknown-371
2301 aGCCGGGGTAg 2291
2301 tCGGCCCCATc 2291

ZFA-unknown-372
2316 gGCCGGGGTTc 2306
2316 cCGGCCCCAAg 2306

ZFA-unknown-373
2345 gGCCGGGGTCc 2335
2345 cCGGCCCCAGg 2335

ZFA-unknown-374
2348 cGGGGCCGGGg 2338
2348 gCCCCGGCCCc 2338

ZFA-unknown-375
2358 gGCGGCCGCTc 2348
2358 cCGCCGGCGAg 2348

ZFA-unknown-376
2349 aGCGGCCGCCg 2359
2349 tCGCCGGCGGc 2359

ZFA-unknown-377
2361 cGCGGCGGCCg 2351
2361 gCGCCGCCGGc 2351

ZFA-unknown-378
2352 gGCCGCCGCGg 2362
2352 cCGGCGGCGCc 2362

ZFA-unknown-379
2364 tGCCGCGGCGg 2354
2364 aCGGCGCCGCc 2354

ZFA-unknown-380
2355 cGCCGCGGCAg 2365
2355 gCGGCGCCGTc 2365

ZFA-unknown-381
2358 cGCGGCAGACc 2368
2358 gCGCCGTCTGg 2368

ZFA-unknown-382
2371 gGGGGTCTGCc 2361
2371 cCCCCAGACGg 2361

ZFA-unknown-383
2375 tGCCGGGGGTc 2365
2375 aCGGCCCCCAg 2365

ZFA-unknown-384
2381 aGAGGGCGACc 2391
2381 tCTCCCGCTGg 2391

ZFA-unknown-385
2394 gGGGGTCGCCc 2384
2394 cCCCCAGCGGg 2384

ZFA-unknown-386
2412 aGGGGGGGCCt 2402
2412 tCCCCCCCGGa 2402

ZFA-unknown-387
2435 cGCAGCCGGGt 2425
2435 gCGTCGGCCCa 2425

ZFA-unknown-388
2429 gGCTGCGGGTt 2439
2429 cCGACGCCCAa 2439

ZFA-unknown-389
2436 gGTTGGGGGTg 2446
2436 cCAACCCCCAc 2446

ZFA-unknown-390

2439 tGGGGGTGGTc 2449
2439 aCCCCCACCAg 2449

ZFA-unknown-391
2440 gGGGGTGGTCg 2450
2440 cCCCCACCAGc 2450

ZFA-unknown-392
2443 gGTGGTCGCGg 2453
2443 cCACCAGCGCc 2453

ZFA-unknown-393
2446 gGTCGCGGGCg 2456
2446 cCAGCGCCCGc 2456

ZFA-unknown-394
2449 cGCGGGCGGTg 2459
2449 gCGCCCGCCAc 2459

ZFA-unknown-395
2452 gGGCGGTGGGc 2462
2452 cCCGCCACCCg 2462

ZFA-unknown-396
2453 gGCGGTGGGCt 2463
2453 cCGCCACCCGa 2463

ZFA-unknown-397
2462 cTCGGGGGCGg 2472
2462 gAGCCCCCGCc 2472

ZFA-unknown-398
2464 cGGGGGCGGGg 2474
2464 gCCCCCGCCCc 2474

ZFA-unknown-399
2465 gGGGGCGGGGa 2475
2465 cCCCCGCCCCt 2475

ZFA-unknown-400
2467 gGGCGGGGACg 2477
2467 cCCGCCCCTGc 2477

ZFA-unknown-401
2468 gGCGGGGACGc 2478
2468 cCGCCCCTGCg 2478

ZFA-unknown-402
2470 cGGGGACGCTt 2480
2470 gCCCCTGCGAa 2480

ZFA-unknown-403
2480 tGACGGGGCCg 2490
2480 aCTGCCCCGGc 2490

ZFA-unknown-404
2483 cGGGGCCGACc 2493
2483 gCCCCGGCTGg 2493

ZFA-unknown-405
2496 gGGGGTCGGCc 2486
2496 cCCCCAGCCGg 2486

ZFA-unknown-406
2500 gGCCGGGGGTc 2490
2500 cCGGCCCCCAg 2490

ZFA-unknown-407
2507 aGCGGTCGGGg 2517
2507 tCGCCAGCCCc 2517

ZFA-unknown-408
2510 gGTCGGGGGAc 2520
2510 cCAGCCCCCTg 2520

ZFA-unknown-409
2511 gTCGGGGGACc 2521
2511 cAGCCCCCTGg 2521

ZFA-unknown-410
2525 gTGGGCCGTGc 2535
2525 cACCCGGCACg 2535

ZFA-unknown-411
2532 gTGCGCCGCCc 2542
2532 cACGCGGCGGg 2542

ZFA-unknown-412
2544 gGGGGCGGCGc 2534
2544 cCCCCGCCGCg 2534

ZFA-unknown-413
2545 gGGGGGCGGCg 2535
2545 cCCCCCGCCGc 2535

ZFA-unknown-414
2548 gTCGGGGGGCg 2538
2548 cAGCCCCCCGc 2538

ZFA-unknown-415
2549 gGTCGGGGGGc 2539
2549 cCAGCCCCCCg 2539

ZFA-unknown-416
2552 cTGGGGGGGCg 2562
2552 gACCCCCCCGc 2562

ZFA-unknown-417
2553 tGGGGGGGCGa 2563
2553 aCCCCCCCGCt 2563

ZFA-unknown-418
2556 gGGGGCGAGGg 2566
2556 cCCCCGCTCCc 2566

ZFA-unknown-419
2558 gGGCGAGGGAg 2568
2558 cCCGCTCCCTc 2568

ZFA-unknown-420
2561 cGAGGGAGGCa 2571
2561 gCTCCCTCCGt 2571

ZFA-unknown-421
2565 gGAGGCAGGGa 2575
2565 cCTCCGTCCCt 2575

ZFA-unknown-422
2572 gGGAGGAGCCc 2582
2572 cCCTCCTCGGg 2582

ZFA-unknown-423
2586 aGCGGGGGACa 2596
2586 tCGCCCCCTGt 2596

ZFA-unknown-424
2589 gGGGGACAGGg 2599
2589 cCCCCTGTCCc 2599

ZFA-unknown-425
2596 aGGGGGGGAGa 2606
2596 tCCCCCCCTCt 2606

ZFA-unknown-426
2598 gGGGGGAGACg 2608
2598 cCCCCCTCTGc 2608

ZFA-unknown-427
2599 gGGGGAGACGa 2609
2599 cCCCCTCTGCt 2609

ZFA-unknown-428
2601 gGGAGACGAGg 2611
2601 cCCTCTGCTCc 2611

ZFA-unknown-429
2604 aGACGAGGGGt 2614
2604 tCTGCTCCCCa 2614

ZFA-unknown-430
2607 cGAGGGGTCGg 2617
2607 gCTCCCCAGCc 2617

ZFA-unknown-431
2609 aGGGGTCGGAa 2619
2609 tCCCCAGCCTt 2619

ZFA-unknown-432
2626 gGACGCAGACc 2636
2626 cCTGCGTCTGg 2636

ZFA-unknown-433
2639 gGTGGTCTGCg 2629
2639 cCACCAGACGc 2629

ZFA-unknown-434
2663 gGGAGAAAGGg 2653
2663 cCCTCTTTCCc 2653

ZFA-unknown-435
2666 gGGGGGAGAAa 2656
2666 cCCCCCTCTTt 2656

ZFA-unknown-436
2668 aGGGGGGGAGa 2658
2668 tCCCCCCCTCt 2658

ZFA-unknown-437
2671 gGAAGGGGGGg 2661
2671 cCTTCCCCCCc 2661

ZFA-unknown-438
2674 tTCGGAAGGGg 2664
2674 aAGCCTTCCCc 2664

ZFA-unknown-439
2680 aGCAGCGGGCg 2690

2680 tCGTCGCCCGc 2690

ZFA-unknown-440
2683 aGCGGGCGGGg 2693
2683 tCGCCCGCCCc 2693

ZFA-unknown-441
2686 gGGCGGGGGGc 2696
2686 cCCGCCCCCCg 2696

ZFA-unknown-442
2687 gGCGGGGGGCc 2697
2687 cCGCCCCCCGg 2697

ZFA-unknown-443
2691 gGGGGCCGGGg 2701
2691 cCCCCGGCCCc 2701

ZFA-unknown-444
2694 gGCCGGGGTGa 2704
2694 cCGGCCCCACt 2704

ZFA-unknown-445
2699 gGGTGAGGGAg 2709
2699 cCCACTCCCTc 2709

ZFA-unknown-446
2702 tGAGGGAGGGa 2712
2702 aCTCCCTCCCt 2712

ZFA-unknown-447
2716 gGGGGACACGg 2726
2716 cCCCCTGTGCc 2726

ZFA-unknown-448
2724 cGGCGCGGGGg 2734
2724 gCCGCGCCCCc 2734

ZFA-unknown-449
2727 cGCGGGGGTCc 2737
2727 gCGCCCCCAGg 2737

ZFA-unknown-450
2744 tGAGGCGGGAc 2734
2744 aCTCCGCCCTg 2734

ZFA-unknown-451
2756 gGGCGCGGGGc 2746
2756 cCCGCGCCCCg 2746

ZFA-unknown-452
2759 aGAGGGCGCGg 2749
2759 tCTCCCGCGCc 2749

ZFA-unknown-453
2796 gGCGGCGGGCt 2786
2796 cCGCCGCCCGa 2786

ZFA-unknown-454
2799 cGGGGCGGCGg 2789
2799 gCCCCGCCGCc 2789

ZFA-unknown-455
2802 gTGCGGGGCGg 2792
2802 cACGCCCCGCc 2792

ZFA-unknown-456
2806 tGGGGGATGCt 2816
2806 aCCCCCTACGa 2816

ZFA-unknown-457
2807 gGGGGATGCTc 2817
2807 cCCCCTACGAg 2817

ZFA-unknown-458
2824 tGTCGTTGAGc 2814
2824 aCAGCAACTCg 2814

ZFA-unknown-459
2825 tGCAGTGGCTc 2835
2825 aCGTCACCGAg 2835

ZFA-unknown-460
2845 cGCTGCTGGCg 2835
2845 gCGACGACCGc 2835

ZFA-unknown-461
2848 aGTCGCTGCTg 2838
2848 tCAGCGACGAc 2838

ZFA-unknown-462
2847 cTCGGAGGAGg 2857
2847 gAGCCTCCTCc 2857

ZFA-unknown-463
2849 cGGAGGAGGAg 2859
2849 gCCTCCTCCTc 2859

ZFA-unknown-464
2850 gGAGGAGGAGa 2860
2850 cCTCCTCCTCt 2860

ZFA-unknown-465
2852 aGGAGGAGACc 2862
2852 tCCTCCTCTGg 2862

ZFA-unknown-466
2862 cGAGGTGGGAa 2872
2862 gCTCCACCCTt 2872

ZFA-unknown-468
2887 gGTCGTCGTCa 2877
2887 cCAGCAGCAGt 2877

ZFA-unknown-467
2877 tGACGACGACc 2887
2877 aCTGCTGCTGg 2887

ZFA-unknown-469
2894 cGGTGAAGGTc 2884
2894 gCCACTTCCAg 2884

ZFA-unknown-470
2899 aGTCGCGGTGa 2889
2899 tCAGCGCCACt 2889

ZFA-unknown-471
2902 tGGAGTCGCGg 2892
2902 aCCTCAGCGCc 2892

ZFA-unknown-472

2904 gGTGGAGTCGc 2894
2904 cCACCTCAGCg 2894

ZFA-unknown-473
2905 aGGTGGAGTCg 2895
2905 tCCACCTCAGc 2895

ZFA-unknown-474
2907 gGAGGTGGAGt 2897
2907 cCTCCACCTCa 2897

ZFA-unknown-475
2908 cGGAGGTGGAg 2898
2908 gCCTCCACCTc 2898

ZFA-unknown-476
2910 cTCGGAGGTGg 2900
2910 gAGCCTCCACc 2900

ZFA-unknown-477
2907 cGAGGCGGGCa 2917
2907 gCTCCGCCCGt 2917

ZFA-unknown-478
2927 cGGAGATGTTc 2937
2927 gCCTCTACAAg 2937

ZFA-unknown-479
2937 cGAGGCGGGCc 2947
2937 gCTCCGCCCGg 2947

ZFA-unknown-480
2952 gGACGCGGCCa 2962
2952 cCTGCGCCGGt 2962

ZFA-unknown-481
2964 cGTGGCCGCGt 2954
2964 gCACCGGCGCa 2954

ZFA-unknown-482
2970 cGGGGGCGTGg 2960
2970 gCCCCCGCACc 2960

ZFA-unknown-483
2973 gGCCGGGGGCg 2963
2973 cCGGCCCCCGc 2963

ZFA-unknown-484
2981 gGGGGCCGGGc 2971
2981 cCCCCGGCCCg 2971

ZFA-unknown-485
2985 gGCCGGGGGCc 2975
2985 cCGGCCCCCGg 2975

ZFA-unknown-486
2988 cTCGGCCGGGg 2978
2988 gAGCCGGCCCc 2978

ZFA-unknown-487
3004 tGGGGCTGCCc 2994
3004 aCCCCGACGGg 2994

ZFA-unknown-488
3012 gGCGGGCGTGg 3002
3012 cCGCCCGCACc 3002

ZFA-unknown-489
3015 gTCGGCGGGCg 3005
3015 cAGCCGCCCGc 3005

ZFA-unknown-490
3016 cGTCGGCGGGc 3006
3016 gCAGCCGCCCg 3006

ZFA-unknown-491
3009 cGCCGACGCGc 3019
3009 gCGGCTGCGCg 3019

ZFA-unknown-492
3021 cTGCGCGTCGg 3011
3021 gACGCGCAGCc 3011

ZFA-unknown-493
3027 cTGTGGGGGTg 3037
3027 gACACCCCCAc 3037

ZFA-unknown-494
3028 tGTGGGGGTGg 3038
3028 aCACCCCCACc 3038

ZFA-unknown-495
3030 tGGGGGTGGGc 3040
3030 aCCCCCACCCg 3040

ZFA-unknown-496
3031 gGGGGTGGGCc 3041
3031 cCCCCACCCGg 3041

ZFA-unknown-497
3042 cGTGGGTGAGg 3052
3042 gCACCCACTCc 3052

ZFA-unknown-498
3045 gGGTGAGGAGg 3055
3045 cCCACTCCTCc 3055

ZFA-unknown-499
3048 tGAGGAGGAAg 3058
3048 aCTCCTCCTTc 3058

ZFA-unknown-500
3051 gGAGGAAGCGg 3061
3051 cCTCCTTCGCc 3061

ZFA-unknown-501
3054 gGAAGCGGAAg 3064
3054 cCTTCGCCTTc 3064

ZFA-unknown-502
3057 aGCGGAAGCGg 3067
3057 tCGCCTTCGCc 3067

ZFA-unknown-503
3060 gGAAGCGGGAg 3070
3060 cCTTCGCCCTc 3070

ZFA-unknown-504
3063 aGCGGGAGGGg 3073
3063 tCGCCCTCCCc 3073

ZFA-unknown-505
3066 gGGAGGGGGGg 3076
3066 cCCTCCCCCCc 3076

ZFA-unknown-506
3067 gGAGGGGGGGg 3077
3067 cCTCCCCCCCc 3077

ZFA-unknown-507
3069 aGGGGGGGGCg 3079
3069 tCCCCCCCCGc 3079

ZFA-unknown-508
3070 gGGGGGGGCGa 3080
3070 cCCCCCCCGCt 3080

ZFA-unknown-509
3072 gGGGGGCGACg 3082
3072 cCCCCCGCTGc 3082

ZFA-unknown-510
3073 gGGGGCGACGt 3083
3073 cCCCCGCTGCa 3083

ZFA-unknown-511
3078 cGACGTGTGTg 3088
3078 gCTGCACACAc 3088

ZFA-unknown-512
3084 gTGTGCCGTGt 3094
3084 cACACGGCACa 3094

ZFA-unknown-513
3117 gGGCGGGGCGa 3107
3117 cCCGCCCCGCt 3107

ZFA-unknown-514
3119 aGGGGCGGGGc 3109
3119 tCCCCGCCCCg 3109

ZFA-unknown-515
3122 cGCAGGGGCGg 3112
3122 gCGTCCCCGCc 3112

ZFA-unknown-516
3118 cTGCGCTGCCa 3128
3118 gACGCGACGGt 3128

ZFA-unknown-517
3149 gGGTGCAGGCa 3139
3149 cCCACGTCCGt 3139

ZFA-unknown-518
3154 aGAAGGGGTGc 3144
3154 tCTTCCCCACg 3144

ZFA-unknown-519
3157 tGCAGAAGGGg 3147
3157 aCGTCTTCCCc 3147

ZFA-unknown-520
3160 gGATGCAGAAg 3150
3160 cCTACGTCTTc 3150

ZFA-unknown-521
3203 aGGGGACACGt 3193

3203 tCCCCTGTGCa 3193

ZFA-unknown-522
3214 gGGTGTTGCAc 3204
3214 cCCACAACGTg 3204

ZFA-unknown-523
3215 cGGTGGCGTAc 3225
3215 gCCACCGCATg 3225

ZFA-unknown-524
3231 aGTGGGCGTGa 3241
3231 tCACCCGCACt 3241

ZFA-unknown-525
3265 tGGTGCTGAAc 3255
3265 aCCACGACTTg 3255

ZFA-unknown-526
3294 cGTGGAGGCCg 3304
3294 gCACCTCCGGc 3304

ZFA-unknown-527
3297 gGAGGCCGAGg 3307
3297 cCTCCGGCTCc 3307

ZFA-unknown-528
3300 gGCCGAGGCGg 3310
3300 cCGGCTCCGCc 3310

ZFA-unknown-529
3303 cGAGGCGGCCg 3313
3303 gCTCCGCCGGc 3313

ZFA-unknown-530
3306 gGCGGCCGTGc 3316
3306 cCGCCGGCACg 3316

ZFA-unknown-531
3327 gGCCGTGGACt 3337
3327 cCGGCACCTGa 3337

ZFA-unknown-532
3362 gTCCGCGGGTt 3352
3362 cAGGCGCCCAa 3352

ZFA-unknown-533
3356 cGCGGACGGCc 3366
3356 gCGCCTGCCGg 3366

ZFA-unknown-534
3369 cGGGGCCGTCc 3359
3369 gCCCCGGCAGg 3359

ZFA-unknown-535
3377 tGTCGCTGGGg 3387
3377 aCAGCGACCCc 3387

ZFA-unknown-536
3380 cGCTGGGGGGa 3390
3380 gCGACCCCCCt 3390

ZFA-unknown-537
3382 cTGGGGGGACa 3392
3382 gACCCCCCTGt 3392

ZFA-unknown-538
3406 gGGCGCGGACc 3396
3406 cCCGCGCCTGg 3396

ZFA-unknown-539
3417 gGTGGGCGACa 3407
3417 cCACCCGCTGt 3407

ZFA-unknown-540
3420 gGGGGTGGGCg 3410
3420 cCCCCACCCGc 3410

ZFA-unknown-541
3421 gGGGGGTGGGc 3411
3421 cCCCCCACCCg 3411

ZFA-unknown-542
3423 cGGGGGGGTGg 3413
3423 gCCCCCCCACc 3413

ZFA-unknown-543
3435 gGACGACGAGg 3445
3435 cCTGCTGCTCc 3445

ZFA-unknown-544
3438 cGACGAGGACg 3448
3438 gCTGCTCCTGc 3448

ZFA-unknown-545
3444 gGACGATGACc 3454
3444 cCTGCTACTGg 3454

ZFA-unknown-546
3459 cGACGGTGAGg 3469
3459 gCTGCCACTCc 3469

ZFA-unknown-547
3462 cGGTGAGGGCg 3472
3462 gCCACTCCCGc 3472

ZFA-unknown-548
3465 tGAGGGCGGGc 3475
3465 aCTCCCGCCCg 3475

ZFA-unknown-549
3469 gGCGGGCGGGg 3479
3469 cCGCCCGCCCc 3479

ZFA-unknown-550
3472 gGGCGGGGGTc 3482
3472 cCCGCCCCCAg 3482

ZFA-unknown-551
3473 gGCGGGGGTCg 3483
3473 cCGCCCCCAGc 3483

ZFA-unknown-552
3476 gGGGGTCGGGc 3486
3476 cCCCCAGCCCg 3486

ZFA-unknown-553
3480 gTCGGGCGGGg 3490
3480 cAGCCCGCCCc 3490

ZFA-unknown-554

3483 gGGCGGGGGGc 3493
3483 cCCGCCCCCCg 3493

ZFA-unknown-555
3484 gGCGGGGGGCg 3494
3484 cCGCCCCCCGc 3494

ZFA-unknown-556
3487 gGGGGGCGGGc 3497
3487 cCCCCCGCCCg 3497

ZFA-unknown-557
3488 gGGGGCGGGCg 3498
3488 cCCCCGCCCGc 3498

ZFA-unknown-558
3491 gGCGGGCGGGg 3501
3491 cCGCCCGCCCc 3501

ZFA-unknown-559
3494 gGGCGGGGGTc 3504
3494 cCCGCCCCCAg 3504

ZFA-unknown-560
3495 gGCGGGGGTCg 3505
3495 cCGCCCCCAGc 3505

ZFA-unknown-561
3498 gGGGGTCGGGc 3508
3498 cCCCCAGCCCg 3508

ZFA-unknown-562
3502 gTCGGGCGGGg 3512
3502 cAGCCCGCCCc 3512

ZFA-unknown-563
3505 gGGCGGGGGTc 3515
3505 cCCGCCCCCAg 3515

ZFA-unknown-564
3506 gGCGGGGGTCg 3516
3506 cCGCCCCCAGc 3516

ZFA-unknown-565
3509 gGGGGTCGGGc 3519
3509 cCCCCAGCCCg 3519

ZFA-unknown-566
3513 gTCGGGCGGGg 3523
3513 cAGCCCGCCCc 3523

ZFA-unknown-567
3516 gGGCGGGGGTc 3526
3516 cCCGCCCCCAg 3526

ZFA-unknown-568
3517 gGCGGGGGTCg 3527
3517 cCGCCCCCAGc 3527

ZFA-unknown-569
3520 gGGGGTCGGGc 3530
3520 cCCCCAGCCCg 3530

ZFA-unknown-570
3524 gTCGGGCGGGg 3534
3524 cAGCCCGCCCc 3534

ZFA-unknown-571
3527 gGGCGGGGGTc 3537
3527 cCCGCCCCCAg 3537

ZFA-unknown-572
3528 gGCGGGGGTCg 3538
3528 cCGCCCCCAGc 3538

ZFA-unknown-573
3531 gGGGGTCGGGc 3541
3531 cCCCCAGCCCg 3541

ZFA-unknown-574
3535 gTCGGGCGGGg 3545
3535 cAGCCCGCCCc 3545

ZFA-unknown-575
3538 gGGCGGGGGTc 3548
3538 cCCGCCCCCAg 3548

ZFA-unknown-576
3539 gGCGGGGGTCg 3549
3539 cCGCCCCCAGc 3549

ZFA-unknown-577
3542 gGGGGTCGGGc 3552
3542 cCCCCAGCCCg 3552

ZFA-unknown-578
3546 gTCGGGCGGGg 3556
3546 cAGCCCGCCCc 3556

ZFA-unknown-579
3549 gGGCGGGGGTc 3559
3549 cCCGCCCCCAg 3559

ZFA-unknown-580
3550 gGCGGGGGTCg 3560
3550 cCGCCCCCAGc 3560

ZFA-unknown-581
3553 gGGGGTCGGGc 3563
3553 cCCCCAGCCCg 3563

ZFA-unknown-582
3557 gTCGGGCGGGg 3567
3557 cAGCCCGCCCc 3567

ZFA-unknown-583
3560 gGGCGGGGGTc 3570
3560 cCCGCCCCCAg 3570

ZFA-unknown-584
3561 gGCGGGGGTCg 3571
3561 cCGCCCCCAGc 3571

ZFA-unknown-585
3564 gGGGGTCGGGc 3574
3564 cCCCCAGCCCg 3574

ZFA-unknown-586
3568 gTCGGGCGGGg 3578
3568 cAGCCCGCCCc 3578

ZFA-unknown-587
3571 gGGCGGGGGTc 3581
3571 cCCGCCCCCAg 3581

ZFA-unknown-588
3572 gGCGGGGGTCg 3582
3572 cCGCCCCCAGc 3582

ZFA-unknown-589
3575 gGGGGTCGGGc 3585
3575 cCCCCAGCCCg 3585

ZFA-unknown-590
3579 gTCGGGCGGGg 3589
3579 cAGCCCGCCCc 3589

ZFA-unknown-591
3582 gGGCGGGGGTc 3592
3582 cCCGCCCCCAg 3592

ZFA-unknown-592
3583 gGCGGGGGTCg 3593
3583 cCGCCCCCAGc 3593

ZFA-unknown-593
3586 gGGGGTCGGGc 3596
3586 cCCCCAGCCCg 3596

ZFA-unknown-594
3590 gTCGGGCGGGg 3600
3590 cAGCCCGCCCc 3600

ZFA-unknown-595
3593 gGGCGGGGGTc 3603
3593 cCCGCCCCCAg 3603

ZFA-unknown-596
3594 gGCGGGGGTCg 3604
3594 cCGCCCCCAGc 3604

ZFA-unknown-597
3597 gGGGGTCGGGc 3607
3597 cCCCCAGCCCg 3607

ZFA-unknown-598
3640 aGAGGGAGACa 3630
3640 tCTCCCTCTGt 3630

ZFA-unknown-599
3643 tGCAGAGGGAg 3633
3643 aCGTCTCCCTc 3633

ZFA-unknown-600
3640 tGCAGTGGACt 3650
3640 aCGTCACCTGa 3650

ZFA-unknown-601
3656 gGACGTAGTCc 3646
3656 cCTGCATCAGg 3646

ZFA-unknown-602
3661 gGGCGGGACGt 3651
3661 cCCGCCCTGCa 3651

ZFA-unknown-603
3664 gGCGGGCGGGa 3654

3664 cCGCCCGCCCt 3654

ZFA-unknown-604
3667 gGGGGCGGGCg 3657
3667 cCCCCGCCCGc 3657

ZFA-unknown-605
3668 gGGGGGCGGGc 3658
3668 cCCCCCGCCCg 3658

ZFA-unknown-606
3671 tTCGGGGGGCg 3661
3671 aAGCCCCCCGc 3661

ZFA-unknown-607
3684 cGCCGGGGCGc 3674
3684 gCGGCCCCGCg 3674

ZFA-unknown-608
3680 cGGCGCGGGGg 3690
3680 gCCGCGCCCCc 3690

ZFA-unknown-609
3683 cGCGGGGGCGg 3693
3683 gCGCCCCCGCc 3693

ZFA-unknown-610
3685 cGGGGGCGGCg 3695
3685 gCCCCCGCCGc 3695

ZFA-unknown-611
3686 gGGGGCGGCGg 3696
3686 cCCCCGCCGCc 3696

ZFA-unknown-612
3689 gGCGGCGGTGc 3699
3689 cCGCCGCCACg 3699

ZFA-unknown-613
3691 cGGCGGTGCGg 3701
3691 gCCGCCACGCc 3701

ZFA-unknown-614
3694 cGGTGCGGGGg 3704
3694 gCCACGCCCCc 3704

ZFA-unknown-615
3696 gTGCGGGGGCg 3706
3696 cACGCCCCCGc 3706

ZFA-unknown-616
3697 tGCGGGGGCGa 3707
3697 aCGCCCCCGCt 3707

ZFA-unknown-617
3699 cGGGGGCGACc 3709
3699 gCCCCCGCTGg 3709

ZFA-unknown-618
3716 tTCCGCGGGTc 3706
3716 aAGGCGCCCAg 3706

ZFA-unknown-619
3724 cTGGGAGGTTc 3714
3724 gACCCTCCAAg 3714

ZFA-unknown-620
3733 cGCGGCGGGCt 3723
3733 gCGCCGCCCGa 3723

ZFA-unknown-621
3736 gGTCGCGGCGg 3726
3736 cCAGCGCCGCc 3726

ZFA-unknown-622
3741 gGTCGGGTCGc 3731
3741 cCAGCCCAGCg 3731

ZFA-unknown-623
3748 gGGCGCCGGTc 3738
3748 cCCGCGGCCAg 3738

ZFA-unknown-624
3751 aGGGGGCGCCg 3741
3751 tCCCCCGCGGc 3741

ZFA-unknown-625
3765 cTCCGCGGGGc 3755
3765 gAGGCGCCCCg 3755

ZFA-unknown-626
3761 cGGAGCAGCAg 3771
3761 gCCTCGTCGTc 3771

ZFA-unknown-627
3773 tGCTGCTGCTc 3763
3773 aCGACGACGAg 3763

ZFA-unknown-628
3764 aGCAGCAGCAg 3774
3764 tCGTCGTCGTc 3774

ZFA-unknown-629
3776 cGCTGCTGCTg 3766
3776 gCGACGACGAc 3766

ZFA-unknown-630
3767 aGCAGCAGCGg 3777
3767 tCGTCGTCGCc 3777

ZFA-unknown-631
3779 cGCCGCTGCTg 3769
3779 gCGGCGACGAc 3769

ZFA-unknown-632
3770 aGCAGCGGCGg 3780
3770 tCGTCGCCGCc 3780

ZFA-unknown-633
3782 cGCCGCCGCTg 3772
3782 gCGGCGGCGAc 3772

ZFA-unknown-634
3773 aGCGGCGGCGc 3783
3773 tCGCCGCCGCg 3783

ZFA-unknown-635
3785 gGGCGCCGCCg 3775
3785 cCCGCGGCGGc 3775

ZFA-unknown-636

```
3786 cGTTGCGGGCg 3796
3786 gCAACGCCCGc 3796

ZFA-unknown-637
3789 tGCGGGCGGGg 3799
3789 aCGCCCGCCCc 3799

ZFA-unknown-638
3792 gGGCGGGGGTg 3802
3792 cCCGCCCCCAc 3802

ZFA-unknown-639
3793 gGCGGGGGTGg 3803
3793 cCGCCCCCACc 3803

ZFA-unknown-640
3795 cGGGGGTGGGa 3805
3795 gCCCCCACCCt 3805

ZFA-unknown-641
3796 gGGGGTGGGAt 3806
3796 cCCCCACCCTa 3806

ZFA-unknown-642
3814 tGGGGGCGGCc 3824
3814 aCCCCCGCCGg 3824

ZFA-unknown-643
3815 gGGGGCGGCCc 3825
3815 cCCCCGCCGGg 3825

ZFA-unknown-644
3826 tGCCGTCGCGg 3836
3826 aCGGCAGCGCc 3836

ZFA-unknown-645
3838 gGCCGCGACGg 3828
3838 cCGGCGCTGCc 3828

ZFA-unknown-646
3829 cGTCGCGGCCg 3839
3829 gCAGCGCCGGc 3839

ZFA-unknown-647
3832 cGCGGCCGTCg 3842
3832 gCGCCGGCAGc 3842

ZFA-unknown-648
3835 gGCCGTCGTGc 3845
3835 cCGGCAGCACg 3845

ZFA-unknown-649
3867 gGGGGAAGAGa 3857
3867 cCCCCTTCTCt 3857

ZFA-unknown-650
3871 cGCAGGGGGAa 3861
3871 gCGTCCCCCTt 3861

ZFA-unknown-651
3874 gGCCGCAGGGg 3864
3874 cCGGCGTCCCc 3864

ZFA-unknown-652
3868 tGCGGCCGGCg 3878
3868 aCGCCGGCCGc 3878
```

ZFA-unknown-653
3871 gGCCGGCGGGg 3881
3871 cCGGCCGCCCc 3881

ZFA-unknown-654
3874 cGGCGGGGGGc 3884
3874 gCCGCCCCCCg 3884

ZFA-unknown-655
3875 gGCGGGGGGCg 3885
3875 cCGCCCCCCGc 3885

ZFA-unknown-656
3878 gGGGGGCGCGc 3888
3878 cCCCCCGCGCg 3888

ZFA-unknown-657
3888 cGCAGGCGCGg 3898
3888 gCGTCCGCGCc 3898

ZFA-unknown-658
3891 aGGCGCGGCGg 3901
3891 tCCGCGCCGCc 3901

ZFA-unknown-659
3894 cGCGGCGGGTg 3904
3894 gCGCCGCCCAc 3904

ZFA-unknown-660
3897 gGCGGGTGGGc 3907
3897 cCGCCCACCCg 3907

ZFA-unknown-661
3901 gGTGGGCGAAg 3911
3901 cCACCCGCTTc 3911

ZFA-unknown-662
3904 gGGCGAAGACg 3914
3904 cCCGCTTCTGc 3914

ZFA-unknown-663
3907 cGAAGACGCCg 3917
3907 gCTTCTGCGGc 3917

ZFA-unknown-664
3910 aGACGCCGCGg 3920
3910 tCTGCGGCGCc 3920

ZFA-unknown-665
3922 cGCCGCGGCGt 3912
3922 gCGGCGCCGCa 3912

ZFA-unknown-666
3913 cGCCGCGGCGg 3923
3913 gCGGCGCCGCc 3923

ZFA-unknown-667
3925 cGCCGCCGCGg 3915
3925 gCGGCGGCGCc 3915

ZFA-unknown-668
3916 cGCGGCGGCGg 3926
3916 gCGCCGCCGCc 3926

ZFA-unknown-669
3919 gGCGGCGGAGg 3929
3919 cCGCCGCCTCc 3929

ZFA-unknown-670
3921 cGGCGGAGGGc 3931
3921 gCCGCCTCCCg 3931

ZFA-unknown-671
3922 gGCGGAGGGCa 3932
3922 cCGCCTCCCGt 3932

ZFA-unknown-672
3943 gGGGGGCGTCc 3933
3943 cCCCCCGCAGg 3933

ZFA-unknown-673
3946 cGCGGGGGGCg 3936
3946 gCGCCCCCCGc 3936

ZFA-unknown-674
3957 cGGGGCTGTCt 3947
3957 gCCCCGACAGa 3947

ZFA-unknown-675
3967 cTGGGCCGCGc 3957
3967 gACCCGGCGCg 3957

ZFA-unknown-676
3978 aTGGGGGGCTc 3968
3978 tACCCCCCGAg 3968

ZFA-unknown-677
3989 cGCTGATGACt 3979
3989 gCGACTACTGa 3979

ZFA-unknown-678
3997 gGGAGAGTCGc 3987
3997 cCCTCTCAGCg 3987

ZFA-unknown-679
4000 cGGGGGAGAGt 3990
4000 gCCCCCTCTCa 3990

ZFA-unknown-680
4002 gGCGGGGGAGa 3992
4002 cCGCCCCCTCt 3992

ZFA-unknown-681
4003 cGGCGGGGGAg 3993
4003 gCCGCCCCCTc 3993

ZFA-unknown-682
4006 aGACGGCGGGg 3996
4006 tCTGCCGCCCc 3996

ZFA-unknown-683
4009 cGGAGACGGCg 3999
4009 gCCTCTGCCGc 3999

ZFA-unknown-684
4011 cGCGGAGACGg 4001
4011 gCGCCTCTGCc 4001

ZFA-unknown-685
4014 cGGCGCGGAGa 4004

4014 gCCGCGCCTCt 4004

ZFA-unknown-686
4019 cGGGGCGGCGc 4009
4019 gCCCCGCCGCg 4009

ZFA-unknown-687
4052 aGGAGGAGACa 4042
4052 tCCTCCTCTGt 4042

ZFA-unknown-688
4054 gGAGGAGGAGa 4044
4054 cCTCCTCCTCt 4044

ZFA-unknown-689
4055 aGGAGGAGGAg 4045
4055 tCCTCCTCCTc 4045

ZFA-unknown-690
4057 gGAGGAGGAGg 4047
4057 cCTCCTCCTCc 4047

ZFA-unknown-691
4058 cGGAGGAGGAg 4048
4058 gCCTCCTCCTc 4048

ZFA-unknown-692
4060 tGCGGAGGAGg 4050
4060 aCGCCTCCTCc 4050

ZFA-unknown-693
4061 gTGCGGAGGAg 4051
4061 cACGCCTCCTc 4051

ZFA-unknown-694
4063 cTGTGCGGAGg 4053
4063 gACACGCCTCc 4053

ZFA-unknown-695
4078 cGGGGGGGGAg 4088
4078 gCCCCCCCCTc 4088

ZFA-unknown-696
4079 gGGGGGGGAGg 4089
4079 cCCCCCCCTCc 4089

ZFA-unknown-697
4081 gGGGGGAGGTc 4091
4081 cCCCCCTCCAg 4091

ZFA-unknown-698
4082 gGGGGAGGTCt 4092
4082 cCCCCTCCAGa 4092

ZFA-unknown-699
4085 gGAGGTCTGCc 4095
4085 cCTCCAGACGg 4095

ZFA-unknown-700
4098 tGTGGCAGACc 4088
4098 aCACCGTCTGg 4088

ZFA-unknown-701
4105 cGACGACTGTg 4095
4105 gCTGCTGACAc 4095

ZFA-unknown-702
4098 aGTCGTCGGGg 4108
4098 tCAGCAGCCCc 4108

ZFA-unknown-703
4101 cGTCGGGGCGc 4111
4101 gCAGCCCCGCg 4111

ZFA-unknown-704
4125 cGGGGGCGCGc 4115
4125 gCCCCCGCGCg 4115

ZFA-unknown-705
4127 cGCGGGGGCGc 4117
4127 gCGCCCCCGCg 4117

ZFA-unknown-706
4126 cGCGGCCGTCg 4136
4126 gCGCCGGCAGc 4136

ZFA-unknown-707
4129 gGCCGTCGCCc 4139
4129 cCGGCAGCGGg 4139

ZFA-unknown-708
4141 cGGGGCGACGg 4131
4141 gCCCCGCTGCc 4131

ZFA-unknown-709
4161 cGGGGCGGACt 4151
4161 gCCCCGCCTGa 4151

ZFA-unknown-710
4166 cGGCGCGGGGc 4156
4166 gCCGCGCCCCg 4156

ZFA-unknown-711
4171 gGCGGCGGCGc 4161
4171 cCGCCGCCGCg 4161

ZFA-unknown-712
4162 cGCCGCCGCCg 4172
4162 gCGGCGGCGGc 4172

ZFA-unknown-713
4174 gGCGGCGGCGg 4164
4174 cCGCCGCCGCc 4164

ZFA-unknown-714
4165 cGCCGCCGCCc 4175
4165 gCGGCGGCGGg 4175

ZFA-unknown-715
4177 gGGGGCGGCGg 4167
4177 cCCCCGCCGCc 4167

ZFA-unknown-716
4178 cGGGGGCGGCg 4168
4178 gCCCCCGCCGc 4168

ZFA-unknown-717
4179 tGGTGTCTGCg 4189
4179 aCCACAGACGc 4189

ZFA-unknown-718

4192 cGCGGACGCGg 4202
4192 gCGCCTGCGCc 4202

ZFA-unknown-719
4195 gGACGCGGCCg 4205
4195 cCTGCGCCGGc 4205

ZFA-unknown-720
4198 cGCGGCCGGGc 4208
4198 gCGCCGGCCCg 4208

ZFA-unknown-721
4216 cGGCGCGGGCc 4206
4216 gCCGCGCCCGg 4206

ZFA-unknown-722
4222 gGCGGGCGGCg 4212
4222 cCGCCCGCCGc 4212

ZFA-unknown-723
4224 tGCCGGTGGAc 4234
4224 aCGGCCACCTg 4234

ZFA-unknown-724
4228 gGTGGACGCGc 4238
4228 cCACCTGCGCg 4238

ZFA-unknown-725
4283 gGGTGTCGGTc 4273
4283 cCCACAGCCAg 4273

ZFA-unknown-726
4295 cTGGGCCGGGc 4305
4295 gACCCGGCCCg 4305

ZFA-unknown-727
4303 gGCAGGCGCGa 4313
4303 cCGTCCGCGCt 4313

ZFA-unknown-728
4318 gTCGGTCGCGc 4308
4318 cAGCCAGCGCg 4308

ZFA-unknown-729
4327 gTCGGGAGGGc 4337
4327 cAGCCCTCCCg 4337

ZFA-unknown-730
4339 gGGCGCGGAGg 4349
4339 cCCGCGCCTCc 4349

ZFA-unknown-731
4342 cGCGGAGGGAg 4352
4342 gCGCCTCCCTc 4352

ZFA-unknown-732
4345 gGAGGGAGGAc 4355
4345 cCTCCCTCCTg 4355

ZFA-unknown-733
4370 cGCGGGGGACc 4360
4370 gCGCCCCCTGg 4360

ZFA-unknown-734
4373 tGCCGCGGGGg 4363
4373 aCGGCGCCCCc 4363

ZFA-unknown-735
4376 tGGTGCCGCGg 4366
4376 aCCACGGCGCc 4366

ZFA-unknown-736
4379 tGTTGGTGCCg 4369
4379 aCAACCACGGc 4369

ZFA-unknown-737
4382 gGGTGTTGGTg 4372
4382 cCCACAACCAc 4372

ZFA-unknown-738
4385 cGGGGGTGTTg 4375
4385 gCCCCCACAAc 4375

ZFA-unknown-739
4384 cGGTGCCGCCc 4394
4384 gCCACGGCGGg 4394

ZFA-unknown-740
4396 gGGGGCGGCAc 4386
4396 cCCCCGCCGTg 4386

ZFA-unknown-741
4397 gGGGGGCGGCa 4387
4397 cCCCCCGCCGt 4387

ZFA-unknown-742
4399 gTGGGGGGCGg 4389
4399 cACCCCCCGCc 4389

ZFA-unknown-743
4400 cGTGGGGGGCg 4390
4400 gCACCCCCCGc 4390

ZFA-unknown-744
4408 cTCCGCGGCGt 4398
4408 gAGGCGCCGCa 4398

ZFA-unknown-745
4399 cGCCGCGGAGg 4409
4399 gCGGCGCCTCc 4409

ZFA-unknown-746
4402 cGCGGAGGGGg 4412
4402 gCGCCTCCCCc 4412

ZFA-unknown-747
4404 cGGAGGGGGCg 4414
4404 gCCTCCCCCGc 4414

ZFA-unknown-748
4405 gGAGGGGGCGg 4415
4405 cCTCCCCCGCc 4415

ZFA-unknown-749
4407 aGGGGGCGGCg 4417
4407 tCCCCCGCCGc 4417

ZFA-unknown-750
4408 gGGGGCGGCGg 4418
4408 cCCCCGCCGCc 4418

ZFA-unknown-751
4420 gGCCGCCGCCc 4410
4420 cCGGCGGCGGg 4410

ZFA-unknown-752
4411 gGCGGCGGCCc 4421
4411 cCGCCGCCGGg 4421

ZFA-unknown-753
4427 gGGGGCGGGCc 4417
4427 cCCCCGCCCGg 4417

ZFA-unknown-754
4428 cGGGGGCGGGc 4418
4428 gCCCCCGCCCg 4418

ZFA-unknown-755
4428 gGAAGAGGCGc 4438
4428 cCTTCTCCGCg 4438

ZFA-unknown-756
4433 aGGCGCGGGTc 4443
4433 tCCGCGCCCAg 4443

ZFA-unknown-757
4462 gGCCGCGGGGc 4452
4462 cCGGCGCCCCg 4452

ZFA-unknown-758
4465 cGAGGCCGCGg 4455
4465 gCTCCGGCGCc 4455

ZFA-unknown-759
4468 gGACGAGGCCg 4458
4468 cCTGCTCCGGc 4458

ZFA-unknown-760
4471 gGAGGACGAGg 4461
4471 cCTCCTGCTCc 4461

ZFA-unknown-761
4474 gGCGGAGGACg 4464
4474 cCGCCTCCTGc 4464

ZFA-unknown-762
4475 aGGCGGAGGAc 4465
4475 tCCGCCTCCTg 4465

ZFA-unknown-763
4477 aGAGGCGGAGg 4467
4477 tCTCCGCCTCc 4467

ZFA-unknown-764
4480 gGAAGAGGCGg 4470
4480 cCTTCTCCGCc 4470

ZFA-unknown-765
4483 gGAGGAAGAGg 4473
4483 cCTCCTTCTCc 4473

ZFA-unknown-766
4486 gGCGGAGGAAg 4476
4486 cCGCCTCCTTc 4476

ZFA-unknown-767
4487 cGGCGGAGGAa 4477

4487 gCCGCCTCCTt 4477

ZFA-unknown-768
4489 gGCGGCGGAGg 4479
4489 cCGCCGCCTCc 4479

ZFA-unknown-769
4492 cGGGGCGGCGg 4482
4492 gCCCCGCCGCc 4482

ZFA-unknown-770
4508 gGGCGAGGGGc 4498
4508 cCCGCTCCCCg 4498

ZFA-unknown-771
4510 gGGGGCGAGGg 4500
4510 cCCCCGCTCCc 4500

ZFA-unknown-772
4513 cTGGGGGGCGa 4503
4513 gACCCCCCGCt 4503

ZFA-unknown-773
4512 aGGGGGTGGGg 4522
4512 tCCCCCACCCc 4522

ZFA-unknown-774
4513 gGGGGTGGGGg 4523
4513 cCCCCACCCCc 4523

ZFA-unknown-775
4515 gGGTGGGGGCc 4525
4515 cCCACCCCCGg 4525

ZFA-unknown-776
4516 gGTGGGGGCCa 4526
4516 cCACCCCCGGt 4526

ZFA-unknown-777
4527 aGAGGGCGGCg 4537
4527 tCTCCCGCCGc 4537

ZFA-unknown-778
4540 cGGCGCCGCCc 4530
4540 gCCGCGGCGGg 4530

ZFA-unknown-779
4533 cGGCGCCGCGc 4543
4533 gCCGCGGCGCg 4543

ZFA-unknown-780
4545 cGGCGCGGCGc 4535
4545 gCCGCGCCGCg 4535

ZFA-unknown-781
4561 cTCGGGCGACc 4571
4561 gAGCCCGCTGg 4571

ZFA-unknown-782
4574 cGCGGTCGCCc 4564
4574 gCGCCAGCGGg 4564

ZFA-unknown-783
4577 gGCCGCGGTCg 4567
4577 cCGGCGCCAGc 4567

ZFA-unknown-784
4580 cGTGGCCGCGg 4570
4580 gCACCGGCGCc 4570

ZFA-unknown-785
4597 cGCCGGGGCGa 4587
4597 gCGGCCCCGCt 4587

ZFA-unknown-786
4600 gGACGCCGGGg 4590
4600 cCTGCGGCCCc 4590

ZFA-unknown-787
4603 cGCGGACGCCg 4593
4603 gCGCCTGCGGc 4593

ZFA-unknown-788
4597 gTCCGCGGGCg 4607
4597 cAGGCGCCCGc 4607

ZFA-unknown-789
4600 cGCGGGCGCCg 4610
4600 gCGCCCGCGGc 4610

ZFA-unknown-790
4603 gGGCGCCGCGc 4613
4603 cCCGCGGCGCg 4613

ZFA-unknown-791
4615 gGGCGCGGCGc 4605
4615 cCCGCGCCGCg 4605

ZFA-unknown-792
4618 cGGGGGCGCGg 4608
4618 gCCCCCGCGCc 4608

ZFA-unknown-793
4621 cGACGGGGGCg 4611
4621 gCTGCCCCCGc 4611

ZFA-unknown-794
4624 cGCCGACGGGg 4614
4624 gCGGCTGCCCc 4614

ZFA-unknown-796
4627 aGACGCCGACg 4617
4627 tCTGCGGCTGc 4617

ZFA-unknown-795
4617 cGTCGGCGTCt 4627
4617 gCAGCCGCAGa 4627

ZFA-unknown-797
4630 cGGAGACGCCg 4620
4630 gCCTCTGCGGc 4620

ZFA-unknown-798
4633 cGACGGAGACg 4623
4633 gCTGCCTCTGc 4623

ZFA-unknown-799
4636 gGACGACGGAg 4626
4636 cCTGCTGCCTc 4626

ZFA-unknown-800

4639 cTGGGACGACg 4629
4639 gACCCTGCTGc 4629

ZFA-unknown-801
4639 gGCCGCGGTCg 4649
4639 cCGGCGCCAGc 4649

ZFA-unknown-802
4642 cGCGGTCGCCg 4652
4642 gCGCCAGCGGc 4652

ZFA-unknown-803
4645 gGTCGCCGCCg 4655
4645 cCAGCGGCGGc 4655

ZFA-unknown-804
4657 gGCGGCGGCGa 4647
4657 cCGCCGCCGCt 4647

ZFA-unknown-805
4648 cGCCGCCGCCt 4658
4648 gCGGCGGCGGa 4658

ZFA-unknown-806
4660 gGAGGCGGCGg 4650
4660 cCTCCGCCGCc 4650

ZFA-unknown-807
4661 aGGAGGCGGCg 4651
4661 tCCTCCGCCGc 4651

ZFA-unknown-808
4663 gGAGGAGGCGg 4653
4663 cCTCCTCCGCc 4653

ZFA-unknown-809
4664 aGGAGGAGGCg 4654
4664 tCCTCCTCCGc 4654

ZFA-unknown-810
4666 gGAGGAGGAGg 4656
4666 cCTCCTCCTCc 4656

ZFA-unknown-811
4667 aGGAGGAGGAg 4657
4667 tCCTCCTCCTc 4657

ZFA-unknown-812
4669 gGAGGAGGAGg 4659
4669 cCTCCTCCTCc 4659

ZFA-unknown-813
4670 cGGAGGAGGAg 4660
4670 gCCTCCTCCTc 4660

ZFA-unknown-814
4672 gGCGGAGGAGg 4662
4672 cCGCCTCCTCc 4662

ZFA-unknown-815
4673 aGGCGGAGGAg 4663
4673 tCCGCCTCCTc 4663

ZFA-unknown-816
4675 gGAGGCGGAGg 4665
4675 cCTCCGCCTCc 4665

ZFA-unknown-817
4676 aGGAGGCGGAg 4666
4676 tCCTCCGCCTc 4666

ZFA-unknown-818
4678 gGAGGAGGCGg 4668
4678 cCTCCTCCGCc 4668

ZFA-unknown-819
4679 aGGAGGAGGCg 4669
4679 tCCTCCTCCGc 4669

ZFA-unknown-820
4681 gGAGGAGGAGg 4671
4681 cCTCCTCCTCc 4671

ZFA-unknown-821
4682 aGGAGGAGGAg 4672
4682 tCCTCCTCCTc 4672

ZFA-unknown-822
4684 gGAGGAGGAGg 4674
4684 cCTCCTCCTCc 4674

ZFA-unknown-823
4685 cGGAGGAGGAg 4675
4685 gCCTCCTCCTc 4675

ZFA-unknown-824
4687 gGCGGAGGAGg 4677
4687 cCGCCTCCTCc 4677

ZFA-unknown-825
4688 aGGCGGAGGAg 4678
4688 tCCGCCTCCTc 4678

ZFA-unknown-826
4690 gGAGGCGGAGg 4680
4690 cCTCCGCCTCc 4680

ZFA-unknown-827
4691 aGGAGGCGGAg 4681
4691 tCCTCCGCCTc 4681

ZFA-unknown-828
4693 gGAGGAGGCGg 4683
4693 cCTCCTCCGCc 4683

ZFA-unknown-829
4694 aGGAGGAGGCg 4684
4694 tCCTCCTCCGc 4684

ZFA-unknown-830
4696 gGAGGAGGAGg 4686
4696 cCTCCTCCTCc 4686

ZFA-unknown-831
4697 aGGAGGAGGAg 4687
4697 tCCTCCTCCTc 4687

ZFA-unknown-832
4699 gGAGGAGGAGg 4689
4699 cCTCCTCCTCc 4689

ZFA-unknown-833
4700 cGGAGGAGGAg 4690
4700 gCCTCCTCCTc 4690

ZFA-unknown-834
4702 gGCGGAGGAGg 4692
4702 cCGCCTCCTCc 4692

ZFA-unknown-835
4703 aGGCGGAGGAg 4693
4703 tCCGCCTCCTc 4693

ZFA-unknown-836
4705 gGAGGCGGAGg 4695
4705 cCTCCGCCTCc 4695

ZFA-unknown-837
4706 aGGAGGCGGAg 4696
4706 tCCTCCGCCTc 4696

ZFA-unknown-838
4708 gGAGGAGGCGg 4698
4708 cCTCCTCCGCc 4698

ZFA-unknown-839
4709 aGGAGGAGGCg 4699
4709 tCCTCCTCCGc 4699

ZFA-unknown-840
4711 gGAGGAGGAGg 4701
4711 cCTCCTCCTCc 4701

ZFA-unknown-841
4712 aGGAGGAGGAg 4702
4712 tCCTCCTCCTc 4702

ZFA-unknown-842
4714 gGAGGAGGAGg 4704
4714 cCTCCTCCTCc 4704

ZFA-unknown-843
4715 cGGAGGAGGAg 4705
4715 gCCTCCTCCTc 4705

ZFA-unknown-844
4717 gGCGGAGGAGg 4707
4717 cCGCCTCCTCc 4707

ZFA-unknown-845
4718 aGGCGGAGGAg 4708
4718 tCCGCCTCCTc 4708

ZFA-unknown-846
4720 gGAGGCGGAGg 4710
4720 cCTCCGCCTCc 4710

ZFA-unknown-847
4721 aGGAGGCGGAg 4711
4721 tCCTCCGCCTc 4711

ZFA-unknown-848
4723 gGAGGAGGCGg 4713
4723 cCTCCTCCGCc 4713

ZFA-unknown-849
4724 aGGAGGAGGCg 4714

4724 tCCTCCTCCGc 4714

ZFA-unknown-850
4726 gGAGGAGGAGg 4716
4726 cCTCCTCCTCc 4716

ZFA-unknown-851
4727 aGGAGGAGGAg 4717
4727 tCCTCCTCCTc 4717

ZFA-unknown-852
4729 gGAGGAGGAGg 4719
4729 cCTCCTCCTCc 4719

ZFA-unknown-853
4730 cGGAGGAGGAg 4720
4730 gCCTCCTCCTc 4720

ZFA-unknown-854
4732 gGCGGAGGAGg 4722
4732 cCGCCTCCTCc 4722

ZFA-unknown-855
4733 aGGCGGAGGAg 4723
4733 tCCGCCTCCTc 4723

ZFA-unknown-856
4735 gGAGGCGGAGg 4725
4735 cCTCCGCCTCc 4725

ZFA-unknown-857
4736 aGGAGGCGGAg 4726
4736 tCCTCCGCCTc 4726

ZFA-unknown-858
4738 gGAGGAGGCGg 4728
4738 cCTCCTCCGCc 4728

ZFA-unknown-859
4739 aGGAGGAGGCg 4729
4739 tCCTCCTCCGc 4729

ZFA-unknown-860
4741 gGAGGAGGAGg 4731
4741 cCTCCTCCTCc 4731

ZFA-unknown-861
4742 aGGAGGAGGAg 4732
4742 tCCTCCTCCTc 4732

ZFA-unknown-862
4744 gGAGGAGGAGg 4734
4744 cCTCCTCCTCc 4734

ZFA-unknown-863
4745 cGGAGGAGGAg 4735
4745 gCCTCCTCCTc 4735

ZFA-unknown-864
4747 gGCGGAGGAGg 4737
4747 cCGCCTCCTCc 4737

ZFA-unknown-865
4748 aGGCGGAGGAg 4738
4748 tCCGCCTCCTc 4738

ZFA-unknown-866
4750 gGAGGCGGAGg 4740
4750 cCTCCGCCTCc 4740

ZFA-unknown-867
4751 aGGAGGCGGAg 4741
4751 tCCTCCGCCTc 4741

ZFA-unknown-868
4753 gGAGGAGGCGg 4743
4753 cCTCCTCCGCc 4743

ZFA-unknown-869
4754 aGGAGGAGGCg 4744
4754 tCCTCCTCCGc 4744

ZFA-unknown-870
4756 gGAGGAGGAGg 4746
4756 cCTCCTCCTCc 4746

ZFA-unknown-871
4757 aGGAGGAGGAg 4747
4757 tCCTCCTCCTc 4747

ZFA-unknown-872
4759 gGAGGAGGAGg 4749
4759 cCTCCTCCTCc 4749

ZFA-unknown-873
4760 cGGAGGAGGAg 4750
4760 gCCTCCTCCTc 4750

ZFA-unknown-874
4762 gGCGGAGGAGg 4752
4762 cCGCCTCCTCc 4752

ZFA-unknown-875
4763 aGGCGGAGGAg 4753
4763 tCCGCCTCCTc 4753

ZFA-unknown-876
4765 aGAGGCGGAGg 4755
4765 tCTCCGCCTCc 4755

ZFA-unknown-877
4768 gGAAGAGGCGg 4758
4768 cCTTCTCCGCc 4758

ZFA-unknown-878
4771 aGAGGAAGAGg 4761
4771 tCTCCTTCTCc 4761

ZFA-unknown-879
4774 cGCAGAGGAAg 4764
4774 gCGTCTCCTTc 4764

ZFA-unknown-880
4771 tGCGGGCGGGg 4781
4771 aCGCCCGCCCc 4781

ZFA-unknown-881
4774 gGGCGGGGCTg 4784
4774 cCCGCCCCGAc 4784

ZFA-unknown-882

4777 cGGGGCTGGTg 4787
4777 gCCCCGACCAc 4787

ZFA-unknown-883
4780 gGCTGGTGGGa 4790
4780 cCGACCACCCt 4790

ZFA-unknown-884
4784 gGTGGGAGCGt 4794
4784 cCACCCTCGCa 4794

ZFA-unknown-885
4787 gGGAGCGTCGc 4797
4787 cCCTCGCAGCg 4797

ZFA-unknown-886
4801 gGACGCGACGc 4791
4801 cCTGCGCTGCg 4791

ZFA-unknown-887
4804 cGCGGACGCGa 4794
4804 gCGCCTGCGCt 4794

ZFA-unknown-888
4807 gGACGCGGACg 4797
4807 cCTGCGCCTGc 4797

ZFA-unknown-889
4807 cGGCGCTGGGg 4817
4807 gCCGCGACCCc 4817

ZFA-unknown-890
4810 cGCTGGGGAGa 4820
4810 gCGACCCCTCt 4820

ZFA-unknown-891
4812 cTGGGGAGAGa 4822
4812 gACCCCTCTCt 4822

ZFA-unknown-892
4815 gGGAGAGACGa 4825
4815 cCCTCTCTGCt 4825

ZFA-unknown-893
4838 cGAGGGAGGTt 4828
4838 gCTCCCTCCAa 4828

ZFA-unknown-894
4841 gGCCGAGGGAg 4831
4841 cCGGCTCCCTc 4831

ZFA-unknown-895
4844 gGGGGCCGAGg 4834
4844 cCCCCGGCTCc 4834

ZFA-unknown-896
4847 cGCGGGGGCCg 4837
4847 gCGCCCCCGGc 4837

ZFA-unknown-897
4855 cGCAGCAGCGc 4845
4855 gCGTCGTCGCg 4845

ZFA-unknown-898
4846 cGCTGCTGCGc 4856
4846 gCGACGACGCg 4856

ZFA-unknown-899
4858 cGGCGCAGCAg 4848
4858 gCCGCGTCGTc 4848

ZFA-unknown-900
4851 cTGCGCCGCGg 4861
4851 gACGCGGCGCc 4861

ZFA-unknown-901
4854 cGCCGCGGGGg 4864
4854 gCGGCGCCCCc 4864

ZFA-unknown-902
4857 cGCGGGGGCCg 4867
4857 gCGCCCCCGGc 4867

ZFA-unknown-903
4860 gGGGGCCGAGg 4870
4860 cCCCCGGCTCc 4870

ZFA-unknown-904
4863 gGCCGAGGAAg 4873
4863 cCGGCTCCTTc 4873

ZFA-unknown-905
4866 cGAGGAAGTGt 4876
4866 gCTCCTTCACa 4876

ZFA-unknown-906
4881 gGAAGACGCGc 4891
4881 cCTTCTGCGCg 4891

ZFA-unknown-907
4894 cGCGGAGGGCg 4904
4894 gCGCCTCCCGc 4904

ZFA-unknown-908
4897 gGAGGGCGGCc 4907
4897 cCTCCCGCCGg 4907

ZFA-unknown-909
4910 cGGGGCCGCCc 4900
4910 gCCCCGGCGGg 4900

ZFA-unknown-910
4928 gGTCGCGGGCc 4918
4928 cCAGCGCCCGg 4918

ZFA-unknown-911
4933 cGCCGGGTCGc 4923
4933 gCGGCCCAGCg 4923

ZFA-unknown-912
4936 gGGCGCCGGGt 4926
4936 cCCGCGGCCCa 4926

ZFA-unknown-913
4943 tGAGGCCGGGc 4933
4943 aCTCCGGCCCg 4933

ZFA-unknown-914
4959 aTGGGCAGGTa 4949
4959 tACCCGTCCAt 4949

ZFA-unknown-915
4960 cGCGGGGGTCt 4970
4960 gCGCCCCCAGa 4970

ZFA-unknown-916
4975 cGTCGTGGCCc 4985
4975 gCAGCACCGGg 4985

ZFA-unknown-917
5014 gGGGGACTGCc 5024
5014 cCCCCTGACGg 5024

ZFA-unknown-918
5040 tGGAGACGGGc 5050
5040 aCCTCTGCCCg 5050

ZFA-unknown-919
5082 aGACGGGGAAc 5092
5082 tCTGCCCCTTg 5092

ZFA-unknown-920
5092 cGTGGCGGACc 5102
5092 gCACCGCCTGg 5102

ZFA-unknown-921
5109 cGCAGCAGGTc 5099
5109 gCGTCGTCCAg 5099

ZFA-unknown-922
5103 tGCTGCGGGCc 5113
5103 aCGACGCCCGg 5113

ZFA-unknown-923
5119 gGCCGCGGCCc 5109
5119 cCGGCGCCGGg 5109

ZFA-unknown-924
5110 gGCCGCGGCCc 5120
5110 cCGGCGCCGGg 5120

ZFA-unknown-925
5122 gGGGGCCGCGg 5112
5122 cCCCCGGCGCc 5112

ZFA-unknown-926
5125 cGCGGGGGCCg 5115
5125 gCGCCCCCGGc 5115

ZFA-unknown-927
5126 tGGAGCCGCCg 5136
5126 aCCTCGGCGGc 5136

ZFA-unknown-928
5138 tGCGGCGGCTc 5128
5138 aCGCCGCCGAg 5128

ZFA-unknown-929
5129 aGCCGCCGCAc 5139
5129 tCGGCGGCGTg 5139

ZFA-unknown-930
5141 gGGTGCGGCGg 5131
5141 cCCACGCCGCc 5131

ZFA-unknown-931
5145 aGCAGGGTGCg 5135

5145 tCGTCCCACGc 5135

ZFA-unknown-932
5148 gGGAGCAGGGt 5138
5148 cCCTCGTCCCa 5138

ZFA-unknown-933
5168 cGCAGTTGCGc 5158
5168 gCGTCAACGCg 5158

ZFA-unknown-934
5182 gTCGGGGGGCc 5172
5182 cAGCCCCCCGg 5172

ZFA-unknown-935
5183 aGTCGGGGGGc 5173
5183 tCAGCCCCCCg 5173

ZFA-unknown-936
5186 gGTAGTCGGGg 5176
5186 cCATCAGCCCc 5176

ZFA-unknown-937
5196 gGGGGCGTCGg 5186
5196 cCCCCGCAGCc 5186

ZFA-unknown-938
5197 gGGGGGCGTCg 5187
5197 cCCCCCGCAGc 5187

ZFA-unknown-939
5200 cGCGGGGGGCg 5190
5200 gCGCCCCCCGc 5190

ZFA-unknown-940
5203 cGACGCGGGGg 5193
5203 gCTGCGCCCCc 5193

ZFA-unknown-941
5199 cGTCGGAGTGg 5209
5199 gCAGCCTCACc 5209

ZFA-unknown-942
5202 cGGAGTGGAAc 5212
5202 gCCTCACCTTg 5212

ZFA-unknown-943
5203 gGAGTGGAACa 5213
5203 cCTCACCTTGt 5213

ZFA-unknown-944
5219 aGAGGCTGTTc 5209
5219 tCTCCGACAAg 5209

ZFA-unknown-945
5263 gGTGGGCGCGc 5273
5263 cCACCCGCGCg 5273

ZFA-unknown-946
5285 cGTGGAAGTCc 5275
5285 gCACCTTCAGg 5275

ZFA-unknown-947
5288 gGCCGTGGAAg 5278
5288 cCGGCACCTTc 5278

ZFA-unknown-948
5291 gGAGGCCGTGg 5281
5291 cCTCCGGCACc 5281

ZFA-unknown-949
5304 gGGTGGCGCGa 5294
5304 cCCACCGCGCt 5294

ZFA-unknown-950
5310 cTCGGGAGCAg 5320
5310 gAGCCCTCGTc 5320

ZFA-unknown-951
5313 gGGAGCAGGGc 5323
5313 cCCTCGTCCCg 5323

ZFA-unknown-952
5332 cGCGGGCGCGc 5322
5332 gCGCCCGCGCg 5322

ZFA-unknown-953
5335 cGGCGCGGGCg 5325
5335 gCCGCGCCCGc 5325

ZFA-unknown-954
5338 gGCCGGCGCGg 5328
5338 cCGGCCGCGCc 5328

ZFA-unknown-956
5345 cGTCGCCGGCc 5335
5345 gCAGCGGCCGg 5335

ZFA-unknown-955
5335 gGCCGGCGACg 5345
5335 cCGGCCGCTGc 5345

ZFA-unknown-957
5350 gGGGGCGTCGc 5340
5350 cCCCCGCAGCg 5340

ZFA-unknown-958
5351 cGGGGGCGTCg 5341
5351 gCCCCCGCAGc 5341

ZFA-unknown-959
5353 cGCGGGGGCGt 5343
5353 gCGCCCCCGCa 5343

ZFA-unknown-960
5361 gGGAGTAGGGg 5371
5361 cCCTCATCCCc 5371

ZFA-unknown-961
5364 aGTAGGGGGAg 5374
5364 tCATCCCCCTc 5374

ZFA-unknown-962
5365 gTAGGGGGAGc 5375
5365 cATCCCCCTCg 5375

ZFA-unknown-963
5367 aGGGGGAGCTa 5377
5367 tCCCCCTCGAt 5377

ZFA-unknown-964

5387 aGCCGAGTGTt 5377
5387 tCGGCTCACAa 5377

ZFA-unknown-965
5399 gGAAGCCGCCc 5409
5399 cCTTCGGCGGg 5409

ZFA-unknown-966
5411 gGGGGCGGCTt 5401
5411 cCCCCGCCGAa 5401

ZFA-unknown-967
5412 gGGGGGCGGCt 5402
5412 cCCCCCGCCGa 5402

ZFA-unknown-968
5414 gTGGGGGGCGg 5404
5414 cACCCCCCGCc 5404

ZFA-unknown-969
5415 gGTGGGGGGCg 5405
5415 cCACCCCCCGc 5405

ZFA-unknown-970
5416 cGGTGGGGGGc 5406
5416 gCCACCCCCCg 5406

ZFA-unknown-971
5427 gGCCGGTGGTc 5417
5427 cCGGCCACCAg 5417

ZFA-unknown-972
5430 cTCGGCCGGTg 5420
5430 gAGCCGGCCAc 5420

ZFA-unknown-973
5424 gGCCGAGGCGc 5434
5424 cCGGCTCCGCg 5434

ZFA-unknown-974
5435 cTCGGGGGCAg 5445
5435 gAGCCCCCGTc 5445

ZFA-unknown-975
5438 gGGGGCAGGGg 5448
5438 cCCCCGTCCCc 5448

ZFA-unknown-976
5441 gGCAGGGGGAg 5451
5441 cCGTCCCCCTc 5451

ZFA-unknown-977
5444 aGGGGGAGGTg 5454
5444 tCCCCCTCCAc 5454

ZFA-unknown-978
5445 gGGGGAGGTGg 5455
5445 cCCCCTCCACc 5455

ZFA-unknown-979
5447 gGGAGGTGGGg 5457
5447 cCCTCCACCCc 5457

ZFA-unknown-980
5448 gGAGGTGGGGg 5458
5448 cCTCCACCCCc 5458

ZFA-unknown-981
5450 aGGTGGGGGGg 5460
5450 tCCACCCCCCc 5460

ZFA-unknown-982
5451 gGTGGGGGGGg 5461
5451 cCACCCCCCCc 5461

ZFA-unknown-983
5452 gTGGGGGGGGg 5462
5452 cACCCCCCCCc 5462

ZFA-unknown-984
5453 tGGGGGGGGGg 5463
5453 aCCCCCCCCCc 5463

ZFA-unknown-985
5454 gGGGGGGGGGa 5464
5454 cCCCCCCCCCt 5464

ZFA-unknown-986
5455 gGGGGGGGGAa 5465
5455 cCCCCCCCCTt 5465

ZFA-unknown-987
5456 gGGGGGGGAAa 5466
5456 cCCCCCCCTTt 5466

ZFA-unknown-988
5466 aGACGGGGAGg 5476
5466 tCTGCCCCTCc 5476

ZFA-unknown-989
5469 cGGGGAGGAGa 5479
5469 gCCCCTCCTCt 5479

ZFA-unknown-990
5471 gGGAGGAGACa 5481
5471 cCCTCCTCTGt 5481

ZFA-unknown-991
5474 aGGAGACAGGa 5484
5474 tCCTCTGTCCt 5484

ZFA-unknown-992
5482 gGAAGTGGGGg 5492
5482 cCTTCACCCCc 5492

ZFA-unknown-993
5485 aGTGGGGTGg 5495
5485 tCACCCCCACc 5495

ZFA-unknown-994
5487 tGGGGGTGGGa 5497
5487 aCCCCCACCCt 5497

ZFA-unknown-995
5488 gGGGGTGGGAg 5498
5488 cCCCCACCCTc 5498

ZFA-unknown-996
5491 gGTGGGAGTGg 5501
5491 cCACCCTCACc 5501

ZFA-unknown-997
5494 gGGAGTGGGGg 5504
5494 cCCTCACCCCc 5504

ZFA-unknown-998
5497 aGTGGGGGGGg 5507
5497 tCACCCCCCCc 5507

ZFA-unknown-999
5498 gTGGGGGGGGg 5508
5498 cACCCCCCCCc 5508

ZFA-unknown-1000
5499 tGGGGGGGGGg 5509
5499 aCCCCCCCCCc 5509

ZFA-unknown-1001
5500 gGGGGGGGGGg 5510
5500 cCCCCCCCCCc 5510

ZFA-unknown-1002
5501 gGGGGGGGGGa 5511
5501 cCCCCCCCCCt 5511

ZFA-unknown-1003
5502 gGGGGGGGGAc 5512
5502 cCCCCCCCCTg 5512

ZFA-unknown-1004
5503 gGGGGGGGACg 5513
5503 cCCCCCCCTGc 5513

ZFA-unknown-1005
5504 gGGGGGGACGg 5514
5504 cCCCCCCTGCc 5514

ZFA-unknown-1006
5506 gGGGGACGGAc 5516
5506 cCCCCTGCCTg 5516

ZFA-unknown-1007
5525 cGGGGCCGTGt 5515
5525 gCCCCGGCACa 5515

ZFA-unknown-1008
5535 tGTTGCTGTTc 5525
5535 aCAACGACAAg 5525

ZFA-unknown-1009
5538 gTGTGTTGCTg 5528
5538 cACACAACGAc 5528

ZFA-unknown-1010
5542 tGGTGTGTGTt 5532
5542 aCCACACACAa 5532

ZFA-unknown-1011
5545 tGCTGGTGTGt 5535
5545 aCGACCACACa 5535

ZFA-unknown-1012
5569 tGTTGAAAACt 5579
5569 aCAACTTTTGa 5579

ZFA-unknown-1013
5627 tTGTGATGCTt 5637

5627 aACACTACGAa 5637

ZFA-unknown-1014
5646 cTTTGTTGTTg 5656
5646 gAAACAACAAc 5656

ZFA-unknown-1015
5649 tGTTGTTGGCc 5659
5649 aCAACAACCGg 5659

ZFA-unknown-1016
5662 tGATGCAGCTc 5672
5662 aCTACGTCGAg 5672

ZFA-unknown-1017
5677 cGGGGGAGCTg 5667
5677 gCCCCCTCGAc 5667

ZFA-unknown-1018
5679 cGCGGGGGAGc 5669
5679 gCGCCCCCTCg 5669

ZFA-unknown-1019
5682 cTGCGCGGGGg 5672
5682 gACGCGCCCCc 5672

ZFA-unknown-1020
5678 cGCAGGGGGGc 5688
5678 gCGTCCCCCCg 5688

ZFA-unknown-1021
5683 gGGGGCCGGGg 5693
5683 cCCCCGGCCCc 5693

ZFA-unknown-1022
5689 cGGGGATGGGg 5699
5689 gCCCCTACCCc 5699

ZFA-unknown-1023
5692 gGATGGGGGGg 5702
5692 cCTACCCCCCc 5702

ZFA-unknown-1024
5694 aTGGGGGGGAa 5704
5694 tACCCCCCCTt 5704

ZFA-unknown-1025
5695 tGGGGGGGAAg 5705
5695 aCCCCCCCTTc 5705

ZFA-unknown-1026
5697 gGGGGGAAGGg 5707
5697 cCCCCCTTCCc 5707

ZFA-unknown-1027
5698 gGGGGAAGGGa 5708
5698 cCCCCTTCCCt 5708

ZFA-unknown-1028
5705 gGGAGGAGGAg 5715
5705 cCCTCCTCCTc 5715

ZFA-unknown-1029
5706 gGAGGAGGAGg 5716
5706 cCTCCTCCTCc 5716

ZFA-unknown-1030
5708 aGGAGGAGGAg 5718
5708 tCCTCCTCCTc 5718

ZFA-unknown-1031
5709 gGAGGAGGAGg 5719
5709 cCTCCTCCTCc 5719

ZFA-unknown-1032
5711 aGGAGGAGGGg 5721
5711 tCCTCCTCCCc 5721

ZFA-unknown-1033
5712 gGAGGAGGGGg 5722
5712 cCTCCTCCCCc 5722

ZFA-unknown-1034
5714 aGGAGGGGGGg 5724
5714 tCCTCCCCCCc 5724

ZFA-unknown-1035
5715 gGAGGGGGGGc 5725
5715 cCTCCCCCCCg 5725

ZFA-unknown-1036
5717 aGGGGGGGCGg 5727
5717 tCCCCCCCGCc 5727

ZFA-unknown-1037
5719 gGGGGGCGGGc 5729
5719 cCCCCCGCCCg 5729

ZFA-unknown-1038
5720 gGGGGCGGGCa 5730
5720 cCCCCGCCCGt 5730

ZFA-unknown-1039
5733 aGAAGCCGCCc 5743
5733 tCTTCGGCGGg 5743

ZFA-unknown-1040
5745 gGGGGCGGCTt 5735
5745 cCCCCGCCGAa 5735

ZFA-unknown-1041
5746 tGGGGGCGGCt 5736
5746 aCCCCCGCCGa 5736

ZFA-unknown-1042
5748 gGTGGGGGCGg 5738
5748 cCACCCCCGCc 5738

ZFA-unknown-1043
5749 gGGTGGGGGCg 5739
5749 cCCACCCCCGc 5739

ZFA-unknown-1044
5751 gGGGGTGGGGg 5741
5751 cCCCCACCCCc 5741

ZFA-unknown-1045
5752 cGGGGGTGGGg 5742
5752 gCCCCCACCCc 5742

ZFA-unknown-1046

5754 cTCGGGGGTGg 5744
5754 gAGCCCCCACc 5744

ZFA-unknown-1047
5751 cGAGGCCTGTt 5761
5751 gCTCCGGACAa 5761

ZFA-unknown-1048
5781 aGCCGGGGCCc 5791
5781 tCGGCCCCGGg 5791

ZFA-unknown-1049
5798 cGAGGCCGGGc 5788
5798 gCTCCGGCCCg 5788

ZFA-unknown-1050
5816 gGTGGGTGGGc 5826
5816 cCACCCACCCg 5826

ZFA-unknown-1051
5817 gTGGGTGGGCg 5827
5817 cACCCACCCGc 5827

ZFA-unknown-1052
5820 gGTGGGCGGGc 5830
5820 cCACCCGCCCg 5830

ZFA-unknown-1053
5821 gTGGGCGGGCt 5831
5821 cACCCGCCCGa 5831

ZFA-unknown-1054
5861 tGTTGACAGGg 5871
5861 aCAACTGTCCc 5871

ZFA-unknown-1055
5869 gGGAGGGGGGc 5879
5869 cCCTCCCCCCg 5879

ZFA-unknown-1056
5870 gGAGGGGGGCc 5880
5870 cCTCCCCCCGg 5880

ZFA-unknown-1057
5919 tGGGGGTGGTg 5929
5919 aCCCCCACCAc 5929

ZFA-unknown-1058
5920 gGGGGTGGTGg 5930
5920 cCCCCACCACc 5930

ZFA-unknown-1059
5922 gGGTGGTGGGc 5932
5922 cCCACCACCCg 5932

ZFA-unknown-1060
5923 gGTGGTGGGCa 5933
5923 cCACCACCCGt 5933

ZFA-unknown-1061
5927 gTGGGCAGGAg 5937
5927 cACCCGTCCTc 5937

ZFA-unknown-1062
5930 gGCAGGAGTGg 5940
5930 cCGTCCTCACc 5940

ZFA-unknown-1063
5933 aGGAGTGGGAg 5943
5933 tCCTCACCCTc 5943

ZFA-unknown-1064
5936 aGTGGGAGGGc 5946
5936 tCACCCTCCCg 5946

ZFA-unknown-1065
5937 gTGGGAGGGCg 5947
5937 cACCCTCCCGc 5947

ZFA-unknown-1066
5940 gGAGGGCGCCt 5950
5940 cCTCCCGCGGa 5950

ZFA-unknown-1067
5955 cGGGGAGGGAg 5965
5955 gCCCCTCCCTc 5965

ZFA-unknown-1068
5958 gGAGGGAGGAg 5968
5958 cCTCCCTCCTc 5968

ZFA-unknown-1069
5961 gGGAGGAGGGg 5971
5961 cCCTCCTCCCc 5971

ZFA-unknown-1070
5962 gGAGGAGGGGg 5972
5962 cCTCCTCCCCc 5972

ZFA-unknown-1071
5964 aGGAGGGGGGg 5974
5964 tCCTCCCCCCc 5974

ZFA-unknown-1072
5965 gGAGGGGGGGg 5975
5965 cCTCCCCCCCc 5975

ZFA-unknown-1073
5967 aGGGGGGGGGt 5977
5967 tCCCCCCCCCa 5977

ZFA-unknown-1074
5968 gGGGGGGGGTc 5978
5968 cCCCCCCCCAg 5978

ZFA-unknown-1075
5969 gGGGGGGGTCa 5979
5969 cCCCCCCCAGt 5979

ZFA-unknown-1076
5985 tGCGGCTGACc 5975
5985 aCGCCGACTGg 5975

ZFA-unknown-1077
5988 tGGTGCGGCTg 5978
5988 aCCACGCCGAc 5978

ZFA-unknown-1078
5991 cGGTGGTGCGg 5981
5991 gCCACCACGCc 5981

ZFA-unknown-1079
5994 cGCCGGTGGTg 5984
5994 gCGGCCACCAc 5984

ZFA-unknown-1080
6024 aGAGGGGGGAa 6034
6024 tCTCCCCCCTt 6034

ZFA-unknown-1081
6036 gTGGGGCGGGg 6046
6036 cACCCCGCCCc 6046

ZFA-unknown-1082
6037 tGGGGCGGGGg 6047
6037 aCCCCGCCCCc 6047

ZFA-unknown-1083
6039 gGGCGGGGGCg 6049
6039 cCCGCCCCCGc 6049

ZFA-unknown-1084
6040 gGCGGGGGCGa 6050
6040 cCGCCCCCGCt 6050

ZFA-unknown-1085
6043 gGGGGCGAGGg 6053
6043 cCCCCGCTCCc 6053

ZFA-unknown-1086
6045 gGGCGAGGGCg 6055
6045 cCCGCTCCCGc 6055

ZFA-unknown-1087
6048 cGAGGGCGGTt 6058
6048 gCTCCCGCCAa 6058

ZFA-unknown-1088
6052 gGCGGTTGAAt 6062
6052 cCGCCAACTTa 6062

ZFA-unknown-1089
6072 aTGCGTTGTGa 6062
6072 tACGCAACACt 6062

ZFA-unknown-1090
6083 aGAGGGCGTGc 6073
6083 tCTCCCGCACg 6073

ZFA-unknown-1091
6086 gGCAGAGGGCg 6076
6086 cCGTCTCCCGc 6076

ZFA-unknown-1092
6089 gGGGGCAGAGg 6079
6089 cCCCCGTCTCc 6079

ZFA-unknown-1093
6089 cGGGGACGGGt 6099
6089 gCCCCTGCCCa 6099

ZFA-unknown-1094
6097 gGTGGGAGGAa 6107
6097 cCACCCTCCTt 6107

ZFA-unknown-1095
6098 gTGGGAGGAAg 6108

6098 cACCCTCCTTc 6108

ZFA-unknown-1096
6100 gGGAGGAAGGa 6110
6100 cCCTCCTTCCt 6110

ZFA-unknown-1097
6101 gGAGGAAGGAg 6111
6101 cCTCCTTCCTc 6111

ZFA-unknown-1098
6104 gGAAGGAGGAg 6114
6104 cCTTCCTCCTc 6114

ZFA-unknown-1099
6107 aGGAGGAGGGa 6117
6107 tCCTCCTCCCt 6117

ZFA-unknown-1100
6108 gGAGGAGGGAg 6118
6108 cCTCCTCCCTc 6118

ZFA-unknown-1101
6111 gGAGGGAGAAg 6121
6111 cCTCCCTCTTc 6121

ZFA-unknown-1102
6114 gGGAGAAGAGa 6124
6114 cCCTCTTCTCt 6124

ZFA-unknown-1103
6130 cGAGGCATGCa 6140
6130 gCTCCGTACGt 6140

ZFA-unknown-1104
6143 gGGTGCATGCc 6133
6143 cCCACGTACGg 6133

ZFA-unknown-1105
6146 tGCGGGTGCAt 6136
6146 aCGCCCACGTa 6136

ZFA-unknown-1106
6147 gTGCGGGTGCa 6137
6147 cACGCCCACGt 6137

ZFA-unknown-1107
6167 gGGGGTGGGCa 6157
6167 cCCCCACCCGt 6157

ZFA-unknown-1108
6168 cGGGGGTGGGc 6158
6168 gCCCCCACCCg 6158

ZFA-unknown-1109
6170 gGCGGGGGTGg 6160
6170 cCGCCCCCACc 6160

ZFA-unknown-1110
6171 gGGCGGGGGTg 6161
6171 cCCGCCCCCAc 6161

ZFA-unknown-1111
6173 cGGGGCGGGGg 6163
6173 gCCCCGCCCCc 6163

ZFA-unknown-1112
6176 cGCCGGGGCGg 6166
6176 gCGGCCCCGCc 6166

ZFA-unknown-1113
6179 gGGCGCCGGGg 6169
6179 cCCGCGGCCCc 6169

ZFA-unknown-1114
6182 gGTGGGCGCCg 6172
6182 cCACCCGCGGc 6172

ZFA-unknown-1115
6185 cGGGGTGGGCg 6175
6185 gCCCCACCCGc 6175

ZFA-unknown-1116
6188 gGGCGGGGTGg 6178
6188 cCCGCCCCACc 6178

ZFA-unknown-1117
6191 tGCGGGCGGGg 6181
6191 aCGCCCGCCCc 6181

ZFA-unknown-1118
6194 gTGTGCGGGCg 6184
6194 cACACGCCCGc 6184

ZFA-unknown-1119
6199 gGCAGGTGTGc 6189
6199 cCGTCCACACg 6189

ZFA-unknown-1120
6206 cGTGGCGGGCa 6196
6206 gCACCGCCCGt 6196

ZFA-unknown-1121
6212 gGCGGGCGTGg 6202
6212 cCGCCCGCACc 6202

ZFA-unknown-1122
6215 aGGGGCGGGCg 6205
6215 tCCCCGCCCGc 6205

ZFA-unknown-1123
6218 aGGAGGGGCGg 6208
6218 tCCTCCCCGCc 6208

ZFA-unknown-1124
6220 tGAGGAGGGGc 6210
6220 aCTCCTCCCCg 6210

ZFA-unknown-1125
6223 gGGTGAGGAGg 6213
6223 cCCACTCCTCc 6213

ZFA-unknown-1126
6229 gGGAGAAAGGa 6239
6229 cCCTCTTTCCt 6239

ZFA-unknown-1127
6236 aGGAGGAGGAg 6246
6236 tCCTCCTCCTc 6246

ZFA-unknown-1128

6237 gGAGGAGGAGc 6247
6237 cCTCCTCCTCg 6247

ZFA-unknown-1129
6239 aGGAGGAGCAg 6249
6239 tCCTCCTCGTc 6249

ZFA-unknown-1130
6242 aGGAGCAGGAa 6252
6242 tCCTCGTCCTt 6252

ZFA-unknown-1131
6249 gGAAGAGGAGa 6259
6249 cCTTCTCCTCt 6259

ZFA-unknown-1132
6262 cGAGGCATGCa 6272
6262 gCTCCGTACGt 6272

ZFA-unknown-1133
6275 gGTTGCATGCc 6265
6275 cCAACGTACGg 6265

ZFA-unknown-1134
6278 tGCGGTTGCAt 6268
6278 aCGCCAACGTa 6268

ZFA-unknown-1135
6285 gGGTGAGTGCg 6275
6285 cCCACTCACGc 6275

ZFA-unknown-1136
6288 gTGGGGTGAGt 6278
6288 cACCCCACTCa 6278

ZFA-unknown-1137
6290 gGGTGGGGTGa 6280
6290 cCCACCCCACt 6280

ZFA-unknown-1138
6292 cGGGGTGGGGt 6282
6292 gCCCCACCCCa 6282

ZFA-unknown-1139
6295 gGGCGGGGTGg 6285
6295 cCCGCCCCACc 6285

ZFA-unknown-1140
6298 tGCGGGCGGGg 6288
6298 aCGCCCGCCCc 6288

ZFA-unknown-1141
6301 gTGTGCGGGCg 6291
6301 cACACGCCCGc 6291

ZFA-unknown-1142
6306 gGCAGGTGTGc 6296
6306 cCGTCCACACg 6296

ZFA-unknown-1143
6313 cGTGGCGGGCa 6303
6313 gCACCGCCCGt 6303

ZFA-unknown-1144
6319 gGCGGGCGTGg 6309
6319 cCGCCCGCACc 6309

ZFA-unknown-1145
6322 aGGGGCGGGCg 6312
6322 tCCCCGCCCGc 6312

ZFA-unknown-1146
6325 aGGAGGGGCGg 6315
6325 tCCTCCCCGCc 6315

ZFA-unknown-1147
6328 gTAAGGAGGGg 6318
6328 cATTCCTCCCc 6318

ZFA-unknown-1148
6332 gGCTGCGGGGa 6342
6332 cCGACGCCCCt 6342

ZFA-unknown-1149
6334 cTGCGGGGAGa 6344
6334 gACGCCCCTCt 6344

ZFA-unknown-1150
6351 aTGGGAGTCTc 6341
6351 tACCCTCAGAg 6341

ZFA-unknown-1151
6352 cGGGGCGAGGg 6362
6352 gCCCCGCTCCc 6362

ZFA-unknown-1152
6354 gGGCGAGGGGg 6364
6354 cCCGCTCCCCc 6364

ZFA-unknown-1153
6357 cGAGGGGGCTc 6367
6357 gCTCCCCCGAg 6367

ZFA-unknown-1154
6381 tGTTGCGAACg 6371
6381 aCAACGCTTGc 6371

ZFA-unknown-1155
6384 tGGTGTTGCGa 6374
6384 aCCACAACGCt 6374

ZFA-unknown-1156
6387 gTGTGGTGTTg 6377
6387 cACACCACAAc 6377

ZFA-unknown-1157
6389 tGGTGTGGTGt 6379
6389 aCCACACCACa 6379

ZFA-unknown-1158
6392 gTGTGGTGTGg 6382
6392 cACACCACACc 6382

ZFA-unknown-1159
6394 tGGTGTGGTGt 6384
6394 aCCACACCACa 6384

ZFA-unknown-1160
6397 gTGTGGTGTGg 6387
6397 cACACCACACc 6387

ZFA-unknown-1161
6404 gTGGGCCGTGt 6394
6404 cACCCGGCACa 6394

ZFA-unknown-1162
6408 tGTGGTGGGCc 6398
6408 aCACCACCCGg 6398

ZFA-unknown-1163
6409 tTGTGGTGGGc 6399
6409 aACACCACCCg 6399

ZFA-unknown-1164
6411 tGTTGTGGTGg 6401
6411 aCAACACCACc 6401

ZFA-unknown-1165
6419 gTGGGCCGTGt 6409
6419 cACCCGGCACa 6409

ZFA-unknown-1166
6423 cGTGGTGGGCc 6413
6423 gCACCACCCGg 6413

ZFA-unknown-1167
6426 tGTCGTGGTGg 6416
6426 aCAGCACCACc 6416

ZFA-unknown-1168
6429 tTGTGTCGTGg 6419
6429 aACACAGCACc 6419

ZFA-unknown-1169
6446 tTGCGGGGCAt 6456
6446 aACGCCCCGTa 6456

ZFA-unknown-1170
6449 cGGGGCATGCa 6459
6449 gCCCCGTACGt 6459

ZFA-unknown-1171
6470 gTGTGTCGACt 6460
6470 cACACAGCTGa 6460

ZFA-unknown-1172
6474 cGCGGTGTGTc 6464
6474 gCGCCACACAg 6464

ZFA-unknown-1173
6500 gGCCGCTAGGg 6490
6500 cCGGCGATCCc 6490

ZFA-unknown-1174
6503 cGGGGCCGCTa 6493
6503 gCCCCGGCGAt 6493

ZFA-unknown-1175
6506 gGCCGGGGCCg 6496
6506 cCGGCCCCGGc 6496

ZFA-unknown-1176
6509 gGGGGCCGGGg 6499
6509 cCCCCGGCCCc 6499

ZFA-unknown-1177
6513 gGCCGGGGGCc 6503

6513 cCGGCCCCCGg 6503

ZFA-unknown-1178
6524 gGAAGGAAACg 6514
6524 cCTTCCTTTGc 6514

ZFA-unknown-1179
6527 gGCGGAAGGAa 6517
6527 cCGCCTTCCTt 6517

ZFA-unknown-1180
6528 tGGCGGAAGGa 6518
6528 aCCGCCTTCCt 6518

ZFA-unknown-1181
6530 gGTGGCGGAAg 6520
6530 cCACCGCCTTc 6520

ZFA-unknown-1182
6531 tGGTGGCGGAa 6521
6531 aCCACCGCCTt 6521

ZFA-unknown-1183
6533 aGTGGTGGCGg 6523
6533 tCACCACCGCc 6523

ZFA-unknown-1184
6536 gGTAGTGGTGg 6526
6536 cCATCACCACc 6526

ZFA-unknown-1185
6539 gGTGGTAGTGg 6529
6539 cCACCATCACc 6529

ZFA-unknown-1186
6543 gGGTGGTGGTa 6533
6543 cCCACCACCAt 6533

ZFA-unknown-1187
6545 gGGGGTGGTGg 6535
6545 cCCCCACCACc 6535

ZFA-unknown-1188
6546 gGGGGGTGGTg 6536
6546 cCCCCCACCAc 6536

ZFA-unknown-1189
6548 cGGGGGGGTGg 6538
6548 gCCCCCCCACc 6538

ZFA-unknown-1190
6550 gGCGGGGGGGt 6540
6550 cCGCCCCCCCa 6540

ZFA-unknown-1191
6551 gGGCGGGGGGg 6541
6551 cCCGCCCCCCc 6541

ZFA-unknown-1192
6554 cGCGGGCGGGg 6544
6554 gCGCCCGCCCc 6544

ZFA-unknown-1193
6557 gGGCGCGGGCg 6547
6557 cCCGCGCCCGc 6547

ZFA-unknown-1194
6560 cGTGGGCGCGg 6550
6560 gCACCCGCGCc 6550

ZFA-unknown-1195
6559 cGCGGTAGAGg 6569
6559 gCGCCATCTCc 6569

ZFA-unknown-1196
6562 gGTAGAGGAAg 6572
6562 cCATCTCCTTc 6572

ZFA-unknown-1197
6565 aGAGGAAGGGg 6575
6565 tCTCCTTCCCc 6575

ZFA-unknown-1198
6568 gGAAGGGGACg 6578
6568 cCTTCCCCTGc 6578

ZFA-unknown-1199
6571 aGGGGACGGGc 6581
6571 tCCCCTGCCCg 6581

ZFA-unknown-1200
6588 gTGTGGCGCCc 6578
6588 cACACCGCGGg 6578

ZFA-unknown-1201
6590 gGGTGTGGCGc 6580
6590 cCCACACCGCg 6580

ZFA-unknown-1202
6593 cGTGGGTGTGg 6583
6593 gCACCCACACc 6583

ZFA-unknown-1203
6596 aGCCGTGGGTg 6586
6596 tCGGCACCCAc 6586

ZFA-unknown-1204
6593 gGCTGTGGCCg 6603
6593 cCGACACCGGc 6603

ZFA-unknown-1205
6596 tGTGGCCGGGc 6606
6596 aCACCGGCCCg 6606

ZFA-unknown-1206
6614 aGGCGCGTGCc 6604
6614 tCCGCGCACGg 6604

ZFA-unknown-1207
6616 tGGGGTTGTTg 6626
6616 aCCCCAACAAc 6626

ZFA-unknown-1208
6619 gGTTGTTGGGg 6629
6619 cCAACAACCCc 6629

ZFA-unknown-1209
6622 tGTTGGGGGGg 6632
6622 aCAACCCCCCc 6632

ZFA-unknown-1210

6624 tTGGGGGGGGg 6634
6624 aACCCCCCCCc 6634

ZFA-unknown-1211
6625 tGGGGGGGGGt 6635
6625 aCCCCCCCCCa 6635

ZFA-unknown-1212
6626 gGGGGGGGGTg 6636
6626 cCCCCCCCCAc 6636

ZFA-unknown-1213
6627 gGGGGGGGTGa 6637
6627 cCCCCCCCACt 6637

ZFA-unknown-1214
6629 gGGGGGTGACc 6639
6629 cCCCCCACTGg 6639

ZFA-unknown-1215
6644 cGTGGGGGCGg 6654
6644 gCACCCCCGCc 6654

ZFA-unknown-1216
6646 tGGGGGCGGTg 6656
6646 aCCCCCGCCAc 6656

ZFA-unknown-1217
6647 gGGGGCGGTGg 6657
6647 cCCCCGCCACc 6657

ZFA-unknown-1218
6649 gGGCGGTGGGc 6659
6649 cCCGCCACCCg 6659

ZFA-unknown-1219
6650 gGCGGTGGGCg 6660
6650 cCGCCACCCGc 6660

ZFA-unknown-1220
6653 gGTGGGCGTAc 6663
6653 cCACCCGCATg 6663

ZFA-unknown-1221
6680 aGGCGCGGGTc 6670
6680 tCCGCGCCCAg 6670

ZFA-unknown-1222
6683 gGCAGGCGCGg 6673
6683 cCGTCCGCGCc 6673

ZFA-unknown-1223
6686 gGGGGCAGGCg 6676
6686 cCCCCGTCCGc 6676

ZFA-unknown-1224
6689 cGGGGGGGCAg 6679
6689 gCCCCCCCGTc 6679

ZFA-unknown-1225
6694 cGACGACGGGg 6704
6694 gCTGCTGCCCc 6704

ZFA-unknown-1226
6697 cGACGGGGGGg 6707
6697 gCTGCCCCCCc 6707

ZFA-unknown-1227
6700 cGGGGGGGGGg 6710
6700 gCCCCCCCCCc 6710

ZFA-unknown-1228
6701 gGGGGGGGGGg 6711
6701 cCCCCCCCCCc 6711

ZFA-unknown-1229
6702 gGGGGGGGGGa 6712
6702 cCCCCCCCCCt 6712

ZFA-unknown-1230
6703 gGGGGGGGGAa 6713
6703 cCCCCCCCCTt 6713

ZFA-unknown-1231
6704 gGGGGGGGAAa 6714
6704 cCCCCCCCTTt 6714

ZFA-unknown-1232
6706 gGGGGGAAACg 6716
6706 cCCCCCTTTGc 6716

ZFA-unknown-1233
6707 gGGGGAAACGg 6717
6707 cCCCCTTTGCc 6717

ZFA-unknown-1234
6715 cGGGGGTGGGt 6725
6715 gCCCCCACCCa 6725

ZFA-unknown-1235
6716 gGGGGTGGGTg 6726
6716 cCCCCACCCAc 6726

ZFA-unknown-1236
6719 gGTGGGTGGAa 6729
6719 cCACCCACCTt 6729

ZFA-unknown-1237
6720 gTGGGTGGAAg 6730
6720 cACCCACCTTc 6730

ZFA-unknown-1238
6722 gGGTGGAAGGg 6732
6722 cCCACCTTCCc 6732

ZFA-unknown-1239
6723 gGTGGAAGGGa 6733
6723 cCACCTTCCCt 6733

ZFA-unknown-1240
6731 gGAAGAGGAAg 6741
6731 cCTTCTCCTTc 6741

ZFA-unknown-1241
6734 aGAGGAAGGAg 6744
6734 tCTCCTTCCTc 6744

ZFA-unknown-1242
6737 gGAAGGAGAAa 6747
6737 cCTTCCTCTTt 6747

ZFA-unknown-1243
6740 aGGAGAAAGGg 6750
6740 tCCTCTTTCCc 6750

ZFA-unknown-1244
6747 aGGGGGGGTGg 6757
6747 tCCCCCCCACc 6757

ZFA-unknown-1245
6749 gGGGGGTGGAt 6759
6749 cCCCCCACCTa 6759

ZFA-unknown-1246
6803 tTTTGTTTGTt 6793
6803 aAAACAAACAa 6793

ZFA-unknown-1247
6852 cGCGGGCGGTg 6862
6852 gCGCCCGCCAc 6862

ZFA-unknown-1248
6855 gGGCGGTGCGg 6865
6855 cCCGCCACGCc 6865

ZFA-unknown-1249
6858 cGGTGCGGCTg 6868
6858 gCCACGCCGAc 6868

ZFA-unknown-1250
6861 tGCGGCTGACc 6871
6861 aCGCCGACTGg 6871

ZFA-unknown-1251
6897 gGCGGGGGTCc 6887
6897 cCGCCCCCAGg 6887

ZFA-unknown-1252
6898 aGGCGGGGGTc 6888
6898 tCCGCCCCCAg 6888

ZFA-unknown-1253
6900 cGAGGCGGGGg 6890
6900 gCTCCGCCCCc 6890

ZFA-unknown-1254
6912 aGGGGGAGGAa 6922
6912 tCCCCCTCCTt 6922

ZFA-unknown-1255
6913 gGGGGAGGAAg 6923
6913 cCCCCTCCTTc 6923

ZFA-unknown-1256
6916 gGAGGAAGAAc 6926
6916 cCTCCTTCTTg 6926

ZFA-unknown-1257
6937 gGGGGAGAACt 6927
6937 cCCCCTCTTGa 6927

ZFA-unknown-1258
6938 tGGGGGAGAAc 6928
6938 aCCCCCTCTTg 6928

ZFA-unknown-1259
6941 gGTTGGGGGAg 6931

```
6941 cCAACCCCCTc 6931

ZFA-unknown-1260
6944 aGGGGTTGGGg 6934
6944 tCCCCAACCCc 6934

ZFA-unknown-1261
6947 aGCAGGGGTTg 6937
6947 tCGTCCCCAAc 6937

ZFA-unknown-1262
6964 gGCGGAAGAGa 6954
6964 cCGCCTTCTCt 6954

ZFA-unknown-1263
6968 gGCGGGCGGAa 6958
6968 cCGCCCGCCTt 6958

ZFA-unknown-1264
6971 gGGGGCGGGCg 6961
6971 cCCCCGCCCGc 6961

ZFA-unknown-1265
6972 gGGGGGCGGGc 6962
6972 cCCCCCGCCCg 6962

ZFA-unknown-1266
6974 gGGGGGGGCGg 6964
6974 cCCCCCCCGCc 6964

ZFA-unknown-1267
6975 gGGGGGGGGCg 6965
6975 cCCCCCCCCGc 6965

ZFA-unknown-1268
6976 gGGGGGGGGGc 6966
6976 cCCCCCCCCCg 6966

ZFA-unknown-1269
6977 gGGGGGGGGGg 6967
6977 cCCCCCCCCCc 6967

ZFA-unknown-1270
6978 aGGGGGGGGGg 6968
6978 tCCCCCCCCCc 6968

ZFA-unknown-1271
6980 aGAGGGGGGGg 6970
6980 tCTCCCCCCCc 6970

ZFA-unknown-1272
6983 gGGAGAGGGGg 6973
6983 cCCTCTCCCCc 6973

ZFA-unknown-1273
6988 cGGCGGGGAGa 6978
6988 gCCGCCCCTCt 6978

ZFA-unknown-1274
6990 gGCGGCGGGGa 6980
6990 cCGCCGCCCCt 6980

ZFA-unknown-1275
6993 tGAGGCGGCGg 6983
6993 aCTCCGCCGCc 6983
```

ZFA-unknown-1276
6996 tGCTGAGGCGg 6986
6996 aCGACTCCGCc 6986

ZFA-unknown-1277
6999 tTCTGCTGAGg 6989
6999 aAGACGACTCc 6989

ZFA-unknown-1278
6993 aGCAGAAGCTc 7003
6993 tCGTCTTCGAg 7003

ZFA-unknown-1279
7020 tTTAGGGTCGt 7010
7020 aAATCCCAGCa 7010

ZFA-unknown-1280
7025 gTAGGTTTAGg 7015
7025 cATCCAAATCc 7015

ZFA-unknown-1281
7030 cGCAGGTAGGt 7020
7030 gCGTCCATCCa 7020

ZFA-unknown-1282
7026 cTGCGAGAACg 7036
7026 gACGCTCTTGc 7036

ZFA-unknown-1284
7086 gGGCGCCTGCg 7076
7086 cCCGCGGACGc 7076

ZFA-unknown-1283
7076 cGCAGGCGCCc 7086
7076 gCGTCCGCGGg 7086

ZFA-unknown-1285
7089 gGGGGGCGCCt 7079
7089 cCCCCCGCGGa 7079

ZFA-unknown-1286
7091 gGGGGGGGCGc 7081
7091 cCCCCCCCGCg 7081

ZFA-unknown-1287
7092 gGGGGGGGGCg 7082
7092 cCCCCCCCCGc 7082

ZFA-unknown-1288
7093 gGGGGGGGGGc 7083
7093 cCCCCCCCCCg 7083

ZFA-unknown-1289
7094 gGGGGGGGGGg 7084
7094 cCCCCCCCCCc 7084

ZFA-unknown-1290
7095 gGGGGGGGGGg 7085
7095 cCCCCCCCCCc 7085

ZFA-unknown-1291
7096 cGGGGGGGGGg 7086
7096 gCCCCCCCCCc 7086

ZFA-unknown-1292

7115 cGCCGGGGGAc 7105
7115 gCGGCCCCCTg 7105

ZFA-unknown-1293
7112 gGCGGACGGCt 7122
7112 cCGCCTGCCGa 7122

ZFA-unknown-1294
7127 cGGCGCGGCGt 7137
7127 gCCGCGCCGCa 7137

ZFA-unknown-1295
7139 aGACGCCGCGc 7129
7139 tCTGCGGCGCg 7129

ZFA-unknown-1296
7138 cTCGGTGGGAc 7148
7138 gAGCCACCCTg 7148

ZFA-unknown-1297
7140 cGGTGGGACGc 7150
7140 gCCACCCTGCg 7150

ZFA-unknown-1298
7142 gTGGGACGCGg 7152
7142 cACCCTGCGCc 7152

ZFA-unknown-1299
7145 gGACGCGGGCa 7155
7145 cCTGCGCCCGt 7155

ZFA-unknown-1300
7168 cGCCGCCGCCc 7158
7168 gCGGCGGCGGg 7158

ZFA-unknown-1301
7159 gGCGGCGGCGg 7169
7159 cCGCCGCCGCc 7169

ZFA-unknown-1302
7171 cGCCGCCGCCg 7161
7171 gCGGCGGCGGc 7161

ZFA-unknown-1303
7162 gGCGGCGGCGg 7172
7162 cCGCCGCCGCc 7172

ZFA-unknown-1304
7165 gGCGGCGGGGg 7175
7165 cCGCCGCCCCc 7175

ZFA-unknown-1305
7167 cGGCGGGGGGg 7177
7167 gCCGCCCCCCc 7177

ZFA-unknown-1306
7168 gGCGGGGGGGg 7178
7168 cCGCCCCCCCc 7178

ZFA-unknown-1307
7170 cGGGGGGGGGg 7180
7170 gCCCCCCCCCc 7180

ZFA-unknown-1308
7171 gGGGGGGGGGg 7181
7171 cCCCCCCCCCc 7181

ZFA-unknown-1309
7172 gGGGGGGGGGg 7182
7172 cCCCCCCCCCc 7182

ZFA-unknown-1310
7173 gGGGGGGGGGg 7183
7173 cCCCCCCCCCc 7183

ZFA-unknown-1311
7174 gGGGGGGGGGg 7184
7174 cCCCCCCCCCc 7184

ZFA-unknown-1312
7175 gGGGGGGGGGg 7185
7175 cCCCCCCCCCc 7185

ZFA-unknown-1313
7176 gGGGGGGGGGa 7186
7176 cCCCCCCCCCt 7186

ZFA-unknown-1314
7177 gGGGGGGGGAa 7187
7177 cCCCCCCCCTt 7187

ZFA-unknown-1315
7178 gGGGGGGGAAa 7188
7178 cCCCCCCCTTt 7188

ZFA-unknown-1316
7188 aTGTGAGGAGa 7198
7188 tACACTCCTCt 7198

ZFA-unknown-1317
7218 gGAAGAGGGAa 7228
7218 cCTTCTCCCTt 7228

ZFA-unknown-1318
7221 aGAGGGAAGGg 7231
7221 tCTCCCTTCCc 7231

ZFA-unknown-1319
7225 gGAAGGGGCGc 7235
7225 cCTTCCCCGCg 7235

ZFA-unknown-1320
7230 gGGCGCGGCGg 7240
7230 cCCGCGCCGCc 7240

ZFA-unknown-1321
7233 cGCGGCGGGAc 7243
7233 gCGCCGCCCTg 7243

ZFA-unknown-1322
7235 cGGCGGGACGg 7245
7235 gCCGCCCTGCc 7245

ZFA-unknown-1323
7240 gGACGGGGGAa 7250
7240 cCTGCCCCCTt 7250

ZFA-unknown-1324
7244 gGGGGAAGACg 7254
7244 cCCCCTTCTGc 7254

ZFA-unknown-1325
7247 gGAAGACGAGg 7257
7247 cCTTCTGCTCc 7257

ZFA-unknown-1326
7250 aGACGAGGAGa 7260
7250 tCTGCTCCTCt 7260

ZFA-unknown-1327
7255 aGGAGAAGGGa 7265
7255 tCCTCTTCCCt 7265

ZFA-unknown-1328
7263 gGAAGGGGCGa 7273
7263 cCTTCCCCGCt 7273

ZFA-unknown-1329
7266 aGGGGCGAGGg 7276
7266 tCCCCGCTCCc 7276

ZFA-unknown-1330
7268 gGGCGAGGGTc 7278
7268 cCCGCTCCCAg 7278

ZFA-unknown-1331
7285 gGGAGCGGGGc 7295
7285 cCCTCGCCCCg 7295

ZFA-unknown-1332
7290 cGGGGCGGCCc 7300
7290 gCCCCGCCGGg 7300

ZFA-unknown-1333
7302 gGAGGGAGAAg 7312
7302 cCTCCCTCTTc 7312

ZFA-unknown-1334
7305 gGGAGAAGAAa 7315
7305 cCCTCTTCTTt 7315

ZFA-unknown-1335
7308 aGAAGAAACGg 7318
7308 tCTTCTTTGCc 7318

ZFA-unknown-1336
7321 cGCGGAAACGc 7331
7321 gCGCCTTTGCg 7331

ZFA-unknown-1338
7339 cGCCGGCGGCg 7329
7339 gCGGCCGCCGc 7329

ZFA-unknown-1337
7329 cGCCGCCGGCg 7339
7329 gCGGCGGCCGc 7339

ZFA-unknown-1339
7332 cGCCGGCGCGg 7342
7332 gCGGCCGCGCc 7342

ZFA-unknown-1340
7335 cGGCGCGGCCc 7345
7335 gCCGCGCCGGg 7345

ZFA-unknown-1341
7365 cGCGGGGGCCc 7355

7365 gCGCCCCCGGg 7355

ZFA-unknown-1342
7373 cGGCGGAGCGc 7363
7373 gCCGCCTCGCg 7363

ZFA-unknown-1343
7369 cGCCGGGGGCc 7379
7369 gCGGCCCCCGg 7379

ZFA-unknown-1344
7394 gGGCGGGGGAc 7404
7394 cCCGCCCCCTg 7404

ZFA-unknown-1345
7395 gGCGGGGGACg 7405
7395 cCGCCCCCTGc 7405

ZFA-unknown-1346
7398 gGGGGACGCCt 7408
7398 cCCCCTGCGGa 7408

ZFA-unknown-1347
7411 gGAAGGCGTCc 7401
7411 cCTTCCGCAGg 7401

ZFA-unknown-1348
7414 gGCGGAAGGCg 7404
7414 cCGCCTTCCGc 7404

ZFA-unknown-1349
7415 gGGCGGAAGGc 7405
7415 cCCGCCTTCCg 7405

ZFA-unknown-1350
7422 cGGCGCCGGGc 7412
7422 gCCGCGGCCCg 7412

ZFA-unknown-1351
7415 cGGCGCCGGGc 7425
7415 gCCGCGGCCCg 7425

ZFA-unknown-1352
7432 gGTAGCCGCCc 7422
7432 cCATCGGCGGg 7422

ZFA-unknown-1353
7446 gGCCGGGGGTc 7436
7446 cCGGCCCCCAg 7436

ZFA-unknown-1354
7485 tTTCGCGAACg 7495
7485 aAAGCGCTTGc 7495

ZFA-unknown-1355
7505 cGGCGCCGCGc 7495
7505 gCCGCGGCGCg 7495

ZFA-unknown-1356
7498 cGGCGCCGGAg 7508
7498 gCCGCGGCCTc 7508

ZFA-unknown-1357
7501 cGCCGGAGGGg 7511
7501 gCGGCCTCCCc 7511

ZFA-unknown-1358
7504 cGGAGGGGGCg 7514
7504 gCCTCCCCCGc 7514

ZFA-unknown-1359
7505 gGAGGGGGCGg 7515
7505 cCTCCCCCGCc 7515

ZFA-unknown-1360
7507 aGGGGGCGGCc 7517
7507 tCCCCCGCCGg 7517

ZFA-unknown-1361
7508 gGGGGCGGCCg 7518
7508 cCCCCGCCGGc 7518

ZFA-unknown-1362
7520 gGCGGCCGCCc 7510
7520 cCGCCGGCGGg 7510

ZFA-unknown-1363
7511 gGCGGCCGCCg 7521
7511 cCGCCGGCGGc 7521

ZFA-unknown-1364
7523 cTCGGCGGCCg 7513
7523 gAGCCGCCGGc 7513

ZFA-unknown-1365
7514 gGCCGCCGAGg 7524
7514 cCGGCGGCTCc 7524

ZFA-unknown-1366
7517 cGCCGAGGTGc 7527
7517 gCGGCTCCACg 7527

ZFA-unknown-1367
7522 aGGTGCGGGGg 7532
7522 tCCACGCCCCc 7532

ZFA-unknown-1368
7524 gTGCGGGGGCc 7534
7524 cACGCCCCCGg 7534

ZFA-unknown-1369
7525 tGCGGGGGCCc 7535
7525 aCGCCCCCGGg 7535

ZFA-unknown-1370
7540 cGGAGGGGCCc 7530
7540 gCCTCCCCGGg 7530

ZFA-unknown-1371
7543 gGCCGGAGGGg 7533
7543 cCGGCCTCCCc 7533

ZFA-unknown-1372
7540 gGCCGGGGCGc 7550
7540 cCGGCCCCGCg 7550

ZFA-unknown-1373
7559 cGCCGAGGTGc 7549
7559 gCGGCTCCACg 7549

ZFA-unknown-1374

7562 gGCCGCCGAGg 7552
7562 cCGGCGGCTCc 7552

ZFA-unknown-1375
7553 cTCGGCGGCCa 7563
7553 gAGCCGCCGGt 7563

ZFA-unknown-1376
7573 gGCCGGGGCTt 7563
7573 cCGGCCCCGAa 7563

ZFA-unknown-1377
7595 aGAGGCGGACc 7605
7595 tCTCCGCCTGg 7605

ZFA-unknown-1378
7606 cTCGGAGGCGc 7616
7606 gAGCCTCCGCg 7616

ZFA-unknown-1379
7608 cGGAGGCGCGg 7618
7608 gCCTCCGCGCc 7618

ZFA-unknown-1380
7611 aGGCGCGGAAg 7621
7611 tCCGCGCCTTc 7621

ZFA-unknown-1381
7614 cGCGGAAGAAg 7624
7614 gCGCCTTCTTc 7624

ZFA-unknown-1382
7617 gGAAGAAGACg 7627
7617 cCTTCTTCTGc 7627

ZFA-unknown-1383
7620 aGAAGACGGGa 7630
7620 tCTTCTGCCCt 7630

ZFA-unknown-1384
7627 gGGAGGCGGGg 7637
7627 cCCTCCGCCCc 7637

ZFA-unknown-1385
7628 gGAGGCGGGGg 7638
7628 cCTCCGCCCCc 7638

ZFA-unknown-1386
7630 aGGCGGGGGAa 7640
7630 tCCGCCCCCTt 7640

ZFA-unknown-1387
7631 gGCGGGGGAAa 7641
7631 cCGCCCCCTTt 7641

ZFA-unknown-1388
7645 gGGGGAAGAGa 7655
7645 cCCCCTTCTCt 7655

ZFA-unknown-1389
7653 aGAGGGGGAGg 7663
7653 tCTCCCCCTCc 7663

ZFA-unknown-1390
7655 aGGGGGAGGTa 7665
7655 tCCCCCTCCAt 7665

ZFA-unknown-1391
7656 gGGGGAGGTAg 7666
7656 cCCCCTCCATc 7666

ZFA-unknown-1392
7658 gGGAGGTAGGg 7668
7658 cCCTCCATCCc 7668

ZFA-unknown-1393
7659 gGAGGTAGGGa 7669
7659 cCTCCATCCCt 7669

ZFA-unknown-1394
7663 gTAGGGAGGGg 7673
7663 cATCCCTCCCc 7673

ZFA-unknown-1395
7666 gGGAGGGGAGa 7676
7666 cCCTCCCCTCt 7676

ZFA-unknown-1396
7671 gGGAGAGGAGa 7681
7671 cCCTCTCCTCt 7681

ZFA-unknown-1397
7676 aGGAGAAGGGc 7686
7676 tCCTCTTCCCg 7686

ZFA-unknown-1398
7688 cGCCGGTGCGc 7698
7688 gCGGCCACGCg 7698

ZFA-unknown-1399
7693 gTGCGCGGAGc 7703
7693 cACGCGCCTCg 7703

ZFA-unknown-1400
7698 cGGAGCAGCCt 7708
7698 gCCTCGTCGGa 7708

ZFA-unknown-1401
7715 aGAAGGAAGGc 7705
7715 tCTTCCTTCCg 7705

ZFA-unknown-1402
7718 cGGAGAAGGAa 7708
7718 gCCTCTTCCTt 7708

ZFA-unknown-1403
7731 aTCGGCGGCGg 7741
7731 tAGCCGCCGCc 7741

ZFA-unknown-1404
7734 gGCGGCGGGCc 7744
7734 cCGCCGCCCGg 7744

ZFA-unknown-1405
7750 cGCAGGGGCCc 7740
7750 gCGTCCCCGGg 7740

ZFA-unknown-1406
7762 gGCAGCAACGa 7752
7762 cCGTCGTTGCt 7752

ZFA-unknown-1407
7753 cGTTGCTGCCg 7763
7753 gCAACGACGGc 7763

ZFA-unknown-1408
7765 cGCGGCAGCAa 7755
7765 gCGCCGTCGTt 7755

ZFA-unknown-1409
7756 tGCTGCCGCGc 7766
7756 aCGACGGCGCg 7766

ZFA-unknown-1410
7768 gGGCGCGGCAg 7758
7768 cCCGCGCCGTc 7758

ZFA-unknown-1411
7771 cGGGGGCGCGg 7761
7771 gCCCCCGCGCc 7761

ZFA-unknown-1412
7787 gGATGACGCAg 7797
7787 cCTACTGCGTc 7797

ZFA-unknown-1413
7790 tGACGCAGCAg 7800
7790 aCTGCGTCGTc 7800

ZFA-unknown-1414
7802 tTCTGCTGCGt 7792
7802 aAGACGACGCa 7792

ZFA-unknown-1415
7793 cGCAGCAGAAa 7803
7793 gCGTCGTCTTt 7803

ZFA-unknown-1416
7814 tGCTGTGGGCa 7804
7814 aCGACACCCGt 7804

ZFA-unknown-1417
7817 tGTTGCTGTGg 7807
7817 aCAACGACACc 7807

ZFA-unknown-1418
7818 cGCGGGCGGGg 7828
7818 gCGCCCGCCCc 7828

ZFA-unknown-1419
7821 gGGCGGGGCTc 7831
7821 cCCGCCCCGAg 7831

ZFA-unknown-1420
7844 cGCCGGAGAGc 7834
7844 gCGGCCTCTCg 7834

ZFA-unknown-1421
7847 aGCCGCCGGAg 7837
7847 tCGGCGGCCTc 7837

ZFA-unknown-1422
7850 tTAAGCCGCCg 7840
7850 aATTCGGCGGc 7840

ZFA-unknown-1423
7872 gGGCGCCGTGc 7862

7872 cCCGCGGCACg 7862

ZFA-unknown-1424
7875 tGCGGGCGCCg 7865
7875 aCGCCCGCGGc 7865

ZFA-unknown-1425
7878 gGTTGCGGGCg 7868
7878 cCAACGCCCGc 7868

ZFA-unknown-1426
7881 tGCGGTTGCGg 7871
7881 aCGCCAACGCc 7871

ZFA-unknown-1427
7893 aGCTGAGTGCt 7883
7893 tCGACTCACGa 7883

ZFA-unknown-1428
7905 gGGGGGCGCGc 7895
7905 cCCCCCGCGCg 7895

ZFA-unknown-1429
7907 gGGGGGGGCGc 7897
7907 cCCCCCCCGCg 7897

ZFA-unknown-1430
7908 gGGGGGGGGCg 7898
7908 cCCCCCCCCGc 7898

ZFA-unknown-1431
7909 tGGGGGGGGGc 7899
7909 aCCCCCCCCCg 7899

ZFA-unknown-1432
7910 tTGGGGGGGGg 7900
7910 aACCCCCCCCc 7900

ZFA-unknown-1433
7912 cGTTGGGGGGg 7902
7912 gCAACCCCCCc 7902

ZFA-unknown-1434
7911 cGTGGGAGTGt 7921
7911 gCACCCTCACa 7921

ZFA-unknown-1435
7912 gTGGGAGTGTt 7922
7912 cACCCTCACAa 7922

ZFA-unknown-1436
7936 tGGGGCCGGCc 7946
7936 aCCCCGGCCGg 7946

ZFA-unknown-1437
7949 gGCGGCCGGCc 7939
7949 cCGCCGGCCGg 7939

ZFA-unknown-1438
7943 gGCCGCCGGAt 7953
7943 cCGGCGGCCTa 7953

ZFA-unknown-1439
7946 cGCCGGATGCc 7956
7946 gCGGCCTACGg 7956

ZFA-unknown-1440
7957 cGCGGGGGCCt 7967
7957 gCGCCCCCGGa 7967

ZFA-unknown-1441
7970 tGCGGCGGGAg 7980
7970 aCGCCGCCCTc 7980

ZFA-unknown-1442
7973 gGCGGGAGGCg 7983
7973 cCGCCCTCCGc 7983

ZFA-unknown-1443
7976 gGGAGGCGTGg 7986
7976 cCCTCCGCACc 7986

ZFA-unknown-1444
7983 gTGGGCCGCTg 7993
7983 cACCCGGCGAc 7993

ZFA-unknown-1445
7986 gGCCGCTGGCg 7996
7986 cCGGCGACCGc 7996

ZFA-unknown-1446
7992 tGGCGCCGCGg 8002
7992 aCCGCGGCGCc 8002

ZFA-unknown-1447
8004 gGCCGCGGCGc 7994
8004 cCGGCGCCGCg 7994

ZFA-unknown-1448
7995 cGCCGCGGCCc 8005
7995 gCGGCGCCGGg 8005

ZFA-unknown-1449
8012 aGCAGACGGGc 8002
8012 tCGTCTGCCCg 8002

ZFA-unknown-1450
8006 gTCTGCTGGCc 8016
8006 cAGACGACCGg 8016

ZFA-unknown-1451
8023 gGCCGCGGGCc 8013
8023 cCGGCGCCCGg 8013

ZFA-unknown-1452
8031 aGCAGACGGGc 8021
8031 tCGTCTGCCCg 8021

ZFA-unknown-1453
8025 gTCTGCTGGCc 8035
8025 cAGACGACCGg 8035

ZFA-unknown-1454
8042 gGCCGCGGGCc 8032
8042 cCGGCGCCCGg 8032

ZFA-unknown-1455
8045 cGTGGCCGCGg 8035
8045 gCACCGGCGCc 8035

ZFA-unknown-1456

```
8072 cGGAGAGAACc 8062
8072 gCCTCTCTTGg 8062

ZFA-unknown-1457
8075 cGGCGGAGAGa 8065
8075 gCCGCCTCTCt 8065

ZFA-unknown-1458
8077 cGCGGCGGAGa 8067
8077 gCGCCGCCTCt 8067

ZFA-unknown-1459
8080 gGCCGCGGCGg 8070
8080 cCGGCGCCGCc 8070

ZFA-unknown-1460
8071 cGCCGCGGCCg 8081
8071 gCGGCGCCGGc 8081

ZFA-unknown-1461
8074 cGCGGCCGGCg 8084
8074 gCGCCGGCCGc 8084

ZFA-unknown-1462
8077 gGCCGGCGCGg 8087
8077 cCGGCCGCGCc 8087

ZFA-unknown-1463
8080 cGGCGCGGGGc 8090
8080 gCCGCGCCCCg 8090

ZFA-unknown-1464
8087 gGGCGTTGCCg 8097
8087 cCCGCAACGGc 8097

ZFA-unknown-1465
8090 cGTTGCCGGCc 8100
8090 gCAACGGCCGg 8100

ZFA-unknown-1466
8113 cGGGGCCGGGc 8103
8113 gCCCCGGCCCg 8103

ZFA-unknown-1467
8125 cGCCGCGGGCt 8115
8125 gCGGCGCCCGa 8115

ZFA-unknown-1468
8121 cGGCGCTGCTc 8131
8121 gCCGCGACGAg 8131

ZFA-unknown-1469
8138 cGCAGCCGAGc 8128
8138 gCGTCGGCTCg 8128

ZFA-unknown-1470
8129 cTCGGCTGCGg 8139
8129 gAGCCGACGCc 8139

ZFA-unknown-1471
8141 gGCCGCAGCCg 8131
8141 cCGGCGTCGGc 8131

ZFA-unknown-1472
8132 gGCTGCGGCCg 8142
8132 cCGACGCCGGc 8142
```

ZFA-unknown-1473
8144 cGCGGCCGCAg 8134
8144 gCGCCGGCGTc 8134

ZFA-unknown-1474
8135 tGCGGCCGCGg 8145
8135 aCGCCGGCGCc 8145

ZFA-unknown-1475
8138 gGCCGCGGGCt 8148
8138 cCGGCGCCCGa 8148

ZFA-unknown-1476
8163 gTGCGGAGCCc 8153
8163 cACGCCTCGGg 8153

ZFA-unknown-1477
8169 gGCAGAGTGCg 8159
8169 cCGTCTCACGc 8159

ZFA-unknown-1478
8184 gGACGGGGCGa 8174
8184 cCTGCCCCGCt 8174
