# Supplementary material for: Identity of zinc finger nucleases with specificity to herpes simplex virus type II genomic DNA: novel HSV-2 vaccine/therapy precursors
Source: Theor Biol Med Model. 2011 Jun 24;8:23. doi: 10.1186/1742-4682-8-23 (PMC3138452; doi:10.1186/1742-4682-8-23)
Supplement: Additional file 3 — A list of the 684 ZFN that are specific to the HSV-2 genome. This file details the entire list of the 684 ZFN that are specific to the HSV-2 genome along with their specificity positions or sites of clevage. [file 1742-4682-8-23-S3.PDF]

Print

Zinc Finger Site Type: Nuclease
Zinc Finger Engineering Method: CoDA
Sequence Name : unknown
Sequence Length:154748
Nucleotide Sequence
:nAGTCCCCGTCCTGCCGCGCGGGGGCGGGCGCGGGAAAAAGCCGCGCGGGGGCGCCCGCGGGAAGGCAGCCCCGCGGCGCGCGGGGGGAGGGGCGGCGCCCGCGGGGGAGCGGCCGGCTCCGGGGGAGGGACGG[illegible]
Selected Module Sets:
Left Module Count: 3
Spacer Nucleotide Count: 5,6,7
Right Module Count: 3
Ignore Asp Overlap: False

The results below are zinc finger arrays that can be constructed using CoDA. Note that other methods (including modular assembly and OPEN) can also potentially be used to target the input sequence of interest.”

Sort By: Position ☐ **Hide intron splice sites**

```
ZFN-unknown-SP-7-1
1 aGTCCCCGTCCTGCCGCGCGGGGGCGg 27
1 tCAGGGGCAGGACGGCGCGCCCCCGCc 27

ZFN-unknown-SP-6-1
8 gTCCTGCCGCGCGGGGGCGGGCGCGg 33
8 cAGGACGGCGCGCCCCCGCCCGCGCc 33

ZFN-unknown-SP-7-2
64 aGGCAGCCCCGCGGCGCGCGGGGGGAg 90
64 tCCGTCGGGGCGCCGCGCGCCCCCCTc 90

ZFN-unknown-SP-5-1
69 gCCCCGCGGCGCGCGGGGGGAGGGg 93
69 cGGGGCGCCGCGCGCCCCCCTCCCc 93

ZFN-unknown-SP-6-2
69 gCCCCGCGGCGCGCGGGGGGAGGGGc 94
69 cGGGGCGCCGCGCGCCCCCCTCCCCg 94

ZFN-unknown-SP-6-3
91 gGGCGGCGCCCGCGGGGGAGCGGCCg 116
91 cCCGCCGCGGGCGCCCCCTCGCCGGc 116

ZFN-unknown-SP-6-4
94 cGGCGCCCGCGGGGGAGCGGCCGGCt 119
94 gCCGCGGGCGCCCCCTCGCCGGCCGa 119

ZFN-unknown-SP-6-5
155 gCCCTGCCGCCCGCCCGCCGCCGCCg 180
155 cGGGACGGCGGGCGGGCGGCGGCGGc 180

ZFN-unknown-SP-5-2
159 tGCCGCCCGCCCGCCGCCGCCGCCc 183
159 aCGGCGGGCGGGCGGCGGCGGCGGg 183

ZFN-unknown-SP-7-3
262 gTCCTGCCGCCGCCCCTTAAGAGGGCc 288
262 cAGGACGGCGGCGGGGAATTCTCCCGg 288

ZFN-unknown-SP-7-4
623 tCGCTCCTGCGGCGCTGGCTGCTGGTg 649
623 aGCGAGGACGCCGCGACCGACGACCAc 649

ZFN-unknown-SP-7-5
626 cTCCTGCGGCGCTGGCTGCTGGTGCCc 652
```



```
626 gAGGACGCCGCGACCGACGACCACGGg 652

ZFN-unknown-SP-6-6
676 gGACTACGCCGGCAACGACGACGCAg 701
676 cCTGATGCGGCCGTTGCTGCTGCGTc 701

ZFN-unknown-SP-6-7
679 cTACGCCGGCAACGACGACGCAGAGt 704
679 gATGCGGCCGTTGCTGCTGCGTCTCa 704

ZFN-unknown-SP-6-8
709 gAACAGCCCCCCGAGCGAGGGCGGGg 734
709 cTTGTCGGGGGGCTCGCTCCCGCCCc 734

ZFN-unknown-SP-6-9
713 aGCCCCCCGAGCGAGGGCGGGGGGAa 738
713 tCGGGGGGCTCGCTCCCGCCCCCCTt 738

ZFN-unknown-SP-5-3
713 aGCCCCCCGAGCGAGGGCGGGGGGa 737
713 tCGGGGGGCTCGCTCCCGCCCCCCt 737

ZFN-unknown-SP-7-6
753 cGCACGCCGCGCCTGCCGCCGCCTGCc 779
753 gCGTGCGGCGCGGACGGCGGCGGACGg 779

ZFN-unknown-SP-5-4
769 cGCCGCCTGCCCCCCGCCGCCGCCg 793
769 gCGGCGGACGGGGGGCGGCGGCGGc 793

ZFN-unknown-SP-5-5
772 cGCCTGCCCCCCGCCGCCGCCGCGc 796
772 gCGGACGGGGGGCGGCGGCGGCGCg 796

ZFN-unknown-SP-7-7
839 cTACGGCTGCGCACCACGACGGAGTAc 865
839 gATGCCGACGCGTGGTGCTGCCTCATg 865

ZFN-unknown-SP-7-8
897 cCCCCGCGTCCCCGCCCGCGGACGCGc 923
897 gGGGGCGCAGGGGCGGGCGCCTGCGCg 923

ZFN-unknown-SP-6-10
1069 cCGTGTCTCCCCGCCCGCAGGTGTGc 1094
1069 gGCACAGAGGGGCGGGCGTCCACACg 1094

ZFN-unknown-SP-7-9
1184 cGACCGCGACCGGTTCCGGCGCCGCGt 1210
1184 gCTGGCGCTGGCCAAGGCCGCGGCGCa 1210

ZFN-unknown-SP-5-6
1197 tTCCGGCGCCGCGTGGCGGCGGCCg 1221
1197 aAGGCCGCGGCGCACCGCCGCCGGc 1221

ZFN-unknown-SP-7-10
1300 gACCCGCGGAGGAGGAGGAGGCGGCGg 1326
1300 cTGGGCGCCTCCTCCTCCTCCGCCGCc 1326

ZFN-unknown-SP-6-11
1300 gACCCGCGGAGGAGGAGGAGGCGGCg 1325
1300 cTGGGCGCCTCCTCCTCCTCCGCCGc 1325

ZFN-unknown-SP-7-11
1506 cCGCTCCGCCCGCCCCAGGGGGCGGCg 1532
1506 gGCGAGGCGGGCGGGGTCCCCCGCCGc 1532
```

ZFN-unknown-SP-5-7
1509 cTCCGCCCGCCCCAGGGGGCGGCGc 1533
1509 gAGGCGGGCGGGGTCCCCCGCCGCg 1533

ZFN-unknown-SP-7-12
1512 cGCCCGCCCCAGGGGGCGGCGCCGGCc 1538
1512 gCGGGCGGGGTCCCCCGCCGCGGCCGg 1538

ZFN-unknown-SP-6-12
1513 gCCCGCCCCAGGGGGCGGCGCCGGCc 1538
1513 cGGGCGGGGTCCCCCGCCGCGGCCGg 1538

ZFN-unknown-SP-5-8
1931 gGGCCGCGCCGGCGGGGCGCCGGGg 1955
1931 cCCGGCGCGGCCGCCCCGCGGCCCc 1955

ZFN-unknown-SP-5-9
1934 cCGCGCCGGCGGGGCGCCGGGGGGa 1958
1934 gGCGCGGCCGCCCCGCGGCCCCCCt 1958

ZFN-unknown-SP-7-13
1960 tCCCATCTGCGTCGGCGGGGGGCGGCg 1986
1960 aGGGTAGACGCAGCCGCCCCCCGCCGc 1986

ZFN-unknown-SP-5-10
1963 cATCTGCGTCGGCGGGGGGCGGCGc 1987
1963 gTAGACGCAGCCGCCCCCCGCCGCg 1987

ZFN-unknown-SP-7-14
1966 cTGCGTCGGCGGGGGGCGGCGCATGCt 1992
1966 gACGCAGCCGCCCCCCGCCGCGTACGa 1992

ZFN-unknown-SP-6-13
2034 gGGCCGCCCCGAGAGGGGGGGATTCc 2059
2034 cCCGGCGGGGCTCTCCCCCCCTAAGg 2059

ZFN-unknown-SP-7-15
2057 tCCCTCCCTCCGCCCCCGCCGGGGCGc 2083
2057 aGGGAGGGAGGCGGGGGCGGCCCCGCg 2083

ZFN-unknown-SP-6-14
2200 gAGCGGCGGCCCGGCGGGAGGAGGAg 2225
2200 cTCGCCGCCGGGCCGCCCTCCTCCTc 2225

ZFN-unknown-SP-7-16
2200 gAGCGGCGGCCCGGCGGGAGGAGGAGa 2226
2200 cTCGCCGCCGGGCCGCCCTCCTCCTCt 2226

ZFN-unknown-SP-5-11
2335 gGACCCCGGCCCCGAGCGGCCGCCg 2359
2335 cCTGGGGCCGGGGCTCGCCGGCGGc 2359

ZFN-unknown-SP-5-12
2338 cCCCGGCCCCGAGCGGCCGCCGCGg 2362
2338 gGGGCCGGGGCTCGCCGGCGGCGCc 2362

ZFN-unknown-SP-7-17
2365 gACCCCCGGCACGGTGAGAGGGCGACc 2391
2365 cTGGGGGCCGTGCCACTCTCCCGCTGg 2391

ZFN-unknown-SP-6-15
2425 aCCCGGCTGCGGGTTGGGGGTGGTCg 2450
2425 tGGGCCGACGCCCAACCCCCACCAGc 2450

ZFN-unknown-SP-5-13

2425 aCCCGGCTGCGGGTTGGGGGTGGTc 2449
2425 tGGGCCGACGCCCAACCCCCACCAg 2449

ZFN-unknown-SP-6-16
2538 cGCCCCCCGACCCTCTGGGGGGGCGa 2563
2538 gCGGGGGGCTGGGAGACCCCCCCGCt 2563

ZFN-unknown-SP-5-14
2538 cGCCCCCCGACCCTCTGGGGGGGCg 2562
2538 gCGGGGGGCTGGGAGACCCCCCCGc 2562

ZFN-unknown-SP-5-15
2539 gCCCCCCGACCCTCTGGGGGGGCGa 2563
2539 cGGGGGGCTGGGAGACCCCCCCGCt 2563

ZFN-unknown-SP-7-18
2664 cCCCTTCCGAACAAAAAGCAGCGGGCg 2690
2664 gGGGAAGGCTTGTTTTTCGTCGCCCGc 2690

ZFN-unknown-SP-5-16
2792 cCGCCCCGCACGCCTGGGGGATGCt 2816
2792 gGCGGGGCGTGCGGACCCCCTACGa 2816

ZFN-unknown-SP-6-17
2792 cCGCCCCGCACGCCTGGGGGATGCTc 2817
2792 gGCGGGGCGTGCGGACCCCCTACGAg 2817

ZFN-unknown-SP-6-18
2835 cGCCAGCAGCGACTCGGAGGAGGAGa 2860
2835 gCGGTCGTCGCTGAGCCTCCTCCTCt 2860

ZFN-unknown-SP-5-17
2835 cGCCAGCAGCGACTCGGAGGAGGAg 2859
2835 gCGGTCGTCGCTGAGCCTCCTCCTc 2859

ZFN-unknown-SP-5-18
2838 cAGCAGCGACTCGGAGGAGGAGACc 2862
2838 gTCGTCGCTGAGCCTCCTCCTCTGg 2862

ZFN-unknown-SP-6-19
2892 cCGCGACTCCACCTCCGAGGCGGGCa 2917
2892 gGCGCTGAGGTGGAGGCTCCGCCCGt 2917

ZFN-unknown-SP-6-20
2994 gGGCAGCCCCACGCCCGCCGACGCGc 3019
2994 cCCGTCGGGGTGCGGGCGGCTGCGCg 3019

ZFN-unknown-SP-7-19
3011 cCGACGCGCAGGGATCCTGTGGGGGTg 3037
3011 gGCTGCGCGTCCCTAGGACACCCCCAc 3037

ZFN-unknown-SP-6-21
3674 gCGCCCCGGCGCGGGGGCGGCGGTGc 3699
3674 cGCGGGGCCGCGCCCCCGCCGCCACg 3699

ZFN-unknown-SP-6-22
3755 gCCCCGCGGAGCAGCAGCAGCGGCGg 3780
3755 cGGGGCGCCTCGTCGTCGTCGCCGCc 3780

ZFN-unknown-SP-5-19
3772 cAGCGGCGGCGCCCCGTTGCGGGCg 3796
3772 gTCGCCGCCGCGGGGCAACGCCCGc 3796

ZFN-unknown-SP-5-20
3775 cGGCGGCGCCCCGTTGCGGGCGGGg 3799
3775 gCCGCCGCGGGGCAACGCCCGCCCc 3799

ZFN-unknown-SP-5-21
3857 tCTCTTCCCCCTGCGGCCGGCGGGg 3881
3857 aGAGAAGGGGGACGCCGGCCGCCCc 3881

ZFN-unknown-SP-5-22
3861 tTCCCCCTGCGGCCGGCGGGGGGCg 3885
3861 aAGGGGGACGCCGGCCGCCCCCCGc 3885

ZFN-unknown-SP-5-23
3864 cCCCTGCGGCCGGCGGGGGGCGCGc 3888
3864 gGGGACGCCGGCCGCCCCCCGCGCg 3888

ZFN-unknown-SP-5-24
4115 gCGCGCCCCCGCGCGGCCGTCGCCc 4139
4115 cGCGCGGGGGCGCGCCGGCAGCGGg 4139

ZFN-unknown-SP-5-25
4151 aGTCCGCCCCGCGCCGCCGCCGCCc 4175
4151 tCAGGCGGGGCGCGGCGGCGGCGGg 4175

ZFN-unknown-SP-6-23
4164 cCGCCGCCGCCCCCGTGGTGTCTGCg 4189
4164 gGCGGCGGCGGGGGCACCACAGACGc 4189

ZFN-unknown-SP-7-20
4212 cGCCGCCCGCCGTGCCGGTGGACGCGc 4238
4212 gCGGCGGGCGGCACGGCCACCTGCGCg 4238

ZFN-unknown-SP-6-24
4369 cGGCACCAACACCCCCGGTGCCGCCc 4394
4369 gCCGTGGTTGTGGGGGCCACGGCGGg 4394

ZFN-unknown-SP-7-21
4386 gTGCCGCCCCCCACGCCGCGGAGGGGg 4412
4386 cACGGCGGGGGGTGCGGCGCCTCCCCc 4412

ZFN-unknown-SP-6-25
4387 tGCCGCCCCCCACGCCGCGGAGGGGg 4412
4387 aCGGCGGGGGGTGCGGCGCCTCCCCc 4412

ZFN-unknown-SP-7-22
4389 cCGCCCCCCACGCCGCGGAGGGGGCGg 4415
4389 gGCGGGGGGTGCGGCGCCTCCCCCGCc 4415

ZFN-unknown-SP-6-26
4389 cCGCCCCCCACGCCGCGGAGGGGGCg 4414
4389 gGCGGGGGGTGCGGCGCCTCCCCCGc 4414

ZFN-unknown-SP-6-27
4390 cGCCCCCCACGCCGCGGAGGGGGCGg 4415
4390 gCGGGGGGTGCGGCGCCTCCCCCGCc 4415

ZFN-unknown-SP-5-26
4390 cGCCCCCCACGCCGCGGAGGGGGCg 4414
4390 gCGGGGGGTGCGGCGCCTCCCCCGc 4414

ZFN-unknown-SP-7-23
4417 gGCCCGCCCCCGGAAGAGGCGCGGGTc 4443
4417 cCGGGCGGGGGCCTTCTCCGCGCCCAg 4443

ZFN-unknown-SP-6-28
4418 gCCCGCCCCCGGAAGAGGCGCGGGTc 4443
4418 cGGGCGGGGGCCTTCTCCGCGCCCAg 4443

ZFN-unknown-SP-5-27
4498 gCCCCTCGCCCCCCAGGGGGTGGGg 4522
4498 cGGGGAGCGGGGGGTCCCCCACCCc 4522

ZFN-unknown-SP-6-29
4498 gCCCCTCGCCCCCCAGGGGGTGGGGg 4523
4498 cGGGGAGCGGGGGGTCCCCCACCCCc 4523

ZFN-unknown-SP-7-24
4500 cCCTCGCCCCCCAGGGGGTGGGGGCCa 4526
4500 gGGAGCGGGGGGTCCCCCACCCCCGGt 4526

ZFN-unknown-SP-6-30
4500 cCCTCGCCCCCCAGGGGGTGGGGGCc 4525
4500 gGGAGCGGGGGGTCCCCCACCCCCGg 4525

ZFN-unknown-SP-7-25
4587 tCGCCCCGGCGTCCGCGGGCGCCGCGc 4613
4587 aGCGGGGCCGCAGGCGCCCGCGGCGg 4613

ZFN-unknown-SP-7-26
4623 cGTCTCCGTCGTCCCAGGCCGCGGTCg 4649
4623 gCAGAGGCAGCAGGGTCCGGCGCCAGc 4649

ZFN-unknown-SP-7-27
4626 cTCCGTCGTCCCAGGCCGCGGTCGCCg 4652
4626 gAGGCAGCAGGGTCCGGCGCCAGCGGc 4652

ZFN-unknown-SP-7-28
4629 cGTCGTCCCAGGCCGCGGTCGCCGCCg 4655
4629 gCAGCAGGGTCCGGCGCCAGCGGCGGc 4655

ZFN-unknown-SP-7-29
4755 cCTCCGCCTCTTCCTCTGCGGGCGGGg 4781
4755 gGAGGCGGAGAAGGAGACGCCCGCCCc 4781

ZFN-unknown-SP-7-30
4758 cCGCCTCTTCCTCTGCGGGCGGGGCTg 4784
4758 gGCGGAGAAGGAGACGCCCGCCCCGAc 4784

ZFN-unknown-SP-7-31
4761 cCTCTTCCTCTGCGGGCGGGGCTGGTg 4787
4761 gGAGAAGGAGACGCCCGCCCCGACCAc 4787

ZFN-unknown-SP-7-32
4764 cTTCCTCTGCGGGCGGGGCTGGTGGGa 4790
4764 gAAGGAGACGCCCGCCCCGACCACCCt 4790

ZFN-unknown-SP-7-33
4791 gCGTCGCGTCCGCGTCCGGCGCTGGGg 4817
4791 cGCAGCGCAGGCGCAGGCCGCGACCCc 4817

ZFN-unknown-SP-7-34
4794 tCGCGTCCGCGTCCGGCGCTGGGGAGa 4820
4794 aGCGCAGGCGCAGGCCGCGACCCCTCt 4820

ZFN-unknown-SP-6-31
4797 cGTCCGCGTCCGGCGCTGGGGAGAGa 4822
4797 gCAGGCGCAGGCCGCGACCCCTCTCt 4822

ZFN-unknown-SP-6-32
4831 cTCCCTCGGCCCCCGCGCTGCTGCGc 4856
4831 gAGGGAGCCGGGGGCGCGACGACGCg 4856

ZFN-unknown-SP-5-28
4837 cGGCCCCCGCGCTGCTGCGCCGCGg 4861

4837 gCCGGGGGCGCGACGACGCGGCGCc 4861

ZFN-unknown-SP-6-33
4845 gCGCTGCTGCGCCGCGGGGGCCGAGg 4870
4845 cGCGACGACGCGGCGCCCCCGGCTCc 4870

ZFN-unknown-SP-6-34
4848 cTGCTGCGCCGCGGGGGCCGAGGAAg 4873
4848 gACGACGCGGCGCCCCCGGCTCCTTc 4873

ZFN-unknown-SP-5-29
5112 cCGCGGCCCCCGCGTGGAGCCGCCg 5136
5112 gGCGCCGGGGGCGCACCTCGGCGGc 5136

ZFN-unknown-SP-5-30
5115 cGGCCCCCGCGTGGAGCCGCCGCAc 5139
5115 gCCGGGGGCGCACCTCGGCGGCGTg 5139

ZFN-unknown-SP-7-35
5186 cCGACGCCCCCCGCGTCGGAGTGGAAc 5212
5186 gGCTGCGGGGGGCGCAGCCTCACCTTg 5212

ZFN-unknown-SP-7-36
5187 cGACGCCCCCCGCGTCGGAGTGGAACa 5213
5187 gCTGCGGGGGGCGCAGCCTCACCTTGt 5213

ZFN-unknown-SP-6-35
5187 cGACGCCCCCCGCGTCGGAGTGGAAc 5212
5187 gCTGCGGGGGGCGCAGCCTCACCTTg 5212

ZFN-unknown-SP-7-37
5294 tCGCGCCACCCGTGGTCTCGGGAGCAg 5320
5294 aGCGCGGTGGGCACCAGAGCCCTCGTc 5320

ZFN-unknown-SP-6-36
5420 cACCGGCCGAGGCGCCTCGGGGGCAg 5445
5420 gTGGCCGGCTCCGCGGAGCCCCCGTc 5445

ZFN-unknown-SP-7-38
5667 cAGCTCCCCCGCGCAGGGGGGCCGGGg 5693
5667 gTCGAGGGGGCGCGTCCCCCCGGCCCc 5693

ZFN-unknown-SP-5-31
5669 gCTCCCCCGCGCAGGGGGGCCGGGg 5693
5669 cGAGGGGGCGCGTCCCCCCGGCCCc 5693

ZFN-unknown-SP-7-39
5735 aAGCCGCCCCCACCCCCGAGGCCTGTt 5761
5735 tTCGGCGGGGGTGGGGGCTCCGGACAa 5761

ZFN-unknown-SP-6-37
5736 aGCCGCCCCCACCCCCGAGGCCTGTt 5761
5736 tCGGCGGGGGTGGGGGCTCCGGACAa 5761

ZFN-unknown-SP-7-40
6073 gCACGCCCTCTGCCCCCGGGGACGGGt 6099
6073 cGTGCGGGAGACGGGGGCCCCTGCCCa 6099

ZFN-unknown-SP-7-41
6213 cCTCCTCACCCTGGCTGGGAGAAAGGa 6239
6213 gGAGGAGTGGGACCGACCCTCTTTCCt 6239

ZFN-unknown-SP-5-32
6318 cCCCTCCTTACCCTGGCTGCGGGGa 6342
6318 gGGGAGGAATGGGACCGACGCCCCt 6342

ZFN-unknown-SP-7-42
6318 cCCCTCCTTACCCTGGCTGCGGGGAGa 6344
6318 gGGGAGGAATGGGACCGACGCCCCTCt 6344

ZFN-unknown-SP-7-43
6341 gAGACTCCCATCGGGGCGAGGGGGCTc 6367
6341 cTCTGAGGGTAGCCCCGCTCCCCCGAg 6367

ZFN-unknown-SP-6-38
6544 cCCCGCCCGCGCCCACGCGGTAGAGg 6569
6544 gGGGCGGGCGCGGGTGCGCCATCTCc 6569

ZFN-unknown-SP-6-39
6547 cGCCCGCGCCCACGCGGTAGAGGAAg 6572
6547 gCGGGCGCGGGTGCGCCATCTCCTTc 6572

ZFN-unknown-SP-6-40
6550 cCGCGCCCACGCGGTAGAGGAAGGGg 6575
6550 gGCGCGGGTGCGCCATCTCCTTCCCc 6575

ZFN-unknown-SP-6-41
6578 gGGCGCCACACCCACGGCTGTGGCCg 6603
6578 cCCGCGGTGTGGGTGCCGACACCGGc 6603

ZFN-unknown-SP-7-44
6580 gCGCCACACCCACGGCTGTGGCCGGGc 6606
6580 cGCGGTGTGGGTGCCGACACCGGCCCg 6606

ZFN-unknown-SP-6-42
6604 gGCACGCGCCTTTGGGGTTGTTGGGg 6629
6604 cCGTGCGCGGAAACCCCAACAACCCc 6629

ZFN-unknown-SP-6-43
6679 cTGCCCCCCCGGGAACGACGACGGGg 6704
6679 gACGGGGGGGCCCTTGCTGCTGCCCc 6704

ZFN-unknown-SP-6-44
6978 tCTCCCCGCCGCCTCAGCAGAAGCTc 7003
6978 aGAGGGGCGGCGGAGTCGTCTTCGAg 7003

ZFN-unknown-SP-7-45
7010 aCGACCCTAAACCTACCTGCGAGAACg 7036
7010 tGCTGGGATTTGGATGGACGCTCTTGc 7036

ZFN-unknown-SP-7-46
7129 gCGCGGCGTCTCGGTGGGACGCGGGCa 7155
7129 cGCGCCGCAGAGCCACCCTGCGCCCGt 7155

ZFN-unknown-SP-6-45
7158 gGGCGGCGGCGGCGGGGGGGGGGGg 7183
7158 cCCGCCGCCGCCGCCCCCCCCCCCc 7183

ZFN-unknown-SP-7-47
7158 gGGCGGCGGCGGCGGGGGGGGGGGGg 7184
7158 cCCGCCGCCGCCGCCCCCCCCCCCCc 7184

ZFN-unknown-SP-5-33
7158 gGGCGGCGGCGGCGGGGGGGGGGg 7182
7158 cCCGCCGCCGCCGCCCCCCCCCCc 7182

ZFN-unknown-SP-5-34
7161 cGGCGGCGGCGGGGGGGGGGGGGg 7185
7161 gCCGCCGCCGCCCCCCCCCCCCCc 7185

ZFN-unknown-SP-7-48

```
7161 cGGCGGCGGCGGGGGGGGGGGGGGGAa 7187
7161 gCCGCCGCCGCCCCCCCCCCCCCCCTt 7187

⊞ ZFN-unknown-SP-6-46
7161 cGGCGGCGGCGGGGGGGGGGGGGGGa 7186
7161 gCCGCCGCCGCCCCCCCCCCCCCCCt 7186

⊞ ZFN-unknown-SP-5-35
7355 gGGCCCCCGCGCTCCGCCGGGGGCc 7379
7355 cCCGGGGGCGCGAGGCGGCCCCCGg 7379

⊞ ZFN-unknown-SP-5-36
7401 gGACGCCTTCCGCCCGGCGCCGGGc 7425
7401 cCTGCGGAAGGCGGGCCGCGGCCCg 7425

⊞ ZFN-unknown-SP-7-49
7495 gCGCGGCGCCGGAGGGGGCGGCCGCCg 7521
7495 cGCGCCGCGGCCTCCCCCGCCGGCGGc 7521

⊞ ZFN-unknown-SP-6-47
7510 gGGCGGCCGCCGAGGTGCGGGGGCCc 7535
7510 cCCGCCGGCGGCTCCACGCCCCCGGg 7535

⊞ ZFN-unknown-SP-5-37
7510 gGGCGGCCGCCGAGGTGCGGGGGCc 7534
7510 cCCGCCGGCGGCTCCACGCCCCCGg 7534

⊞ ZFN-unknown-SP-7-50
7740 gGGCCCCTGCGTTCGTTGCTGCCGCGc 7766
7740 cCCGGGGACGCAAGCAACGACGGCGCg 7766

⊞ ZFN-unknown-SP-5-38
7804 tGCCCACAGCAACACGCGGGCGGGg 7828
7804 aCGGGTGTCGTTGTGCGCCCGCCCc 7828

⊞ ZFN-unknown-SP-5-39
7807 cCACAGCAACACGCGGGCGGGGCTc 7831
7807 gGTGTCGTTGTGCGCCCGCCCCGAg 7831

⊞ ZFN-unknown-SP-7-51
7895 gCGCGCCCCCCCCCAACGTGGGAGTGt 7921
7895 cGCGCGGGGGGGGGTTGCACCCTCACa 7921

⊞ ZFN-unknown-SP-6-48
7897 gCGCCCCCCCCCAACGTGGGAGTGTt 7922
7897 cGCGGGGGGGGGTTGCACCCTCACAa 7922

⊞ ZFN-unknown-SP-5-40
7897 gCGCCCCCCCCCAACGTGGGAGTGt 7921
7897 cGCGGGGGGGGGTTGCACCCTCACa 7921

⊞ ZFN-unknown-SP-5-41
7898 cGCCCCCCCCCAACGTGGGAGTGTt 7922
7898 gCGGGGGGGGGTTGCACCCTCACAa 7922

⊞ ZFN-unknown-SP-6-49
8062 gGTTCTCTCCGCCGCGGCCGGCGCGg 8087
8062 cCAAGAGAGGCGGCGCCGGCCGCGCc 8087

⊞ ZFN-unknown-SP-6-50
8065 tCTCTCCGCCGCGGCCGGCGCGGGGc 8090
8065 aGAGAGGCGGCGCCGGCCGCGCCCCg 8090

⊞ ZFN-unknown-SP-5-42
8115 aGCCCGCGGCGCTGCTCGGCTGCGg 8139
8115 tCGGGCGCCGCGACGAGCCGACGCc 8139
```

ZFN-unknown-SP-7-52
8330 gGTCTCCTTCGTCGGGCGGCGGGGGGg 8356
8330 cCAGAGGAAGCAGCCCGCCGCCCCCCc 8356

ZFN-unknown-SP-5-43
8330 gGTCTCCTTCGTCGGGCGGCGGGGg 8354
8330 cCAGAGGAAGCAGCCCGCCGCCCCc 8354

ZFN-unknown-SP-7-53
8333 cTCCTTCGTCGGGCGGCGGGGGGGGg 8359
8333 gAGGAAGCAGCCCGCCGCCCCCCCCc 8359

ZFN-unknown-SP-5-44
8333 cTCCTTCGTCGGGCGGCGGGGGGGg 8357
8333 gAGGAAGCAGCCCGCCGCCCCCCCc 8357

ZFN-unknown-SP-6-51
8691 gCGCGGCGGCGGTCGGGGTGGGGGGa 8716
8691 cGCGCCGCCGCCAGCCCCACCCCCCt 8716

ZFN-unknown-SP-7-54
8691 gCGCGGCGGCGGTCGGGGTGGGGGGAg 8717
8691 cGCGCCGCCGCCAGCCCCACCCCCCTc 8717

ZFN-unknown-SP-7-55
8751 cCACTCCCACCCCGACCGCCGCCGCGc 8777
8751 gGTGAGGGTGGGGCTGGCGGCGGCGCg 8777

ZFN-unknown-SP-7-56
8787 tGCCGCCCGCGCGCTCCTGTGTGGACc 8813
8787 aCGGCGGGCGCGCGAGGACACACCTGg 8813

ZFN-unknown-SP-7-57
8948 gCCCCCCTGCCGGGCGGGGCGGTGGGg 8974
8948 cGGGGGGACGGCCCGCCCCGCCACCCc 8974

ZFN-unknown-SP-5-45
8948 gCCCCCCTGCCGGGCGGGGCGGTGg 8972
8948 cGGGGGGACGGCCCGCCCCGCCACc 8972

ZFN-unknown-SP-5-46
9029 gGGCGCCGGCGCGACGCGGGCGGCc 9053
9029 cCCGCGGCCGCGCTGCGCCCGCCGg 9053

ZFN-unknown-SP-5-47
9064 gCGCGGCGGCCGGGCGGGGGCGCGc 9088
9064 cGCGCCGCCGGCCCGCCCCCGCGCg 9088

ZFN-unknown-SP-5-48
9085 gCGCGGCGGCCGGGCGGGGGCGCGc 9109
9085 cGCGCCGCCGGCCCGCCCCCGCGCg 9109

ZFN-unknown-SP-5-49
9106 gCGCGGCGGCCGGGCGGGGGCGCGc 9130
9106 cGCGCCGCCGGCCCGCCCCCGCGCg 9130

ZFN-unknown-SP-5-50
9127 gCGCGGCGGCCGGGCGGGGGCGCGc 9151
9127 cGCGCCGCCGGCCCGCCCCCGCGCg 9151

ZFN-unknown-SP-5-51
9148 gCGCGGCGGCCGGGCGGGGGCGCGc 9172
9148 cGCGCCGCCGGCCCGCCCCCGCGCg 9172

ZFN-unknown-SP-5-52
9169 gCGCGGCGGCCGGGCGGGGGCGCGc 9193
9169 cGCGCCGCCGGCCCGCCCCCGCGCg 9193

ZFN-unknown-SP-6-52
9892 cGCACTCGCCGCTGCGTCGGGCGACg 9917
9892 gCGTGAGCGGCGACGCAGCCCGCTGc 9917

ZFN-unknown-SP-5-53
10052 tCTCCCCGACGCGAGGTCGGCGCCg 10076
10052 aGAGGGGCTGCGCTCCAGCCGCGGc 10076

ZFN-unknown-SP-5-54
10145 cCGACCCGTCTAGCGGCCGGGTCGg 10169
10145 gGCTGGGCAGATCGCCGGCCCAGCc 10169

ZFN-unknown-SP-6-53
10175 cAGCGTCGTCGCCCTAGAGGCTGTCc 10200
10175 gTCGCAGCAGCGGGATCTCCGACAGg 10200

ZFN-unknown-SP-7-58
10337 tGGCGGCCCCTGTTGGAGCCGGAGCTc 10363
10337 aCCGCCGGGGACAACCTCGGCCTCGAg 10363

ZFN-unknown-SP-5-55
10424 gTGCTGCCGCCGCGGGAGGATGTGt 10448
10424 cACGACGGCGGCGCCCTCCTACACa 10448

ZFN-unknown-SP-5-56
10660 tCGCGACGGCGTGCTGTTGTTGAAc 10684
10660 aGCGCTGCCGCACGACAACAACTTg 10684

ZFN-unknown-SP-5-57
11275 gGCCGGCGACCCGCCGGGGGCGGAc 11299
11275 cCGGCCGCTGGGCGGCCCCCGCCTg 11299

ZFN-unknown-SP-6-54
11275 gGCCGGCGACCCGCCGGGGGCGGACg 11300
11275 cCGGCCGCTGGGCGGCCCCCGCCTGc 11300

ZFN-unknown-SP-6-55
11430 aGACCTCGTCGTTTTCGGGGACGGCc 11455
11430 tCTGGAGCAGCAAAAGCCCCTGCCGg 11455

ZFN-unknown-SP-6-56
11949 gGTCTGCGTCGTCCAGGGTGGGGCTg 11974
11949 cCAGACGCAGCAGGTCCCACCCCGAc 11974

ZFN-unknown-SP-6-57
12094 gCACCCCCGCGCCGCCTCGGGGGCCg 12119
12094 cGTGGGGGCGCGGCGGAGCCCCCGGc 12119

ZFN-unknown-SP-6-58
12097 cCCCCGCGCCGCCTCGGGGGCCGTCc 12122
12097 gGGGGCGCGGCGGAGCCCCCGGCAGg 12122

ZFN-unknown-SP-5-58
12160 cCACACCACCGTCAGGATGCCGGCc 12184
12160 gGTGTGGTGGCAGTCCTACGGCCGg 12184

ZFN-unknown-SP-5-59
12337 cACCAGCAGCCCGATGGGGGTGTGt 12361
12337 gTGGTCGTCGGGCTACCCCCACACa 12361

ZFN-unknown-SP-6-59
12337 cACCAGCAGCCCGATGGGGGTGTGTt 12362

12337 gTGGTCGTCGGGCTACCCCCACACAa 12362

ZFN-unknown-SP-5-60
12369 cCACGCCTCCGCGGGGCAGGCGGTc 12393
12369 gGTGCGGAGGCGCCCCGTCCGCCAg 12393

ZFN-unknown-SP-7-59
12990 aCTCGGCCACGCCGGCCGTCGCGTGCc 13016
12990 tGAGCCGGTGCGGCCGGCAGCGCACGg 13016

ZFN-unknown-SP-6-60
13015 cCGCCGCCGCAGCTCCTCCGCGAACg 13040
13015 gGCGGCGGCGTCGAGGAGGCGCTTGc 13040

ZFN-unknown-SP-5-61
13060 gTACCCCATCAGCGTGTAGTTGTCc 13084
13060 cATGGGGTAGTCGCACATCAACAGg 13084

ZFN-unknown-SP-5-62
13423 aCGCTCCCCCGGAGAGCGGTCGCTg 13447
13423 tGCGAGGGGGCCTCTCGCCAGCGAc 13447

ZFN-unknown-SP-7-60
13449 tGTCGGCGGCCCTGGTTGCCGATGCGc 13475
13449 aCAGCCGCCGGGACCAACGGCTACGCg 13475

ZFN-unknown-SP-5-63
13473 cGCCGGCGGCGTCCCGGCGTAGCGa 13497
13473 gCGGCCGCCGCAGGGCCGCATCGCt 13497

ZFN-unknown-SP-5-64
13507 tGCCGTCAGCTCCCCTAGGCGGCCg 13531
13507 aCGGCAGTCGAGGGGATCCGCCGGc 13531

ZFN-unknown-SP-6-61
13746 tCACCGCGACCTGGCTGTTGAGGACg 13771
13746 aGTGGCGCTGGACCGACAACTCCTGc 13771

ZFN-unknown-SP-7-61
14389 gCACACCAGCACCGGCCGCAGGCGGCc 14415
14389 cGTGTGGTCGTGGCCGGCGTCCGCCGg 14415

ZFN-unknown-SP-7-62
14637 gCGCGTCTTCGCCCCCGTGCGCCGCCa 14663
14637 cGCGCAGAAGCGGGGGCACGCGGCGGt 14663

ZFN-unknown-SP-5-65
15052 gCGCGGCACCTGCGTGACGTCGAGc 15076
15052 cGCGCCGTGGACGCACTGCAGCTCg 15076

ZFN-unknown-SP-6-62
15249 tGGCCGCACAGCGCGCGCGGGCGCCg 15274
15249 aCCGGCGTGTCGCGCGCGCCCGCGGc 15274

ZFN-unknown-SP-6-63
15267 gGGCGCCGGCGATGCGGACGCGGGGc 15292
15267 cCCGCGGCCGCTACGCCTGCGCCCCg 15292

ZFN-unknown-SP-6-64
15307 aTGCGCCCCCGAGGACGGCTGGGTGa 15332
15307 tACGCGGGGGCTCCTGCCGACCCACt 15332

ZFN-unknown-SP-7-63
15335 gTTCACCCCACCCCCGGGACGATGTTg 15361
15335 cAAGTGGGGTGGGGGCCCTGCTACAAc 15361

ZFN-unknown-SP-5-66
15337 tCACCCCACCCCCGGGACGATGTTg 15361
15337 aGTGGGGTGGGGGCCCTGCTACAAc 15361

ZFN-unknown-SP-6-65
15339 aCCCCACCCCCGGGACGATGTTGTTc 15364
15339 tGGGGTGGGGGCCCTGCTACAACAAg 15364

ZFN-unknown-SP-5-67
15340 cCCCACCCCCGGGACGATGTTGTTc 15364
15340 gGGGTGGGGGCCCTGCTACAACAAg 15364

ZFN-unknown-SP-6-66
15435 aGGCCGCCACCCGACAGCTGCAGGCg 15460
15435 tCCGGCGGTGGGCTGTCGACGTCCGc 15460

ZFN-unknown-SP-5-68
15436 gGCCGCCACCCGACAGCTGCAGGCg 15460
15436 cCGGCGGTGGGCTGTCGACGTCCGc 15460

ZFN-unknown-SP-5-69
15439 cGCCACCCGACAGCTGCAGGCGGCg 15463
15439 gCGGTGGGCTGTCGACGTCCGCCGc 15463

ZFN-unknown-SP-5-70
15532 gGCCCGCGGCCTCCAGCCGCAGCGg 15556
15532 cCGGGCGCCGGAGGTCGGCGTCGCc 15556

ZFN-unknown-SP-5-71
15535 cCGCGGCCTCCAGCCGCAGCGGCTg 15559
15535 gGCGCCGGAGGTCGGCGTCGCCGAc 15559

ZFN-unknown-SP-6-67
15786 aGGCGACGCAGCGCCTGGGGGCGTTt 15811
15786 tCCGCTGCGTCGCGGACCCCCGCAAa 15811

ZFN-unknown-SP-7-64
15940 cATCTACTACAACCATCGCCGGGGGGa 15966
15940 gTAGATGATGTTGGTAGCGGCCCCCCt 15966

ZFN-unknown-SP-7-65
15950 aACCATCGCCGGGGGGAGTGGCTGGTg 15976
15950 tTGGTAGCGGCCCCCCTCACCGACCAc 15976

ZFN-unknown-SP-5-72
16110 aACACGCGCACATCTGCCGTCTGCg 16134
16110 tTGTGCGCGTGTAGACGGCAGACGc 16134

ZFN-unknown-SP-6-68
16137 aCACCGCGTCCGTCAAGGTGCTGTTg 16162
16137 tGTGGCGCAGGCAGTTCCACGACAAc 16162

ZFN-unknown-SP-6-69
16171 gAGCGACAGCGAGCGCGGGGTGGCTg 16196
16171 cTCGCTGTCGCTCGCGCCCCACCGAc 16196

ZFN-unknown-SP-6-70
16330 aCGCGCCTACTTGGACGAGGCGGGGg 16355
16330 tGCGCGGATGAACCTGCTCCGCCCCc 16355

ZFN-unknown-SP-7-66
16384 cCACACCCTCCCTGGGTTCGGCAAGGg 16410
16384 gGTGTGGGAGGGACCCAAGCCGTTCCc 16410

ZFN-unknown-SP-6-71

```
16385 cACACCCTCCCTGGGTTCGGCAAGGg 16410
16385 gTGTGGGAGGGACCCAAGCCGTTCCc 16410

ZFN-unknown-SP-7-67
16427 tCGCGCCCCCAGGACCCGGGGGCGCGa 16453
16427 aGCGCGGGGGTCCTGGGCCCCCGCGCt 16453

ZFN-unknown-SP-5-73
16495 cAACATCAACGGCATGCTGGAGGGc 16519
16495 gTTGTAGTTGCCGTACGACCTCCCg 16519

ZFN-unknown-SP-7-68
16604 gAGCTGCGGCGCGCCCAGGCGGGGGCg 16630
16604 cTCGACGCCGCGCGGGTCCGCCCCCGc 16630

ZFN-unknown-SP-5-74
16996 tGTCATCCTAGGGGAGGAGGAGTTa 17020
16996 aCAGTAGGATCCCCTCCTCCTCAAt 17020

ZFN-unknown-SP-7-69
17067 aCGTCGCGGCCCTGTTCGTGGCGGACg 17093
17067 tGCAGCGCCGGGACAAGCACCGCCTGc 17093

ZFN-unknown-SP-6-72
17261 gGCCGACCCCACGCCCGCCGATGAGg 17286
17261 cCGGCTGGGGTGCGGGCGGCTACTCc 17286

ZFN-unknown-SP-7-70
17263 cCGACCCCACGCCCGCCGATGAGGGAa 17289
17263 gGCTGGGGTGCGGGCGGCTACTCCCTt 17289

ZFN-unknown-SP-6-73
17429 gACCAGCGTCCTGCGCGTTGACGTCa 17454
17429 cTGGTCGCAGGACGCGCAACTGCAGt 17454

ZFN-unknown-SP-5-75
17447 tGACGTCACCCCCAGGGGGCGGTTg 17471
17447 aCTGCAGTGGGGGTCCCCCGCCAAc 17471

ZFN-unknown-SP-6-74
17449 aCGTCACCCCCAGGGGGCGGTTGCGg 17474
17449 tGCAGTGGGGGTCCCCCGCCAACGCc 17474

ZFN-unknown-SP-5-76
17450 cGTCACCCCCAGGGGGCGGTTGCGg 17474
17450 gCAGTGGGGGTCCCCCGCCAACGCc 17474

ZFN-unknown-SP-6-75
17492 tTCCGACGACGCGTACGTGGCGTCGg 17517
17492 aAGGCTGCTGCGCATGCACCGCAGCc 17517

ZFN-unknown-SP-6-76
17962 cCACCCCGCAGAGCTCTGCGTTGCAg 17987
17962 gGTGGGGCGTCTCGAGACGCAACGTc 17987

ZFN-unknown-SP-6-77
17999 cGTCCCCCGCACGGACTGTGTGGCCt 18024
17999 gCAGGGGGCGTGCCTGACACACCGGa 18024

ZFN-unknown-SP-5-77
18000 gTCCCCCGCACGGACTGTGTGGCCt 18024
18000 cAGGGGGCGTGCCTGACACACCGGa 18024

ZFN-unknown-SP-7-71
18032 gGCCGGCGTCATGGGAAGCTGCTGGAa 18058
18032 cCGGCCGCAGTACCCTTCGACGACCTt 18058
```

ZFN-unknown-SP-5-78
18193 tCGTGTCATCTCTCGGGGGATGGTg 18217
18193 aGCACAGTAGAGAGCCCCCTACCAc 18217

ZFN-unknown-SP-6-78
18688 gGTCGTCGGCCTCGCCTGGGGGGGTc 18713
18688 cCAGCAGCCGGAGCGGACCCCCCCAg 18713

ZFN-unknown-SP-7-72
18688 gGTCGTCGGCCTCGCCTGGGGGGGTCa 18714
18688 cCAGCAGCCGGAGCGGACCCCCCCAGt 18714

ZFN-unknown-SP-6-79
18808 cGGCCTCCTCCGGCGGGCCGGCGGCc 18833
18808 gCCGGAGGAGGCCGCCCGGCCGCCGg 18833

ZFN-unknown-SP-5-79
18809 gGCCTCCTCCGGCGGGCCGGCGGCc 18833
18809 cCGGAGGAGGCCGCCCGGCCGCCGg 18833

ZFN-unknown-SP-6-80
18812 cTCCTCCGGCGGGCCGGCGGCCGGCc 18837
18812 gAGGAGGCCGCCCGGCCGCCGGCCGg 18837

ZFN-unknown-SP-5-80
18823 gGCCGGCGGCCGGCCGCCGGAGGAt 18847
18823 cCGGCCGCCGGCCGGCGGCCTCCTa 18847

ZFN-unknown-SP-5-81
18858 gCGTCGCGCATAACCTCGGCCGCCg 18882
18858 cGCAGCGCGTATTGGAGCCGGCGGc 18882

ZFN-unknown-SP-6-81
19144 aCGCAGCCCCGGTGCCGCCGCAGACc 19169
19144 tGCGTCGGGGCCACGGCGGCGTCTGg 19169

ZFN-unknown-SP-6-82
19172 aAACTTCACCGAGCTGGCCGTCTGCt 19197
19172 tTTGAAGTGGCTCGACCGGCAGACGa 19197

ZFN-unknown-SP-5-82
19222 tGACCCCGCCGAGCAGCTGCCGGAg 19246
19222 aCTGGGGCGGCTCGTCGACGGCCTc 19246

ZFN-unknown-SP-6-83
19224 aCCCCGCCGAGCAGCTGCCGGAGCGc 19249
19224 tGGGGCGGCTCGTCGACGGCCTCGCg 19249

ZFN-unknown-SP-6-84
19456 gCCCGACGGCCGCCGGGGGGTCGAGt 19481
19456 cGGGCTGCCGGCGGCCCCCCAGCTCa 19481

ZFN-unknown-SP-7-73
19627 gGCACGCGGCATGGGTGGCCGCGGCGc 19653
19627 cCGTGCGCCGTACCCACCGGCGCCGCg 19653

ZFN-unknown-SP-6-85
19667 aCGCGCCGACTCGGCGGCGGCGGCGg 19692
19667 tGCGCGGCTGAGCCGCCGCCGCCGCc 19692

ZFN-unknown-SP-7-74
19672 cCGACTCGGCGGCGGCGGCGGCCGGCa 19698
19672 gGCTGAGCCGCCGCCGCCGCCGGCCGt 19698

ZFN-unknown-SP-6-86
19873 cGTCGCCGGCGCTCACGAAGTAGTCg 19898
19873 gCAGCGGCCGCGAGTGCTTCATCAGc 19898

ZFN-unknown-SP-7-75
19916 cACCCGCGCCGTGACCCGCTGCGGGTg 19942
19916 gTGGGCGCGGCACTGGGCGACGCCCAc 19942

ZFN-unknown-SP-5-83
19928 gACCCGCTGCGGGTGTCCGCGGGGc 19952
19928 cTGGGCGACGCCCACAGGCGCCCCg 19952

ZFN-unknown-SP-7-76
19990 aGGCGCCCTCCTTCCTGGTCGGCGGAa 20016
19990 tCCGCGGGAGGAAGGACCAGCCGCCTt 20016

ZFN-unknown-SP-6-87
19994 gCCCTCCTTCCTGGTCGGCGGAAACg 20019
19994 cGGGAGGAAGGACCAGCCGCCTTTGc 20019

ZFN-unknown-SP-7-77
19994 gCCCTCCTTCCTGGTCGGCGGAAACGc 20020
19994 cGGGAGGAAGGACCAGCCGCCTTTGCg 20020

ZFN-unknown-SP-5-84
20051 gGTCCTCGTCGTGATGGTGACGGCg 20075
20051 cCAGGAGCAGCACTACCACTGCCGc 20075

ZFN-unknown-SP-6-88
20197 tGGCCTCCACGTCCAGGAGGGCGGCg 20222
20197 aCCGGAGGTGCAGGTCCTCCCGCCGc 20222

ZFN-unknown-SP-6-89
20393 gGGCTCCAGCGGGGAGGCGGCCGGGg 20418
20393 cCCGAGGTCGCCCCTCCGCCGGCCCc 20418

ZFN-unknown-SP-7-78
20419 cCGTCGCGGCGCGGGCGGCCGCGACGg 20445
20419 gGCAGCGCCGCGCCCGCCGGCGCTGCc 20445

ZFN-unknown-SP-7-79
20434 cGGCCGCGACGGCCCCGGGGGCCGAGa 20460
20434 gCCGGCGCTGCCGGGGCCCCCGGCTCt 20460

ZFN-unknown-SP-7-80
20437 cCGCGACGGCCCCGGGGGCCGAGACGt 20463
20437 gGCGCTGCCGGGGCCCCCGGCTCTGCa 20463

ZFN-unknown-SP-6-90
20632 tCTCCTCCACCCAAACGATGCCGGGg 20657
20632 aGAGGAGGTGGGTTTGCTACGGCCCc 20657

ZFN-unknown-SP-5-85
20633 cTCCTCCACCCAAACGATGCCGGGg 20657
20633 gAGGAGGTGGGTTTGCTACGGCCCc 20657

ZFN-unknown-SP-6-91
20635 cCTCCACCCAAACGATGCCGGGGGCt 20660
20635 gGAGGTGGGTTTGCTACGGCCCCCGa 20660

ZFN-unknown-SP-7-81
20701 gCGCGGCCCCAGACTTATAGGGTGCTa 20727
20701 cGCGCCGGGGTCTGAATATCCCACGAt 20727

ZFN-unknown-SP-5-86
20836 aAGCAGCGCCCCCACGCGGGCGCGc 20860

20836 tTCGTCGCGGGGGTGCGCCCGCGCg 20860

ZFN-unknown-SP-6-92
21158 cGTCCTCCGACAGCAAGGGGCCGTCg 21183
21158 gCAGGAGGCTGTCGTTCCCCGGCAGc 21183

ZFN-unknown-SP-5-87
21159 gTCCTCCGACAGCAAGGGGCCGTCg 21183
21159 cAGGAGGCTGTCGTTCCCCGGCAGc 21183

ZFN-unknown-SP-5-88
21162 cTCCGACAGCAAGGGGCCGTCGGTc 21186
21162 gAGGCTGTCGTTCCCCGGCAGCCAg 21186

ZFN-unknown-SP-5-89
21207 gAACACCAGCTGCATGTAGCGGTCg 21231
21207 cTTGTGGTCGACGTACATCGCCAGc 21231

ZFN-unknown-SP-5-90
21210 cACCAGCTGCATGTAGCGGTCGTAg 21234
21210 gTGGTCGACGTACATCGCCAGCATc 21234

ZFN-unknown-SP-5-91
21501 cTCCAGCAGCACCAGGTAGATGAAc 21525
21501 gAGGTCGTCGTGGTCCATCTACTTg 21525

ZFN-unknown-SP-7-82
21529 gTGCGGCCGACCAGGCTGTTGAGGCCg 21555
21529 cACGCCGGCTGGTCCGACAACTCCGGc 21555

ZFN-unknown-SP-7-83
21563 gCGCGACCACCTCGGCCGGCGCGACGt 21589
21563 cGCGCTGGTGGAGCCGGCCGCGCTGCa 21589

ZFN-unknown-SP-7-84
21617 gGCCCACCTCCTCCGTCTCGGCGGCCt 21643
21617 cCGGGTGGAGGAGGCAGAGCCGCCGGa 21643

ZFN-unknown-SP-6-93
21618 gCCCACCTCCTCCGTCTCGGCGGCCt 21643
21618 cGGGTGGAGGAGGCAGAGCCGCCGGa 21643

ZFN-unknown-SP-7-85
21629 cCGTCTCGGCGGCCTGGGCCGACAGGg 21655
21629 gGCAGAGCCGCCGGACCCGGCTGTCCc 21655

ZFN-unknown-SP-6-94
21894 gCGCGACCCCCGCGCCTGCGCCGCGt 21919
21894 cGCGCTGGGGGCGCGGACGCGGCGCa 21919

ZFN-unknown-SP-6-95
21897 cGACCCCCGCGCCTGCGCCGCGTCGg 21922
21897 gCTGGGGGCGCGGACGCGGCGCAGCc 21922

ZFN-unknown-SP-6-96
21903 cCGCGCCTGCGCCGCGTCGGCGTGCg 21928
21903 gGCGCGGACGCGGCGCAGCCGCACGc 21928

ZFN-unknown-SP-5-92
21903 cCGCGCCTGCGCCGCGTCGGCGTGc 21927
21903 gGCGCGGACGCGGCGCAGCCGCACg 21927

ZFN-unknown-SP-7-86
21911 gCGCCGCGTCGGCGTGCGACGCGTCGc 21937
21911 cGCGGCGCAGCCGCACGCTGCGCAGCg 21937

ZFN-unknown-SP-6-97
22214 cCGTCACGACCGTTGTGTAGATGACc 22239
22214 gGCAGTGCTGGCAACACATCTACTGg 22239

ZFN-unknown-SP-6-98
22328 tGGCAACGACCTCCGCGAAGGAGACc 22353
22328 aCCGTTGCTGGAGGCGCTTCCTCTGg 22353

ZFN-unknown-SP-7-87
22337 cCTCCGCGAAGGAGACCGTCGACGAGa 22363
22337 gGAGGCGCTTCCTCTGGCAGCTGCTCt 22363

ZFN-unknown-SP-5-93
22395 cGCCTCCAGCTCCCCGAAGAAGGTg 22419
22395 gCGGAGGTCGAGGGGCTTCTTCCAc 22419

ZFN-unknown-SP-7-88
22452 gCCCGCCGGCCCCCGGCGGCGCAGGGc 22478
22452 cGGGCGGCCGGGGGCCGCCGCGTCCCg 22478

ZFN-unknown-SP-5-94
22462 cCCCGGCGGCGCAGGGCCGCCTGCa 22486
22462 gGGGCCGCCGCGTCCCGGCGGACGt 22486

ZFN-unknown-SP-6-99
22605 cACCAGCTGCGCGTTGGCGGTGGCGt 22630
22605 gTGGTCGACGCGCAACCGCCACCGCa 22630

ZFN-unknown-SP-5-95
22605 cACCAGCTGCGCGTTGGCGGTGGCg 22629
22605 gTGGTCGACGCGCAACCGCCACCGc 22629

ZFN-unknown-SP-5-96
22665 gCGCAGCATCAACGCGTCGACGCGg 22689
22665 cGCGTCGTAGTTGCGCAGCTGCGCc 22689

ZFN-unknown-SP-5-97
22668 cAGCATCAACGCGTCGACGCGGCCc 22692
22668 gTCGTAGTTGCGCAGCTGCGCCGGg 22692

ZFN-unknown-SP-7-89
22800 tTCCACCTGCACGATGAGACGGTGGAa 22826
22800 aAGGTGGACGTGCTACTCTGCCACCTt 22826

ZFN-unknown-SP-6-100
23192 cGCACTCGTCATCCCCGATGGGACGc 23217
23192 gCGTGAGCAGTAGGGGCTACCCTGCg 23217

ZFN-unknown-SP-5-98
23195 aCTCGTCATCCCCGATGGGACGCCg 23219
23195 tGAGCAGTAGGGGCTACCCTGCGGc 23219

ZFN-unknown-SP-5-99
23198 cGTCATCCCCGATGGGACGCCGGGc 23222
23198 gCAGTAGGGGCTACCCTGCGGCCCg 23222

ZFN-unknown-SP-7-90
23240 aGGCCGCGCCGGGCGCCGACGTCGCGc 23266
23240 tCCGGCGCGGCCCGCGGCTGCAGCGCg 23266

ZFN-unknown-SP-6-101
23258 aCGTCGCGCCCGGGGCGCGGGCGGCg 23283
23258 tGCAGCGCGGGCCCCGCGCCCGCCGc 23283

ZFN-unknown-SP-7-91

23318 tCGTCTCGGCCATCTGCGTCGTGGGGc 23344
23318 aGCAGAGCCGGTAGACGCAGCACCCCg 23344

ZFN-unknown-SP-6-102
23393 cCCCCGCGACCTCTTATGCGAAGGCg 23418
23393 gGGGGCGCTGGAGAATACGCTTCCGc 23418

ZFN-unknown-SP-6-103
23396 cCGCGACCTCTTATGCGAAGGCGAAc 23421
23396 gGCGCTGGAGAATACGCTTCCGCTTg 23421

ZFN-unknown-SP-7-92
23516 tGGCGGCGGCCGTGTACGCCGCGGTGg 23542
23516 aCCGCCGCCGGCACATGCGGCGCCACc 23542

ZFN-unknown-SP-6-104
23591 tGGCGGCGGCCCGTATGGCGATGCCa 23616
23591 aCCGCCGCCGGGCATACCGCTACGGt 23616

ZFN-unknown-SP-7-93
23615 cACACGCCACGCTAATCGCCGGAAACg 23641
23615 gTGTGCGGTGCGATTAGCGGCCTTTGc 23641

ZFN-unknown-SP-5-100
23637 aAACGTCTGCGCGTGGCTGTTGCAg 23661
23637 tTTGCAGACGCGCACCGACAACGTc 23661

ZFN-unknown-SP-5-101
23832 tCGTATCGTCGGTCCGGTGCGGGCa 23856
23832 aGCATAGCAGCCAGGCCACGCCCGt 23856

ZFN-unknown-SP-5-102
23964 gCTCATCTGCCTGACGACGCTGTTc 23988
23964 cGAGTAGACGGACTGCTGCGACAAg 23988

ZFN-unknown-SP-5-103
24540 tTACGACACCGTGGAGGGGTATGCg 24564
24540 aATGCTGTGGCACCTCCCCATACGc 24564

ZFN-unknown-SP-7-94
24655 aTACTTCGGCGCGCGTGTGTGTGTGTt 24681
24655 tATGAAGCCGCGCGCACACACACACAa 24681

ZFN-unknown-SP-5-104
24655 aTACTTCGGCGCGCGTGTGTGTGTg 24679
24655 tATGAAGCCGCGCGCACACACACAc 24679

ZFN-unknown-SP-6-105
24992 cCACGACGTCAAATTCGTGGGCGGTc 25017
24992 gGTGCTGCAGTTTAAGCACCCGCCAg 25017

ZFN-unknown-SP-7-95
24992 cCACGACGTCAAATTCGTGGGCGGTCa 25018
24992 gGTGCTGCAGTTTAAGCACCCGCCAGt 25018

ZFN-unknown-SP-6-106
25022 aGACGACCTCCCCGCCGTCGGTGGTg 25047
25022 tCTGCTGGAGGGGCGGCAGCCACCAc 25047

ZFN-unknown-SP-7-96
25022 aGACGACCTCCCCGCCGTCGGTGGTGa 25048
25022 tCTGCTGGAGGGGCGGCAGCCACCACt 25048

ZFN-unknown-SP-6-107
25028 cCTCCCCGCCGTCGGTGGTGATGACg 25053
25028 gGAGGGGCGGCAGCCACCACTACTGc 25053

ZFN-unknown-SP-6-108
25031 cCCCGCCGTCGGTGGTGATGACGTTg 25056
25031 gGGGCGGCAGCCACCACTACTGCAAc 25056

ZFN-unknown-SP-7-97
25243 gGGCCCCGCCAGGCCCGGCGGGCGCTc 25269
25243 cCCGGGGCGGTCCGGGCCGCCCGCGAg 25269

ZFN-unknown-SP-5-105
25303 cCTCCGCGCCCGCGCGGTGCCTGCc 25327
25303 gGAGGCGCGGGCGCGCCACGGACGg 25327

ZFN-unknown-SP-7-98
25331 gAACGTCACCAGGTGCGGTTGCAGGGg 25357
25331 cTTGCAGTGGTCCACGCCAACGTCCCc 25357

ZFN-unknown-SP-6-109
25553 cACCAGCGCCCGATCCGGGGCGGAGc 25578
25553 gTGGTCGCGGGCTAGGCCCCGCCTCg 25578

ZFN-unknown-SP-5-106
25556 cAGCGCCCGATCCGGGGCGGAGCAt 25580
25556 gTCGCGGGCTAGGCCCCGCCTCGTa 25580

ZFN-unknown-SP-7-99
25575 gAGCATCGCCTTTTTTCGCCGGCGGCg 25601
25575 cTCGTAGCGGAAAAAAGCGGCCGCCGc 25601

ZFN-unknown-SP-6-110
25990 gCCCGGCGTCGGCGTCGTCGTTGACg 26015
25990 cGGGCCGCAGCCGCAGCAGCAACTGc 26015

ZFN-unknown-SP-6-111
25993 cGGCGTCGGCGTCGTCGTTGACGCCc 26018
25993 gCCGCAGCCGCAGCAGCAACTGCGGg 26018

ZFN-unknown-SP-5-107
26108 gTCCAGCCGCAGGCTGGCGTTGTGg 26132
26108 cAGGTCGGCGTCCGACCGCAACACc 26132

ZFN-unknown-SP-7-100
26164 gCGCGCCCCACCGCAGCGTGGAGGCGg 26190
26164 cGCGCGGGGTGGCGTCGCACCTCCGCc 26190

ZFN-unknown-SP-5-108
26166 gCGCCCCACCGCAGCGTGGAGGCGg 26190
26166 cGCGGGGTGGCGTCGCACCTCCGCc 26190

ZFN-unknown-SP-7-101
26166 gCGCCCCACCGCAGCGTGGAGGCGGTc 26192
26166 cGCGGGGTGGCGTCGCACCTCCGCCAg 26192

ZFN-unknown-SP-6-112
26168 gCCCCACCGCAGCGTGGAGGCGGTCg 26193
26168 cGGGGTGGCGTCGCACCTCCGCCAGc 26193

ZFN-unknown-SP-5-109
26168 gCCCCACCGCAGCGTGGAGGCGGTc 26192
26168 cGGGGTGGCGTCGCACCTCCGCCAg 26192

ZFN-unknown-SP-6-113
26171 cCACCGCAGCGTGGAGGCGGTCGTCa 26196
26171 gGTGGCGTCGCACCTCCGCCAGCAGt 26196

```
ZFN-unknown-SP-7-102
26224 gGCAGTCGGCCTGGTTTTGCGTCGCCg 26250
26224 cCGTCAGCCGGACCAAAACGCAGCGGc 26250

ZFN-unknown-SP-7-103
26278 gGGCGACGGCTTCGGCGGCGGACGGGg 26304
26278 cCCGCTGCCGAAGCCGCCGCCTGCCCc 26304

ZFN-unknown-SP-7-104
26281 cGACGGCTTCGGCGGCGGACGGGGGGg 26307
26281 gCTGCCGAAGCCGCCGCCTGCCCCCCc 26307

ZFN-unknown-SP-7-105
26284 cGGCTTCGGCGGCGGACGGGGGGGCGc 26310
26284 gCCGAAGCCGCCGCCTGCCCCCCCGCg 26310

ZFN-unknown-SP-6-114
26287 cTTCGGCGGCGGACGGGGGGGCGCGg 26312
26287 gAAGCCGCCGCCTGCCCCCCCGCGCc 26312

ZFN-unknown-SP-5-110
26363 cGCCTCCAGCGACACGACGAGGGAg 26387
26363 gCGGAGGTCGCTGTGCTGCTCCCTc 26387

ZFN-unknown-SP-5-111
26366 cTCCAGCGACACGACGAGGGAGCAc 26390
26366 gAGGTCGCTGTGCTGCTCCCTCGTg 26390

ZFN-unknown-SP-7-106
26473 aCGTTTCCGCCAAGATATGGGGGGGCa 26499
26473 tGCAAAGGCGGTTCTATACCCCCCCGt 26499

ZFN-unknown-SP-7-107
26474 cGTTTCCGCCAAGATATGGGGGGGCAg 26500
26474 gCAAAGGCGGTTCTATACCCCCCCGTc 26500

ZFN-unknown-SP-6-115
26474 cGTTTCCGCCAAGATATGGGGGGGCa 26499
26474 gCAAAGGCGGTTCTATACCCCCCCGt 26499

ZFN-unknown-SP-5-112
26519 cGCCGACCACATCGGGTCGGGGTCc 26543
26519 gCGGCTGGTGTAGCCCAGCCCCAGg 26543

ZFN-unknown-SP-7-108
26626 aGGCCTCAGAGAGGGCCGGGGACGCGg 26652
26626 tCCGGAGTCTCTCCCGGCCCCTGCGCc 26652

ZFN-unknown-SP-7-109
26885 gAGCAGCCGAAGAGCTCGAGGGCGGAa 26911
26885 cTCGTCGGCTTCTCGAGCTCCCGCCTt 26911

ZFN-unknown-SP-6-116
26976 cAGCAGCGGCCTCAGCTCGGGCGGCa 27001
26976 gTCGTCGCCGGAGTCGAGCCCGCCGt 27001

ZFN-unknown-SP-7-110
26979 cAGCGGCCTCAGCTCGGGCGGCAGCGa 27005
26979 gTCGCCGGAGTCGAGCCCGCCGTCGCt 27005

ZFN-unknown-SP-6-117
26994 gGGCGGCAGCGACACCGACGACAGGa 27019
26994 cCCGCCGTCGCTGTGGCTGCTGTCCt 27019

ZFN-unknown-SP-5-113
27306 cAGCGTCTGCCCCAGGGCGTAGAGa 27330
```

27306 gTCGCAGACGGGGTCCCGCATCTCt 27330

ZFN-unknown-SP-5-114
27612 tCGCAGCATCACGAGGACGTTGGCg 27636
27612 aGCGTCGTAGTGCTCCTGCAACCGc 27636

ZFN-unknown-SP-7-111
27722 cCGTCGCGACGGAGGGGGTGGTCGCGc 27748
27722 gGCAGCGCTGCCTCCCCCACCAGCGCg 27748

ZFN-unknown-SP-7-112
27847 aAGCCGCGGATGTCGTGGGTGCGGCCg 27873
27847 tTCGGCGCCTACAGCACCCACGCCGGc 27873

ZFN-unknown-SP-5-115
27884 cGCACTCCCCCACGAGCAGGGTCGc 27908
27884 gCGTGAGGGGGTGCTCGTCCCAGCg 27908

ZFN-unknown-SP-7-113
27887 aCTCCCCCACGAGCAGGGTCGCGACGa 27913
27887 tGAGGGGGTGCTCGTCCCAGCGCTGCt 27913

ZFN-unknown-SP-6-118
27992 aCCCCCCCGAGCCCCCGAAGCTGCGg 28017
27992 tGGGGGGGCTCGGGGGCTTCGACGCc 28017

ZFN-unknown-SP-7-114
28307 aGACGGCGCCGTCTCGGGGGGAGGGGc 28333
28307 tCTGCCGCGGCAGAGCCCCCCTCCCCg 28333

ZFN-unknown-SP-6-119
28307 aGACGGCGCCGTCTCGGGGGGAGGGg 28332
28307 tCTGCCGCGGCAGAGCCCCCCTCCCc 28332

ZFN-unknown-SP-6-120
28310 cGGCGCCGTCTCGGGGGGAGGGGCCg 28335
28310 gCCGCGGCAGAGCCCCCCTCCCCGGc 28335

ZFN-unknown-SP-5-116
28780 tCACGCCCTCCCGGTGTAGCTGCAg 28804
28780 aGTGCGGGAGGGCCACATCGACGTc 28804

ZFN-unknown-SP-5-117
28837 gCGTCTCGGCCAAACGTCGGCGCAg 28861
28837 cGCAGAGCCGGTTTGCAGCCGCGTc 28861

ZFN-unknown-SP-7-115
28851 cGTCGGCGCAGGGCCGCGTGGCTGGCg 28877
28851 gCAGCCGCGTCCCGGCGCACCGACCGc 28877

ZFN-unknown-SP-5-118
29043 gGGCGTCGACGAGGCGTCGGCGGGc 29067
29043 cCCGCAGCTGCTCCGCAGCCGCCCg 29067

ZFN-unknown-SP-6-121
29043 gGGCGTCGACGAGGCGTCGGCGGGCc 29068
29043 cCCGCAGCTGCTCCGCAGCCGCCCGg 29068

ZFN-unknown-SP-7-116
29180 aGCACTCGCCGCTCTTCTCGGCCGTCg 29206
29180 tCGTGAGCGGCGAGAAGAGCCGGCAGc 29206

ZFN-unknown-SP-5-119
29234 tGCCGGCGCAACTCAGGGGGCGTGa 29258
29234 aCGGCCGCGTTGAGTCCCCCGCACt 29258

ZFN-unknown-SP-6-122
29294 tGGCCCCCGAGCTGGTGCGGGCGGTg 29319
29294 aCCGGGGGCTCGACCACGCCCGCCAc 29319

ZFN-unknown-SP-5-120
29451 gGACTTCGCCCAGTTGTTGAAAACc 29475
29451 cCTGAAGCGGGTCAACAACTTTTGg 29475

ZFN-unknown-SP-7-117
30002 gTACGCCTTCCTCCCGCGGTGCCTGTt 30028
30002 cATGCGGAAGGAGGGCGCCACGGACAa 30028

ZFN-unknown-SP-7-118
30104 aGCCCCCCCCCCGCCATGGCGGGGGGg 30130
30104 tCGGGGGGGGGGCGGTACCGCCCCCCc 30130

ZFN-unknown-SP-6-123
30105 gCCCCCCCCCCGCCATGGCGGGGGGg 30130
30105 cGGGGGGGGGGCGGTACCGCCCCCCc 30130

ZFN-unknown-SP-7-119
30105 gCCCCCCCCCCGCCATGGCGGGGGGGa 30131
30105 cGGGGGGGGGGCGGTACCGCCCCCCCt 30131

ZFN-unknown-SP-6-124
30106 cCCCCCCCCCGCCATGGCGGGGGGGa 30131
30106 gGGGGGGGGGCGGTACCGCCCCCCCt 30131

ZFN-unknown-SP-5-121
30106 cCCCCCCCCCGCCATGGCGGGGGGg 30130
30106 gGGGGGGGGGCGGTACCGCCCCCCc 30130

ZFN-unknown-SP-6-125
30108 cCCCCCCCGCCATGGCGGGGGGGAAg 30133
30108 gGGGGGGGCGGTACCGCCCCCCCTTc 30133

ZFN-unknown-SP-5-122
30109 cCCCCCCGCCATGGCGGGGGGGAAg 30133
30109 gGGGGGGCGGTACCGCCCCCCCTTc 30133

ZFN-unknown-SP-5-123
30292 cCACGACGACCTCGTGCAGGTGGGc 30316
30292 gGTGCTGCTGGAGCACGTCCACCCg 30316

ZFN-unknown-SP-7-120
30295 cGACGACCTCGTGCAGGTGGGCCGTGa 30321
30295 gCTGCTGGAGCACGTCCACCCGGCACt 30321

ZFN-unknown-SP-7-121
30460 gACAGGCCTCCGCCACGGCGGGGGCGc 30486
30460 cTGTCCGGAGGCGGTGCCGCCCCCGCg 30486

ZFN-unknown-SP-6-126
30460 gACAGGCCTCCGCCACGGCGGGGGCg 30485
30460 cTGTCCGGAGGCGGTGCCGCCCCCGc 30485

ZFN-unknown-SP-6-127
30463 aGGCCTCCGCCACGGCGGGGGCGCGc 30488
30463 tCCGGAGGCGGTGCCGCCCCCGCGCg 30488

ZFN-unknown-SP-5-124
30464 gGCCTCCGCCACGGCGGGGGCGCGc 30488
30464 cCGGAGGCGGTGCCGCCCCCGCGCg 30488

ZFN-unknown-SP-7-122

```
30531 gCGCCGCCTCCTCCCGGGGGGTCGGTa 30557
30531 cGCGGCGGAGGAGGGCCCCCCAGCCAt 30557
```

ZFN-unknown-SP-5-125
```
30531 gCGCCGCCTCCTCCCGGGGGGTCGg 30555
30531 cGCGGCGGAGGAGGGCCCCCCAGCc 30555
```

ZFN-unknown-SP-6-128
```
30532 cGCCGCCTCCTCCCGGGGGGTCGGTa 30557
30532 gCGGCGGAGGAGGGCCCCCCAGCCAt 30557
```

ZFN-unknown-SP-7-123
```
30691 aGCACGCCCCGGATTCGGGGGCCGTGt 30717
30691 tCGTGCGGGGCCTAAGCCCCCGGCACa 30717
```

ZFN-unknown-SP-7-124
```
30760 gCTCGTCCGCGAGCTGTTCGGCGGCGg 30786
30760 cGAGCAGGCGCTCGACAAGCCGCCGCc 30786
```

ZFN-unknown-SP-6-129
```
30794 gGTTTCCTCCGGGGGGGAGGCAGCTt 30819
30794 cCAAAGGAGGCCCCCCCTCCGTCGAa 30819
```

ZFN-unknown-SP-6-130
```
30811 aGGCAGCTTCCAGGTGGCCGAAGGCt 30836
30811 tCCGTCGAAGGTCCACCGGCTTCCGa 30836
```

ZFN-unknown-SP-7-125
```
30842 gCACAGCAGCGGGGTCCGGGGGTGCGt 30868
30842 cGTGTCGTCGCCCCAGGCCCCCACGCa 30868
```

ZFN-unknown-SP-6-131
```
30945 gGCAGGCGCAGCGCCTTGGGCCGCGt 30970
30945 cCGTCCGCGTCGCGGAACCCGGCGCa 30970
```

ZFN-unknown-SP-6-132
```
31410 gTCCGACGGCACGCGCGACGGGGTCt 31435
31410 cAGGCTGCCGTGCGCGCTGCCCCAGa 31435
```

ZFN-unknown-SP-6-133
```
31680 gCGTGTCTCCGCTGGTGCTGTAGTAg 31705
31680 cGCACAGAGGCGACCACGACATCATc 31705
```

ZFN-unknown-SP-7-126
```
31918 cCCTAGCCACTCGCTCTGGTGGGGGCc 31944
31918 gGGATCGGTGAGCGAGACCACCCCCGg 31944
```

ZFN-unknown-SP-7-127
```
32074 gGGCTGCAGCACGGGGCGCGGGTGGCg 32100
32074 cCCGACGTCGTGCCCCGCGCCCACCGc 32100
```

ZFN-unknown-SP-6-134
```
32128 aCGCAGCAGCGCCGTCTGGGCCGCGg 32153
32128 tGCGTCGTCGCGGCAGACCCGGCGCc 32153
```

ZFN-unknown-SP-6-135
```
32131 cAGCAGCGCCGTCTGGGCCGCGGGGg 32156
32131 gTCGTCGCGGCAGACCCGGCGCCCCc 32156
```

ZFN-unknown-SP-6-136
```
32134 cAGCGCCGTCTGGGCCGCGGGGACa 32159
32134 gTCGCGGCAGACCCGGCGCCCCCTGt 32159
```

ZFN-unknown-SP-7-128
```
32190 cACAGGCCTCCGTCAGGGCCGTGGCCa 32216
32190 gTGTCCGGAGGCAGTCCCGGCACCGGt 32216
```


ZFN-unknown-SP-5-126
32224 gGACAGCCGCGGGGGGCGGGCGCGt 32248
32224 cCTGTCGGCGCCCCCCGCCCGCGCa 32248

ZFN-unknown-SP-6-137
32259 aCGCCACCGAATTCTCGTAGGAGACg 32284
32259 tGCGGTGGCTTAAGAGCATCCTCTGc 32284

ZFN-unknown-SP-7-129
32259 aCGCCACCGAATTCTCGTAGGAGACGa 32285
32259 tGCGGTGGCTTAAGAGCATCCTCTGCt 32285

ZFN-unknown-SP-5-127
32767 gAGCTGCTCCTCCGGGGAGATGGGg 32791
32767 cTCGACGAGGAGGCCCCTCTACCCc 32791

ZFN-unknown-SP-7-130
32770 cTGCTCCTCCGGGGAGATGGGGGGTc 32796
32770 gACGAGGAGGCCCCTCTACCCCCCAg 32796

ZFN-unknown-SP-5-128
32770 cTGCTCCTCCGGGGAGATGGGGGGg 32794
32770 gACGAGGAGGCCCCTCTACCCCCCc 32794

ZFN-unknown-SP-7-131
32820 cGGCCCCGCCCGAGACCGAGGACGCCt 32846
32820 gCCGGGGCGGGCTCTGGCTCCTGCGGa 32846

ZFN-unknown-SP-7-132
32889 gGGCCGCCGCCTCCGTCGCGGCGGCGg 32915
32889 cCCGGCGGCGGAGGCAGCGCCGCCGCc 32915

ZFN-unknown-SP-7-133
32892 cCGCCGCCTCCGTCGCGGCGGCGGGAg 32918
32892 gGCGGCGGAGGCAGCGCCGCCGCCCTc 32918

ZFN-unknown-SP-6-138
32893 cGCCGCCTCCGTCGCGGCGGCGGGAg 32918
32893 gCGGCGGAGGCAGCGCCGCCGCCCTc 32918

ZFN-unknown-SP-6-139
32896 cGCCTCCGTCGCGGCGGCGGGAGCCg 32921
32896 gCGGAGGCAGCGCCGCCGCCCTCGGc 32921

ZFN-unknown-SP-7-134
32901 cCGTCGCGGCGGCGGGAGCCGCGGCCc 32927
32901 gGCAGCGCCGCCGCCCTCGGCGCCGGg 32927

ZFN-unknown-SP-5-129
32923 gGCCCCCAGCCGCGCGACGTAGCGa 32947
32923 cCGGGGGTCGGCGCGCTGCATCGCt 32947

ZFN-unknown-SP-6-140
33117 cGCACGCGACCGGCAGGGAGACGAAc 33142
33117 gCGTGCGCTGGCCGTCCCTCTGCTTg 33142

ZFN-unknown-SP-6-141
33216 cCGCATCCGCCGGCACGTAGGCGGCa 33241
33216 gGCGTAGGCGGCCGTGCATCCGCCGt 33241

ZFN-unknown-SP-7-135
33216 cCGCATCCGCCGGCACGTAGGCGGCAa 33242
33216 gGCGTAGGCGGCCGTGCATCCGCCGTt 33242

ZFN-unknown-SP-6-142
33217 cGCATCCGCCGGCACGTAGGCGGCAa 33242
33217 gCGTAGGCGGCCGTGCATCCGCCGTt 33242

ZFN-unknown-SP-5-130
33217 cGCATCCGCCGGCACGTAGGCGGCa 33241
33217 gCGTAGGCGGCCGTGCATCCGCCGt 33241

ZFN-unknown-SP-6-143
33220 aTCCGCCGGCACGTAGGCGGCAAACg 33245
33220 tAGGCGGCCGTGCATCCGCCGTTTGc 33245

ZFN-unknown-SP-6-144
33309 tCGCGGCCGCCCCACCGTCGGGGCGg 33334
33309 aGCGCCGGCGGGGTGGCAGCCCCGCc 33334

ZFN-unknown-SP-6-145
33312 cGGCCGCCCCACCGTCGGGGCGGCCg 33337
33312 gCCGGCGGGGTGGCAGCCCCGCCGGc 33337

ZFN-unknown-SP-5-131
33313 gGCCGCCCCACCGTCGGGGCGGCCg 33337
33313 cCGGCGGGGTGGCAGCCCCGCCGGc 33337

ZFN-unknown-SP-6-146
33315 cCGCCCCACCGTCGGGGCGGCCGCGc 33340
33315 gGCGGGGTGGCAGCCCCGCCGGCGCg 33340

ZFN-unknown-SP-7-136
33534 gCGCGTCCTCGGGGCCCGCGGGCGCAc 33560
33534 cGCGCAGGAGCCCCGGGCGCCCGCGTg 33560

ZFN-unknown-SP-6-147
33755 gTACAACCTCCGCGGGGCCGCCGACg 33780
33755 cATGTTGGAGGCGCCCCGGCGGCTGc 33780

ZFN-unknown-SP-6-148
33758 cAACCTCCGCGGGGCCGCCGACGAGc 33783
33758 gTTGGAGGCGCCCCGGCGGCTGCTCg 33783

ZFN-unknown-SP-5-132
33806 aTGCGACGACCACATGCCGCGGGTg 33830
33806 tACGCTGCTGGTGTACGGCGCCCAc 33830

ZFN-unknown-SP-5-133
33809 cGACGACCACATGCCGCGGGTGGTg 33833
33809 gCTGCTGGTGTACGGCGCCCACCAc 33833

ZFN-unknown-SP-5-134
33932 gCCCGACTCCTTCATGCAGGAGATc 33956
33932 cGGGCTGAGGAAGTACGTCCTCTAg 33956

ZFN-unknown-SP-6-149
33992 gCCCGTCCTAACAAAGTCGGCGGGGg 34017
33992 cGGGCAGGATTGTTTCAGCCGCCCCc 34017

ZFN-unknown-SP-5-135
33992 gCCCGTCCTAACAAAGTCGGCGGGg 34016
33992 cGGGCAGGATTGTTTCAGCCGCCCc 34016

ZFN-unknown-SP-6-150
34121 cTCCGGCACCGGCATCGCGGTCGTCg 34146
34121 gAGGCCGTGGCCGTAGCGCCAGCAGc 34146

ZFN-unknown-SP-6-151
34124 cGGCACCGGCATCGCGGTCGTCGGGa 34149

34124 gCCGTGGCCGTAGCGCCAGCAGCCCt 34149

ZFN-unknown-SP-7-137
34127 cACCGGCATCGCGGTCGTCGGGAGGTa 34153
34127 gTGGCCGTAGCGCCAGCAGCCCTCCAt 34153

ZFN-unknown-SP-5-136
34244 gCACAGCCTCGCCCAGGTGCTGGCg 34268
34244 cGTGTCGGAGCGGGTCCACGACCGc 34268

ZFN-unknown-SP-6-152
34249 gCCTCGCCCAGGTGCTGGCGCTGCAc 34274
34249 cGGAGCGGGTCCACGACCGCGACGTg 34274

ZFN-unknown-SP-6-153
34313 cAACAGCAGCCAGGACTCGGCCGTGg 34338
34313 gTTGTCGTCGGTCCTGAGCCGGCACc 34338

ZFN-unknown-SP-6-154
34367 gCACCGCATCCTGGCCTCGGCGGGGg 34392
34367 cGTGGCGTAGGACCGGAGCCGCCCCc 34392

ZFN-unknown-SP-7-138
34547 gCTCGTCTCCGTGACGGTGCGCCTGCa 34573
34547 cGAGCAGAGGCACTGCCACGCGGACGt 34573

ZFN-unknown-SP-7-139
35101 cGCCTGCCCCCGCCTCAGGGGTAGCGg 35127
35101 gCGGACGGGGGCGGAGTCCCCATCGCc 35127

ZFN-unknown-SP-7-140
35107 cCCCCGCCTCAGGGGTAGCGGATAACc 35133
35107 gGGGGCGGAGTCCCCATCGCCTATTGg 35133

ZFN-unknown-SP-7-141
35244 gACCAGCGCCTCGTAAAGCGGCAGCCt 35270
35244 cTGGTCGCGGAGCATTTCGCCGTCGGa 35270

ZFN-unknown-SP-5-137
36122 gCGCAACGCAACCGGGACGATGATg 36146
36122 cGCGTTGCGTTGGCCCTGCTACTAc 36146

ZFN-unknown-SP-6-155
36160 gGGCACCGACCGTGTGGGAGAGGGGg 36185
36160 cCCGTGGCTGGCACACCCTCTCCCCc 36185

ZFN-unknown-SP-6-156
36195 gCTCAGCAGCACGCACGGGGAGGTCt 36220
36195 cGAGTCGTCGTGCGTGCCCCTCCAGa 36220

ZFN-unknown-SP-6-157
36519 gGGCCTCCCCATCGCGGGCGCTGCGt 36544
36519 cCCGGAGGGGTAGCGCCCGCGACGCa 36544

ZFN-unknown-SP-5-138
36520 gGCCTCCCCATCGCGGGCGCTGCGt 36544
36520 cCGGAGGGGTAGCGCCCGCGACGCa 36544

ZFN-unknown-SP-7-142
36831 gCGACGCCCACGGGTTGGCCGTGGCCg 36857
36831 cGCTGCGGGTGCCCAACCGGCACCGGc 36857

ZFN-unknown-SP-6-158
36832 cGACGCCCACGGGTTGGCCGTGGCCg 36857
36832 gCTGCGGGTGCCCAACCGGCACCGGc 36857

ZFN-unknown-SP-7-143
36852 tGGCCGCGAAGGGCCGCGCCGGGTCGc 36878
36852 aCCGGCGCTTCCCGGCGCGGCCCAGCg 36878

ZFN-unknown-SP-6-159
36921 tGTCCCCCGCGTACACGCCGCCGGCc 36946
36921 aCAGGGGGCGCATGTGCGGCGGCCGg 36946

ZFN-unknown-SP-5-139
37051 gCGCCGCGGCACCTGGGCGCAGCCa 37075
37051 cGCGGCGCCGTGGACCCGCGTCGGt 37075

ZFN-unknown-SP-7-144
37307 aCCCCCCCGCCCGTTTCGTGGCGGGCc 37333
37307 tGGGGGGGCGGGCAAAGCACCGCCCGg 37333

ZFN-unknown-SP-6-160
37308 cCCCCCCGCCCGTTTCGTGGCGGGCc 37333
37308 gGGGGGGCGGGCAAAGCACCGCCCGg 37333

ZFN-unknown-SP-7-145
37356 aCGCCTCCGACGCGCGCTCGGAAAACa 37382
37356 tGCGGAGGCTGCGCGCGAGCCTTTTGt 37382

ZFN-unknown-SP-6-161
37357 cGCCTCCGACGCGCGCTCGGAAAACa 37382
37357 gCGGAGGCTGCGCGCGAGCCTTTTGt 37382

ZFN-unknown-SP-6-162
37524 cCCCCGCCGCGCTCTCGCGGGCGTGc 37549
37524 gGGGGCGGCGCGAGAGCGCCCGCACg 37549

ZFN-unknown-SP-5-140
37771 gTGCAGCGCCCCGTCGAAGATGCGc 37795
37771 cACGTCGCGGGGCAGCTTCTACGCg 37795

ZFN-unknown-SP-7-146
37818 tGGCGCCCGCGTCGGGCGCCGCGGAGc 37844
37818 aCCGCGGGCGCAGCCCGCGGCGCCTCg 37844

ZFN-unknown-SP-6-163
37914 cGACGACGACGCGCCCGTTGTGGAAc 37939
37914 gCTGCTGCTGCGCGGGCAACACCTTg 37939

ZFN-unknown-SP-5-141
38218 gTGCAGCAGCCGGCCGTCGTTGCGg 38242
38218 cACGTCGTCGGCCGGCAGCAACGCc 38242

ZFN-unknown-SP-7-147
38253 cCGTCGCCACGTTGCACGCCGCCGCGt 38279
38253 gGCAGCGGTGCAACGTGCGGCGGCGCa 38279

ZFN-unknown-SP-5-142
38355 cGCAGTCCCACACGAGGGGCGGCAg 38379
38355 gCGTCAGGGTGTGCTCCCCGCCGTc 38379

ZFN-unknown-SP-5-143
38692 aAACACCGCCGGGTAGCTGCGGTCc 38716
38692 tTTGTGGCGGCCCATCGACGCCAGg 38716

ZFN-unknown-SP-7-148
38730 gGACGGCGGCTATGGTAGCCGGCGCCa 38756
38730 cCTGCCGCCGATACCATCGGCCGCGGt 38756

ZFN-unknown-SP-5-144

38746 aGCCGGCGCCATGGCGTGGCGGCCa 38770
38746 tCGGCCGCGGTACCGCACCGCCGGt 38770

ZFN-unknown-SP-6-164
38907 cGTCGGCCACGCCCGCGAAGAAGTCg 38932
38907 gCAGCCGGTGCGGGCGCTTCTTCAGc 38932

ZFN-unknown-SP-7-149
39008 gCGACCCACCGGACCCGGCCGTGGGCg 39034
39008 cGCTGGGTGGCCTGGGCCGGCACCCGc 39034

ZFN-unknown-SP-7-150
39072 tGTCCGCGCCGGGGCCGGCCGGGGCCg 39098
39072 aCAGGCGCGGCCCCGGCCGGCCCCGGc 39098

ZFN-unknown-SP-6-165
39120 tCGCGGCCTCGACGGGGTCGTGGTTc 39145
39120 aGCGCCGGAGCTGCCCCAGCACCAAg 39145

ZFN-unknown-SP-7-151
39146 aCGCCCCCGACGGCCGCGTCGATGTTc 39172
39146 tGCGGGGGCTGCCGGCGCAGCTACAAg 39172

ZFN-unknown-SP-6-166
39147 cGCCCCCGACGGCCGCGTCGATGTTc 39172
39147 gCGGGGGCTGCCGGCGCAGCTACAAg 39172

ZFN-unknown-SP-6-167
39306 gGTCCCCGGCGTGCGCGGCGAAGCGc 39331
39306 cCAGGGGCCGCACGCGCCGCTTCGCg 39331

ZFN-unknown-SP-7-152
39547 cTCCTCCTGCATATCCAGCAGGTGGCg 39573
39547 gAGGAGGACGTATAGGTCGTCCACCGc 39573

ZFN-unknown-SP-5-145
39610 cACCAGCGCCGTGACGAGGTTGGCc 39634
39610 gTGGTCGCGGCACTGCTCCAACCGg 39634

ZFN-unknown-SP-5-146
39755 gGCCGCCCGCGCGTGTCGGCGTGCg 39779
39755 cCGGCGGGCGCGCACAGCCGCACGc 39779

ZFN-unknown-SP-7-153
39789 gCACGGCCACGGACGGCTGCGTCGCCg 39815
39789 cGTGCCGGTGCCTGCCGACGCAGCGGc 39815

ZFN-unknown-SP-5-147
39789 gCACGGCCACGGACGGCTGCGTCGc 39813
39789 cGTGCCGGTGCCTGCCGACGCAGCg 39813

ZFN-unknown-SP-6-168
39799 gGACGGCTGCGTCGCCGTGGTCAGGt 39824
39799 cCTGCCGACGCAGCGGCACCAGTCCa 39824

ZFN-unknown-SP-7-154
39973 gGGCAACAGCAGGGCCAGGGGAGGCGc 39999
39973 cCCGTTGTCGTCCCGGTCCCCTCCGCg 39999

ZFN-unknown-SP-5-148
40635 gCACAACGCCTCACCGCCGGCGGAc 40659
40635 cGTGTTGCGGAGTGGCGGCCGCCTg 40659

ZFN-unknown-SP-6-169
40704 gACCAACAACAGGCGAGGGGAGGAAa 40729
40704 cTGGTTGTTGTCCGCTCCCCTCCTTt 40729

ZFN-unknown-SP-6-170
40970 cCGCCACCCACATGGCGGCGCCGGCc 40995
40970 gGCGGTGGGTGTACCGCCGCGGCCGg 40995

ZFN-unknown-SP-5-149
40971 cGCCACCCACATGGCGGCGCCGGCc 40995
40971 gCGGTGGGTGTACCGCCGCGGCCGg 40995

ZFN-unknown-SP-5-150
41850 cGCCCTCCCCCGCGGGCTGATGACg 41874
41850 gCGGGAGGGGGCGCCCGACTACTGc 41874

ZFN-unknown-SP-7-155
41851 gCCCTCCCCCGCGGGCTGATGACGTGg 41877
41851 cGGGAGGGGGCGCCCGACTACTGCACc 41877

ZFN-unknown-SP-5-151
41853 cCTCCCCCGCGGGCTGATGACGTGg 41877
41853 gGAGGGGGCGCCCGACTACTGCACc 41877

ZFN-unknown-SP-6-171
42007 cGTTTGCCCAAGCACCGACGCCGCGa 42032
42007 gCAAACGGGTTCGTGGCTGCGGCGCt 42032

ZFN-unknown-SP-7-156
42059 cGGCCACAACCCTACGCGCGGGCGGCa 42085
42059 gCCGGTGTTGGGATGCGCGCCCGCCGt 42085

ZFN-unknown-SP-5-152
42222 aCCCTGCACCACCGGGACGTTGTGt 42246
42222 tGGGACGTGGTGGCCCTGCAACACa 42246

ZFN-unknown-SP-7-157
42266 aAACCGCGCCTACTTTGTGTGCGGGGg 42292
42266 tTTGGCGCGGATGAAACACACGCCCCc 42292

ZFN-unknown-SP-7-158
42269 cCGCGCCTACTTTGTGTGCGGGGGGTc 42295
42269 gGCGCGGATGAAACACACGCCCCCCAg 42295

ZFN-unknown-SP-6-172
42269 cCGCGCCTACTTTGTGTGCGGGGGGt 42294
42269 gGCGCGGATGAAACACACGCCCCCCa 42294

ZFN-unknown-SP-7-159
42732 cCCCGGCTCCCGAGCTCGCTGGAGCCc 42758
42732 gGGGCCGAGGGCTCGAGCGACCTCGGg 42758

ZFN-unknown-SP-7-160
43035 gGCCCCCCGCTCAGGGAGCTGTGGTGg 43061
43035 cCGGGGGGCGAGTCCCTCGACACCACc 43061

ZFN-unknown-SP-5-153
43067 cTACGCCGGCGACCGGGCGCTGGAg 43091
43067 gATGCGGCCGCTGGCCCGCGACCTc 43091

ZFN-unknown-SP-5-154
43070 cGCCGGCGACCGGGCGCTGGAGGAg 43094
43070 gCGGCCGCTGGCCCGCGACCTCCTc 43094

ZFN-unknown-SP-7-161
43253 tCTCATCCACCGGGAGCGGCGCATGTc 43279
43253 aGAGTAGGTGGCCCTCGCCGCGTACAg 43279

ZFN-unknown-SP-6-173
43342 cCGCGCCCGACGAACCGACGTTGGCc 43367
43342 gGCGCGGGCTGCTTGGCTGCAACCGg 43367

ZFN-unknown-SP-6-174
43385 gTTCCGCGACGCGCTGGCGGCCGGGa 43410
43385 cAAGGCGCTGCGCGACCGCCGGCCCt 43410

ZFN-unknown-SP-7-162
43443 cTCCCGCCCAAGGACGTGCCGGTGGGg 43469
43443 gAGGGCGGGTTCCTGCACGGCCACCCc 43469

ZFN-unknown-SP-7-163
43515 tCGCAACGCCTGACCCCGGGGGGGTCc 43541
43515 aGCGTTGCGGACTGGGGCCCCCCCAGg 43541

ZFN-unknown-SP-7-164
43562 gTACCTCGGCGCGTTCCTGGGCGTGTt 43588
43562 cATGGAGCCGCGCAAGGACCCGCACAa 43588

ZFN-unknown-SP-7-165
43949 aACAGACCGCGGACACGGAGGGAGTCg 43975
43949 tTGTCTGGCGCCTGTGCCTCCCTCAGc 43975

ZFN-unknown-SP-7-166
43952 aGACCGCGGACACGGAGGGAGTCGGTt 43978
43952 tCTGGCGCCTGTGCCTCCCTCAGCCAa 43978

ZFN-unknown-SP-7-167
44020 tCGCGTCTCCACAAAAATGGGACGCAc 44046
44020 aGCGCAGAGGTGTTTTTACCCTGCGTg 44046

ZFN-unknown-SP-5-155
44022 gCGTCTCCACAAAAATGGGACGCAc 44046
44022 cGCAGAGGTGTTTTTACCCTGCGTg 44046

ZFN-unknown-SP-5-156
44092 aCGACCCCCAGCGCGGACGCGGCCa 44116
44092 tGCTGGGGGTCGCGCCTGCGCCGGt 44116

ZFN-unknown-SP-7-168
44244 cACCGGCCCCTGGGCCAGCTGCTGTTg 44270
44244 gTGGCCGGGGACCCGGTCGACGACAAc 44270

ZFN-unknown-SP-6-175
44303 tGACCTCCCCGGCCGGGGTGTAGCGc 44328
44303 aCTGGAGGGGCCGGCCCCACATCGCg 44328

ZFN-unknown-SP-6-176
44333 aCACGGCCCCCACGAGGCCGAGGTCg 44358
44333 tGTGCCGGGGGTGCTCCGGCTCCAGc 44358

ZFN-unknown-SP-6-177
44336 cGGCCCCCACGAGGCCGAGGTCGCGc 44361
44336 gCCGGGGGTGCTCCGGCTCCAGCGCg 44361

ZFN-unknown-SP-5-157
44453 gGACGTCAACGCCCGTAAGCTTGTa 44477
44453 cCTGCAGTTGCGGGCATTCGAACAt 44477

ZFN-unknown-SP-5-158
44569 gGGCCGCTACACTGGTGGGTGGCCt 44593
44569 cCCGGCGATGTGACCACCCACCGGa 44593

ZFN-unknown-SP-6-178
44907 gCGTTCCCGCTGCTCGGCCGAGGGCg 44932

44907 cGCAAGGGCGACGAGCCGGCTCCCGc 44932

ZFN-unknown-SP-6-179
44910 tTCCCGCTGCTCGGCCGAGGGCGCGc 44935
44910 aAGGGCGACGAGCCGGCTCCCGCGCg 44935

ZFN-unknown-SP-7-169
45062 gGGCCACCGCGCCATAGGCCGCGGGGc 45088
45062 cCCGGTGGCGCGGTATCCGGCGCCCCg 45088

ZFN-unknown-SP-6-180
45183 cTCCAGCCGCAGGCGGGCCGCCGGGt 45208
45183 gAGGTCGGCGTCCGCCCGGCGGCCCa 45208

ZFN-unknown-SP-6-181
45500 aGCCGGCCACCCGCCGGAGGGCGTCc 45525
45500 tCGGCCGGTGGGCGGCCTCCCGCAGg 45525

ZFN-unknown-SP-5-159
45522 gTCCAGCGCCGAGCCGTTGTCGGCg 45546
45522 cAGGTCGCGGCTCGGCAACAGCCGc 45546

ZFN-unknown-SP-7-170
45788 aGACGCCCACGGGCCACGTCGAGGCCg 45814
45788 tCTGCGGGTGCCCGGTGCAGCTCCGGc 45814

ZFN-unknown-SP-6-182
45798 gGGCCACGTCGAGGCCGACGGGGAGa 45823
45798 cCCGGTGCAGCTCCGGCTGCCCCTCt 45823

ZFN-unknown-SP-5-160
45992 cCCCGTCGTCTCCCCGCGGGAACGt 46016
45992 gGGGCAGCAGAGGGGCGCCCTTGCa 46016

ZFN-unknown-SP-5-161
46062 gGACCGCAACAGCGCGGAGGCGCCg 46086
46062 cCTGGCGTTGTCGCGCCTCCGCGGc 46086

ZFN-unknown-SP-5-162
46065 cCGCAACAGCGCGGAGGCGCCGGGg 46089
46065 gGCGTTGTCGCGCCTCCGCGGCCCc 46089

ZFN-unknown-SP-5-163
46115 tCACCTCGGCGGGGGGACGGGACGt 46139
46115 aGTGGAGCCGCCCCCCTGCCCTGCa 46139

ZFN-unknown-SP-7-171
46336 aAGCGTCCGCGCTGGGGGTCGCTGGCg 46362
46336 tTCGCAGGCGCGACCCCCAGCGACCGc 46362

ZFN-unknown-SP-6-183
46541 tCCCGGCTGCCAGTCCGCAGATGGGg 46566
46541 aGGGCCGACGGTCAGGCGTCTACCCc 46566

ZFN-unknown-SP-7-172
46713 gGCCCGCTCCGTTTTTCGGTGGGGTGc 46739
46713 cCGGGCGAGGCAAAAAGCCACCCCACg 46739

ZFN-unknown-SP-5-164
46713 gGCCCGCTCCGTTTTTCGGTGGGGt 46737
46713 cCGGGCGAGGCAAAAAGCCACCCCa 46737

ZFN-unknown-SP-5-165
47136 gCGTGTCCTCGATGCGGGGCAGAGa 47160
47136 cGCACAGGAGCTACGCCCCGTCTCt 47160

```
ZFN-unknown-SP-6-184
47160 aCCCCGCGCCGTCCTCGGGGTCGGGg 47185
47160 tGGGGCGCGGCAGGAGCCCCAGCCCc 47185

ZFN-unknown-SP-6-185
47163 cCGCGCCGTCCTCGGGGTCGGGGCGc 47188
47163 gGCGCGGCAGGAGCCCCAGCCCCGCg 47188

ZFN-unknown-SP-7-173
47230 cCGCCACCTCCCGCCGCGCTGCAGGTa 47256
47230 gGCGGTGGAGGGCGGCGCGACGTCCAt 47256

ZFN-unknown-SP-6-186
47231 cGCCACCTCCCGCCGCGCTGCAGGTa 47256
47231 gCGGTGGAGGGCGGCGCGACGTCCAt 47256

ZFN-unknown-SP-6-187
47652 cCCCGGCCGATATCTCGCCGCGGTCc 47677
47652 gGGGCCGGCTATAGAGCGGCGCCAGg 47677

ZFN-unknown-SP-5-166
47740 aGTCATCGGCTCGGGGACGTAGACg 47764
47740 tCAGTAGCCGAGCCCCTGCATCTGc 47764

ZFN-unknown-SP-6-188
47785 gGCCTCCATCAGCTGCGCGGAGGTGg 47810
47785 cCGGAGGTAGTCGACGCGCCTCCACc 47810

ZFN-unknown-SP-5-167
47788 cTCCATCAGCTGCGCGGAGGTGGTg 47812
47788 gAGGTAGTCGACGCGCCTCCACCAc 47812

ZFN-unknown-SP-6-189
47788 cTCCATCAGCTGCGCGGAGGTGGTGg 47813
47788 gAGGTAGTCGACGCGCCTCCACCACc 47813

ZFN-unknown-SP-5-168
47954 gCCTGACCGAACGCAGGCGCGTGCt 47978
47954 cGGACTGGCTTGCGTCCGCGCACGa 47978

ZFN-unknown-SP-5-169
48018 gTTCGCCCGAGAGGTGCGGGAGTTc 48042
48018 cAAGCGGGCTCTCCACGCCCTCAAg 48042

ZFN-unknown-SP-6-190
48042 cAACGCCACCAGGATTTGTGGAACGc 48067
48042 gTTGCGGTGGTCCTAAACACCTTGCg 48067

ZFN-unknown-SP-6-191
48120 gCGCGTCACCTTAATATGCGAAGTGg 48145
48120 cGCGCAGTGGAATTATACGCTTCACc 48145

ZFN-unknown-SP-7-174
48304 cAGCTGCGCCACTCCCTGAAGCTGCTg 48330
48304 gTCGACGCGGTGAGGGACTTCGACGAc 48330

ZFN-unknown-SP-5-170
48309 gCGCCACTCCCTGAAGCTGCTGCAg 48333
48309 cGCGGTGAGGGACTTCGACGACGTc 48333

ZFN-unknown-SP-6-192
48444 cTCCGGCAACATCACCGCGGCCGTGc 48469
48444 gAGGCCGTTGTAGTGGCGCCGGCACg 48469

ZFN-unknown-SP-7-175
```

```
48450 cAACATCACCGCGGCCGTGCGGATGCt 48476
48450 gTTGTAGTGGCGCCGGCACGCCTACGa 48476
```

ZFN-unknown-SP-7-176
```
48623 gCGACCCCCCCTCCCCAGGGGTCGTAg 48649
48623 cGCTGGGGGGGAGGGGTCCCCAGCATc 48649
```

ZFN-unknown-SP-6-193
```
48624 cGACCCCCCCTCCCCAGGGGTCGTAg 48649
48624 gCTGGGGGGGAGGGGTCCCCAGCATc 48649
```

ZFN-unknown-SP-7-177
```
48626 aCCCCCCCTCCCCAGGGGTCGTAGGCg 48652
48626 tGGGGGGGAGGGGTCCCCAGCATCCGc 48652
```

ZFN-unknown-SP-6-194
```
48627 cCCCCCCTCCCCAGGGGTCGTAGGCg 48652
48627 gGGGGGGAGGGGTCCCCAGCATCCGc 48652
```

ZFN-unknown-SP-7-178
```
48629 cCCCCTCCCCAGGGGTCGTAGGCGTCg 48655
48629 gGGGGAGGGGTCCCCAGCATCCGCAGc 48655
```

ZFN-unknown-SP-6-195
```
48630 cCCCTCCCCAGGGGTCGTAGGCGTCg 48655
48630 gGGGAGGGGTCCCCAGCATCCGCAGc 48655
```

ZFN-unknown-SP-7-179
```
48630 cCCCTCCCCAGGGGTCGTAGGCGTCGc 48656
48630 gGGGAGGGGTCCCCAGCATCCGCAGCg 48656
```

ZFN-unknown-SP-7-180
```
48650 gCGTCGCTGCGGAGGGTGGGGGTGTGc 48676
48650 cGCAGCGACGCCTCCCACCCCCACACg 48676
```

ZFN-unknown-SP-6-196
```
48650 gCGTCGCTGCGGAGGGTGGGGGTGTg 48675
48650 cGCAGCGACGCCTCCCACCCCCACAc 48675
```

ZFN-unknown-SP-5-171
```
48650 gCGTCGCTGCGGAGGGTGGGGGTGt 48674
48650 cGCAGCGACGCCTCCCACCCCCACa 48674
```

ZFN-unknown-SP-5-172
```
48898 cGACAACAGCCCCTGGGTGTCGACc 48922
48898 gCTGTTGTCGGGGACCCACAGCTGg 48922
```

ZFN-unknown-SP-5-173
```
49156 gTTCAACATCCCCCGGGAGACGGCg 49180
49156 cAAGTTGTAGGGGGCCCTCTGCCGc 49180
```

ZFN-unknown-SP-5-174
```
49159 cAACATCCCCCGGGAGACGGCGGCg 49183
49159 gTTGTAGGGGGCCCTCTGCCGCCGc 49183
```

ZFN-unknown-SP-7-181
```
49173 aGACGGCGGCGGAGCGGGCGGCAGTGc 49199
49173 tCTGCCGCCGCCTCGCCCGCCGTCACg 49199
```

ZFN-unknown-SP-7-182
```
49200 tGCAGGCCCAGCGCACCGCGGCCGCGg 49226
49200 aCGTCCGGGTCGCGTGGCGCCGGCGCc 49226
```

ZFN-unknown-SP-5-175
```
49205 gCCCAGCGCACCGCGGCCGCGGCGg 49229
49205 cGGGTCGCGTGGCGCCGGCGCCGCc 49229
```

ZFN-unknown-SP-7-183
49286 aTACGCCCGATCGAGCAGCAGGTGCAt 49312
49286 tATGCGGGCTAGCTCGTCGTCCACGTa 49312

ZFN-unknown-SP-7-184
49341 aGACCGCGGCGGCCGCGGCCGAAGAGg 49367
49341 tCTGGCGCCGCCGGCGCCGGCTTCTCc 49367

ZFN-unknown-SP-7-185
49344 cCGCGGCGGCCGCGGCCGAAGAGGCGg 49370
49344 gGCGCCGCCGGCGCCGGCTTCTCCGCc 49370

ZFN-unknown-SP-7-186
49350 cGGCCGCGGCCGAAGAGGCGGATGCCg 49376
49350 gCCGGCGCCGGCTTCTCCGCCTACGGc 49376

ZFN-unknown-SP-6-197
49350 cGGCCGCGGCCGAAGAGGCGGATGCc 49375
49350 gCCGGCGCCGGCTTCTCCGCCTACGg 49375

ZFN-unknown-SP-7-187
49353 cCGCGGCCGAAGAGGCGGATGCCGCGc 49379
49353 gGCGCCGGCTTCTCCGCCTACGGCGCg 49379

ZFN-unknown-SP-7-188
49428 cGCCCACCGCGGGCCCCGCCGCCGCGg 49454
49428 gCGGGTGGCGCCCGGGGCGGCGGCGCc 49454

ZFN-unknown-SP-5-176
49438 gGGCCCCGCCGCCGCGGAGATGGAg 49462
49438 cCCGGGGCGGCGGCGCCTCTACCTc 49462

ZFN-unknown-SP-6-198
49440 gCCCCGCCGCCGCGGAGATGGAGGTt 49465
49440 cGGGGCGGCGGCGCCTCTACCTCCAa 49465

ZFN-unknown-SP-7-189
49734 aCTATTCCGCCTTCGAGTGCGCCGTGc 49760
49734 tGATAAGGCGGAAGCTCACGCGGCACg 49760

ZFN-unknown-SP-5-177
49944 aGTTCGCGGCGGCCGGCGGCCGCTa 49968
49944 tCAAGCGCCGCCGGCCGCCGGCGAt 49968

ZFN-unknown-SP-6-199
50098 cGACGACGTCAACCGCGCCGCCGCCg 50123
50098 gCTGCTGCAGTTGGCGCGGCGGCGGc 50123

ZFN-unknown-SP-6-200
50101 cGACGTCAACCGCGCCGCCGCCGCGt 50126
50101 gCTGCAGTTGGCGCGGCGGCGGCGCa 50126

ZFN-unknown-SP-6-201
50140 cCACAACCTCTTCCTGTGGGAGGACc 50165
50140 gGTGTTGGAGAAGGACACCCTCCTGg 50165

ZFN-unknown-SP-5-178
50140 cCACAACCTCTTCCTGTGGGAGGAc 50164
50140 gGTGTTGGAGAAGGACACCCTCCTg 50164

ZFN-unknown-SP-5-179
50254 cCTCGACAACCGCCTGCAGCTGGGc 50278
50254 gGAGCTGTTGGCGGACGTCGACCCg 50278

ZFN-unknown-SP-7-190
50257 cGACAACCGCCTGCAGCTGGGCATGCt 50283
50257 gCTGTTGGCGGACGTCGACCCGTACGa 50283

ZFN-unknown-SP-5-180
50356 cGGCGACAACAACCTGGAGGCGCTg 50380
50356 gCCGCTGTTGTTGGACCTCCGCGAc 50380

ZFN-unknown-SP-5-181
50359 cGACAACAACCTGGAGGCGCTGTGc 50383
50359 gCTGTTGTTGGACCTCCGCGACACg 50383

ZFN-unknown-SP-7-191
50480 gGGCGGCCACTGGCGTCGACGAGGCGc 50506
50480 cCCGCCGGTGACCGCAGCTGCTCCGCg 50506

ZFN-unknown-SP-6-202
50584 cACAAACACCACCCCTGTGGGGGAGa 50609
50584 gTGTTTGTGGTGGGGACACCCCCTCt 50609

ZFN-unknown-SP-5-182
50584 cACAAACACCACCCCTGTGGGGGAg 50608
50584 gTGTTTGTGGTGGGGACACCCCCTc 50608

ZFN-unknown-SP-5-183
50587 aAACACCACCCCTGTGGGGGAGATt 50611
50587 tTTGTGGTGGGGACACCCCCTCTAa 50611

ZFN-unknown-SP-7-192
50917 cAACAACGGCGGGCCCCGTGGGTGGGt 50943
50917 gTTGTTGCCGCCCGGGGCACCCACCCa 50943

ZFN-unknown-SP-7-193
50989 cCCCCCCCCCTTCCCGTTTTGTTTGTt 51015
50989 gGGGGGGGGGAAGGGCAAAACAAACAa 51015

ZFN-unknown-SP-7-194
51062 gCGCCTCTGCCCGACCGGGCGGTGCCc 51088
51062 cGCGGAGACGGGCTGGCCCGCCACGGg 51088

ZFN-unknown-SP-6-203
51205 aGACCACCGCGCTCGGTGCGAGGTGg 51230
51205 tCTGGTGGCGCGAGCCACGCTCCACc 51230

ZFN-unknown-SP-5-184
51247 gGTCAACGACCCTCGGGGGCCGTTt 51271
51247 cCAGTTGCTGGGAGCCCCCGGCAAa 51271

ZFN-unknown-SP-6-204
51517 cTACGACACCAGCCTAGACGCGGCCa 51542
51517 gATGCTGTGGTCGGATCTGCGCCGGt 51542

ZFN-unknown-SP-6-205
51564 aCCCGGCGACGCGCGAGGGGGTGCGa 51589
51564 tGGGCCGCTGCGCGCTCCCCCACGCt 51589

ZFN-unknown-SP-5-185
51676 cGCCGTCAACAACATGATGCTGCGt 51700
51676 gCGGCAGTTGTTGTACTACGACGCa 51700

ZFN-unknown-SP-7-195
51870 gCGTTCCCGCGCCGCAGGTCGCCGTCc 51896
51870 cGCAAGGGCGCGGCGTCCAGCGGCAGg 51896

ZFN-unknown-SP-7-196
52242 tCGCCGCCGACCGCCAGGCGGGTGGGc 52268

52242 aGCGGCGGCTGGCGGTCCGCCCACCCg 52268

ZFN-unknown-SP-6-206
52243 cGCCGCCGACCGCCAGGCGGGTGGGc 52268
52243 gCGGCGGCTGGCGGTCCGCCCACCCg 52268

ZFN-unknown-SP-5-186
52312 gCGCCGCCGACACGAGGTGGAGCAg 52336
52312 cGCGGCGGCTGTGCTCCACCTCGTc 52336

ZFN-unknown-SP-7-197
52313 cGCCGCCGACACGAGGTGGAGCAGCCg 52339
52313 gCGGCGGCTGTGCTCCACCTCGTCGGc 52339

ZFN-unknown-SP-5-187
52568 tACCACCACCCCCACGCAGACACGg 52592
52568 aTGGTGGTGGGGGTGCGTCTGTGCc 52592

ZFN-unknown-SP-5-188
52664 gGGCCTCCTCTATCCGGGGCGGTCc 52688
52664 cCCGGAGGAGATAGGCCCCGCCAGg 52688

ZFN-unknown-SP-5-189
52762 cGCCCACCCCCCTCCGCCGCCGCCg 52786
52762 gCGGGTGGGGGGAGGCGGCGGCGGc 52786

ZFN-unknown-SP-7-198
52763 gCCCACCCCCCTCCGCCGCCGCCGGGa 52789
52763 cGGGTGGGGGGAGGCGGCGGCGGCCCt 52789

ZFN-unknown-SP-7-199
52869 gCGCGGCCCACGTGAACGTGGACACGg 52895
52869 cGCGCCGGGTGCACTTGCACCTGTGCc 52895

ZFN-unknown-SP-5-190
53171 aGACTACCACGGGGAGGGGGTGTGg 53195
53171 tCTGATGGTGCCCCTCCCCCACACc 53195

ZFN-unknown-SP-6-207
53420 cTCCGGCCTCGTCCTCGTTGTGGAGc 53445
53420 gAGGCCGGAGCAGGAGCAACACCTCg 53445

ZFN-unknown-SP-5-191
53423 cGGCCTCGTCCTCGTTGTGGAGCGg 53447
53423 gCCGGAGCAGGAGCAACACCTCGCc 53447

ZFN-unknown-SP-5-192
53426 cCTCGTCCTCGTTGTGGAGCGGAGa 53450
53426 gGAGCAGGAGCAACACCTCGCCTCt 53450

ZFN-unknown-SP-7-200
53644 tCCCCGCCCACGCCCCCGGGGTCGGAa 53670
53644 aGGGGCGGGTGCGGGGGCCCCAGCCTt 53670

ZFN-unknown-SP-6-208
53645 cCCCGCCCACGCCCCCGGGGTCGGAa 53670
53645 gGGGCGGGTGCGGGGGCCCCAGCCTt 53670

ZFN-unknown-SP-6-209
53924 gCCCCGCGAACATGGCGGCGTTGGCg 53949
53924 cGGGGCGCTTGTACCGCCGCAACCGc 53949

ZFN-unknown-SP-7-201
54187 tAGCCCCCGCCGAAGATGAAGTAGCGc 54213
54187 aTCGGGGGCGGCTTCTACTTCATCGCg 54213

```
ZFN-unknown-SP-6-210
54188 aGCCCCCGCCGAAGATGAAGTAGCGc 54213
54188 tCGGGGGCGGCTTCTACTTCATCGCg 54213

ZFN-unknown-SP-5-193
54342 cGTCCCCGGCCGCGAGCTGACGCGc 54366
54342 gCAGGGGCCGGCGCTCGACTGCGCg 54366

ZFN-unknown-SP-5-194
54447 gCTCACCCGCCGGCCGACGGTGGCg 54471
54447 cGAGTGGGCGGCCGGCTGCCACCGc 54471

ZFN-unknown-SP-5-195
54451 aCCCGCCGGCCGACGGTGGCGGAGg 54475
54451 tGGGCGGCCGGCTGCCACCGCCTCc 54475

ZFN-unknown-SP-7-202
54451 aCCCGCCGGCCGACGGTGGCGGAGGCg 54477
54451 tGGGCGGCCGGCTGCCACCGCCTCCGc 54477

ZFN-unknown-SP-5-196
54454 cGCCGGCCGACGGTGGCGGAGGCGa 54478
54454 gCGGCCGGCTGCCACCGCCTCCGCt 54478

ZFN-unknown-SP-6-211
54669 gGGCGCCTCCCGCAGTGGCGCGGGCg 54694
54669 cCCGCGGAGGGCGTCACCGCGCCCGc 54694

ZFN-unknown-SP-6-212
54672 cGCCTCCCGCAGTGGCGCGGGCGTGg 54697
54672 gCGGAGGGCGTCACCGCGCCCGCACc 54697

ZFN-unknown-SP-6-213
55160 aGCCGTCGACCTGCTTGAAGCGGTCg 55185
55160 tCGGCAGCTGGACGAACTTCGCCAGc 55185

ZFN-unknown-SP-5-197
55209 gTGCGACCCCTCCCGGTAGCCGTAa 55233
55209 cACGCTGGGGAGGGCCATCGGCATt 55233

ZFN-unknown-SP-6-214
55298 gCGCGTCCACCTCCTCGACGATGCAg 55323
55298 cGCGCAGGTGGAGGAGCTGCTACGTc 55323

ZFN-unknown-SP-6-215
55304 cCACCTCCTCGACGATGCAGTTGACc 55329
55304 gGTGGAGGAGCTGCTACGTCAACTGg 55329

ZFN-unknown-SP-6-216
55349 aCGCCTCCACCCGCGAGGGGTTGTAc 55374
55349 tGCGGAGGTGGGCGCTCCCCAACATg 55374

ZFN-unknown-SP-5-198
55350 cGCCTCCACCCGCGAGGGGTTGTAc 55374
55350 gCGGAGGTGGGCGCTCCCCAACATg 55374

ZFN-unknown-SP-7-203
56012 cGCCGCCCGAGGCGCGGGGGGCCGCCg 56038
56012 gCGGCGGGCTCCGCGCCCCCCGGCGGc 56038

ZFN-unknown-SP-7-204
56145 cGCCGCCCGCTAGTCTGGGGGCGAGGt 56171
56145 gCGGCGGGCGATCAGACCCCCGCTCCa 56171

ZFN-unknown-SP-5-199
```

56266 gGCCACCAGCGGACAGTCGGAGTTg 56290
56266 cCGGTGGTCGCCTGTCAGCCTCAAc 56290

ZFN-unknown-SP-5-200
56386 gACCGACGCAAAGAGTGTGGTGGCg 56410
56386 cTGGCTGCGTTTCTCACACCACCGc 56410

ZFN-unknown-SP-6-217
56386 gACCGACGCAAAGAGTGTGGTGGCGa 56411
56386 cTGGCTGCGTTTCTCACACCACCGCt 56411

ZFN-unknown-SP-6-218
56523 aACCCCCCGCGCCCATGATGAAGCGg 56548
56523 tTGGGGGGCGCGGGTACTACTTCGCc 56548

ZFN-unknown-SP-6-219
56526 cCCCCGCGCCCATGATGAAGCGGGCg 56551
56526 gGGGGCGCGGGTACTACTTCGCCCGc 56551

ZFN-unknown-SP-6-220
56793 cCGCGCCCGCCGTCAGGGCGCAGAAg 56818
56793 gGCGCGGGCGGCAGTCCCGCGTCTTc 56818

ZFN-unknown-SP-5-201
56797 gCCCGCCGTCAGGGCGCAGAAGCGc 56821
56797 cGGGCGGCAGTCCCGCGTCTTCGCg 56821

ZFN-unknown-SP-7-205
56841 tGGCGACCGCGGCGAAGGTCGCGGGCa 56867
56841 aCCGCTGGCGCCGCTTCCAGCGCCCGt 56867

ZFN-unknown-SP-5-202
56863 gGGCAGCACCTCGCCGTGGACGCTg 56887
56863 cCCGTCGTGGAGCGGCACCTGCGAc 56887

ZFN-unknown-SP-6-221
56986 gCGCTCCCGCACGTCCGCGGCGGCCc 57011
56986 cGCGAGGGCGTGCAGGCGCCGCCGGg 57011

ZFN-unknown-SP-7-206
57066 gCTCCCCGTCCTCGCTCGCGGGCGTCc 57092
57066 cGAGGGGCAGGAGCGAGCGCCCGCAGg 57092

ZFN-unknown-SP-6-222
57103 cCTCCGCCCCCCCTCCTCCGCGGCGg 57128
57103 gGAGGCGGGGGGGAGGAGGCGCCGCc 57128

ZFN-unknown-SP-5-203
57104 cTCCGCCCCCCCTCCTCCGCGGCGg 57128
57104 gAGGCGGGGGGGAGGAGGCGCCGCc 57128

ZFN-unknown-SP-6-223
57106 cCGCCCCCCCTCCTCCGCGGCGGCCc 57131
57106 gGCGGGGGGGAGGAGGCGCCGCCGGg 57131

ZFN-unknown-SP-6-224
57147 cGGCCCCCCCGGTCGCGCCGCCGGCc 57172
57147 gCCGGGGGGGCCAGCGCGGCGGCCGg 57172

ZFN-unknown-SP-7-207
57175 cAGCCGCGCCAGCACGCGGCGCAGCGc 57201
57175 gTCGGCGCGGTCGTGCGCCGCGTCGCg 57201

ZFN-unknown-SP-6-225
57204 cCTCGTCGCACTGCTCGGGGCTGACg 57229
57204 gGAGCAGCGTGACGAGCCCCGACTGc 57229

ZFN-unknown-SP-6-226
57232 cCGCCGCAGCAGCGGCGTCGTCAGGt 57257
57232 gGCGGCGTCGTCGCCGCAGCAGTCCa 57257

ZFN-unknown-SP-7-208
57238 cAGCAGCGGCGTCGTCAGGTGGTGGTc 57264
57238 gTCGTCGCCGCAGCAGTCCACCACCAg 57264

ZFN-unknown-SP-5-204
57241 cAGCGGCGTCGTCAGGTGGTGGTCg 57265
57241 gTCGCCGCAGCAGTCCACCACCAGc 57265

ZFN-unknown-SP-5-205
57244 cGGCGTCGTCAGGTGGTGGTCGTAg 57268
57244 gCCGCAGCAGTCCACCACCAGCATc 57268

ZFN-unknown-SP-5-206
57870 aACAGACCGCGCACGGGTGGGACGg 57894
57870 tTGTCTGGCGCGTGCCCACCCTGCc 57894

ZFN-unknown-SP-7-209
57870 aACAGACCGCGCACGGGTGGGACGGGt 57896
57870 tTGTCTGGCGCGTGCCCACCCTGCCCa 57896

ZFN-unknown-SP-7-210
57873 aGACCGCGCACGGGTGGGACGGGTCGc 57899
57873 tCTGGCGCGTGCCCACCCTGCCCAGCg 57899

ZFN-unknown-SP-7-211
58075 gTACGTCTGCAGCCGGATCTGCTGGTg 58101
58075 cATGCAGACGTCGGCCTAGACGACCAc 58101

ZFN-unknown-SP-7-212
58078 cGTCTGCAGCCGGATCTGCTGGTGGTa 58104
58078 gCAGACGTCGGCCTAGACGACCACCAt 58104

ZFN-unknown-SP-7-213
58270 aGCCTCCAGCCCCGGCCGCAGGTGCCg 58296
58270 tCGGAGGTCGGGGCCGGCGTCCACGGc 58296

ZFN-unknown-SP-7-214
58273 cTCCAGCCCCGGCCGCAGGTGCCGCCg 58299
58273 gAGGTCGGGGCCGGCGTCCACGGCGGc 58299

ZFN-unknown-SP-7-215
58276 cAGCCCCGGCCGCAGGTGCCGCCGCAg 58302
58276 gTCGGGGCCGGCGTCCACGGCGGCGTc 58302

ZFN-unknown-SP-7-216
58279 cCCCGGCCGCAGGTGCCGCCGCAGCAc 58305
58279 gGGGCCGGCGTCCACGGCGGCGTCGTg 58305

ZFN-unknown-SP-7-217
58455 aAACCGCGGCCGGGGGCGCGGCCGCCa 58481
58455 tTTGGCGCCGGCCCCCGCGCCGGCGGt 58481

ZFN-unknown-SP-7-218
58601 gCCCGCCCGCCCGCCCCGCCGGTGTGc 58627
58601 cGGGCGGGCGGGCGGGGCGGCCACACg 58627

ZFN-unknown-SP-5-207
58608 cGCCCGCCCCGCCGGTGTGCCGTGa 58632
58608 gCGGGCGGGGCGGCCACACGGCACt 58632

ZFN-unknown-SP-7-219
58940 cGACGCCGGCCGCCCCCGCCGCCGCCg 58966
58940 gCTGCGGCCGGCGGGGGCGGCGGCGGc 58966

ZFN-unknown-SP-7-220
58943 cGCCGGCCGCCCCCGCCGCCGCCGCCa 58969
58943 gCGGCCGGCGGGGGCGGCGGCGGCGGt 58969

ZFN-unknown-SP-5-208
59022 aAACACCTCCCCGGCGGCGCCGAGc 59046
59022 tTTGTGGAGGGGCCGCCGCGGCTCg 59046

ZFN-unknown-SP-5-209
59193 gATCTGCAGCTGCTGGGTGCGGGGg 59217
59193 cTAGACGTCGACGACCCACGCCCCc 59217

ZFN-unknown-SP-7-221
59193 gATCTGCAGCTGCTGGGTGCGGGGGCc 59219
59193 cTAGACGTCGACGACCCACGCCCCCGg 59219

ZFN-unknown-SP-5-210
59196 cTGCAGCTGCTGGGTGCGGGGGCCc 59220
59196 gACGTCGACGACCCACGCCCCCGGg 59220

ZFN-unknown-SP-5-211
59232 gGTCGCCACAAACACGCTGCTGGCg 59256
59232 cCAGCGGTGTTTGTGCGACGACCGc 59256

ZFN-unknown-SP-7-222
59318 cCCCGCCCCCGAGACTCGACGCCGCGc 59344
59318 gGGGCGGGGGCTCTGAGCTGCGGCGCg 59344

ZFN-unknown-SP-5-212
59320 cCGCCCCCGAGACTCGACGCCGCGc 59344
59320 gGCGGGGGCTCTGAGCTGCGGCGCg 59344

ZFN-unknown-SP-6-227
59654 cGGCCGCCGCCTGCACGCTGGGGGGa 59679
59654 gCCGGCGGCGGACGTGCGACCCCCCt 59679

ZFN-unknown-SP-5-213
59658 cGCCGCCTGCACGCTGGGGGGACGg 59682
59658 gCGGCGGACGTGCGACCCCCCTGCc 59682

ZFN-unknown-SP-7-223
59658 cGCCGCCTGCACGCTGGGGGGACGGCg 59684
59658 gCGGCGGACGTGCGACCCCCCTGCCGc 59684

ZFN-unknown-SP-6-228
60164 gGGCAACGTCGCCCTCGAAGCTGCTc 60189
60164 cCCGTTGCAGCGGGAGCTTCGACGAg 60189

ZFN-unknown-SP-7-224
60420 gGACCGCCGCGCGAGGAGCTGCAGCAg 60446
60420 cCTGGCGGCGCGCTCCTCGACGTCGTc 60446

ZFN-unknown-SP-5-214
60477 cGACAGCGACATGGCGTGGTTGGCc 60501
60477 gCTGTCGCTGTACCGCACCAACCGg 60501

ZFN-unknown-SP-6-229
60497 tGGCCTCGGCCAGACCGTCGCGGAAc 60522
60497 aCCGGAGCCGGTCTGGCAGCGCCTTg 60522

ZFN-unknown-SP-5-215
60573 aTCCACCAGCTGCTTGATGTTGTTc 60597

60573 tAGGTGGTCGACGAACTACAACAAg 60597

ZFN-unknown-SP-6-230
60636 cTCCAGCCTCCCCAGGGCCGTGGGCa 60661
60636 gAGGTCGGAGGGGTCCCGGCACCCGt 60661

ZFN-unknown-SP-5-216
60795 gTTCCCCAGCACGTCGCAGTCGCTg 60819
60795 cAAGGGGTCGTGCAGCGTCAGCGAc 60819

ZFN-unknown-SP-5-217
61120 gTTCCGCCGACGAGGGGCGCCGTGg 61144
61120 cAAGGCGGCTGCTCCCCGCGGCACc 61144

ZFN-unknown-SP-7-225
61121 tTCCGCCGACGAGGGGCGCCGTGGGGc 61147
61121 aAGGCGGCTGCTCCCCGCGGCACCCCg 61147

ZFN-unknown-SP-7-226
61271 gCCCGGCGTCGTCCACCTCGGTCAGGt 61297
61271 cGGGCCGCAGCAGGTGGAGCCAGTCCa 61297

ZFN-unknown-SP-6-231
61280 cGTCCACCTCGGTCAGGTGGAGGGCc 61305
61280 gCAGGTGGAGCCAGTCCACCTCCCGg 61305

ZFN-unknown-SP-5-218
61280 cGTCCACCTCGGTCAGGTGGAGGGc 61304
61280 gCAGGTGGAGCCAGTCCACCTCCCg 61304

ZFN-unknown-SP-6-232
61358 aCGCCCCGACGGCGCCGGCGCGGGCc 61383
61358 tGCGGGGCTGCCGCGGCCGCGCCCGg 61383

ZFN-unknown-SP-7-227
61364 cGACGGCGCCGGCGCGGGCCGCGGGCg 61390
61364 gCTGCCGCGGCCGCGCCCGGCGCCCGc 61390

ZFN-unknown-SP-7-228
61367 cGGCGCCGGCGCGGGCCGCGGGCGTTt 61393
61367 gCCGCGGCCGCGCCCGGCGCCCGCAAa 61393

ZFN-unknown-SP-7-229
61491 cGCCATCACCGAGGCCAGGCGCTGCTc 61517
61491 gCGGTAGTGGCTCCGGTCCGCGACGAg 61517

ZFN-unknown-SP-5-219
61715 cGACCGCCTCGAACAGCAGGCGGGc 61739
61715 gCTGGCGGAGCTTGTCGTCCGCCCg 61739

ZFN-unknown-SP-7-230
61906 aTCCCGCCCAGATGCAGGAAGGTGTTg 61932
61906 tAGGGCGGGTCTACGTCCTTCCACAAc 61932

ZFN-unknown-SP-7-231
62143 tGGCGGCCGCCGTGAAAGACGTAAACg 62169
62143 aCCGCCGGCGGCACTTTCTGCATTTGc 62169

ZFN-unknown-SP-6-233
62303 cGACGGCGACGTCCGCGTCGCCGCTg 62328
62303 gCTGCCGCTGCAGGCGCAGCGGCGAc 62328

ZFN-unknown-SP-6-234
62306 cGGCGACGTCCGCGTCGCCGCTGCGc 62331
62306 gCCGCTGCAGGCGCAGCGGCGACGCg 62331

ZFN-unknown-SP-6-235
62357 gGCCTTCGGCCGGACAGGCGCGGCCa 62382
62357 cCGGAAGCCGGCCTGTCCGCGCCGGt 62382

ZFN-unknown-SP-5-220
62528 cGACGGCTGCTGCCCGCCGTCGTCt 62552
62528 gCTGCCGACGACGGGCGGCAGCAGa 62552

ZFN-unknown-SP-5-221
62542 cGCCGTCGTCTCTCCGATGGGGTCg 62566
62542 gCGGCAGCAGAGAGGCTACCCCAGc 62566

ZFN-unknown-SP-6-236
62547 tCGTCTCTCCGATGGGGTCGAATGCc 62572
62547 aGCAGAGAGGCTACCCCAGCTTACGg 62572

ZFN-unknown-SP-5-222
62609 gAGCGGCCCCTGGTGGGGGTGGGAt 62633
62609 cTCGCCGGGGACCACCCCCACCCTa 62633

ZFN-unknown-SP-7-232
62720 cTCCAGCGGCGATTCGAGAGGCCTGCg 62746
62720 gAGGTCGCCGCTAAGCTCTCCGGACGc 62746

ZFN-unknown-SP-7-233
62852 cGTCATCGGCAGGGGGCGTGGCCGCCc 62878
62852 gCAGTAGCCGTCCCCCGCACCGGCGGg 62878

ZFN-unknown-SP-5-223
63023 cGGCCGCCCATAAGCGCGGCCTGCc 63047
63023 gCCGGCGGGTATTCGCGCCGGACGg 63047

ZFN-unknown-SP-6-237
63169 cGCCATCCCACCCCGAGCTGTTGGGt 63194
63169 gCGGTAGGGTGGGGCTCGACAACCCa 63194

ZFN-unknown-SP-7-234
63173 aTCCCACCCCGAGCTGTTGGGTGGGCg 63199
63173 tAGGGTGGGGCTCGACAACCCACCCGc 63199

ZFN-unknown-SP-7-235
63254 gACCGGCGGCGATGTTTTGTGCCGCGg 63280
63254 cTGGCCGCCGCTACAAAACACGGCGCc 63280

ZFN-unknown-SP-6-238
63284 gCCCGGCTTCCCCCGGGGGGAAGCCg 63309
63284 cGGGCCGAAGGGGGCCCCCTTCGGc 63309

ZFN-unknown-SP-6-239
63287 cGGCTTCCCCCGGGGGGAAGCCGGCg 63312
63287 gCCGAAGGGGGCCCCCTTCGGCCGc 63312

ZFN-unknown-SP-5-224
63368 cGGCACCGCCGCCTTGCCGCCGGCa 63392
63368 gCCGTGGCGGCGGAACGGCGGCCGt 63392

ZFN-unknown-SP-7-236
63569 gCGCCCCTAAGGTGTACTGCGGGGGGg 63595
63569 cGCGGGGATTCCACATGACGCCCCCCc 63595

ZFN-unknown-SP-5-225
63855 tCACGTCTACGGCACGCGGCAGTAc 63879
63855 aGTGCAGATGCCGTGCGCCGTCATg 63879

ZFN-unknown-SP-7-237

63995 tCTCCGCGGACCACTTCGAGGCGGAGg 64021
63995 aGAGGCGCCTGGTGAAGCTCCGCCTCc 64021

ZFN-unknown-SP-6-240
64002 gGACCACTTCGAGGCGGAGGTGGTGg 64027
64002 cCTGGTGAAGCTCCGCCTCCACCACc 64027

ZFN-unknown-SP-5-226
64002 gGACCACTTCGAGGCGGAGGTGGTg 64026
64002 cCTGGTGAAGCTCCGCCTCCACCAc 64026

ZFN-unknown-SP-7-238
64068 gTACTACCGCGTCTTCGTGCGAAGCGg 64094
64068 cATGATGGCGCAGAAGCACGCTTCGCc 64094

ZFN-unknown-SP-7-239
64071 cTACCGCGTCTTCGTGCGAAGCGGGCg 64097
64071 gATGGCGCAGAAGCACGCTTCGCCCGc 64097

ZFN-unknown-SP-7-240
64074 cCGCGTCTTCGTGCGAAGCGGGCGCGc 64100
64074 gGCGCAGAAGCACGCTTCGCCCGCGCg 64100

ZFN-unknown-SP-7-241
64212 gTACCGCCTCAAGCCCGGCCGCGGGAa 64238
64212 cATGGCGGAGTTCGGGCCGGCGCCCTt 64238

ZFN-unknown-SP-7-242
64246 gCCCAACCGCGCCCCCCGACGGCGTTc 64272
64246 cGGGTTGGCGCGGGGGGCTGCCGCAAg 64272

ZFN-unknown-SP-6-241
65286 gGACGACGACGAGGACGGGGACGAGg 65311
65286 cCTGCTGCTGCTCCTGCCCCTGCTCc 65311

ZFN-unknown-SP-5-227
65403 tCACGTCGACCCCGTGGTGGTGTTt 65427
65403 aGTGCAGCTGGGGCACCACCACAAa 65427

ZFN-unknown-SP-5-228
65488 tCCCTGCGGCCCGAGGCCGTCGCGc 65512
65488 aGGGACGCCGGGCTCCGGCAGCGCg 65512

ZFN-unknown-SP-6-242
65696 aACAGGCCGCCATCAAGGTGGTGTGc 65721
65696 tTGTCCGGCGGTAGTTCCACCACACg 65721

ZFN-unknown-SP-6-243
65958 gTGCCGCGGCCTCACGGCCGCGGGCc 65983
65958 cACGGCGCCGGAGTGCCGGCGCCCGg 65983

ZFN-unknown-SP-6-244
66099 gTACATCGGCGTCATCTGCGGGGGCa 66124
66099 cATGTAGCCGCAGTAGACGCCCCCGt 66124

ZFN-unknown-SP-7-243
66099 gTACATCGGCGTCATCTGCGGGGGCAa 66125
66099 cATGTAGCCGCAGTAGACGCCCCCGTt 66125

ZFN-unknown-SP-5-229
66255 gCGCCCCGCAGAGGAGTGGCTGGCg 66279
66255 cGCGGGGCGTCTCCTCACCGACCGc 66279

ZFN-unknown-SP-5-230
66336 gCGCATCACCGACCCGGAGAGGGAc 66360
66336 cGCGTAGTGGCTGGGCCTCTCCCTg 66360

ZFN-unknown-SP-6-245
66552 cGCCCTCCGCGAGCTAGACGCCGCCg 66577
66552 gCGGGAGGCGCTCGATCTGCGGCGGc 66577

ZFN-unknown-SP-7-244
66625 aAGCGCCCCCGGGAGACGCCGTCGCAt 66651
66625 tTCGCGGGGGCCCTCTGCGGCAGCGTa 66651

ZFN-unknown-SP-7-245
66647 cGCATGCCGACCCCCCGGGAGGCGCGt 66673
66647 gCGTACGGCTGGGGGGCCCTCCGCGCa 66673

ZFN-unknown-SP-5-231
66766 tCGCACCTGCTGGGGGCGGCCTGCg 66790
66766 aGCGTGGACGACCCCCGCCGGACGc 66790

ZFN-unknown-SP-5-232
66865 tGGCACCCCCCGGACGACGTGGCCg 66889
66865 aCCGTGGGGGGCCTGCTGCACCGGc 66889

ZFN-unknown-SP-7-246
66866 gGCACCCCCCGGACGACGTGGCCGCGc 66892
66866 cCGTGGGGGGCCTGCTGCACCGGCGCg 66892

ZFN-unknown-SP-6-246
67103 gCTCTGCCACACGTGGGCGGCGAACc 67128
67103 cGAGACGGTGTGCACCCGCCGCTTGg 67128

ZFN-unknown-SP-7-247
67491 cAGCCCCCGATCCCCAGGTAGTAGCCc 67517
67491 gTCGGGGGCTAGGGGTCCATCATCGGg 67517

ZFN-unknown-SP-6-247
68279 tGTTCTCCGAGTAGCGGACGACGGAc 68304
68279 aCAAGAGGCTCATCGCCTGCTGCCTg 68304

ZFN-unknown-SP-5-233
68543 cGTCCGCGGCGGCGCGCAGCCGGGg 68567
68543 gCAGGCGCCGCCGCGCGTCGGCCCc 68567

ZFN-unknown-SP-5-234
68546 cCGCGGCGGCGCGCAGCCGGGGCCc 68570
68546 gGCGCCGCCGCGCGTCGGCCCCGGg 68570

ZFN-unknown-SP-5-235
68565 gGGCCCCGACTCCCAGACGGCGGGg 68589
68565 cCCGGGGCTGAGGGTCTGCCGCCCc 68589

ZFN-unknown-SP-5-236
68568 cCCCGACTCCCAGACGGCGGGGGTg 68592
68568 gGGGCTGAGGGTCTGCCGCCCCCAc 68592

ZFN-unknown-SP-6-248
68568 cCCCGACTCCCAGACGGCGGGGGTGc 68593
68568 gGGGCTGAGGGTCTGCCGCCCCCACg 68593

ZFN-unknown-SP-6-249
68570 cCGACTCCCAGACGGCGGGGGTGCCg 68595
68570 gGCTGAGGGTCTGCCGCCCCCACGGc 68595

ZFN-unknown-SP-6-250
68690 gGGCGGCGGCGCGCGTGCAGCAGCTg 68715
68690 cCCGCCGCCGCGCGCACGTCGTCGAc 68715

ZFN-unknown-SP-6-251
68978 cCCCGCCGTCGGTCCGGAAGCAGCAg 69003
68978 gGGGCGGCAGCCAGGCCTTCGTCGTc 69003

ZFN-unknown-SP-5-237
69386 gCGCGGCCCCGCGCGGCCGCGGGGg 69410
69386 cGCGCCGGGGCGCGCCGGCGCCCCc 69410

ZFN-unknown-SP-6-252
69396 gCGCGGCCGCGGGGGGGCGGGCGCGa 69421
69396 cGCGCCGGCGCCCCCCCGCCCGCGCt 69421

ZFN-unknown-SP-6-253
69557 aCGCGCCCTCCTTCCAGCTGCTGCCc 69582
69557 tGCGCGGGAGGAAGGTCGACGACGGg 69582

ZFN-unknown-SP-6-254
69606 gCTCGACGGCACCCCCGGGGCGGACg 69631
69606 cGAGCTGCCGTGGGGGCCCCGCCTGc 69631

ZFN-unknown-SP-6-255
69609 cGACGGCACCCCCGGGGCGGACGTCg 69634
69609 gCTGCCGTGGGGGCCCCGCCTGCAGc 69634

ZFN-unknown-SP-6-256
69612 cGGCACCCCCGGGGCGGACGTCGCCa 69637
69612 gCCGTGGGGGCCCCGCCTGCAGCGGt 69637

ZFN-unknown-SP-6-257
69732 gCGCTACGTCTCGCGAGACGGCGCGg 69757
69732 cGCGATGCAGAGCGCTCTGCCGCGCc 69757

ZFN-unknown-SP-5-238
69834 gAACTACCTCTCGCGGACGCAGCGg 69858
69834 cTTGATGGAGAGCGCCTGCGTCGCc 69858

ZFN-unknown-SP-6-258
70003 gACCTGCACCAGATCCTGCGGGGCTg 70028
70003 cTGGACGTGGTCTAGGACGCCCCGAc 70028

ZFN-unknown-SP-6-259
70139 cTACGGCGGCCGCCCGGGGGACGCGt 70164
70139 gATGCCGCCGGCGGGCCCCCTGCGCa 70164

ZFN-unknown-SP-7-248
70196 tGTTCCCGCCACGCTGCGCGGCGGGGg 70222
70196 aCAAGGGCGGTGCGACGCGCCGCCCCc 70222

ZFN-unknown-SP-7-249
70198 tTCCCGCCACGCTGCGCGGCGGGGGTg 70224
70198 aAGGGCGGTGCGACGCGCCGCCCCCAc 70224

ZFN-unknown-SP-5-239
70198 tTCCCGCCACGCTGCGCGGCGGGGg 70222
70198 aAGGGCGGTGCGACGCGCCGCCCCc 70222

ZFN-unknown-SP-7-250
70355 gCCCTGCAACCCCTACCTGCGCGTGCa 70381
70355 cGGGACGTTGGGGATGGACGCGCACGt 70381

ZFN-unknown-SP-5-240
70382 gAACACCGGCGTTTCGGTGCTGTTt 70406
70382 cTTGTGGCCGCAAAGCCACGACAAa 70406

ZFN-unknown-SP-6-260
70424 gCCCCACGGCGCCCCGGGGGGCGCGa 70449

70424 cGGGGTGCCGCGGGGCCCCCCGCGCt 70449

ZFN-unknown-SP-6-261
70644 cGCACGCGGCGGATCCTGTGTCGCGc 70669
70644 gCGTGCGCCGCCTAGGACACAGCGCg 70669

ZFN-unknown-SP-5-241
70795 cGCCAGCGCCGCCCGGACGGGGACc 70819
70795 gCGGTCGCGGCGGGCCTGCCCCTGg 70819

ZFN-unknown-SP-6-262
70870 gCCCCCCGGCGCTTCTGTTGCTGGCg 70895
70870 cGGGGGGCCGCGAAGACAACGACCGc 70895

ZFN-unknown-SP-6-263
71084 cCCCAGCACCATTACCGCCGACAACg 71109
71084 gGGGTCGTGGTAATGGCGGCTGTTGc 71109

ZFN-unknown-SP-7-251
71102 cGACAACGTCCGGGCGCTCGGCATGCg 71128
71102 gCTGTTGCAGGCCCGCGAGCCGTACGc 71128

ZFN-unknown-SP-7-252
71181 cCGCATCCGCACGGAACGCAGGGTGCg 71207
71181 gGCGTAGGCGTGCCTTGCGTCCCACGc 71207

ZFN-unknown-SP-6-264
71182 cGCATCCGCACGGAACGCAGGGTGCg 71207
71182 gCGTAGGCGTGCCTTGCGTCCCACGc 71207

ZFN-unknown-SP-7-253
71278 tCGCCCCGCAGCCTATGTTCGCGGGCg 71304
71278 aGCGGGGCGTCGGATACAAGCGCCCGc 71304

ZFN-unknown-SP-5-242
71280 gCCCCGCAGCCTATGTTCGCGGGCg 71304
71280 cGGGGCGTCGGATACAAGCGCCCGc 71304

ZFN-unknown-SP-5-243
71283 cCGCAGCCTATGTTCGCGGGCGACg 71307
71283 gGCGTCGGATACAAGCGCCCGCTGc 71307

ZFN-unknown-SP-6-265
71305 aCGCCGCGGCCGAATGGCTGCGGCCc 71330
71305 tGCGGCGCCGGCTTACCGACGCCGGg 71330

ZFN-unknown-SP-7-254
71399 aGTCCTCGGCGGGTCCCTCCGCGGCCg 71425
71399 tCAGGAGCCGCCCAGGGAGGCGCCGGc 71425

ZFN-unknown-SP-7-255
71638 gGTCCGCTGCAGCTGTTGTTGTATGCg 71664
71638 cCAGGCGACGTCGACAACAACATACGc 71664

ZFN-unknown-SP-7-256
72079 aAGCGTCCGAGTTACGGGCGGCAGGGg 72105
72079 tTCGCAGGCTCAATGCCCGCCGTCCCc 72105

ZFN-unknown-SP-6-266
72459 gCACTGCCGCCGCTGCGGCGGAAACc 72484
72459 cGTGACGGCGGCGACGCCGCCTTTGg 72484

ZFN-unknown-SP-7-257
72468 cCGCTGCGGCGGAAACCGCGGCCGCGt 72494
72468 gGCGACGCCGCCTTTGGCGCCGGCGCa 72494

ZFN-unknown-SP-7-258
72480 aAACCGCGGCCGCGTGGTCGGCTGGGt 72506
72480 tTTGGCGCCGGCGCACCAGCCGACCCa 72506

ZFN-unknown-SP-7-259
72629 tGGCGGCGGCCGCGGCGGCGGTCGCCc 72655
72629 aCCGCCGCCGGCGCCGCCGCCAGCGGg 72655

ZFN-unknown-SP-7-260
72660 gGACGACCGCTCCTTCGGCGGGCGGAg 72686
72660 cCTGCTGGCGAGGAAGCCGCCCGCCTc 72686

ZFN-unknown-SP-7-261
72663 cGACCGCTCCTTCGGCGGGCGGAGGCg 72689
72663 gCTGGCGAGGAAGCCGCCCGCCTCCGc 72689

ZFN-unknown-SP-5-244
72666 cCGCTCCTTCGGCGGGCGGAGGCGg 72690
72666 gGCGAGGAAGCCGCCCGCCTCCGCc 72690

ZFN-unknown-SP-7-262
72666 cCGCTCCTTCGGCGGGCGGAGGCGGGa 72692
72666 gGCGAGGAAGCCGCCCGCCTCCGCCCt 72692

ZFN-unknown-SP-5-245
72669 cTCCTTCGGCGGGCGGAGGCGGGAt 72693
72669 gAGGAAGCCGCCCGCCTCCGCCCTa 72693

ZFN-unknown-SP-5-246
72857 gGACGCCACCCCTCGGGGGGGCGg 72881
72857 cCTGCGGTGGGGAGCCCCCCCGCc 72881

ZFN-unknown-SP-7-263
72857 gGACGCCACCCCTCGGGGGGGGCGGAg 72883
72857 cCTGCGGTGGGGAGCCCCCCCCGCCTc 72883

ZFN-unknown-SP-5-247
72859 aCGCCACCCCTCGGGGGGGGCGGAg 72883
72859 tGCGGTGGGGAGCCCCCCCCGCCTc 72883

ZFN-unknown-SP-6-267
72859 aCGCCACCCCTCGGGGGGGGCGGAGg 72884
72859 tGCGGTGGGGAGCCCCCCCCGCCTCc 72884

ZFN-unknown-SP-7-264
72936 gCACGGCGGCGGAGAAAGCGGCGGCAg 72962
72936 cGTGCCGCCGCCTCTTTCGCCGCCGTc 72962

ZFN-unknown-SP-6-268
73052 gGCACGCGCCCCGTGTTTTGTTGAGg 73077
73052 cCGTGCGCGGGGCACAAAACAACTCc 73077

ZFN-unknown-SP-5-248
73324 gTCCGCCTGCCGTGCGGGGGGGGCa 73348
73324 cAGGCGGACGGCACGCCCCCCCCGt 73348

ZFN-unknown-SP-6-269
73324 gTCCGCCTGCCGTGCGGGGGGGGCAg 73349
73324 cAGGCGGACGGCACGCCCCCCCCGTc 73349

ZFN-unknown-SP-6-270
73425 gACCTGCCTCGTCGGCGAGGGGGCGc 73450
73425 cTGGACGGAGCAGCCGCTCCCCCGCg 73450

ZFN-unknown-SP-5-249

73428 cTGCCTCGTCGGCGAGGGGGCGCTg 73452
73428 gACGGAGCAGCCGCTCCCCCGCGAc 73452

ZFN-unknown-SP-5-250
73431 cCTCGTCGGCGAGGGGGCGCTGGTa 73455
73431 gGAGCAGCCGCTCCCCCGCGACCAt 73455

ZFN-unknown-SP-5-251
73474 cAGCTCCCCCGTCAGGAGGGAGGCg 73498
73474 gTCGAGGGGGCAGTCCTCCCTCCGc 73498

ZFN-unknown-SP-5-252
73477 cTCCCCCGTCAGGAGGGAGGCGTCg 73501
73477 gAGGGGGCAGTCCTCCCTCCGCAGc 73501

ZFN-unknown-SP-6-271
73477 cTCCCCCGTCAGGAGGGAGGCGTCGa 73502
73477 gAGGGGGCAGTCCTCCCTCCGCAGCt 73502

ZFN-unknown-SP-6-272
73812 cGACGGCCCACGTCTCGATGTCGGAc 73837
73812 gCTGCCGGGTGCAGAGCTACAGCCTg 73837

ZFN-unknown-SP-6-273
73816 gGCCCACGTCTCGATGTCGGACGACa 73841
73816 cCGGGTGCAGAGCTACAGCCTGCTGt 73841

ZFN-unknown-SP-6-274
73854 gGGCGGCGTCCGGCCCGCCGGGGCGc 73879
73854 cCCGCCGCAGGCCGGGCGGCCCCGCg 73879

ZFN-unknown-SP-6-275
73947 cCACCACGACGGGCGCGCCGAGGAGc 73972
73947 gGTGGTGCTGCCCGCGCGGCTCCTCg 73972

ZFN-unknown-SP-7-265
74231 gGCAGACACATGCGCCCGAGGACGCTc 74257
74231 cCGTCTGTGTACGCGGGCTCCTGCGAg 74257

ZFN-unknown-SP-7-266
74256 tCACCGCCGCCAGGGCCTGGGCCGGAc 74282
74256 aGTGGCGGCGGTCCCGGACCCGGCCTg 74282

ZFN-unknown-SP-5-253
74440 aAACGTCGGCAACACGCGGATGCGg 74464
74440 tTTGCAGCCGTTGTGCGCCTACGCc 74464

ZFN-unknown-SP-5-254
74443 cGTCGGCAACACGCGGATGCGGGCg 74467
74443 gCAGCCGTTGTGCGCCTACGCCCGc 74467

ZFN-unknown-SP-6-276
74674 gCCCGGCGGCGGACGCGGCGCCGCCg 74699
74674 cGGGCCGCCGCCTGCGCCGCGGCGGc 74699

ZFN-unknown-SP-7-267
74803 gCCCCACGTCGCCTGGCGATGGCGCAc 74829
74803 cGGGGTGCAGCGGACCGCTACCGCGTg 74829

ZFN-unknown-SP-5-255
75126 gGTCTCCGCCGGCGCGCAGATACGc 75150
75126 cCAGAGGCGGCCGCGCGTCTATGCg 75150

ZFN-unknown-SP-5-256
75147 aCGCCTCGCCCCGGCGGCGCAGCAg 75171
75147 tGCGGAGCGGGGCCGCCGCGTCGTc 75171

ZFN-unknown-SP-6-277
75149 gCCTCGCCCCGGCGGCGCAGCAGCTg 75174
75149 cGGAGCGGGGCCGCCGCGTCGTCGAc 75174

ZFN-unknown-SP-6-278
75152 tCGCCCCGGCGGCGCAGCAGCTGCGg 75177
75152 aGCGGGGCCGCCGCGTCGTCGACGCc 75177

ZFN-unknown-SP-6-279
75155 cCCCGGCGGCGCAGCAGCTGCGGGTc 75180
75155 gGGGCCGCCGCGTCGTCGACGCCCAg 75180

ZFN-unknown-SP-5-257
75158 cGGCGGCGCAGCAGCTGCGGGTCGa 75182
75158 gCCGCCGCGTCGTCGACGCCCAGCt 75182

ZFN-unknown-SP-7-268
75177 gGTCGACCTCGTGGCCCTCGGGGGAAg 75203
75177 cCAGCTGGAGCACCGGGAGCCCCCTTc 75203

ZFN-unknown-SP-6-280
75270 cGCCCGCGCCCGTCTGGCCGCCGGCc 75295
75270 gCGGGCGCGGGCAGACCGGCGGCCGg 75295

ZFN-unknown-SP-7-269
75451 gCGCACCACCTCATCCCGCAGCCGGGc 75477
75451 cGCGTGGTGGAGTAGGGCGTCGGCCCg 75477

ZFN-unknown-SP-5-258
75453 gCACCACCTCATCCCGCAGCCGGGc 75477
75453 cGTGGTGGAGTAGGGCGTCGGCCCg 75477

ZFN-unknown-SP-5-259
75604 cAGCCCCGACACCGTGTTGGTGGTg 75628
75604 gTCGGGGCTGTGGCACAACCACCAc 75628

ZFN-unknown-SP-5-260
75607 cCCCGACACCGTGTTGGTGGTGTCc 75631
75607 gGGGCTGTGGCACAACCACCACAGg 75631

ZFN-unknown-SP-5-261
75751 gGACCTCCGCAGGGCGTCGACGCGg 75775
75751 cCTGGAGGCGTCCCGCAGCTGCGCc 75775

ZFN-unknown-SP-7-270
75752 gACCTCCGCAGGGCGTCGACGCGGCGc 75778
75752 cTGGAGGCGTCCCGCAGCTGCGCCGCg 75778

ZFN-unknown-SP-5-262
75790 gAGCGCCCCCAGGCGGCCGGCGTGc 75814
75790 cTCGCGGGGGTCCGCCGGCCGCACg 75814

ZFN-unknown-SP-5-263
75811 gTGCCGCGCCAGGGGGCCGGGGCCg 75835
75811 cACGGCGCGGTCCCCCGGCCCCGGc 75835

ZFN-unknown-SP-7-271
76158 cCACTGCGTCCTTGGTCTCGGCCGTGt 76184
76158 gGTGACGCAGGAACCAGAGCCGGCACa 76184

ZFN-unknown-SP-7-272
76350 gGGCCGCGGATATCTGCTCCGCGGCGg 76376
76350 cCCGGCGCCTATAGACGAGGCGCCGCc 76376

ZFN-unknown-SP-7-273
76529 aGGCGCCCCACGGCCTCGTGGTAGTCg 76555
76529 tCCGCGGGGTGCCGGAGCACCATCAGc 76555

ZFN-unknown-SP-7-274
76588 gAGCCCCTCCGCGATGCGCAGCAGCCg 76614
76588 cTCGGGGAGGCGCTACGCGTCGTCGGc 76614

ZFN-unknown-SP-5-264
76590 gCCCCTCCGCGATGCGCAGCAGCCg 76614
76590 cGGGGAGGCGCTACGCGTCGTCGGc 76614

ZFN-unknown-SP-6-281
76609 cAGCCGCGCCAGGGGCTCGGCGTCGa 76634
76609 gTCGGCGCGGTCCCCGAGCCGCAGCt 76634

ZFN-unknown-SP-7-275
76679 gCGCTCCAGCCGAGGCGGTGGATGGCg 76705
76679 cGCGAGGTCGGCTCCGCCACCTACCGc 76705

ZFN-unknown-SP-7-276
76682 cTCCAGCCGAGGCGGTGGATGGCGGCg 76708
76682 gAGGTCGGCTCCGCCACCTACCGCCGc 76708

ZFN-unknown-SP-7-277
76861 cGCCAGCGCCTGCAGGCGGCGCAGCTc 76887
76861 gCGGTCGCGGACGTCCGCCGCGTCGAg 76887

ZFN-unknown-SP-6-282
76887 cGACCACGTCGAACTCGGCGCGGTTc 76912
76887 gCTGGTGCAGCTTGAGCCGCGCCAAg 76912

ZFN-unknown-SP-5-265
76921 gTCCAGCGCCGCCTCGACGCCGGCg 76945
76921 cAGGTCGCGGCGGAGCTGCGGCCGc 76945

ZFN-unknown-SP-6-283
76941 cGGCGGCCCAGCGCTCGCTGCTGCCc 76966
76941 gCCGCCGGGTCGCGAGCGACGACGGg 76966

ZFN-unknown-SP-5-266
76984 cATCTTCGCCGTCAGGTCGGCGACg 77008
76984 gTAGAAGCGGCAGTCCAGCCGCTGc 77008

ZFN-unknown-SP-6-284
76984 cATCTTCGCCGTCAGGTCGGCGACGg 77009
76984 gTAGAAGCGGCAGTCCAGCCGCTGCc 77009

ZFN-unknown-SP-6-285
77007 cGGCGGCCTCAAGTTCGTCGGCGCGg 77032
77007 gCCGCCGGAGTTCAAGCAGCCGCGCc 77032

ZFN-unknown-SP-7-278
77027 gCGCGGCGTCGCGTGGCGCCGATGACc 77053
77027 cGCGCCGCAGCGCACCGCGGCTACTGg 77053

ZFN-unknown-SP-7-279
77091 gGTCCCCGGCCGTCCCTTCGGCGTGCa 77117
77091 cCAGGGGCCGGCAGGGAAGCCGCACGt 77117

ZFN-unknown-SP-5-267
77100 cCGTCCCTTCGGCGTGCAGCAGGCc 77124
77100 gGCAGGGAAGCCGCACGTCGTCCGg 77124

ZFN-unknown-SP-6-286
77178 gGGCCGCGGCATCGATGAGGGCGGCa 77203

77178 cCCGGCGCCGTAGCTACTCCCGCCGt 77203

ZFN-unknown-SP-6-287
77205 gGTCCCCCTCCGGCTGGGCGCAGGCc 77230
77205 cCAGGGGGAGGCCGACCCGCGTCCGg 77230

ZFN-unknown-SP-5-268
77206 gTCCCCCTCCGGCTGGGCGCAGGCc 77230
77206 cAGGGGGAGGCCGACCCGCGTCCGg 77230

ZFN-unknown-SP-7-280
77296 cCGCGCCTCCAGGGCCAGCCGAGTCGc 77322
77296 gGCGCGGAGGTCCCGGTCGGCTCAGCg 77322

ZFN-unknown-SP-7-281
77439 gCACGGCCGCGTCCACCGTCGCCGCGg 77465
77439 cGTGCCGGCGCAGGTGGCAGCGGCGCc 77465

ZFN-unknown-SP-7-282
77442 cGGCCGCGTCCACCGTCGCCGCGGAc 77468
77442 gCCGGCGCAGGTGGCAGCGGCGCCCTg 77468

ZFN-unknown-SP-7-283
77454 cCGTCGCCGCGGACTTGCCGTCGCGa 77480
77454 gGCAGCGGCGCCCTGAACGGCAGCGCt 77480

ZFN-unknown-SP-6-288
77478 cGACGGCGGCGCTCCCGGCGTTGATg 77503
77478 gCTGCCGCCGCGAGGGCCGCAACTAc 77503

ZFN-unknown-SP-5-269
77593 cTTCAGCACCACCACGAAGGCGGGa 77617
77593 gAAGTCGTGGTGGTGCTTCCGCCCt 77617

ZFN-unknown-SP-6-289
77596 cAGCACCACCACGAAGGCGGGATGCa 77621
77596 gTCGTGGTGGTGCTTCCGCCCTACGt 77621

ZFN-unknown-SP-7-284
77815 tAGCCCCACAAAGTCCGGGAGGGGCCg 77841
77815 aTCGGGGTGTTTCAGGCCCTCCCCGGc 77841

ZFN-unknown-SP-5-270
77866 gAACTTCTCCCGGGCGTCGTGGGCc 77890
77866 cTTGAAGAGGGCCCGCAGCACCCGg 77890

ZFN-unknown-SP-7-285
78060 cGCCCCCGTCGACGTACGGCGCGGGGc 78086
78060 gCGGGGGCAGCTGCATGCCGCGCCCCg 78086

ZFN-unknown-SP-6-290
78118 cAGCTGCTGCGCGAGCGCCGCGTCGa 78143
78118 gTCGACGACGCGCTCGCGGCGCAGCt 78143

ZFN-unknown-SP-6-291
78132 gCGCCGCGTCGAGGGCGTCGAAGCGc 78157
78132 cGCGGCGCAGCTCCCGCAGCTTCGCg 78157

ZFN-unknown-SP-5-271
78181 gCGCCGCGCCTGCTGGTCGTTGATg 78205
78181 cGCGGCGCGGACGACCAGCAACTAc 78205

ZFN-unknown-SP-5-272
78184 cCGCGCCTGCTGGTCGTTGATGCCg 78208
78184 gGCGCGGACGACCAGCAACTACGGc 78208

ZFN-unknown-SP-5-273
78402 gAGCGACGTCCCGCGTGTGTTGGTc 78426
78402 cTCGCTGCAGGGCGCACACAACCAg 78426

ZFN-unknown-SP-7-286
78657 tGCCGCCGGCGCCCGGGGCGGCGGCa 78683
78657 aCGGCGGCCGCGGGCCCCGCCGCCCGt 78683

ZFN-unknown-SP-6-292
78723 aGTCGACCCCGCGGGCGAGGGCGTCt 78748
78723 tCAGCTGGGGCGCCCGCTCCCGCAGa 78748

ZFN-unknown-SP-7-287
78811 gGCCAGCGCCTGGATCCGATGGAGAAg 78837
78811 cCGGTCGCGGACCTAGGCTACCTCTTc 78837

ZFN-unknown-SP-6-293
78835 aAGCGGCTCCGGGTGCGTCGGGGTGg 78860
78835 tTCGCCGAGGCCCACGCAGCCCCACc 78860

ZFN-unknown-SP-5-274
79140 cCCCGACGGCCGTTTTGCGGGGCAg 79164
79140 gGGGCTGCCGGCAAAACGCCCCGTc 79164

ZFN-unknown-SP-7-288
79200 gGCCGGCCACCCCGGCCTGCGTATGCg 79226
79200 cCGGCCGGTGGGCCGGACGCATACGc 79226

ZFN-unknown-SP-6-294
79288 cTCCAACAGCCCCGGGTGCGCCGGGt 79313
79288 gAGGTTGTCGGGCCCACGCGGCCCa 79313

ZFN-unknown-SP-5-275
79312 gTACGGCGACCGCAGGGCGTTGATg 79336
79312 cATGCCGCTGGCGTCCCGCAACTAc 79336

ZFN-unknown-SP-5-276
79315 cGGCGACCGCAGGGCGTTGATGGTg 79339
79315 gCCGCTGGCGTCCCGCAACTACCAc 79339

ZFN-unknown-SP-5-277
79429 gAACAGCTGCGCCAGGTCGGCGCCg 79453
79429 cTTGTCGACGCGGTCCAGCCGCGGc 79453

ZFN-unknown-SP-5-278
79432 cAGCTGCGCCAGGTCGGCGCCGGGg 79456
79432 gTCGACGCGGTCCAGCCGCGGCCCc 79456

ZFN-unknown-SP-6-295
79830 cTTCGGCCGCGTCCCCGGCGGGTGTc 79855
79830 gAAGCCGGCGCAGGGGCCGCCCACAg 79855

ZFN-unknown-SP-7-289
79830 cTTCGGCCGCGTCCCCGGCGGGTGTCt 79856
79830 gAAGCCGGCGCAGGGGCCGCCCACAGa 79856

ZFN-unknown-SP-7-290
79833 cGGCCGCGTCCCCGGCGGGTGTCTGCg 79859
79833 gCCGGCGCAGGGGCCGCCCACAGACGc 79859

ZFN-unknown-SP-7-291
79839 cGTCCCCGGCGGGTGTCTGCGGGGGCg 79865
79839 gCAGGGGCCGCCCACAGACGCCCCCGc 79865

ZFN-unknown-SP-7-292

79887 aGGCCGCGGCGTCCGGGGCCGAGGGCt 79913
79887 tCCGGCGCCGCAGGCCCCGGCTCCCGa 79913

ZFN-unknown-SP-7-293
79938 cTGCTGCCCACACATCATCGGGGGGGc 79964
79938 gACGACGGGTGTGTAGTAGCCCCCCCg 79964

ZFN-unknown-SP-7-294
79942 tGCCCACACATCATCGGGGGGGCGGTt 79968
79942 aCGGGTGTGTAGTAGCCCCCCCGCCAa 79968

ZFN-unknown-SP-5-279
79942 tGCCCACACATCATCGGGGGGGCGg 79966
79942 aCGGGTGTGTAGTAGCCCCCCCGCc 79966

ZFN-unknown-SP-5-280
79972 gTGCCCCGCCTGCGGTGTGTCGGGt 79996
79972 cACGGGGCGGACGCCACACAGCCCa 79996

ZFN-unknown-SP-6-296
79975 cCCCGCCTGCGGTGTGTCGGGTGGGc 80000
79975 gGGGCGGACGCCACACAGCCCACCCg 80000

ZFN-unknown-SP-7-295
80150 tCGCCCCGCAGGGGGTGGGTGATGGCc 80176
80150 aGCGGGGCGTCCCCCACCCACTACCGg 80176

ZFN-unknown-SP-6-297
80262 gCGCCACGGCCCCCGGGCCGGAGGGc 80287
80262 cGCGGTGCCGGGGGCCCGGCCTCCCg 80287

ZFN-unknown-SP-7-296
80305 gCCCGCCCACCGCACCTTGGGGCGGTc 80331
80305 cGGGCGGGTGGCGTGGAACCCCGCCAg 80331

ZFN-unknown-SP-5-281
80308 cGCCCACCGCACCTTGGGGCGGTCg 80332
80308 gCGGGTGGCGTGGAACCCCGCCAGc 80332

ZFN-unknown-SP-5-282
80311 cCACCGCACCTTGGGGCGGTCGTGg 80335
80311 gGTGGCGTGGAACCCCGCCAGCACc 80335

ZFN-unknown-SP-6-298
80545 cGTCTCCAGCAGCCCCGCCGTGGGCt 80570
80545 gCAGAGGTCGTCGGGGCGGCACCCGa 80570

ZFN-unknown-SP-5-283
80615 cGCAGGCCGCCGCGCGCCGCCGGGt 80639
80615 gCGTCCGGCGGCGCGCGGCGGCCCa 80639

ZFN-unknown-SP-7-297
80730 gACATCCCTCGATGGCCTCCGCGGACa 80756
80730 cTGTAGGGAGCTACCGGAGGCGCCTGt 80756

ZFN-unknown-SP-6-299
80745 cCTCCGCGGACAGCACGTCGCGGGGc 80770
80745 gGAGGCGCCTGTCGTGCAGCGCCCCg 80770

ZFN-unknown-SP-6-300
80827 cCCCCCCGGCTCCAGATCGGTCGCGa 80852
80827 gGGGGGGCCGAGGTCTAGCCAGCGCt 80852

ZFN-unknown-SP-6-301
80830 cCCCGGCTCCAGATCGGTCGCGAACt 80855
80830 gGGGCCGAGGTCTAGCCAGCGCTTGa 80855

ZFN-unknown-SP-7-298
80896 gCGCTTCATCGTGGGGTGAGGTAGCGc 80922
80896 cGCGAAGTAGCACCCCACTCCATCGCg 80922

ZFN-unknown-SP-7-299
80940 cCGCGCCCGCCACGAGCGGGGCCTGTt 80966
80940 gGCGCGGGCGGTGCTCGCCCCGGACAa 80966

ZFN-unknown-SP-6-302
81170 aACCAACTCCCAGATGGATGGGTGCg 81195
81170 tTGGTTGAGGGTCTACCTACCCACGc 81195

ZFN-unknown-SP-6-303
81269 tCTCGTCGTCCTCCACGGGGGTGTTg 81294
81269 aGAGCAGCAGGAGGTGCCCCCACAAc 81294

ZFN-unknown-SP-6-304
81272 cGTCGTCCTCCACGGGGGTGTTGGGc 81297
81272 gCAGCAGGAGGTGCCCCCACAACCCg 81297

ZFN-unknown-SP-7-300
81638 gGTCTGCGGCCGGGCTGGGGGGCGCGt 81664
81638 cCAGACGCCGGCCCGACCCCCCGCGCa 81664

ZFN-unknown-SP-5-284
81638 gGTCTGCGGCCGGGCTGGGGGGCGc 81662
81638 cCAGACGCCGGCCCGACCCCCCGCg 81662

ZFN-unknown-SP-7-301
82028 cGCATACCCCGAGGTGCGCCGTCGTCt 82054
82028 gCGTATGGGGCTCCACGCGGCAGCAGa 82054

ZFN-unknown-SP-5-285
82041 gTGCGCCGTCGTCTCGGTGACGACg 82065
82041 cACGCGGCAGCAGAGCCACTGCTGc 82065

ZFN-unknown-SP-5-286
82044 cGCCGTCGTCTCGGTGACGACGGAc 82068
82044 gCGGCAGCAGAGCCACTGCTGCCTg 82068

ZFN-unknown-SP-5-287
82190 cGACCACGGCGTACAGGCGGTGCCc 82214
82190 gCTGGTGCCGCATGTCCGCCACGGg 82214

ZFN-unknown-SP-5-288
82375 aGGCTGCTGCCCACCTCGGGAGGCt 82399
82375 tCCGACGACGGGTGGAGCCCTCCGa 82399

ZFN-unknown-SP-6-305
82378 cTGCTGCCCACCTCGGGAGGCTGGGg 82403
82378 gACGACGGGTGGAGCCCTCCGACCCc 82403

ZFN-unknown-SP-7-302
82382 tGCCCACCTCGGGAGGCTGGGGGGAg 82408
82382 aCGGGTGGAGCCCTCCGACCCCCCTc 82408

ZFN-unknown-SP-5-289
82382 tGCCCACCTCGGGAGGCTGGGGGg 82406
82382 aCGGGTGGAGCCCTCCGACCCCCc 82406

ZFN-unknown-SP-5-290
82467 gCGCAGCAGCTGCTCGATGTTGTCg 82491
82467 cGCGTCGTCGACGAGCTACAACAGc 82491

ZFN-unknown-SP-5-291
82470 cAGCAGCTGCTCGATGTTGTCGGCc 82494
82470 gTCGTCGACGAGCTACAACAGCCGg 82494

ZFN-unknown-SP-7-303
82812 gCTAGGCGGCCCGACGGGGAGCAGCGc 82838
82812 cGATCCGCCGGGCTGCCCCTCGTCGCg 82838

ZFN-unknown-SP-6-306
82839 gGACAGCGGCGTGGCCGGGGTGGTGg 82864
82839 cCTGTCGCCGCACCGGCCCCACCACc 82864

ZFN-unknown-SP-7-304
82968 aAACTCCCCCACGAGCGTGCGCCGCAg 82994
82968 tTTGAGGGGGTGCTCGCACGCGGCGTc 82994

ZFN-unknown-SP-5-292
82970 aCTCCCCCACGAGCGTGCGCCGCAg 82994
82970 tGAGGGGGTGCTCGCACGCGGCGTc 82994

ZFN-unknown-SP-5-293
83037 cGCCAGCGTCTGGCTGAGGACGCCg 83061
83037 gCGGTCGCAGACCGACTCCTGCGGc 83061

ZFN-unknown-SP-7-305
83388 cTGCTGCAGCAGCGCGAGGAGCCGTTg 83414
83388 gACGACGTCGTCGCGCTCCTCGGCAAc 83414

ZFN-unknown-SP-7-306
83391 cTGCAGCAGCGCGAGGAGCCGTTGCTg 83417
83391 gACGTCGTCGCGCTCCTCGGCAACGAc 83417

ZFN-unknown-SP-7-307
83430 gGGCGGCTCCCCTAGCTGCAGCAGGCc 83456
83430 cCCGCCGAGGGGATCGACGTCGTCCGg 83456

ZFN-unknown-SP-5-294
83439 cCCTAGCTGCAGCAGGCCGGTGACg 83463
83439 gGGATCGACGTCGTCCGGCCACTGc 83463

ZFN-unknown-SP-5-295
83442 tAGCTGCAGCAGGCCGGTGACGGCc 83466
83442 aTCGACGTCGTCCGGCCACTGCCGg 83466

ZFN-unknown-SP-6-307
83544 cGCCATCAGCGTCCCCGGGGGCGCGc 83569
83544 gCGGTAGTCGCAGGGGCCCCCGCGCg 83569

ZFN-unknown-SP-6-308
83567 cGCACGCCGACCGCGGGCTGACGCGg 83592
83567 gCGTGCGGCTGGCGCCCGACTGCGCc 83592

ZFN-unknown-SP-5-296
83568 gCACGCCGACCGCGGGCTGACGCGg 83592
83568 cGTGCGGCTGGCGCCCGACTGCGCc 83592

ZFN-unknown-SP-5-297
83571 cGCCGACCGCGGGCTGACGCGGCTt 83595
83571 gCGGCTGGCGCCCGACTGCGCCGAa 83595

ZFN-unknown-SP-5-298
83587 aCGCGGCTTAGGGCGGTCGACACGc 83611
83587 tGCGCCGAATCCCGCCAGCTGTGCg 83611

ZFN-unknown-SP-7-308
83720 gGACCACCGCGCCCCGATCGGGGGTCg 83746

83720 cCTGGTGGCGCGGGGCTAGCCCCCAGc 83746

ZFN-unknown-SP-7-309
83723 cCACCGCGCCCCGATCGGGGGTCGTCa 83749
83723 gGTGGCGCGGGGCTAGCCCCCAGCAGt 83749

ZFN-unknown-SP-5-299
83801 gGGCGGCGTCGTCGATGCGCTGTAg 83825
83801 cCCGCCGCAGCAGCTACGCGACATc 83825

ZFN-unknown-SP-5-300
83804 cGGCGTCGTCGATGCGCTGTAGGTg 83828
83804 gCCGCAGCAGCTACGCGACATCCAc 83828

ZFN-unknown-SP-6-309
84056 aCTCCACGGCACACTGGCGGACGCGc 84081
84056 tGAGGTGCCGTGTGACCGCCTGCGCg 84081

ZFN-unknown-SP-6-310
84078 gCGCACCGGCCCCAGGGCCGCCGTGg 84103
84078 cGCGTGGCCGGGGTCCCGGCGGCACc 84103

ZFN-unknown-SP-6-311
84081 cACCGGCCCCAGGGCCGCCGTGGTGc 84106
84081 gTGGCCGGGGTCCCGGCGGCACCACg 84106

ZFN-unknown-SP-7-310
84113 cGGCGGCCTCCAGCGCGTGGGTCGTGt 84139
84113 gCCGCCGGAGGTCGCGCACCCAGCACa 84139

ZFN-unknown-SP-5-301
84231 gTGCACCAGCAGCCCGGGGAGGTTc 84255
84231 cACGTGGTCGTCGGGCCCCTCCAAg 84255

ZFN-unknown-SP-6-312
84363 gCCCGACAACAGAAACGCCGCCGTGg 84388
84363 cGGGCTGTTGTCTTTGCGGCGGCACc 84388

ZFN-unknown-SP-6-313
84435 gCCCGCCGGCTCGCTCGAGGGGGAGg 84460
84435 cGGGCGGCCGAGCGAGCTCCCCCTCc 84460

ZFN-unknown-SP-7-311
84639 cTGCCGCCGCCCTATACGGAGGGGCTa 84665
84639 gACGGCGGCGGGATATGCCTCCCCGAt 84665

ZFN-unknown-SP-6-314
84937 aGCCGCCTTCGCCCGTGAGGGTGCGt 84962
84937 tCGGCGGAAGCGGGCACTCCCACGCa 84962

ZFN-unknown-SP-6-315
84940 cGCCTTCGCCCGTGAGGGTGCGTGTg 84965
84940 gCGGAAGCGGGCACTCCCACGCACAc 84965

ZFN-unknown-SP-7-312
85073 cCACTCCCGACAGCCCCGATGGCGTGg 85099
85073 gGTGAGGGCTGTCGGGGCTACCGCACc 85099

ZFN-unknown-SP-7-313
85080 cGACAGCCCCGATGGCGTGGGCCGAGa 85106
85080 gCTGTCGGGGCTACCGCACCCGGCTCt 85106

ZFN-unknown-SP-6-316
85172 cGCCCGCCCATCGCTCGCCGCGACGg 85197
85172 gCGGGCGGGTAGCGAGCGGCGCTGCc 85197

ZFN-unknown-SP-7-314
85515 gGCAGACCGAGCGACTGGCCGAGGCCt 85541
85515 cCGTCTGGCTCGCTGACCGGCTCCGGa 85541

ZFN-unknown-SP-5-302
85696 gACCAACTACGGCGGGACGCGGGCc 85720
85696 cTGGTTGATGCCGCCCTGCGCCCGg 85720

ZFN-unknown-SP-7-315
85843 tAGCGTCACCGCCGTCTGCAGCGGACc 85869
85843 aTCGCAGTGGCGGCAGACGTCGCCTGg 85869

ZFN-unknown-SP-5-303
85845 gCGTCACCGCCGTCTGCAGCGGACc 85869
85845 cGCAGTGGCGGCAGACGTCGCCTGg 85869

ZFN-unknown-SP-7-316
86060 cTCCCCCCGCGCGGACTGGAGGCGGCc 86086
86060 gAGGGGGGCGCGCCTGACCTCCGCCGg 86086

ZFN-unknown-SP-5-304
86317 cGCCGCCTTCGCAGAGCTGGGTGTc 86341
86317 gCGGCGGAAGCGTCTCGACCCACAg 86341

ZFN-unknown-SP-7-317
86670 tACACCCCCCTGACTCAGGAGATAGGc 86696
86670 aTGTGGGGGGACTGAGTCCTCTATCCg 86696

ZFN-unknown-SP-6-317
86727 tCGCACCCCACCCCTGTGTGCCGGGg 86752
86727 aGCGTGGGGTGGGGACACACGGCCCc 86752

ZFN-unknown-SP-5-305
86728 cGCACCCCACCCCTGTGTGCCGGGg 86752
86728 gCGTGGGGTGGGGACACACGGCCCc 86752

ZFN-unknown-SP-7-318
86959 cCGCCTCCCACGGGCCCGCCGCTGTTt 86985
86959 gGCGGAGGGTGCCCGGGCGGCGACAAa 86985

ZFN-unknown-SP-6-318
86960 cGCCTCCCACGGGCCCGCCGCTGTTt 86985
86960 gCGGAGGGTGCCCGGGCGGCGACAAa 86985

ZFN-unknown-SP-7-319
87037 cTGCCGCATCCGCCCTCGCCGGAGCGc 87063
87037 gACGGCGTAGGCGGGAGCGGCCTCGCg 87063

ZFN-unknown-SP-6-319
87320 aAACGTCGCAGCCGGCGGGGATGGCc 87345
87320 tTTGCAGCGTCGGCCGCCCCTACCGg 87345

ZFN-unknown-SP-7-320
87347 aACCGCCGTCGTGGCGCTCGGCGGAAc 87373
87347 tTGGCGGCAGCACCGCGAGCCGCCTTg 87373

ZFN-unknown-SP-6-320
87536 gGGCGGCGCCGAGAAGGACGTCGGGg 87561
87536 cCCGCCGCGGCTCTTCCTGCAGCCCc 87561

ZFN-unknown-SP-5-306
87698 cAGCGACTCCGACTCGCGGTCGGAc 87722
87698 gTCGCTGAGGCTGAGCGCCAGCCTg 87722

ZFN-unknown-SP-7-321

87700 gCGACTCCGACTCGCGGTCGGACGACt 87726
87700 cGCTGAGGCTGAGCGCCAGCCTGCTGa 87726

ZFN-unknown-SP-6-321
87701 cGACTCCGACTCGCGGTCGGACGACt 87726
87701 gCTGAGGCTGAGCGCCAGCCTGCTGa 87726

ZFN-unknown-SP-7-322
87874 cGTCGGCCGACTCCGATTCCGCGGCCc 87900
87874 gCAGCCGGCTGAGGCTAAGGCGCCGGg 87900

ZFN-unknown-SP-7-323
87901 aCGCCGCCGCACCCCAGGCGGACGTGg 87927
87901 tGCGGCGGCGTGGGGTCCGCCTGCACc 87927

ZFN-unknown-SP-7-324
87904 cCGCCGCACCCCAGGCGGACGTGGCGc 87930
87904 gGCGGCGTGGGGTCCGCCTGCACCGCg 87930

ZFN-unknown-SP-6-322
87991 tCACGCCCGAGAACGCGGAGGCGGTg 88016
87991 aGTGCGGGCTCTTGCGCCTCCGCCAc 88016

ZFN-unknown-SP-7-325
87991 tCACGCCCGAGAACGCGGAGGCGGTGg 88017
87991 aGTGCGGGCTCTTGCGCCTCCGCCACc 88017

ZFN-unknown-SP-6-323
88104 gTGCCCCCACGAACCTTCGGCAGCGc 88129
88104 cACGGGGGTGCTTGGAAGCCGTCGCg 88129

ZFN-unknown-SP-6-324
88127 cGCCCCCCGCCTCACGGAGGACGACt 88152
88127 gCGGGGGGCGGAGTGCCTCCTGCTGa 88152

ZFN-unknown-SP-5-307
88128 gCCCCCCGCCTCACGGAGGACGACt 88152
88128 cGGGGGGCGGAGTGCCTCCTGCTGa 88152

ZFN-unknown-SP-6-325
88641 aTGCGCCACATCGCCCTGGGGCGACa 88666
88641 tACGCGGTGTAGCGGGACCCCGCTGt 88666

ZFN-unknown-SP-5-308
88943 gGTCCTCGACTCCCTGGTGGCGGCg 88967
88943 cCAGGAGCTGAGGGACCACCGCCGc 88967

ZFN-unknown-SP-6-326
88943 gGTCCTCGACTCCCTGGTGGCGGCGc 88968
88943 cCAGGAGCTGAGGGACCACCGCCGCg 88968

ZFN-unknown-SP-5-309
88980 aGCACGCGCCCCACCGGGGCGTGCg 89004
88980 tCGTGCGCGGGGTGGCCCCGCACGc 89004

ZFN-unknown-SP-6-327
89054 gGGCGTCCTCGCCGGCGAGGAGGCCc 89079
89054 cCCGCAGGAGCGGCCGCTCCTCCGGg 89079

ZFN-unknown-SP-5-310
89096 cTTCAGCGCCCTCTGGATGCCGGAc 89120
89096 gAAGTCGCGGGAGACCTACGGCCTg 89120

ZFN-unknown-SP-6-328
89372 cATCTACGACACGCAAGGGGCGGCCa 89397
89372 gTAGATGCTGTGCGTTCCCCGCCGGt 89397

ZFN-unknown-SP-5-311
89522 gCTCCGCGACGCCGTGCAGGCGTGc 89546
89522 cGAGGCGCTGCGGCACGTCCGCACg 89546

ZFN-unknown-SP-7-326
89709 aACACACACATCGCCGAGGTGATGCTg 89735
89709 tTGTGTGTGTAGCGGCTCCACTACGAc 89735

ZFN-unknown-SP-5-312
89711 cACACACATCGCCGAGGTGATGCTg 89735
89711 gTGTGTGTAGCGGCTCCACTACGAc 89735

ZFN-unknown-SP-5-313
89714 aCACATCGCCGAGGTGATGCTGCTc 89738
89714 tGTGTAGCGGCTCCACTACGACGAg 89738

ZFN-unknown-SP-7-327
89786 cTTCAGCCACTTTAAGCGCAGCATGTa 89812
89786 gAAGTCGGTGAAATTCGCGTCGTACAt 89812

ZFN-unknown-SP-7-328
89947 cCGCCTCGGCCCAGATCTCGGACGTCa 89973
89947 gGCGGAGCCGGGTCTAGAGCCTGCAGt 89973

ZFN-unknown-SP-6-329
90767 cGGCCGCGGACGACCTGGTGACGGAa 90792
90767 gCCGGCGCCTGCTGGACCACTGCCTt 90792

ZFN-unknown-SP-5-314
90885 cTACAACATCATCCAGCTGGTGCTc 90909
90885 gATGTTGTAGTAGGTCGACCACGAg 90909

ZFN-unknown-SP-6-330
91137 cGACCTCATCAGCCGCGACGAGGCCg 91162
91137 gCTGGAGTAGTCGGCGCTGCTCCGGc 91162

ZFN-unknown-SP-6-331
91140 cCTCATCAGCCGCGACGAGGCCGTGc 91165
91140 gGAGTAGTCGGCGCTGCTCCGGCACg 91165

ZFN-unknown-SP-7-329
91185 cATCTACAACAACTACCTCGGGGGCCa 91211
91185 gTAGATGTTGTTGATGGAGCCCCCGGt 91211

ZFN-unknown-SP-7-330
91458 cACCAACTTCTTCGAGTGCCGCAGCAc 91484
91458 gTGGTTGAAGAAGCTCACGGCGTCGTg 91484

ZFN-unknown-SP-6-332
91476 cCGCAGCACCTCGTACGCCGGGGCCg 91501
91476 gGCGTCGTGGAGCATGCGGCCCCGGc 91501

ZFN-unknown-SP-7-331
91497 gGCCGTCGTCAACGATCTGTGAGGGTc 91523
91497 cCGGCAGCAGTTGCTAGACACTCCCAg 91523

ZFN-unknown-SP-5-315
92031 gTGCTCCACCAGCTCGGCGATGGTc 92055
92031 cACGAGGTGGTCGAGCCGCTACCAg 92055

ZFN-unknown-SP-5-316
92225 cACCGGCGACGTCCGGGGGCTGGAg 92249
92225 gTGGCCGCTGCAGGCCCCCGACCTc 92249

ZFN-unknown-SP-5-317
92228 cGGCGACGTCCGGGGGCTGGAGGGg 92252
92228 gCCGCTGCAGGCCCCCGACCTCCCc 92252

ZFN-unknown-SP-6-333
92450 cGTCCTCGACGGACGCGTAGGTGTTa 92475
92450 gCAGGAGCTGCCTGCGCATCCACAAt 92475

ZFN-unknown-SP-7-332
92505 gAACAGCGCCAGGAACTGCGGGTAACt 92531
92505 cTTGTCGCGGTCCTTGACGCCCATTGa 92531

ZFN-unknown-SP-5-318
92541 gTACTGCAGCAGGTCGCGGCAGTGa 92565
92541 cATGACGTCGTCCAGCGCCGTCACt 92565

ZFN-unknown-SP-7-333
93466 aACCGCCGGCGCGCGCCGGTGCAGGCc 93492
93466 tTGGCGGCCGCGCGCGGCCACGTCCGg 93492

ZFN-unknown-SP-6-334
93703 cCGCGCCTCCGGGCCCGGTGACACGa 93728
93703 gGCGCGGAGGCCCGGGCCACTGTGCt 93728

ZFN-unknown-SP-5-319
93772 gCTCATCTTCCCGGCGGTGCGGCCg 93796
93772 cGAGTAGAAGGGCCGCCACGCCGGc 93796

ZFN-unknown-SP-7-334
93791 cGGCCGCCGCCCCCCTTTCGGAGGACg 93817
93791 gCCGGCGGCGGGGGGAAAGCCTCCTGc 93817

ZFN-unknown-SP-7-335
93794 cCGCCGCCCCCCTTTCGGAGGACGCGa 93820
93794 gGCGGCGGGGGGAAAGCCTCCTGCGCt 93820

ZFN-unknown-SP-6-335
93795 cGCCGCCCCCCTTTCGGAGGACGCGa 93820
93795 gCGGCGGGGGGAAAGCCTCCTGCGCt 93820

ZFN-unknown-SP-7-336
94190 gGACGACCGCGTCCGACGGAGAGGCCg 94216
94190 cCTGCTGGCGCAGGCTGCCTCTCCGGc 94216

ZFN-unknown-SP-7-337
94193 cGACCGCGTCCGACGGAGAGGCCGTGg 94219
94193 gCTGGCGCAGGCTGCCTCTCCGGCACc 94219

ZFN-unknown-SP-7-338
94196 cCGCGTCCGACGGAGAGGCCGTGGAGc 94222
94196 gGCGCAGGCTGCCTCTCCGGCACCTCg 94222

ZFN-unknown-SP-7-339
94312 aAGCCCCAGCTCACGAAGGTGGTGAAc 94338
94312 tTCGGGGTCGAGTGCTTCCACCACTTg 94338

ZFN-unknown-SP-6-336
94431 tGCCGCCCGCGAGGAGGGCGCGTCGt 94456
94431 aCGGCGGGCGCTCCTCCCGCGCAGCa 94456

ZFN-unknown-SP-6-337
94548 cACACACCGCACATTCTCGGTGGTCg 94573
94548 gTGTGTGGCGTGTAAGAGCCACCAGc 94573

ZFN-unknown-SP-6-338
94551 aCACCGCACATTCTCGGTGGTCGTCg 94576

94551 tGTGGCGTGTAAGAGCCACCAGCAGc 94576

ZFN-unknown-SP-5-320
94575 cGACGACTGCAGCATGCGGGCGGTc 94599
94575 gCTGCTGACGTCGTACGCCCGCCAg 94599

ZFN-unknown-SP-5-321
94600 cTCCGGCGGCTCCAGGTCGGCGGGg 94624
94600 gAGGCCGCCGAGGTCCAGCCGCCCc 94624

ZFN-unknown-SP-6-339
94600 cTCCGGCGGCTCCAGGTCGGCGGGGg 94625
94600 gAGGCCGCCGAGGTCCAGCCGCCCCc 94625

ZFN-unknown-SP-5-322
94603 cGGCGGCTCCAGGTCGGCGGGGGa 94627
94603 gCCGCCGAGGTCCAGCCGCCCCCt 94627

ZFN-unknown-SP-6-340
94603 cGGCGGCTCCAGGTCGGCGGGGGAc 94628
94603 gCCGCCGAGGTCCAGCCGCCCCCTg 94628

ZFN-unknown-SP-5-323
94677 cACCGGCCCCAACGCGGTGTCGGCg 94701
94677 gTGGCCGGGGTTGCGCCACAGCCGc 94701

ZFN-unknown-SP-5-324
94680 cGGCCCCAACGCGGTGTCGGCGGTg 94704
94680 gCCGGGGTTGCGCCACAGCCGCCAc 94704

ZFN-unknown-SP-6-341
94680 cGGCCCCAACGCGGTGTCGGCGGTGt 94705
94680 gCCGGGGTTGCGCCACAGCCGCCACa 94705

ZFN-unknown-SP-7-340
94817 aCTCCTCCTCCGAGCCGGACGCGGGCg 94843
94817 tGAGGAGGAGGCTCGGCCTGCGCCCGc 94843

ZFN-unknown-SP-6-342
94818 cTCCTCCTCCGAGCCGGACGCGGGCg 94843
94818 gAGGAGGAGGCTCGGCCTGCGCCCGc 94843

ZFN-unknown-SP-7-341
94820 cCTCCTCCGAGCCGGACGCGGGCGACc 94846
94820 gGAGGAGGCTCGGCCTGCGCCCGCTGg 94846

ZFN-unknown-SP-6-343
94842 cGACCGCGGCGCCCCAGAAGAAGAAg 94867
94842 gCTGGCGCCGCGGGGTCTTCTTCTTc 94867

ZFN-unknown-SP-6-344
94845 cCGCGGCGCCCCAGAAGAAGAAGGCc 94870
94845 gGCGCCGCGGGGTCTTCTTCTTCCGg 94870

ZFN-unknown-SP-7-342
94991 cCGCCGCCCCCACGCGAGCCGAGTCGc 95017
94991 gGCGGCGGGGGTGCGCTCGGCTCAGCg 95017

ZFN-unknown-SP-6-345
94992 cGCCGCCCCCACGCGAGCCGAGTCGc 95017
94992 gCGGCGGGGGTGCGCTCGGCTCAGCg 95017

ZFN-unknown-SP-5-325
95176 cCCTGACGGCAACGGGTGGTGGCCg 95200
95176 gGGACTGCCGTTGCCCACCACCGGc 95200

ZFN-unknown-SP-5-326
95179 tGACGGCAACGGGTGGTGGCCGAAc 95203
95179 aCTGCCGTTGCCCACCACCGGCTTg 95203

ZFN-unknown-SP-7-343
95207 tCACCGCGCCCGGGCACGCGGGGTGCg 95233
95207 aGTGGCGCGGGCCCGTGCGCCCCACGc 95233

ZFN-unknown-SP-7-344
95275 cCCCTCCAACCAATATGGCTGTCGTGt 95301
95275 gGGGAGGTTGGTTATACCGACAGCACa 95301

ZFN-unknown-SP-6-346
95395 cAGCTTCACCGAGAGCGCCGTCGGGc 95420
95395 gTCGAAGTGGCTCTCGCGGCAGCCCg 95420

ZFN-unknown-SP-6-347
95444 gGACAGCCCCGGGGTCGCGGGCGGGa 95469
95444 cCTGTCGGGGCCCCAGCGCCCGCCCt 95469

ZFN-unknown-SP-6-348
95477 aCACTGCCTCGGAGGGGATGATGGGg 95502
95477 tGTGACGGAGCCTCCCCTACTACCCc 95502

ZFN-unknown-SP-6-349
95513 cCCCCGCCTCGCCTGCGTGGGTGCCa 95538
95513 gGGGGCGGAGCGGACGCACCCACGGt 95538

ZFN-unknown-SP-6-350
95827 gGGCCGCCTACGCGGCGCTGCTGGTg 95852
95827 cCCGGCGGATGCGCCGCGACGACCAc 95852

ZFN-unknown-SP-5-327
95828 gGCCGCCTACGCGGCGCTGCTGGTg 95852
95828 cCGGCGGATGCGCCGCGACGACCAc 95852

ZFN-unknown-SP-6-351
95903 tGTCGGCACCCTGGGGGTTGTCGTCg 95928
95903 aCAGCCGTGGGACCCCCAACAGCAGc 95928

ZFN-unknown-SP-5-328
95921 tGTCGTCGGCGGGCTGACGATAGGc 95945
95921 aCAGCAGCCGCCCGACTGCTATCCg 95945

ZFN-unknown-SP-6-352
95977 gCGCCGCCGCGGCCCTGACGGCGGCg 96002
95977 cGCGGCGGCGCCGGGACTGCCGCCGc 96002

ZFN-unknown-SP-7-345
95980 cCGCCGCGGCCCTGACGGCGGCGGTGg 96006
95980 gGCGGCGCCGGGACTGCCGCCGCCACc 96006

ZFN-unknown-SP-7-346
95989 cCCTGACGGCGGCGGTGGTGGCGGGCc 96015
95989 gGGACTGCCGCCGCCACCACCGCCCGg 96015

ZFN-unknown-SP-6-353
95989 cCCTGACGGCGGCGGTGGTGGCGGGc 96014
95989 gGGACTGCCGCCGCCACCACCGCCCg 96014

ZFN-unknown-SP-6-354
96059 cCGCCACCGCCGCTTTTGCGTCGTCt 96084
96059 gGCGGTGGCGGCGAAAACGCAGCAGa 96084

ZFN-unknown-SP-5-329

96060 cGCCACCGCCGCTTTTGCGTCGTCt 96084
96060 gCGGTGGCGGCGAAAACGCAGCAGa 96084

ZFN-unknown-SP-6-355
96074 tTGCGTCGTCTCCGCGGTCGAGTCTc 96099
96074 aACGCAGCAGAGGCGCCAGCTCAGAg 96099

ZFN-unknown-SP-7-347
96165 aCGCACCACCAGCGATCTCCGCGGGTc 96191
96165 tGCGTGGTGGTCGCTAGAGGCGCCCAg 96191

ZFN-unknown-SP-6-356
96346 aCGCGGCGGCGGGCCTGACGGAGCTg 96371
96346 tGCGCCGCCGCCCGGACTGCCTCGAc 96371

ZFN-unknown-SP-5-330
96571 gGGCGGCCGCGACCCTGCGGGGCCt 96595
96571 cCCGCCGGCGCTGGGACGCCCCGGa 96595

ZFN-unknown-SP-5-331
96602 cTCCGTCTACGCATTGGGGTTTGCg 96626
96602 gAGGCAGATGCGTAACCCCAAACGc 96626

ZFN-unknown-SP-6-357
96619 gGTTTGCGGCGGGGGTGCTGGTGCGg 96644
96619 cCAAACGCCGCCCCCACGACCACGCc 96644

ZFN-unknown-SP-5-332
96886 aGTTGTCCGCGGGAGGCGGTGGTTt 96910
96886 tCAACAGGCGCCCTCCGCCACCAAa 96910

ZFN-unknown-SP-6-358
96936 gCGCGCCCACCGGTCCTTCGCGGGGg 96961
96936 cGCGCGGGTGGCCAGGAAGCGCCCCc 96961

ZFN-unknown-SP-7-348
97394 gAACGGCGCCTAGCTTAGAGGAGGTGa 97420
97394 cTTGCCGCGGATCGAATCTCCTCCACt 97420

ZFN-unknown-SP-5-333
97401 gCCTAGCTTAGAGGAGGTGATGGTa 97425
97401 cGGATCGAATCTCCTCCACTACCAt 97425

ZFN-unknown-SP-5-334
97525 aGCCCGCGGCTGTACTCGGTCGTCg 97549
97525 tCGGGCGCCGACATGAGCCAGCAGc 97549

ZFN-unknown-SP-7-349
97696 aTCCACCCCCACGCGGTGCTGGAGGGc 97722
97696 tAGGTGGGGGTGCGCCACGACCTCCCg 97722

ZFN-unknown-SP-5-335
97698 cCACCCCCACGCGGTGCTGGAGGGc 97722
97698 gGTGGGGGTGCGCCACGACCTCCCg 97722

ZFN-unknown-SP-7-350
97865 cCGTCTCCACCGTGACCTCCGCGGCCg 97891
97865 gGCAGAGGTGGCACTGGAGGCGCCGGc 97891

ZFN-unknown-SP-6-359
97866 cGTCTCCACCGTGACCTCCGCGGCCg 97891
97866 gCAGAGGTGGCACTGGAGGCGCCGGc 97891

ZFN-unknown-SP-5-336
97877 tGACCTCCGCGGCCGTCGGCGGCCa 97901
97877 aCTGGAGGCGCCGGCAGCCGCCGGt 97901

ZFN-unknown-SP-5-337
97914 cACCTTCACCTGCCAGCTGACGTGg 97938
97914 gTGGAAGTGGACGGTCGACTGCACc 97938

ZFN-unknown-SP-6-360
97979 gCACGGCATCGGTGCTGCCGCGGCCa 98004
97979 cGTGCCGTAGCCACGACGGCGCCGGt 98004

ZFN-unknown-SP-5-338
98091 gGACGACTCCTCGCCGGCGGAGAAg 98115
98091 cCTGCTGAGGAGCGGCCGCCTCTTc 98115

ZFN-unknown-SP-6-361
98093 aCGACTCCTCGCCGGCGGAGAAGGTg 98118
98093 tGCTGAGGAGCGGCCGCCTCTTCCAc 98118

ZFN-unknown-SP-6-362
98096 aCTCCTCGCCGGCGGAGAAGGTGGCc 98121
98096 tGAGGAGCGGCCGCCTCTTCCACCGg 98121

ZFN-unknown-SP-6-363
98691 cCCCCGCGGCCTCCCCGGCGCGGGCt 98716
98691 gGGGGCGCCGGAGGGGCCGCGCCCGa 98716

ZFN-unknown-SP-5-339
99145 aCGTAACGCCCTGGGGTTGCCGTGa 99169
99145 tGCATTGCGGGACCCCAACGGCACt 99169

ZFN-unknown-SP-5-340
99218 cACCCACCGACCAACGACGGCGTTt 99242
99218 gTGGGTGGCTGGTTGCTGCCGCAAa 99242

ZFN-unknown-SP-5-341
99456 gAGCTTCGTCAACAGGGCGCTGAGg 99480
99456 cTCGAAGCAGTTGTCCCGCGACTCc 99480

ZFN-unknown-SP-5-342
99459 cTTCGTCAACAGGGCGCTGAGGGCg 99483
99459 gAAGCAGTTGTCCCGCGACTCCCGc 99483

ZFN-unknown-SP-5-343
99576 gGCCCGCCGAGGGGAGAAGAGGGCa 99600
99576 cCGGGCGGCTCCCCTCTTCTCCCGt 99600

ZFN-unknown-SP-5-344
99671 cGGCCCCGCCGGCAAGACGCCGGCg 99695
99671 gCCGGGGCGGCCGTTCTGCGGCCGc 99695

ZFN-unknown-SP-6-364
99779 aATCTGCGTCCTCGGAGGGGGCGCGg 99804
99779 tTAGACGCAGGAGCCTCCCCCGCGCc 99804

ZFN-unknown-SP-6-365
99782 cTGCGTCCTCGGAGGGGGCGCGGGGg 99807
99782 gACGCAGGAGCCTCCCCCGCGCCCCc 99807

ZFN-unknown-SP-6-366
99827 aGGCGGCGGCGGCGGTGGTGTCGGCg 99852
99827 tCCGCCGCCGCCGCCACCACAGCCGc 99852

ZFN-unknown-SP-7-351
99830 cGGCGGCGGCGGTGGTGTCGGCGGCGg 99856
99830 gCCGCCGCCGCCACCACAGCCGCCGCc 99856

ZFN-unknown-SP-6-367
99830 cGGCGGCGGCGGTGGTGTCGGCGGCg 99855
99830 gCCGCCGCCGCCACCACAGCCGCCGc 99855

ZFN-unknown-SP-5-345
99890 cGGCGTCATCGTCGGGCAGCAGATa 99914
99890 gCCGCAGTAGCAGCCCGTCGTCTAt 99914

ZFN-unknown-SP-5-346
99893 cGTCATCGTCGGGCAGCAGATACGt 99917
99893 gCAGTAGCAGCCCGTCGTCTATGCa 99917

ZFN-unknown-SP-5-347
100013 gCGCCGCGGCTGCCGGCCGCTGCTc 100037
100013 cGCGGCGCCGACGGCCGGCGACGAg 100037

ZFN-unknown-SP-6-368
100023 tGCCGGCCGCTGCTCGGGGGGCGCGg 100048
100023 aCGGCCGGCGACGAGCCCCCCGCGCc 100048

ZFN-unknown-SP-6-369
100026 cGGCCGCTGCTCGGGGGGCGCGGGGt 100051
100026 gCCGGCGACGAGCCCCCCGCGCCCCa 100051

ZFN-unknown-SP-7-352
100133 gGTCGGCCGCGTCGACGTGCGGGGGCt 100159
100133 cCAGCCGGCGCAGCTGCACGCCCCCGa 100159

ZFN-unknown-SP-5-348
100136 cGGCCGCGTCGACGTGCGGGGGCTc 100160
100136 gCCGGCGCAGCTGCACGCCCCCGAg 100160

ZFN-unknown-SP-5-349
100180 cGCCTGCACCTCCGAGGGGCCGCGg 100204
100180 gCGGACGTGGAGGCTCCCCGGCGCc 100204

ZFN-unknown-SP-5-350
100183 cTGCACCTCCGAGGGGCCGCGGGGg 100207
100183 gACGTGGAGGCTCCCCGGCGCCCCc 100207

ZFN-unknown-SP-6-370
100185 gCACCTCCGAGGGGCCGCGGGGTCg 100210
100185 cGTGGAGGCTCCCCGGCGCCCCAGc 100210

ZFN-unknown-SP-7-353
100279 cAGCTCCCGCCGACCGCGCCGCGGCCc 100305
100279 gTCGAGGGCGGCTGGCGCGGCGCCGGg 100305

ZFN-unknown-SP-7-354
100294 gCGCCGCGGCCCCCCGGTGGGCCGGAc 100320
100294 cGCGGCGCCGGGGGGCCACCCGGCCTg 100320

ZFN-unknown-SP-6-371
100604 gAACCTCGACGGTCGAGACGATGGCg 100629
100604 cTTGGAGCTGCCAGCTCTGCTACCGc 100629

ZFN-unknown-SP-6-372
101022 gCGCAACCCCGCCACCGCGGCGGCGt 101047
101022 cGCGTTGGGGCGGTGGCGCCGCCGCa 101047

ZFN-unknown-SP-7-355
101036 cCGCGGCGGCGTACTTGGCCGCGGCGg 101062
101036 gGCGCCGCCGCATGAACCGGCGCCGCc 101062

ZFN-unknown-SP-7-356
101082 gCGCACCAGCAGCTTCGGGAGCAGGGt 101108

101082 cGCGTGGTCGTCGAAGCCCTCGTCCCa 101108

ZFN-unknown-SP-6-373
101259 gAGCGCCTGCTTGGCGGCCGACGCGt 101284
101259 cTCGCGGACGAACCGCCGGCTGCGCa 101284

ZFN-unknown-SP-5-351
101400 aAACGCCGGCTGCAGGTCGTCGGCg 101424
101400 tTTGCGGCCGACGTCCAGCAGCCGc 101424

ZFN-unknown-SP-5-352
101403 cGCCGGCTGCAGGTCGTCGGCGCGc 101427
101403 gCGGCCGACGTCCAGCAGCCGCGCg 101427

ZFN-unknown-SP-5-353
101414 gGTCGTCGGCGCGCTGCCGCAGGAc 101438
101414 cCAGCAGCCGCGCGACGGCGTCCTg 101438

ZFN-unknown-SP-6-374
101435 gGACGCCCACGGCCGCGCTGAGGAGc 101460
101435 cCTGCGGGTGCCGGCGCGACTCCTCg 101460

ZFN-unknown-SP-7-357
101457 gAGCCCCTCCGGGGTGGGGAGCAGACa 101483
101457 cTCGGGGAGGCCCCACCCCTCGTCTGt 101483

ZFN-unknown-SP-5-354
101480 gACACCCGGCGAAGATGCGCCGCTc 101504
101480 cTGTGGGCCGCTTCTACGCGGCGAg 101504

ZFN-unknown-SP-5-355
101618 gGGCCCCGCCGGTCGGAGGTATGCc 101642
101618 cCCGGGGCGGCCAGCCTCCATACGg 101642

ZFN-unknown-SP-6-375
101960 cGTCCGCCCAAAATTTGGCGGGGGTg 101985
101960 gCAGGCGGGTTTTAAACCGCCCCCAc 101985

ZFN-unknown-SP-7-358
101960 cGTCCGCCCAAAATTTGGCGGGGGTGg 101986
101960 gCAGGCGGGTTTTAAACCGCCCCCACc 101986

ZFN-unknown-SP-5-356
102170 cCGCCGCGTCGTCCCTGCGCCGGCg 102194
102170 gGCGGCGCAGCAGGGACGCGGCCGc 102194

ZFN-unknown-SP-5-357
102173 cCGCGTCGTCCCTGCGCCGGCGCGc 102197
102173 gGCGCAGCAGGGACGCGGCCGCGCg 102197

ZFN-unknown-SP-7-359
102298 aGCAGGCGGCGCGCCGCGGTGTCGCCc 102324
102298 tCGTCCGCCGCGCGGCGCCACAGCGGg 102324

ZFN-unknown-SP-6-376
102321 gCCCAGCGCCTCGTGCTCGGCCGCCa 102346
102321 cGGGTCGCGGAGCACGAGCCGGCGGt 102346

ZFN-unknown-SP-6-377
102338 cGGCCGCCACGACCCCGGGGCTGCCc 102363
102338 gCCGGCGGTGCTGGGGCCCCGACGGg 102363

ZFN-unknown-SP-6-378
102395 cCACGTCCGCAAACGCGGGGTGGGCg 102420
102395 gGTGCAGGCGTTTGCGCCCCACCCGc 102420

ZFN-unknown-SP-6-379
102542 tGACGGCGCCGGGGTTGAAGCGGGCg 102567
102542 aCTGCCGCGGCCCCAACTTCGCCCGc 102567

ZFN-unknown-SP-6-380
102689 aGACCGCGGCGTCGGGGCCGGGGTGg 102714
102689 tCTGGCGCCGCAGCCCCGGCCCCACc 102714

ZFN-unknown-SP-6-381
102692 cCGCGGCGTCGGGGCCGGGGTGGGGg 102717
102692 gGCGCCGCAGCCCCGGCCCCACCCCc 102717

ZFN-unknown-SP-5-358
102725 cCCCTCCCGCGCTGAGCAGCGGCAc 102749
102725 gGGGAGGGCGCGACTCGTCGCCGTg 102749

ZFN-unknown-SP-5-359
102752 cGGCGGCCCCCACGGGCCGCAACGc 102776
102752 gCCGCCGGGGGTGCCCGGCGTTGCg 102776

ZFN-unknown-SP-6-382
102829 gAGCCGCGCCCCTCCGTGTGCAGGCg 102854
102829 cTCGGCGCGGGGAGGCACACGTCCGc 102854

ZFN-unknown-SP-7-360
102984 gGCCAGCGCCTCGCCCTGCTGCAGCGt 103010
102984 cCGGTCGCGGAGCGGGACGACGTCGCa 103010

ZFN-unknown-SP-6-383
103259 gGCCGTCATCGTGCGCGACGGCGGCg 103284
103259 cCGGCAGTAGCACGCGCTGCCGCCGc 103284

ZFN-unknown-SP-6-384
103271 gCGCGACGGCGGCGGGGTAGTCGTCc 103296
103271 cGCGCTGCCGCCGCCCCATCAGCAGg 103296

ZFN-unknown-SP-7-361
103499 gCGCCGCCGCCCGGGCCTCGGACGCGc 103525
103499 cGCGGCGGCGGGCCCGGAGCCTGCGCg 103525

ZFN-unknown-SP-5-360
103552 tCCCGGCGACGGCGCGGGGGCGGCt 103576
103552 aGGGCCGCTGCCGCGCCCCCGCCGa 103576

ZFN-unknown-SP-6-385
103552 tCCCGGCGACGGCGCGGGGGCGGCTc 103577
103552 aGGGCCGCTGCCGCGCCCCCGCCGAg 103577

ZFN-unknown-SP-6-386
103595 cCGCGGCCTCGACCTCGCTGTCGTCg 103620
103595 gGCGCCGGAGCTGGAGCGACAGCAGc 103620

ZFN-unknown-SP-6-387
103598 cGGCCTCGACCTCGCTGTCGTCGTCc 103623
103598 gCCGGAGCTGGAGCGACAGCAGCAGg 103623

ZFN-unknown-SP-6-388
103679 cGTCCTCCACGGGCGAGTCGGCGCGa 103704
103679 gCAGGAGGTGCCCGCTCAGCCGCGCt 103704

ZFN-unknown-SP-7-362
103801 cGCCCTCCCCGCAGACGGACGAGGAAa 103827
103801 gCGGGAGGGGCGTCTGCCTGCTCCTTt 103827

ZFN-unknown-SP-7-363

103804 cCTCCCCGCAGACGGACGAGGAAAGGg 103830
103804 gGAGGGGCGTCTGCCTGCTCCTTTCCc 103830

ZFN-unknown-SP-7-364
103929 cGACCGCTCCAACAAGGGTGGGGGGGt 103955
103929 gCTGGCGAGGTTGTTCCCACCCCCCCa 103955

ZFN-unknown-SP-6-389
103929 cGACCGCTCCAACAAGGGTGGGGGGg 103954
103929 gCTGGCGAGGTTGTTCCCACCCCCCc 103954

ZFN-unknown-SP-5-361
103932 cCGCTCCAACAAGGGTGGGGGGTg 103956
103932 gGCGAGGTTGTTCCCACCCCCCAc 103956

ZFN-unknown-SP-6-390
103932 cCGCTCCAACAAGGGTGGGGGGTGg 103957
103932 gGCGAGGTTGTTCCCACCCCCCACc 103957

ZFN-unknown-SP-6-391
104062 aCACGACCGCGGGCTCGTAGGCGCGa 104087
104062 tGTGCTGGCGCCCGAGCATCCGCGCt 104087

ZFN-unknown-SP-5-362
104420 cCCCAGCATCTCCAAGTCGAAGTCg 104444
104420 gGGGTCGTAGAGGTTCAGCTTCAGc 104444

ZFN-unknown-SP-5-363
104423 cAGCATCTCCAAGTCGAAGTCGTCc 104447
104423 gTCGTAGAGGTTCAGCTTCAGCAGg 104447

ZFN-unknown-SP-5-364
104473 cCTCCTCGCCGTCCAGGCGGAGTTc 104497
104473 gGAGGAGCGGCAGGTCCGCCTCAAg 104497

ZFN-unknown-SP-6-392
104494 gTTCGTCTCCCAGGCTGACGTCGGTa 104519
104494 cAAGCAGAGGGTCCGACTGCAGCCAt 104519

ZFN-unknown-SP-6-393
104608 cGTCATCGTCGTCCGGGAGGTCGAGc 104633
104608 gCAGTAGCAGCAGGCCCTCCAGCTCg 104633

ZFN-unknown-SP-6-394
104707 cGCCCTCCGAGGTCGCGACGCTGGAg 104732
104707 gCGGGAGGCTCCAGCGCTGCGACCTc 104732

ZFN-unknown-SP-5-365
105152 cAGCATCTCCCGCAGGTCGCGGTTg 105176
105152 gTCGTAGAGGGCGTCCAGCGCCAAc 105176

ZFN-unknown-SP-5-366
105155 cATCTCCCGCAGGTCGCGGTTGCGg 105179
105155 gTAGAGGGCGTCCAGCGCCAACGCc 105179

ZFN-unknown-SP-7-365
105691 gACCCCCGGCTGGCCTGGGGGGCGGTg 105717
105691 cTGGGGGCCGACCGGACCCCCCGCCAc 105717

ZFN-unknown-SP-5-367
105691 gACCCCCGGCTGGCCTGGGGGGCGg 105715
105691 cTGGGGGCCGACCGGACCCCCCGCc 105715

ZFN-unknown-SP-5-368
105824 gCCCGGCGGCGGAGAGGGGGGGAGg 105848
105824 cGGGCCGCCGCCTCTCCCCCCCTCc 105848

ZFN-unknown-SP-7-366
105824 gCCCGGCGGCGGAGAGGGGGGGAGGGa 105850
105824 cGGGCCGCCGCCTCTCCCCCCCTCCCt 105850

ZFN-unknown-SP-7-367
105881 cCCTCGCGAAGGGAATCGCCGGGGGTg 105907
105881 gGGAGCGCTTCCCTTAGCGGCCCCCAc 105907

ZFN-unknown-SP-5-369
106009 gCCCGTCCCCCGCGCTCGGAAGGGa 106033
106009 cGGGCAGGGGGCGCGAGCCTTCCCt 106033

ZFN-unknown-SP-5-370
106320 gGGCGGCCCCGGGCGGCCGCGGTCg 106344
106320 cCCGCCGGGGCCCGCCGGCGCCAGc 106344

ZFN-unknown-SP-6-395
106330 gGGCGGCCGCGGTCGCGTCGACGTCc 106355
106330 cCCGCCGGCGCCAGCGCAGCTGCAGg 106355

ZFN-unknown-SP-7-368
106342 tCGCGTCGACGTCCTGCGCCGCGTCGg 106368
106342 aGCGCAGCTGCAGGACGCGGCGCAGCc 106368

ZFN-unknown-SP-5-371
106456 gGTCTTCGTCGGTGTGCGGCCGCGa 106480
106456 cCAGAAGCAGCCACACGCCGGCGCt 106480

ZFN-unknown-SP-7-369
106527 aGGCGCCCGACCGCGGCGCAGAAGACg 106553
106527 tCCGCGGGCTGGCGCCGCGTCTTCTGc 106553

ZFN-unknown-SP-6-396
106531 gCCCGACCGCGGCGCAGAAGACGCGc 106556
106531 cGGGCTGGCGCCGCGTCTTCTGCGCg 106556

ZFN-unknown-SP-7-370
106583 cCACGGCGCCGTGGGGCTCGGTGGGGc 106609
106583 gGTGCCGCGGCACCCCGAGCCACCCCg 106609

ZFN-unknown-SP-5-372
106716 gGTCGCCTGCCGCGGGCGGGGTCGg 106740
106716 cCAGCGGACGGCGCCCGCCCCAGCc 106740

ZFN-unknown-SP-7-371
106716 gGTCGCCTGCCGCGGGCGGGGTCGGTc 106742
106716 cCAGCGGACGGCGCCCGCCCCAGCCAg 106742

ZFN-unknown-SP-6-397
107017 gGCCAGCGCCGCGGCGGAAGTCGTCg 107042
107017 cCGGTCGCGGCGCCGCCTTCAGCAGc 107042

ZFN-unknown-SP-5-373
107283 gCGTAACCCACGGGGGCCGCGGGAg 107307
107283 cGCATTGGGTGCCCCCGGCGCCCTc 107307

ZFN-unknown-SP-5-374
107599 gGACACCACACCGGCGCTGTCGCCc 107623
107599 cCTGTGGTGTGGCCGCGACAGCGGg 107623

ZFN-unknown-SP-6-398
107631 gCGCGTCCCCGATAACGCCGCGTGCg 107656
107631 cGCGCAGGGGCTATTGCGGCGCACGc 107656

ZFN-unknown-SP-5-375
107652 gTGCGCCTGCCGCGTTCGGCGGCTc 107676
107652 cACGCGGACGGCGCAAGCCGCCGAg 107676

ZFN-unknown-SP-5-376
107740 tTTCCCCGTCATCGCGGCGATGAGt 107764
107740 aAAGGGGCAGTAGCGCCGCTACTCa 107764

ZFN-unknown-SP-6-399
107800 cAGCACCAACCGGAATGCCGATGGGg 107825
107800 gTCGTGGTTGGCCTTACGGCTACCCc 107825

ZFN-unknown-SP-7-372
108185 tCCCTGCACCGGTTTCCGCAGTTGGCg 108211
108185 aGGGACGTGGCCAAAGGCGTCAACCGc 108211

ZFN-unknown-SP-7-373
108261 tCGCGACCGCCGGGGGGGCGGTGGCCc 108287
108261 aGCGCTGGCGGCCCCCCCGCCACCGGg 108287

ZFN-unknown-SP-6-400
108261 tCGCGACCGCCGGGGGGGCGGTGGCc 108286
108261 aGCGCTGGCGGCCCCCCCGCCACCGg 108286

ZFN-unknown-SP-7-374
108492 gCGCGGCCGACGGACCAGAGGCCTGCt 108518
108492 cGCGCCGGCTGCCTGGTCTCCGGACGa 108518

ZFN-unknown-SP-6-401
108700 cCCCGACAACATCCACGAGGACGGCg 108725
108700 gGGGCTGTTGTAGGTGCTCCTGCCGc 108725

ZFN-unknown-SP-6-402
108703 cGACAACATCCACGAGGACGGCGCAt 108728
108703 gCTGTTGTAGGTGCTCCTGCCGCGTa 108728

ZFN-unknown-SP-6-403
108816 aCGCCCCCCCAAGCAAGCGGGGGGCc 108841
108816 tGCGGGGGGGTTCGTTCGCCCCCCGg 108841

ZFN-unknown-SP-5-377
108817 cGCCCCCCCAAGCAAGCGGGGGGCc 108841
108817 gCGGGGGGGTTCGTTCGCCCCCCGg 108841

ZFN-unknown-SP-6-404
108874 gCCTCGCGTCGGGCCAGATGGGGCCc 108899
108874 cGGAGCGCAGCCCGGTCTACCCCGGg 108899

ZFN-unknown-SP-6-405
109008 cACAAACACACGGGTAGAGTGGAACc 109033
109008 gTGTTTGTGTGCCCATCTCACCTTGg 109033

ZFN-unknown-SP-6-406
109214 gACCGACGCAACCTCCGGGGCTTGTg 109239
109214 cTGGCTGCGTTGGAGGCCCCGAACAc 109239

ZFN-unknown-SP-7-375
109291 tCCCTCCGGCCAGAGCCGAGGTGGAGa 109317
109291 aGGGAGGCCGGTCTCGGCTCCACCTCt 109317

ZFN-unknown-SP-7-376
109399 gCACGTCCGCCGACTTGTCCGCGGCCc 109425
109399 cGTGCAGGCGGCTGAACAGGCGCCGGg 109425

ZFN-unknown-SP-6-407
109495 gCGCCGCAACGGCCCCGTCGATGGTg 109520

109495 cGCGGCGTTGCCGGGGCAGCTACCAc 109520

ZFN-unknown-SP-6-408
109501 cAACGGCCCCGTCGATGGTGGAGCCc 109526
109501 gTTGCCGGGGCAGCTACCACCTCGGg 109526

ZFN-unknown-SP-7-377
109598 gCGCGCCTTCACGAGGCGCCGGGTGTc 109624
109598 cGCGCGGAAGTGCTCCGCGGCCCACAg 109624

ZFN-unknown-SP-6-409
109639 gGGCGTCGTCGAGCGTGATGGGGGCg 109664
109639 cCCGCAGCAGCTCGCACTACCCCCGc 109664

ZFN-unknown-SP-6-410
109703 cCGCGGCTCCGCCTCCGAGGGCGGAg 109728
109703 gGCGCCGAGGCGGAGGCTCCCGCCTc 109728

ZFN-unknown-SP-6-411
109706 cGGCTCCGCCTCCGAGGGCGGAGCCg 109731
109706 gCCGAGGCGGAGGCTCCCGCCTCGGc 109731

ZFN-unknown-SP-7-378
109708 gCTCCGCCTCCGAGGGCGGAGCCGCGg 109734
109708 cGAGGCGGAGGCTCCCGCCTCGGCGCc 109734

ZFN-unknown-SP-6-412
109709 cTCCGCCTCCGAGGGCGGAGCCGCGg 109734
109709 gAGGCGGAGGCTCCCGCCTCGGCGCc 109734

ZFN-unknown-SP-7-379
109711 cCGCCTCCGAGGGCGGAGCCGCGGCCc 109737
109711 gGCGGAGGCTCCCGCCTCGGCGCCGGg 109737

ZFN-unknown-SP-7-380
109849 cCACCCCCTCCGGGTTCGCAGGGGCGa 109875
109849 gGTGGGGGAGGCCCAAGCGTCCCCGCt 109875

ZFN-unknown-SP-6-413
109850 cACCCCCTCCGGGTTCGCAGGGGCGa 109875
109850 gTGGGGGAGGCCCAAGCGTCCCCGCt 109875

ZFN-unknown-SP-6-414
109985 cACAAACTGCCTGCTGGGGGCCGAGc 110010
109985 gTGTTTGACGGACGACCCCCGGCTCg 110010

ZFN-unknown-SP-6-415
110068 gCGCGCCCACCGAACAGGAGAGGTTc 110093
110068 cGCGCGGGTGGCTTGTCCTCTCCAAg 110093

ZFN-unknown-SP-6-416
110208 aCCCGGCCCATGTTTGTGTGCCGCTt 110233
110208 tGGGCCGGGTACAAACACACGGCGAa 110233

ZFN-unknown-SP-6-417
110225 gTGCCGCTTCGAGCGAGCGGACGACg 110250
110225 cACGGCGAAGCTCGCTCGCCTGCTGc 110250

ZFN-unknown-SP-7-381
110384 cAACGCCCCCGCCCGCATCGGCAGCGg 110410
110384 gTTGCGGGGGCGGGCGTAGCCGTCGCc 110410

ZFN-unknown-SP-5-378
110386 aCGCCCCCGCCCGCATCGGCAGCGg 110410
110386 tGCGGGGGCGGGCGTAGCCGTCGCc 110410

ZFN-unknown-SP-7-382
110387 cGCCCCCGCCCGCATCGGCAGCGGCAg 110413
110387 gCGGGGGCGGGCGTAGCCGTCGCCGTc 110413

ZFN-unknown-SP-7-383
110390 cCCCGCCCGCATCGGCAGCGGCAGCAc 110416
110390 gGGGCGGGCGTAGCCGTCGCCGTCGTg 110416

ZFN-unknown-SP-6-418
110798 gTACTCCCGCGGGGCCGCGGCCGCTg 110823
110798 cATGAGGGCGCCCCGGCGCCGGCGAc 110823

ZFN-unknown-SP-7-384
110888 gTTCATCGCCCACGATCGCAGCTGCCt 110914
110888 cAAGTAGCGGGTGCTAGCGTCGACGGa 110914

ZFN-unknown-SP-6-419
111025 gCCCCGCGGCCAGCACGGAGCAGCCc 111050
111025 cGGGGCGCCGGTCGTGCCTCGTCGGg 111050

ZFN-unknown-SP-6-420
111046 aGCCCTCGCCCCTGGGTCGGGAGGCg 111071
111046 tCGGGAGCGGGGACCCAGCCCTCCGc 111071

ZFN-unknown-SP-7-385
111048 cCCTCGCCCCTGGGTCGGGAGGCGGTg 111074
111048 gGGAGCGGGGACCCAGCCCTCCGCCAc 111074

ZFN-unknown-SP-7-386
111217 cGCCCGCCCCCGCGTACTGCGGGGTCg 111243
111217 gCGGGCGGGGGCGCATGACGCCCCAGc 111243

ZFN-unknown-SP-6-421
111218 gCCCGCCCCCGCGTACTGCGGGGTCg 111243
111218 cGGGCGGGGGCGCATGACGCCCCAGc 111243

ZFN-unknown-SP-7-387
111218 gCCCGCCCCCGCGTACTGCGGGGTCGc 111244
111218 cGGGCGGGGGCGCATGACGCCCCAGCg 111244

ZFN-unknown-SP-5-379
111220 cCGCCCCCGCGTACTGCGGGGTCGc 111244
111220 gGCGGGGGCGCATGACGCCCCAGCg 111244

ZFN-unknown-SP-7-388
111220 cCGCCCCCGCGTACTGCGGGGTCGCAg 111246
111220 gGCGGGGGCGCATGACGCCCCAGCGTc 111246

ZFN-unknown-SP-6-422
111247 aCTCGTCCACCAAAATGATGGGACGt 111272
111247 tGAGCAGGTGGTTTTACTACCCTGCa 111272

ZFN-unknown-SP-7-389
111319 tCGCACCAACAACCCCCGGGGACGACg 111345
111319 aGCGTGGTTGTTGGGGGCCCCTGCTGc 111345

ZFN-unknown-SP-7-390
111322 cACCAACAACCCCCGGGGACGACGCGg 111348
111322 gTGGTTGTTGGGGGCCCCTGCTGCGCc 111348

ZFN-unknown-SP-7-391
111325 cAACAACCCCCGGGGACGACGCGGGGg 111351
111325 gTTGTTGGGGGCCCCTGCTGCGCCCCc 111351

ZFN-unknown-SP-6-423

111502 tGTCCCCGACCGGCCAGGCGTTTGCc 111527
111502 aCAGGGGCTGGCCGGTCCGCAAACGg 111527

ZFN-unknown-SP-6-424
111728 cTTCAACGCCGCGCTGGCCGTTACGa 111753
111728 gAAGTTGCGGCGCGACCGGCAATGCt 111753

ZFN-unknown-SP-7-392
111958 gGCCCTCGGCCGGTGGCGACGACGGCg 111984
111958 cCGGGAGCCGGCCACCGCTGCTGCCGc 111984

ZFN-unknown-SP-6-425
112013 tCCCGGCGACGAGGAGTGGGAGGAGg 112038
112013 aGGGCCGCTGCTCCTCACCCTCCTCc 112038

ZFN-unknown-SP-5-380
112013 tCCCGGCGACGAGGAGTGGGAGGAg 112037
112013 aGGGCCGCTGCTCCTCACCCTCCTc 112037

ZFN-unknown-SP-6-426
112061 cCACGGCCCCCTCCCGGACGACGAGg 112086
112061 gGTGCCGGGGGAGGGCCTGCTGCTCc 112086

ZFN-unknown-SP-6-427
112064 cGGCCCCCTCCCGGACGACGAGGCCg 112089
112064 gCCGGGGGAGGGCCTGCTGCTCCGGc 112089

ZFN-unknown-SP-5-381
112065 gGCCCCCTCCCGGACGACGAGGCCg 112089
112065 cCGGGGGAGGGCCTGCTGCTCCGGc 112089

ZFN-unknown-SP-6-428
112274 gGACCGCGACTTCGTGGAGGCCGTAg 112299
112274 cCTGGCGCTGAAGCACCTCCGGCATc 112299

ZFN-unknown-SP-6-429
112277 cCGCGACTTCGTGGAGGCCGTAGGGa 112302
112277 gGCGCTGAAGCACCTCCGGCATCCCt 112302

ZFN-unknown-SP-7-393
112388 cCCCGACGGCTCCGCGTGCGGCCGGTt 112414
112388 gGGGCTGCCGAGGCGCACGCCGGCCAa 112414

ZFN-unknown-SP-7-394
112501 aCGCCCCGCCCATGTTTTCCGCGGCCc 112527
112501 tGCGGGGCGGGTACAAAAGGCGCCGGg 112527

ZFN-unknown-SP-5-382
112503 gCCCCGCCCATGTTTTCCGCGGCCc 112527
112503 cGGGGCGGGTACAAAAGGCGCCGGg 112527

ZFN-unknown-SP-6-430
112513 tGTTTTCCGCGGCCCCGCGGGAGATc 112538
112513 aCAAAAGGCGCCGGGGCGCCCTCTAg 112538

ZFN-unknown-SP-7-395
112538 cCGCGTCCTCCACAGCCTGGGCGGGGa 112564
112538 gGCGCAGGAGGTGTCGGACCCGCCCCt 112564

ZFN-unknown-SP-6-431
112541 cGTCCTCCACAGCCTGGGCGGGGACt 112566
112541 gCAGGAGGTGTCGGACCCGCCCCTGa 112566

ZFN-unknown-SP-5-383
112542 gTCCTCCACAGCCTGGGCGGGGACt 112566
112542 cAGGAGGTGTCGGACCCGCCCCTGa 112566

ZFN-unknown-SP-6-432
112690 gGTTCGCGTCAGAACTGCTGGGGCGa 112715
112690 cCAAGCGCAGTCTTGACGACCCCGCt 112715

ZFN-unknown-SP-5-384
113173 tACCGCCCTCGTGTGGATGAAGATa 113197
113173 aTGGCGGGAGCACACCTACTTCTAt 113197

ZFN-unknown-SP-7-396
113223 cGCCGACCGCCCCCCCGGCGGGGCAt 113249
113223 gCGGCTGGCGGGGGGGGCCGCCCCGTa 113249

ZFN-unknown-SP-6-433
113438 gCCCTTCACCAACGACGATGCATGTt 113463
113438 cGGGAAGTGGTTGCTGCTACGTACAa 113463

ZFN-unknown-SP-7-397
113597 gTTCAACGCCAGACCCTGGTGCAGCTg 113623
113597 cAAGTTGCGGTCTGGGACCACGTCGAc 113623

ZFN-unknown-SP-5-385
113803 gTCCATCATCGCCCTGACGGAGCTg 113827
113803 cAGGTAGTAGCGGGACTGCCTCGAc 113827

ZFN-unknown-SP-5-386
113868 aACCAGCGGCCCCCAGGGGGCGCTc 113892
113868 tTGGTCGCCGGGGGTCCCCCGCGAg 113892

ZFN-unknown-SP-5-387
114048 aCGCCTCTTCCGTTGGAAGAGGCGg 114072
114048 tGCGGAGAAGGCAACCTTCTCCGCc 114072

ZFN-unknown-SP-6-434
114442 aGCCACCGGCCCACTGGGCGCTGTGt 114467
114442 tCGGTGGCCGGGTGACCCGCGACACa 114467

ZFN-unknown-SP-6-435
114633 gTCCGACAGCGAGCTCGAGGAGGACg 114658
114633 cAGGCTGTCGCTCGAGCTCCTCCTGc 114658

ZFN-unknown-SP-6-436
114744 aGACCCCTGCGGAGACGGAGGGGCGg 114769
114744 tCTGGGGACGCCTCTGCCTCCCCGCc 114769

ZFN-unknown-SP-7-398
114797 gTCCCCCGGCCCGCCCCGAGGACGCCg 114823
114797 cAGGGGGCCGGGCGGGGCTCCTGCGGc 114823

ZFN-unknown-SP-7-399
114841 aCGCCTCGCCCGGCAGCGCGGCGGGa 114867
114841 tGCGGAGCGGGCCGTCGCGCCGCCCCt 114867

ZFN-unknown-SP-7-400
114848 gCCCGGCAGCGCGGCGGGGAGCCGACg 114874
114848 cGGGCCGTCGCGCCGCCCCTCGGCTGc 114874

ZFN-unknown-SP-6-437
114884 cCGCGACCACCGGCGTGTGGTCGCGc 114909
114884 gGCGCTGGTGGCCGCACACCAGCGCg 114909

ZFN-unknown-SP-6-438
114988 gCACCGCATCCCCGAGGCGGGCGGCg 115013
114988 cGTGGCGTAGGGGCTCCGCCCGCCGc 115013

ZFN-unknown-SP-7-401
114991 cCGCATCCCCGAGGCGGGCGGCGAGGt 115017
114991 gGCGTAGGGGCTCCGCCCGCCGCTCCa 115017

ZFN-unknown-SP-7-402
115092 aAACGCCCACAACCAAGGGGGTCGCCa 115118
115092 tTTGCGGGTGTTGGTTCCCCAGCGGt 115118

ZFN-unknown-SP-7-403
115111 gGTCGCCACCCCGCGTCGGCGCGGACg 115137
115111 cCAGCGGTGGGGCGCAGCCGCGCCTGc 115137

ZFN-unknown-SP-5-388
115113 tCGCCACCCCGCGTCGGCGCGGACg 115137
115113 aGCGGTGGGGCGCAGCCGCGCCTGc 115137

ZFN-unknown-SP-6-439
115143 cCCCGGCGCCACCCACGGCGAGGCGc 115168
115143 gGGGCCGCGGTGGGTGCCGCTCCGCg 115168

ZFN-unknown-SP-6-440
115148 gCGCCACCCACGGCGAGGCGCGGCGc 115173
115148 cGCGGTGGGTGCCGCTCCGCGCCGCg 115173

ZFN-unknown-SP-5-389
115149 cGCCACCCACGGCGAGGCGCGGCGc 115173
115149 gCGGTGGGTGCCGCTCCGCGCCGCg 115173

ZFN-unknown-SP-7-404
115152 cACCCACGGCGAGGCGCGGCGCGGAGg 115178
115152 gTGGGTGCCGCTCCGCGCCGCGCCTCc 115178

ZFN-unknown-SP-6-441
115226 aGGCCCCCCCTCCGCTGATGGCGCTg 115251
115226 tCCGGGGGGGAGGCGACTACCGCGAc 115251

ZFN-unknown-SP-7-405
115228 gCCCCCCCTCCGCTGATGGCGCTGTCc 115254
115228 cGGGGGGGAGGCGACTACCGCGACAGg 115254

ZFN-unknown-SP-6-442
115229 cCCCCCCTCCGCTGATGGCGCTGTCc 115254
115229 gGGGGGGAGGCGACTACCGCGACAGg 115254

ZFN-unknown-SP-7-406
115253 cCCTGACCCCCCCGCACGCGGACGGCc 115279
115253 gGGACTGGGGGGCGTGCGCCTGCCGg 115279

ZFN-unknown-SP-6-443
115646 tGGCGTCCGCGGATGAGACGCTGGCg 115671
115646 aCCGCAGGCGCCTACTCTGCGACCGc 115671

ZFN-unknown-SP-6-444
115719 cCCTATCATCGGAACGGCGGCCGCCg 115744
115719 gGGATAGTAGCCTTGCCGCCGGCGGc 115744

ZFN-unknown-SP-7-407
115896 cGCCAACCGCGTCGAGCGCGGCGTGTc 115922
115896 gCGGTTGGCGCAGCTCGCGCCGCACAg 115922

ZFN-unknown-SP-6-445
116612 aACCACCCGCCCGTCTTGCGCGAACt 116637
116612 tTGGTGGGCGGGCAGAACGCGCTTGa 116637

ZFN-unknown-SP-5-390
117362 tCGCCTCTGCGTAAGTAGGGAGGCc 117386

117362 aGCGGAGACGCATTCATCCCTCCGg 117386

ZFN-unknown-SP-7-408
117566 aCACCTCGCCGTCACCGGAAGAAGCCg 117592
117566 tGTGGAGCGGCAGTGGCCTTCTTCGGc 117592

ZFN-unknown-SP-7-409
117572 cGCCGTCACCGGAAGAAGCCGGTGAAa 117598
117572 gCGGCAGTGGCCTTCTTCGGCCACTTt 117598

ZFN-unknown-SP-7-410
117614 aCGTCCCTGCAGAGTACGGTGGAGGCg 117640
117614 tGCAGGGACGTCTCATGCCACCTCCGc 117640

ZFN-unknown-SP-7-411
117768 aGCCCCCAACGCCATGGTGGGGATTCg 117794
117768 tCGGGGGTTGCGGTACCACCCCTAAGc 117794

ZFN-unknown-SP-6-446
117872 cGCCGACCACTAGACCGCAGACGTCg 117897
117872 gCGGCTGGTGATCTGGCGTCTGCAGc 117897

ZFN-unknown-SP-5-391
118057 gCGCGCCCCCGCCCGGCCGCCGCGc 118081
118057 cGCGCGGGGGCGGGCCGGCGGCGCg 118081

ZFN-unknown-SP-5-392
118078 gCGCGCCCCCGCCCGGCCGCCGCGc 118102
118078 cGCGCGGGGGCGGGCCGGCGGCGCg 118102

ZFN-unknown-SP-5-393
118099 gCGCGCCCCCGCCCGGCCGCCGCGc 118123
118099 cGCGCGGGGGCGGGCCGGCGGCGCg 118123

ZFN-unknown-SP-5-394
118120 gCGCGCCCCCGCCCGGCCGCCGCGc 118144
118120 cGCGCGGGGGCGGGCCGGCGGCGCg 118144

ZFN-unknown-SP-5-395
118141 gCGCGCCCCCGCCCGGCCGCCGCGc 118165
118141 cGCGCGGGGGCGGGCCGGCGGCGCg 118165

ZFN-unknown-SP-5-396
118162 gCGCGCCCCCGCCCGGCCGCCGCGc 118186
118162 cGCGCGGGGGCGGGCCGGCGGCGCg 118186

ZFN-unknown-SP-5-397
118197 gGCCGCCCGCGTCGCGCCGGCGCCc 118221
118197 cCGGCGGGCGCAGCGCGGCCGCGGg 118221

ZFN-unknown-SP-7-412
118276 cCCCACCGCCCCGCCCGGCAGGGGGGc 118302
118276 gGGGTGGCGGGGCGGGCCGTCCCCCCg 118302

ZFN-unknown-SP-5-398
118278 cCACCGCCCCGCCCGGCAGGGGGGc 118302
118278 gGTGGCGGGGCGGGCCGTCCCCCCg 118302

ZFN-unknown-SP-7-413
118437 gGTCCACACAGGAGCGCGCGGGCGGCa 118463
118437 cCAGGTGTGTCCTCGCGCGCCCGCCGt 118463

ZFN-unknown-SP-7-414
118473 gCGCGGCGGCGGTCGGGGTGGGAGTGg 118499
118473 cGCGCCGCCGCCAGCCCCACCCTCACc 118499

ZFN-unknown-SP-7-415
118533 cTCCCCCCACCCCGACCGCCGCCGCGc 118559
118533 gAGGGGGGTGGGGCTGGCGGCGGCGCg 118559

ZFN-unknown-SP-6-447
118534 tCCCCCCACCCCGACCGCCGCCGCGc 118559
118534 aGGGGGGTGGGGCTGGCGGCGGCGCg 118559

ZFN-unknown-SP-7-416
118891 cCCCCCCCCCGCCGCCCGACGAAGGAg 118917
118891 gGGGGGGGGGCGGCGGGCTGCTTCCTc 118917

ZFN-unknown-SP-5-399
118893 cCCCCCCCGCCGCCCGACGAAGGAg 118917
118893 gGGGGGGCGGCGGGCTGCTTCCTc 118917

ZFN-unknown-SP-7-417
118894 cCCCCCCGCCGCCCGACGAAGGAGACc 118920
118894 gGGGGGGCGGCGGGCTGCTTCCTCTGg 118920

ZFN-unknown-SP-5-400
118896 cCCCCGCCGCCCGACGAAGGAGACc 118920
118896 gGGGGCGGCGGGCTGCTTCCTCTGg 118920

ZFN-unknown-SP-5-401
119111 cCGCAGCCGAGCAGCGCCGCGGGCt 119135
119111 gGCGTCGGCTCGTCGCGGCGCCCGa 119135

ZFN-unknown-SP-6-448
119160 gCCCCGCGCCGGCCGCGGCGGAGAGa 119185
119160 cGGGGCGCGGCCGGCGCCGCCTCTCt 119185

ZFN-unknown-SP-6-449
119163 cCGCGCCGGCCGCGGCGGAGAGAACc 119188
119163 gGCGCGGCCGGCGCCGCCTCTCTTGg 119188

ZFN-unknown-SP-5-402
119328 aACACTCCCACGTTGGGGGGGGGCg 119352
119328 tTGTGAGGGTGCAACCCCCCCCCGc 119352

ZFN-unknown-SP-6-450
119328 aACACTCCCACGTTGGGGGGGGGCGc 119353
119328 tTGTGAGGGTGCAACCCCCCCCCGCg 119353

ZFN-unknown-SP-5-403
119329 aCACTCCCACGTTGGGGGGGGCGc 119353
119329 tGTGAGGGTGCAACCCCCCCCCGCg 119353

ZFN-unknown-SP-7-418
119329 aCACTCCCACGTTGGGGGGGGGCGCGc 119355
119329 tGTGAGGGTGCAACCCCCCCCCCGCGCg 119355

ZFN-unknown-SP-5-404
119419 gAGCCCCGCCCGCGTGTTGCTGTGg 119443
119419 cTCGGGGCGGGCGCACAACGACACc 119443

ZFN-unknown-SP-5-405
119422 cCCCGCCCGCGTGTTGCTGTGGGCa 119446
119422 gGGGCGGGCGCACAACGACACCCGt 119446

ZFN-unknown-SP-7-419
119484 gCGCGGCAGCAACGAACGCAGGGGCCc 119510
119484 cGCGCCGTCGTTGCTTGCGTCCCCGGg 119510

ZFN-unknown-SP-6-451

119715 gGGCCCCCGCACCTCGGCGGCCGCCc 119740
119715 cCCGGGGGCGTGGAGCCGCCGGCGGg 119740

ZFN-unknown-SP-5-406
119716 gGCCCCCGCACCTCGGCGGCCGCCc 119740
119716 cCGGGGGCGTGGAGCCGCCGGCGGg 119740

ZFN-unknown-SP-7-420
119729 cGGCGGCCGCCCCCTCCGGCGCCGCGc 119755
119729 gCCGCCGGCGGGGGAGGCCGCGGCGCg 119755

ZFN-unknown-SP-5-407
119825 gCCCGGCGCCGGGCGGAAGGCGTCc 119849
119825 cGGGCCGCGGCCCGCCTTCCGCAGg 119849

ZFN-unknown-SP-5-408
119871 gGCCCCCGGCGGAGCGCGGGGGCCc 119895
119871 cCGGGGGCCGCCTCGCGCCCCCGGg 119895

ZFN-unknown-SP-7-421
120063 tTCCCCCCCCCCCCCCCGCCGCCGCCg 120089
120063 aAGGGGGGGGGGGGGGGCGGCGGCGGc 120089

ZFN-unknown-SP-6-452
120064 tCCCCCCCCCCCCCCCGCCGCCGCCg 120089
120064 aGGGGGGGGGGGGGGGCGGCGGCGGc 120089

ZFN-unknown-SP-5-409
120065 cCCCCCCCCCCCCCCGCCGCCGCCg 120089
120065 gGGGGGGGGGGGGGGCGGCGGCGGc 120089

ZFN-unknown-SP-7-422
120066 cCCCCCCCCCCCCCGCCGCCGCCGCCc 120092
120066 gGGGGGGGGGGGGGCGGCGGCGGCGGg 120092

ZFN-unknown-SP-6-453
120067 cCCCCCCCCCCCCGCCGCCGCCGCCc 120092
120067 gGGGGGGGGGGGGCGGCGGCGGCGGg 120092

ZFN-unknown-SP-5-410
120068 cCCCCCCCCCCCGCCGCCGCCGCCc 120092
120068 gGGGGGGGGGGGCGGCGGCGGCGGg 120092

ZFN-unknown-SP-7-423
120095 tGCCCGCGTCCCACCGAGACGCCGCGc 120121
120095 aCGGGCGCAGGGTGGCTCTGCGGCGCg 120121

ZFN-unknown-SP-7-424
120214 cGTTCTCGCAGGTAGGTTTAGGGTCGt 120240
120214 gCAAGAGCGTCCATCCAAATCCCAGCa 120240

ZFN-unknown-SP-6-454
120247 gAGCTTCTGCTGAGGCGGCGGGGAGa 120272
120247 cTCGAAGACGACTCCGCCGCCCCTCt 120272

ZFN-unknown-SP-6-455
120546 cCCCGTCGTCGTTCCCGGGGGGGCAg 120571
120546 gGGGCAGCAGCAAGGGCCCCCCCGTc 120571

ZFN-unknown-SP-6-456
120621 cCCCAACAACCCCAAAGGCGCGTGCc 120646
120621 gGGGTTGTTGGGGTTTCCGCGCACGg 120646

ZFN-unknown-SP-7-425
120644 gCCCGGCCACAGCCGTGGGTGTGGCGc 120670
120644 cGGGCCGGTGTCGGCACCCACACCGCg 120670

ZFN-unknown-SP-6-457
120647 cGGCCACAGCCGTGGGTGTGGCGCCc 120672
120647 gCCGGTGTCGGCACCCACACCGCGGg 120672

ZFN-unknown-SP-6-458
120675 cCCCTTCCTCTACCGCGTGGGCGCGg 120700
120675 gGGGAAGGAGATGGCGCACCCGCGCc 120700

ZFN-unknown-SP-6-459
120678 cTTCCTCTACCGCGTGGGCGCGGGCg 120703
120678 gAAGGAGATGGCGCACCCGCGCCCGc 120703

ZFN-unknown-SP-6-460
120681 cCTCTACCGCGTGGGCGCGGGCGGGg 120706
120681 gGAGATGGCGCACCCGCGCCCGCCCc 120706

ZFN-unknown-SP-7-426
120883 gAGCCCCCTCGCCCCGATGGGAGTCTc 120909
120883 cTCGGGGGAGCGGGGCTACCCTCAGAg 120909

ZFN-unknown-SP-7-427
120906 tCTCCCCGCAGCCAGGGTAAGGAGGGg 120932
120906 aGAGGGGCGTCGGTCCCATTCCTCCCc 120932

ZFN-unknown-SP-5-411
120908 tCCCCGCAGCCAGGGTAAGGAGGGg 120932
120908 aGGGGCGTCGGTCCCATTCCTCCCc 120932

ZFN-unknown-SP-7-428
121011 tCCTTTCTCCCAGCCAGGGTGAGGAGg 121037
121011 aGGAAAGAGGGTCGGTCCCACTCCTCc 121037

ZFN-unknown-SP-7-429
121151 aCCCGTCCCCGGGGGCAGAGGGCGTGc 121177
121151 tGGGCAGGGGCCCCCGTCTCCCGCACg 121177

ZFN-unknown-SP-6-461
121489 aACAGGCCTCGGGGGTGGGGGCGGCt 121514
121489 tTGTCCGGAGCCCCCACCCCCGCCGa 121514

ZFN-unknown-SP-7-430
121489 aACAGGCCTCGGGGGTGGGGGCGGCTt 121515
121489 tTGTCCGGAGCCCCCACCCCCGCCGAa 121515

ZFN-unknown-SP-5-412
121557 cCCCGGCCCCCCTGCGCGGGGGAGc 121581
121557 gGGGCCGGGGGGACGCGCCCCCTCg 121581

ZFN-unknown-SP-7-431
121557 cCCCGGCCCCCCTGCGCGGGGGAGCTg 121583
121557 gGGGCCGGGGGGACGCGCCCCCTCGAc 121583

ZFN-unknown-SP-6-462
121805 cTGCCCCCGAGGCGCCTCGGCCGGTg 121830
121805 gACGGGGGCTCCGCGGAGCCGGCCAc 121830

ZFN-unknown-SP-7-432
121930 cTGCTCCCGAGACCACGGGTGGCGCGa 121956
121930 gACGAGGGCTCTGGTGCCCACCGCGCt 121956

ZFN-unknown-SP-7-433
122037 tGTTCCACTCCGACGCGGGGGGCGTCg 122063
122037 aCAAGGTGAGGCTGCGCCCCCCGCAGc 122063

ZFN-unknown-SP-7-434
122038 gTTCCACTCCGACGCGGGGGGCGTCGg 122064
122038 cAAGGTGAGGCTGCGCCCCCCGCAGCc 122064

ZFN-unknown-SP-6-463
122038 gTTCCACTCCGACGCGGGGGGCGTCg 122063
122038 cAAGGTGAGGCTGCGCCCCCCGCAGc 122063

ZFN-unknown-SP-5-413
122111 gTGCGGCGGCTCCACGCGGGGGCCg 122135
122111 cACGCCGCCGAGGTGCGCCCCCGGc 122135

ZFN-unknown-SP-5-414
122114 cGGCGGCTCCACGCGGGGGCCGCGg 122138
122114 gCCGCCGAGGTGCGCCCCCGGCGCc 122138

ZFN-unknown-SP-6-464
122377 cTTCCTCGGCCCCCGCGGCGCAGCAg 122402
122377 gAAGGAGCCGGGGGCGCCGCGTCGTc 122402

ZFN-unknown-SP-6-465
122380 cCTCGGCCCCCGCGGCGCAGCAGCGc 122405
122380 gGAGCCGGGGGCGCCGCGTCGTCGCg 122405

ZFN-unknown-SP-5-415
122389 cCGCGGCGCAGCAGCGCGGGGGCCg 122413
122389 gGCGCCGCGTCGTCGCGCCCCCGGc 122413

ZFN-unknown-SP-6-466
122394 gCGCAGCAGCGCGGGGGCCGAGGGAg 122419
122394 cGCGTCGTCGCGCCCCCGGCTCCCTc 122419

ZFN-unknown-SP-6-467
122428 tCTCTCCCCAGCGCCGGACGCGGACg 122453
122428 aGAGAGGGGTCGCGGCCTGCGCCTGc 122453

ZFN-unknown-SP-7-435
122430 tCTCCCCAGCGCCGGACGCGGACGCGa 122456
122430 aGAGGGGTCGCGGCCTGCGCCTGCGCt 122456

ZFN-unknown-SP-7-436
122433 cCCCAGCGCCGGACGCGGACGCGACGc 122459
122433 gGGGTCGCGGCCTGCGCCTGCGCTGCg 122459

ZFN-unknown-SP-7-437
122460 tCCCACCAGCCCCGCCCGCAGAGGAAg 122486
122460 aGGGTGGTCGGGGCGGGCGTCTCCTTc 122486

ZFN-unknown-SP-7-438
122463 cACCAGCCCCGCCCGCAGAGGAAGAGg 122489
122463 gTGGTCGGGGCGGGCGTCTCCTTCTCc 122489

ZFN-unknown-SP-7-439
122466 cAGCCCCGCCCGCAGAGGAAGAGGCGg 122492
122466 gTCGGGGCGGGCGTCTCCTTCTCCGCc 122492

ZFN-unknown-SP-7-440
122469 cCCCGCCCGCAGAGGAAGAGGCGGAGg 122495
122469 gGGGCGGGCGTCTCCTTCTCCGCCTCc 122495

ZFN-unknown-SP-7-441
122595 cGGCGGCGACCGCGGCCTGGGACGACg 122621
122595 gCCGCCGCTGGCGCCGGACCCTGCTGc 122621

ZFN-unknown-SP-7-442
122598 cGGCGACCGCGGCCTGGGACGACGGAg 122624

122598 gCCGCTGGCGCCGGACCCTGCTGCCTc 122624

ZFN-unknown-SP-7-443
122601 cGACCGCGGCCTGGGACGACGGAGACg 122627
122601 gCTGGCGCCGGACCCTGCTGCCTCTGc 122627

ZFN-unknown-SP-7-444
122637 gCGCGGCGCCCGCGGACGCCGGGGCGa 122663
122637 cGCGCCGCGGGCGCCTGCGGCCCCGCt 122663

ZFN-unknown-SP-7-445
122724 tGGCCCCCACCCCCTGGGGGGCGAGGg 122750
122724 aCCGGGGGTGGGGGACCCCCCGCTCCc 122750

ZFN-unknown-SP-6-468
122725 gGCCCCCACCCCCTGGGGGGCGAGGg 122750
122725 cCGGGGGTGGGGGACCCCCCGCTCCc 122750

ZFN-unknown-SP-6-469
122727 cCCCCACCCCCTGGGGGGCGAGGGGc 122752
122727 gGGGGTGGGGGACCCCCCGCTCCCCg 122752

ZFN-unknown-SP-5-416
122728 cCCCACCCCCTGGGGGGCGAGGGGc 122752
122728 gGGGTGGGGGACCCCCCGCTCCCCg 122752

ZFN-unknown-SP-6-470
122807 gACCCGCGCCTCTTCCGGGGGCGGGc 122832
122807 cTGGGCGCGGAGAAGGCCCCCGCCCg 122832

ZFN-unknown-SP-7-446
122807 gACCCGCGCCTCTTCCGGGGGCGGGCc 122833
122807 cTGGGCGCGGAGAAGGCCCCCGCCCGg 122833

ZFN-unknown-SP-7-447
122835 cCGCCCCCTCCGCGGCGTGGGGGGCGg 122861
122835 gGCGGGGGAGGCGCCGCACCCCCCGCc 122861

ZFN-unknown-SP-6-471
122835 cCGCCCCCTCCGCGGCGTGGGGGGCg 122860
122835 gGCGGGGGAGGCGCCGCACCCCCCGc 122860

ZFN-unknown-SP-5-417
122836 cGCCCCCTCCGCGGCGTGGGGGGCg 122860
122836 gCGGGGGAGGCGCCGCACCCCCCGc 122860

ZFN-unknown-SP-6-472
122836 cGCCCCCTCCGCGGCGTGGGGGGCGg 122861
122836 gCGGGGGAGGCGCCGCACCCCCCGCc 122861

ZFN-unknown-SP-6-473
122838 cCCCCTCCGCGGCGTGGGGGGCGGCa 122863
122838 gGGGGAGGCGCCGCACCCCCCGCCGt 122863

ZFN-unknown-SP-7-448
122838 cCCCCTCCGCGGCGTGGGGGGCGGCAc 122864
122838 gGGGGAGGCGCCGCACCCCCCGCCGTg 122864

ZFN-unknown-SP-6-474
122856 gGGCGGCACCGGGGGTGTTGGTGCCg 122881
122856 cCCGCCGTGGCCCCCACAACCACGGc 122881

ZFN-unknown-SP-7-449
123012 gCGCGTCCACCGGCACGGCGGGCGGCg 123038
123012 cGCGCAGGTGGCCGTGCCGCCCGCCGc 123038

ZFN-unknown-SP-6-475
123061 cGCAGACACCACGGGGGCGGCGGCGg 123086
123061 gCGTCTGTGGTGCCCCCGCCGCCGCc 123086

ZFN-unknown-SP-5-418
123075 gGGCGGCGGCGGCGCGGGGCGGACt 123099
123075 cCCGCCGCCGCCGCGCCCCGCCTGa 123099

ZFN-unknown-SP-5-419
123111 gGGCGACGGCCGCGCGGGGGCGCGc 123135
123111 cCCGCTGCCGGCGCGCCCCCGCGCg 123135

ZFN-unknown-SP-5-420
123362 gCGCGCCCCCCGCCGGCCGCAGGGg 123386
123362 cGCGCGGGGGGCGGCCGGCGTCCCc 123386

ZFN-unknown-SP-5-421
123365 cGCCCCCCGCCGGCCGCAGGGGGAa 123389
123365 gCGGGGGGCGGCCGGCGTCCCCCTt 123389

ZFN-unknown-SP-5-422
123369 cCCCGCCGGCCGCAGGGGGAAGAGa 123393
123369 gGGGCGGCCGGCGTCCCCCTTCTCt 123393

ZFN-unknown-SP-5-423
123451 cCCCGCCCGCAACGGGGCGCCGCCg 123475
123451 gGGGCGGGCGTTGCCCCGCGGCGGc 123475

ZFN-unknown-SP-5-424
123454 cGCCCGCAACGGGGCGCCGCCGCTg 123478
123454 gCGGGCGTTGCCCCGCGGCGGCGAc 123478

ZFN-unknown-SP-6-476
123470 cCGCCGCTGCTGCTGCTCCGCGGGGc 123495
123470 gGCGGCGACGACGACGAGGCGCCCCg 123495

ZFN-unknown-SP-6-477
123551 gCACCGCCGCCCCCGCGCCGGGGCGc 123576
123551 cGTGGCGGCGGGGGCGCGGCCCCGCg 123576

ZFN-unknown-SP-7-450
124213 cACCCCCACAGGATCCCTGCGCGTCGg 124239
124213 gTGGGGGTGTCCTAGGGACGCGCAGCc 124239

ZFN-unknown-SP-6-478
124231 gCGCGTCGGCGGGCGTGGGGCTGCCc 124256
124231 cGCGCAGCCGCCCGCACCCCGACGGg 124256

ZFN-unknown-SP-6-479
124333 tGCCCGCCTCGGAGGTGGAGTCGCGg 124358
124333 aCGGGCGGAGCCTCCACCTCAGCGCc 124358

ZFN-unknown-SP-5-425
124388 gGTCTCCTCCTCCGAGTCGCTGCTg 124412
124388 cCAGAGGAGGAGGCTCAGCGACGAc 124412

ZFN-unknown-SP-6-480
124390 tCTCCTCCTCCGAGTCGCTGCTGGCg 124415
124390 aGAGGAGGAGGCTCAGCGACGACCGc 124415

ZFN-unknown-SP-5-426
124391 cTCCTCCTCCGAGTCGCTGCTGGCg 124415
124391 gAGGAGGAGGCTCAGCGACGACCGc 124415

ZFN-unknown-SP-6-481

```
124433 gAGCATCCCCCAGGCGTGCGGGGCGg 124458
124433 cTCGTAGGGGGTCCGCACGCCCCGCc 124458

ZFN-unknown-SP-5-427
124434 aGCATCCCCCAGGCGTGCGGGGCGg 124458
124434 tCGTAGGGGGTCCGCACGCCCCGCc 124458

ZFN-unknown-SP-7-451
124560 cGCCCGCTGCTTTTTGTTCGGAAGGGg 124586
124560 gCGGGCGACGAAAAACAAGCCTTCCCc 124586

ZFN-unknown-SP-6-482
124687 tCGCCCCCCCAGAGGGTCGGGGGGCg 124712
124687 aGCGGGGGGGTCTCCCAGCCCCCCGc 124712

ZFN-unknown-SP-5-428
124687 tCGCCCCCCCAGAGGGTCGGGGGGc 124711
124687 aGCGGGGGGGTCTCCCAGCCCCCCg 124711

ZFN-unknown-SP-5-429
124688 cGCCCCCCCAGAGGGTCGGGGGGCg 124712
124688 gCGGGGGGGTCTCCCAGCCCCCCGc 124712

ZFN-unknown-SP-6-483
124800 cGACCACCCCCAACCCGCAGCCGGGt 124825
124800 gCTGGTGGGGGTTGGGCGTCGGCCCa 124825

ZFN-unknown-SP-5-430
124801 gACCACCCCCAACCCGCAGCCGGGt 124825
124801 cTGGTGGGGGTTGGGCGTCGGCCCa 124825

ZFN-unknown-SP-7-452
124859 gGTCGCCCTCTCACCGTGCCGGGGGTc 124885
124859 cCAGCGGGAGAGTGGCACGGCCCCCAg 124885

ZFN-unknown-SP-5-431
124888 cCGCGGCGGCCGCTCGGGGCCGGGg 124912
124888 gGCGCCGCCGGCGAGCCCCGGCCCc 124912

ZFN-unknown-SP-5-432
124891 cGGCGGCCGCTCGGGGCCGGGGTCc 124915
124891 gCCGCCGGCGAGCCCCGGCCCCAGg 124915

ZFN-unknown-SP-7-453
125024 tCTCCTCCTCCCGCCGGGCCGCCGCTc 125050
125024 aGAGGAGGAGGGCGGCCCGGCGGCGAg 125050

ZFN-unknown-SP-6-484
125025 cTCCTCCTCCCGCCGGGCCGCCGCTc 125050
125025 gAGGAGGAGGGCGGCCCGGCGGCGAg 125050

ZFN-unknown-SP-7-454
125167 gCGCCCCGGCGGGGGCGGAGGGAGGGa 125193
125167 cGCGGGGCCGCCCCCGCCTCCCTCCCt 125193

ZFN-unknown-SP-6-485
125191 gGAATCCCCCCCTCTCGGGGCGGCCc 125216
125191 cCTTAGGGGGGGAGAGCCCCGCCGGg 125216

ZFN-unknown-SP-7-455
125258 aGCATGCGCCGCCCCCCGCCGACGCAg 125284
125258 tCGTACGCGGCGGGGGGCGGCTGCGTc 125284

ZFN-unknown-SP-5-433
125263 gCGCCGCCCCCCGCCGACGCAGATg 125287
125263 cGCGGCGGGGGGCGGCTGCGTCTAc 125287
```

ZFN-unknown-SP-7-456
125264 cGCCGCCCCCCGCCGACGCAGATGGGa 125290
125264 gCGGCGGGGGCGGCTGCGTCTACCCt 125290

ZFN-unknown-SP-5-434
125292 tCCCCCCGGCGCCCCGCCGGCGCGg 125316
125292 aGGGGGGCCGCGGGGCGGCCGCGCc 125316

ZFN-unknown-SP-5-435
125295 cCCCGGCGCCCCGCCGGCGCGGCCc 125319
125295 gGGGCCGCGGGGCGGCCGCGCCGGg 125319

ZFN-unknown-SP-6-486
125712 gGCCGGCGCCGCCCCCTGGGCGGGc 125737
125712 cCGGCCGCGGCGGGGACCCCGCCCg 125737

ZFN-unknown-SP-7-457
125712 gGCCGGCGCCGCCCCCTGGGGCGGGCg 125738
125712 cCGGCCGCGGCGGGGACCCCGCCCGc 125738

ZFN-unknown-SP-5-436
125717 gCGCCGCCCCCTGGGGCGGGCGGAg 125741
125717 cGCGGCGGGGGACCCCGCCCGCCTc 125741

ZFN-unknown-SP-7-458
125718 cGCCGCCCCCTGGGGCGGGCGGAGCGg 125744
125718 gCGGCGGGGGACCCCGCCCGCCTCGCc 125744

ZFN-unknown-SP-7-459
125924 cCGCCGCCTCCTCCTCCTCCGCGGGTc 125950
125924 gGCGGCGGAGGAGGAGGAGGCGCCCAg 125950

ZFN-unknown-SP-6-487
125925 cGCCGCCTCCTCCTCCTCCGCGGGTc 125950
125925 gCGGCGGAGGAGGAGGAGGCGCCCAg 125950

ZFN-unknown-SP-5-437
126029 cGGCCGCCGCCACGCGGCGCCGGAa 126053
126029 gCCGGCGGCGGTGCGCCGCGGCCTt 126053

ZFN-unknown-SP-7-460
126040 aCGCGGCGCCGGAACCGGTCGCGGTCg 126066
126040 tGCGCCGCGGCCTTGGCCAGCGCCAGc 126066

ZFN-unknown-SP-6-488
126156 gCACACCTGCGGGCGGGGAGACACGg 126181
126156 cGTGTGGACGCCCGCCCCTCTGTGCc 126181

ZFN-unknown-SP-7-461
126327 gCGCGTCCGCGGGCGGGGACGCGGGGg 126353
126327 cGCGCAGGCGCCCGCCCCTGCGCCCCc 126353

ZFN-unknown-SP-7-462
126385 gTACTCCGTCGTGGTGCGCAGCCGTAg 126411
126385 cATGAGGCAGCACCACGCGTCGGCATc 126411

ZFN-unknown-SP-5-438
126454 gCGCGGCGGCGGCGGGGGGCAGGCg 126478
126454 cGCGCCGCCGCCGCCCCCCGTCCGc 126478

ZFN-unknown-SP-5-439
126457 cGGCGGCGGCGGGGGGCAGGCGGCg 126481
126457 gCCGCCGCCGCCCCCCGTCCGCCGc 126481

ZFN-unknown-SP-7-463
126471 gGCAGGCGGCGGCAGGCGCGGCGTGCg 126497
126471 cCGTCCGCCGCCGTCCGCGCCGCACGc 126497

ZFN-unknown-SP-6-489
126512 tTCCCCCCGCCCTCGCTCGGGGGGCt 126537
126512 aAGGGGGGCGGGAGCGAGCCCCCCGa 126537

ZFN-unknown-SP-5-440
126513 tCCCCCCGCCCTCGCTCGGGGGGCt 126537
126513 aGGGGGGCGGGAGCGAGCCCCCCGa 126537

ZFN-unknown-SP-6-490
126516 cCCCGCCCTCGCTCGGGGGGCTGTTc 126541
126516 gGGGCGGGAGCGAGCCCCCCGACAAg 126541

ZFN-unknown-SP-6-491
126546 aCTCTGCGTCGTCGTTGCCGGCGTAg 126571
126546 tGAGACGCAGCAGCAACGGCCGCATc 126571

ZFN-unknown-SP-6-492
126549 cTGCGTCGTCGTTGCCGGCGTAGTCc 126574
126549 gACGCAGCAGCAACGGCCGCATCAGg 126574

ZFN-unknown-SP-7-464
126598 gGGCACCAGCAGCCAGCGCCGCAGGAg 126624
126598 cCCGTGGTCGTCGGTCGCGGCGTCCTc 126624

ZFN-unknown-SP-7-465
126601 cACCAGCAGCCAGCGCCGCAGGAGCGa 126627
126601 gTGGTCGTCGGTCGCGGCGTCCTCGCt 126627

ZFN-unknown-SP-7-466
126962 gGCCCTCTTAAGGGGCGGCGGCAGGAc 126988
126962 cCGGGAGAATTCCCCGCCGCCGTCCTg 126988

ZFN-unknown-SP-5-441
127067 gGGCGGCGGCGGCGGGCGGGCGGCa 127091
127067 cCCGCCGCCGCCGCCCGCCCGCCGt 127091

ZFN-unknown-SP-6-493
127070 cGGCGGCGGCGGGCGGGCGGCAGGGc 127095
127070 gCCGCCGCCGCCCGCCCGCCGTCCCg 127095

ZFN-unknown-SP-6-494
127131 aGCCGGCCGCTCCCCCGCGGGCGCCg 127156
127131 tCGGCCGGCGAGGGGGCGCCCGCGGc 127156

ZFN-unknown-SP-6-495
127134 cGGCCGCTCCCCCGCGGGCGCCGCCc 127159
127134 gCCGGCGAGGGGGCGCCCGCGGCGGg 127159

ZFN-unknown-SP-6-496
127156 gCCCCTCCCCCCGCGCGCCGCGGGGc 127181
127156 cGGGGAGGGGGGCGCGCGGCGCCCCg 127181

ZFN-unknown-SP-5-442
127157 cCCCTCCCCCCGCGCGCCGCGGGGc 127181
127157 gGGGAGGGGGGCGCGCGGCGCCCCg 127181

ZFN-unknown-SP-7-467
127160 cTCCCCCCGCGCGCCGCGGGGCTGCCt 127186
127160 gAGGGGGGCGCGCGGCGCCCCGACGGa 127186

ZFN-unknown-SP-6-497
127217 cCGCGCCCGCCCCCGCGCGGCAGGAc 127242

127217 gGCGCGGGCGGGGGCGCGCCGTCCTg 127242

ZFN-unknown-SP-7-468
127223 cCGCCCCCGCGCGGCAGGACGGGGACt 127249
127223 gGCGGGGGCGCGCCGTCCTGCCCCTGa 127249

ZFN-unknown-SP-5-443
127285 gCTCCCCGCCCCCCCGAAGACGCCa 127309
127285 cGAGGGGCGGGGGGGCTTCTGCGGt 127309

ZFN-unknown-SP-7-469
127427 tCCCCCCCGCGCGCCACGGGGCTGCGg 127453
127427 aGGGGGGGCGCGCGGTGCCCCGACGCc 127453

ZFN-unknown-SP-7-470
127456 cCGCGGCCGCCTCCCCCGCGGCCGCCt 127482
127456 gGCGCCGGCGGAGGGGGCGCCGGCGGa 127482

ZFN-unknown-SP-6-498
127529 cCGCGGCCCCGAGCTCGCAGCAGCCc 127554
127529 gGCGCCGGGGCTCGAGCGTCGTCGGg 127554

ZFN-unknown-SP-7-471
127573 gCCTTCCCTCCCGCTCCTGCGGGGGGg 127599
127573 cGGAAGGGAGGGCGAGGACGCCCCCCc 127599

ZFN-unknown-SP-6-499
127577 tCCCTCCCGCTCCTGCGGGGGGCTc 127602
127577 aGGGAGGGCGAGGACGCCCCCCGAg 127602

ZFN-unknown-SP-7-472
127646 gACCCCCCGCGGGCGCGGGAGACGTGc 127672
127646 cTGGGGGGCGCCCGCGCCCTCTGCACg 127672

ZFN-unknown-SP-6-500
128168 tCCCCACCACCTCCACGCCGCCGCCc 128193
128168 aGGGGTGGTGGAGGTGCGGCGGCGGg 128193

ZFN-unknown-SP-5-444
128169 cCCCACCACCTCCACGCCGCCGCCc 128193
128169 gGGGTGGTGGAGGTGCGGCGGCGGg 128193

ZFN-unknown-SP-7-473
128207 cCGTCCCCGCGCGGCCCGCGGCCGACg 128233
128207 gGCAGGGGCGCGCCGGGCGCCGGCTGc 128233

ZFN-unknown-SP-7-474
128228 cCGACGCCCAGCGTATCTGCGGGGGCg 128254
128228 gGCTGCGGGTCGCATAGACGCCCCCGc 128254

ZFN-unknown-SP-6-501
128269 tCGTCGCGCAGCACCAGCGGGGGCGc 128294
128269 aGCAGCGCGTCGTGGTCGCCCCCGCg 128294

ZFN-unknown-SP-6-502
128274 gCGCAGCACCAGCGGGGGCGCGTCGc 128299
128274 cGCGTCGTGGTCGCCCCCGCGCAGCg 128299

ZFN-unknown-SP-7-475
128377 tCGCGCCCCAGCGCCACGTAGACGGGc 128403
128377 aGCGCGGGGTCGCGGTGCATCTGCCCg 128403

ZFN-unknown-SP-5-445
128379 gCGCCCCAGCGCCACGTAGACGGGc 128403
128379 cGCGGGGTCGCGGTGCATCTGCCCg 128403

ZFN-unknown-SP-7-476
128419 aGGCCCCAGCGCGCGCAGGCGCGGTGc 128445
128419 tCCGGGGTCGCGCGCGTCCGCGCCACg 128445

ZFN-unknown-SP-7-477
128492 cGTCGCCCGCGCCCGAGGCGGCGGCCc 128518
128492 gCAGCGGGCGCGGGCTCCGCCGCCGGg 128518

ZFN-unknown-SP-5-446
128568 gGGCACCAGCGTGTCGGGGCCGAAg 128592
128568 cCCGTGGTCGCACAGCCCCGGCTTc 128592

ZFN-unknown-SP-6-503
128620 gCCCCGCGGCAGAGGCGCAGCGGCGg 128645
128620 cGGGGCGCCGTCTCCGCGTCGCCGCc 128645

ZFN-unknown-SP-5-447
128634 gCGCAGCGGCGGCGCGTCGGGGTAc 128658
128634 cGCGTCGCCGCCGCGCAGCCCCATg 128658

ZFN-unknown-SP-6-504
128783 gCACCTCGCAGGCCAGGTAGGCGTGc 128808
128783 cGTGGAGCGTCCGGTCCATCCGCACg 128808

ZFN-unknown-SP-7-478
128783 gCACCTCGCAGGCCAGGTAGGCGTGCt 128809
128783 cGTGGAGCGTCCGGTCCATCCGCACGa 128809

ZFN-unknown-SP-7-479
128825 gCCCGTCGGCGGGCCAGTCCGCGGCGc 128851
128825 cGGGCAGCCGCCCGGTCAGGCGCCGCg 128851

ZFN-unknown-SP-6-505
128840 aGTCCGCGGCGCGCACGGCGTTGACg 128865
128840 tCAGGCGCCGCGCGTGCCGCAACTGc 128865

ZFN-unknown-SP-5-448
128967 gCCCAGCGCCGAGACGTCGGGGGCg 128991
128967 cGGGTCGCGGCTCTGCAGCCCCCGc 128991

ZFN-unknown-SP-6-506
128967 gCCCAGCGCCGAGACGTCGGGGGCGc 128992
128967 cGGGTCGCGGCTCTGCAGCCCCCGCg 128992

ZFN-unknown-SP-7-480
129002 tGCCCGCCCAGGCGGCCGTGGCGGGCc 129028
129002 aCGGGCGGGTCCGCCGGCACCGCCCGg 129028

ZFN-unknown-SP-5-449
129200 aGGCCGCCGCGCGGCGCAGCGGGCc 129224
129200 tCCGGCGGCGCGCCGCGTCGCCCGg 129224

ZFN-unknown-SP-6-507
129368 cGGCCACGGCCCGCGGGCGCAGTAg 129393
129368 gCCGGTGCCGGGCGCCCCGCGTCATc 129393

ZFN-unknown-SP-7-481
129401 gGGCGGCGGCCGAGGGCGCCGGCGTGt 129427
129401 cCCGCCGCCGGCTCCCGCGGCCGCACa 129427

ZFN-unknown-SP-7-482
129451 cGCCAGCCGCCCTGCGGGTCGGGGCCc 129477
129451 gCGGTCGGCGGGACGCCCAGCCCCGGg 129477

ZFN-unknown-SP-7-483


```
129492 gGTCAGCGCCGCGGGGCGCGGCGGCCg 129518
129492 cCAGTCGCGGCGCCCCGCGCCGCCGGc 129518
```

```
ZFN-unknown-SP-7-484
129507 gCGCGGCGGCCGCGGCGGCGGCGTCGg 129533
129507 cGCGCCGCCGGCGCCGCCGCCGCAGCc 129533
```

```
ZFN-unknown-SP-7-485
129513 cGGCCGCGGCGGCGGCGTCGGCGGGGc 129539
129513 gCCGGCGCCGCCGCCGCAGCCGCCCCg 129539
```

```
ZFN-unknown-SP-6-508
129513 cGGCCGCGGCGGCGGCGTCGGCGGGg 129538
129513 gCCGGCGCCGCCGCCGCAGCCGCCCc 129538
```

```
ZFN-unknown-SP-6-509
129516 cCGCGGCGGCGGCGTCGGCGGGGCGg 129541
129516 gGCGCCGCCGCCGCAGCCGCCCCGCc 129541
```

```
ZFN-unknown-SP-6-510
129519 cGGCGGCGGCGTCGGCGGGGCGGGGg 129544
129519 gCCGCCGCCGCAGCCGCCCCGCCCCc 129544
```

```
ZFN-unknown-SP-6-511
129522 cGGCGGCGTCGGCGGGGCGGGGGGCg 129547
129522 gCCGCCGCAGCCGCCCCGCCCCCCGc 129547
```

```
ZFN-unknown-SP-5-450
129522 cGGCGGCGTCGGCGGGGCGGGGGGc 129546
129522 gCCGCCGCAGCCGCCCCGCCCCCCg 129546
```

```
ZFN-unknown-SP-6-512
129525 cGGCGTCGGCGGGGCGGGGGGCGCGg 129550
129525 gCCGCAGCCGCCCCGCCCCCCGCGCc 129550
```

```
ZFN-unknown-SP-7-486
129545 gCGCGGCCCCGCGGGAGGGGCGGCCg 129571
129545 cGCGCCGGGGCGCCCTCCCCGCCGGc 129571
```

```
ZFN-unknown-SP-7-487
129548 cGGCCCCGCGGGAGGGGCGGCCGCGg 129574
129548 gCCGGGGCGCCCTCCCCGCCGGCGCc 129574
```

```
ZFN-unknown-SP-6-513
129563 gGGCGGCCGCGGGGCGGGGGCGTCc 129588
129563 cCCGCCGGCGCCCCGCCCCCGCAGg 129588
```

```
ZFN-unknown-SP-6-514
129621 gCCCGGCGGCGCCCTGGCCGGGGCGg 129646
129621 cGGGCCGCCGCGGGACCGGCCCCGCc 129646
```

```
ZFN-unknown-SP-6-515
129624 cGGCGGCGCCCTGGCCGGGGCGGGGc 129649
129624 gCCGCCGCGGGACCGGCCCCGCCCCg 129649
```

```
ZFN-unknown-SP-6-516
129660 gCGCGCCTCCCGCGGCGCGGAGGCGg 129685
129660 cGCGCGGAGGGCGCCGCGCCTCCGCc 129685
```

```
ZFN-unknown-SP-6-517
129663 cGCCTCCCGCGGCGCGGAGGCGGGCg 129688
129663 gCGGAGGGCGCCGCGCCTCCGCCCGc 129688
```

```
ZFN-unknown-SP-5-451
129663 cGCCTCCCGCGGCGCGGAGGCGGGc 129687
129663 gCGGAGGGCGCCGCGCCTCCGCCCg 129687
```


ZFN-unknown-SP-6-518
129666 cTCCCGCGGCGCGGAGGCGGGCGCGg 129691
129666 gAGGGCGCCGCGCCTCCGCCCGCGCc 129691

ZFN-unknown-SP-6-519
129698 aGTCGGCCGCGGCGACGGTGTCGGCc 129723
129698 tCAGCCGGCGCCGCTGCCACAGCCGg 129723

ZFN-unknown-SP-7-488
129758 gGTCCGCGGCGGCGGCGGCGGCGGAGc 129784
129758 cCAGGCGCCGCCGCCGCCGCCGCCTCg 129784

ZFN-unknown-SP-6-520
129761 cCGCGGCGGCGGCGGCGGCGGAGCTc 129786
129761 gGCGCCGCCGCCGCCGCCGCCTCGAg 129786

ZFN-unknown-SP-6-521
129854 cGGCCACGGCGGCCTCGCTGCCGCCg 129879
129854 gCCGGTGCCGCCGGAGCGACGGCGGc 129879

ZFN-unknown-SP-6-522
129857 cCACGGCGGCCTCGCTGCCGCCGGCc 129882
129857 gGTGCCGCCGGAGCGACGGCGGCCGg 129882

ZFN-unknown-SP-7-489
129965 gCGCGTCGGCGTGCGGCGGGGCGGCCg 129991
129965 cGCGCAGCCGCACGCCGCCCCGCCGGc 129991

ZFN-unknown-SP-6-523
130046 gCACGGCCGCCAGGTCGCCGTCGAAg 130071
130046 cGTGCCGGCGGTCCAGCGGCAGCTTc 130071

ZFN-unknown-SP-6-524
130133 cCGCCTCCGCGCGCCGGCCGCCGCCa 130158
130133 gGCGGAGGCGCGCGGCCGGCGGCGGt 130158

ZFN-unknown-SP-7-490
130147 cGGCCGCCGCCACCACCGCCGGCGCCg 130173
130147 gCCGGCGGCGGTGGTGGCGGCCGCGGc 130173

ZFN-unknown-SP-7-491
130150 cCGCCGCCACCACCGCCGGCGCCGTCg 130176
130150 gGCGGCGGTGGTGGCGGCCGCGGCAGc 130176

ZFN-unknown-SP-6-525
130151 cGCCGCCACCACCGCCGGCGCCGTCg 130176
130151 gCGGCGGTGGTGGCGGCCGCGGCAGc 130176

ZFN-unknown-SP-7-492
130153 cCGCCACCACCGCCGGCGCCGTCGTCg 130179
130153 gGCGGTGGTGGCGGCCGCGGCAGCAGc 130179

ZFN-unknown-SP-6-526
130154 cGCCACCACCGCCGGCGCCGTCGTCg 130179
130154 gCGGTGGTGGCGGCCGCGGCAGCAGc 130179

ZFN-unknown-SP-7-493
130156 cCACCACCGCCGGCGCCGTCGTCGTCg 130182
130156 gGTGGTGGCGGCCGCGGCAGCAGCAGc 130182

ZFN-unknown-SP-6-527
130157 cACCACCGCCGGCGCCGTCGTCGTCg 130182
130157 gTGGTGGCGGCCGCGGCAGCAGCAGc 130182

ZFN-unknown-SP-6-528
130160 cACCGCCGGCGCCGTCGTCGTCGTCg 130185
130160 gTGGCGGCCGCGGCAGCAGCAGCAGc 130185

ZFN-unknown-SP-6-529
130163 cGCCGGCGCCGTCGTCGTCGTCGTCg 130188
130163 gCGGCCGCGGCAGCAGCAGCAGCAGc 130188

ZFN-unknown-SP-6-530
130166 cGGCGCCGTCGTCGTCGTCGTCGTCg 130191
130166 gCCGCGGCAGCAGCAGCAGCAGCAGc 130191

ZFN-unknown-SP-6-531
130169 cGCCGTCGTCGTCGTCGTCGTCGTCg 130194
130169 gCGGCAGCAGCAGCAGCAGCAGCAGc 130194

ZFN-unknown-SP-6-532
130172 cGTCGTCGTCGTCGTCGTCGTCGGCc 130197
130172 gCAGCAGCAGCAGCAGCAGCAGCCGg 130197

ZFN-unknown-SP-7-494
130184 cGTCGTCGTCGGCCCCGGCCGGCGCGg 130210
130184 gCAGCAGCAGCCGGGGCCGGCCGCGCc 130210

ZFN-unknown-SP-7-495
130187 cGTCGTCGGCCCCGGCCGGCGCGGAGg 130213
130187 gCAGCAGCCGGGGCCGGCCGCGCCTCc 130213

ZFN-unknown-SP-7-496
130190 cGTCGGCCCCGGCCGGCGCGGAGGCGg 130216
130190 gCAGCCGGGGCCGGCCGCGCCTCCGCc 130216

ZFN-unknown-SP-7-497
130193 cGGCCCCGGCCGGCGCGGAGGCGGGCg 130219
130193 gCCGGGGCCGGCCGCGCCTCCGCCCGc 130219

ZFN-unknown-SP-6-533
130193 cGGCCCCGGCCGGCGCGGAGGCGGGc 130218
130193 gCCGGGGCCGGCCGCGCCTCCGCCCg 130218

ZFN-unknown-SP-5-452
130245 gAGCACCCCCGCGGCGCCGTAGCCg 130269
130245 cTCGTGGGGGCGCCGCGGCATCGGc 130269

ZFN-unknown-SP-6-534
130247 gCACCCCCGCGGCGCCGTAGCCGGCg 130272
130247 cGTGGGGGCGCCGCGGCATCGGCCGc 130272

ZFN-unknown-SP-6-535
130250 cCCCCGCGGCGCCGTAGCCGGCGGGc 130275
130250 gGGGGCGCCGCGGCATCGGCCGCCCg 130275

ZFN-unknown-SP-7-498
130283 gCTCGTCGGCCGGCGACGCCGCCGCCg 130309
130283 cGAGCAGCCGGCCGCTGCGGCGGCGGc 130309

ZFN-unknown-SP-6-536
130290 gGCCGGCGACGCCGCCGCCGACGGCa 130315
130290 cCGGCCGCTGCGGCGGCGGCTGCCGt 130315

ZFN-unknown-SP-7-499
130301 cCGCCGCCGACGGCAACGGGGCGGCGg 130327
130301 gGCGGCGGCTGCCGTTGCCCCGCCGCc 130327

ZFN-unknown-SP-6-537
130302 cGCCGCCGACGGCAACGGGGCGGCGg 130327

130302 gCGGCGGCTGCCGTTGCCCCGCCGCc 130327

ZFN-unknown-SP-6-538
130305 cGCCGACGGCAACGGGGCGGCGGCGg 130330
130305 gCGGCTGCCGTTGCCCCGCCGCCGCc 130330

ZFN-unknown-SP-6-539
130308 cGACGGCAACGGGGCGGCGGCGGCGg 130333
130308 gCTGCCGTTGCCCCGCCGCCGCCGCc 130333

ZFN-unknown-SP-6-540
130346 gGGCGTCGTCGCCGTCGTGGCGGTTg 130371
130346 cCCGCAGCAGCGGCAGCACCGCCAAc 130371

ZFN-unknown-SP-5-453
130372 gCGTCGCCGCCGTCGTCGGGGTTc 130396
130372 cGCAGCGGCGGCAGCAGCCCCCAAg 130396

ZFN-unknown-SP-5-454
130470 gCCCTTCTGCGCGCGGTCGTAGCGg 130494
130470 cGGGAAGACGCGCGCCAGCATCGCc 130494

ZFN-unknown-SP-5-455
130578 gGCCCGCGCCACGCTGCCGGAGATg 130602
130578 cCGGGCGCGGTGCGACGGCCTCTAc 130602

ZFN-unknown-SP-6-541
130622 cCGCGCCCGAGATCCGGAAGCAGGCc 130647
130622 gGCGCGGGCTCTAGGCCTTCGTCCGg 130647

ZFN-unknown-SP-7-500
130751 gCGCCGCGTCGCCCAGCTCGGGCGCCc 130777
130751 cGCGGCGCAGCGGGTCGAGCCCGCGGg 130777

ZFN-unknown-SP-6-542
130770 gGGCGCCCACACGGGCGCCGGGGCGc 130795
130770 cCCGCGGGTGTGCCCGCGGCCCCGCg 130795

ZFN-unknown-SP-6-543
130815 cCGCGCCTCCTCCGCCTCGGGCGCCc 130840
130815 gGCGCGGAGGAGGCGGAGCCCGCGGg 130840

ZFN-unknown-SP-5-456
130833 gGGCGCCCCCCAGAGGCCGGGGCGg 130857
130833 cCCGCGGGGGGTCTCCGGCCCCGCc 130857

ZFN-unknown-SP-6-544
130835 gCGCCCCCCAGAGGCCGGGGCGGCTg 130860
130835 cGCGGGGGGTCTCCGGCCCCGCCGAc 130860

ZFN-unknown-SP-6-545
130879 aGCACGCGCCCCGGGGGCGGGGGGCc 130904
130879 tCGTGCGCGGGGCCCCCGCCCCCCGg 130904

ZFN-unknown-SP-5-457
130879 aGCACGCGCCCCGGGGGCGGGGGGc 130903
130879 tCGTGCGCGGGGCCCCCGCCCCCCg 130903

ZFN-unknown-SP-5-458
130911 gGGCCACGGCTCCCCGCTGACGTAc 130935
130911 cCCGGTGCCGAGGGGCGACTGCATg 130935

ZFN-unknown-SP-6-546
131168 cCTCCTCGTCGTCCGCGCCGAGGGTc 131193
131168 gGAGGAGCAGCAGGCGCGGCTCCCAg 131193

ZFN-unknown-SP-6-547
131207 gGTCTGCGGCGCTGGCGGGGGCGCGg 131232
131207 cCAGACGCCGCGACCGCCCCCGCGCc 131232

ZFN-unknown-SP-6-548
131231 gGGCGGCGTCGTCGTCGTCGTCGTCg 131256
131231 cCCGCCGCAGCAGCAGCAGCAGCAGc 131256

ZFN-unknown-SP-6-549
131234 cGGCGTCGTCGTCGTCGTCGTCGTCa 131259
131234 gCCGCAGCAGCAGCAGCAGCAGCAGt 131259

ZFN-unknown-SP-7-501
131243 cGTCGTCGTCGTCGTCAGACGAGGAGg 131269
131243 gCAGCAGCAGCAGCAGTCTGCTCCTCc 131269

ZFN-unknown-SP-7-502
131246 cGTCGTCGTCGTCAGACGAGGAGGCGg 131272
131246 gCAGCAGCAGCAGTCTGCTCCTCCGCc 131272

ZFN-unknown-SP-6-550
131249 cGTCGTCGTCAGACGAGGAGGCGGAt 131274
131249 gCAGCAGCAGTCTGCTCCTCCGCCTa 131274

ZFN-unknown-SP-6-551
131348 aGGCGGCCGAGCGCCGGCGGGGGGCg 131373
131348 tCCGCCGGCTCGCGGCCGCCCCCCGc 131373

ZFN-unknown-SP-5-459
131348 aGGCGGCCGAGCGCCGGCGGGGGGc 131372
131348 tCCGCCGGCTCGCGGCCGCCCCCCg 131372

ZFN-unknown-SP-7-503
131371 gCGCGCCGGCGGCGGTGGTGGTGGTGg 131397
131371 cGCGCGGCCGCCGCCACCACCACCACc 131397

ZFN-unknown-SP-6-552
131371 gCGCGCCGGCGGCGGTGGTGGTGGTg 131396
131371 cGCGCGGCCGCCGCCACCACCACCAc 131396

ZFN-unknown-SP-6-553
131374 cGCCGGCGGCGGTGGTGGTGGTGGTg 131399
131374 gCGGCCGCCGCCACCACCACCACCAc 131399

ZFN-unknown-SP-7-504
131374 cGCCGGCGGCGGTGGTGGTGGTGGTGg 131400
131374 gCGGCCGCCGCCACCACCACCACCACc 131400

ZFN-unknown-SP-5-460
131458 gCCCCGCGGACCGCGGACGTCGTCt 131482
131458 cGGGGCGCCTGGCGCCTGCAGCAGa 131482

ZFN-unknown-SP-5-461
131472 gGACGTCGTCTCCGGTCCGCGGACc 131496
131472 cCTGCAGCAGAGGCCAGGCGCCTGg 131496

ZFN-unknown-SP-7-505
131494 aCCCAGCGGCCCGCGTCGCGGTCGTCg 131520
131494 tGGGTCGCCGGGCGCAGCGCCAGCAGc 131520

ZFN-unknown-SP-6-554
131513 gGTCGTCGTCATCGTCGTCGTCGTCg 131538
131513 cCAGCAGCAGTAGCAGCAGCAGCAGc 131538

ZFN-unknown-SP-6-555

131516 cGTCGTCATCGTCGTCGTCGTCGTCg 131541
131516 gCAGCAGTAGCAGCAGCAGCAGCAGc 131541

ZFN-unknown-SP-6-556
131519 cGTCATCGTCGTCGTCGTCGTCGTCg 131544
131519 gCAGTAGCAGCAGCAGCAGCAGCAGc 131544

ZFN-unknown-SP-6-557
131606 cCGCTTCTTCTTGCGCGCCGTCGCGc 131631
131606 gGCGAAGAAGAACGCGCGGCAGCGCg 131631

ZFN-unknown-SP-7-506
131618 gCGCGCCGTCGCGCTCCGGGGGGGGCg 131644
131618 cGCGCGGCAGCGCGAGGCCCCCCCCGc 131644

ZFN-unknown-SP-5-462
131691 cAGCTGCCGCGGCGAGACGACGCCg 131715
131691 gTCGACGGCGCCGCTCTGCTGCGGc 131715

ZFN-unknown-SP-5-463
131694 cTGCCGCGGCGAGACGACGCCGTCc 131718
131694 gACGGCGCCGCTCTGCTGCGGCAGg 131718

ZFN-unknown-SP-6-558
131714 cGTCCGCGGCAGGCTCGTCGACGGCc 131739
131714 gCAGGCGCCGTCCGAGCAGCTGCCGg 131739

ZFN-unknown-SP-7-507
131729 cGTCGACGGCCTCCCCGGACGCCGGGg 131755
131729 gCAGCTGCCGGAGGGGCCTGCGGCCCc 131755

ZFN-unknown-SP-7-508
131732 cGACGGCCTCCCCGGACGCCGGGGCCg 131758
131732 gCTGCCGGAGGGGCCTGCGGCCCCGGc 131758

ZFN-unknown-SP-7-509
131735 cGGCCTCCCCGGACGCCGGGGCCGCCt 131761
131735 gCCGGAGGGGCCTGCGGCCCCGGCGGa 131761

ZFN-unknown-SP-7-510
131759 cCTCGTCGGCATCGGCATCGGCGGCGg 131785
131759 gGAGCAGCCGTAGCCGTAGCCGCCGCc 131785

ZFN-unknown-SP-7-511
131762 cGTCGGCATCGGCATCGGCGGCGGCGt 131788
131762 gCAGCCGTAGCCGTAGCCGCCGCCGCa 131788

ZFN-unknown-SP-7-512
131765 cGGCATCGGCATCGGCGGCGGCGTCGt 131791
131765 gCCGTAGCCGTAGCCGCCGCCGCAGCa 131791

ZFN-unknown-SP-6-559
131771 cGGCATCGGCGGCGGCGTCGTCGGCc 131796
131771 gCCGTAGCCGCCGCCGCAGCAGCCGg 131796

ZFN-unknown-SP-7-513
131919 cGTCTCCGCCGCGGCCCGGAGACGTCc 131945
131919 gCAGAGGCGGCGCCGGGCCTCTGCAGg 131945

ZFN-unknown-SP-5-464
131921 tCTCCGCCGCGGCCCGGAGACGTCc 131945
131921 aGAGGCGGCGCCGGGCCTCTGCAGg 131945

ZFN-unknown-SP-7-514
131969 cCTCGGCCCCGCGGCCCTGCGTCGTCg 131995
131969 gGAGCCGGGGCGCCGGGACGCAGCAGc 131995

ZFN-unknown-SP-5-465
131974 gCCCCGCGGCCCTGCGTCGTCGTCg 131998
131974 cGGGGCGCCGGGACGCAGCAGCAGc 131998

ZFN-unknown-SP-6-560
132032 tCGCCTCCGACCGGGGTGTGCGGGGg 132057
132032 aGCGGAGGCTGGCCCCACACGCCCCc 132057

ZFN-unknown-SP-5-466
132033 cGCCTCCGACCGGGGTGTGCGGGGg 132057
132033 gCGGAGGCTGGCCCCACACGCCCCc 132057

ZFN-unknown-SP-7-515
132033 cGCCTCCGACCGGGGTGTGCGGGGGGg 132059
132033 gCGGAGGCTGGCCCCACACGCCCCCCc 132059

ZFN-unknown-SP-7-516
132064 cTTCTTCTTCTTCTTCAGGGGCGGCAg 132090
132064 gAAGAAGAAGAAGAAGTCCCCGCCGTc 132090

ZFN-unknown-SP-7-517
132067 cTTCTTCTTCTTCAGGGGCGGCAGTGg 132093
132067 gAAGAAGAAGAAGTCCCCGCCGTCACc 132093

ZFN-unknown-SP-7-518
132070 cTTCTTCTTCAGGGGCGGCAGTGGGGg 132096
132070 gAAGAAGAAGTCCCCGCCGTCACCCCc 132096

ZFN-unknown-SP-6-561
132113 tCTCTCCCCCCCGTGCGGTGCGTGCg 132138
132113 aGAGAGGGGGGGCACGCCACGCACGc 132138

ZFN-unknown-SP-7-519
132304 cCGCCGCGCCCCGGCTCGCCGGGGTTc 132330
132304 gGCGGCGCGGGGCCGAGCGGCCCCAAg 132330

ZFN-unknown-SP-7-520
132377 gCGCGTCCGCGTCCGTGGCGGCGGCCc 132403
132377 cGCGCAGGCGCAGGCACCGCCGCCGGg 132403

ZFN-unknown-SP-6-562
132572 cCCCGCCGGCCCCAAAGGGGCCGGCg 132597
132572 gGGGCGGCCGGGGTTTCCCCGGCCGc 132597

ZFN-unknown-SP-5-467
132621 cGGCTCCGCCCCAAAGGGGGCGGGg 132645
132621 gCCGAGGCGGGGTTTCCCCCGCCCc 132645

ZFN-unknown-SP-6-563
132621 cGGCTCCGCCCCAAAGGGGGCGGGGc 132646
132621 gCCGAGGCGGGGTTTCCCCCGCCCCg 132646

ZFN-unknown-SP-6-564
132623 gCTCCGCCCCAAAGGGGGCGGGGCCg 132648
132623 cGAGGCGGGGTTTCCCCCGCCCCGGc 132648

ZFN-unknown-SP-5-468
132624 cTCCGCCCCAAAGGGGGCGGGGCCg 132648
132624 gAGGCGGGGTTTCCCCCGCCCCGGc 132648

ZFN-unknown-SP-6-565
132759 gCGCTCCGCCCCAAAGGGGGCGGGGc 132784
132759 cGCGAGGCGGGGTTTCCCCCGCCCCg 132784

ZFN-unknown-SP-5-469
132759 gCGCTCCGCCCCAAAGGGGGCGGGg 132783
132759 cGCGAGGCGGGGTTTCCCCCGCCCc 132783

ZFN-unknown-SP-6-566
132761 gCTCCGCCCCAAAGGGGGCGGGGCCg 132786
132761 cGAGGCGGGGTTTCCCCCGCCCCGGc 132786

ZFN-unknown-SP-5-470
132762 cTCCGCCCCAAAGGGGGCGGGGCCg 132786
132762 gAGGCGGGGTTTCCCCCGCCCCGGc 132786

ZFN-unknown-SP-6-567
132957 gGCCGGCGTCCCGGTCGCCGCCGCAc 132982
132957 cCGGCCGCAGGGCCAGCGGCGGCGTg 132982

ZFN-unknown-SP-5-471
132988 gCACACCGGCTGCGCGGCGGAGACc 133012
132988 cGTGTGGCCGACGCGCCGCCTCTGg 133012

ZFN-unknown-SP-5-472
133020 cAGCGGCGGCATCGCGAAGGGGGCc 133044
133020 gTCGCCGCCGTAGCGCTTCCCCCGg 133044

ZFN-unknown-SP-6-568
133197 cCACTGCAGCCTTATCGCAGGTACGg 133222
133197 gGTGACGTCGGAATAGCGTCCATGCc 133222

ZFN-unknown-SP-7-521
133893 cCTCGACCGAGTCGTCCGAGGATGAGg 133919
133893 gGAGCTGGCTCAGCAGGCTCCTACTCc 133919

ZFN-unknown-SP-7-522
133902 aGTCGTCCGAGGATGAGGCGGGAGACc 133928
133902 tCAGCAGGCTCCTACTCCGCCCTCTGg 133928

ZFN-unknown-SP-7-523
133937 gGGCGCCGTCGCTCCCCGCGGGAGCTc 133963
133937 cCCGCGGCAGCGAGGGGCGCCCTCGAg 133963

ZFN-unknown-SP-6-569
134127 gCTCCCCCGACCGGCGTGCGGGTGTg 134152
134127 cGAGGGGGCTGGCCGCACGCCCACAc 134152

ZFN-unknown-SP-7-524
134127 gCTCCCCCGACCGGCGTGCGGGTGTGg 134153
134127 cGAGGGGGCTGGCCGCACGCCCACACc 134153

ZFN-unknown-SP-5-473
134128 cTCCCCCGACCGGCGTGCGGGTGTg 134152
134128 gAGGGGGCTGGCCGCACGCCCACAc 134152

ZFN-unknown-SP-6-570
134128 cTCCCCCGACCGGCGTGCGGGTGTGg 134153
134128 gAGGGGGCTGGCCGCACGCCCACACc 134153

ZFN-unknown-SP-7-525
134152 gGTTTTCCCCGAGGTGTGGAGAAGCGa 134178
134152 cCAAAAGGGGCTCCACACCTCTTCGCt 134178

ZFN-unknown-SP-6-571
134432 gGGCCGCGCACGTGGGGCCGCCTGCt 134457
134432 cCCGGCGCGTGCACCCCGGCGGACGa 134457

ZFN-unknown-SP-5-474
134450 cGCCTGCTGCAGATCTCGGGCGGAa 134474

134450 gCGGACGACGTCTAGAGCCCGCCTt 134474

ZFN-unknown-SP-6-572
134572 gCGTTACGGCCCCGAGTGTGATGTTg 134597
134572 cGCAATGCCGGGGCTCACACTACAAc 134597

ZFN-unknown-SP-6-573
134575 tTACGGCCCCGAGTGTGATGTTGGCa 134600
134575 aATGCCGGGGCTCACACTACAACCGt 134600

ZFN-unknown-SP-7-526
135405 aGACAGCTGCGTTCCCGGTCGCCGGGc 135431
135405 tCTGTCGACGCAAGGGCCAGCGGCCCg 135431

ZFN-unknown-SP-7-527
135555 gGTTCCCCACCCACGCGTCGTTGGTc 135581
135555 cCAAGGGGTGGGTGCGCAGCAACCAg 135581

ZFN-unknown-SP-6-574
135556 gTTCCCCACCCACGCGTCGTTGGTc 135581
135556 cAAGGGGTGGGTGCGCAGCAACCAg 135581

ZFN-unknown-SP-5-475
135557 tTCCCCACCCACGCGTCGTTGGTc 135581
135557 aAGGGGTGGGTGCGCAGCAACCAg 135581

ZFN-unknown-SP-6-575
135655 cCCCGACCACCCACAGGTGGTATAGc 135680
135655 gGGGCTGGTGGGTGTCCACCATATCg 135680

ZFN-unknown-SP-5-476
135964 tTGCGTCAGCGGAAGTCCGCGGCAg 135988
135964 aACGCAGTCGCCTTCAGGCGCCGTc 135988

ZFN-unknown-SP-7-528
136494 gCCCACCGTCCCCGGCGAGGCTGCGc 136520
136494 cGGGTGGCAGGGGCCGCTCCGACGCg 136520

ZFN-unknown-SP-6-576
136567 gGACTACCCCATCGTGGACGCCGCGg 136592
136567 cCTGATGGGGTAGCACCTGCGGCGCc 136592

ZFN-unknown-SP-6-577
136570 cTACCCCATCGTGGACGCCGCGGGTg 136595
136570 gATGGGGTAGCACCTGCGGCGCCCAc 136595

ZFN-unknown-SP-6-578
136744 cCCCGACGTCCGGACCTGCGACACGg 136769
136744 gGGGCTGCAGGCCTGGACGCTGTGCc 136769

ZFN-unknown-SP-5-477
137482 gCGCGACCCCGAAAGGCGGCCGTGc 137506
137482 cGCGCTGGGGCTTTCCGCCGGCACg 137506

ZFN-unknown-SP-7-529
137532 gACAGGCCCAGGTACACGTCGACGAGt 137558
137532 cTGTCCGGGTCCATGTGCAGCTGCTCa 137558

ZFN-unknown-SP-6-579
137590 gCACTACCGCTCGCGGGCGGCCGGGa 137615
137590 cGTGATGGCGAGCGCCCGCCGGCCCt 137615

ZFN-unknown-SP-5-478
137714 gCGCTCCGCCCAAGCGCCGCGGAGt 137738
137714 cGCGAGGCGGGTTCGCGGCGCCTCa 137738

ZFN-unknown-SP-5-479
137727 gCGCCGCGGAGTTGCTGCGCCTGCc 137751
137727 cGCGGCGCCTCAACGACGCGGACGg 137751

ZFN-unknown-SP-5-480
137770 aCCCCGCTCCCCCCGGGGGGCGTGg 137794
137770 tGGGGCGAGGGGGGCCCCCCGCACc 137794

ZFN-unknown-SP-7-530
138238 cGTCAGCCCATCCTCCTTCGGCAGTAt 138264
138238 gCAGTCGGGTAGGAGGAAGCCGTCATa 138264

ZFN-unknown-SP-5-481
138250 cTCCTTCGGCAGTATGGAGGGTGTc 138274
138250 gAGGAAGCCGTCATACCTCCCACAg 138274

ZFN-unknown-SP-6-580
138250 cTCCTTCGGCAGTATGGAGGGTGTCg 138275
138250 gAGGAAGCCGTCATACCTCCCACAGc 138275

ZFN-unknown-SP-7-531
138287 cGCCGTCCCCAAAGACGTGCGGGTCGt 138313
138287 gCGGCAGGGGTTTCTGCACGCCCAGCa 138313

ZFN-unknown-SP-6-581
138491 cCGCCCCCCCACAGGCGGCGCGTGCg 138516
138491 gGCGGGGGGGTGTCCGCCGCGCACGc 138516

ZFN-unknown-SP-5-482
138492 cGCCCCCCCACAGGCGGCGCGTGCg 138516
138492 gCGGGGGGGTGTCCGCCGCGCACGc 138516

ZFN-unknown-SP-7-532
138500 cACAGGCGGCGCGTGCGGAGGGCGGCc 138526
138500 gTGTCCGCCGCGCACGCCTCCCGCCGg 138526

ZFN-unknown-SP-7-533
138591 cCCCAACCCCGGGTTTCGTGGCCTGCg 138617
138591 gGGGTTGGGGCCCAAAGCACCGGACGc 138617

ZFN-unknown-SP-6-582
138683 tTGCTCCCCAGAGCCTGCTGGTGGGg 138708
138683 aACGAGGGGTCTCGGACGACCACCCc 138708

ZFN-unknown-SP-5-483
138736 gCACGACCCACGGAAGACGTCGGGg 138760
138736 cGTGCTGGGTGCCTTCTGCAGCCCc 138760

ZFN-unknown-SP-6-583
138938 cGCCTACGTCCGCTCCGGAGAAGACg 138963
138938 gCGGATGCAGGCGAGGCCTCTTCTGc 138963

ZFN-unknown-SP-6-584
138941 cTACGTCCGCTCCGGAGAAGACGCCc 138966
138941 gATGCAGGCGAGGCCTCTTCTGCGGg 138966

ZFN-unknown-SP-7-534
139061 cACAAGCCGCAACGGTCGCCGTTACGc 139087
139061 gTGTTCGGCGTTGCCAGCGGCAATGCg 139087

ZFN-unknown-SP-7-535
139112 cGCCCGCGACTGCATCCGTGGAGTCGt 139138
139112 gCGGGCGCTGACGTAGGCACCTCAGCa 139138

ZFN-unknown-SP-6-585

139115 cCGCGACTGCATCCGTGGAGTCGTCg 139140
139115 gGCGCTGACGTAGGCACCTCAGCAGc 139140

ZFN-unknown-SP-6-586
139118 cGACTGCATCCGTGGAGTCGTCGCCa 139143
139118 gCTGACGTAGGCACCTCAGCAGCGGt 139143

ZFN-unknown-SP-5-484
139135 tCGTCGCCACTCCCCGCCGCGGCGg 139159
139135 aGCAGCGGTGAGGGGCGGCGCCGCc 139159

ZFN-unknown-SP-5-485
139138 tCGCCACTCCCCGCCGCGGCGGCGg 139162
139138 aGCGGTGAGGGGCGGCGCCGCCGCc 139162

ZFN-unknown-SP-6-587
139143 aCTCCCCGCCGCGGCGGCGGCAACGc 139168
139143 tGAGGGGCGGCGCCGCCGCCGTTGCg 139168

ZFN-unknown-SP-7-536
139154 cGGCGGCGGCAACGCCCGGGGCCGGGc 139180
139154 gCCGCCGCCGTTGCGGGCCCCGGCCCg 139180

ZFN-unknown-SP-7-537
139313 cCGCAACCCCGGGTCCGGTGGGCGCCt 139339
139313 gGCGTTGGGGCCCAGGCCACCCGCGGa 139339

ZFN-unknown-SP-6-588
139376 cGCCCCCCGCTACCGCGCCGGGGCCc 139401
139376 gCGGGGGGCGATGGCGCGGCCCCGGg 139401

ZFN-unknown-SP-7-538
139385 cTACCGCGCCGGGGCCCTCGGCCGCCa 139411
139385 gATGGCGCGGCCCCGGGAGCCGGCGGt 139411

ZFN-unknown-SP-7-539
139400 cCTCGGCCGCCAACGTTTCGGTCGCCg 139426
139400 gGAGCCGGCGGTTGCAAAGCCAGCGGc 139426

ZFN-unknown-SP-7-540
139487 cGCACCCACACGGACCCGCGGACGCTc 139513
139487 gCGTGGGTGTGCCTGGGCGCCTGCGAg 139513

ZFN-unknown-SP-5-486
139535 cACCCCCCGAACATCGCGGCGGACc 139559
139535 gTGGGGGGCTTGTAGCGCCGCCTGg 139559

ZFN-unknown-SP-5-487
139886 tGGCAGCACAACTTTGGCGCGGCCg 139910
139886 aCCGTCGTGTTGAAACCGCGCCGGc 139910

ZFN-unknown-SP-6-589
139958 tGCCACCCGAGCGGGATTAGGGGGTg 139983
139958 aCGGTGGGCTCGCCCTAATCCCCCAc 139983

ZFN-unknown-SP-7-541
139958 tGCCACCCGAGCGGGATTAGGGGGTGg 139984
139958 aCGGTGGGCTCGCCCTAATCCCCCACc 139984

ZFN-unknown-SP-7-542
140436 tGCCGTCCCCCTCATCGTGGGGGGGCt 140462
140436 aCGGCAGGGGGAGTAGCACCCCCCCGa 140462

ZFN-unknown-SP-6-590
140436 tGCCGTCCCCCTCATCGTGGGGGGGc 140461
140436 aCGGCAGGGGGAGTAGCACCCCCCCg 140461

ZFN-unknown-SP-5-488
140439 cGTCCCCCTCATCGTGGGGGGGCTg 140463
140439 gCAGGGGGAGTAGCACCCCCCCGAc 140463

ZFN-unknown-SP-5-489
140442 cCCCCTCATCGTGGGGGGGCTGTGt 140466
140442 gGGGGAGTAGCACCCCCCCGACACa 140466

ZFN-unknown-SP-7-543
140593 cCCCATCGCCCCGATTGTGTGTCGGGt 140619
140593 gGGGTAGCGGGGCTAACACACAGCCCa 140619

ZFN-unknown-SP-6-591
140627 gTACAGCAGCTATGGAGCGGTCGGTa 140652
140627 cATGTCGTCGATACCTCGCCAGCCAt 140652

ZFN-unknown-SP-5-490
140733 gCGTACCAACTGGGGGGGGGGGGGg 140757
140733 cGCATGGTTGACCCCCCCCCCCCCc 140757

ZFN-unknown-SP-6-592
140733 gCGTACCAACTGGGGGGGGGGGGGGg 140758
140733 cGCATGGTTGACCCCCCCCCCCCCCc 140758

ZFN-unknown-SP-7-544
140733 gCGTACCAACTGGGGGGGGGGGGGGGg 140759
140733 cGCATGGTTGACCCCCCCCCCCCCCCc 140759

ZFN-unknown-SP-5-491
141027 gACCTCCGGCGTCGGGACGGCGGCc 141051
141027 cTGGAGGCCGCAGCCCTGCCGCCGg 141051

ZFN-unknown-SP-7-545
141041 gGACGGCGGCCCTGCTAGTTGTCGCGg 141067
141041 cCTGCCGCCGGGACGATCAACAGCGCc 141067

ZFN-unknown-SP-5-492
141102 aGCAGACCCCTCGCTTAAGATGGCc 141126
141102 tCGTCTGGGGAGCGAATTCTACCGg 141126

ZFN-unknown-SP-5-493
141174 gACCGACCCCCCCGGGGTGAAGCGt 141198
141174 cTGGCTGGGGGGGCCCCACTTCGCa 141198

ZFN-unknown-SP-6-593
141176 cCGACCCCCCCGGGGTGAAGCGTGTt 141201
141176 gGCTGGGGGGGCCCCACTTCGCACAa 141201

ZFN-unknown-SP-5-494
141177 cGACCCCCCCGGGGTGAAGCGTGTt 141201
141177 gCTGGGGGGGCCCCACTTCGCACAa 141201

ZFN-unknown-SP-5-495
141317 aGGCCCCCCAGATCGTGCGCGGGGc 141341
141317 tCCGGGGGGTCTAGCACGCGCCCCg 141341

ZFN-unknown-SP-5-496
141441 gTGCCCCTACAACAAGTCGTTGGGg 141465
141441 cACGGGGATGTTGTTCAGCAACCCc 141465

ZFN-unknown-SP-5-497
141444 cCCCTACAACAAGTCGTTGGGGGTc 141468
141444 gGGGATGTTGTTCAGCAACCCCCAg 141468

ZFN-unknown-SP-6-594
141555 cCCCGCCTTCGAGACCGCGGTACGt 141580
141555 gGGGCGGAAGCTCTGGCGCCCATGCa 141580

ZFN-unknown-SP-5-498
141831 gTACACCAGCACCCTGCTGCCGCCg 141855
141831 cATGTGGTCGTGGGACGACGGCGGc 141855

ZFN-unknown-SP-5-499
141834 cACCAGCACCCTGCTGCCGCCGGAg 141858
141834 gTGGTCGTGGGACGACGGCGGCCTc 141858

ZFN-unknown-SP-6-595
142140 tCTCCCCCACATCCGGGATGACGACg 142165
142140 aGAGGGGGTGTAGGCCCTACTGCTGc 142165

ZFN-unknown-SP-5-500
142141 cTCCCCCACATCCGGGATGACGACg 142165
142141 gAGGGGGTGTAGGCCCTACTGCTGc 142165

ZFN-unknown-SP-6-596
142143 cCCCCACATCCGGGATGACGACGCGc 142168
142143 gGGGGTGTAGGCCCTACTGCTGCGCg 142168

ZFN-unknown-SP-7-546
142449 cGCCACCGGCCTGGTCGTCCGCGGCCc 142475
142449 gCGGTGGCCGGACCAGCAGGCGCCGGg 142475

ZFN-unknown-SP-6-597
142686 aTGCCCCCGCCGCCCCGCCGTGGCGt 142711
142686 tACGGGGGCGGCGGGGCGGCACCGCa 142711

ZFN-unknown-SP-5-501
142687 tGCCCCCGCCGCCCCGCCGTGGCGt 142711
142687 aCGGGGGCGGCGGGGCGGCACCGCa 142711

ZFN-unknown-SP-5-502
142935 cGGCTCCTGCGATCCGGCGCAGCTt 142959
142935 gCCGAGGACGCTAGGCCGCGTCGAa 142959

ZFN-unknown-SP-7-547
143367 cACCCCCCCCAAACCCCGACGCCGTTc 143393
143367 gTGGGGGGGGTTTGGGGCTGCGGCAAg 143393

ZFN-unknown-SP-6-598
143368 aCCCCCCCCAAACCCCGACGCCGTTc 143393
143368 tGGGGGGGGTTTGGGGCTGCGGCAAg 143393

ZFN-unknown-SP-7-548
143377 aAACCCCGACGCCGTTCGTCGTCGTCc 143403
143377 tTTGGGGCTGCGGCAAGCAGCAGCAGg 143403

ZFN-unknown-SP-7-549
143492 gCCCGACGGCCCCCCAAGAGGTCTAGg 143518
143492 cGGGCTGCCGGGGGGTTCTCCAGATCc 143518

ZFN-unknown-SP-5-503
143728 gGCCCGCCGCGCATTTAAGGAGTCg 143752
143728 cCGGGCGGCGCGTAAATTCCTCAGc 143752

ZFN-unknown-SP-5-504
143731 cCGCCGCGCATTTAAGGAGTCGCCg 143755
143731 gGCGGCGCGTAAATTCCTCAGCGGc 143755

ZFN-unknown-SP-5-505
143987 cACAAACTACTGTGGGCCGCGGAAc 144011

143987 gTGTTTGATGACACCCGGCGCCTTg 144011

ZFN-unknown-SP-7-550
144000 gGGCCGCGGAACCCCTGGATGCCTGCg 144026
144000 cCCGGCGCCTTGGGGACCTACGGACGc 144026

ZFN-unknown-SP-7-551
144026 gGTCCCCTGCGCCCGTCGTGGGTGGCg 144052
144026 cCAGGGGACGCGGGCAGCACCCACCGc 144052

ZFN-unknown-SP-7-552
144029 cCCCTGCGCCCGTCGTGGGTGGCGCTg 144055
144029 gGGGACGCGGGCAGCACCCACCGCGAc 144055

ZFN-unknown-SP-5-506
144029 cCCCTGCGCCCGTCGTGGGTGGCGc 144053
144029 gGGGACGCGGGCAGCACCCACCGCg 144053

ZFN-unknown-SP-7-553
144342 cGACCCCCGACGACTACGACGAAGAAg 144368
144342 gCTGGGGGCTGCTGATGCTGCTTCTTc 144368

ZFN-unknown-SP-6-599
144343 gACCCCCGACGACTACGACGAAGAAg 144368
144343 cTGGGGGCTGCTGATGCTGCTTCTTc 144368

ZFN-unknown-SP-6-600
144346 cCCCGACGACTACGACGAAGAAGACg 144371
144346 gGGGCTGCTGATGCTGCTTCTTCTGc 144371

ZFN-unknown-SP-6-601
144349 cGACGACTACGACGAAGAAGACGACg 144374
144349 gCTGCTGATGCTGCTTCTTCTGCTGc 144374

ZFN-unknown-SP-6-602
144352 cGACTACGACGAAGAAGACGACGCGg 144377
144352 gCTGATGCTGCTTCTTCTGCTGCGCc 144377

ZFN-unknown-SP-7-554
144699 cTCCGGCCGACGCGCCGTGCGCCGTAa 144725
144699 gAGGCCGGCTGCGCGGCACGCGGCATt 144725

ZFN-unknown-SP-5-507
144946 gACCATCAGCACCGCGGCGCAGTAc 144970
144946 cTGGTAGTCGTGGCGCCGCGTCATg 144970

ZFN-unknown-SP-7-555
145073 gGCCCGCTGCGCCTCGGGGCGGTGCTg 145099
145073 cCGGGCGACGCGGAGCCCCGCCACGAc 145099

ZFN-unknown-SP-5-508
145073 gGCCCGCTGCGCCTCGGGGCGGTGc 145097
145073 cCGGGCGACGCGGAGCCCCGCCACg 145097

ZFN-unknown-SP-7-556
145076 cCGCTGCGCCTCGGGGCGGTGCTGGGg 145102
145076 gGCGACGCGGAGCCCCGCCACGACCCc 145102

ZFN-unknown-SP-7-557
145206 gCCCCACTTACATTCGCGTGGCGGACa 145232
145206 cGGGGTGAATGTAAGCGCACCGCCTGt 145232

ZFN-unknown-SP-7-558
145480 aGGCGTCTTCCGACGACGCGGACGTCg 145506
145480 tCCGCAGAAGGCTGCTGCGCCTGCAGc 145506

ZFN-unknown-SP-7-559
145483 cGTCTTCCGACGACGCGGACGTCGGCg 145509
145483 gCAGAAGGCTGCTGCGCCTGCAGCCGc 145509

ZFN-unknown-SP-5-509
145554 aCCCTGCCCCCCGGCGCGGGCGGCg 145578
145554 tGGGACGGGGGCCGCGCCCGCCGc 145578

ZFN-unknown-SP-6-603
145559 gCCCCCCGGCGCGGGCGGCGCCGCGc 145584
145559 cGGGGGGCCGCGCCCGCCGCGGCGCg 145584

ZFN-unknown-SP-5-510
145576 gCGCCGCGCCCGCGTGCCGCAGACc 145600
145576 cGCGGCGCGGGCGCACGGCGTCTGg 145600

ZFN-unknown-SP-6-604
145772 gTCCGGCGGCCACCCCGCCGCAGCTc 145797
145772 cAGGCCGCCGGTGGGGCGGCGTCGAg 145797

ZFN-unknown-SP-5-511
145899 gCGTTCCAGCGCGTCGGTGCCGCTt 145923
145899 cGCAAGGTCGCGCAGCCACGGCGAa 145923

ZFN-unknown-SP-7-560
145931 cGGCCTCGCCCGTCCCGGCAGAAGCCt 145957
145931 gCCGGAGCGGGCAGGGCCGTCTTCGGa 145957

ZFN-unknown-SP-7-561
145989 cGACTTCCTCGTGCGCATGGGCCGCCa 146015
145989 gCTGAAGGAGCACGCGTACCCGGCGGt 146015

ZFN-unknown-SP-6-605
146035 cGACGGCGGCGCACGCGGTGCGTCGg 146060
146035 gCTGCCGCCGCGTGCGCCACGCAGCc 146060

ZFN-unknown-SP-5-512
146038 cGGCGGCGCACGCGGTGCGTCGGGc 146062
146038 gCCGCCGCGTGCGCCACGCAGCCCg 146062

ZFN-unknown-SP-6-606
146064 gGTTATCGCCTGTCTCGTCGTGGCCc 146089
146064 cCAATAGCGGACAGAGCAGCACCGGg 146089

ZFN-unknown-SP-7-562
146420 tCGCCCCCCCCCCCCCAGGAGGGGGGc 146446
146420 aGCGGGGGGGGGGGGGTCCTCCCCCCg 146446

ZFN-unknown-SP-6-607
146421 cGCCCCCCCCCCCCCAGGAGGGGGGc 146446
146421 gCGGGGGGGGGGGGGTCCTCCCCCCg 146446

ZFN-unknown-SP-7-563
146421 cGCCCCCCCCCCCCCAGGAGGGGGGCa 146447
146421 gCGGGGGGGGGGGGGTCCTCCCCCCGt 146447

ZFN-unknown-SP-5-513
146422 gCCCCCCCCCCCCCAGGAGGGGGGc 146446
146422 cGGGGGGGGGGGGGTCCTCCCCCCg 146446

ZFN-unknown-SP-6-608
146422 gCCCCCCCCCCCCCAGGAGGGGGGCa 146447
146422 cGGGGGGGGGGGGGTCCTCCCCCCGt 146447

ZFN-unknown-SP-5-514

146423 cCCCCCCCCCCCCAGGAGGGGGGCa 146447
146423 gGGGGGGGGGGGTCCTCCCCCCGt 146447

ZFN-unknown-SP-7-564
146425 cCCCCCCCCCCAGGAGGGGGGCAGTGc 146451
146425 gGGGGGGGGGGTCCTCCCCCCGTCACg 146451

ZFN-unknown-SP-6-609
146426 cCCCCCCCCCAGGAGGGGGGCAGTGc 146451
146426 gGGGGGGGGGTCCTCCCCCGTCACg 146451

ZFN-unknown-SP-5-515
146427 cCCCCCCCCAGGAGGGGGGCAGTGc 146451
146427 gGGGGGGGGTCCTCCCCCCGTCACg 146451

ZFN-unknown-SP-5-516
146641 cCCTGTCCCCGGGACGTGGTCAGGa 146665
146641 gGGACAGGGGCCCTGCACCAGTCCt 146665

ZFN-unknown-SP-5-517
146930 tGGCTCCCGCCAGCGGGGGCGGTGg 146954
146930 aCCGAGGGCGGTCGCCCCCGCCACc 146954

ZFN-unknown-SP-7-565
146930 tGGCTCCCGCCAGCGGGGGCGGTGGGt 146956
146930 aCCGAGGGCGGTCGCCCCCGCCACCCa 146956

ZFN-unknown-SP-7-566
147214 gGGCTGCCGAGGGGGGCGTAGGGGACc 147240
147214 cCCGACGGCTCCCCCCGCATCCCCTGg 147240

ZFN-unknown-SP-5-518
147477 gGGCCGCACAGACTCGTAGTAGACc 147501
147477 cCCGGCGTGTCTGAGCATCATCTGg 147501

ZFN-unknown-SP-5-519
147585 gCGTGTCCGCTCCTCTGGGCTGCAt 147609
147585 cGCACAGGCGAGGAGACCCGACGTa 147609

ZFN-unknown-SP-5-520
147588 tGTCCGCTCCTCTGGGCTGCATGCc 147612
147588 aCAGGCGAGGAGACCCGACGTACGg 147612

ZFN-unknown-SP-6-610
148520 cCGTACCTGCGATAAGGCTGCAGTGg 148545
148520 gGCATGGACGCTATTCCGACGTCACc 148545

ZFN-unknown-SP-5-521
148698 gGCCCCCTTCGCGATGCCGCCGCTg 148722
148698 cCGGGGGAAGCGCTACGGCGGCGAc 148722

ZFN-unknown-SP-5-522
148730 gGTCTCCGCCGCGCAGCCGGTGTGc 148754
148730 cCAGAGGCGGCGCGTCGGCCACACg 148754

ZFN-unknown-SP-6-611
148760 gTGCGGCGGCGACCGGGACGCCGGCc 148785
148760 cACGCCGCCGCTGGCCCTGCGGCCGg 148785

ZFN-unknown-SP-6-612
148956 cGGCCCCGCCCCCTTTGGGGCGGAGc 148981
148956 gCCGGGGCGGGGGAAACCCCGCCTCg 148981

ZFN-unknown-SP-5-523
148956 cGGCCCCGCCCCCTTTGGGGCGGAg 148980
148956 gCCGGGGCGGGGGAAACCCCGCCTc 148980

ZFN-unknown-SP-6-613
148958 gCCCCGCCCCCTTTGGGGCGGAGCGc 148983
148958 cGGGGCGGGGGAAACCCCGCCTCGCg 148983

ZFN-unknown-SP-5-524
148959 cCCCGCCCCCTTTGGGGCGGAGCGc 148983
148959 gGGGCGGGGGAAACCCCGCCTCGCg 148983

ZFN-unknown-SP-6-614
149094 cGGCCCCGCCCCCTTTGGGGCGGAGc 149119
149094 gCCGGGGCGGGGGAAACCCCGCCTCg 149119

ZFN-unknown-SP-5-525
149094 cGGCCCCGCCCCCTTTGGGGCGGAg 149118
149094 gCCGGGGCGGGGGAAACCCCGCCTc 149118

ZFN-unknown-SP-6-615
149096 gCCCCGCCCCCTTTGGGGCGGAGCCg 149121
149096 cGGGGCGGGGGAAACCCCGCCTCGGc 149121

ZFN-unknown-SP-5-526
149097 cCCCGCCCCCTTTGGGGCGGAGCCg 149121
149097 gGGGCGGGGGAAACCCCGCCTCGGc 149121

ZFN-unknown-SP-6-616
149145 cGCCGGCCCCTTTGGGGCCGGCGGGg 149170
149145 gCGGCCGGGGAAACCCCGGCCGCCCc 149170

ZFN-unknown-SP-7-567
149339 gGGCCGCCGCCACGGACGCGGACGCGc 149365
149339 cCCGGCGGCGGTGCCTGCGCCTGCGCg 149365

ZFN-unknown-SP-7-568
149412 gAACCCCGGCGAGCCGGGGCGCGGCGg 149438
149412 cTTGGGGCCGCTCGGCCCCGCGCCGCc 149438

ZFN-unknown-SP-6-617
149604 cGCACGCACCGCACGGGGGGGAGAGa 149629
149604 gCGTGCGTGGCGTGCCCCCCCTCTCt 149629

ZFN-unknown-SP-7-569
149646 cCCCCACTGCCGCCCCTGAAGAAGAAg 149672
149646 gGGGGTGACGGCGGGGACTTCTTCTTc 149672

ZFN-unknown-SP-7-570
149649 cCACTGCCGCCCCTGAAGAAGAAGAAg 149675
149649 gGTGACGGCGGGGACTTCTTCTTCTTc 149675

ZFN-unknown-SP-7-571
149652 cTGCCGCCCCTGAAGAAGAAGAAGAAg 149678
149652 gACGGCGGGGACTTCTTCTTCTTCTTc 149678

ZFN-unknown-SP-7-572
149683 cCCCCCCGCACACCCCGGTCGGAGGCg 149709
149683 gGGGGGGCGTGTGGGGCCAGCCTCCGc 149709

ZFN-unknown-SP-6-618
149685 cCCCCGCACACCCCGGTCGGAGGCGa 149710
149685 gGGGGCGTGTGGGGCCAGCCTCCGCt 149710

ZFN-unknown-SP-5-527
149685 cCCCCGCACACCCCGGTCGGAGGCg 149709
149685 gGGGGCGTGTGGGGCCAGCCTCCGc 149709

ZFN-unknown-SP-5-528
149744 cGACGACGACGCAGGGCCGCGGGGc 149768
149744 gCTGCTGCTGCGTCCCGGCGCCCCg 149768

ZFN-unknown-SP-7-573
149747 cGACGACGCAGGGCCGCGGGGCCGAGg 149773
149747 gCTGCTGCGTCCCGGCGCCCCGGCTCc 149773

ZFN-unknown-SP-7-574
149797 gGACGTCTCCGGGCCGCGGCGGAGACg 149823
149797 cCTGCAGAGGCCCGGCGCCGCCTCTGc 149823

ZFN-unknown-SP-5-529
149797 gGACGTCTCCGGGCCGCGGCGGAGa 149821
149797 cCTGCAGAGGCCCGGCGCCGCCTCt 149821

ZFN-unknown-SP-6-619
149946 gGCCGACGACGCCGCCGCCGATGCCg 149971
149946 cCGGCTGCTGCGGCGGCGGCTACGGc 149971

ZFN-unknown-SP-7-575
149951 aCGACGCCGCCGCCGATGCCGATGCCg 149977
149951 tGCTGCGGCGGCGGCTACGGCTACGGc 149977

ZFN-unknown-SP-7-576
149954 aCGCCGCCGCCGATGCCGATGCCGACg 149980
149954 tGCGGCGGCGGCTACGGCTACGGCTGc 149980

ZFN-unknown-SP-7-577
149957 cCGCCGCCGATGCCGATGCCGACGAGg 149983
149957 gGCGGCGGCTACGGCTACGGCTGCTCc 149983

ZFN-unknown-SP-7-578
149981 aGGCGGCCCCGGCGTCCGGGGAGGCCg 150007
149981 tCCGCCGGGGCCGCAGGCCCCTCCGGc 150007

ZFN-unknown-SP-7-579
149984 cGGCCCCGGCGTCCGGGGAGGCCGTCg 150010
149984 gCCGGGGCCGCAGGCCCCTCCGGCAGc 150010

ZFN-unknown-SP-7-580
149987 cCCCGGCGTCCGGGGAGGCCGTCGACg 150013
149987 gGGGCCGCAGGCCCCTCCGGCAGCTGc 150013

ZFN-unknown-SP-6-620
150003 gGCCGTCGACGAGCCTGCCGCGGACg 150028
150003 cCGGCAGCTGCTCGGACGGCGCCTGc 150028

ZFN-unknown-SP-5-530
150024 gGACGGCGTCGTCTCGCCGCGGCAg 150048
150024 cCTGCCGCAGCAGAGCGGCGCCGTc 150048

ZFN-unknown-SP-5-531
150027 cGGCGTCGTCTCGCCGCGGCAGCTg 150051
150027 gCCGCAGCAGAGCGGCGCCGTCGAc 150051

ZFN-unknown-SP-7-581
150098 cGCCCCCCCCGGAGCGCGACGGCGCGc 150124
150098 gCGGGGGGGGCCTCGCGCTGCCGCGCg 150124

ZFN-unknown-SP-6-621
150111 gCGCGACGGCGCGCAAGAAGAAGCGg 150136
150111 cGCGCTGCCGCGCGTTCTTCTTCGCc 150136

ZFN-unknown-SP-6-622
150198 cGACGACGACGACGACGACGATGACg 150223

150198 gCTGCTGCTGCTGCTGCTACTGc 150223

ZFN-unknown-SP-6-623
150201 cGACGACGACGACGACGATGACGACg 150226
150201 gCTGCTGCTGCTGCTACTGCTGc 150226

ZFN-unknown-SP-6-624
150204 cGACGACGACGATGACGACGACc 150229
150204 gCTGCTGCTGCTACTGCTGCTGg 150229

ZFN-unknown-SP-7-582
150222 cGACGACCGCGACGCGGGCCGCTGGGt 150248
150222 gCTGCTGGCGCTGCGCCCGGCGACCCa 150248

ZFN-unknown-SP-5-532
150246 gGTCCGCGGACCGGAGACGACGTCc 150270
150246 cCAGGCGCCTGGCCTCTGCTGCAGg 150270

ZFN-unknown-SP-5-533
150260 aGACGACGTCCGCGGTCCGCGGGGc 150284
150260 tCTGCTGCAGGCGCCAGGCGCCCCg 150284

ZFN-unknown-SP-7-583
150342 cCACCACCACCACCACCGCCGCCGGCg 150368
150342 gGTGGTGGTGGTGGTGGCGGCGGCCGc 150368

ZFN-unknown-SP-6-625
150343 cACCACCACCACCACCGCCGCCGGCg 150368
150343 gTGGTGGTGGTGGTGGCGGCGGCCGc 150368

ZFN-unknown-SP-7-584
150345 cCACCACCACCACCGCCGCCGGCGCGc 150371
150345 gGTGGTGGTGGTGGCGGCGGCCGCGCg 150371

ZFN-unknown-SP-6-626
150346 cACCACCACCACCGCCGCCGGCGCGc 150371
150346 gTGGTGGTGGTGGCGGCGGCCGCGCg 150371

ZFN-unknown-SP-6-627
150369 cGCCCCCCGCCGGCGCTCGGCCGCCt 150394
150369 gCGGGGGGCGGCCGCGAGCCGGCGGa 150394

ZFN-unknown-SP-5-534
150370 gCCCCCCGCCGGCGCTCGGCCGCCt 150394
150370 cGGGGGGCGGCCGCGAGCCGGCGGa 150394

ZFN-unknown-SP-6-628
150468 aTCCGCCTCCTCGTCTGACGACGACg 150493
150468 tAGGCGGAGGAGCAGACTGCTGCTGc 150493

ZFN-unknown-SP-7-585
150470 cCGCCTCCTCGTCTGACGACGACGACg 150496
150470 gGCGGAGGAGCAGACTGCTGCTGCTGc 150496

ZFN-unknown-SP-7-586
150473 cCTCCTCGTCTGACGACGACGACGACg 150499
150473 gGAGGAGCAGACTGCTGCTGCTGCTGc 150499

ZFN-unknown-SP-6-629
150483 tGACGACGACGACGACGACGACGCCg 150508
150483 aCTGCTGCTGCTGCTGCTGCTGCGGc 150508

ZFN-unknown-SP-6-630
150486 cGACGACGACGACGACGACGCCGCCc 150511
150486 gCTGCTGCTGCTGCTGCTGCGGCGGg 150511

```
ZFN-unknown-SP-6-631
150510 cCGCGCCCCCGCCAGCGCCGCAGACc 150535
150510 gGCGCGGGGGCGGTCGCGGCGTCTGg 150535

ZFN-unknown-SP-6-632
150549 gACCCTCGGCGCGGACGACGAGGAGg 150574
150549 cTGGGAGCCGCGCCTGCTGCTCCTCc 150574

ZFN-unknown-SP-5-535
150807 gTACGTCAGCGGGGAGCCGTGGCCc 150831
150807 cATGCAGTCGCCCCTCGGCACCGGg 150831

ZFN-unknown-SP-6-633
150838 gGCCCCCCGCCCCCGGGGCGCGTGCt 150863
150838 cCGGGGGGCGGGGGCCCCGCGCACGa 150863

ZFN-unknown-SP-5-536
150839 gCCCCCCGCCCCCGGGGCGCGTGCt 150863
150839 cGGGGGGCGGGGGCCCCGCGCACGa 150863

ZFN-unknown-SP-6-634
150882 cAGCCGCCCCGGCCTCTGGGGGGCGc 150907
150882 gTCGGCGGGGCCGGAGACCCCCCGCg 150907

ZFN-unknown-SP-5-537
150885 cCGCCCCGGCCTCTGGGGGGCGCCc 150909
150885 gGCGGGGCCGGAGACCCCCCGCGGg 150909

ZFN-unknown-SP-6-635
150902 gGGCGCCCGAGGCGGAGGAGGCGCGg 150927
150902 cCCGCGGGCTCCGCCTCCTCCGCGCc 150927

ZFN-unknown-SP-6-636
150947 gCGCCCCGGCGCCCGTGTGGGCGCCc 150972
150947 cGCGGGGCCGCGGGCACACCCGCGGg 150972

ZFN-unknown-SP-7-587
150965 gGGCGCCCGAGCTGGGCGACGCGGCGc 150991
150965 cCCGCGGGCTCGACCCGCTGCGCCGCg 150991

ZFN-unknown-SP-6-637
151095 gGCCTGCTTCCGGATCTCGGGCGCGg 151120
151095 cCGGACGAAGGCCTAGAGCCCGCGCc 151120

ZFN-unknown-SP-5-538
151140 cATCTCCGGCAGCGTGGCGCGGGCc 151164
151140 gTAGAGGCCGTCGCACCGCGCCCGg 151164

ZFN-unknown-SP-5-539
151248 cCGCTACGACCGCGCGCAGAAGGGc 151272
151248 gGCGATGCTGGCGCGCGTCTTCCCg 151272

ZFN-unknown-SP-5-540
151346 gAACCCCCGACGACGGCGGCGACGc 151370
151346 cTTGGGGGCTGCTGCCGCCGCTGCg 151370

ZFN-unknown-SP-6-638
151371 cAACCGCCACGACGGCGACGACGCCc 151396
151371 gTTGGCGGTGCTGCCGCTGCTGCGGg 151396

ZFN-unknown-SP-6-639
151409 cCGCCGCCGCCGCCCCGTTGCCGTCg 151434
151409 gGCGGCGGCGGCGGGGCAACGGCAGc 151434

ZFN-unknown-SP-6-640
```

151412 cCGCCGCCGCCCCGTTGCCGTCGGCg 151437
151412 gGCGGCGGCGGGGCAACGGCAGCCGc 151437

ZFN-unknown-SP-6-641
151415 cCGCCGCCCCGTTGCCGTCGGCGGCg 151440
151415 gGCGGCGGGGCAACGGCAGCCGCCGc 151440

ZFN-unknown-SP-7-588
151415 cCGCCGCCCCGTTGCCGTCGGCGGCGg 151441
151415 gGCGGCGGGGCAACGGCAGCCGCCGCc 151441

ZFN-unknown-SP-6-642
151427 tGCCGTCGGCGGCGGCGTCGCCGGCc 151452
151427 aCGGCAGCCGCCGCCGCAGCGGCCGg 151452

ZFN-unknown-SP-7-589
151433 cGGCGGCGGCGTCGCCGGCCGACGAGc 151459
151433 gCCGCCGCCGCAGCGGCCGGCTGCTCg 151459

ZFN-unknown-SP-6-643
151467 gCCCGCCGGCTACGGCGCCGCGGGGg 151492
151467 cGGGCGGCCGATGCCGCGGCGCCCCc 151492

ZFN-unknown-SP-6-644
151470 cGCCGGCTACGGCGCCGCGGGGGTGc 151495
151470 gCGGCCGATGCCGCGGCGCCCCCACg 151495

ZFN-unknown-SP-5-541
151473 cGGCTACGGCGCCGCGGGGGTGCTc 151497
151473 gCCGATGCCGCGGCGCCCCCACGAg 151497

ZFN-unknown-SP-7-590
151523 cGCCCGCCTCCGCGCCGGCCGGGGCCg 151549
151523 gCGGGCGGAGGCGCGGCCGGCCCCGGc 151549

ZFN-unknown-SP-6-645
151524 gCCCGCCTCCGCGCCGGCCGGGGCCg 151549
151524 cGGGCGGAGGCGCGGCCGGCCCCGGc 151549

ZFN-unknown-SP-7-591
151526 cCGCCTCCGCGCCGGCCGGGGCCGACg 151552
151526 gGCGGAGGCGCGGCCGGCCCCGGCTGc 151552

ZFN-unknown-SP-7-592
151529 cCTCCGCGCCGGCCGGGGCCGACGACg 151555
151529 gGAGGCGCGGCCGGCCCCGGCTGCTGc 151555

ZFN-unknown-SP-7-593
151532 cCGCGCCGGCCGGGGCCGACGACGACg 151558
151532 gGCGCGGCCGGCCCCGGCTGCTGCTGc 151558

ZFN-unknown-SP-6-646
151545 gGCCGACGACGACGACGACGACGACg 151570
151545 cCGGCTGCTGCTGCTGCTGCTGCTGc 151570

ZFN-unknown-SP-6-647
151548 cGACGACGACGACGACGACGACGGCg 151573
151548 gCTGCTGCTGCTGCTGCTGCTGCCGc 151573

ZFN-unknown-SP-6-648
151551 cGACGACGACGACGACGACGGCGCCg 151576
151551 gCTGCTGCTGCTGCTGCTGCCGCGGc 151576

ZFN-unknown-SP-6-649
151554 cGACGACGACGACGACGGCGCCGGCg 151579
151554 gCTGCTGCTGCTGCTGCCGCGGCCGc 151579

ZFN-unknown-SP-6-650
151557 cGACGACGACGACGGCGCCGGCGGTg 151582
151557 gCTGCTGCTGCTGCCGCGGCCGCCAc 151582

ZFN-unknown-SP-7-594
151560 cGACGACGACGGCGCCGGCGGTGGTGg 151586
151560 gCTGCTGCTGCCGCGGCCGCCACCACc 151586

ZFN-unknown-SP-6-651
151560 cGACGACGACGGCGCCGGCGGTGGTg 151585
151560 gCTGCTGCTGCCGCGGCCGCCACCAc 151585

ZFN-unknown-SP-6-652
151563 cGACGACGGCGCCGGCGGTGGTGGCg 151588
151563 gCTGCTGCCGCGGCCGCCACCACCGc 151588

ZFN-unknown-SP-7-595
151563 cGACGACGGCGCCGGCGGTGGTGGCGg 151589
151563 gCTGCTGCCGCGGCCGCCACCACCGCc 151589

ZFN-unknown-SP-6-653
151566 cGACGGCGCCGGCGGTGGTGGCGGCg 151591
151566 gCTGCCGCGGCCGCCACCACCGCCGc 151591

ZFN-unknown-SP-7-596
151566 cGACGGCGCCGGCGGTGGTGGCGGCGg 151592
151566 gCTGCCGCGGCCGCCACCACCGCCGCc 151592

ZFN-unknown-SP-7-597
151569 cGGCGCCGGCGGTGGTGGCGGCGGCCg 151595
151569 gCCGCGGCCGCCACCACCGCCGCCGGc 151595

ZFN-unknown-SP-6-654
151584 tGGCGGCGGCCGGCGCGCGGAGGCGg 151609
151584 aCCGCCGCCGGCCGCGCGCCTCCGCc 151609

ZFN-unknown-SP-6-655
151671 cTTCGACGGCGACCTGGCGGCCGTGc 151696
151671 gAAGCTGCCGCTGGACCGCCGGCACg 151696

ZFN-unknown-SP-7-598
151751 cGGCCGCCCCGCCGCACGCCGACGCGc 151777
151751 gCCGGCGGGGCGGCGTGCGGCTGCGCg 151777

ZFN-unknown-SP-6-656
151860 gGCCGGCGGCAGCGAGGCCGCCGTGg 151885
151860 cCGGCCGCCGTCGCTCCGGCGGCACc 151885

ZFN-unknown-SP-6-657
151863 cGGCGGCAGCGAGGCCGCCGTGGCCg 151888
151863 gCCGCCGTCGCTCCGGCGGCACCGGc 151888

ZFN-unknown-SP-6-658
151956 gAGCTCCGCCGCCGCCGCCGCCGCGg 151981
151956 cTCGAGGCGGCGGCGGCGGCGGCGCc 151981

ZFN-unknown-SP-7-599
151958 gCTCCGCCGCCGCCGCCGCCGCGGACc 151984
151958 cGAGGCGGCGGCGGCGGCGGCGCCTGg 151984

ZFN-unknown-SP-6-659
152019 gGCCGACACCGTCGCCGCGGCCGACt 152044
152019 cCGGCTGTGGCAGCGGCGCCGGCTGa 152044

```
ZFN-unknown-SP-6-660
152051 cCGCGCCCGCCTCCGCGCCGCGGGAg 152076
152051 gGCGCGGGCGGAGGCGCGGCGCCCTc 152076

ZFN-unknown-SP-6-661
152054 cGCCCGCCTCCGCGCCGCGGGAGGCg 152079
152054 gCGGGCGGAGGCGCGGCGCCCTCCGc 152079

ZFN-unknown-SP-5-542
152055 gCCCGCCTCCGCGCCGCGGGAGGCg 152079
152055 cGGGCGGAGGCGCGGCGCCCTCCGc 152079

ZFN-unknown-SP-6-662
152057 cCGCCTCCGCGCCGCGGGAGGCGCGc 152082
152057 gGCGGAGGCGCGGCGCCCTCCGCGg 152082

ZFN-unknown-SP-6-663
152093 gCCCCGCCCCGGCCAGGGCGCCGCCg 152118
152093 cGGGGCGGGGCCGGTCCCGCGGCGGc 152118

ZFN-unknown-SP-6-664
152096 cCGCCCCGGCCAGGGCGCCGCCGGGc 152121
152096 gGCGGGGCCGGTCCCGCGGCGGCCCg 152121

ZFN-unknown-SP-6-665
152154 gGACGCCCCCCGCCCCGCGGCCGCCc 152179
152154 cCTGCGGGGGGCGGGGCGCCGGCGGg 152179

ZFN-unknown-SP-7-600
152168 cCGCGGCCGCCCCTCCCGCGGGGGCCg 152194
152168 gGCGCCGGCGGGGAGGGCGCCCCCGGc 152194

ZFN-unknown-SP-7-601
152171 cGGCCGCCCCTCCCGCGGGGGCCGCGc 152197
152171 gCCGGCGGGGAGGGCGCCCCCGGCGCg 152197

ZFN-unknown-SP-6-666
152192 cCGCGCCCCCCGCCCCGCCGACGCCg 152217
152192 gGCGCGGGGGGCGGGGCGGCTGCGGc 152217

ZFN-unknown-SP-6-667
152195 cGCCCCCCGCCCCGCCGACGCCGCCg 152220
152195 gCGGGGGGCGGGGCGGCTGCGGCGGc 152220

ZFN-unknown-SP-5-543
152196 gCCCCCCGCCCCGCCGACGCCGCCg 152220
152196 cGGGGGGCGGGGCGGCTGCGGCGGc 152220

ZFN-unknown-SP-6-668
152198 cCCCCGCCCCGCCGACGCCGCCGCCg 152223
152198 gGGGGCGGGGCGGCTGCGGCGGCGGc 152223

ZFN-unknown-SP-6-669
152201 cCGCCCCGCCGACGCCGCCGCCGCGg 152226
152201 gGCGGGGCGGCTGCGGCGGCGGCGCc 152226

ZFN-unknown-SP-7-602
152203 gCCCCGCCGACGCCGCCGCCGCGGCCg 152229
152203 cGGGGCGGCTGCGGCGGCGGCGCCGGc 152229

ZFN-unknown-SP-6-670
152204 cCCCGCCGACGCCGCCGCCGCGGCCg 152229
152204 gGGGCGGCTGCGGCGGCGGCGCCGGc 152229

ZFN-unknown-SP-7-603
152209 cCGACGCCGCCGCCGCGGCCGCCGCGc 152235
```

152209 gGCTGCGGCGGCGGCGCCGGCGGCGCg 152235

ZFN-unknown-SP-7-604
152224 cGGCCGCCGCGCCCCGCGGCGCTGACc 152250
152224 gCCGGCGGCGCGGGGCGCCGCGACTGg 152250

ZFN-unknown-SP-7-605
152265 gGGCCCCGACCCGCAGGGCGGCTGGCg 152291
152265 cCCGGGGCTGGGCGTCCCGCCGACCGc 152291

ZFN-unknown-SP-7-606
152315 aCACGCCGGCGCCCTCGGCCGCCGCCc 152341
152315 tGTGCGGCCGCGGGAGCCGGCGGCGGg 152341

ZFN-unknown-SP-6-671
152349 cTACTGCGCCCCGCGGGCCGTGGCCg 152374
152349 gATGACGCGGGGCGCCCGGCACCGGc 152374

ZFN-unknown-SP-5-544
152518 gGCCCGCTGCGCCGCGCGGCGGCCt 152542
152518 cCGGGCGACGCGGCGCGCCGCCGGa 152542

ZFN-unknown-SP-7-607
152714 gGCCCGCCACGGCCGCCTGGGCGGGCa 152740
152714 cCGGGCGGTGCCGGCGGACCCGCCCGt 152740

ZFN-unknown-SP-6-672
152750 gCGCCCCCGACGTCTCGGCGCTGGGc 152775
152750 cGCGGGGGCTGCAGAGCCGCGACCCg 152775

ZFN-unknown-SP-5-545
152751 cGCCCCCGACGTCTCGGCGCTGGGc 152775
152751 gCGGGGGCTGCAGAGCCGCGACCCg 152775

ZFN-unknown-SP-6-673
152877 cGTCAACGCCGTGCGCGCCGCGGACt 152902
152877 gCAGTTGCGGCACGCGCGGCGCCTGa 152902

ZFN-unknown-SP-7-608
152891 gCGCCGCGGACTGGCCCGCCGACGGGc 152917
152891 cGCGGCGCCTGACCGGGCGGCTGCCCg 152917

ZFN-unknown-SP-7-609
152933 aGCACGCCTACCTGGCCTGCGAGGTGc 152959
152933 tCGTGCGGATGGACCGGACGCTCCACg 152959

ZFN-unknown-SP-6-674
152934 gCACGCCTACCTGGCCTGCGAGGTGc 152959
152934 cGTGCGGATGGACCGGACGCTCCACg 152959

ZFN-unknown-SP-5-546
153084 gTACCCCGACGCGCCGCCGCTGCGc 153108
153084 cATGGGGCTGCGCGGCGGCGACGCg 153108

ZFN-unknown-SP-6-675
153097 cCGCCGCTGCGCCTCTGCCGCGGGGc 153122
153097 gGCGGCGACGCGGAGACGGCGCCCCg 153122

ZFN-unknown-SP-5-547
153150 cTTCGGCCCCGACACGCTGGTGCCc 153174
153150 gAAGCCGGGGCTGTGCGACCACGGg 153174

ZFN-unknown-SP-7-610
153224 gGGCCGCCGCCTCGGGCGCGGGCGACg 153250
153224 cCCGGCGGCGGAGCCCGCGCCCGCTGc 153250

ZFN-unknown-SP-7-611
153297 gCACCGCGCCTGCGCGCGCTGGGGCCt 153323
153297 cGTGGCGCGGACGCGCGCGACCCCGGa 153323

ZFN-unknown-SP-7-612
153339 gCCCGTCTACGTGGCGCTGGGGCGCGa 153365
153339 cGGGCAGATGCACCGCGACCCCGCGCt 153365

ZFN-unknown-SP-5-548
153339 gCCCGTCTACGTGGCGCTGGGGCGc 153363
153339 cGGGCAGATGCACCGCGACCCCGCg 153363

ZFN-unknown-SP-6-676
153443 gCGACGCGCCCCCGCTGGTGCTGCGc 153468
153443 cGCTGCGCGGGGGCGACCACGACGCg 153468

ZFN-unknown-SP-6-677
153448 gCGCCCCCGCTGGTGCTGCGCGACGa 153473
153448 cGCGGGGGCGACCACGACGCGCTGCt 153473

ZFN-unknown-SP-7-613
153488 cGCCCCCGCAGATACGCTGGGCGTCGg 153514
153488 gCGGGGGCGTCTATGCGACCCGCAGCc 153514

ZFN-unknown-SP-7-614
153509 cGTCGGCCGCGGGCCGCGCGGGGACGg 153535
153509 gCAGCCGGCGCCCGGCGCGCCCCTGCc 153535

ZFN-unknown-SP-5-549
153549 gGGCGGCGGCGTGGAGGTGGTGGGg 153573
153549 cCCGCCGCCGCACCTCCACCACCCc 153573

ZFN-unknown-SP-6-678
153549 gGGCGGCGGCGTGGAGGTGGTGGGGa 153574
153549 cCCGCCGCCGCACCTCCACCACCCCt 153574

ZFN-unknown-SP-7-615
154070 gCACGTCTCCCGCGCCCGCGGGGGGTc 154096
154070 cGTGCAGAGGGCGCGGGCGCCCCCCAg 154096

ZFN-unknown-SP-6-679
154140 gAGCCCCCCCGCAGGAGCGGGAGGGa 154165
154140 cTCGGGGGGGCGTCCTCGCCCTCCCt 154165

ZFN-unknown-SP-7-616
154143 cCCCCCCGCAGGAGCGGGAGGGAAGGc 154169
154143 gGGGGGGCGTCCTCGCCCTCCCTTCCg 154169

ZFN-unknown-SP-6-680
154188 gGGCTGCTGCGAGCTCGGGGCCGCGg 154213
154188 cCCGACGACGCTCGAGCCCCGGCGCc 154213

ZFN-unknown-SP-7-617
154260 aGGCGGCCGCGGGGGAGGCGGCCGCGg 154286
154260 tCCGCCGGCGCCCCCTCCGCCGGCGCc 154286

ZFN-unknown-SP-7-618
154289 cCGCAGCCCCGTGGCGCGCGGGGGGGa 154315
154289 gGCGTCGGGGCACCGCGCGCCCCCCCt 154315

ZFN-unknown-SP-5-550
154433 tGGCGTCTTCGGGGGGGCGGGGAGc 154457
154433 aCCGCAGAAGCCCCCCCGCCCCTCg 154457

ZFN-unknown-SP-7-619

```
154493 aGTCCCCGTCCTGCCGCGCGGGGGCGg 154519
154493 tCAGGGGCAGGACGGCGCGCCCCCGCc 154519
```

ZFN-unknown-SP-6-681

```
154500 gTCCTGCCGCGCGGGGGCGGGCGCGg 154525
154500 cAGGACGGCGCGCCCCCGCCCGCGCc 154525
```

ZFN-unknown-SP-7-620

```
154556 aGGCAGCCCCGCGGCGCGCGGGGGGAg 154582
154556 tCCGTCGGGGCGCCGCGCGCCCCCCTc 154582
```

ZFN-unknown-SP-5-551

```
154561 gCCCCGCGGCGCGCGGGGGGAGGGg 154585
154561 cGGGGCGCCGCGCGCCCCCCTCCCc 154585
```

ZFN-unknown-SP-6-682

```
154561 gCCCCGCGGCGCGCGGGGGGAGGGGc 154586
154561 cGGGGCGCCGCGCGCCCCCCTCCCCg 154586
```

ZFN-unknown-SP-6-683

```
154583 gGGCGGCGCCCGCGGGGGAGCGGCCg 154608
154583 cCCGCCGCGGGCGCCCCCTCGCCGGc 154608
```

ZFN-unknown-SP-6-684

```
154586 cGGCGCCCGCGGGGGAGCGGCCGGCt 154611
154586 gCCGCGGGCGCCCCCTCGCCGGCCGa 154611
```

ZFN-unknown-SP-6-685

```
154647 gCCCTGCCGCCCGCCCGCCGCCGCCg 154672
154647 cGGGACGGCGGGCGGGCGGCGGCGGc 154672
```

ZFN-unknown-SP-5-552

```
154651 tGCCGCCCGCCCGCCGCCGCCGCCc 154675
154651 aCGGCGGGCGGGCGGCGGCGGCGGg 154675
```
