# Supplementary material for: Identity of zinc finger nucleases with specificity to herpes simplex virus type II genomic DNA: novel HSV-2 vaccine/therapy precursors
Source: Theor Biol Med Model. 2011 Jun 24;8:23. doi: 10.1186/1742-4682-8-23 (PMC3138452; doi:10.1186/1742-4682-8-23)
Supplement: Additional file 4 — A list of the -1 to 6 recognition domians of the alpha-helix for some of the ZFN cleaving within the 3,094.9 bp at the 5' end of the HSV-2 genome. This file lists details of the -1 to 6 recognition domians (denoted F1, F2, F3/F3, F2, F1) of the alpha-helix for some of the ZFN cleaving within the 3,094.9 bp at the 5' end of the HSV-2 genome. [file 1742-4682-8-23-S4.PDF]

Zinc Finger Site Type: Nuclease
Zinc Finger Engineering Method: CoDA
Sequence Name : HSV-2 Genomic
Sequence Length:154748
Nucleotide Sequence

Sort By: Position ☐ **Hide intron splice sites**

⊞ ZFN-unknown-SP-7-1
1 aGTCCCCGTCCTGCCGCGCGGGGGCGg 27
1 tCAGGGGCAGGACGGCGCGCCCCCGCc 27

FINGER	HELIX	TRIPLET	REFERENCE NUMBER	SOURCE
Left F1	EEANLRR	GAC	-	CoDA
Left F2	RREHLVR	GGG	-	CoDA
Left F3	DPSNLQR	GAC	-	CoDA
Right F1	KRHTLTR	GCG	-	CoDA
Right F2	RREHLVR	GGG	-	CoDA
Right F3	RTDSLPR	GCG	-	CoDA

ZF DNA Sequence Bos taurus (cow) Build 3.1

Blast GTCCCCGTCNNNNNNNGCGGGGGCG

⊞ ZFN-unknown-SP-6-1
8 gTCCTGCCGCGCGGGGGCGGGCGCGg 33
8 cAGGACGGCGCGCCCCCGCCCGCGCc 33

FINGER	HELIX	TRIPLET	REFERENCE NUMBER	SOURCE
Left F1	RTDRLIR	GGA	-	CoDA
Left F2	QSTTLKR	GCA	-	CoDA
Left F3	RLDMLAR	GCG	-	CoDA
Right F1	RRHGLDR	GCG	-	CoDA
Right F2	LKEHLTR	GGC	-	CoDA
Right F3	RKDGLTR	GCG	-	CoDA

ZF DNA Sequence Bos taurus (cow) Build 3.1

Blast TCCTGCCGCNNNNNNGCGGGCGCG

⊞ ZFN-unknown-SP-7-2
64 aGGCAGCCCCGCGGCGCGCGGGGGGAg 90
64 tCCGTCGGGGCGCCGCGCGCCCCCCTc 90

FINGER	HELIX	TRIPLET	REFERENCE NUMBER	SOURCE

Left F1	DRRTLDR	GCC	-	CoDA
Left F2	QRSDLTR	GCT	-	CoDA
Left F3	RTEHLAR	GGG	-	CoDA
Right F1	TSAHLAR	GGA	-	CoDA
Right F2	RREHLVR	GGG	-	CoDA
Right F3	RTDSLPR	GCG	-	CoDA

ZF DNA Sequence   Bos taurus (cow) Build 3.1

Blast GGCAGCCCCNNNNNNNGCGGGGGGA

ZFN-unknown-SP-5-1

69 gCCCCGCGGCGCGCGGGGGGAGGGg 93
69 cGGGGCGCCGCGCGCCCCCCTCCCc 93

FINGER	HELIX	TRIPLET	REFERENCE NUMBER	SOURCE
Left F1	RGNHLRR	GGG	-	CoDA
Left F2	RTDTLAR	GCG	-	CoDA
Left F3	ERRGLAR	GCC	-	CoDA
Right F1	KKDHLHR	GGG	-	CoDA
Right F2	QSAHLKR	GGA	-	CoDA
Right F3	RTEHLAR	GGG	-	CoDA

ZF DNA Sequence   Bos taurus (cow) Build 3.1

Blast CCCCGCGGCNNNNNNGGGGGAGGG

ZFN-unknown-SP-6-2

69 gCCCCGCGGCGCGCGGGGGAGGGGc 94
69 cGGGGCGCCGCGCGCCCCCCTCCCCg 94

FINGER	HELIX	TRIPLET	REFERENCE NUMBER	SOURCE
Left F1	RGNHLRR	GGG	-	CoDA
Left F2	RTDTLAR	GCG	-	CoDA
Left F3	ERRGLAR	GCC	-	CoDA
Right F1	RNTHLAR	GGG	-	CoDA
Right F2	RQDNLGR	GAG	-	CoDA
Right F3	RIDKLGG	GGG	-	CoDA

ZF DNA Sequence   Bos taurus (cow) Build 3.1

Blast CCCCGCGGCNNNNNNGGGGAGGGG

ZFN-unknown-SP-6-3

91 gGGCGGCGCCCGCGGGGGAGCGGCCg 116
91 cCCGCCGCGGGCGCCCCCTCGCCGGc 116

FINGER	HELIX	TRIPLET	REFERENCE NUMBER	SOURCE
Left F1	DGSTLRR	GCC	-	CoDA
Left F2	DSSVLRR	GCC	-	CoDA
Left F3	ENSKLNR	GGC	-	CoDA
Right F1	DPSTLRR	GCC	-	CoDA
Right F2	RTDTLAR	GCG	-	CoDA
Right F3	QTTHLSR	GGA	-	CoDA

ZF DNA Sequence Bos taurus (cow) Build 3.1

Blast GGCGGCGCCNNNNNNGGAGCGGCC

ZFN-unknown-SP-6-4
94 cGGCGCCCGCGGGGGAGCGGCCGGCt 119
94 gCCGCGGGCGCCCCCTCGCCGGCCGa 119

FINGER	HELIX	TRIPLET	REFERENCE NUMBER	SOURCE
Left F1	SNKDLTR	GCC	-	CoDA
Left F2	LKEHLTR	GGC	-	CoDA
Left F3	RKDGLTR	GCG	-	CoDA
Right F1	TPSKLDR	GGC	-	CoDA
Right F2	DSSVLRR	GCC	-	CoDA
Right F3	RVDDLGR	GCG	-	CoDA

ZF DNA Sequence Bos taurus (cow) Build 3.1

Blast GGCGCCCGCNNNNNNGCGGCCGGC

ZFN-unknown-SP-6-5
155 gCCCTGCCGCCCGCCCGCCGCCGCCg 180
155 cGGGACGGCGGGCGGGCGGCGGCGGc 180

FINGER	HELIX	TRIPLET	REFERENCE NUMBER	SOURCE
Left F1	RRAHLQN	GGG	-	CoDA
Left F2	QSTTLKR	GCA	-	CoDA
Left F3	RLDMLAR	GCG	-	CoDA
Right F1	DGSTLRR	GCC	-	CoDA
Right F2	DSSVLRR	GCC	-	CoDA
Right F3	ERRGLAR	GCC	-	CoDA

ZF DNA Sequence Bos taurus (cow) Build 3.1

Blast CCCTGCCGCNNNNNNGCCGCCGCC

ZFN-unknown-SP-5-2

159 tGCCGCCCGCCCGCCGCCGCCGCCc 183
159 aCGGCGGGCGGGCGGCGGCGGCGGg 183

FINGER	HELIX	TRIPLET	REFERENCE NUMBER	SOURCE
Left F1	SKHKLER	GGC	-	CoDA
Left F2	LKEHLTR	GGC	-	CoDA
Left F3	RKDGLTR	GCG	-	CoDA
Right F1	DGSTLRR	GCC	-	CoDA
Right F2	DSSVLRR	GCC	-	CoDA
Right F3	ERRGLAR	GCC	-	CoDA

ZF DNA Sequence

Bos taurus (cow) Build 3.1

Blast GCCGCCCGCNNNNNGCCGCCGCC

ZFN-unknown-SP-7-3
262 gTCCTGCCGCCGCCCCTTAAGAGGGCc 288
262 cAGGACGGCGGCGGGGAATTCTCCCGg 288

FINGER	HELIX	TRIPLET	REFERENCE NUMBER	SOURCE
Left F1	RTDRLIR	GGA	-	CoDA
Left F2	QSTTLKR	GCA	-	CoDA
Left F3	RLDMLAR	GCG	-	CoDA
Right F1	SPSKLVR	GGC	-	CoDA
Right F2	RQDNLGR	GAG	-	CoDA
Right F3	QGGNLTL	TAA	-	CoDA

ZF DNA Sequence

Bos taurus (cow) Build 3.1

Blast TCCTGCCGCNNNNNNNTAAGAGGGC

ZFN-unknown-SP-7-4
623 tCGCTCCTGCGGCGCTGGCTGCTGGTg 649
623 aGCGAGGACGCCGCGACCGACGACCAc 649

FINGER	HELIX	TRIPLET	REFERENCE NUMBER	SOURCE
Left F1	KNNDLTR	GCG	-	CoDA
Left F2	QSAHLKR	GGA	-	CoDA
Left F3	QDVSLVR	GCA	-	CoDA
Right F1	RRQKLTI	GGT	-	CoDA
Right F2	QRSDLTR	GCT	-	CoDA
Right F3	LRASLRR	GCT	-	CoDA

ZF DNA Sequence

Bos taurus (cow) Build 3.1

Blast CGCTCCTGCNNNNNNNGCTGCTGGT

ZFN-unknown-SP-7-5
626 cTCCTGCGGCGCTGGCTGCTGGTGCCc 652
626 gAGGACGCCGCGACCGACGACCACGGg 652

FINGER	HELIX	TRIPLET	REFERENCE NUMBER	SOURCE
Left F1	RTDRLIR	GGA	-	CoDA
Left F2	QSTTLKR	GCA	-	CoDA
Left F3	DPSNLRR	GCC	-	CoDA
Right F1	SKKSLTR	GCC	-	CoDA
Right F2	EAHHLSR	GGT	-	CoDA
Right F3	EGSGLKR	GCT	-	CoDA

ZF DNA Sequence

Bos taurus (cow) Build 3.1

Blast TCCTGCGGCNNNNNNNGCTGGTGCC

ZFN-unknown-SP-6-6
676 gGACTACGCCGGCAACGACGACGCAg 701
676 cCTGATGCGGCCGTTGCTGCTGCGTc 701

FINGER	HELIX	TRIPLET	REFERENCE NUMBER	SOURCE
Left F1	TSTILAR	GTC	-	CoDA
Left F2	QRSSLVR	GTA	-	CoDA
Left F3	ESGHLKR	GGC	-	CoDA
Right F1	LKHSLLR	GCA	-	CoDA
Right F2	DRGNLTR	GAC	-	CoDA
Right F3	DQGNLIR	GAC	-	CoDA

ZF DNA Sequence

Bos taurus (cow) Build 3.1

Blast GACTACGCCNNNNNNGACGACGCA

ZFN-unknown-SP-6-7
679 cTACGCCGGCAACGACGACGCAGAGt 704
679 gATGCGGCCGTTGCTGCTGCGTCTCa 704

FINGER	HELIX	TRIPLET	REFERENCE NUMBER	SOURCE
Left F1	QQSSLLR	GTA	-	CoDA
Left F2	LKEHLTR	GGC	-	CoDA
Left F3	DPSNLRR	GCC	-	CoDA
Right F1	KHSNLAR	GAG	-	CoDA
Right F2	QSTTLKR	GCA	-	CoDA
Right F3	DPSNLRR	GAC	-	CoDA

ZF DNA Sequence

Bos taurus (cow) Build 3.1

Blast TACGCCGGCNNNNNNGACGCAGAG

ZFN-unknown-SP-6-8
709 gAACAGCCCCCCGAGCGAGGGCGGGg 734
709 cTTGTCGGGGGGCTCGCTCCCGCCCc 734

FINGER	HELIX	TRIPLET	REFERENCE NUMBER	SOURCE
Left F1	QATLLRR	GTT	-	CoDA
Left F2	QRSDLTR	GCT	-	CoDA
Left F3	RTEHLAR	GGG	-	CoDA
Right F1	RKHHLGR	GGG	-	CoDA
Right F2	LKEHLTR	GGC	-	CoDA
Right F3	RVDNLPR	GAG	-	CoDA

ZF DNA Sequence

Bos taurus (cow) Build 3.1

Blast AACAGCCCCNNNNNNGAGGGCGGG

ZFN-unknown-SP-6-9
713 aGCCCCCCGAGCGAGGGCGGGGGGAa 738
713 tCGGGGGGCTCGCTCCCGCCCCCCTt 738

FINGER	HELIX	TRIPLET	REFERENCE NUMBER	SOURCE
Left F1	TNSKLTR	GGC	-	CoDA
Left F2	RREHLVR	GGG	-	CoDA
Left F3	RMDSLGG	TCG	-	CoDA
Right F1	TSAHLAR	GGA	-	CoDA
Right F2	RREHLVR	GGG	-	CoDA
Right F3	RTDSLPR	GCG	-	CoDA

ZF DNA Sequence

Bos taurus (cow) Build 3.1

Blast GCCCCCCGANNNNNNGCGGGGGGA

ZFN-unknown-SP-5-3
713 aGCCCCCCGAGCGAGGGCGGGGGGa 737
713 tCGGGGGGCTCGCTCCCGCCCCCCt 737

FINGER	HELIX	TRIPLET	REFERENCE NUMBER	SOURCE
Left F1	TNSKLTR	GGC	-	CoDA
Left F2	RREHLVR	GGG	-	CoDA
Left F3	RMDSLGG	TCG	-	CoDA
Right F1	RKHHLGR	GGG	-	CoDA
Right F2	RREHLVR	GGG	-	CoDA
Right F3	ESGHLKR	GGC	-	CoDA

[ZF DNA Sequence] Bos taurus (cow) Build 3.1

Blast GCCCCCCGANNNNNGGCGGGGGG

ZFN-unknown-SP-7-6

753 cGCACGCCGCGCCTGCCGCCGCCTGCc 779
753 gCGTGCGGCGCGGACGGCGGCGGACGg 779

FINGER	HELIX	TRIPLET	REFERENCE NUMBER	SOURCE
Left F1	RSRNLTL	TGC	-	CoDA
Left F2	RTDTLAR	GCG	-	CoDA
Left F3	RVDDLGR	GCG	-	CoDA
Right F1	RGRNLEM	TGC	-	CoDA
Right F2	DSSVLRR	GCC	-	CoDA
Right F3	ERRGLAR	GCC	-	CoDA

[ZF DNA Sequence] Bos taurus (cow) Build 3.1

Blast GCACGCCGCNNNNNNGCCGCCTGC

ZFN-unknown-SP-5-4

769 cGCCGCCTGCCCCCCGCCGCCGCCg 793
769 gCGGCGGACGGGGGGCGGCGGCGGc 793

FINGER	HELIX	TRIPLET	REFERENCE NUMBER	SOURCE
Left F1	SKHKLER	GGC	-	CoDA
Left F2	LKEHLTR	GGC	-	CoDA
Left F3	QTNTLKR	GCA	-	CoDA
Right F1	DGSTLRR	GCC	-	CoDA
Right F2	DSSVLRR	GCC	-	CoDA
Right F3	ERRGLAR	GCC	-	CoDA

[ZF DNA Sequence] Bos taurus (cow) Build 3.1

Blast GCCGCCTGCNNNNNGCCGCCGCC

ZFN-unknown-SP-5-5

772 cGCCTGCCCCCCGCCGCCGCCGCGc 796
772 gCGGACGGGGGGCGGCGGCGGCGCg 796

FINGER	HELIX	TRIPLET	REFERENCE NUMBER	SOURCE
Left F1	VPSKLLR	GGC	-	CoDA
Left F2	QSTTLKR	GCA	-	CoDA
Left F3	RTEHLAR	GGG	-	CoDA
Right F1	RLRDLPR	GCG	-	CoDA

Right F2	DSSVLRR	GCC	-	CoDA
Right F3	ERRGLAR	GCC	-	CoDA

ZF DNA Sequence Bos taurus (cow) Build 3.1

Blast GCCTGCCCCNNNNNGCCGCCGCG

ZFN-unknown-SP-7-7
839 cTACGGCTGCGCACCACGACGGAGTAc 865
839 gATGCCGACGCGTGGTGCTGCCTCATg 865

FINGER	HELIX	TRIPLET	REFERENCE NUMBER	SOURCE
Left F1	QKQALTR	GTA	-	CoDA
Left F2	DSSVLRR	GCC	-	CoDA
Left F3	QGGTLRR	GCA	-	CoDA
Right F1	QQQALTR	GTA	-	CoDA
Right F2	QSAHLKR	GGA	-	CoDA
Right F3	DPSNLRR	GAC	-	CoDA

ZF DNA Sequence Bos taurus (cow) Build 3.1

Blast TACGGCTGCNNNNNNNGACGGAGTA

ZFN-unknown-SP-7-8
897 cCCCCGCGTCCCCGCCCGCGGACGCGc 923
897 gGGGGCGCAGGGGCGGGCGCCTGCGCg 923

FINGER	HELIX	TRIPLET	REFERENCE NUMBER	SOURCE
Left F1	RGNHLRR	GGG	-	CoDA
Left F2	RTDTLAR	GCG	-	CoDA
Left F3	DPSNLRR	GAC	-	CoDA
Right F1	KHDTLHR	GCG	-	CoDA
Right F2	DRGNLTR	GAC	-	CoDA
Right F3	RRDGLTR	GCG	-	CoDA

ZF DNA Sequence Bos taurus (cow) Build 3.1

Blast CCCCGCGTCNNNNNNNGCGGACGCG

ZFN-unknown-SP-6-10
1069 cCGTGTCTCCCCGCCCGCAGGTGTGc 1094
1069 gGCACAGAGGGGCGGGCGTCCACACg 1094

FINGER	HELIX	TRIPLET	REFERENCE NUMBER	SOURCE
Left F1	KGNDLTR	ACG	-	CoDA
Left F2	DRGNLTR	GAC	-	CoDA

Left F3	QGGHLKR	GGA	-	CoDA
Right F1	RRFILSR	GTG	-	CoDA
Right F2	EAHHLSR	GGT	-	CoDA
Right F3	QNGTLTR	GCA	-	CoDA

ZF DNA Sequence Bos taurus (cow) Build 3.1

Blast CGTGTCTCCNNNNNNGCAGGTGTG

ZFN-unknown-SP-7-9
1184 cGACCGCGACCGGTTCCGGCGCCGCGt 1210
1184 gCTGGCGCTGGCCAAGGCCGCGGCGCa 1210

FINGER	HELIX	TRIPLET	REFERENCE NUMBER	SOURCE
Left F1	TSTLLNR	GTC	-	CoDA
Left F2	RTDTLAR	GCG	-	CoDA
Left F3	ESGALRR	GTC	-	CoDA
Right F1	RLRDLPR	GCG	-	CoDA
Right F2	DSSVLRR	GCC	-	CoDA
Right F3	ENSKLNR	GGC	-	CoDA

ZF DNA Sequence Bos taurus (cow) Build 3.1

Blast GACCGCGACNNNNNNNGGCGCCGCG

ZFN-unknown-SP-5-6
1197 tTCCGGCGCCGCGTGGCGGCGGCCg 1221
1197 aAGGCCGCGGCGCACCGCCGCCGGc 1221

FINGER	HELIX	TRIPLET	REFERENCE NUMBER	SOURCE
Left F1	DNAHLAR	GGA	-	CoDA
Left F2	DSSVLRR	GCC	-	CoDA
Left F3	ENSKLNR	GGC	-	CoDA
Right F1	DPSTLRR	GCC	-	CoDA
Right F2	RTDTLAR	GCG	-	CoDA
Right F3	RVDDLGR	GCG	-	CoDA

ZF DNA Sequence Bos taurus (cow) Build 3.1

Blast TCCGGCGCCNNNNNGCGGCGGCC

ZFN-unknown-SP-7-10
1300 gACCCGCGGAGGAGGAGGAGGCGGCGg 1326
1300 cTGGGCGCCTCCTCCTCCTCCGCCGCc 1326

FINGER	HELIX	TRIPLET	REFERENCE NUMBER	SOURCE

Left F1	TKQKLQT	GGT	-	CoDA
Left F2	RTDTLAR	GCG	-	CoDA
Left F3	DKRSLPH	TCC	-	CoDA
Right F1	RSNTLLR	GCG	-	CoDA
Right F2	RTDTLAR	GCG	-	CoDA
Right F3	RRDNLLR	GAG	-	CoDA

ZF DNA Sequence
Bos taurus (cow) Build 3.1

Blast ACCCGCGGANNNNNNNGAGGCGGCG

ZFN-unknown-SP-6-11
1300 gACCCGCGGAGGAGGAGGAGGCGGCg 1325
1300 cTGGGCGCCTCCTCCTCCTCCGCCGc 1325

FINGER	HELIX	TRIPLET	REFERENCE NUMBER	SOURCE
Left F1	TKQKLQT	GGT	-	CoDA
Left F2	RTDTLAR	GCG	-	CoDA
Left F3	DKRSLPH	TCC	-	CoDA
Right F1	SKHKLER	GGC	-	CoDA
Right F2	LKEHLTR	GGC	-	CoDA
Right F3	QSQHLVR	GGA	-	CoDA

ZF DNA Sequence
Bos taurus (cow) Build 3.1

Blast ACCCGCGGANNNNNNGGAGGCGGC

ZFN-unknown-SP-7-11
1506 cCGCTCCGCCCGCCCCAGGGGGCGGCg 1532
1506 gGCGAGGCGGGCGGGGTCCCCCGCCGc 1532

FINGER	HELIX	TRIPLET	REFERENCE NUMBER	SOURCE
Left F1	KNNDLTR	GCG	-	CoDA
Left F2	QSAHLKR	GGA	-	CoDA
Left F3	EKSHLKR	GGC	-	CoDA
Right F1	SKHKLER	GGC	-	CoDA
Right F2	LKEHLTR	GGC	-	CoDA
Right F3	RGDKLAL	GGG	-	CoDA

ZF DNA Sequence
Bos taurus (cow) Build 3.1

Blast CGCTCCGCCNNNNNNNGGGGGCGGC

ZFN-unknown-SP-5-7
1509 cTCCGCCCGCCCCAGGGGGCGGCGc 1533
1509 gAGGCGGGCGGGGTCCCCCGCCGCg 1533

FINGER	HELIX	TRIPLET	REFERENCE NUMBER	SOURCE
Left F1	RPSKLVL	GGA	-	CoDA
Left F2	LKEHLTR	GGC	-	CoDA
Left F3	RKDGLTR	GCG	-	CoDA
Right F1	RSNTLLR	GCG	-	CoDA
Right F2	RTDTLAR	GCG	-	CoDA
Right F3	RKHRLDG	GGG	-	CoDA

ZF DNA Sequence Bos taurus (cow) Build 3.1

Blast TCCGCCCGCNNNNNGGGGCGGCG

ZFN-unknown-SP-7-12
1512 cGCCCGCCCCAGGGGGCGGCGCCGGCc 1538
1512 gCGGGCGGGGTCCCCCGCCGCGGCCGg 1538

FINGER	HELIX	TRIPLET	REFERENCE NUMBER	SOURCE
Left F1	VPSKLAR	GGC	-	CoDA
Left F2	RTDTLAR	GCG	-	CoDA
Left F3	RKHRLDG	GGG	-	CoDA
Right F1	TPSKLDR	GGC	-	CoDA
Right F2	DSSVLRR	GCC	-	CoDA
Right F3	ENSKLNR	GGC	-	CoDA

ZF DNA Sequence Bos taurus (cow) Build 3.1

Blast GCCCGCCCCNNNNNNNGGCGCCGGC

ZFN-unknown-SP-6-12
1513 gCCCGCCCCAGGGGGCGGCGCCGGCc 1538
1513 cGGGCGGGGTCCCCCGCCGCGGCCGg 1538

FINGER	HELIX	TRIPLET	REFERENCE NUMBER	SOURCE
Left F1	RKHHLGR	GGG	-	CoDA
Left F2	LKEHLTR	GGC	-	CoDA
Left F3	RTESLHI	TGG	-	CoDA
Right F1	TPSKLDR	GGC	-	CoDA
Right F2	DSSVLRR	GCC	-	CoDA
Right F3	ENSKLNR	GGC	-	CoDA

ZF DNA Sequence Bos taurus (cow) Build 3.1

Blast CCCGCCCCANNNNNNGGCGCCGGC

ZFN-unknown-SP-5-8

1931 gGGCCGCGCCGGCGGGGCGCCGGGg 1955
1931 cCCGGCGCGGCCGCCCCGCGGCCCc 1955

FINGER	HELIX	TRIPLET	REFERENCE NUMBER	SOURCE
Left F1	DPSTLRR	GCC	-	CoDA
Left F2	RTDTLAR	GCG	-	CoDA
Left F3	ESGHLKR	GGC	-	CoDA
Right F1	KGDHLRR	GGG	-	CoDA
Right F2	DSSVLRR	GCC	-	CoDA
Right F3	ENSKLNR	GGC	-	CoDA

ZF DNA Sequence

Bos taurus (cow) Build 3.1

Blast GGCCGCGCCNNNNNGGCGCCGGG

ZFN-unknown-SP-5-9
1934 cCGCGCCGGCGGGGCGCCGGGGGGa 1958
1934 gGCGCGGCCGCCCCGCGGCCCCCCt 1958

FINGER	HELIX	TRIPLET	REFERENCE NUMBER	SOURCE
Left F1	RRHGLDR	GCG	-	CoDA
Left F2	LKEHLTR	GGC	-	CoDA
Left F3	DPSNLRR	GCC	-	CoDA
Right F1	RKHHLGR	GGG	-	CoDA
Right F2	RREHLVR	GGG	-	CoDA
Right F3	EHRGLKR	GCC	-	CoDA

ZF DNA Sequence

Bos taurus (cow) Build 3.1

Blast CGCGCCGGCNNNNNGCCGGGGGG

ZFN-unknown-SP-7-13
1960 tCCCATCTGCGTCGGCGGGGGGCGGCg 1986
1960 aGGGTAGACGCAGCCGCCCCCCGCCGc 1986

FINGER	HELIX	TRIPLET	REFERENCE NUMBER	SOURCE
Left F1	KKDHLHR	GGG	-	CoDA
Left F2	VRHNLTR	GAT	-	CoDA
Left F3	QGNTLTR	GCA	-	CoDA
Right F1	SKHKLER	GGC	-	CoDA
Right F2	LKEHLTR	GGC	-	CoDA
Right F3	RGDKLAL	GGG	-	CoDA

ZF DNA Sequence

Bos taurus (cow) Build 3.1

Blast CCCATCTGCNNNNNNNGGGGGCGGC

ZFN-unknown-SP-5-10
1963 cATCTGCGTCGGCGGGGGCGGCGc 1987
1963 gTAGACGCAGCCGCCCCCGCCGCg 1987

FINGER	HELIX	TRIPLET	REFERENCE NUMBER	SOURCE
Left F1	SKQALAV	GAT	-	CoDA
Left F2	QSTTLKR	GCA	-	CoDA
Left F3	DPSNLRR	GAC	-	CoDA
Right F1	RSNTLLR	GCG	-	CoDA
Right F2	RTDTLAR	GCG	-	CoDA
Right F3	RKHRLDG	GGG	-	CoDA

ZF DNA Sequence Bos taurus (cow) Build 3.1

Blast ATCTGCGTCNNNNNGGGGCGGCG

ZFN-unknown-SP-7-14
1966 cTGCGTCGGCGGGGGGCGGCGCATGCt 1992
1966 gACGCAGCCGCCCCCCGCCGCGTACGa 1992

FINGER	HELIX	TRIPLET	REFERENCE NUMBER	SOURCE
Left F1	LKHSLLR	GCA	-	CoDA
Left F2	DRGNLTR	GAC	-	CoDA
Left F3	DKSVLAR	GCC	-	CoDA
Right F1	RNRNLVL	TGC	-	CoDA
Right F2	QSTTLKR	GCA	-	CoDA
Right F3	DGGHLTR	GGC	-	CoDA

ZF DNA Sequence Bos taurus (cow) Build 3.1

Blast TGCGTCGGCNNNNNNNGGCGCATGC

ZFN-unknown-SP-6-13
2034 gGGCCGCCCCGAGAGGGGGGGATTCc 2059
2034 cCCGGCGGGGCTCTCCCCCCCTAAGg 2059

FINGER	HELIX	TRIPLET	REFERENCE NUMBER	SOURCE
Left F1	DPSTLRR	GCC	-	CoDA
Left F2	RTDTLAR	GCG	-	CoDA
Left F3	RKHRLDG	GGG	-	CoDA
Right F1	RANHLTI	TTC	-	CoDA
Right F2	QSAHLKR	GGA	-	CoDA
Right F3	RTEHLAR	GGG	-	CoDA

ZF DNA Sequence Bos taurus (cow) Build 3.1

Blast GGCCGCCCCNNNNNNGGGGGATTC

ZFN-unknown-SP-7-15
2057 tCCCTCCCTCCGCCCCCGCCGGGGCGc 2083
2057 aGGGAGGGAGGCGGGGGCGGCCCCGCg 2083

FINGER	HELIX	TRIPLET	REFERENCE NUMBER	SOURCE
Left F1	KKDHLHR	GGG	-	CoDA
Left F2	QSAHLKR	GGA	-	CoDA
Left F3	VHWNLMR	GAG	-	CoDA
Right F1	KRHTLTR	GCG	-	CoDA
Right F2	RREHLVR	GGG	-	CoDA
Right F3	EHRGLKR	GCC	-	CoDA

ZF DNA Sequence Bos taurus (cow) Build 3.1

Blast CCCTCCCTCNNNNNNNGCCGGGGCG

ZFN-unknown-SP-6-14
2200 gAGCGGCGGCCCGGCGGGAGGAGGAg 2225
2200 cTCGCCGCCGGGCCGCCCTCCTCCTc 2225

FINGER	HELIX	TRIPLET	REFERENCE NUMBER	SOURCE
Left F1	LRTSLVR	GCT	-	CoDA
Left F2	DSSVLRR	GCC	-	CoDA
Left F3	ERRGLAR	GCC	-	CoDA
Right F1	RTDRLIR	GGA	-	CoDA
Right F2	QSAHLKR	GGA	-	CoDA
Right F3	QMSHLKR	GGA	-	CoDA

ZF DNA Sequence Bos taurus (cow) Build 3.1

Blast AGCGGCGGCNNNNNNGGAGGAGGA

ZFN-unknown-SP-7-16
2200 gAGCGGCGGCCCGGCGGGAGGAGGAGa 2226
2200 cTCGCCGCCGGGCCGCCCTCCTCCTCt 2226

FINGER	HELIX	TRIPLET	REFERENCE NUMBER	SOURCE
Left F1	LRTSLVR	GCT	-	CoDA
Left F2	DSSVLRR	GCC	-	CoDA
Left F3	ERRGLAR	GCC	-	CoDA
Right F1	RQMNLDR	GAG	-	CoDA
Right F2	RQDNLGR	GAG	-	CoDA
Right F3	RVDNLPR	GAG	-	CoDA

ZF DNA Sequence — Bos taurus (cow) Build 3.1

Blast AGCGGCGGCNNNNNNNGAGGAGGAG

ZFN-unknown-SP-5-11
2335 gGACCCCGGCCCCGAGCGGCCGCCg 2359
2335 cCTGGGGCCGGGGCTCGCCGGCGGc 2359

FINGER	HELIX	TRIPLET	REFERENCE NUMBER	SOURCE
Left F1	TKSLLAR	GTC	-	CoDA
Left F2	RREHLVR	GGG	-	CoDA
Left F3	EHRGLKR	GCC	-	CoDA
Right F1	DGSTLRR	GCC	-	CoDA
Right F2	DSSVLRR	GCC	-	CoDA
Right F3	RVDDLGR	GCG	-	CoDA

ZF DNA Sequence — Bos taurus (cow) Build 3.1

Blast GACCCCGGCNNNNNGCGGCCGCC

ZFN-unknown-SP-5-12
2338 cCCCGGCCCCGAGCGGCCGCCGCGg 2362
2338 gGGGCCGGGGCTCGCCGGCGGCGCc 2362

FINGER	HELIX	TRIPLET	REFERENCE NUMBER	SOURCE
Left F1	KGDHLRR	GGG	-	CoDA
Left F2	DSSVLRR	GCC	-	CoDA
Left F3	RTEHLAR	GGG	-	CoDA
Right F1	RLRDLPR	GCG	-	CoDA
Right F2	DSSVLRR	GCC	-	CoDA
Right F3	ERRGLAR	GCC	-	CoDA

ZF DNA Sequence — Bos taurus (cow) Build 3.1

Blast CCCGGCCCCNNNNNGCCGCCGCG

ZFN-unknown-SP-7-17
2365 gACCCCCGGCACGGTGAGAGGGCGACc 2391
2365 cTGGGGGCCGTGCCACTCTCCCGCTGg 2391

FINGER	HELIX	TRIPLET	REFERENCE NUMBER	SOURCE
Left F1	RQSRLQR	GGT	-	CoDA
Left F2	RREHLVR	GGG	-	CoDA
Left F3	EHRGLKR	GCC	-	CoDA
Right F1	DPSNLRR	GAC	-	CoDA

Right F2	LKEHLTR	GGC	-	CoDA
Right F3	RVDNLPR	GAG	-	CoDA

ZF DNA Sequence Bos taurus (cow) Build 3.1

Blast ACCCCCGGCNNNNNNNGAGGGCGAC

ZFN-unknown-SP-6-15
2425 aCCCGGCTGCGGGTTGGGGGTGGTCg 2450
2425 tGGGCCGACGCCCAACCCCCACCAGc 2450

FINGER	HELIX	TRIPLET	REFERENCE NUMBER	SOURCE
Left F1	KGDHLRR	GGG	-	CoDA
Left F2	DSSVLRR	GCC	-	CoDA
Left F3	QGGTLRR	GCA	-	CoDA
Right F1	TMAVLRR	GTC	-	CoDA
Right F2	RREVLEN	GTG	-	CoDA
Right F3	RQGHLKR	GGG	-	CoDA

ZF DNA Sequence Bos taurus (cow) Build 3.1

Blast CCCGGCTGCNNNNNNGGGGTGGTC

ZFN-unknown-SP-5-13
2425 aCCCGGCTGCGGGTTGGGGGTGGTc 2449
2425 tGGGCCGACGCCCAACCCCCACCAg 2449

FINGER	HELIX	TRIPLET	REFERENCE NUMBER	SOURCE
Left F1	KGDHLRR	GGG	-	CoDA
Left F2	DSSVLRR	GCC	-	CoDA
Left F3	QGGTLRR	GCA	-	CoDA
Right F1	MKHHLAR	GGT	-	CoDA
Right F2	EAHHLSR	GGT	-	CoDA
Right F3	RTEHLAR	GGG	-	CoDA

ZF DNA Sequence Bos taurus (cow) Build 3.1

Blast CCCGGCTGCNNNNNGGGGGTGGT

ZFN-unknown-SP-6-16
2538 cGCCCCCCGACCCTCTGGGGGGGCGa 2563
2538 gCGGGGGGCTGGGAGACCCCCCCGCt 2563

FINGER	HELIX	TRIPLET	REFERENCE NUMBER	SOURCE
Left F1	TNSKLTR	GGC	-	CoDA
Left F2	RREHLVR	GGG	-	CoDA

Left F3	RMDSLGG	TCG	-	CoDA
Right F1	KRHTLTR	GCG	-	CoDA
Right F2	RREHLVR	GGG	-	CoDA
Right F3	RNDKLVP	GGG	-	CoDA

ZF DNA Sequence

Bos taurus (cow) Build 3.1

Blast GCCCCCCGANNNNNNGGGGGGGCG

ZFN-unknown-SP-5-14

2538 cGCCCCCCGACCCTCTGGGGGGGCg 2562
2538 gCGGGGGGCTGGGAGACCCCCCCGc 2562

FINGER	HELIX	TRIPLET	REFERENCE NUMBER	SOURCE
Left F1	TNSKLTR	GGC	-	CoDA
Left F2	RREHLVR	GGG	-	CoDA
Left F3	RMDSLGG	TCG	-	CoDA
Right F1	TNSKLTR	GGC	-	CoDA
Right F2	RREHLVR	GGG	-	CoDA
Right F3	RMDHLAG	TGG	-	CoDA

ZF DNA Sequence

Bos taurus (cow) Build 3.1

Blast GCCCCCCGANNNNNTGGGGGGGC

ZFN-unknown-SP-5-15

2539 gCCCCCCGACCCTCTGGGGGGGCGa 2563
2539 cGGGGGGCTGGGAGACCCCCCCGCt 2563

FINGER	HELIX	TRIPLET	REFERENCE NUMBER	SOURCE
Left F1	RKHHLGR	GGG	-	CoDA
Left F2	RREHLVR	GGG	-	CoDA
Left F3	DPTSLNR	GTC	-	CoDA
Right F1	KRHTLTR	GCG	-	CoDA
Right F2	RREHLVR	GGG	-	CoDA
Right F3	RNDKLVP	GGG	-	CoDA

ZF DNA Sequence

Bos taurus (cow) Build 3.1

Blast CCCCCCGACNNNNNGGGGGGGCG

ZFN-unknown-SP-7-18

2664 cCCCTTCCGAACAAAAAGCAGCGGGCg 2690
2664 gGGGAAGGCTTGTTTTTCGTCGCCCGc 2690

FINGER	HELIX	TRIPLET	REFERENCE NUMBER	SOURCE

Left F1	KRERLDR	GGG	–	CoDA
Left F2	QQTNLTR	GAA	–	CoDA
Left F3	RRDGLSG	TCG	–	CoDA
Right F1	VPSKLAR	GGC	–	CoDA
Right F2	RTDTLAR	GCG	–	CoDA
Right F3	QKGTLGR	GCA	–	CoDA

ZF DNA Sequence

Bos taurus (cow) Build 3.1

Blast CCCTTCCGANNNNNNNGCAGCGGGC

ZFN-unknown-SP-5-16

2792 cCGCCCCGCACGCCTGGGGGATGCt 2816

2792 gGCGGGGCGTGCGGACCCCCTACGa 2816

FINGER	HELIX	TRIPLET	REFERENCE NUMBER	SOURCE
Left F1	KRHTLTR	GCG	–	CoDA
Left F2	RREHLVR	GGG	–	CoDA
Left F3	ANRTLVH	TGC	–	CoDA
Right F1	RSRNLLL	TGC	–	CoDA
Right F2	QSAHLKR	GGA	–	CoDA
Right F3	RTEHLAR	GGG	–	CoDA

ZF DNA Sequence

Bos taurus (cow) Build 3.1

Blast CGCCCCGCANNNNNGGGGGATGC

ZFN-unknown-SP-6-17

2792 cCGCCCCGCACGCCTGGGGGATGCTc 2817

2792 gGCGGGGCGTGCGGACCCCCTACGAg 2817

FINGER	HELIX	TRIPLET	REFERENCE NUMBER	SOURCE
Left F1	KRHTLTR	GCG	–	CoDA
Left F2	RREHLVR	GGG	–	CoDA
Left F3	ANRTLVH	TGC	–	CoDA
Right F1	TKQVLDR	GCT	–	CoDA
Right F2	VRHNLTR	GAT	–	CoDA
Right F3	RGDKLGP	GGG	–	CoDA

ZF DNA Sequence

Bos taurus (cow) Build 3.1

Blast CGCCCCGCANNNNNNGGGGATGCT

ZFN-unknown-SP-6-18

2835 cGCCAGCAGCGACTCGGAGGAGGAGa 2860

2835 gCGGTCGTCGCTGAGCCTCCTCCTCt 2860

FINGER	HELIX	TRIPLET	REFERENCE NUMBER	SOURCE
Left F1	APSKLAR	GGC	-	CoDA
Left F2	QRSDLTR	GCT	-	CoDA
Left F3	LRASLRR	GCT	-	CoDA
Right F1	RQMNLDR	GAG	-	CoDA
Right F2	RQDNLGR	GAG	-	CoDA
Right F3	RVDNLPR	GAG	-	CoDA

ZF DNA Sequence Bos taurus (cow) Build 3.1

Blast GCCAGCAGCNNNNNNGAGGAGGAG

ZFN-unknown-SP-5-17
2835 cGCCAGCAGCGACTCGGAGGAGGAg 2859
2835 gCGGTCGTCGCTGAGCCTCCTCCTc 2859

FINGER	HELIX	TRIPLET	REFERENCE NUMBER	SOURCE
Left F1	APSKLAR	GGC	-	CoDA
Left F2	QRSDLTR	GCT	-	CoDA
Left F3	LRASLRR	GCT	-	CoDA
Right F1	RTDRLIR	GGA	-	CoDA
Right F2	QSAHLKR	GGA	-	CoDA
Right F3	QMSHLKR	GGA	-	CoDA

ZF DNA Sequence Bos taurus (cow) Build 3.1

Blast GCCAGCAGCNNNNNGGAGGAGGA

ZFN-unknown-SP-5-18
2838 cAGCAGCGACTCGGAGGAGGAGACc 2862
2838 gTCGTCGCTGAGCCTCCTCCTCTGg 2862

FINGER	HELIX	TRIPLET	REFERENCE NUMBER	SOURCE
Left F1	MKNTLTR	GCT	-	CoDA
Left F2	QRSDLTR	GCT	-	CoDA
Left F3	DRSSLRR	GTC	-	CoDA
Right F1	DEANLRR	GAC	-	CoDA
Right F2	QSAHLKR	GGA	-	CoDA
Right F3	QMSHLKR	GGA	-	CoDA

ZF DNA Sequence Bos taurus (cow) Build 3.1

Blast AGCAGCGACNNNNNGGAGGAGAC

ZFN-unknown-SP-6-19

2892 cCGCGACTCCACCTCCGAGGCGGGCa 2917
2892 gGCGCTGAGGTGGAGGCTCCGCCCGt 2917

FINGER	HELIX	TRIPLET	REFERENCE NUMBER	SOURCE
Left F1	RRHGLDR	GCG	-	CoDA
Left F2	DHSSLKR	GTC	-	CoDA
Left F3	QTTHLSR	GGA	-	CoDA
Right F1	VPSKLAR	GGC	-	CoDA
Right F2	RTDTLAR	GCG	-	CoDA
Right F3	RRDNLLR	GAG	-	CoDA

ZF DNA Sequence   Bos taurus (cow) Build 3.1

Blast CGCGACTCCNNNNNNGAGGCGGGC

ZFN-unknown-SP-6-20
2994 gGGCAGCCCCACGCCCGCCGACGCGc 3019
2994 cCCGTCGGGGTGCGGGCGGCTGCGCg 3019

FINGER	HELIX	TRIPLET	REFERENCE NUMBER	SOURCE
Left F1	DRRTLDR	GCC	-	CoDA
Left F2	QRSDLTR	GCT	-	CoDA
Left F3	RTEHLAR	GGG	-	CoDA
Right F1	KHDTLHR	GCG	-	CoDA
Right F2	DRGNLTR	GAC	-	CoDA
Right F3	DKSVLAR	GCC	-	CoDA

ZF DNA Sequence   Bos taurus (cow) Build 3.1

Blast GGCAGCCCCNNNNNNGCCGACGCG

ZFN-unknown-SP-7-19
3011 cCGACGCGCAGGGATCCTGTGGGGGTg 3037
3011 gGCTGCGCGTCCCTAGGACACCCCCAc 3037

FINGER	HELIX	TRIPLET	REFERENCE NUMBER	SOURCE
Left F1	KNNDLLK	TCG	-	CoDA
Left F2	RTDTLAR	GCG	-	CoDA
Left F3	QRRSLGH	TGC	-	CoDA
Right F1	RQSRLQR	GGT	-	CoDA
Right F2	RREHLVR	GGG	-	CoDA
Right F3	QRHGLSS	TGT	-	CoDA

ZF DNA Sequence   Bos taurus (cow) Build 3.1

Blast CGACGCGCANNNNNNTGTGGGGGT

ZFN-unknown-SP-6-21

3674 gCGCCCCGGCGCGGGGGCGGCGGTGc 3699
3674 cGCGGGGCCGCGCCCCCGCCGCCACg 3699

FINGER	HELIX	TRIPLET	REFERENCE NUMBER	SOURCE
Left F1	KRHTLTR	GCG	-	CoDA
Left F2	RREHLVR	GGG	-	CoDA
Left F3	EHRGLKR	GCC	-	CoDA
Right F1	RNFILAR	GTG	-	CoDA
Right F2	RTDTLAR	GCG	-	CoDA
Right F3	RVDDLGR	GCG	-	CoDA

ZF DNA Sequence Bos taurus (cow) Build 3.1

Blast CGCCCCGGCNNNNNNGCGGCGGTG

ZFN-unknown-SP-6-22

3755 gCCCCGCGGAGCAGCAGCAGCGGCGg 3780
3755 cGGGGCGCCTCGTCGTCGTCGCCGCc 3780

FINGER	HELIX	TRIPLET	REFERENCE NUMBER	SOURCE
Left F1	RGNHLRR	GGG	-	CoDA
Left F2	RTDTLAR	GCG	-	CoDA
Left F3	DKRSLPH	TCC	-	CoDA
Right F1	RSNTLLR	GCG	-	CoDA
Right F2	RTDTLAR	GCG	-	CoDA
Right F3	QKGTLGR	GCA	-	CoDA

ZF DNA Sequence Bos taurus (cow) Build 3.1

Blast CCCCGCGGANNNNNNGCAGCGGCG

ZFN-unknown-SP-5-19

3772 cAGCGGCGGCGCCCCGTTGCGGGCg 3796
3772 gTCGCCGCCGCGGGGCAACGCCCGc 3796

FINGER	HELIX	TRIPLET	REFERENCE NUMBER	SOURCE
Left F1	LRTSLVR	GCT	-	CoDA
Left F2	DSSVLRR	GCC	-	CoDA
Left F3	ERRGLAR	GCC	-	CoDA
Right F1	VPSKLAR	GGC	-	CoDA
Right F2	RTDTLAR	GCG	-	CoDA
Right F3	IRTSLKR	GTT	-	CoDA

ZF DNA Sequence Bos taurus (cow) Build 3.1

Blast AGCGGCGGCNNNNNGTTGCGGGC

ZFN-unknown-SP-5-20
3775 cGGCGGCGCCCCGTTGCGGGCGGGg 3799
3775 gCCGCCGCGGGGCAACGCCCGCCCc 3799

FINGER	HELIX	TRIPLET	REFERENCE NUMBER	SOURCE
Left F1	DGSTLRR	GCC	-	CoDA
Left F2	DSSVLRR	GCC	-	CoDA
Left F3	ENSKLNR	GGC	-	CoDA
Right F1	RKHHLGR	GGG	-	CoDA
Right F2	LKEHLTR	GGC	-	CoDA
Right F3	RKDGLTR	GCG	-	CoDA

ZF DNA Sequence

Bos taurus (cow) Build 3.1

Blast GGCGGCGCCNNNNNGCGGGCGGG

ZFN-unknown-SP-5-21
3857 tCTCTTCCCCCTGCGGCCGGCGGGg 3881
3857 aGAGAAGGGGGACGCCGGCCGCCCc 3881

FINGER	HELIX	TRIPLET	REFERENCE NUMBER	SOURCE
Left F1	KHSNLTR	GAG	-	CoDA
Left F2	QQTNLTR	GAA	-	CoDA
Left F3	RIDKLGG	GGG	-	CoDA
Right F1	RKHHLGR	GGG	-	CoDA
Right F2	LKEHLTR	GGC	-	CoDA
Right F3	DPSNLRR	GCC	-	CoDA

ZF DNA Sequence

Bos taurus (cow) Build 3.1

Blast CTCTTCCCCNNNNNGCCGGCGGG

ZFN-unknown-SP-5-22
3861 tTCCCCCTGCGGCCGGCGGGGGCg 3885
3861 aAGGGGGACGCCGGCCGCCCCCGc 3885

FINGER	HELIX	TRIPLET	REFERENCE NUMBER	SOURCE
Left F1	TSAHLAR	GGA	-	CoDA
Left F2	RREHLVR	GGG	-	CoDA
Left F3	QTATLKR	GCA	-	CoDA
Right F1	TNSKLTR	GGC	-	CoDA
Right F2	RREHLVR	GGG	-	CoDA
Right F3	RTDSLPR	GCG	-	CoDA

ZF DNA Sequence — Bos taurus (cow) Build 3.1

Blast TCCCCCTGCNNNNNGCGGGGGGC

ZFN-unknown-SP-5-23

3864 cCCCTGCGGCCGGCGGGGGGCGCGc 3888
3864 gGGGACGCCGGCCGCCCCCCGCGCg 3888

FINGER	HELIX	TRIPLET	REFERENCE NUMBER	SOURCE
Left F1	RRAHLQN	GGG	-	CoDA
Left F2	QSTTLKR	GCA	-	CoDA
Left F3	DPSNLRR	GCC	-	CoDA
Right F1	RRHGLDR	GCG	-	CoDA
Right F2	LKEHLTR	GGC	-	CoDA
Right F3	RGDKLAL	GGG	-	CoDA

ZF DNA Sequence — Bos taurus (cow) Build 3.1

Blast CCCTGCGGCNNNNNGGGGGCGCG

ZFN-unknown-SP-5-24

4115 gCGCGCCCCCGCGCGGCCGTCGCCc 4139
4115 cGCGCGGGGGCGCGCCGGCAGCGGg 4139

FINGER	HELIX	TRIPLET	REFERENCE NUMBER	SOURCE
Left F1	RRHGLDR	GCG	-	CoDA
Left F2	LKEHLTR	GGC	-	CoDA
Left F3	RGDKLAL	GGG	-	CoDA
Right F1	DSPTLRR	GCC	-	CoDA
Right F2	DHSSLKR	GTC	-	CoDA
Right F3	DPSNLRR	GCC	-	CoDA

ZF DNA Sequence — Bos taurus (cow) Build 3.1

Blast CGCGCCCCCNNNNNGCCGTCGCC

ZFN-unknown-SP-5-25

4151 aGTCCGCCCCGCGCCGCCGCCGCCc 4175
4151 tCAGGCGGGGCGCGGCGGCGGCGGg 4175

FINGER	HELIX	TRIPLET	REFERENCE NUMBER	SOURCE
Left F1	DPSNLIR	GAC	-	CoDA
Left F2	RTDTLAR	GCG	-	CoDA
Left F3	RKHRLDG	GGG	-	CoDA
Right F1	DGSTLRR	GCC	-	CoDA

Right F2	DSSVLRR	GCC	-	CoDA
Right F3	ERRGLAR	GCC	-	CoDA

ZF DNA Sequence

Bos taurus (cow) Build 3.1

Blast GTCCGCCCCNNNNNGCCGCCGCC

ZFN-unknown-SP-6-23

4164 cCGCCGCCGCCCCCGTGGTGTCTGCg 4189

4164 gGCGGCGGCGGGGGCACCACAGACGc 4189

FINGER	HELIX	TRIPLET	REFERENCE NUMBER	SOURCE
Left F1	RSNTLLR	GCG	-	CoDA
Left F2	RTDTLAR	GCG	-	CoDA
Left F3	RVDDLGR	GCG	-	CoDA
Right F1	RKRNLIM	TGC	-	CoDA
Right F2	DHSSLKR	GTC	-	CoDA
Right F3	QPHHLPR	GGT	-	CoDA

ZF DNA Sequence

Bos taurus (cow) Build 3.1

Blast CGCCGCCGCNNNNNNGGTGTCTGC

ZFN-unknown-SP-7-20

4212 cGCCGCCCGCCGTGCCGGTGGACGCGc 4238

4212 gCGGCGGGCGGCACGGCCACCTGCGCg 4238

FINGER	HELIX	TRIPLET	REFERENCE NUMBER	SOURCE
Left F1	SKHKLER	GGC	-	CoDA
Left F2	LKEHLTR	GGC	-	CoDA
Left F3	RKDGLTR	GCG	-	CoDA
Right F1	KHDTLHR	GCG	-	CoDA
Right F2	DRGNLTR	GAC	-	CoDA
Right F3	RGDALAR	GTG	-	CoDA

ZF DNA Sequence

Bos taurus (cow) Build 3.1

Blast GCCGCCCGCNNNNNNNGTGGACGCG

ZFN-unknown-SP-6-24

4369 cGGCACCAACACCCCCGGTGCCGCCc 4394

4369 gCCGTGGTTGTGGGGGCCACGGCGGg 4394

FINGER	HELIX	TRIPLET	REFERENCE NUMBER	SOURCE
Left F1	SKKSLTR	GCC	-	CoDA
Left F2	EAHHLSR	GGT	-	CoDA

Left F3	IRTSLKR	GTT	-	CoDA
Right F1	DGSTLRR	GCC	-	CoDA
Right F2	DSSVLRR	GCC	-	CoDA
Right F3	LTQGLRR	GGT	-	CoDA

ZF DNA Sequence   Bos taurus (cow) Build 3.1

Blast GGCACCAACNNNNNNGGTGCCGCC

ZFN-unknown-SP-7-21
4386 gTGCCGCCCCCACGCCGCGGAGGGGg 4412
4386 cACGGCGGGGGGTGCGGCGCCTCCCCc 4412

FINGER	HELIX	TRIPLET	REFERENCE NUMBER	SOURCE
Left F1	SPEQLAR	GCA	-	CoDA
Left F2	RTDTLAR	GCG	-	CoDA
Left F3	RKHRLDG	GGG	-	CoDA
Right F1	RNTHLAR	GGG	-	CoDA
Right F2	RQDNLGR	GAG	-	CoDA
Right F3	RHAALLS	GCG	-	CoDA

ZF DNA Sequence   Bos taurus (cow) Build 3.1

Blast TGCCGCCCCNNNNNNNGCGGAGGGG

ZFN-unknown-SP-6-25
4387 tGCCGCCCCCACGCCGCGGAGGGGg 4412
4387 aCGGCGGGGGGTGCGGCGCCTCCCCc 4412

FINGER	HELIX	TRIPLET	REFERENCE NUMBER	SOURCE
Left F1	SKHKLER	GGC	-	CoDA
Left F2	LKEHLTR	GGC	-	CoDA
Left F3	RGDKLAL	GGG	-	CoDA
Right F1	RNTHLAR	GGG	-	CoDA
Right F2	RQDNLGR	GAG	-	CoDA
Right F3	RHAALLS	GCG	-	CoDA

ZF DNA Sequence   Bos taurus (cow) Build 3.1

Blast GCCGCCCCCNNNNNNGCGGAGGGG

ZFN-unknown-SP-7-22
4389 cCGCCCCCACGCCGCGGAGGGGCGg 4415
4389 gGCGGGGGTGCGGCGCCTCCCCCGCc 4415

FINGER	HELIX	TRIPLET	REFERENCE NUMBER	SOURCE

Left F1	KRHTLTR	GCG	-	CoDA
Left F2	RREHLVR	GGG	-	CoDA
Left F3	RMDHLAG	TGG	-	CoDA
Right F1	KRHTLTR	GCG	-	CoDA
Right F2	RREHLVR	GGG	-	CoDA
Right F3	RRDNLLR	GAG	-	CoDA

ZF DNA Sequence Bos taurus (cow) Build 3.1

Blast CGCCCCCCANNNNNNNGAGGGGGCG

ZFN-unknown-SP-6-26

4389 cCGCCCCCCACGCCGCGGAGGGGGCg 4414

4389 gGCGGGGGGTGCGGCGCCTCCCCCGc 4414

FINGER	HELIX	TRIPLET	REFERENCE NUMBER	SOURCE
Left F1	KRHTLTR	GCG	-	CoDA
Left F2	RREHLVR	GGG	-	CoDA
Left F3	RMDHLAG	TGG	-	CoDA
Right F1	TNSKLTR	GGC	-	CoDA
Right F2	RREHLVR	GGG	-	CoDA
Right F3	QTTHLRR	GGA	-	CoDA

ZF DNA Sequence Bos taurus (cow) Build 3.1

Blast CGCCCCCCANNNNNNGGAGGGGGC

ZFN-unknown-SP-6-27

4390 cGCCCCCCACGCCGCGGAGGGGGCGg 4415

4390 gCGGGGGGTGCGGCGCCTCCCCCGCc 4415

FINGER	HELIX	TRIPLET	REFERENCE NUMBER	SOURCE
Left F1	TNSKLTR	GGC	-	CoDA
Left F2	RREHLVR	GGG	-	CoDA
Left F3	RPDALPR	GTG	-	CoDA
Right F1	KRHTLTR	GCG	-	CoDA
Right F2	RREHLVR	GGG	-	CoDA
Right F3	RRDNLLR	GAG	-	CoDA

ZF DNA Sequence Bos taurus (cow) Build 3.1

Blast GCCCCCCACNNNNNNGAGGGGGCG

ZFN-unknown-SP-5-26

4390 cGCCCCCCACGCCGCGGAGGGGGCg 4414

4390 gCGGGGGGTGCGGCGCCTCCCCCGc 4414

FINGER	HELIX	TRIPLET	REFERENCE NUMBER	SOURCE
Left F1	TNSKLTR	GGC	-	CoDA
Left F2	RREHLVR	GGG	-	CoDA
Left F3	RPDALPR	GTG	-	CoDA
Right F1	TNSKLTR	GGC	-	CoDA
Right F2	RREHLVR	GGG	-	CoDA
Right F3	QTTHLRR	GGA	-	CoDA

ZF DNA Sequence

Bos taurus (cow) Build 3.1

Blast GCCCCCCACNNNNNGGAGGGGGC

ZFN-unknown-SP-7-23

4417 gGCCCGCCCCCGGAAGAGGCGCGGGTc 4443

4417 cCGGGCGGGGGCCTTCTCCGCGCCCAg 4443

FINGER	HELIX	TRIPLET	REFERENCE NUMBER	SOURCE
Left F1	VPSKLAR	GGC	-	CoDA
Left F2	RTDTLAR	GCG	-	CoDA
Left F3	RKHRLDG	GGG	-	CoDA
Right F1	TKQKLQT	GGT	-	CoDA
Right F2	RTDTLAR	GCG	-	CoDA
Right F3	ESGHLKR	GGC	-	CoDA

ZF DNA Sequence

Bos taurus (cow) Build 3.1

Blast GCCCGCCCCNNNNNNNGGCGCGGGT

ZFN-unknown-SP-6-28

4418 gCCCGCCCCCGGAAGAGGCGCGGGTc 4443

4418 cGGGCGGGGGCCTTCTCCGCGCCCAg 4443

FINGER	HELIX	TRIPLET	REFERENCE NUMBER	SOURCE
Left F1	RKHHLGR	GGG	-	CoDA
Left F2	LKEHLTR	GGC	-	CoDA
Left F3	RGDKLAL	GGG	-	CoDA
Right F1	TKQKLQT	GGT	-	CoDA
Right F2	RTDTLAR	GCG	-	CoDA
Right F3	ESGHLKR	GGC	-	CoDA

ZF DNA Sequence

Bos taurus (cow) Build 3.1

Blast CCCGCCCCCNNNNNNGGCGCGGGT

ZFN-unknown-SP-5-27

4498 gCCCCTCGCCCCCCAGGGGGTGGGg 4522
4498 cGGGGAGCGGGGGGTCCCCCACCCCc 4522

FINGER	HELIX	TRIPLET	REFERENCE NUMBER	SOURCE
Left F1	RNTHLAR	GGG	-	CoDA
Left F2	RQDNLGR	GAG	-	CoDA
Left F3	KNHSLNN	GGC	-	CoDA
Right F1	KGDHLRR	GGG	-	CoDA
Right F2	EAHHLSR	GGT	-	CoDA
Right F3	RTEHLAR	GGG	-	CoDA

ZF DNA Sequence Bos taurus (cow) Build 3.1

Blast CCCCTCGCCNNNNNGGGGGTGGG

ZFN-unknown-SP-6-29
4498 gCCCCTCGCCCCCCAGGGGGTGGGGg 4523
4498 cGGGGAGCGGGGGGTCCCCCACCCCCc 4523

FINGER	HELIX	TRIPLET	REFERENCE NUMBER	SOURCE
Left F1	RNTHLAR	GGG	-	CoDA
Left F2	RQDNLGR	GAG	-	CoDA
Left F3	KNHSLNN	GGC	-	CoDA
Right F1	KKDHLHR	GGG	-	CoDA
Right F2	RREVLEN	GTG	-	CoDA
Right F3	RQGHLKR	GGG	-	CoDA

ZF DNA Sequence Bos taurus (cow) Build 3.1

Blast CCCCTCGCCNNNNNNGGGGTGGGG

ZFN-unknown-SP-7-24
4500 cCCTCGCCCCCCAGGGGGTGGGGGCCa 4526
4500 gGGAGCGGGGGGTCCCCCACCCCCGGt 4526

FINGER	HELIX	TRIPLET	REFERENCE NUMBER	SOURCE
Left F1	RSSHLKM	AGG	-	CoDA
Left F2	RTDTLAR	GCG	-	CoDA
Left F3	RKHRLDG	GGG	-	CoDA
Right F1	SHTVLTR	GCC	-	CoDA
Right F2	RREHLVR	GGG	-	CoDA
Right F3	RPDALPR	GTG	-	CoDA

ZF DNA Sequence Bos taurus (cow) Build 3.1

Blast CCTCGCCCCNNNNNNNGTGGGGGCC

ZFN-unknown-SP-6-30
4500 cCCTCGCCCCCCAGGGGGTGGGGGCc 4525
4500 gGGAGCGGGGGGTCCCCCACCCCCGg 4525

FINGER	HELIX	TRIPLET	REFERENCE NUMBER	SOURCE
Left F1	RSSHLKM	AGG	-	CoDA
Left F2	RTDTLAR	GCG	-	CoDA
Left F3	RKHRLDG	GGG	-	CoDA
Right F1	TNSKLTR	GGC	-	CoDA
Right F2	RREHLVR	GGG	-	CoDA
Right F3	VDHHLRR	GGT	-	CoDA

ZF DNA Sequence   Bos taurus (cow) Build 3.1

Blast CCTCGCCCCNNNNNNGGTGGGGGC

ZFN-unknown-SP-7-25
4587 tCGCCCCGGCGTCCGCGGGCGCCGCGc 4613
4587 aGCGGGGCCGCAGGCGCCCGCGGCGCg 4613

FINGER	HELIX	TRIPLET	REFERENCE NUMBER	SOURCE
Left F1	KRHTLTR	GCG	-	CoDA
Left F2	RREHLVR	GGG	-	CoDA
Left F3	EHRGLKR	GCC	-	CoDA
Right F1	RLRDLPR	GCG	-	CoDA
Right F2	DSSVLRR	GCC	-	CoDA
Right F3	ENSKLNR	GGC	-	CoDA

ZF DNA Sequence   Bos taurus (cow) Build 3.1

Blast CGCCCCGGCNNNNNNNGGCGCCGCG

ZFN-unknown-SP-7-26
4623 cGTCTCCGTCGTCCCAGGCCGCGGTCg 4649
4623 gCAGAGGCAGCAGGGTCCGGCGCCAGc 4649

FINGER	HELIX	TRIPLET	REFERENCE NUMBER	SOURCE
Left F1	DEANLRR	GAC	-	CoDA
Left F2	QSAHLKR	GGA	-	CoDA
Left F3	DPSNLRR	GAC	-	CoDA
Right F1	TSTLLNR	GTC	-	CoDA
Right F2	RTDTLAR	GCG	-	CoDA
Right F3	ERRGLAR	GCC	-	CoDA

ZF DNA Sequence   Bos taurus (cow) Build 3.1

Blast GTCTCCGTCNNNNNNNGCCGCGGTC

ZFN-unknown-SP-7-27
4626 cTCCGTCGTCCCAGGCCGCGGTCGCCg 4652
4626 gAGGCAGCAGGGTCCGGCGCCAGCGGc 4652

FINGER	HELIX	TRIPLET	REFERENCE NUMBER	SOURCE
Left F1	RPAKLVL	GGA	-	CoDA
Left F2	DRGNLTR	GAC	-	CoDA
Left F3	DQGNLIR	GAC	-	CoDA
Right F1	DSPTLRR	GCC	-	CoDA
Right F2	DHSSLKR	GTC	-	CoDA
Right F3	RTDLLRR	GCG	-	CoDA

ZF DNA Sequence

Bos taurus (cow) Build 3.1

Blast TCCGTCGTCNNNNNNNGCGGTCGCC

ZFN-unknown-SP-7-28
4629 cGTCGTCCCAGGCCGCGGTCGCCGCCg 4655
4629 gCAGCAGGGTCCGGCGCCAGCGGCGGc 4655

FINGER	HELIX	TRIPLET	REFERENCE NUMBER	SOURCE
Left F1	EESNLRR	GAC	-	CoDA
Left F2	DRGNLTR	GAC	-	CoDA
Left F3	RSDHLSL	TGG	-	CoDA
Right F1	DGSTLRR	GCC	-	CoDA
Right F2	DSSVLRR	GCC	-	CoDA
Right F3	EGGALRR	GTC	-	CoDA

ZF DNA Sequence

Bos taurus (cow) Build 3.1

Blast GTCGTCCCANNNNNNNGTCGCCGCC

ZFN-unknown-SP-7-29
4755 cCTCCGCCTCTTCCTCTGCGGGCGGGg 4781
4755 gGAGGCGGAGAAGGAGACGCCCGCCCc 4781

FINGER	HELIX	TRIPLET	REFERENCE NUMBER	SOURCE
Left F1	RMSNLDR	GAG	-	CoDA
Left F2	RTDTLAR	GCG	-	CoDA
Left F3	RRDNLLR	GAG	-	CoDA
Right F1	RKHHLGR	GGG	-	CoDA
Right F2	LKEHLTR	GGC	-	CoDA
Right F3	RKDGLTR	GCG	-	CoDA

ZF DNA Sequence

Bos taurus (cow) Build 3.1

Blast CTCCGCCTCNNNNNNNGCGGGCGGG

ZFN-unknown-SP-7-30

4758 cCGCCTCTTCCTCTGCGGGCGGGGCTg 4784

4758 gGCGGAGAAGGAGACGCCCGCCCCGAc 4784

FINGER	HELIX	TRIPLET	REFERENCE NUMBER	SOURCE
Left F1	RKLGLLR	GCG	-	CoDA
Left F2	RQDNLGR	GAG	-	CoDA
Left F3	QRNNLGR	GAA	-	CoDA
Right F1	THSMLAR	GCT	-	CoDA
Right F2	RREHLVR	GGG	-	CoDA
Right F3	ESGHLKR	GGC	-	CoDA

ZF DNA Sequence

Bos taurus (cow) Build 3.1

Blast CGCCTCTTCNNNNNNNGGCGGGGCT

ZFN-unknown-SP-7-31

4761 cCTCTTCCTCTGCGGGCGGGGCTGGTg 4787

4761 gGAGAAGGAGACGCCCGCCCCGACCAc 4787

FINGER	HELIX	TRIPLET	REFERENCE NUMBER	SOURCE
Left F1	KHSNLTR	GAG	-	CoDA
Left F2	QQTNLTR	GAA	-	CoDA
Left F3	RRDNLNR	GAG	-	CoDA
Right F1	RRQKLTI	GGT	-	CoDA
Right F2	QRSDLTR	GCT	-	CoDA
Right F3	RTEHLAR	GGG	-	CoDA

ZF DNA Sequence

Bos taurus (cow) Build 3.1

Blast CTCTTCCTCNNNNNNNGGGGCTGGT

ZFN-unknown-SP-7-32

4764 cTTCCTCTGCGGGCGGGGCTGGTGGGa 4790

4764 gAAGGAGACGCCCGCCCCGACCACCCt 4790

FINGER	HELIX	TRIPLET	REFERENCE NUMBER	SOURCE
Left F1	QASNLLR	GAA	-	CoDA
Left F2	RQDNLGR	GAG	-	CoDA
Left F3	QSNVLSR	GCA	-	CoDA
Right F1	KGDHLRR	GGG	-	CoDA

Right F2	EAHHLSR	GGT	-	CoDA
Right F3	EGSGLKR	GCT	-	CoDA

ZF DNA Sequence

Bos taurus (cow) Build 3.1

Blast TTCCTCTGCNNNNNNNGCTGGTGGG

ZFN-unknown-SP-7-33

4791 gCGTCGCGTCCGCGTCCGGCGCTGGGg 4817

4791 cGCAGCGCAGGCGCAGGCCGCGACCCc 4817

FINGER	HELIX	TRIPLET	REFERENCE NUMBER	SOURCE
Left F1	RSQTLAQ	ACG	-	CoDA
Left F2	RTDTLAR	GCG	-	CoDA
Left F3	DPSNLRR	GAC	-	CoDA
Right F1	KKDHLHR	GGG	-	CoDA
Right F2	QRSDLTR	GCT	-	CoDA
Right F3	ESGHLRR	GGC	-	CoDA

ZF DNA Sequence

Bos taurus (cow) Build 3.1

Blast CGTCGCGTCNNNNNNNGGCGCTGGG

ZFN-unknown-SP-7-34

4794 tCGCGTCCGCGTCCGGCGCTGGGGAGa 4820

4794 aGCGCAGGCGCAGGCCGCGACCCCTCt 4820

FINGER	HELIX	TRIPLET	REFERENCE NUMBER	SOURCE
Left F1	KHDTLHR	GCG	-	CoDA
Left F2	DRGNLTR	GAC	-	CoDA
Left F3	RRDGLTR	GCG	-	CoDA
Right F1	RNTNLTR	GAG	-	CoDA
Right F2	RREHLVR	GGG	-	CoDA
Right F3	VSNSLAR	GCT	-	CoDA

ZF DNA Sequence

Bos taurus (cow) Build 3.1

Blast CGCGTCCGCNNNNNNNGCTGGGGAG

ZFN-unknown-SP-6-31

4797 cGTCCGCGTCCGGCGCTGGGGAGAGa 4822

4797 gCAGGCGCAGGCCGCGACCCCTCTCt 4822

FINGER	HELIX	TRIPLET	REFERENCE NUMBER	SOURCE
Left F1	DPSNLIR	GAC	-	CoDA
Left F2	RTDTLAR	GCG	-	CoDA

Left F3	DPSNLRR	GAC	-	CoDA
Right F1	KHSNLTR	GAG	-	CoDA
Right F2	QSAHLKR	GGA	-	CoDA
Right F3	RSDHLSL	TGG	-	CoDA

ZF DNA Sequence Bos taurus (cow) Build 3.1

Blast GTCCGCGTCNNNNNNTGGGGAGAG

ZFN-unknown-SP-6-32
4831 cTCCCTCGGCCCCCGCGCTGCTGCGc 4856
4831 gAGGGAGCCGGGGGCGCGACGACGCg 4856

FINGER	HELIX	TRIPLET	REFERENCE NUMBER	SOURCE
Left F1	THAHLTR	GGA	-	CoDA
Left F2	RQDNLGR	GAG	-	CoDA
Left F3	DKSVLAR	GCC	-	CoDA
Right F1	RKGTLDR	GCG	-	CoDA
Right F2	QRSDLTR	GCT	-	CoDA
Right F3	LRASLRR	GCT	-	CoDA

ZF DNA Sequence Bos taurus (cow) Build 3.1

Blast TCCCTCGGCNNNNNNGCTGCTGCG

ZFN-unknown-SP-5-28
4837 cGGCCCCCGCGCTGCTGCGCCGCGg 4861
4837 gCCGGGGGCGCGACGACGCGGCGCc 4861

FINGER	HELIX	TRIPLET	REFERENCE NUMBER	SOURCE
Left F1	SHTVLTR	GCC	-	CoDA
Left F2	RREHLVR	GGG	-	CoDA
Left F3	RTDSLPR	GCG	-	CoDA
Right F1	RLRDLPR	GCG	-	CoDA
Right F2	DSSVLRR	GCC	-	CoDA
Right F3	QGRSLRA	TGC	-	CoDA

ZF DNA Sequence Bos taurus (cow) Build 3.1

Blast GGCCCCCGCNNNNNTGCGCCGCG

ZFN-unknown-SP-6-33
4845 gCGCTGCTGCGCCGCGGGGGCCGAGg 4870
4845 cGCGACGACGCGGCGCCCCCGGCTCc 4870

FINGER	HELIX	TRIPLET	REFERENCE NUMBER	SOURCE

Left F1	RRHTLTR	GCG	-	CoDA
Left F2	QSTTLKR	GCA	-	CoDA
Left F3	QPNTLTR	GCA	-	CoDA
Right F1	KKTNLTR	GAG	-	CoDA
Right F2	DSSVLRR	GCC	-	CoDA
Right F3	RTEHLAR	GGG	-	CoDA

ZF DNA Sequence Bos taurus (cow) Build 3.1

Blast CGCTGCTGCNNNNNNGGGGCCGAG

ZFN-unknown-SP-6-34
4848 cTGCTGCGCCGCGGGGGCCGAGGAAg 4873
4848 gACGACGCGGCGCCCCCGGCTCCTTc 4873

FINGER	HELIX	TRIPLET	REFERENCE NUMBER	SOURCE
Left F1	QRGTLNR	GCA	-	CoDA
Left F2	QSTTLKR	GCA	-	CoDA
Left F3	DGGHLTR	GGC	-	CoDA
Right F1	QASNLLR	GAA	-	CoDA
Right F2	RQDNLGR	GAG	-	CoDA
Right F3	DKSVLAR	GCC	-	CoDA

ZF DNA Sequence Bos taurus (cow) Build 3.1

Blast TGCTGCGCCNNNNNNGCCGAGGAA

ZFN-unknown-SP-5-29
5112 cCGCGGCCCCCGCGTGGAGCCGCCg 5136
5112 gGCGCCGGGGGCGCACCTCGGCGGc 5136

FINGER	HELIX	TRIPLET	REFERENCE NUMBER	SOURCE
Left F1	RLRDLPR	GCG	-	CoDA
Left F2	DSSVLRR	GCC	-	CoDA
Left F3	RTEHLAR	GGG	-	CoDA
Right F1	DGSTLRR	GCC	-	CoDA
Right F2	DSSVLRR	GCC	-	CoDA
Right F3	QNSHLRR	GGA	-	CoDA

ZF DNA Sequence Bos taurus (cow) Build 3.1

Blast CGCGGCCCCNNNNNGGAGCCGCC

ZFN-unknown-SP-5-30
5115 cGGCCCCCGCGTGGAGCCGCCGCAc 5139
5115 gCCGGGGGCGCACCTCGGCGGCGTg 5139

FINGER	HELIX	TRIPLET	REFERENCE NUMBER	SOURCE
Left F1	SHTVLTR	GCC	-	CoDA
Left F2	RREHLVR	GGG	-	CoDA
Left F3	RTDSLPR	GCG	-	CoDA
Right F1	LNHTLKR	GCA	-	CoDA
Right F2	DSSVLRR	GCC	-	CoDA
Right F3	ERRGLAR	GCC	-	CoDA

ZF DNA Sequence Bos taurus (cow) Build 3.1

Blast GGCCCCCGCNNNNNGCCGCCGCA

ZFN-unknown-SP-7-35
5186 cCGACGCCCCCCGCGTCGGAGTGGAAc 5212
5186 gGCTGCGGGGGGCGCAGCCTCACCTTg 5212

FINGER	HELIX	TRIPLET	REFERENCE NUMBER	SOURCE
Left F1	KNNDLLK	TCG	-	CoDA
Left F2	RTDTLAR	GCG	-	CoDA
Left F3	RKHRLDG	GGG	-	CoDA
Right F1	HKPNLHR	GAA	-	CoDA
Right F2	RREVLEN	GTG	-	CoDA
Right F3	QKPHLSR	GGA	-	CoDA

ZF DNA Sequence Bos taurus (cow) Build 3.1

Blast CGACGCCCCNNNNNNNGGAGTGGAA

ZFN-unknown-SP-7-36
5187 cGACGCCCCCCGCGTCGGAGTGGAACa 5213
5187 gCTGCGGGGGGCGCAGCCTCACCTTGt 5213

FINGER	HELIX	TRIPLET	REFERENCE NUMBER	SOURCE
Left F1	TGAVLTR	GTC	-	CoDA
Left F2	LKEHLTR	GGC	-	CoDA
Left F3	RGDKLAL	GGG	-	CoDA
Right F1	GPTALVN	AAC	-	CoDA
Right F2	RREHLTI	TGG	-	CoDA
Right F3	RRDNLNR	GAG	-	CoDA

ZF DNA Sequence Bos taurus (cow) Build 3.1

Blast GACGCCCCCNNNNNNNGAGTGGAAC

ZFN-unknown-SP-6-35

5187 cGACGCCCCCCGCGTCGGAGTGGAAc 5212
5187 gCTGCGGGGGGCGCAGCCTCACCTTg 5212

FINGER	HELIX	TRIPLET	REFERENCE NUMBER	SOURCE
Left F1	TGAVLTR	GTC	-	CoDA
Left F2	LKEHLTR	GGC	-	CoDA
Left F3	RGDKLAL	GGG	-	CoDA
Right F1	HKPNLHR	GAA	-	CoDA
Right F2	RREVLEN	GTG	-	CoDA
Right F3	QKPHLSR	GGA	-	CoDA

ZF DNA Sequence

Bos taurus (cow) Build 3.1

Blast GACGCCCCCNNNNNNGGAGTGGAA

ZFN-unknown-SP-7-37
5294 tCGCGCCACCCGTGGTCTCGGGAGCAg 5320
5294 aGCGCGGTGGGCACCAGAGCCCTCGTc 5320

FINGER	HELIX	TRIPLET	REFERENCE NUMBER	SOURCE
Left F1	RRHGLDR	GCG	-	CoDA
Left F2	LKEHLTR	GGC	-	CoDA
Left F3	MGHHLKR	GGT	-	CoDA
Right F1	KNTRLSV	GCA	-	CoDA
Right F2	QSAHLKR	GGA	-	CoDA
Right F3	RGDSLKK	TCG	-	CoDA

ZF DNA Sequence

Bos taurus (cow) Build 3.1

Blast CGCGCCACCNNNNNNNTCGGGAGCA

ZFN-unknown-SP-6-36
5420 cACCGGCCGAGGCGCCTCGGGGGCAg 5445
5420 gTGGCCGGCTCCGCGGAGCCCCCGTc 5445

FINGER	HELIX	TRIPLET	REFERENCE NUMBER	SOURCE
Left F1	RQSRLQR	GGT	-	CoDA
Left F2	DSSVLRR	GCC	-	CoDA
Left F3	RRDGLSG	TCG	-	CoDA
Right F1	DRSQLAR	GCA	-	CoDA
Right F2	RREHLVR	GGG	-	CoDA
Right F3	RMDSLGG	TCG	-	CoDA

ZF DNA Sequence

Bos taurus (cow) Build 3.1

Blast ACCGGCCGANNNNNNTCGGGGGCA

ZFN-unknown-SP-7-38
5667 cAGCTCCCCCGCGCAGGGGGGCCGGGg 5693
5667 gTCGAGGGGGCGCGTCCCCCGGCCCc 5693

FINGER	HELIX	TRIPLET	REFERENCE NUMBER	SOURCE
Left F1	TTQALRR	GCT	-	CoDA
Left F2	QSAHLKR	GGA	-	CoDA
Left F3	RTEHLAR	GGG	-	CoDA
Right F1	KGDHLRR	GGG	-	CoDA
Right F2	DSSVLRR	GCC	-	CoDA
Right F3	RTEHLAR	GGG	-	CoDA

ZF DNA Sequence Bos taurus (cow) Build 3.1

Blast AGCTCCCCCNNNNNNNGGGGCCGGG

ZFN-unknown-SP-5-31
5669 gCTCCCCCGCGCAGGGGGGCCGGGg 5693
5669 cGAGGGGGCGCGTCCCCCGGCCCc 5693

FINGER	HELIX	TRIPLET	REFERENCE NUMBER	SOURCE
Left F1	RNTNLTR	GAG	-	CoDA
Left F2	RREHLVR	GGG	-	CoDA
Left F3	RTDSLPR	GCG	-	CoDA
Right F1	KGDHLRR	GGG	-	CoDA
Right F2	DSSVLRR	GCC	-	CoDA
Right F3	RTEHLAR	GGG	-	CoDA

ZF DNA Sequence Bos taurus (cow) Build 3.1

Blast CTCCCCCGCNNNNNNGGGGCCGGG

ZFN-unknown-SP-7-39
5735 aAGCCGCCCCCACCCCCGAGGCCTGTt 5761
5735 tTCGGCGGGGGTGGGGGCTCCGGACAa 5761

FINGER	HELIX	TRIPLET	REFERENCE NUMBER	SOURCE
Left F1	KHQTLQR	GCT	-	CoDA
Left F2	RTDTLAR	GCG	-	CoDA
Left F3	RKHRLDG	GGG	-	CoDA
Right F1	RKQHLTL	TGT	-	CoDA
Right F2	DSSVLRR	GCC	-	CoDA
Right F3	RVDNLPR	GAG	-	CoDA

ZF DNA Sequence Bos taurus (cow) Build 3.1

Blast AGCCGCCCCNNNNNNNGAGGCCTGT

ZFN-unknown-SP-6-37
5736 aGCCGCCCCCACCCCCGAGGCCTGTt 5761
5736 tCGGCGGGGGTGGGGGCTCCGGACAa 5761

FINGER	HELIX	TRIPLET	REFERENCE NUMBER	SOURCE
Left F1	SKHKLER	GGC	-	CoDA
Left F2	LKEHLTR	GGC	-	CoDA
Left F3	RGDKLAL	GGG	-	CoDA
Right F1	RKQHLTL	TGT	-	CoDA
Right F2	DSSVLRR	GCC	-	CoDA
Right F3	RVDNLPR	GAG	-	CoDA

ZF DNA Sequence

Bos taurus (cow) Build 3.1

Blast GCCGCCCCCNNNNNNGAGGCCTGT

ZFN-unknown-SP-7-40
6073 gCACGCCCTCTGCCCCCGGGGACGGGt 6099
6073 cGTGCGGGAGACGGGGGCCCCTGCCCa 6099

FINGER	HELIX	TRIPLET	REFERENCE NUMBER	SOURCE
Left F1	SRFTLGR	GTG	-	CoDA
Left F2	LKEHLTR	GGC	-	CoDA
Left F3	RVDNLPR	GAG	-	CoDA
Right F1	KKDHLHR	GGG	-	CoDA
Right F2	DRGNLTR	GAC	-	CoDA
Right F3	RNHGLVR	GGG	-	CoDA

ZF DNA Sequence

Bos taurus (cow) Build 3.1

Blast CACGCCCTCNNNNNNNGGGGACGGG

ZFN-unknown-SP-7-41
6213 cCTCCTCACCCTGGCTGGGAGAAAGGa 6239
6213 gGAGGAGTGGGACCGACCCTCTTTCCt 6239

FINGER	HELIX	TRIPLET	REFERENCE NUMBER	SOURCE
Left F1	RQMNLDR	GAG	-	CoDA
Left F2	RQDNLGR	GAG	-	CoDA
Left F3	VKHGLGR	GGT	-	CoDA
Right F1	RMAHLHA	AGG	-	CoDA
Right F2	QQTNLTR	GAA	-	CoDA
Right F3	QTTHLSR	GGA	-	CoDA

ZF DNA Sequence | Bos taurus (cow) Build 3.1

Blast CTCCTCACCNNNNNNNGGAGAAAGG

ZFN-unknown-SP-5-32

6318 cCCCTCCTTACCCTGGCTGCGGGGa 6342
6318 gGGGAGGAATGGGACCGACGCCCCt 6342

FINGER	HELIX	TRIPLET	REFERENCE NUMBER	SOURCE
Left F1	KKDHLHR	GGG	-	CoDA
Left F2	QSAHLKR	GGA	-	CoDA
Left F3	QRGNLNM	TAA	-	CoDA
Right F1	RGNHLRR	GGG	-	CoDA
Right F2	RTDTLAR	GCG	-	CoDA
Right F3	LKHDLRR	GCT	-	CoDA

ZF DNA Sequence | Bos taurus (cow) Build 3.1

Blast CCCTCCTTANNNNNGCTGCGGGG

ZFN-unknown-SP-7-42

6318 cCCCTCCTTACCCTGGCTGCGGGGAGa 6344
6318 gGGGAGGAATGGGACCGACGCCCCTCt 6344

FINGER	HELIX	TRIPLET	REFERENCE NUMBER	SOURCE
Left F1	KKDHLHR	GGG	-	CoDA
Left F2	QSAHLKR	GGA	-	CoDA
Left F3	QRGNLNM	TAA	-	CoDA
Right F1	RNTNLTR	GAG	-	CoDA
Right F2	RREHLVR	GGG	-	CoDA
Right F3	ANRTLVH	TGC	-	CoDA

ZF DNA Sequence | Bos taurus (cow) Build 3.1

Blast CCCTCCTTANNNNNNNTGCGGGGAG

ZFN-unknown-SP-7-43

6341 gAGACTCCCATCGGGGCGAGGGGGCTc 6367
6341 cTCTGAGGGTAGCCCCGCTCCCCCGAg 6367

FINGER	HELIX	TRIPLET	REFERENCE NUMBER	SOURCE
Left F1	SKPNLKM	TCT	-	CoDA
Left F2	RQDNLGR	GAG	-	CoDA
Left F3	RMDHLAG	TGG	-	CoDA
Right F1	THSMLAR	GCT	-	CoDA

Right F2	RREHLVR	GGG	-	CoDA
Right F3	RRDNLLR	GAG	-	CoDA

ZF DNA Sequence Bos taurus (cow) Build 3.1

Blast AGACTCCCANNNNNNNGAGGGGGCT

ZFN-unknown-SP-6-38
6544 cCCCGCCCGCGCCCACGCGGTAGAGg 6569
6544 gGGGCGGGCGCGGGTGCGCCATCTCc 6569

FINGER	HELIX	TRIPLET	REFERENCE NUMBER	SOURCE
Left F1	RKHHLGR	GGG	-	CoDA
Left F2	LKEHLTR	GGC	-	CoDA
Left F3	RKDGLTR	GCG	-	CoDA
Right F1	KHSNLTR	GAG	-	CoDA
Right F2	QRSSLVR	GTA	-	CoDA
Right F3	RRDDLTR	GCG	-	CoDA

ZF DNA Sequence Bos taurus (cow) Build 3.1

Blast CCCGCCCGCNNNNNNGCGGTAGAG

ZFN-unknown-SP-6-39
6547 cGCCCGCGCCCACGCGGTAGAGGAAg 6572
6547 gCGGGCGCGGGTGCGCCATCTCCTTc 6572

FINGER	HELIX	TRIPLET	REFERENCE NUMBER	SOURCE
Left F1	VPSKLAR	GGC	-	CoDA
Left F2	RTDTLAR	GCG	-	CoDA
Left F3	ESGHLKR	GGC	-	CoDA
Right F1	QASNLLR	GAA	-	CoDA
Right F2	RQDNLGR	GAG	-	CoDA
Right F3	QMNALQR	GTA	-	CoDA

ZF DNA Sequence Bos taurus (cow) Build 3.1

Blast GCCCGCGCCNNNNNNGTAGAGGAA

ZFN-unknown-SP-6-40
6550 cCGCGCCCACGCGGTAGAGGAAGGGg 6575
6550 gGCGCGGGTGCGCCATCTCCTTCCCc 6575

FINGER	HELIX	TRIPLET	REFERENCE NUMBER	SOURCE
Left F1	RRHGLDR	GCG	-	CoDA
Left F2	LKEHLTR	GGC	-	CoDA

Left F3	RKDALHV	GTG	-	CoDA
Right F1	KRERLDR	GGG	-	CoDA
Right F2	QQTNLTR	GAA	-	CoDA
Right F3	RRDNLNR	GAG	-	CoDA

ZF DNA Sequence Bos taurus (cow) Build 3.1

Blast CGCGCCCACNNNNNNGAGGAAGGG

ZFN-unknown-SP-6-41
6578 gGGCGCCACACCCACGGCTGTGGCCg 6603
6578 cCCGCGGTGTGGGTGCCGACACCGGc 6603

FINGER	HELIX	TRIPLET	REFERENCE NUMBER	SOURCE
Left F1	SNKDLTR	GCC	-	CoDA
Left F2	LKEHLTR	GGC	-	CoDA
Left F3	QAHGLTG	TGT	-	CoDA
Right F1	KRRDLDR	GCC	-	CoDA
Right F2	RREVLEN	GTG	-	CoDA
Right F3	VGASLKR	GCT	-	CoDA

ZF DNA Sequence Bos taurus (cow) Build 3.1

Blast GGCGCCACANNNNNNGCTGTGGCC

ZFN-unknown-SP-7-44
6580 gCGCCACACCCACGGCTGTGGCCGGGc 6606
6580 cGCGGTGTGGGTGCCGACACCGGCCCg 6606

FINGER	HELIX	TRIPLET	REFERENCE NUMBER	SOURCE
Left F1	RAHTLRR	GCG	-	CoDA
Left F2	RREVLEN	GTG	-	CoDA
Left F3	VKHGLTR	GGT	-	CoDA
Right F1	KGDHLRR	GGG	-	CoDA
Right F2	DSSVLRR	GCC	-	CoDA
Right F3	RHTSLTR	GTG	-	CoDA

ZF DNA Sequence Bos taurus (cow) Build 3.1

Blast CGCCACACCNNNNNNNGTGGCCGGG

ZFN-unknown-SP-6-42
6604 gGCACGCGCCTTTGGGGTTGTTGGGg 6629
6604 cCGTGCGCGGAAACCCCAACAACCCc 6629

FINGER	HELIX	TRIPLET	REFERENCE NUMBER	SOURCE

Left F1	RSRNLTL	TGC	-	CoDA
Left F2	RTDTLAR	GCG	-	CoDA
Left F3	ESGHLKR	GGC	-	CoDA
Right F1	KSNHLHV	GGG	-	CoDA
Right F2	HKSSLTR	GTT	-	CoDA
Right F3	IRTSLKR	GTT	-	CoDA

ZF DNA Sequence

Bos taurus (cow) Build 3.1

Blast GCACGCGCCNNNNNNGTTGTTGGG

ZFN-unknown-SP-6-43
6679 cTGCCCCCCCGGGAACGACGACGGGg 6704
6679 gACGGGGGGGCCCTTGCTGCTGCCCc 6704

FINGER	HELIX	TRIPLET	REFERENCE NUMBER	SOURCE
Left F1	DRSQLAR	GCA	-	CoDA
Left F2	RREHLVR	GGG	-	CoDA
Left F3	RNDKLVP	GGG	-	CoDA
Right F1	KKDHLHR	GGG	-	CoDA
Right F2	DRGNLTR	GAC	-	CoDA
Right F3	DQGNLIR	GAC	-	CoDA

ZF DNA Sequence

Bos taurus (cow) Build 3.1

Blast TGCCCCCCCNNNNNNGACGACGGG

ZFN-unknown-SP-6-44
6978 tCTCCCCGCCGCCTCAGCAGAAGCTc 7003
6978 aGAGGGGCGGCGGAGTCGTCTTCGAg 7003

FINGER	HELIX	TRIPLET	REFERENCE NUMBER	SOURCE
Left F1	RNTNLTR	GAG	-	CoDA
Left F2	RREHLVR	GGG	-	CoDA
Left F3	ESGHLKR	GGC	-	CoDA
Right F1	QRQALDR	GCT	-	CoDA
Right F2	QQTNLTR	GAA	-	CoDA
Right F3	QGNTLTR	GCA	-	CoDA

ZF DNA Sequence

Bos taurus (cow) Build 3.1

Blast CTCCCCGCCNNNNNNGCAGAAGCT

ZFN-unknown-SP-7-45
7010 aCGACCCTAAACCTACCTGCGAGAACg 7036
7010 tGCTGGGATTTGGATGGACGCTCTTGc 7036

FINGER	HELIX	TRIPLET	REFERENCE NUMBER	SOURCE
Left F1	KNNDLLK	TCG	-	CoDA
Left F2	RREHLVR	GGG	-	CoDA
Left F3	QQTGLNV	TTA	-	CoDA
Right F1	GHTALRN	AAC	-	CoDA
Right F2	RQDNLGR	GAG	-	CoDA
Right F3	ANRTLVH	TGC	-	CoDA

ZF DNA Sequence

Bos taurus (cow) Build 3.1

Blast CGACCCTAANNNNNNNTGCGAGAAC

ZFN-unknown-SP-7-46

7129 gCGCGGCGTCTCGGTGGGACGCGGGCa 7155

7129 cGCGCCGCAGAGCCACCCTGCGCCCGt 7155

FINGER	HELIX	TRIPLET	REFERENCE NUMBER	SOURCE
Left F1	RLRDLPR	GCG	-	CoDA
Left F2	DSSVLRR	GCC	-	CoDA
Left F3	EGGNLMR	GAC	-	CoDA
Right F1	VPSKLAR	GGC	-	CoDA
Right F2	RTDTLAR	GCG	-	CoDA
Right F3	DPSNLRR	GAC	-	CoDA

ZF DNA Sequence

Bos taurus (cow) Build 3.1

Blast CGCGGCGTCNNNNNNNGACGCGGGC

ZFN-unknown-SP-6-45

7158 gGGCGGCGGCGGCGGGGGGGGGGGg 7183

7158 cCCGCCGCCGCCGCCCCCCCCCCCc 7183

FINGER	HELIX	TRIPLET	REFERENCE NUMBER	SOURCE
Left F1	DGSTLRR	GCC	-	CoDA
Left F2	DSSVLRR	GCC	-	CoDA
Left F3	ERRGLAR	GCC	-	CoDA
Right F1	RKHHLGR	GGG	-	CoDA
Right F2	RREHLVR	GGG	-	CoDA
Right F3	RNDKLVP	GGG	-	CoDA

ZF DNA Sequence

Bos taurus (cow) Build 3.1

Blast GGCGGCGGCNNNNNNGGGGGGGGG

ZFN-unknown-SP-7-47

7158 gGGCGGCGGCGGCGGGGGGGGGGGGg 7184
7158 cCCGCCGCCGCCGCCCCCCCCCCCCCc 7184

FINGER	HELIX	TRIPLET	REFERENCE NUMBER	SOURCE
Left F1	DGSTLRR	GCC	-	CoDA
Left F2	DSSVLRR	GCC	-	CoDA
Left F3	ERRGLAR	GCC	-	CoDA
Right F1	RKHHLGR	GGG	-	CoDA
Right F2	RREHLVR	GGG	-	CoDA
Right F3	RNDKLVP	GGG	-	CoDA

Bos taurus (cow) Build 3.1

ZF DNA Sequence

Blast GGCGGCGGCNNNNNNNGGGGGGGGG

ZFN-unknown-SP-5-33
7158 gGGCGGCGGCGGCGGGGGGGGGGGg 7182
7158 cCCGCCGCCGCCGCCCCCCCCCCCCc 7182

FINGER	HELIX	TRIPLET	REFERENCE NUMBER	SOURCE
Left F1	DGSTLRR	GCC	-	CoDA
Left F2	DSSVLRR	GCC	-	CoDA
Left F3	ERRGLAR	GCC	-	CoDA
Right F1	RKHHLGR	GGG	-	CoDA
Right F2	RREHLVR	GGG	-	CoDA
Right F3	RNDKLVP	GGG	-	CoDA

Bos taurus (cow) Build 3.1

ZF DNA Sequence

Blast GGCGGCGGCNNNNNGGGGGGGGG

ZFN-unknown-SP-5-34
7161 cGGCGGCGGCGGGGGGGGGGGGGGg 7185
7161 gCCGCCGCCGCCCCCCCCCCCCCCc 7185

FINGER	HELIX	TRIPLET	REFERENCE NUMBER	SOURCE
Left F1	DGSTLRR	GCC	-	CoDA
Left F2	DSSVLRR	GCC	-	CoDA
Left F3	ERRGLAR	GCC	-	CoDA
Right F1	RKHHLGR	GGG	-	CoDA
Right F2	RREHLVR	GGG	-	CoDA
Right F3	RNDKLVP	GGG	-	CoDA

Bos taurus (cow) Build 3.1

ZF DNA Sequence

Blast GGCGGCGGCNNNNNGGGGGGGGG

ZFN-unknown-SP-7-48
7161 cGGCGGCGGCGGGGGGGGGGGGGGGAa 7187
7161 gCCGCCGCCGCCCCCCCCCCCCCCCTt 7187

FINGER	HELIX	TRIPLET	REFERENCE NUMBER	SOURCE
Left F1	DGSTLRR	GCC	-	CoDA
Left F2	DSSVLRR	GCC	-	CoDA
Left F3	ERRGLAR	GCC	-	CoDA
Right F1	TSAHLAR	GGA	-	CoDA
Right F2	RREHLVR	GGG	-	CoDA
Right F3	RNDKLVP	GGG	-	CoDA

ZF DNA Sequence

Bos taurus (cow) Build 3.1

Blast GGCGGCGGCNNNNNNNGGGGGGGGA

ZFN-unknown-SP-6-46
7161 cGGCGGCGGCGGGGGGGGGGGGGGGa 7186
7161 gCCGCCGCCGCCCCCCCCCCCCCCCt 7186

FINGER	HELIX	TRIPLET	REFERENCE NUMBER	SOURCE
Left F1	DGSTLRR	GCC	-	CoDA
Left F2	DSSVLRR	GCC	-	CoDA
Left F3	ERRGLAR	GCC	-	CoDA
Right F1	RKHHLGR	GGG	-	CoDA
Right F2	RREHLVR	GGG	-	CoDA
Right F3	RNDKLVP	GGG	-	CoDA

ZF DNA Sequence

Bos taurus (cow) Build 3.1

Blast GGCGGCGGCNNNNNNGGGGGGGGG

ZFN-unknown-SP-5-35
7355 gGGCCCCCGCGCTCCGCCGGGGGCc 7379
7355 cCCGGGGGCGCGAGGCGGCCCCCGg 7379

FINGER	HELIX	TRIPLET	REFERENCE NUMBER	SOURCE
Left F1	SHTVLTR	GCC	-	CoDA
Left F2	RREHLVR	GGG	-	CoDA
Left F3	RTDSLPR	GCG	-	CoDA
Right F1	TNSKLTR	GGC	-	CoDA
Right F2	RREHLVR	GGG	-	CoDA
Right F3	EHRGLKR	GCC	-	CoDA

ZF DNA Sequence

Bos taurus (cow) Build 3.1

Blast GGCCCCCGCNNNNNGCCGGGGGC

ZFN-unknown-SP-5-36

7401 gGACGCCTTCCGCCCGGCGCCGGGc 7425

7401 cCTGCGGAAGGCGGGCCGCGGCCCg 7425

FINGER	HELIX	TRIPLET	REFERENCE NUMBER	SOURCE
Left F1	TGAVLTR	GTC	-	CoDA
Left F2	LKEHLTR	GGC	-	CoDA
Left F3	QTNNLTR	GAA	-	CoDA
Right F1	KGDHLRR	GGG	-	CoDA
Right F2	DSSVLRR	GCC	-	CoDA
Right F3	ENSKLNR	GGC	-	CoDA

ZF DNA Sequence

Bos taurus (cow) Build 3.1

Blast GACGCCTTCNNNNNGGCGCCGGG

ZFN-unknown-SP-7-49

7495 gCGCGGCGCCGGAGGGGCGGCCGCCg 7521

7495 cGCGCCGCGGCCTCCCCCGCCGGCGGc 7521

FINGER	HELIX	TRIPLET	REFERENCE NUMBER	SOURCE
Left F1	RLRDLPR	GCG	-	CoDA
Left F2	DSSVLRR	GCC	-	CoDA
Left F3	ENSKLNR	GGC	-	CoDA
Right F1	DGSTLRR	GCC	-	CoDA
Right F2	DSSVLRR	GCC	-	CoDA
Right F3	RVDDLGR	GCG	-	CoDA

ZF DNA Sequence

Bos taurus (cow) Build 3.1

Blast CGCGGCGCCNNNNNNNGCGGCCGCC

ZFN-unknown-SP-6-47

7510 gGGCGGCCGCCGAGGTGCGGGGGCCc 7535

7510 cCCGCCGGCGGCTCCACGCCCCCGGg 7535

FINGER	HELIX	TRIPLET	REFERENCE NUMBER	SOURCE
Left F1	DGSTLRR	GCC	-	CoDA
Left F2	DSSVLRR	GCC	-	CoDA
Left F3	RVDDLGR	GCG	-	CoDA
Right F1	SHTVLTR	GCC	-	CoDA
Right F2	RREHLVR	GGG	-	CoDA
Right F3	RTDSLPR	GCG	-	CoDA

ZF DNA Sequence
Bos taurus (cow) Build 3.1

Blast GGCGGCCGCNNNNNNGCGGGGGCC

ZFN-unknown-SP-5-37
7510 gGGCGGCCGCCGAGGTGCGGGGGCc 7534
7510 cCCGCCGGCGGCTCCACGCCCCCGg 7534

FINGER	HELIX	TRIPLET	REFERENCE NUMBER	SOURCE
Left F1	DGSTLRR	GCC	-	CoDA
Left F2	DSSVLRR	GCC	-	CoDA
Left F3	RVDDLGR	GCG	-	CoDA
Right F1	TNSKLTR	GGC	-	CoDA
Right F2	RREHLVR	GGG	-	CoDA
Right F3	ANRTLVH	TGC	-	CoDA

ZF DNA Sequence
Bos taurus (cow) Build 3.1

Blast GGCGGCCGCNNNNNTGCGGGGGC
