# Supplementary material for: Identity of zinc finger nucleases with specificity to herpes simplex virus type II genomic DNA: novel HSV-2 vaccine/therapy precursors
Source: Theor Biol Med Model. 2011 Jun 24;8:23. doi: 10.1186/1742-4682-8-23 (PMC3138452; doi:10.1186/1742-4682-8-23)
Supplement: Additional file 5 — A list of the -1 to 6 recognition domians of the alpha-helix for some of the ZFN cleaving within the 3,094.9 bp at the 3' end of the HSV-2 genome. This file lists details of the -1 to 6 recognition domians (denoted F1, F2, F3/F3, F2, F1) of the alpha-helix for some of the ZFN cleaving within the 3,094.9 bp at the 3' end of the HSV-2 genome. [file 1742-4682-8-23-S5.PDF]

Zinc Finger Site Type: Nuclease
Zinc Finger Engineering Method: CoDA
Sequence Name : HSV-2 Genomic
Sequence Length:154748
Nucleotide Sequence
Sort By: Position ☐ **Hide intron splice sites**

⊞ ZFN-unknown-SP-6-669
152201 cCGCCCCGCCGACGCCGCCGCCGCGg 152226
152201 gGCGGGGCGGCTGCGGCGGCGGCGCc 152226

FINGER	HELIX	TRIPLET	REFERENCE NUMBER	SOURCE
Left F1	KRHTLTR	GCG	-	CoDA
Left F2	RREHLVR	GGG	-	CoDA
Left F3	ESGHLKR	GGC	-	CoDA
Right F1	RLRDLPR	GCG	-	CoDA
Right F2	DSSVLRR	GCC	-	CoDA
Right F3	ERRGLAR	GCC	-	CoDA

ZF DNA Sequence Bos taurus (cow) Build 3.1
Blast CGCCCCGCCNNNNNNGCCGCCGCG

⊞ ZFN-unknown-SP-7-602
152203 gCCCCGCCGACGCCGCCGCCGCGGCCg
152229
152203 cGGGGCGGCTGCGGCGGCGGCGCCGGc
152229

FINGER	HELIX	TRIPLET	REFERENCE NUMBER	SOURCE
Left F1	RGNHLRR	GGG	-	CoDA
Left F2	RTDTLAR	GCG	-	CoDA
Left F3	RSDTLPL	TCG	-	CoDA
Right F1	DPSTLRR	GCC	-	CoDA
Right F2	RTDTLAR	GCG	-	CoDA
Right F3	ERRGLAR	GCC	-	CoDA

ZF DNA Sequence Bos taurus (cow) Build 3.1
Blast CCCCGCCGA NNNNNNGCCGCGGCC

⊞ ZFN-unknown-SP-6-670
152204 cCCCGCCGACGCCGCCGCCGCGGCCg
152229

152204 gGGGCGGCTGCGGCGGCGGCGCCGGc
152229

FINGER	HELIX	TRIPLET	REFERENCE NUMBER	SOURCE
Left F1	RKHHLGR	GGG	-	CoDA
Left F2	LKEHLTR	GGC	-	CoDA
Left F3	DPTSLNR	GTC	-	CoDA
Right F1	DPSTLRR	GCC	-	CoDA
Right F2	RTDTLAR	GCG	-	CoDA
Right F3	ERRGLAR	GCC	-	CoDA

ZF DNA Sequence Bos taurus (cow) Build 3.1

Blast CCCGCCGACNNNNNNGCCGCGGCC

ZFN-unknown-SP-7-603
152209 cCGACGCCGCCGCCGCGGCCGCCGCGc
152235
152209 gGCTGCGGCGGCGGCGCCGGCGGCGCg
152235

FINGER	HELIX	TRIPLET	REFERENCE NUMBER	SOURCE
Left F1	KNNDLLK	TCG	-	CoDA
Left F2	RTDTLAR	GCG	-	CoDA
Left F3	RVDDLGR	GCG	-	CoDA
Right F1	RLRDLPR	GCG	-	CoDA
Right F2	DSSVLRR	GCC	-	CoDA
Right F3	ERRGLAR	GCC	-	CoDA

ZF DNA Sequence Bos taurus (cow) Build 3.1

Blast CGACGCCGCNNNNNNGCCGCCGCG

ZFN-unknown-SP-7-604
152224 cGGCCGCCGCGCCCCGCGGCGCTGACc
152250
152224 gCCGGCGGCGCGGGGCGCCGCGACTGg
152250

FINGER	HELIX	TRIPLET	REFERENCE NUMBER	SOURCE
Left F1	DPSTLRR	GCC	-	CoDA
Left F2	RTDTLAR	GCG	-	CoDA
Left F3	RVDDLGR	GCG	-	CoDA
Right F1	DLSNLKR	GAC	-	CoDA
Right F2	QRSDLTR	GCT	-	CoDA
Right F3	ESGHLRR	GGC	-	CoDA

Bos taurus (cow) Build 3.1

ZF DNA Sequence

Blast GGCCGCCGCNNNNNNNGGCGCTGAC

ZFN-unknown-SP-7-605
152265 gGGCCCCGACCCGCAGGGCGGCTGGCg
152291
152265 cCCGGGGCTGGGCGTCCCGCCGACCGc
152291

FINGER	HELIX	TRIPLET	REFERENCE NUMBER	SOURCE
Left F1	SHTVLTR	GCC	-	CoDA
Left F2	RREHLVR	GGG	-	CoDA
Left F3	DPTSLNR	GTC	-	CoDA
Right F1	APSKLAR	GGC	-	CoDA
Right F2	QRSDLTR	GCT	-	CoDA
Right F3	RLDMLAR	GCG	-	CoDA

Bos taurus (cow) Build 3.1

ZF DNA Sequence

Blast GGCCCCGACNNNNNNNGCGGCTGGC

ZFN-unknown-SP-7-606
152315 aCACGCCGGCGCCCTCGGCCGCCGCCc
152341
152315 tGTGCGGCCGCGGGAGCCGGCGGCGGg
152341

FINGER	HELIX	TRIPLET	REFERENCE NUMBER	SOURCE
Left F1	SRFTLGR	GTG	-	CoDA
Left F2	LKEHLTR	GGC	-	CoDA
Left F3	DPSNLRR	GCC	-	CoDA
Right F1	DGSTLRR	GCC	-	CoDA
Right F2	DSSVLRR	GCC	-	CoDA
Right F3	ERRGLAR	GCC	-	CoDA

Bos taurus (cow) Build 3.1

ZF DNA Sequence

Blast CACGCCGGCNNNNNNNGCCGCCGCC

ZFN-unknown-SP-6-671
152349 cTACTGCGCCCCGCGGGCCGTGGCCg
152374
152349 gATGACGCGGGGCGCCCGGCACCGGc
152374

FINGER	HELIX	TRIPLET	REFERENCE NUMBER	SOURCE

Left F1	QKQALTR	GTA	-	CoDA
Left F2	QSTTLKR	GCA	-	CoDA
Left F3	DGGHLTR	GGC	-	CoDA
Right F1	KRRDLDR	GCC	-	CoDA
Right F2	RREVLEN	GTG	-	CoDA
Right F3	ERRGLHR	GCC	-	CoDA

ZF DNA Sequence Bos taurus (cow) Build 3.1

Blast TACTGCGCCNNNNNNGCCGTGGCC

ZFN-unknown-SP-5-544
152518 gGCCCGCTGCGCCGCGCGGCGGCCt 152542
152518 cCGGGCGACGCGGCGCGCCGCCGGa 152542

FINGER	HELIX	TRIPLET	REFERENCE NUMBER	SOURCE
Left F1	VPSKLAR	GGC	-	CoDA
Left F2	RTDTLAR	GCG	-	CoDA
Left F3	QKGTLGR	GCA	-	CoDA
Right F1	DPSTLRR	GCC	-	CoDA
Right F2	RTDTLAR	GCG	-	CoDA
Right F3	RVDDLGR	GCG	-	CoDA

ZF DNA Sequence Bos taurus (cow) Build 3.1

Blast GCCCGCTGCNNNNNGCGGCGGCC

ZFN-unknown-SP-7-607
152714 gGCCCGCCACGGCCGCCTGGGCGGGCa
152740
152714 cCGGGCGGTGCCGGCGGACCCGCCCGt
152740

FINGER	HELIX	TRIPLET	REFERENCE NUMBER	SOURCE
Left F1	VPSKLAR	GGC	-	CoDA
Left F2	RTDTLAR	GCG	-	CoDA
Left F3	RKTALNR	GTG	-	CoDA
Right F1	VPSKLAR	GGC	-	CoDA
Right F2	RTDTLAR	GCG	-	CoDA
Right F3	RSDHLSL	TGG	-	CoDA

ZF DNA Sequence Bos taurus (cow) Build 3.1

Blast GCCCGCCACNNNNNNNTGGGCGGGC

ZFN-unknown-SP-6-672

152750 gCGCCCCCGACGTCTCGGCGCTGGGc
152775
152750 cGCGGGGGCTGCAGAGCCGCGACCCg
152775

FINGER	HELIX	TRIPLET	REFERENCE NUMBER	SOURCE
Left F1	KRHTLTR	GCG	-	CoDA
Left F2	RREHLVR	GGG	-	CoDA
Left F3	RMDSLGG	TCG	-	CoDA
Right F1	KKDHLHR	GGG	-	CoDA
Right F2	QRSDLTR	GCT	-	CoDA
Right F3	ESGHLRR	GGC	-	CoDA

ZF DNA Sequence Bos taurus (cow) Build 3.1

Blast CGCCCCCGA NNNNNNGGCGCTGGG

ZFN-unknown-SP-5-545
152751 cGCCCCCGACGTCTCGGCGCTGGGc 152775
152751 gCGGGGGCTGCAGAGCCGCGACCCg 152775

FINGER	HELIX	TRIPLET	REFERENCE NUMBER	SOURCE
Left F1	TNSKLTR	GGC	-	CoDA
Left F2	RREHLVR	GGG	-	CoDA
Left F3	DPTSLNR	GTC	-	CoDA
Right F1	KKDHLHR	GGG	-	CoDA
Right F2	QRSDLTR	GCT	-	CoDA
Right F3	ESGHLRR	GGC	-	CoDA

ZF DNA Sequence Bos taurus (cow) Build 3.1

Blast GCCCCCGA CNNNNNGGCGCTGGG

ZFN-unknown-SP-6-673
152877 cGTCAACGCCGTGCGCGCCGCGGACt
152902
152877 gCAGTTGCGGCACGCGCGGCGCCTGa
152902

FINGER	HELIX	TRIPLET	REFERENCE NUMBER	SOURCE
Left F1	EQANLRR	GAC	-	CoDA
Left F2	HKSSLTR	GTT	-	CoDA
Left F3	ESGHLKR	GGC	-	CoDA
Right F1	DPSNLIR	GAC	-	CoDA
Right F2	RTDTLAR	GCG	-	CoDA
Right F3	ERRGLAR	GCC	-	CoDA

ZF DNA Sequence Bos taurus (cow) Build 3.1

Blast GTCAACGCCNNNNNNGCCGCGGAC

ZFN-unknown-SP-7-608
152891 gCGCCGCGGACTGGCCCGCCGACGGGc
152917
152891 cGCGGCGCCTGACCGGGCGGCTGCCCg
152917

FINGER	HELIX	TRIPLET	REFERENCE NUMBER	SOURCE
Left F1	RSNTLLR	GCG	-	CoDA
Left F2	RTDTLAR	GCG	-	CoDA
Left F3	DKRSLPH	TCC	-	CoDA
Right F1	KKDHLHR	GGG	-	CoDA
Right F2	DRGNLTR	GAC	-	CoDA
Right F3	DKSVLAR	GCC	-	CoDA

ZF DNA Sequence Bos taurus (cow) Build 3.1

Blast CGCCGCGGANNNNNNNGCCGACGGG

ZFN-unknown-SP-7-609
152933 aGCACGCCTACCTGGCCTGCGAGGTGc
152959
152933 tCGTGCGGATGGACCGGACGCTCCACg
152959

FINGER	HELIX	TRIPLET	REFERENCE NUMBER	SOURCE
Left F1	RSRNLTL	TGC	-	CoDA
Left F2	RTDTLAR	GCG	-	CoDA
Left F3	RRDHLSP	TAG	-	CoDA
Right F1	RRHILDR	GTG	-	CoDA
Right F2	RQDNLGR	GAG	-	CoDA
Right F3	ANRTLVH	TGC	-	CoDA

ZF DNA Sequence Bos taurus (cow) Build 3.1

Blast GCACGCCTANNNNNNNTGCGAGGTG

ZFN-unknown-SP-6-674
152934 gCACGCCTACCTGGCCTGCGAGGTGc
152959
152934 cGTGCGGATGGACCGGACGCTCCACg
152959

FINGER	HELIX	TRIPLET	REFERENCE NUMBER	SOURCE

Left F1	SRFTLGR	GTG	-	CoDA
Left F2	LKEHLTR	GGC	-	CoDA
Left F3	QTQSLQR	GTA	-	CoDA
Right F1	RRHILDR	GTG	-	CoDA
Right F2	RQDNLGR	GAG	-	CoDA
Right F3	ANRTLVH	TGC	-	CoDA

Bos taurus (cow) Build 3.1

ZF DNA Sequence

Blast CACGCCTACNNNNNNTGCGAGGTG

ZFN-unknown-SP-5-546
153084 gTACCCCGACGCGCCGCCGCTGCGc 153108
153084 cATGGGGCTGCGCGGCGGCGACGCg 153108

FINGER	HELIX	TRIPLET	REFERENCE NUMBER	SOURCE
Left F1	QQSSLLR	GTA	-	CoDA
Left F2	RREHLVR	GGG	-	CoDA
Left F3	DPTSLNR	GTC	-	CoDA
Right F1	RKGTLDR	GCG	-	CoDA
Right F2	QRSDLTR	GCT	-	CoDA
Right F3	DPSNLRR	GCC	-	CoDA

Bos taurus (cow) Build 3.1

ZF DNA Sequence

Blast TACCCCGACNNNNNGCCGCTGCG

ZFN-unknown-SP-6-675
153097 cCGCCGCTGCGCCTCTGCCGCGGGGc
153122
153097 gGCGGCGACGCGGAGACGGCGCCCCg
153122

FINGER	HELIX	TRIPLET	REFERENCE NUMBER	SOURCE
Left F1	RSNTLLR	GCG	-	CoDA
Left F2	RTDTLAR	GCG	-	CoDA
Left F3	QKGTLGR	GCA	-	CoDA
Right F1	RGNHLRR	GGG	-	CoDA
Right F2	RTDTLAR	GCG	-	CoDA
Right F3	ERRGLAR	GCC	-	CoDA

Bos taurus (cow) Build 3.1

ZF DNA Sequence

Blast CGCCGCTGCNNNNNNGCCGCGGGG

ZFN-unknown-SP-5-547

153150 cTTCGGCCCCGACACGCTGGTGCCc 153174
153150 gAAGCCGGGGCTGTGCGACCACGGg 153174

FINGER	HELIX	TRIPLET	REFERENCE NUMBER	SOURCE
Left F1	QASNLAR	GAA	-	CoDA
Left F2	DSSVLRR	GCC	-	CoDA
Left F3	RTEHLAR	GGG	-	CoDA
Right F1	SKKSLTR	GCC	-	CoDA
Right F2	EAHHLSR	GGT	-	CoDA
Right F3	EGSGLKR	GCT	-	CoDA

ZF DNA Sequence
Bos taurus (cow) Build 3.1
Blast TTCGGCCCCNNNNNGCTGGTGCC

ZFN-unknown-SP-7-610
153224 gGGCCGCCGCCTCGGGCGCGGGCGACg
153250
153224 cCCGGCGGCGGAGCCCGCGCCCGCTGc
153250

FINGER	HELIX	TRIPLET	REFERENCE NUMBER	SOURCE
Left F1	DPSTLRR	GCC	-	CoDA
Left F2	RTDTLAR	GCG	-	CoDA
Left F3	RVDDLGR	GCG	-	CoDA
Right F1	DPSNLRR	GAC	-	CoDA
Right F2	LKEHLTR	GGC	-	CoDA
Right F3	RKDGLTR	GCG	-	CoDA

ZF DNA Sequence
Bos taurus (cow) Build 3.1
Blast GGCCGCCGCNNNNNNNGCGGGCGAC

ZFN-unknown-SP-7-611
153297 gCACCGCGCCTGCGCGCGCTGGGGCCt
153323
153297 cGTGGCGCGGACGCGCGCGACCCCGGa
153323

FINGER	HELIX	TRIPLET	REFERENCE NUMBER	SOURCE
Left F1	RNFILAR	GTG	-	CoDA
Left F2	RTDTLAR	GCG	-	CoDA
Left F3	ESGHLKR	GGC	-	CoDA
Right F1	SHTVLTR	GCC	-	CoDA
Right F2	RREHLVR	GGG	-	CoDA
Right F3	VSNSLAR	GCT	-	CoDA

Bos taurus (cow) Build 3.1

ZF DNA Sequence

Blast CACCGCGCCNNNNNNNGCTGGGGCC

ZFN-unknown-SP-7-612
153339 gCCCGTCTACGTGGCGCTGGGGCGCGa
153365
153339 cGGGCAGATGCACCGCGACCCCGCGCt
153365

FINGER	HELIX	TRIPLET	REFERENCE NUMBER	SOURCE
Left F1	KKDHLHR	GGG	-	CoDA
Left F2	DRGNLTR	GAC	-	CoDA
Left F3	QSTSLQR	GTA	-	CoDA
Right F1	RRHGLDR	GCG	-	CoDA
Right F2	LKEHLTR	GGC	-	CoDA
Right F3	RTESLHI	TGG	-	CoDA

Bos taurus (cow) Build 3.1

ZF DNA Sequence

Blast CCCGTCTACNNNNNNNTGGGGCGCG

ZFN-unknown-SP-5-548
153339 gCCCGTCTACGTGGCGCTGGGGCGc 153363
153339 cGGGCAGATGCACCGCGACCCCGCg 153363

FINGER	HELIX	TRIPLET	REFERENCE NUMBER	SOURCE
Left F1	KKDHLHR	GGG	-	CoDA
Left F2	DRGNLTR	GAC	-	CoDA
Left F3	QSTSLQR	GTA	-	CoDA
Right F1	KRHTLTR	GCG	-	CoDA
Right F2	RREHLVR	GGG	-	CoDA
Right F3	VSNSLAR	GCT	-	CoDA

Bos taurus (cow) Build 3.1

ZF DNA Sequence

Blast CCCGTCTACNNNNNGCTGGGGCG

ZFN-unknown-SP-6-676
153443 gCGACGCGCCCCCGCTGGTGCTGCGc
153468
153443 cGCTGCGCGGGGGCGACCACGACGCg
153468

FINGER	HELIX	TRIPLET	REFERENCE NUMBER	SOURCE
Left F1	KNNDLLK	TCG	-	CoDA

Left F2	RTDTLAR	GCG	-	CoDA
Left F3	ESGHLKR	GGC	-	CoDA
Right F1	RKGTLDR	GCG	-	CoDA
Right F2	QRSDLTR	GCT	-	CoDA
Right F3	HGHRLKT	GGT	-	CoDA

ZF DNA Sequence

Bos taurus (cow) Build 3.1

Blast CGACGCGCCNNNNNNGGTGCTGCG

ZFN-unknown-SP-6-677
153448 gCGCCCCCGCTGGTGCTGCGCGACGa
153473
153448 cGCGGGGGCGACCACGACGCGCTGCt
153473

FINGER	HELIX	TRIPLET	REFERENCE NUMBER	SOURCE
Left F1	KRHTLTR	GCG	-	CoDA
Left F2	RREHLVR	GGG	-	CoDA
Left F3	RTDSLPR	GCG	-	CoDA
Right F1	RSQTLAQ	ACG	-	CoDA
Right F2	RTDTLAR	GCG	-	CoDA
Right F3	QRRSLGH	TGC	-	CoDA

ZF DNA Sequence

Bos taurus (cow) Build 3.1

Blast CGCCCCCGCNNNNNNTGCGCGACG

ZFN-unknown-SP-7-613
153488 cGCCCCCGCAGATACGCTGGGCGTCGg
153514
153488 gCGGGGGCGTCTATGCGACCCGCAGCc
153514

FINGER	HELIX	TRIPLET	REFERENCE NUMBER	SOURCE
Left F1	TNSKLTR	GGC	-	CoDA
Left F2	RREHLVR	GGG	-	CoDA
Left F3	ANRTLVH	TGC	-	CoDA
Right F1	KNNDLLK	TCG	-	CoDA
Right F2	RTDTLAR	GCG	-	CoDA
Right F3	RSDHLSL	TGG	-	CoDA

ZF DNA Sequence

Bos taurus (cow) Build 3.1

Blast GCCCCCGCANNNNNNNTGGGCGTCG

ZFN-unknown-SP-7-614

153509 cGTCGGCCGCGGGCCGCGCGGGGACGg
153535
153509 gCAGCCGGCGCCCGGCGCGCCCCTGCc
153535

FINGER	HELIX	TRIPLET	REFERENCE NUMBER	SOURCE
Left F1	DPSNLRR	GAC	-	CoDA
Left F2	DSSVLRR	GCC	-	CoDA
Left F3	RVDDLGR	GCG	-	CoDA
Right F1	KNNDLTR	ACG	-	CoDA
Right F2	RREHLVR	GGG	-	CoDA
Right F3	RTDSLPR	GCG	-	CoDA

ZF DNA Sequence

Bos taurus (cow) Build 3.1

Blast GTCGGCCGCNNNNNNNGCGGGGACG

ZFN-unknown-SP-5-549
153549 gGGCGGCGGCGTGGAGGTGGTGGGg 153573
153549 cCCGCCGCCGCACCTCCACCACCCc 153573

FINGER	HELIX	TRIPLET	REFERENCE NUMBER	SOURCE
Left F1	DGSTLRR	GCC	-	CoDA
Left F2	DSSVLRR	GCC	-	CoDA
Left F3	ERRGLAR	GCC	-	CoDA
Right F1	KGDHLRR	GGG	-	CoDA
Right F2	EAHHLSR	GGT	-	CoDA
Right F3	IRHHLKR	GGT	-	CoDA

ZF DNA Sequence

Bos taurus (cow) Build 3.1

Blast GGCGGCGGCNNNNNGGTGGTGGG

ZFN-unknown-SP-6-678
153549 gGGCGGCGGCGTGGAGGTGGTGGGGa
153574
153549 cCCGCCGCCGCACCTCCACCACCCCt
153574

FINGER	HELIX	TRIPLET	REFERENCE NUMBER	SOURCE
Left F1	DGSTLRR	GCC	-	CoDA
Left F2	DSSVLRR	GCC	-	CoDA
Left F3	ERRGLAR	GCC	-	CoDA
Right F1	KKDHLHR	GGG	-	CoDA
Right F2	RREVLEN	GTG	-	CoDA
Right F3	RKDALHV	GTG	-	CoDA

ZF DNA Sequence Bos taurus (cow) Build 3.1

Blast GGCGGCGGCNNNNNNGTGGTGGGG

ZFN-unknown-SP-7-615
154070 gCACGTCTCCCGCGCCCGCGGGGGGTc
154096
154070 cGTGCAGAGGGCGCGGGCGCCCCCCAg
154096

FINGER	HELIX	TRIPLET	REFERENCE NUMBER	SOURCE
Left F1	RASVLDI	GTG	-	CoDA
Left F2	DRGNLTR	GAC	-	CoDA
Left F3	QGGHLKR	GGA	-	CoDA
Right F1	RQSRLQR	GGT	-	CoDA
Right F2	RREHLVR	GGG	-	CoDA
Right F3	RTDSLPR	GCG	-	CoDA

ZF DNA Sequence Bos taurus (cow) Build 3.1

Blast CACGTCTCCNNNNNNNGCGGGGGGT

ZFN-unknown-SP-6-679
154140 gAGCCCCCCCGCAGGAGCGGGAGGGa
154165
154140 cTCGGGGGGGCGTCCTCGCCCTCCCt
154165

FINGER	HELIX	TRIPLET	REFERENCE NUMBER	SOURCE
Left F1	THSMLAR	GCT	-	CoDA
Left F2	RREHLVR	GGG	-	CoDA
Left F3	RNDKLVP	GGG	-	CoDA
Right F1	KKDHLHR	GGG	-	CoDA
Right F2	QSAHLKR	GGA	-	CoDA
Right F3	REDSLPR	GCG	-	CoDA

ZF DNA Sequence Bos taurus (cow) Build 3.1

Blast AGCCCCCCCNNNNNNGCGGGAGGG

ZFN-unknown-SP-7-616
154143 cCCCCCCGCAGGAGCGGGAGGGAAGGc
154169
154143 gGGGGGGCGTCCTCGCCCTCCCTTCCg
154169

FINGER	HELIX	TRIPLET	REFERENCE NUMBER	SOURCE

Left F1	RKHHLGR	GGG	-	CoDA
Left F2	RREHLVR	GGG	-	CoDA
Left F3	ANRTLVH	TGC	-	CoDA
Right F1	RPHHLDA	AGG	-	CoDA
Right F2	QSAHLKR	GGA	-	CoDA
Right F3	VHWNLMR	GAG	-	CoDA

ZF DNA Sequence

Bos taurus (cow) Build 3.1

Blast CCCCCCGCANNNNNNNGAGGGAAGG

ZFN-unknown-SP-6-680
154188 gGGCTGCTGCGAGCTCGGGGCCGCGg
154213
154188 cCCGACGACGCTCGAGCCCCGGCGCc
154213

FINGER	HELIX	TRIPLET	REFERENCE NUMBER	SOURCE
Left F1	DPSTLRR	GCC	-	CoDA
Left F2	QSTTLKR	GCA	-	CoDA
Left F3	QPNTLTR	GCA	-	CoDA
Right F1	RLRDLPR	GCG	-	CoDA
Right F2	DSSVLRR	GCC	-	CoDA
Right F3	RTEHLAR	GGG	-	CoDA

ZF DNA Sequence

Bos taurus (cow) Build 3.1

Blast GGCTGCTGCNNNNNNGGGGCCGCG

ZFN-unknown-SP-7-617
154260 aGGCGGCCGCGGGGGAGGCGGCCGCGg
154286
154260 tCCGCCGGCGCCCCCTCCGCCGGCGCc
154286

FINGER	HELIX	TRIPLET	REFERENCE NUMBER	SOURCE
Left F1	DGSTLRR	GCC	-	CoDA
Left F2	DSSVLRR	GCC	-	CoDA
Left F3	RVDDLGR	GCG	-	CoDA
Right F1	RLRDLPR	GCG	-	CoDA
Right F2	DSSVLRR	GCC	-	CoDA
Right F3	RVDDLGR	GCG	-	CoDA

ZF DNA Sequence

Bos taurus (cow) Build 3.1

Blast GGCGGCCGCNNNNNNNGCGGCCGCG

ZFN-unknown-SP-7-618
154289 cCGCAGCCCCGTGGCGCGCGGGGGGGa
154315
154289 gGCGTCGGGGCACCGCGCGCCCCCCCt
154315

FINGER	HELIX	TRIPLET	REFERENCE NUMBER	SOURCE
Left F1	RKGTLDR	GCG	-	CoDA
Left F2	QRSDLTR	GCT	-	CoDA
Left F3	RTEHLAR	GGG	-	CoDA
Right F1	RKHHLGR	GGG	-	CoDA
Right F2	RREHLVR	GGG	-	CoDA
Right F3	RTDSLPR	GCG	-	CoDA

ZF DNA Sequence

Bos taurus (cow) Build 3.1

Blast CGCAGCCCCNNNNNNNGCGGGGGGG

ZFN-unknown-SP-5-550
154433 tGGCGTCTTCGGGGGGGCGGGGAGc 154457
154433 aCCGCAGAAGCCCCCCCGCCCCTCg 154457

FINGER	HELIX	TRIPLET	REFERENCE NUMBER	SOURCE
Left F1	ERRGLDR	GCC	-	CoDA
Left F2	DRGNLTR	GAC	-	CoDA
Left F3	QSNNLNR	GAA	-	CoDA
Right F1	RNTNLTR	GAG	-	CoDA
Right F2	RREHLVR	GGG	-	CoDA
Right F3	ESGHLKR	GGC	-	CoDA

ZF DNA Sequence

Bos taurus (cow) Build 3.1

Blast GGCGTCTTCNNNNNGGCGGGGAG

ZFN-unknown-SP-7-619
154493 aGTCCCCGTCCTGCCGCGCGGGGGCGg
154519
154493 tCAGGGGCAGGACGGCGCGCCCCCGCc
154519

FINGER	HELIX	TRIPLET	REFERENCE NUMBER	SOURCE
Left F1	EEANLRR	GAC	-	CoDA
Left F2	RREHLVR	GGG	-	CoDA
Left F3	DPSNLQR	GAC	-	CoDA
Right F1	KRHTLTR	GCG	-	CoDA
Right F2	RREHLVR	GGG	-	CoDA

Right F3	RTDSLPR	GCG	–	CoDA

ZF DNA Sequence

Bos taurus (cow) Build 3.1

Blast GTCCCCGTCNNNNNNNGCGGGGGCG

ZFN-unknown-SP-6-681
154500 gTCCTGCCGCGCGGGGGCGGGCGCGg
154525
154500 cAGGACGGCGCGCCCCCGCCCGCGCc
154525

FINGER	HELIX	TRIPLET	REFERENCE NUMBER	SOURCE
Left F1	RTDRLIR	GGA	–	CoDA
Left F2	QSTTLKR	GCA	–	CoDA
Left F3	RLDMLAR	GCG	–	CoDA
Right F1	RRHGLDR	GCG	–	CoDA
Right F2	LKEHLTR	GGC	–	CoDA
Right F3	RKDGLTR	GCG	–	CoDA

ZF DNA Sequence

Bos taurus (cow) Build 3.1

Blast TCCTGCCGCNNNNNNGCGGGCGCG

ZFN-unknown-SP-7-620
154556 aGGCAGCCCCGCGGCGCGCGGGGGGAg
154582
154556 tCCGTCGGGGCGCCGCGCGCCCCCCTc
154582

FINGER	HELIX	TRIPLET	REFERENCE NUMBER	SOURCE
Left F1	DRRTLDR	GCC	–	CoDA
Left F2	QRSDLTR	GCT	–	CoDA
Left F3	RTEHLAR	GGG	–	CoDA
Right F1	TSAHLAR	GGA	–	CoDA
Right F2	RREHLVR	GGG	–	CoDA
Right F3	RTDSLPR	GCG	–	CoDA

ZF DNA Sequence

Bos taurus (cow) Build 3.1

Blast GGCAGCCCCNNNNNNNGCGGGGGGA

ZFN-unknown-SP-5-551
154561 gCCCCGCGGCGCGCGGGGGGAGGGg 154585
154561 cGGGGCGCCGCGCGCCCCCCTCCCc 154585

FINGER	HELIX	TRIPLET	REFERENCE NUMBER	SOURCE

Left F1	RGNHLRR	GGG	-	CoDA
Left F2	RTDTLAR	GCG	-	CoDA
Left F3	ERRGLAR	GCC	-	CoDA
Right F1	KKDHLHR	GGG	-	CoDA
Right F2	QSAHLKR	GGA	-	CoDA
Right F3	RTEHLAR	GGG	-	CoDA

Bos taurus (cow) Build 3.1

ZF DNA Sequence

Blast CCCCGCGGCNNNNNGGGGGAGGG

ZFN-unknown-SP-6-682
154561 gCCCCGCGGCGCGCGGGGGGAGGGGc
154586
154561 cGGGGCGCCGCGCGCCCCCCTCCCCg
154586

FINGER	HELIX	TRIPLET	REFERENCE NUMBER	SOURCE
Left F1	RGNHLRR	GGG	-	CoDA
Left F2	RTDTLAR	GCG	-	CoDA
Left F3	ERRGLAR	GCC	-	CoDA
Right F1	RNTHLAR	GGG	-	CoDA
Right F2	RQDNLGR	GAG	-	CoDA
Right F3	RIDKLGG	GGG	-	CoDA

Bos taurus (cow) Build 3.1

ZF DNA Sequence

Blast CCCCGCGGCNNNNNNGGGGAGGGG

ZFN-unknown-SP-6-683
154583 gGGCGGCGCCCGCGGGGGAGCGGCCg
154608
154583 cCCGCCGCGGGCGCCCCCTCGCCGGc
154608

FINGER	HELIX	TRIPLET	REFERENCE NUMBER	SOURCE
Left F1	DGSTLRR	GCC	-	CoDA
Left F2	DSSVLRR	GCC	-	CoDA
Left F3	ENSKLNR	GGC	-	CoDA
Right F1	DPSTLRR	GCC	-	CoDA
Right F2	RTDTLAR	GCG	-	CoDA
Right F3	QTTHLSR	GGA	-	CoDA

Bos taurus (cow) Build 3.1

ZF DNA Sequence

Blast GGCGGCGCCNNNNNNGGAGCGGCC

ZFN-unknown-SP-6-684
154586 cGGCGCCCGCGGGGGAGCGGCCGGCt
154611
154586 gCCGCGGGCGCCCCCTCGCCGGCCGa
154611

FINGER	HELIX	TRIPLET	REFERENCE NUMBER	SOURCE
Left F1	SNKDLTR	GCC	-	CoDA
Left F2	LKEHLTR	GGC	-	CoDA
Left F3	RKDGLTR	GCG	-	CoDA
Right F1	TPSKLDR	GGC	-	CoDA
Right F2	DSSVLRR	GCC	-	CoDA
Right F3	RVDDLGR	GCG	-	CoDA

ZF DNA Sequence

Bos taurus (cow) Build 3.1

Blast GGCGCCCGCNNNNNNGCGGCCGGC

ZFN-unknown-SP-6-685
154647 gCCCTGCCGCCCGCCCGCCGCCGCCg
154672
154647 cGGGACGGCGGGCGGGCGGCGGCGGc
154672

FINGER	HELIX	TRIPLET	REFERENCE NUMBER	SOURCE
Left F1	RRAHLQN	GGG	-	CoDA
Left F2	QSTTLKR	GCA	-	CoDA
Left F3	RLDMLAR	GCG	-	CoDA
Right F1	DGSTLRR	GCC	-	CoDA
Right F2	DSSVLRR	GCC	-	CoDA
Right F3	ERRGLAR	GCC	-	CoDA

ZF DNA Sequence

Bos taurus (cow) Build 3.1

Blast CCCTGCCGCNNNNNNGCCGCCGCC

ZFN-unknown-SP-5-552
154651 tGCCGCCCGCCCGCCGCCGCCGCCc 154675
154651 aCGGCGGGCGGGCGGCGGCGGCGGg 154675
